# HK3 stimulates immune cell infiltration to promote glioma deterioration

**DOI:** 10.1186/s12935-023-03039-w

**Published:** 2023-10-01

**Authors:** Shupeng Li, Ziwei Li, Xinyu Wang, Junzhe Zhong, Daohan Yu, Hao Chen, Wenbin Ma, Lingling Liu, Minghuang Ye, Ruofei Shen, Chuanlu Jiang, Xiangqi Meng, Jinquan Cai

**Affiliations:** 1 Department of Neurosurgery, The Second Affiliated Hospital of Harbin Medical University, Harbin, China; 2 Department of Neurosurgery, The Dalian Municipal Central Hospital, Dalian, China; 3 Tiantan Hospital, Capital Medical University, Beijing, China; 4 Future Medical Laboratory, The Second Affiliated Hospital of Harbin Medical University, Harbin, China; 5 Department of Clinical Medical Record, The Second Affiliated Hospital of Harbin Medical University, Harbin, China; 6 The Sixth Affiliated Hospital of Harbin Medical University, Harbin, China

**Keywords:** HK3, Glycolysis, Immune infiltration, Glioma, Prognosis

## Abstract

**Background:**

Glioma is the most common and lethal type of brain tumor, and it is characterized by unfavorable prognosis and high recurrence rates. The reprogramming of energy metabolism and an immunosuppressive tumor microenvironment (TME) are two hallmarks of tumors. Complex and dynamic interactions between neoplastic cells and the surrounding microenvironment can generate an immunosuppressive TME, which can accelerate the malignant progression of glioma. Therefore, it is crucial to explore associations between energy metabolism and the immunosuppressive TME and to identify new biomarkers for glioma prognosis.

**Methods:**

In our work, we analyzed the co-expression relationship between glycolytic genes and immune checkpoints based on the transcriptomic data from The Cancer Genome Atlas (TCGA) and Chinese Glioma Genome Atlas (CGGA) and found the correlation between HK3 expression and glioma tumor immune status. To investigate the biological role of HK3 in glioma, we performed bioinformatics analysis and established a mouse glioblastoma (GBM) xenograft model.

**Results:**

Our study showed that HK3 significantly stimulated immune cell infiltration into the glioma TME. Tissue samples with higher HK3 expressive level showed increasing levels of immune cells infiltration, including M2 macrophages, neutrophils, and various subtypes of activated memory CD4^+^ T cells. Furthermore, HK3 expression was significantly increasing along with the elevated tumor grade, had a higher level in the mesenchymal subtype compared with those in other subtypes of GBM and could independently predict poor outcomes of GBM patients.

**Conclusion:**

The present work mainly concentrated on the biological role of HK3 in glioma and offered a novel insight of HK3 regulating the activation of immune cells in the glioma microenvironment. These findings could provide a new theoretical evidence for understanding the metabolic molecular within the glioma microenvironment and identifying new therapeutic targets.

**Supplementary Information:**

The online version contains supplementary material available at 10.1186/s12935-023-03039-w.

## Background

Glioma is the most prevalent malignant primary brain cancer in the central nervous system (CNS), which is associated with a median survival of 14.6 months after diagnosis [1, 2]. Although the approaches used in the treatment of glioma are constantly improving [3], the 5-year survival rate remains unsatisfactory [4]. In a recent study, explorations of tumor immunity and immunotherapy in glioma have resulted in new hope for patients [5]. The tumor microenvironment (TME) is an integrated part of a tumor which is consisted of tumor cells, endothelial cells, pericytes, fibroblasts and various immune cells [6]. The energy metabolism and dynamic interaction among these cells in TME can play crucial roles in many tumor-related pathological processes [7, 8]. In particular, the homeostasis of infiltrating immune cells in TME is closely involved in the occurrence, proliferation, deterioration, and metastasis of various tumors [9].

The reprogramming of energy metabolism and an immunosuppressive TME are two hallmarks of tumors [10]. On the one hand, tumor cells primarily use the glycolysis for energy consumption regardless of oxygen availability, and this is popularly known as the “Warburg effect” [11]. Aerobic glycolysis can produce up to 60% of ATP in cells [12]. This reprogramming of energy metabolism allows tumor cells to undergo large-scale biosynthesis and uninterrupted cancer growth, facilitates tumor cells to evade apoptosis, and produces a TME which is prone to metastasis [13, 14]. On the other hand, during the malignant progression of tumors, the body can generate an adaptive immune response to eliminate tumor antigens [15]. However, the infiltration of immune cells can also generate an immunosuppressive TME through complex mechanisms, which in turns facilitates tumor cell to evade from immune detection and attack [16]. Some studies have revealed that high number of immune cells, like M2 macrophages, neutrophils, or activated memory CD4 + T cells could cause poor outcomes in cancers [17–22]. In addition, PD-1 and its ligands, PD-L1 and PD-L2, are key regulators of immunosuppressive microenvironment that promotes tumor growth [23]. Therefore, studies on the tumor energy metabolism reprogramming and the immune cells in TME are important for exploring the biological role of tumor cells and tumor cell immune evasion. Recently, increasing researches have indicated that tumor glycolysis and tumor immune evasion are interdependent [24, 25]. For example, the competition between tumor cells and infiltrating CD8^+^ T cells induce high glucose consumption, altering metabolic microenvironment of T cells and stimulating tumor progression and immune evasion [26]. In addition, tumor and immune infiltrating cells reshape the metabolic modes to fit the hypoxic, aciditic, and low-nutrient microenvironment [27]. Thus, increasing the understanding of glycolysis in the immune evasion and biological progression of glioma has essential clinical translational significances for glioma diagnosis and treatment.

Hexokinases (HKs) are the key enzymes participating in the conversion of glucose to glucose 6-phosphate (G6P) in glycolysis [28], consisting of five isoenzymes (HK1, HK2, HK3, HK4, and HKDC1). These five members of the mammalian HK family with similar structure have tissue-specific expression [12]. Many work have proven that various malignant tumors have higher expression level of HK1 and HK2 [29, 30], and downregulating HK2 weakens the tumor cells proliferation [31]. HK3 is involved in epithelial-mesenchymal transition in colorectal cancer [32]. In recent years, a study demonstrated that HK3 expression was assoiated with immune cell infiltration and could predict the response to immunotherapy in non-small cell lung cancer [33]. Consistent with the majority of tumors, the glycolytic pathway can also affect the occurrence and prognosis of glioma [34]. Benefiting from these positive studies, we assume that HK3 may also have an effect on infiltration level of immune cells in glioma TME.

In our work, we analyzed the co-expression relationship between glycolysis related genes and immune checkpoint genes based on data from TCGA and CGGA and found an association between the expression level of HK3 and glioma immune status. We also systematically analyzed HK3 expression levels in various grades and subtypes of glioma, its latent biological effect, and its prognostic value. The results demonstrated that HK3 could induce higher infiltration level of M2 macrophages, neutrophils, and various subtypes of activated memory CD4^+^ T cells into the TME and could be a predictor of the unfavorable prognosis of glioma in vitro and in vivo.

## Methods

### Patients and data collection

After removing incomplete data, we collected and included data from 577 glioma patients (209 grade 2 patients, 229 grade 3 patients, and 139 grade 4 patients) from TCGA and data from 693 glioma patients (189 grade 2 patients, 255 grade 3 patients, and 249 grade 4 patients) from the CGGA for further study. Transcriptome expression data from TCGA and CGGA were acquired from official websites (https://portal.gdc.cancer.gov/ and http://www.cgga.org.cn/download.jsp). Information about patient clinical traits and tumor molecular features, such as tumor grade, survival outcome, isocitrate dehydrogenase (IDH) mutation status, and O6-Methylguanine-DNA methyltransferase (MGMT) promoter methylation status, were downloaded from TCGA, CGGA and cBioPortal (http://www.cbioportal.org/). We downloaded the glycolysis-related gene sets from the Gene Set Enrichment Analysis (GSEA) database [35]. All the analyses were carried out with TCGA datasets and then validated with CGGA datasets.

### Bioinformatics analysis

The R software (version 4.0.3) and GraphPad Prism 9.0 software were applied to analyze various data and produce figures. The TCGA and CGGA datasets were respectively processed. Pearson’s correlation coefficient (cor) was calculated to determine the correlation between the HK3 expression and other genes using the CORRPLOT R package. Then we calculated stromal scores, immune scores, ESTIMATE scores and tumor purity by the ESTIMATE R package following the method described in a previous study [36]. The correlate levels of infiltrating immune cells were calculated using the CIBERSORT algorithm [37] and single sample GSEA (ssGSEA) algorithm by enrolling signatures of CD4^+^ T cell subtype reported in Zhang and colleagues [38, 39]. Gene Ontology (GO) analysis was performed by using DAVID database [40]. The PHEATMAP R package was used to drawed heatmaps to distinguish different group information. The fractions of significantly conserved bases and the most conserved 200 nt sliding window (phastCons scores averaged within each window from the UCSC Genome Browser) were applied to evaluate the evolutionary conservation of transcripts [41].

### Cell cultures

The GL261 mouse glioma cell line was aquired from the Chinese Academy of Sciences Cell Bank. GL261 cells were clutured in Dulbecco’s modified Eagle’s medium (DMEM; Corning, cat.D6429) supplemented with 10% fetal bovine serum (FBS; BD Biosciences, cat.04-001-1 A) and 1% penicillin–streptomycin (Seven, cat.SC118-01). The cells were grown at 37 °C in 5% CO_2_ and tested negative for mycoplasma using the Mycoplasma Detection Set (ACMEC, cat.AC10863-100T). All cells were maintained for fewer than eight passages.

### Plasmid and transfection

Plasmids were transfected into GL261 cells using Lipofectamine 2000 Reagent (Invitrogen, cat.11,668,019). The Hk3 overexpression plasmid and negative control plasmid were provided by GeneChem (Shanghai) to establish Hk3-overexpression GL261 cells (GL261 Hk3-OE) and Hk3 negative control GL261 cells (GL261 NC), respectively. To overexpress Hk3, GL261 cells were seeded in 6-well plates and transfected with plasmids for 24 h [42]. Subsequently, transfected GL261 cells were screened for 2 weeks using puromycin (Selleck, cat.58-60-6) in a final concentration of 5 µg /ml, and overexpression efficiency was validated by agarose gel electrophoresis, qPCR, Western blotting assay and immunofluorescence assay.

### RNA extraction, PCR, and quantitative real-time PCR assays

Total RNA was extracted by TRIzol reagent (Invitrogen, cat.B1277). Then, we synthesized cDNAs using the PrimeScript RT Reagent Kit (TAKARA, cat. RR037A).The BioRad system was used for routine polymerase chain reaction (PCR), and 1.5% agarose gel electrophoresis was applied to detect the PCR products.

Quantitative real-time PCR was conducted in a reaction mix of SYBR Green (Roche, Switzerland) in triplicate with LightCycler 480 II (Roche, cat. 4,913,914,001), and tested gene expression was normalized to GAPDH expression. PCR primers were designed and synthesized by a primer design tool (http://www.ncbi.nlm.nih.gov/tools/primer-blast/).

Hk3 forward: 5’-CGAGGTGATTTCCTGGCCTT-3’.

Hk3 reverse: 5’-CTGGAAGTCCACGATGCAGT-3’.

Gapdh forward: 5’-CATGTTCGTCATGGGTGTGAA-3’.

Gapdh reverse: 5’-GGCATGGACTGTGGTCATGAG-3’.

### Protein extraction and Western blotting assay

Total proteins of GL261 cells were extracted using prechilled RIPA buffer supplemented with proteinase and phosphatase inhibitor cocktails (Beyotime, cat. P1005). The concentrations of protein were tested with a BCA Protein Assay Kit (Beyotime, cat. P0010). Western blotting assays were performed following standard procedures [43]. The same quantity of proteins (15 µg/sample) were subjected to sodium dodecyl sulfate‒polyacrylamide gel electrophoresis and transferred to PVDF membranes (Roche, cat. 3,010,040,001). The membranes were blocked with 5% BSA in TBS-Tween for 60 min, subsquently incubated with primary antibodies for 8 to 10 h at 4 °C and then incubated with an HRP-labeled secondary antibody (1:4 000; Zsbio Store-bio, cat. ZB-2305/ ZB-2306) at room temperature for 60 min. Specific bands were tested with ECL Western blotting substrate (Boster, cat. EK1001) using a ChemiDoc™ MP Imaging System (Bio-Rad, USA). The primary antibodies were rabbit anti-HK3 (Abcam, cat. ab126217) and mouse anti-GAPDH (Abcam, cat. ab8245), and these antibodies were used at a dilution of 1:1 000.

### Immunofluorescence assay

Cells were seeded on coverslips (WHB-24-CS, China) in 24-well tissue culture plates for 12 h. Subsequently, the cells were incubated in 4% formaldehyde for 15 min at room temperature. After washing 5 min with 1× PBS for three times, the cells were processed with 0.5% Triton X-100 (Thermo Fisher, cat. HFH10) and blocked with blocking buffer (5% bovine serum albumin diluted in PBS, BioFroxx, cat. 9048-46-8) for 1 h at room temperature. The diluted HK3 antibodies were added and incubated at 4 °C for 8 to 10 h. The cells were incubated with FITC-labeled anti-IgG antibodies (Alexa Fluor 488 and 594, Thermo Fisher, cat. A-110,081, cat. A-11,011,

cat. A-110,051, cat. A-110,121) for 60 min at room temperature after rinsing three times in PBS. DAPI (Sigma, cat. D9542) was applied to stain the DNA [44]. The images of the subcellular localization of proteins were detected using a fluorescence microscope.

### Mouse intracranial model

All the following experimental protocols were authorized by the Committee on the Ethics of Animal Experiments of Harbin Medical University (ID: SYDW2019-62). C57BL/6 N mice were purchased at the age of three to four weeks (Charles River, China). To construct the intracranial tumor model, 1 × 10^6^ GBM cells (GL261 Hk3-OE or NC) per mouse were separately implanted stereotactically with cranial guide screws. Each group included 10 mice. The growth degree of mice intracranial tumors were assessed by bioluminescence imaging on days seven, fourteen, and twenty-one [45]. In addition, overall survival time of each mouse was recorded. After implantation for 25 days, randomly selected mice from each group were killed via CO_2_ asphyxiation (10–20%/min). The brain tissues were discreetly removed and fixed in 10% formalin. Then, they were wrapped in paraffin and sectioned (4 μm) for hematoxylin and eosin (H&E) staining and immunohistochemistry (IHC). At last, all the mice were dead, and the overall survival curves were generated on the basis of the Kaplan–Meier method.

### H&E staining and immunohistochemistry assays

For H&E staining, the slides were dyed in hematoxylin solution (ZSGB-BIO, cat.ZLI-9610) for 8 min and in eosin–phloxine solution (ZSGB-BIO, cat.ZLI-9613) for 0.5 to 1 min after deparaffinization and rehydration. Subsequently, the slides were mounted uing xylene.

IHC was performed to ascertain the expression of CD206, CD4, LY6G, TBX21, IFNG, GATA3, IL4, IL17, RORC, IL2RA, and FOXP3 in tumor tissues. In brief, paraffin sections were incubated at 80 °C for 15 min, dewaxed in xylene, washed in gradient ethanol solutions, and rehydrated in double-distilled water. Then, the slides were pretreated by steaming them in sodium citrate buffer for 15 min at 95 °C for antigen retrieval [46]. After rinsing in PBS for 3 min, the slides were immunostained with primary antibodies against CD206 (1:100; Abclonal, cat.A8301), CD4 (1:100; Affinity, cat. DF6451), LY6G (1:100; Cell Signaling Technology, cat.87,048 S), TBX21 (1:100; Abcam, cat.ab307193), IFNG (1:100; Abclonal, cat. A12450), GATA3 (1:100; Abclonal, cat.A19636), IL4 (1:100; Abcam, cat.ab300138), IL17 (1:100; Abclonal, cat.A21266), RORC (1:100; Abcam, cat.ab207082), IL2RA (1:100; Cell Signaling Technology, cat. 39,475), and FOXP3 (1:100; Cell Signaling Technology, cat.72,338) at 4 °C overnight. After being rinsed with PBS buffer for 10 min 3 times, the slides were covered with an HRP-labelled anti-mouse/rabbit polymer for 0.5 h. The dyeing reactions were carried out by incubating the samples with the prepared DAB substrate kit (ZSGB-bio, cat. ZIL-9017) [47]. The tissues were incubated for 1 min to allow for the suitable development of a brown color.

### Statistical analysis

Student’s t test was applied to analyze differences between two groups. Kaplan‒Meier survival analyses and the log-rank test were used to assess the value of HK3 expression to survival. Univariate and multivariate Cox regression analyses were applied to indicate the prognostic significance of HK3. The area under the curve (AUC) and the receiver operating characteristic (ROC) curve were drawn by R software. Time-dependent ROC curve analyses were applied to evaluate one-, three- and five-year overall survival (OS) prediction. At last, all the statistical data was processed using SPSS 22.0 software and R software. A p value < 0.05 was reckoned to be statistically significant. Every statistical method was double-sided.

## Results

### The co-expression relationship between glycolytic pathway genes and immune checkpoint genes

**Fig. 1:**
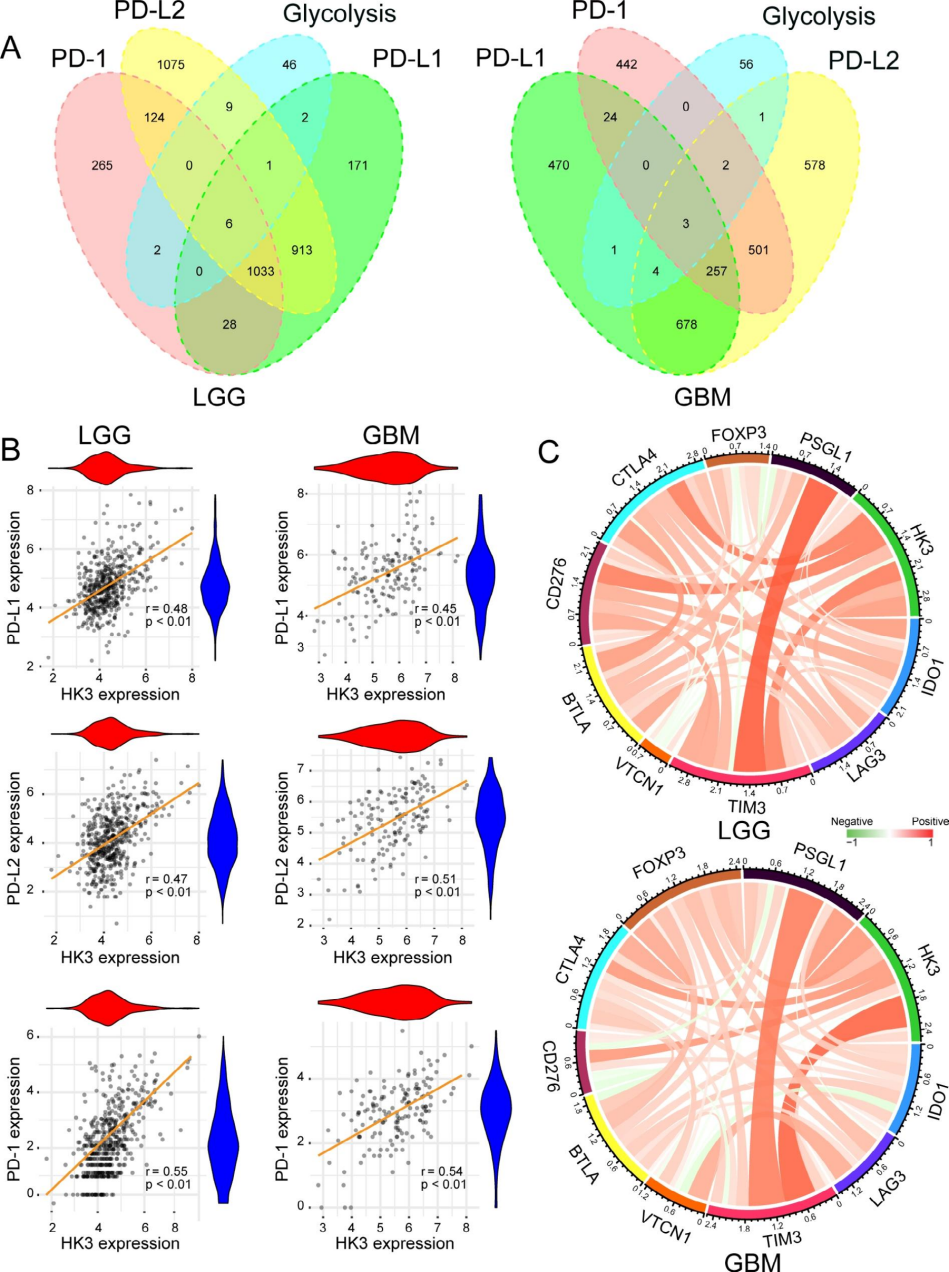
The correlation between immune checkpoint expression and HK3 expression in LGG and GBM. **(A)** Venn diagram of the number of genes whose expression was associated with PD-L1, PD-L2, PD-1, and glycolytic pathway-related gene expression in LGG and GBM from the TCGA database. **(B)** Pearson’s correlation coefficients between HK3 and PD-L1, PD-L2, and PD-1 expression in LGG and GBM from the TCGA database. **(C)** The simultaneous cooperation and operation between HK3 and other immune checkpoints in LGG and GBM.

Several studies have revealed that the glycolysis is associated with tumor immune evasion [48]. However, the specific mechanism in glioma remains unclear. By examining the TCGA database, we screened for genes whose expression was positively correlated with that of three immune checkpoints, namely, PD-1, PD-L1 and PD-L2 (cor > 0.3). We overlapped the three sets of genes and acquired genes whose expression were positively correlated with that of these three immune checkpoints. Subsequently, we crossed these genes with the glycolysis related genes from the GSEA database and found interesting genes in the glycolytic pathway whose expression was positively correlated with that of the three immune checkpoints. We performed the above procedures in lower-grade glioma (LGG) and GBM datasets. As presented in Fig. 1A, we identified 6 genes (PCK2, LDHA, GALM, HK2, HK3, and PGM2) from the LGG datasets and 3 genes (ALDH3B1, ADPGK, and HK3) from the GBM datasets. Finally, we summarized the overlapping genes between LGG and GBM and discovered that the first rate-limiting enzyme in the glycolytic pathway, HK3, exhibited same trend in both LGG and GBM. Pearson’s correlation coefficient analysis between HK3 expression and the expression of these three immune checkpoints was shown in Fig. 1B. To deeply explore the relationship between HK3 expression level and immune checkpoint expression level, other immune checkpoints related genes, such as TIM-3, PSGL-1, CTLA-4, and CD276 according to the study by Liu et al. [33], were also studied here. As presented in Fig. 1C, HK3 expression was positively correlated with the expression of multiple immune checkpoint genes in both LGG and GBM. Taken together, these data showed that Hk3 expression might be closely associated with the reprogramming of the immunosuppressive microenvironment in glioma.

### HK3 expression correlates with immune infiltration levels in glioma

**Fig. 2:**
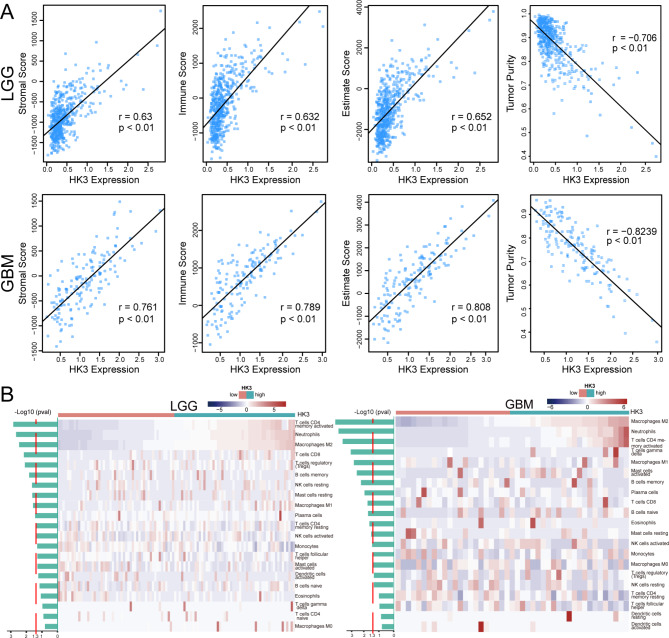
The correlation between HK3 expression and immune cell infiltration levels in LGG and GBM from TCGA datasets. **(A)** Pearson’s correlation coefficients between HK3 mRNA expression and immune scores in the LGG and GBM datasets. **(B)** Immune cell infiltration in LGG and GBM samples with low and high HK3 expression levels

Previous studies have shown that the immune cell infiltration into tumor tissues is an independent prognostic factor of different cancers [49]. Consequently, we investigated the association between HK3 expression and immune cell infiltration in glioma. The stromal score, immune score, ESTIMATE score and tumor purity of each patient in TCGA and CGGA datasets were calculated using the ESTIMATE algorithm. As presented in Fig. 2A and Figure S1A, the stromal score, immune score, and ESTIMATE score were positively correlated with HK3 expression in both LGG and GBM. Tumor purity was negatively correlated with HK3 expression. These results illustrated that the extent of immune cell infiltration in tumor tissues increased with high HK3 expression. Next, we focused on the association between various immune cell types and HK3 expression. By utilizing the CIBERSORT algorithm, the relationship between HK3 expression and the different types of immune cells were explored in TCGA and validated in the CGGA datasets. Samples with high HK3 expression showed high numbers of immune cells, including M2 macrophages, neutrophils, and activated memory CD4^+^ T cells, in both LGG and GBM, as shown in Fig. 2B and Figure S1B. These results might partially explain the poor outcomes of glioma patients with high HK3 expression.

### HK3 expression is associated with immune-related functions

**Fig. 3:**
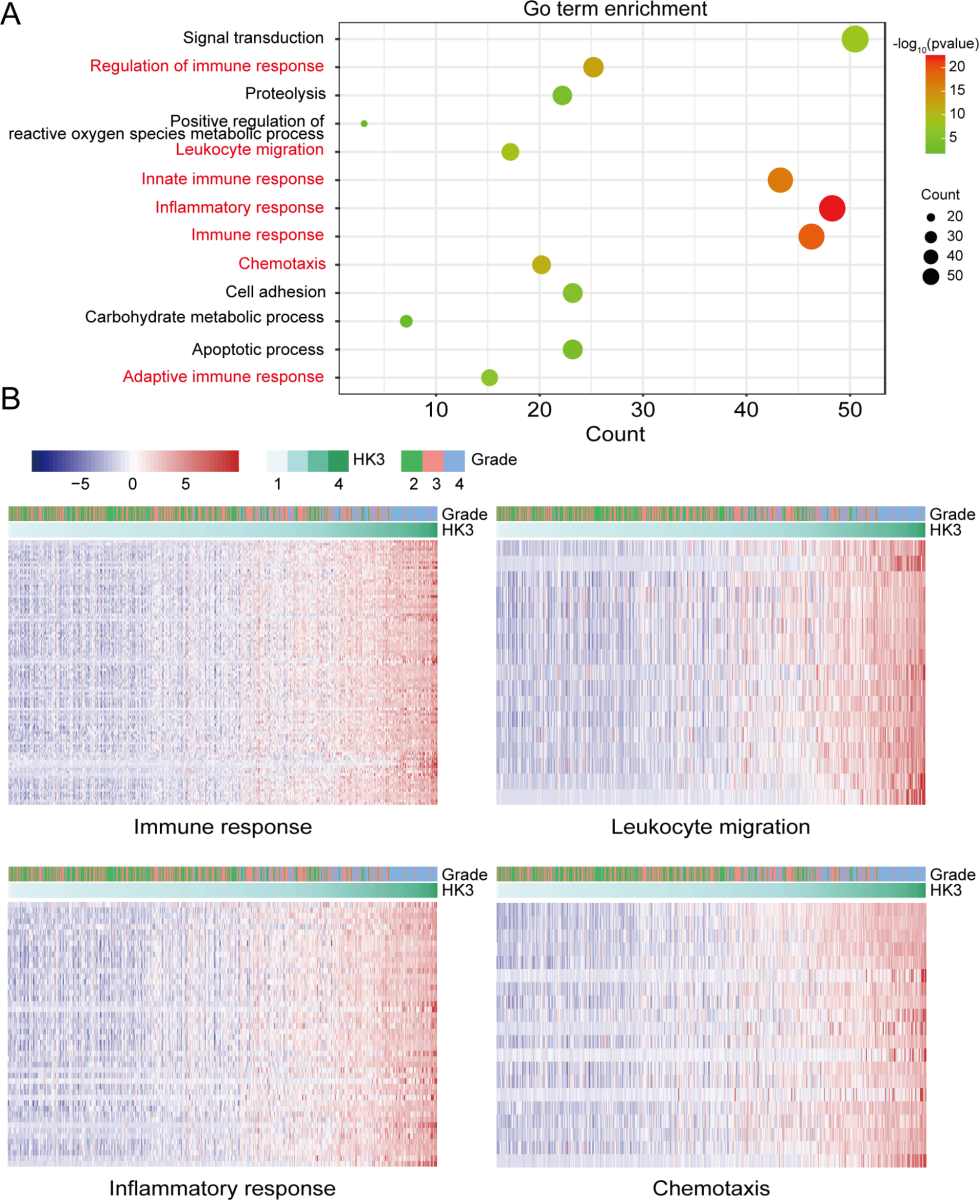
Functional analysis of HK3-related genes in the TCGA datasets. **(A)** GO enriched pathways of genes whose expression was positively correlated with that of HK3. Immunity-related terms are marked in red. **(B)** Heatmap of genes positively correlated with HK3 in the immune response, leukocyte migration, inflammatory response, and chemotaxis

To explore the biological role of HK3 in glioma, Pearson’s correlation coefficient analysis was used to assess genes whose expression was apparently correlated with HK3 expression (|cor| > 0.6, p < 0.05) in 577 glioma patient samples from TCGA. The whole number of positively related genes in the TCGA datasets was 322. The biological functions of these related genes were studied through the DAVID database. The results of the Gene Ontology (GO) analysis showed that these genes whose expression was positively correlated with that of HK3 were involved mainly in the inflammatory response, immune response, chemotaxis and leukocyte migration (Fig. 3A). Heatmaps of genes whose expression was positively correlated with that of HK3 in various immune functions were shown in Fig. 3B and Table S3-6. There were too few genes with negative correlations, and the data were not displayed. In addition, we also performed an RNA-seq analysis between GL261 Hk3-KD and NC groups to investigate the related pathway affected by HK3 expression. Firstly, we performed the different expression genes analysis using the RNA-seq data. All the different expression genes and immune related cytokines affected by Hk3 expression alteration were illustrated by the Figure S2A-B. Then, we used the KEGG enrichment analysis to explore the signaling pathway affected by Hk3. As shown in Figure S2C, Hk3 could regulated several intracellular transduction signaling pathways such as TNF signaling pathway, MAPK signaling pathway, NF-kappaB signaling pathway and so on. Hence, all these results illustrated that HK3 was involved in TNF signaling pathway and regulated several cytokines in glioma cells, indicating that HK3 reprogramed the tumor microenvironment through regulating intracellular transduction signaling pathway in glioma cells.”

### HK3 expression is upregulated in glioblastoma, mesenchymal subtypes and glioma with wild-type IDH

**Fig. 4:**
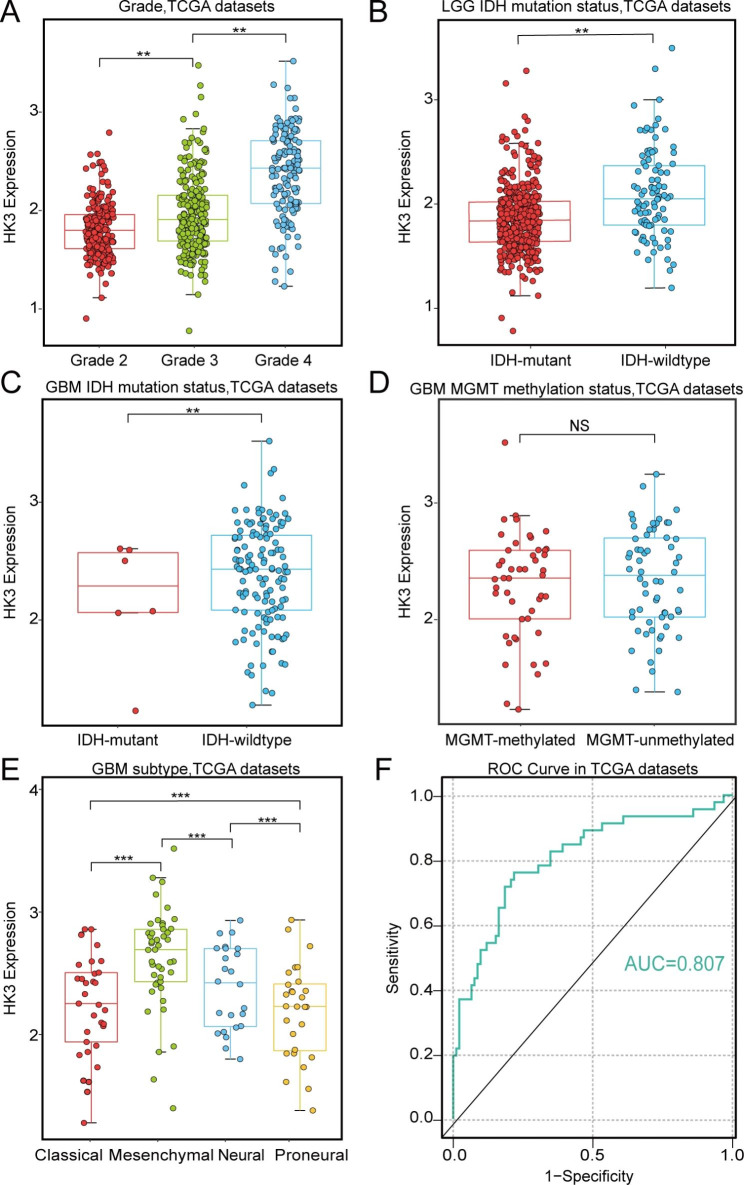
HK3 expression in stratified glioma. **(A)** HK3 expression in tumors of different grades in the TCGA datasets. **(B)** HK3 expression in LGG stratified according to IDH mutation status in TCGA datasets. **C and D**. HK3 expression in GBM according to IDH mutation status and MGMT methylation status in TCGA datasets. **E**. HK3 expression in different molecular subtypes in the TCGA datasets. **F**. ROC curve analysis of the efficiency of HK3 expression in predicting the mesenchymal subtype in the TCGA datasets. The p value was determined by Student’s t test. The data are presented as the mean ± S.D. Significant results are presented as ns: nonsignificant, **p < 0.01, or ***p < 0.001

We analyzed the HK3 expression level in samples from TCGA stratified according to tumor grade, IDH mutation status and MGMT promoter methylation status. As presented in Fig. 4A, The expression level of HK3 was significantly elevated along with the tumor grade and was the highest in glioblastoma (GBM, grade 4). In the LGG and GBM patients who were stratified according to IDH mutation status, the HK3 expression level was higher in the IDH wild-type group, which had a potentially worse prognosis than the IDH mutant-type group, as shown in Fig. 4B-C. In the GBM patients who were stratified according to MGMT promoter methylation status, there was no significant association with HK3 expression, as shown in Fig. 4D. Moreover, we also validated these results using the CGGA datasets, and the results were shown in Figure S3.

Next, we investigated the expression of HK3 in four molecular subtypes of tumors in TCGA. Based on the TCGA datasets, the mesenchymal subtype, which was recognized as a more aggressive subtype of glioma, showed the higher expression of HK3 compared with other GBM subtypes (Fig. 4E). The glioma was subsequently divided into mesenchymal and non-mesenchymal subtypes for ROC curve analysis, and the results demonstrated that HK3 expression was a good predictor of mesenchymal subtype (Fig. 4F). This was further proven by the area under the ROC curve (AUC), which was 0.807 for the TCGA datasets. All these results indicated that a high level of HK3 expression was involved in the malignant progression of glioma.

### High HK3 expression correlates with poor outcomes and is an independent prognostic predictor in glioma

**Fig. 5:**
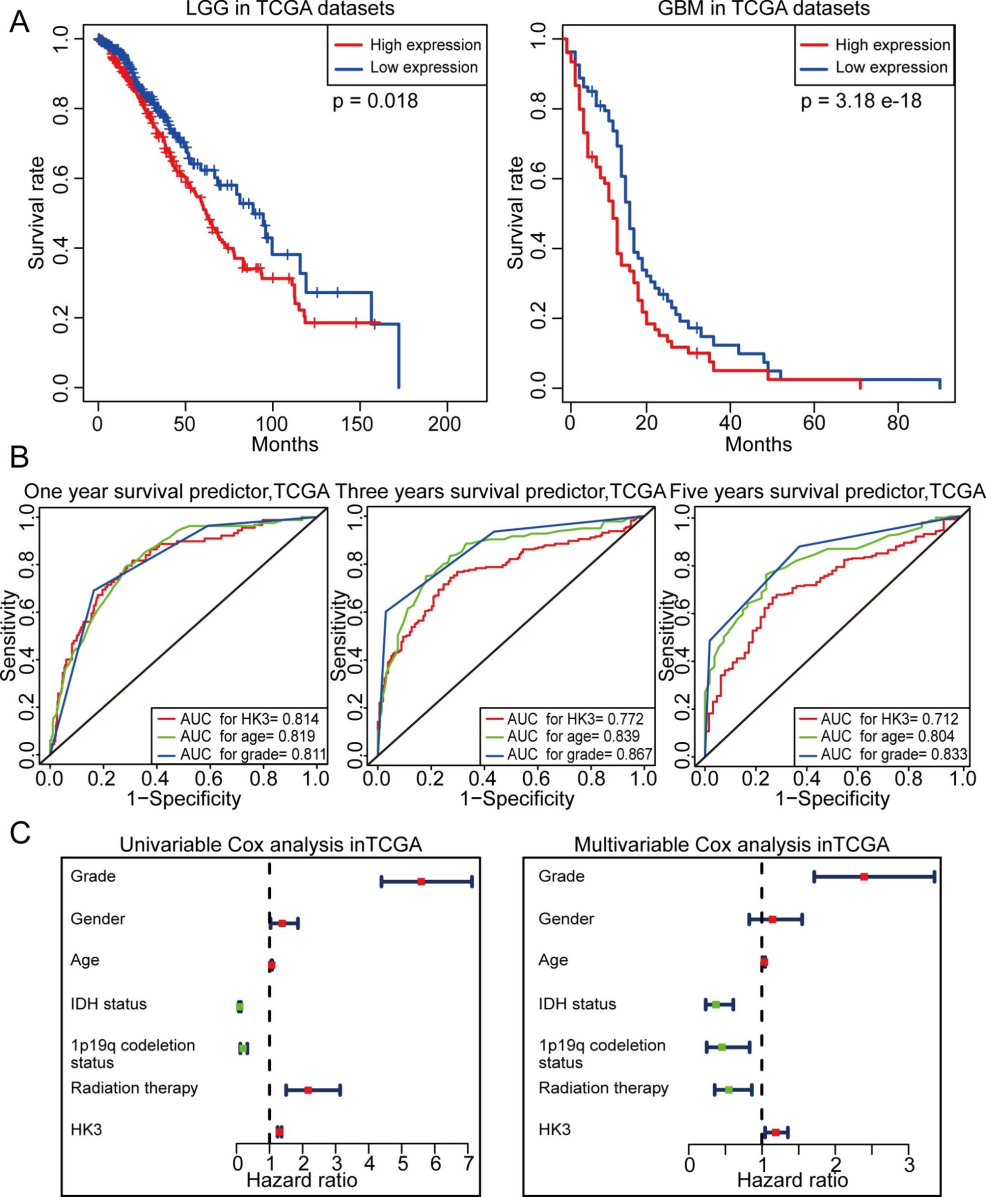
Survival analysis of HK3 in glioma. **(A)** Kaplan‒Meier survival analysis of LGG and GBM in TCGA datasets. In GBM group, the high HK3 expression patients is 77 and low HK3 expression patients is 76. In LGG group, the high HK3 expression patients is 253 and low HK3 expression patients is 252. **(B)** Time-dependent ROC curve analysis of the efficiency of HK3 expression, patient age at diagnosis, and tumor grade in predicting one-year, three-year, and five-year OS in the TCGA datasets. **(C)** Univariable and multivariable Cox regression analyses of HK3 expression and several other clinical factors in the TCGA datasets

We investigated the prognostic value of HK3 expression in glioma. Kaplan‒Meier survival analyses were performed respectivly on the LGG (grade 2, 3) and GBM patients (grade 4) in TCGA and CGGA datasets, and the median HK3 expression level was used as a cutoff point (1.824 for GBM and 0.387 for LGG in TCGA, 1.679 for GBM and 0.846 for LGG in CGGA). In both patients with LGG and those with GBM, higher HK3 expression predicted shorter overall survival (OS). As shown in Fig. 5A and Figure S4A, consistent results were obtained with these two datasets. Subsequently, we also compared the specificity and sensitivity of HK3 expression, patient age at diagnosis, and tumor grade in predicting OS. The AUCs based on HK3 expression for one-, three-, and five-year OS were 0.814, 0.772, and 0.712, respectively, in TCGA datasets and were similar to the corresponding AUCs based on age and tumor grade (Fig. 5B). In the datasets from CGGA, HK3 expression was still a better predictor of OS, which was consistent the results of tumor grade and age (Figure S4B). Next, we performed univariable and multivariable Cox analyses to determine that HK3 expression was an independent prognostic predictor for glioma using the TCGA datasets shown in Fig. 5C and Table S1. Similar results after univariable and multivariable Cox regression analysis with the CGGA datasets were also shown in Figure S4C and Table S2. Overall, these results indicated that high HK3 expression was associated with poor prognosis in patients with glioma and that the gene expression level could independently predict OS.

### Overexpression of HK3 stimulates glioblastoma growth and alters immune cell infiltration in vivo

**Fig. 6:**
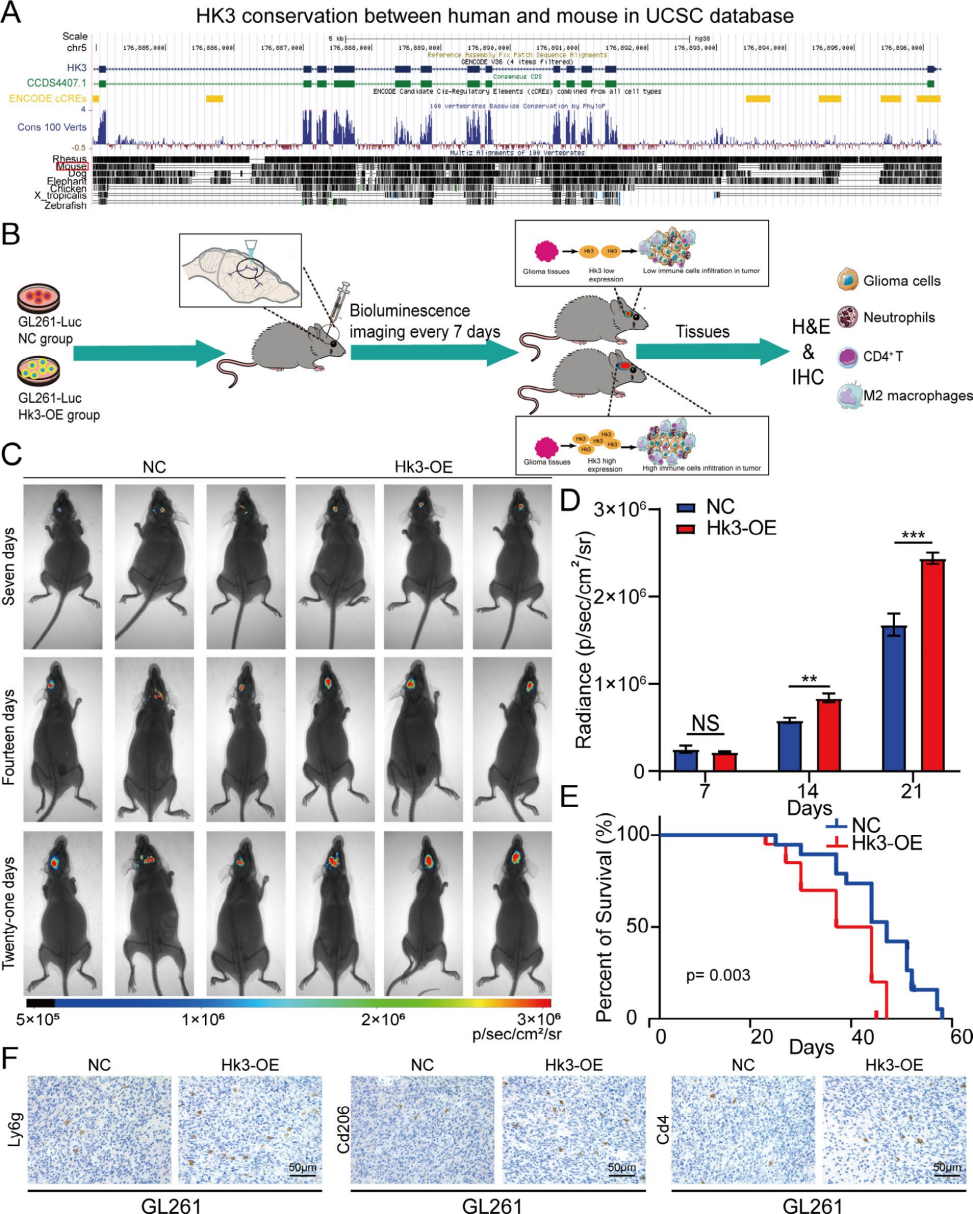
Overexpression of Hk3 in GL261 cells promotes orthotopic tumor growth in vivo **(A)** Analysis of HK3 gene conservation between different species according to the UCSC database. **(B)** C57BL/6 N mice were orthotopically xenografted with GL261-Luc cells. **(C)** Bioluminescence images of C57BL/6 N mice. **(D)** Quantification of the signal intensities revealed by bioluminescence imaging of C57BL/6 N mice. **(E)** Kaplan‒Meier survival curve of C57BL/6 N mice is shown N = 10. **(F)** IHC staining for Ly6g, Cd206 and Cd4 in brain sections from mice tumors derived from GL261-Luc cells. Scale bar = 50 μm. The data are presented as the mean ± S.D. Significant results are presented as ns: nonsignificant, **p < 0.01, or ***p < 0.001

Phylogenetic analysis using the UCSC Genome Browser (http://genome.ucsc.edu/) revealed that the HK3 gene was highly conserved among mammals and vertebrates, as shown in Fig. 6A, suggesting an important function for HK3. As a result, we established orthotopic mouse models using GL261 cells to further verify the role of Hk3 in vivo. We used plasmid systems to overexpress Hk3 in GL261 cells. The Hk3 overexpression plasmid and negative control plasmid were transfected into GL261 cells to construct the GL261 Hk3-OE and GL261 NC cells. The expression level of Hk3 were determined by agarose gel electrophoresis, qPCR, Western blotting assay, and immunofluorescence assay after plasmid transfection. The results demonstrated a substantial elevation of Hk3 expression in GL261 cells, which was shown in Figure S5A-D (the original western blots was provided in Figure S6). Subsequently, Hk3-OE and NC GL261 cells were injected into the brains of C57BL/6 N mice through cranial guide screws, as shown in Fig. 6B. In these models, the Hk3-OE group showed a significant increase in intracranial tumor volume compared to the NC group (Fig. 6C-D). In addition, overexpression of Hk3 in GL261 tumors was associated with shortened survival time, as shown in Fig. 6E. At endpoint of the in vivo experiment, we used the western blotting assay and immunofluorescence assay to prove the HK3 overexpression in mouse brain tissues shown in Figure S7. In addition, we also established orthotopic mouse models using GL261 Hk3-KD and GL261 NC cells to further determine that HK3 knocking down could improve OS. In these models, the Hk3-KD groups showed a significant decrease in intracranial tumor volume compared to the NC groups (Figure S8A-B). In addition, knocking down of Hk3 in GL261 tumors was associated with longer survival time, as shown in Figure S10C. Finally, as shown in Figure S9, H&E staining showed that the mouse brain tissues extracted from the Hk3-OE group and HK3 NC group contained tumor tissues. Furthermore, to confirm the associations between the expression of Hk3 and immune cell infiltration, IHC for several biomarkers of immune cells, such as CD206 for M2 macrophages [50], CD4 for CD4^+^ T cells [51], and LY6G for neutrophils [52], was carried out on samples from the Hk3-OE group and NC group. As shown in Fig. 6F, the IHC results showed that Hk3-overexpressing samples also had high expression levels of CD206, CD4, and LY6G in their GL261 cell-derived tumors, which indicated the increased infiltration of M2 macrophages, CD4^+^ T cells, and neutrophils. Due to activated memory CD4^+^ T-cells could differentiate into several distinct T cell including T helper (Th) 1 and Th2 cells but also Th17 cells and regulatory T cells (Treg). So, we further analyzed the relationship between HK3 expression and various subtypes of CD4^+^ T cells in both LGG and GBM. HK3 expression was positively correlated with the infiltration level and related markers of Th2, Th17 and Treg cell and negatively correlated with the infiltration level and related markers of Th1 cell in both GBM and LGG (Figure S10A, B). To confirm the associations between the expression of HK3 and markers, IHC for several markers of immune cells, such as TXB21 [53], IFNG for Th1 cell [54], GATA3, IL4 for Th2 cells [55, 56], IL17, RORC for Th17 cell [57, 58] and IL2RA, FoxP3 for Treg cell [58, 59], was carried out on samples from the Hk3-OE group and NC group, and showed that Hk3-overexpressing group also had higher expression levels of GATA3, IL4, RORC, IL17 and IL2RA, FoxP3 and less expression of TBX21, IFNG in their GL261 cell-derived tumors, which indicated more Th2, Th17 and Treg cells infiltration and less Th1 cells infiltration into the tumor microenvironment, compared with those in NC group (Figure S10C). The above results demonstrated that HK3 expression could affect the activated memory CD4^+^ T cells infiltration and induced more Th2, Th17 and Treg cells infiltration and less Th1 cells infiltration into the tumor microenvironment. These results were consistent with our bioinformatic analysis results and partially explained the poor outcome of glioma patients with a high level of Hk3 expression.

## Discussion

Glioma, which is the most common primary brain tumors of the CNS, consists of a heterogeneous population including tumor cells, immune cells, and extracellular matrix components [60]. The interactions among these cells can accelerate tumor development, progression and immune evasion [61, 62]. Currently, the standard treatments for glioma are still surgery, temozolomide (TMZ) chemotherapy and radiation [63, 64]. However, the prognosis of glioma patients remains poor because of the development of resistance to TMZ chemotherapy [65]. In recent years, the development of immunotherapy for cancer treatment has resulted in hope for glioma patients [66, 67]. However, the distinctively immune-privileged microenvironment that is formed due to the intrinsic expression of immunosuppressive cytokines, such as PD-1, PD-L1, TGF-β and IL10, and the lack of antigen-presenting cells (APCs) in the CNS result in challenges for glioma patient prognosis. Therefore, it is crucial to study the immunosuppressive tumor microenvironment of glioma and to identify new biomarkers to predict glioma prognosis and develop new therapies.

Previous studies have revealed that immune dysfunction and metabolic reprogramming are two characteristics of tumors [24, 25]. In our study, by examining the co-expression relationship between immune checkpoint genes and glycolytic pathway-related genes through the TCGA and CGGA datasets, we revealed that HK3 expression is positively correlated with the expression levels of multiple immune checkpoint genes in LGG and GBM. Hexokinases (HKs), the first rate-limiting enzymes in cell glycolysis, are considered the key molecules that regulate cell energy metabolism and cell fate in various tumor cells. Some previous studies have revealed that HK3 can induce epithelial-mesenchymal transition in colorectal cancer [32]. Moreover, HK3 expression is correlated with immune cell infiltration in non-small cell lung cancer [33] and clear cell renal cell carcinoma [12]. Therefore, although HK3 was a glycolysis enzyme, we hypothesized that HK3 might not play a major role in glycolysis in glioma. In this study, through GO analysis of the biological role of HK3, we found that HK3 played a pivotal role in inflammatory activities and immunological reactions in glioma. In addition, the ESTIMATE algorithm, CIBERSORT algorithm and ssGSEA algorithm illustrated that high expression of HK3 could stimulate the infiltration of M2 macrophages, neutrophils, and various subtypes of activated memory CD4^+^ T cells, which was consistent with our IHC results. The high infiltration level of immune cells in glioma prompts tumor cells to defensively upregulate various immune checkpoints to escape the immune system [33]. Therefore, high expression of HK3 had a positive correlation with the infiltration of immune cells and predicts shorter overall survival (OS) in glioma patients.

The tumor microenvironment adapts to pathologies characterized by chronic inflammation and metabolic alterations through a complex network of various cells [68]. In glioma, M2 macrophages can promote the stemness and migration abilities of glioma cells by secreting TGF-β1 via the SMAD2/3 signaling pathway [69]. Our previous study demonstrated that neutrophil extracellular traps (NETs), which were produced by tumor-infiltrating neutrophils (TINs), were oncogenic markers of higher-grade glioma and were involved in cell proliferation and invasion [70]. Activated memory CD4^+^ T cells have been shown to differentiate into distinct subtypes of T cells including Th1, Th2, Th17 and Treg cells and produce many cytokines that regulate the pro or anti-tumor role of CD4^+^ T cells during malignant biological process of glioma cells [71]. Some studies have proven that there is a Th1/Th2 imbalance in glioblastoma patients [72, 73]. The Th2 cell and their cytokines are strongly expressed in almost all glioblastomas and stimulate tumor deterioration [73, 74]. In contrast, the Th1 cell and their cytokines are essentially spare in glioblastoma. Th17 cells are associated with a poor clinical outcome and there is a strong link between angiogenesis and Th17 cytokines expressions in glioblastoma [75]. Treg cells depress immune function and produce an immunosuppressive microenvironment in glioblastoma [76, 77]. Yue et al. found a significant association between the density of Tregs infiltration and poor prognosis in glioblastoma patients [78]. In this study, the results of the in vivo experiments demonstrated that the overexpression of Hk3 could promote the malignant progression of glioma in mice along with increased infiltration level of M2 macrophages, neutrophils, and various subtypes of activated memory CD4^+^ T cells.

In summary, our current work demonstrated that HK3 could increase the infiltration of M2 macrophages, neutrophils, and various subtypes of activated memory CD4^+^ T cells into the tumor microenvironment and could be a predictor of the unfavorable prognosis of glioma. These findings could offer novel insights into how HK3 regulated the activation of immune cells in the tumor microenvironment to mediate immune evasion, and these findings could provide a new theoretical basis for understanding the metabolic network within the glioma microenvironment and identifying new therapeutic targets. The advantage of our research was that we first assessed the prognostic role of the differential expression levels of HK3 in glioma. Second, this study paid more attention to the diversity and complexity of the immune cells that infiltrated the tumor microenvironment of glioma. Third, we performed not only bioinformatics methods but also biological experiments in our study to provide rigorous and objective results.

## Conclusion

Our present study demonstrates that HK3 can regulate glioma microenvironment by elevating the infiltration level of M2 macrophage, neutrophil, and various subtypes of activated memory CD4^+^ T. In addition, HK3 expression is significantly elevated along with the tumor grade and is the highest in GBM samples compared with Grade 2 and 3 samples and can predict unfavorable prognosis of glioma patients in vitro and in vivo.

### Electronic supplementary material

Below is the link to the electronic supplementary material.

Supplementary Table S1. Univariable and multivariable Cox regression analyses of HK3 expression and several other clinical factors in the TCGA datasets.

Supplementary Table S2. Univariable and multivariable Cox regression analyses of HK3 expression and clinicopathologic factors in the CGGA dataset.

Supplementary Table S3. Genes positively correlated with HK3 in immune response.

Supplementary Table S4. Genes positively correlated with HK3 in leukocyte migration.

Supplementary Table S5. Genes positively correlated with HK3 in inflammatory response.

Supplementary Table S6. Genes positively correlated with HK3 in chemotaxis.

Supplementary Figures: Supplementary Figure S1. The correlation between HK3 expression and immune cells infiltration levels in LGG and GBM from CGGA datasets. A. Pearson’s correlation coefficients between HK3 mRNA expression and immune scores in LGG and GBM datasets. B. Immune cells infiltration in LGG and GBM samples with low and high HK3 expression levels. Supplementary Figure S2. RNA-seq analysis between GL261 Hk3-OE and NC groups. A. Volcano map of differential expression genes. B. Heatmap of differential immune related cytokines. C. KEGG enrichment analysis of intracellular transduction signaling pathway. Supplementary Figure S3. HK3 expression in stratified glioma. A. HK3 expression in different tumor grades in the CGGA datasets. B. HK3 expression in LGG stratified according to IDH mutation status in CGGA datasets. C and D. HK3 expression in GBM according to IDH mutation status and MGMT methylation status in CGGA datasets. Supplementary Figure S4. Survival analysis of HK3 in glioma. A. Kaplan-Meier survival analysis of LGG and GBM in CGGA datasets. In GBM group, the high HK3 expression patients is 119 and low HK3 expression patients is 118. In LGG group, the high HK3 expression patients is 210 and low HK3 expression patients is 210. B. Timedependent ROC curve analysis of the efficiency of HK3 expression, patient age at diagnosis, and tumor grade in predicting 1-year, 3-years, 5-years OS in the CGGA datasets. C. Univariable and multivariable Cox regression analyses of HK3 expression and several other clinical factors in the CGGA datasets. Supplementary Figure S5. Validation of Hk3-overexpression (Hk3-OE) in in GL261 cells. A and B. PCR and qPCR were used to examine whether the Hk3 expression level was high in the GL261 Hk3-OE groups. All the experiments were repeated three times. C. Western blotting assays revealed that the Hk3 expression level was high in the GL261 Hk3-OE groups. All the experiments were repeated three times. D. The results of the immunofluorescence assay illustrate that the Hk3 expression level is high in the GL261 Hk3-OE groups. Scale bar = 20 μm. The data are presented as the mean ± S.D. Significant results are presented as **p<0.01. Supplementary Figure S6. The original blots for figure S7. A. The original blots of Hk3. B. The original blots of Gapdh. Supplementary Figure S7. Validation of Hk3-overexpression (Hk3-OE) in brain sections from mice tumors derived from GL261-Luc cells. A. Western blotting assays revealed that the Hk3 expression level was high in the GL261 Hk3-OE groups. B. IHC staining for Hk3 in brain sections from mice tumors derived from GL261-Luc cells. Supplementary Figure S8. Knock-down of Hk3 in GL261 cells suppress orthotopic tumor growth in vivo. A. Bioluminescence images of C57BL/6N mice. B. Quantification of the signal intensities revealed by bioluminescence imaging of C57BL/6N mice. C. Kaplan‒Meier survival curve of C57BL/6N mice is shown N=10. Supplementary Figure S9. H&E staining for Hk3 NC group and Hk3-OE group. Scale bar = 50 μm. Supplementary Figure S10. Correlation between HK3 expression and markers of CD4+ T cell in glioma. A. Correlation between HK3 expression and subtypes of CD4+ T cell in LGG and GBM samples in TCGA datasets. B. Correlation between HK3 expression and markers of CD4+ T cell in LGG and GBM samples in TCGA datasets. C. IHC staining for markers of CD4+ T cell in brain sections from mice tumors derived from GL261-Luc cells. Scale bar = 50 μm.

## Data Availability

All the data obtained and/or analyzed in this study were available from the TCGA and CGGA databases.

## List of abbreviations

TME: Tumor microenvironment
TCGA: The Cancer Genome Atlas
CGGA: Chinese Glioma Genome Atlas
CNS: Central nervous system
IDH: Isocitrate dehydrogenase
MGMT: O6-Methylguanine-DNA methyltransferase
GSEA: Gene Set Enrichment Analysis
GO: Gene Ontology
HK3-OE: HK3 overexpression
NC: Negative control
PCR: Polymerase chain reaction
qRT‒PCR: Quantitative real-time PCR
IHC: Immunohistochemistry
AUC: Area under the curve
ROC: Receiver operating characteristic
OS: Overall survival
