# Supplementary material for: HK3 stimulates immune cell infiltration to promote glioma deterioration
Source: Cancer Cell Int. 2023 Oct 1;23:227. doi: 10.1186/s12935-023-03039-w (PMC10543879; doi:10.1186/s12935-023-03039-w)
Supplement: Supplementary file 1 — Supplementary Table S1. Univariable and multivariable Cox regression analyses of HK3 expression and several other clinical factors in the TCGA datasets. [file 12935_2023_3039_MOESM1_ESM.pdf]

Table S1. Univariable and multivariable Cox regression analyses of HK3 expression and clinicopathologic factors in the TCGA dataset.

Factors	Patient numbers	Univariate analysis HR (95% CI)	p value	Multivariate analysis HR (95% CI)	p value
Grade		5.592(4.387—7.128)	6.19E-44	2.394(1.712—3.354)	3.13E-07
2	180				
3	203				
4	124				
Gender		1.384(1.030—1.860)	0.031	1.143(0.825—1.548）	0.418
Male	293				
Female	214				
Age	507	1.065(1.054—1.077)	2.97E-30	1.032(1.006—1.043)	0.054
IDH status		0.097(0.070—0.134)	6.41E-45	0.372(0.231—0.608)	7.74E-05
Wildtype	187				
Mutant	320				
1p19q codeletion status		0.195(0.116—0.326)	4.55E-10	0.454(0.244—0.831)	0.01
Non-codel	379				
Codel	128				
Radiation therapy		2.170(1.502—3.136)	3.67E-05	0.547(0.353—0.861)	0.008
Yes	345				
No	162				
HK3	507	1.303(1.243—1.366)	5.18E-28	1.188(1.042—1.353)	0.015
