# Supplementary material for: HK3 stimulates immune cell infiltration to promote glioma deterioration
Source: Cancer Cell Int. 2023 Oct 1;23:227. doi: 10.1186/s12935-023-03039-w (PMC10543879; doi:10.1186/s12935-023-03039-w)
Supplement: Supplementary file 2 — Supplementary Table S2. Univariable and multivariable Cox regression analyses of HK3 expression and clinicopathologic factors in the CGGA dataset. [file 12935_2023_3039_MOESM2_ESM.pdf]

Table S2 Univariable and multivariable Cox regression analyses of HK3 expression and clinicopathologic factors in the CGGA dataset.

Factors	Patient numbers	Univariate analysis HR (95% CI)	p value	Multivariate analysis HR (95% CI)	p value
Grade		2.835(2.404—3.343)	3.40E-35	2.296(1.897—2.778)	1.31E-17
II	143				
III	201				
IV	190				
Gender		1.103(0.882—1.381)	0.390	1.034(0.823—1.294)	0.774
Male	303				
Female	231				
Age	534	1.026(1.016—1.036)	1.02E-07	1.009(0.992—1.017)	0.054
IDH status		0.322(0.257—0.404)	9.79E-23	0.686(0.521—0.902)	0.007
Wildtype	229				
Mutant	305				
1p19q codeletion status		0.285(0.202—0.402)	9.06E-13	0.414(0.284—0.601)	3.84E-06
Non-codel	413				
Codel	121				
Radiation therapy		1.108(0.839—1.463)	<0.470	0.687(0.513—0.920)	0.012
Yes	422				
No	112				
HK3	534	1.466(1.321—1.627)	5.91E-13	1.379(1.267—1.507)	0.028
