# Supplementary material for: HK3 stimulates immune cell infiltration to promote glioma deterioration
Source: Cancer Cell Int. 2023 Oct 1;23:227. doi: 10.1186/s12935-023-03039-w (PMC10543879; doi:10.1186/s12935-023-03039-w)
Supplement: Supplementary file 3 — Supplementary Table S3. Genes positively correlated with HK3 in immune response. [file 12935_2023_3039_MOESM3_ESM.pdf]

Table S3. Genes positively correlated with HK3 in immune_response.

Gene Name	TCGA-E1-5304-01	TCGA-E1-5318-01	TCGA-FG-7638-01	TCGA-DB-A4XH-01	TCGA-E1-A7YM-01	TCGA-TM-A7CF-02	TCGA-S9-A6U2-01	TCGA-HW-7491-01	TCGA-HT-7855-01	TCGA-CS-4944-01	TCGA-DU-7018-01	TCGA-HT-7608-01	TCGA-P5-A733-01	TCGA-HT-7470-01	TCGA-E1-A7YS-01	TCGA-E1-5307-01	TCGA-FG-7637-01
FCGR2B	0.007784526	0.004906935	0.09581328	0.003113606	0.05048948	0.008522025	0.04350352	0.02210805	0.02429794	0.8725152	0.02853952	0.1277176	0.1094268	0.02273775	0.02527509	0.01272463	0.1670988
S100A12	0	0.1636671	0	0.08308159	0.6315145	0	0.07738811	0.7239902	0.09725274	0.06485145	0.04479596	0.227196	0.4866465	0.0275782	0	0.1697682	0.2197982
CASP4	0.217489	0.6963702	0.3574258	0.3351087	0.6071219	0.2489161	0.3370052	0.3349959	0.5600521	0.4305614	0.4221291	0.4561689	0.5360087	0.4154137	0.2214747	0.4460018	0.4864278
TREM1	0.03314013	0.01175046	0.0084978	0.004970693	0.05541495	0.004534974	0.04630058	0.2310168	0.01551611	0.01552	0.026801	0.088354	0.1289405	0.07919893	0.03228019	0.004062828	0.03005788
IFITM2	2.824179	10.47245	10.06099	6.512616	33.32727	11.73079	6.598904	6.499351	22.47318	8.196878	8.992985	11.88888	12.49795	7.804719	2.682972	7.055711	11.64802
CTSC	1.581165	1.953768	1.177273	0.8176927	1.856026	0.5569335	1.886594	1.120093	1.727604	1.853045	0.740563	1.300941	0.7646329	1.3121	0.7111848	2.698025	1.216213
RELB	0.4963321	0.9832754	1.545417	0.9075192	0.7976018	1.377791	2.496359	0.7276744	2.495331	1.60494	0.9212913	1.01789	1.198417	1.365007	2.054675	0.7591519	1.2862
RNF135	0.7413623	1.231435	2.846367	1.190109	6.079521	1.33804	1.461273	0.4966216	1.519751	1.496674	0.5801077	3.024372	1.483728	1.524319	4.810913	1.31173	0.8995764
LILRA5	0	0.09634042	0.03135254	0.02445242	0.07434648	0.02230898	0	0.2367594	0.04770542	0.1145218	0.05932919	0.08358489	0.04092255	0.06493412	0.01984956	0	0.04620758
LAIR1	0.664348	1.256305	0.8887945	0.4159123	2.650718	1.27701	2.354263	0.7555024	2.39474	1.743958	0.452816	2.298414	0.9682282	2.166455	0.5994957	3.504086	0.7597486
CD300E	0	0.0135575	0.04412084	0.01720533	0.005812447	0.02616192	0	0.04072195	0.01342668	0.004476686	0.03092258	0.007841653	0.0239951	0.01522975	0.01396664	0.004687629	0.004335039
C3	14.71803	19.56608	19.96824	6.924663	15.26686	23.94112	54.61667	12.40716	29.0619	20.26151	4.98887	44.18935	16.15517	59.29823	6.48953	54.14514	23.73747
CD33	0.1500666	0.4114824	0.3509385	0.1152435	0.6058875	0.4205663	0.5568577	0.125532	0.5986211	0.6887278	0.09320558	0.4677956	0.3314898	0.5307753	0.1987946	0.8565863	0.1361094
NOD2	0.2153078	0.1357181	0.08641451	0.09360593	0.07968929	0.07173656	0.1150924	0.3601171	0.2279102	0.09937043	0.4118391	0.1612649	0.03446409	0.1242866	0.6322019	0.1622	0.3169806
LILRB1	0.5135698	0.6871543	0.5930177	0.1937872	0.7423935	0.8486414	1.4946	0.4553241	1.125133	0.5415306	0.1212041	0.7392566	0.4637692	1.175878	0.23911	1.121424	0.4013534
IL1RN	0.01652299	0.01562277	0.04519285	0.07930515	0.06028093	0.02411782	0.1600527	0.09385058	0.04641608	0.04642774	0.1175894	0.1445793	0.05530074	0.06581161	0.05364747	0.05401715	0.0349679
CD300LB	0.01318599	0	0.04057387	0.03164431	0.008017758	0.01443522	0.06632047	0.03574603	0.006173642	0.1111535	0.008530995	0.04326747	0.01323965	0.03151215	0.03853151	0	0.005979804
TLR2	0.4995608	0.9487238	0.8408452	0.7070243	5.623426	0.8553901	2.414767	1.097962	1.983091	1.307729	0.4640861	1.7478	0.5144549	1.190459	1.264323	4.744568	1.115319
CD74	85.11394	95.62198	114.0177	23.47125	129.1032	83.49108	185.0563	55.85619	152.0695	266.5974	46.27379	193.1181	40.62643	122.71	53.70305	108.4279	83.67519
GPR65	0.2101828	0.4199607	0.1382969	0.02855126	0.434044	0.03473137	0.367893	0.147438	0.2896506	0.4680146	0.04105141	0.4554471	0.1274192	0.1832285	0.4094576	0.3111541	0.1402781
FCGR3A	4.223543	8.551474	6.118403	1.268846	7.556883	2.846388	7.18174	3.391772	9.645529	8.721609	1.855218	9.312337	3.965924	5.33167	2.768887	5.661342	11.93223
TEC	0.04004878	0.04628165	0.04107727	0.04271589	0.07576085	0.0243572	0.05470936	0.05859257	0.1166711	0.05835021	0.04606314	0.051105	0.07148753	0.0389927	0.03467517	0.07419249	0.06053996
LCP2	0.7327656	1.19843	0.85061	1.137692	0.9934759	0.8699394	2.332508	0.9050285	1.277272	0.8741427	0.7863967	0.8833875	0.7730317	1.494811	0.6751702	1.89622	1.086733
CYBA	2.879315	6.934903	3.720339	2.596021	6.91863	5.584888	6.478171	2.544617	6.877993	9.001176	2.094401	4.820386	3.754586	4.995884	2.27386	6.863348	2.776254
IL4R	0.7089206	3.699577	1.479218	2.427818	2.208951	1.282369	1.870366	1.515303	2.512863	1.020216	2.049176	2.099067	1.594688	1.519683	1.238796	1.814302	5.888981
SIGLEC7	0.1579996	0.7718551	0.1170413	0.1474566	0.2134929	0.05765611	0.4709197	0.6118893	0.4931661	0.542619	0.1419745	0.230421	0.03525388	0.1398483	0.1367997	0.1893958	0.1353432
HLA-DMA	4.564623	6.764968	5.70532	3.099494	7.194846	4.849871	8.178769	2.943516	10.66252	21.64856	3.159229	12.0985	3.204684	5.496594	4.590936	6.788101	4.480275
TNFRSF1B	2.833273	8.036725	3.990303	2.576919	5.703977	3.096482	5.833165	4.507021	9.493048	6.841707	3.909488	6.972658	4.202657	5.511641	3.576084	8.275599	5.515266
AQP9	0	0.02620698	0.2103737	0.006651663	0.1011204	0	0.111525	0.7513849	0.04152657	0.04672913	0.03227802	0.07730633	0.2282049	0.2605394	0.05399572	0.173977	0.04525058
LILRB2	0.1032913	0.2253777	0.1711401	0.06673767	0.1787566	0.1087279	0.4484705	0.2892444	0.372005	0.4353552	0.2133316	0.3030832	0.1635451	0.1708944	0.1509165	0.1753344	0.1225105
CD200R1	0.02296153	0.03039472	0.03768192	0.03857283	0.08377068	0.03519163	0.0307967	0.06758203	0.0645031	0.04301287	0.03862433	0.03013765	0.01844397	0.07682346	0.03131199	0.004503965	0.04998228
CD300LF	0.2157597	0.5649356	0.2298122	0.03982995	0.8376164	0.2816238	0.5843322	0.3599415	0.2253479	0.6995314	0.1879109	0.2995286	0.1083188	0.2776452	0.1939948	0.5127455	0.06021312
HAVCR2	2.027121	3.494594	2.31611	1.482058	5.0393	3.364879	5.384984	1.617566	3.995015	4.547955	0.8265467	3.116987	1.571495	5.772035	1.597984	6.648665	1.492247
TNFSF8	0.1481042	0.3792617	0.2911564	0.06664252	0.4652838	0.1080903	0.3448644	0.5066511	0.3293742	0.647354	0.1876467	0.2784245	0.1858836	0.4276803	0.1202176	0.6112823	0.1287325
NCF4	0.8155976	1.089266	1.390753	0.8807876	1.822524	1.863859	1.868754	1.058127	3.780417	4.230165	0.8178892	1.814823	1.729961	2.314593	0.8838078	1.779796	0.924677
FES	0.3809218	0.9804576	1.16343	1.21887	3.371356	1.056425	1.036	0.4884572	1.609081	1.018812	0.7557693	1.312423	1.457642	1.308184	0.5400656	1.124934	0.9289937
FCGR1A	1.268107	1.22164	1.368161	1.220174	0.5528455	1.813888	3.202417	0.6625263	2.789382	3.546431	0.3947364	1.938233	1.867859	2.449216	0.2738419	3.631408	1.611316
FGR	0.6607421	1.542334	1.768401	2.558143	3.245507	0.9945927	1.840496	1.491344	1.730468	1.426303	1.583023	1.748883	2.845495	0.9416756	1.231884	1.199871	2.584432
NLRC4	0.4156645	0.542739	0.2791506	0.2196939	0.3731001	0.2491908	0.3816233	0.3142855	0.5606703	0.4912891	0.1600739	0.2110841	0.2832057	0.4099593	0.4241585	0.5872368	0.2019669
CTSS	2.269189	4.218927	2.819059	1.786392	4.08003	2.043055	5.228951	3.984965	5.635828	7.329609	1.701356	4.610217	2.495617	5.449981	2.053313	7.778981	2.815996
LYN	1.101782	2.398775	2.227441	3.284255	2.563401	1.066503	4.235551	1.61244	2.476085	1.906412	2.783766	1.909925	1.903941	2.926392	0.7314653	4.131825	1.888168
VAV1	0.6165103	0.9334615	0.880153	0.3648835	1.230991	1.135504	1.783198	0.7291787	1.482248	1.517735	0.2937595	1.264355	1.049823	2.080607	0.3164864	2.214329	0.6007307
SIGLEC9	0.3460869	0.8280951	0.3187529	0.3220516	0.5325383	0.3402142	0.9394167	0.4650021	0.7539673	0.7607721	0.2604658	0.573605	0.3333117	0.405103	0.2751889	1.295373	0.2882741
OSCAR	0.4130431	0.3254494	0.5913454	0.6918017	1.130181	0.7724646	1.433054	0.3465807	0.6446183	0.7979155	0.2115556	0.931787	0.4060836	1.213308	1.391374	0.8945904	0.3121894
ICAM3	0.04863174	0.03448661	0.03741048	0.03647141	0.05174851	0.05989394	0.06114973	0.1553784	0.09107692	0.0455499	0.01966467	0.05485435	0.04272591	0.02905527	0.04736981	0.02981014	0.09924448
IL10	0.02719861	0.03428901	0.03719613	0.02175746	0.01102543	0.02977539	0.03039968	0.01404437	0.1613012	0.3821252	0.04692479	0.1561829	0.06372166	0.1155552	0.02649285	0.1244852	0.01644597
CCL23	0	0	0	0	0	0.0266015	0	0	0.04550761	0	0.03144215	0	0	0.03871411	0	0.02383196	0.04407878
VNN1	0.04618919	0.09280442	0.03553153	0.04849547	0.1895763	0.05056511	0.2194075	0.08496711	0.06487691	0.4758835	0.08217881	0.2415512	0.01739142	0.03679461	0.07873362	0.2265033	0.1047332
CD14	7.654309	13.55754	9.128476	6.332218	5.561926	2.883371	14.04959	7.59304	23.14952	27.85765	7.099091	34.10193	12.66907	10.83153	4.742004	17.40147	6.57157
IL10RB	13.25418	4.923544	5.14584	2.77326	6.075476	5.646583	3.985077	3.694874	5.364301	5.154454	5.333799	3.994208	4.287461	5.006944	7.062545	5.556283	5.23422
ITGB2	2.757089	3.764378	3.907635	1.766532	5.993513	5.118101	10.82533	3.649748	5.232618	6.904442	1.211936	6.806813	2.715326	7.358181	2.08676	11.42947	2.658598
CCR1	0.8864697	0.9933893	1.31334	0.5515449	1.253718	1.222052	2.88433	0.656095	2.643974	2.091109	0.441825	2.310873	1.925203	3.567453	0.6076249	5.036178	0.7682877
C1QA	25.95485	44.64258	27.80714	16.6461	37.94164	30.26585	30.47498	20.76931	80.54234	83.00547	18.53426	55.53611	27.68835	41.38529	21.34015	54.39554	38.91085
C1RL	0.2789729	1.143019	0.7116747	0.7295736	2.801066	0.5755664	1.283201	0.3628989	0.8741109	1.122225	0.6941855	1.49926	0.5961274	1.142499	0.8221694	0.6770006	1.858772
TLR1	1.372158	0.9347251	0.3041922	0.2657145	0.9088796	0.2207778	0.9900218	0.5053652	0.9960596	0.3814867	0.2276939	0.8758443	0.6471809	1.263173	0.3890252	1.807277	0.3837624
CLEC4A	0.8259518	0.7280057	0.4738369	0.5543312	1.327906	1.18006	1.048334	0.7373242	1.337092	0.7080502	0.8423109	1.286202	0.871481	1.081728	0.872699	1.15331	0.4824923
S100A8	0.03565199	1.904591	0.07313514	0.8342019	3.706978	0.1170889	0.7172632	4.473482	1.068298	0.6678539	0.8880376	1.155233	3.633402	1.150225	0.3646322	0.5070095	1.034756
CCR2	0.004803898	0.004542168	0.02463634	0	0.02921015	0	0.07516992	0.01116251	0.02699005	0.08099048	0.009323992	0.01182235	0.01929378	0.03444135	0.01871697	0	0.02178552
HLA-DRA	62.66726	82.73956	84.13584	12.188	72.88743	27.20859	138.3629	56.3517	106.1064	291.7871	39.84615	161.7744	23.71717	76.94084	39.50684	108.5371	69.85084
FCGR3B	0.02917825	0.187602	0.02394206	0.01400464	0.1135479	0.02555406	0.1891514	1.514192	0.1748629	0.06012424	0.0830613	0.05744591	0.1757819	0.07437947	0.04547378	0.02289357	0.06880764
CLEC7A	0.1540779	0.2711328	0.2633912	0.2978643	2.321361	0.7965212	1.659929	0.1657504	0.5851257	0.3728107	0.1135294	0.881252	0.7090637	0.405722	0.7045411	0.9234741	0.05046441
CD300C	0.3191758	0.2682544	0.3394975	0.2127699	0.3450226	0.4400031	0.8323941	0.23806	0.6641651	0.9189925	0.1300175	0.4848695	0.2611274	0.7251048	0.09211663	1.18258	0.1393842
CD300A	1.815995	3.093223	1.225776	0.8331666	2.59004	2.002659	2.298364	1.334174	2.094349	2.970346	0.9114126	2.048359	1.179837	3.441073	1.287635	4.766959	1.011268
TYROBP	12.3771	23.62985	21.69904	8.967865	32.43594	23.83101	26.36375	11.56214	33.10325	57.48226	8.416998	29.16237	17.82725	28.0355	11.22781	41.01277	14.84838
CCL13	0	0	0	0.0252857	0	0	0.04710581	0	0.01973244	0.07894959	0.08180132	0	0	0	0.2668379	0	0.05733867
NCF1	0.07251916	0.1126476	0.159389	0.05593968	0.6299336	0.2835341	0.370533	0.2768348	0.2279718	0.2231774	0.05362058	0.3739349	0.1976387	0.2682143	0.1210927	0.4267448	0.07517074
IL7R	0.0266331	0.2238405	0.1001626	0.02485593	0.07197459	0.02591671	0.148838	0.1306474	0.0720462	0.252225	0.04977822	0.01699284	0.05942548	0.1390833	0.03458934	0.02612077	0.2120364
CD4	2.637314	7.29589	4.539174	3.267864	8.646232	5.110378	7.719139	4.576419	11.64433	6.193787	4.126448	7.196581	3.363096	8.22905	3.197759	10.12374	6.182358
LTBR	0.736736	1.830591	0.8991721	1.278067	2.146104	1.109765	1.509647	0.5883296	1.0669	0.9815804	0.9551454	1.173596	1.390585	1.310263	0.5145715	1.397517	1.072251
SLAMF6	0.03265286	0.02469907	0.04018971	0.05485321	0.07147659	0.007149273	0.03649585	0.0809316	0.006115188	0.1284512	0.04647622	0.0214289	0.03934287	0.03641608	0.03180557	0.006404947	0.2843129
FCER1G	5.748218	8.653848	10.16984	4.397377	12.01266	7.188861	12.45443	5.84989	16.40908	23.55695	4.958597	16.23697	11.41247	10.5363	4.095105	23.07967	7.3564
CEBPB	1.421067	2.444564	6.131163	6.545648	7.726561	4.506506	8.310478	2.130351	8.911255	5.504383	2.853086	6.167163	5.771837	5.996115	7.706827	7.229417	5.563052
JAK3	0.1395048	0.08959532	0.2321797	0.1800284	0.3008925	0.2247598	0.2971372	0.122324	0.2292213	0.1060107	0.05449419	0.377868	0.1691441	0.5682331	0.1102462	0.2684794	0.3199059
S100A9	0.5594335	5.260154	0.5100447	2.461346	8.693084	0.2381687	2.466361	19.71545	2.531942	3.260331	2.312388	3.594875	8.924945	2.698648	1.120107	1.798425	3.918275
C1S	1.665084	3.028029	3.207902	2.624	5.892494	2.640069	9.13466	1.134921	2.343959	5.253138	3.890725	14.07807	3.705954	4.233479	10.79089	2.011377	10.56147
ICAM1	0.4500174	1.166691	0.9727803	0.7780436	0.5943439	0.3920024	1.124947	0.5359562	1.250587	2.6559	1.621672	1.571919	1.676212	1.83869	0.8578268	1.381032	0.8470475
CCL5	0.3202313	0.2242846	1.240831	0.2561682	0.4038574	0.1558088	0.5832771	0.4776945	0.1221663	2.910509	0.3146087	0.3697194	0.3810777	0.3212349	0.3812377	0.1163226	0.8605856
TNFSF15	0.01591211	0.05373277	0.01165768	0.01909328	0.008293181	0.004977032	0.02794761	0.03345264	0.006385717	0.02129107	0.01617742	0.005594223	0.01369445	0.0271622	0.002214174	0.02006488	0.008246961
C1QC	49.68838	64.75	39.11544	21.38918	39.25494	58.66042	57.15782	38.2154	90.85455	88.58752	21.72688	64.66202	28.59367	69.5228	22.15182	103.0116	49.92828
CCR5	0.05860442	0.1062053	0.2003648	0.03516034	0.2672586	0.1657378	0.447595	0.07565298	0.1783497	0.484867	0.03791553	0.5208122	0.2500822	0.2723272	0.09038255	0.2490673	0.06201279
NCF2	0.753331	1.355083	1.036518	1.499214	2.822864	1.106306	1.834585	1.693541	1.187161	4.158976	0.8955214	1.406784	1.648083	1.614926	1.16928	4.16273	1.838708
IL1R2	0.0350139	0.01655312	0.04189861	0.01400464	0.1419349	0	0.05870215	0.2937984	0.0655736	0.1749069	0.04908168	0.1627634	0.1347661	0.03718973	0.06252645	0.02289357	0.1852513
S1PR4	0.1185472	0.550252	0.2431831	0.4138102	0.3014369	0.1887683	0.3011346	0.3171969	0.322929	0.2523516	0.2788979	0.3978319	0.2272382	0.1717008	0.1679579	0.2325334	0.5180581
MARCO	0	0.008155803	0.07077821	0.05175116	0.1993063	0.009442955	1.465423	0.04676729	0.1292337	0.06463309	0.4687739	0.3608744	0.7967988	0.3366954	0.008401932	0.01691966	0.5945864
CLEC4D	0.03563428	0.01684641	0.0182747	0.01068958	0.02166746	0.00975256	0.01991408	0.0966013	0.04170965	0.008344026	0	0	0.02683446	0.007096625	0.008677405	0.0349488	0.03232006
CR1	0.04536028	0	0.001604316	0.4785978	0.09130383	0.035959	0.01223765	0.05451765	0.008787949	0.007325131	0.03643066	0.01026494	0.006282048	0.08223667	0.1416911	0.009204353	0.0496535
IL2RA	0.01129279	0.2295668	0.005791401	0.00677523	0	0.02472532	0.0189328	0.1618154	0.169192	0.04230863	0.07306142	0.04631908	0	0	0.0274994	0	0.09218238
TLR8	0.1235903	0.1713899	0.0971859	0.01482986	0.2404775	0.04058974	0.2532492	0.2680336	0.2237431	0.2816785	0.01066129	0.1318003	0.07445591	0.2723862	0.03611499	0.2787895	0.1494608
CLEC10A	0.02178974	0.1304829	0.1340959	0.03486129	0.008832848	0.03975679	0.01623615	0.005625713	0.5304981	0.4966166	0.02819478	0.02979129	0.007292799	0.01735784	0.05659819	0.01424705	0.1317543
TREML2	0	0.01027009	0	0.006516696	0.02641829	0.005945459	0	0.07151062	0.01017099	0	0.007027349	0.004455161	0.02726517	0.008652638	0.005290011	0.01065293	0.1132939
IL15	0.004563092	0.01510069	0.02106125	0.04380281	0.06659023	0.03247007	0.07140186	0.02120593	0.03418281	0.06410887	0.01180881	0.05614863	0.03665327	0.02180993	0.02222343	0.01790126	0.03104021
C2	0.4373101	0.615632	0.5282812	0.1865734	0.3545425	0.9397497	0.4960192	0.5519825	0.6855241	0.9102163	0.2011934	0.255103	0.1756358	0.6709233	0.1924719	0.4829071	0.760956
IL2RG	0.246903	0.2641682	0.3532083	0.2105035	0.4266842	0.1778252	0.3558451	0.3019541	0.2798718	0.4929415	0.130314	0.2025419	0.2218121	0.09316634	0.2151808	0.1911737	0.6011003
LY96	0.7681331	3.659349	1.969649	1.347099	1.868256	0.8732478	2.575609	1.647565	1.853517	7.969356	1.968734	3.126378	2.907052	2.047511	7.424456	1.680565	2.411625
C5AR1	0.522614	0.6952442	0.4176089	0.4739676	0.6503323	0.5521681	1.969712	1.962753	1.05272	1.644932	0.9121313	2.333013	1.574222	0.9100899	1.675139	3.403165	0.4795191
SLA2	0.1040723	0.03690079	0.05337247	0.08585399	0.1423828	0.08544898	0.05089033	0.07053261	0.1218158	0.1096618	0.01262476	0.1387321	0.1567436	0.04663386	0.1330504	0.1020703	0.1002924
HLA-DMB	1.564077	3.154419	1.971719	0.7534813	2.64698	1.429692	3.39728	1.557577	4.866342	6.401082	0.7975702	4.199966	1.806394	2.746623	1.455947	5.259393	1.125102
SLC11A1	0.4878028	0.2032521	0.5257705	0.3149842	4.615035	0.7806601	1.455442	0.3746235	0.913551	0.7395427	0.08558527	0.7714935	0.2718736	0.4281041	3.100522	1.301462	0.341199
C1QB	39.8871	45.75237	34.86836	18.73596	33.1824	47.51632	46.2591	32.3866	73.81902	67.32249	18.92144	57.4471	23.25812	59.21213	20.17341	90.62041	47.26115
NAIP	0.05278279	0.05168943	0.01353458	0.02261971	0.08482152	0.1114394	0.1306316	0.04818314	0.1094421	0.03354714	0.03780792	0.03402089	0.04164094	0.1036161	0.04223223	0.07949998	0.06326159
CST7	0.154262	0.1823217	0.356003	0.2545155	0.4924455	0.0844383	0.4094913	0.4331255	0.2166749	0.6321273	0.1746561	0.1740004	0.2323345	0.09216456	0.1878238	0.03782362	0.4547223

TCGA-TQ-A7RP-01	TCGA-DU-7298-01	TCGA-TQ-A7RO-01	TCGA-E1-A7Z6-01	TCGA-DB-A64W-01	TCGA-HT-7603-01	TCGA-IK-7675-01	TCGA-S9-A7QW-01	TCGA-DU-6399-01	TCGA-DU-A76K-01	TCGA-F6-A8O3-01	TCGA-DU-7009-01	TCGA-CS-5390-01	TCGA-HT-7620-01	TCGA-F6-A8O4-01	TCGA-DU-6410-01	TCGA-HT-A616-01	TCGA-FG-6689-01
0.1053894	0.0915185	0.03094193	0.1246344	0.03622096	0.03051727	0.008931514	0.002970598	0.04095059	0.04470638	0.02624871	0.1045317	0.05227523	0.02897123	0.03108878	0.02066613	0.04666889	0.02481002
0.146466	0.05679127	0.04128179	0.1511668	0	0.1163292	0.02979039	0.03963283	0.1214112	0.1897824	0.05002892	0.02681982	0.06064698	0.08589453	0.3258963	0.06127139	0.2490569	0.4965117
0.6294565	0.644631	0.5599441	0.8255632	0.1599489	0.1951592	0.3466541	0.1612733	0.7627354	1.130325	0.2267918	0.2871968	0.3441984	0.4506977	0.2347698	0.188087	0.2815162	0.8231249
0.1261861	0.1155241	0.1383116	0.04974292	0.01051358	0.01739967	0.1033752	0.08536302	0.02905567	0.2627757	0.005986366	0.02246446	0.03991297	0.02055595	0.09926289	0.01832905	0.2135787	0.07261424
24.88004	18.48388	5.571196	11.82432	7.403652	9.226232	6.820629	3.126704	10.10821	10.51245	3.913279	4.330767	7.584017	10.2799	7.429698	2.550605	7.092983	13.76067
1.331251	4.257749	1.735657	1.546068	0.4403483	1.37179	2.788762	0.8718112	3.470541	0.7427122	1.099361	0.7286321	1.160773	1.423897	1.147546	0.7725528	1.282095	2.582214
3.044774	0.974134	2.473072	1.502877	0.9972434	1.111854	3.111707	0.8928943	1.859792	2.036018	1.003298	1.49226	1.211061	1.69324	1.451242	0.6326801	2.458373	2.123267
2.90762	1.848889	0.6331566	1.711542	0.8390718	1.169823	1.245721	0.7626995	2.397939	3.628661	0.6985462	0.6674689	0.9213937	1.58461	1.105995	0.5319315	1.045065	1.863891
0.03448608	0.008357351	0.09719986	0.07785949	0.02585982	0.07703504	0.01753571	0.01166466	0.05360029	0.01595898	0.08098431	0.05525493	0.04462378	0.01264016	0.05231847	0.009016643	0.01221699	0.08118467
2.959197	3.130957	0.9114218	3.031858	0.197369	0.9537884	1.223654	0.8368611	3.748057	0.4245709	0.5859848	0.6833588	0.8524241	2.450415	0.8347447	0.279202	0.7752517	3.522998
0.0404421	0	0.02849676	0.01043502	0.006065204	0.02007547	0.008225705	0	0.01676199	0.01871522	0.03798837	0.02221642	0.008372905	0.01185857	0.04499314	0.004229555	0.01146157	0.01142471
72.99785	56.10697	24.29063	26.43291	5.604452	12.47608	17.63273	19.58812	100.0513	9.231065	11.31913	12.75997	12.90103	43.20579	26.29845	4.897224	26.38683	80.74171
0.7440913	1.009316	0.2648391	0.9992821	0.0875988	0.3050745	0.501037	0.2680042	1.136775	0.126924	0.2060187	0.2162373	0.3969612	0.7334889	0.3827056	0.01859164	0.1871296	0.6600212
0.203304	0.5400358	0.2790669	0.1635032	0.3801353	0.3643612	0.1288861	0.3215038	0.257167	0.2712499	0.1826269	0.1184515	0.6040321	0.3483907	0.2136315	0.6047285	0.306796	0.2585704
0.7242574	1.830669	0.7818706	0.705191	0.1393599	0.3093242	0.6892999	0.4252391	2.341988	0.1998322	0.3220702	0.5354882	0.5403721	1.490592	0.569422	0.08003245	0.5034669	2.269895
0.07922478	0.09938472	0.1050809	0.06012316	0.02795657	0.03238711	0.0473938	0.04413656	0.1352079	0.1121442	0.09153018	0.01706716	0.01929677	0.1503154	0.05184714	0.02924317	0.1056605	0.05704869
0.09483672	0.04866928	0.06289403	0.02159129	0.008366413	0.0166154	0.06240649	0.05283406	0.04046296	0.02065279	0.01905513	0.03064559	0.005774842	0.07361035	0.03385313	0.01750289	0.07114603	0.03151876
7.001659	4.318124	2.372602	6.879601	0.3630227	1.076045	1.78931	1.197572	3.459005	0.6353193	0.2498944	0.2447756	0.7816386	0.9852102	0.2850111	0.347613	2.98467	3.823876
258.2561	157.7608	103.1588	69.43859	21.50589	32.20633	96.39308	45.82454	373.1289	64.91334	49.06672	18.96279	55.74608	139.0488	72.80068	21.19727	76.01304	284.2488
0.3892458	0.8945064	0.3168346	0.324681	0.05032429	0.1499134	0.2866518	0.1407395	0.6606198	0.1459671	0.1146173	0.1013839	0.08683972	0.1672683	0.04411937	0.09826186	0.3518667	0.5655997
8.958007	22.66289	4.724796	5.092638	1.973348	4.875234	7.750503	1.993876	31.2928	2.766887	2.894424	3.170786	6.79953	10.27838	4.029654	1.788942	2.640193	23.63079
0.07906982	0.09489638	0.05306196	0.1165822	0.01694043	0.0523337	0.07658274	0.005094244	0.08583139	0.03484839	0.02250681	0.07584086	0.03507894	0.05520269	0.04950565	0.01968895	0.04801916	0.08154729
1.296608	2.03253	2.057513	0.9755622	0.6000164	0.9337409	1.301574	0.9522383	3.633336	0.7580294	0.524095	1.390417	1.376906	1.360475	0.9491089	0.5301447	2.656572	2.000075
10.01195	7.948172	4.204176	7.994887	1.911639	4.079163	4.523241	3.858894	11.92437	2.664869	2.597674	2.309237	3.574794	7.799924	4.297274	1.437076	3.599665	9.866175
4.011011	2.435684	4.001784	3.630247	1.166017	1.160391	1.887692	0.6931509	2.72894	1.422499	0.7694471	0.9711196	1.953452	1.830614	0.7004202	1.661241	2.560967	3.483641
0.6313164	0.5399757	0.2616736	0.5078479	0.0891107	0.06636401	0.430539	0.08039082	0.6310649	0.02062247	0.1458748	0.1428027	0.4689974	0.3266767	0.2028205	0.1165146	0.2210182	0.6084678
11.53909	11.46415	5.603376	5.546819	4.445359	2.75848	6.260261	4.075174	20.22117	4.672063	3.037391	1.776332	3.031177	7.225343	4.13042	2.010277	5.634281	14.64938
12.60729	9.049551	6.098349	10.28187	1.994077	3.036127	5.765271	3.924976	12.81701	3.016865	3.044976	3.22785	5.476378	6.379824	3.190788	1.883583	7.61074	11.13562
0.09381066	0.9230029	0.1057631	0.06051345	0.2743459	1.019831	0.02862088	0.04442307	0.03888161	0.2821804	0.02403244	0.1331291	0.05341059	0.02750749	0.2229667	0.1226375	0.5981985	0.3091793
0.3193438	0.3519197	0.3647686	0.368624	0.04537213	0.08009551	0.1401613	0.1910172	0.6861723	0.04666781	0.2152884	0.1538842	0.1600683	0.3745565	0.2753848	0.02812457	0.100031	0.3165375
0.174859	0.0602671	0.04928444	0.05013086	0.06410322	0.08872894	0.09484101	0.02628656	0.04428944	0.06473501	0.02654543	0.07826855	0.01206727	0.05696974	0.05895039	0.01625536	0.07158127	0.01829514
0.3440629	0.5445234	0.1088494	0.4529408	0.1684897	0.2718744	0.278494	0.2565038	1.236866	0.1169783	0.1259173	0.1221475	0.3488956	0.329428	0.1278304	0.09546543	0.1392997	0.6149147
4.67901	11.46477	3.687667	4.317652	1.208423	3.001838	4.778711	2.892738	10.60996	1.299541	1.379543	1.986754	2.374304	6.228369	1.948602	0.7968019	4.889367	10.0891
0.6840253	0.8807141	0.4341549	0.5321789	0.01566183	0.1036795	0.2442689	0.2190034	1.417536	0.04349453	0.1471429	0.1721046	0.2918819	0.3444944	0.1689938	0.01092174	0.3329615	0.5654432
2.898478	3.010357	1.033336	2.893568	0.4398669	1.19044	2.044071	1.71568	3.941861	0.950099	0.9207994	0.9477658	1.357334	2.314464	1.518098	0.351849	1.662456	3.289849
2.822047	1.468464	1.075002	1.607855	0.8593493	1.329755	0.7539071	0.5911344	1.7144	3.80183	0.6819731	1.052524	0.9156879	1.249637	1.477809	0.7415907	1.451392	1.821051
0.8553652	5.470469	1.483611	1.560603	0.4933896	2.698368	1.868448	2.088589	4.914015	0.5574463	0.5532217	1.538481	0.8540139	2.827213	1.289987	0.2064384	1.491794	5.79547
5.018648	2.362576	3.410109	2.310363	1.277359	0.9193154	1.82121	0.6145941	1.190295	1.689796	1.327826	1.79157	1.650335	1.32558	1.038137	0.6852026	1.949655	1.961893
0.3517123	0.9700431	0.2124235	0.3997327	0.3202508	0.3034542	0.43007	0.2435932	0.9501331	0.2170139	0.2324053	0.2951808	0.4421005	0.3744613	0.2540853	0.2452208	0.3678583	0.7806632
5.214674	9.720145	3.550508	6.259907	1.081856	3.042856	4.544021	2.126787	10.55001	1.98302	1.848005	2.740417	3.514513	5.44065	3.125249	1.208588	2.695367	9.131394
2.288961	4.846696	1.887736	2.307353	1.048609	2.050836	2.083304	1.07545	5.88491	1.6008	2.103366	1.810427	1.909797	2.906276	1.821264	1.193083	3.462555	5.195609
1.815225	3.109206	0.8792015	1.700688	0.5233235	0.7208627	0.9606553	0.9299215	3.933427	0.4077788	0.5989641	0.8842255	0.627573	1.96887	1.165712	0.1806261	0.7941472	3.216158
1.027927	2.091346	0.8506423	0.8943788	0.1702939	0.1720655	0.996752	0.4204608	1.467622	0.1935945	0.2041353	0.393963	0.5815327	0.8849606	0.3807977	0.1937566	0.8129907	1.64889
1.179543	1.129285	0.9234919	0.6763336	0.9390938	0.6650413	0.7848996	0.6107716	1.644702	0.7480196	0.4165795	0.3999808	0.6029777	1.259649	0.4123887	0.5101931	0.5674689	1.323263
0.07201161	0.04986081	0.05074162	0.0597237	0.05399882	0.0561732	0.1203128	0.07655182	0.04796771	0.07140966	0.03074658	0.02825627	0.0425968	0.02262374	0.04161831	0.04303537	0.05102148	0.0484356
0.09972719	0.2900145	0.06486543	0.07917533	0.01150488	0.01523218	0.1560306	0.03113722	0.09538575	0.0142001	0.006550803	0.02809437	0	0.1687059	0.04655233	0.01604579	0.03261159	0.2383825
0	0	0	0.02652588	0.03083556	0.02041278	0	0	0	0	0	0	0.02128397	0	0	0	0	0
0.170987	0.3457027	0.08950148	0.1008428	0.007326672	0.08730307	0.1043335	0.04626807	0.2733511	0.05878001	0.03337409	0.05814703	0.0505717	0.1002748	0.02470501	0.02554619	0.0138454	0.07360465
17.99786	27.26535	10.79403	21.61052	2.449416	7.31665	16.78147	16.33894	45.19249	6.604952	7.962741	8.780519	8.395138	7.97389	9.032288	2.086736	14.75264	25.81931
3.881412	7.692572	4.352415	4.830566	5.311374	4.445398	6.43936	6.88898	7.444367	4.916745	5.315371	4.372505	4.641293	5.157384	3.744728	6.765493	3.497757	5.08098
7.369275	12.78078	4.766325	8.104627	1.215339	3.628189	6.116265	3.956752	14.16829	1.799652	2.42107	3.084307	2.756971	8.899327	4.567674	0.7587935	4.542702	13.25003
2.316919	8.698255	1.346775	1.763354	0.2166506	0.8715531	2.05677	1.526016	9.004168	0.3599681	0.8350487	1.378584	1.351622	3.519065	1.264379	0.3195939	1.448682	4.975601
54.37305	82.13647	24.34276	64.88431	9.589278	38.44323	54.50738	25.04894	119.9478	16.29622	18.21383	24.77089	31.3287	36.5881	36.87081	6.088219	29.61584	101.5472
3.425773	1.273171	1.646233	1.460204	0.9894193	0.8052096	0.8094264	0.2374808	1.555342	2.428413	0.3592124	0.6400491	1.015008	1.464181	0.5448189	0.4241095	0.9991913	1.689885
0.5922328	2.426083	0.6412833	1.096434	0.1605761	0.3521171	0.8506848	0.3893194	2.253536	0.5233559	0.351438	0.4135634	0.6061355	1.152805	0.4670015	0.1469703	0.5499938	1.909335
1.622811	2.50316	0.7678865	1.405934	0.6928252	0.7291234	1.276917	1.426349	2.417941	0.7126101	1.183471	0.62902	0.6130979	1.146199	1.138128	0.9167272	0.8896185	1.238114
0.7089186	0.1169697	0.2338207	1.089723	0.271451	1.332756	0.276109	0.5509988	0.7970778	5.486335	0.3606458	0.3176256	0.2966638	0.420166	2.288283	0.3312675	0.8335731	3.437195
0.1056846	0.02758172	0.09165376	0.01048813	0	0.008071058	0.008267572	0.005499546	0.01684731	0.00752419	0.01735533	0.007443165	0.06732416	0.02383786	0.01233331	0	0	0.04975905
169.2361	157.8568	85.73635	40.11571	15.92965	24.42118	96.579	97.42034	272.0896	34.22103	28.7798	18.37522	31.09608	86.3797	43.91809	19.72503	50.15993	193.319
0.08888049	0.339842	0.1113386	0.03185178	0.02221605	0.09314285	0.01506484	0.08684916	0.306985	0.03199066	0.02529936	0.04972969	0.03066886	0.108591	0.1797862	0.04647693	0.06997042	0.09299384
2.176491	1.832474	0.2449715	1.588514	0.1031924	0.3775146	0.4750963	0.6908598	0.664183	0.3519354	0.2628608	0.03315672	0.08997176	2.389262	0.2783659	0.06817357	2.098874	0.6206462
0.9802503	1.056872	0.507464	0.774269	0.1650119	0.3177773	0.3865485	0.500727	1.813765	0.231442	0.1879135	0.2197917	0.3831114	0.9238899	0.5058257	0.08368776	0.3968711	1.412838
3.152279	3.564973	1.50468	2.470699	0.9488995	1.710615	2.19464	1.513366	4.957993	0.7006264	0.7525571	0.9516942	1.491225	2.236264	1.554102	1.152089	1.737124	5.548365
37.32405	46.88373	14.16953	33.3247	5.268903	23.75129	26.16706	19.87339	64.01777	10.28131	9.385965	13.67823	12.85007	31.2729	18.57854	3.359321	21.52562	62.60475
0.01783064	0	0	0.02300364	0	0	0	0	0	0.03300564	0.06090477	0.09795065	0.16612	0.02614181	0.03606757	0.2797172	0	0.01679025
0.6705959	0.3908788	0.105005	0.2827282	0.02629306	0.1392456	0.1515507	0.1660412	0.4223616	0.3042439	0.02619943	0.08427094	0.0725942	0.2955943	0.1950483	0.09626086	0.1614817	0.6397221
0.3856067	0.3495193	0.1623206	0.07429873	0.02253133	0.0596619	0.03055727	0.02710205	0.3139361	0.1877155	0.0192438	0.08253069	0.2721608	0.09911888	0.03798701	0.05761129	0.04612621	0.06130381
7.836911	11.59897	4.4513	7.013737	1.906732	2.833452	7.239266	4.121192	21.24956	2.779362	4.373824	4.086257	5.531751	8.805473	5.294178	2.07066	4.494617	15.67589
2.3534	1.44513	1.154326	1.754991	0.9711434	0.9499353	1.147528	0.6505464	1.714982	1.370846	0.6127985	0.6437526	0.9104387	1.530345	0.7477876	0.6165776	1.095639	2.370799
0.1878775	0.06963445	0.1245971	0.1140632	0.008287197	0.01097205	0.0112392	0.02242877	0.09161107	0.08182899	0.01887471	0.0404739	0.06864196	0.04860892	0.03912136	0.005779057	0.0234908	0.0832542
14.90629	29.67727	7.16496	17.48446	2.903597	11.37661	14.83914	9.306561	26.96028	3.918889	4.139945	8.47395	6.810958	13.45417	10.44343	1.991892	8.052064	24.93077
21.90748	4.278827	4.296472	9.988215	4.02546	7.986719	5.2227	5.951125	6.342152	8.220995	1.881353	5.07125	2.866872	3.798769	5.35881	1.046594	9.530755	11.31538
0.5189363	0.3000943	0.4488342	0.2040075	0.5644904	1.236038	0.09512986	0.1627197	0.5561695	0.6926098	0.1483464	0.2141098	0.274358	0.1861237	0.4302407	0.2166218	0.766911	0.8556743
2.761277	0.9686996	1.111818	3.053465	0.7887918	3.655196	1.069769	1.245309	3.896626	9.443732	1.145289	1.974348	1.06169	1.619343	6.3568	1.210137	2.459491	7.701429
21.11005	4.174589	5.380739	8.942843	3.148319	3.30107	2.686529	1.887141	4.248208	8.465025	1.487845	2.728226	3.369033	7.480972	2.441421	2.298616	2.84496	5.592019
2.595854	1.9686	1.627167	3.512711	0.7245774	0.406493	0.9535337	0.4930255	1.561245	0.8526383	1.031425	1.083373	1.051127	1.548744	0.5093499	0.4967182	2.135106	3.265613
2.408552	0.5350474	1.088998	0.2201017	0.03010138	0.1992676	0.1735014	0.2036687	1.237439	1.19819	0.5227558	0.4502253	0.945363	0.3825488	0.8018475	0.07346894	0.2559751	1.162358
0.003846844	0.02610282	0.01626363	0.004962885	0.01730763	0.02291489	0.0371653	0.015614	0.03985993	0.03738394	0.009854853	0.01937119	0.01194643	0.01127985	0.003890671	0.02413886	0.04906001	0.01267835
81.41608	119.8641	40.63755	67.14529	13.19374	32.32971	74.33669	34.29323	176.4952	13.8335	21.34346	40.81426	54.5235	56.35826	48.55781	10.49632	42.55285	143.5058
0.3925703	0.7811119	0.3552348	0.1599355	0.05577609	0.04512825	0.1302762	0.1900908	1.520037	0.05736887	0.08116074	0.1248524	0.1240522	0.3695663	0.1546378	0.05186043	0.1815248	0.2723843
3.099036	4.154471	1.183129	2.841476	0.396376	1.301691	1.591629	0.9816164	3.280943	0.5995518	0.880649	1.584751	1.228495	2.40094	0.9224824	1.756028	1.072158	2.435097
0.05925366	0.04786508	0.04871063	0.05096284	0.0148107	0.0882406	0.08034584	0.01336141	0.02046567	0.1051122	0.01264968	0.009041762	0.04089182	0.02171821	0.1148634	0.04131283	0.02798817	0.1255417
0.647443	0.1944691	0.5654415	0.1999962	0.4239519	0.3711838	0.3338529	0.3454535	0.2929118	0.4895109	0.4282824	0.208724	0.3775858	0.3074962	0.2121246	0.3337892	0.219671	0.1889105
0.1240769	0.3325253	0.0205714	0.05649668	0.1860813	0.01449219	0.03711261	0.01974969	0.2722554	0.2094088	0.4113489	0.4544018	0.1435516	0.2461153	0.6938888	0.01526627	1.251434	0.04810944
0.01507588	0	0	0.03889936	0	0	0.03066353	0.01019861	0.007810606	0.0139532	0.01287381	0.006901474	0.01560613	0	0.03049529	0.01576681	0	0.03549057
0.04499884	0.03335651	0.00932576	0.02219706	0.02381855	0.01708157	0.009421715	0.04476626	0.002742739	0.03307329	0.01130178	0.07149301	0.02877095	0.1028412	0.01472432	0.04982944	0.009377196	0.009970172
0.100331	0.004631278	0.02019896	0	0	0	0.00485876	0.006464044	0.06930683	0.004421883	0.008159628	0.008748531	0.06429421	0.02101387	0.009664202	0.01998651	0	0
0.3172117	0.4426535	0.05403714	0.1708918	0.01568342	0.1245869	0.1488904	0.07545989	0.4081486	0.03548884	0.04167352	0.03510667	0.1479463	0.2402008	0.1269201	0.04374718	0.06915386	0.3446572
0.006145757	0.06553192	0.06062689	0.02378627	0.009216948	0	0.3625041	0.03326011	0.02547226	0.01137618	0.06297686	0.01688052	0.0827049	0.02703121	0.4910464	0.01928225	0.04354376	0.05787162
0.01378608	0.008909109	0.006476066	0	0	0.01368682	0	0	0.009523168	0	0	0	0.009513973	0.02694934	0.004647714	0.009611927	0.006511784	0.008654454
0.1370668	0.06362645	0.06529456	0.06226495	0.02605723	0.07858141	0.01766957	0.01828354	0.04200735	0.07504374	0.01648536	0.03358278	0.03796995	0.0594377	0.04295529	0.006056981	0.04103418	0.05453627
0.3398773	1.35505	0.3128796	0.388976	0.2014226	0.5877818	0.827878	0.6341396	1.201359	0.3247145	0.2457616	0.4517124	0.7121748	0.9885628	0.6181947	0.2207253	0.6563987	1.995125
0.8356627	0.4370047	0.2556779	0.1418558	0.1731484	0.1419132	0.2124616	0.17852	0.5411816	0.4325114	0.183095	0.1560408	0.2959397	0.2579325	0.1779334	0.06324717	0.1480204	0.2484957
3.09977	3.537899	1.409158	1.967285	1.574595	2.605908	1.906683	1.589622	5.387697	1.388194	1.003298	3.547551	3.622833	2.015762	1.820373	0.8627456	3.542325	4.66083
3.064598	3.887825	1.811587	2.155958	0.1927876	0.4441284	0.9987806	1.954888	3.116845	0.7233727	0.7113227	0.8144464	0.910198	0.9197205	0.6864694	0.2312367	5.71247	1.956135
0.1541054	0.06935662	0.1240999	0.1775127	0.0495248	0.05464139	0.1343323	0.05957143	0.04562278	0.1375355	0.02819911	0.02015621	0.07976277	0.1129683	0.1224623	0.080584	0.08578927	0.09846991
4.15611	8.566258	1.920193	3.755415	0.8359584	1.640766	3.557017	1.345132	8.579116	1.080068	1.080023	0.9102607	1.342989	3.612995	1.46709	0.3443176	1.838402	5.767583
5.862474	1.203705	1.924952	4.244196	0.2360648	0.4115165	0.9480115	0.9843706	0.453053	0.9501809	0.1583096	0.1585263	0.2878635	0.4871911	0.2847887	0.1115752	1.261465	1.495385
60.73341	106.3986	32.24291	46.24411	11.75499	36.03844	71.06551	30.76265	146.1302	12.61407	14.46286	31.59475	41.32435	51.30216	41.28574	10.13018	36.82588	127.1953
0.02552108	0.2473909	0.1146413	0.1049491	0.03588244	0.03167167	0.05353064	0.08416508	0.3999691	0.06643283	0.03813831	0.06863822	0.07760493	0.1496676	0.0564634	0.05504958	0.04294506	0.1111478
0.6689558	0.3795855	0.3219092	0.2104957	0.09787804	0.06479411	0.1493363	0.1986755	0.185968	0.483231	0.320454	0.1344452	0.4391363	0.2631328	0.4620528	0.01706375	0.208083	0.2765517

TCGA-QH-A6CS-01	TCGA-QH-A65R-01	TCGA-DH-5143-01	TCGA-HT-7606-01	TCGA-CS-5394-01	TCGA-HT-7875-01	TCGA-S9-A6WM-01	TCGA-HT-A5RB-01	TCGA-P5-A5ET-01	TCGA-DH-5144-01	TCGA-CS-5395-01	TCGA-QH-A870-01	TCGA-S9-A7IZ-01	TCGA-VV-A829-01	TCGA-P5-A730-01	TCGA-QH-A6XA-01	TCGA-14-0790-01	TCGA-DU-A76R-01
0.06896002	0.09112316	0.01659204	0.01158015	0	0.02733825	0.08203454	0.02198585	0.008056438	0.0179315	0.02964359	0.02645658	0.0384959	0.1131772	0.1936826	0.0308872	0.6163599	0.1069695
0.08000381	0.1389415	0.09487116	0.03089978	0.06252801	0.05611364	0.04758609	0.04190409	0	0.209332	0.09887396	0.03921952	0.07337152	0.1473147	0.08203351	0.1873126	0.1183207	0.07511343
0.4883253	0.4711486	0.5237613	0.4455939	0.3191634	0.214316	1.640815	0.2901754	0.2915884	0.4333773	0.6658549	0.2967841	0.874735	0.7887509	0.6822596	0.5134885	3.860196	0.6327499
0.04307897	0.07065826	0.01892017	0.06285596	0.01122298	0.0134289	0.05694062	0.02005666	0.01286164	0.03220493	0.07493012	0.1548668	0.2677747	0.0528822	0.0392639	0.0941366	0.1911334	0.02246983
7.627687	11.43944	14.20473	5.487504	4.387173	11.35925	13.56287	5.683314	7.986653	11.55518	14.38361	7.491666	11.05956	5.576048	14.47198	9.495281	11.67809	17.97922
2.788561	1.080741	2.664921	1.470132	0.8791479	0.7571484	1.614386	1.028204	0.7328838	1.443128	1.367752	1.289632	2.988336	2.091299	1.084565	2.407645	1.50797	1.607997
2.82652	1.837827	2.914592	1.070588	1.334	2.217126	2.745161	1.416096	1.920143	1.393386	2.064419	1.419088	2.335487	0.9365769	1.617128	1.617665	2.160805	1.076882
3.727462	0.5629069	1.445928	1.180337	0.6785512	0.6617177	6.713228	1.182327	1.171624	1.380305	4.334822	1.083883	3.970506	1.284251	1.448098	1.360559	5.564047	1.287902
0.01177329	0.1942417	0.02792231	0.04547183	0.04600783	0.02477289	0.0280109	0.03699941	0.02109017	0.1496248	0.04850067	0.01154302	0	0.05419674	0.01207198	0.0110259	0.1741197	0.02210725
3.776555	0.6754868	3.401648	1.759063	0.6601504	0.7040728	1.862998	0.9628128	0.8393212	1.063285	1.556829	2.323306	4.640733	2.148584	0.6212633	1.916275	1.146674	1.586046
0.0110453	0.02877332	0.01309788	0.008532028	0.0129489	0.04260867	0.04598805	0.01157053	0.02967913	0.04128617	0.04095153	0	0.01519448	0.0813529	0.07927865	0.005172065	0.07350894	0.1140715
79.95793	6.410301	97.64725	31.17612	6.827544	12.9506	46.27719	23.09294	15.77812	12.21687	45.94211	29.21237	72.57415	30.49199	4.352405	57.16586	22.57166	16.20497
1.130551	0.09034101	0.9019879	0.4687974	0.1626251	0.2383726	0.4868037	0.4868028	0.3603153	0.344811	0.431449	0.5508193	0.7664899	0.351213	0.199132	0.8541706	0.3641505	0.4623467
0.1225881	0.134626	0.2622342	1.242163	0.1042641	0.1137993	0.1200955	0.1246404	0.4133629	0.2156335	0.1188254	0.1484705	0.2645307	0.3950223	0.1146071	0.783382	0.3092767	0.3114325
1.645885	0.2074112	1.510648	1.092634	0.2100184	0.3848009	0.95899	0.7350122	0.5315087	0.7282131	0.9778407	1.090433	1.653198	0.6631682	0.2602259	1.020611	0.5133268	0.4730441
0.1527345	1.066534	0.2465213	0.04915873	0.01989528	0.02231792	0.1135577	0.02666624	0.03420027	0.104666	0.01572995	0.01247894	0.09338194	0.7734022	0.1370332	0.05959947	0.1035306	0.02389973
0.02285403	0.145531	0.06624704	0.04707672	0.005953954	0.03205904	0.1087482	0.01596053	0.006823289	0.02278025	0.08787181	0.04481407	0.0698648	0.1332602	0.09373534	0.0998817	0.09013253	0.02145703
2.358246	1.19096	1.244183	1.787346	0.4588514	0.5571149	1.660913	0.8682528	0.3844436	0.398329	1.556825	1.959015	6.338933	2.284728	0.6981051	3.626997	1.707368	2.515173
319.4312	91.14001	272.4642	104.3914	63.05985	31.66408	311.8282	75.28357	19.65519	109.0957	93.2646	102.7909	466.2176	138.2502	118.8052	97.56427	263.5533	66.0679
0.4811375	0.05172666	0.6774133	0.5026236	0.132509	0.06427878	0.566908	0.1296043	0.07387621	0.2706225	0.3775373	0.260573	0.4622633	0.7340651	0.1362569	0.1244498	0.8538871	0.4345184
12.02435	2.63996	5.505818	13.61877	1.600938	2.182787	17.47616	4.058171	4.068507	3.535482	10.81893	2.677756	10.49363	15.14005	3.611125	11.537	12.53227	6.093411
0.097692	0.09822437	0.03658307	0.05560426	0.07635244	0.0324568	0.03058262	0.05386183	0.02302648	0.0538134	0.06778059	0.0504112	0.08959336	0.05680572	0.09489831	0.07222923	0.03041693	0.1158573
2.008025	1.137598	1.659817	1.385397	0.7511648	0.8667093	1.779644	1.147642	0.6046391	1.159013	1.515385	1.20593	2.649538	1.466873	0.9825562	3.560191	1.408725	1.004409
13.52388	4.443291	10.70602	6.336636	1.707758	2.437391	11.77042	3.679147	3.8921	3.123955	4.526152	6.420903	10.22336	5.427101	2.765802	8.751879	17.46321	4.572387
2.028505	4.170059	1.225663	1.947126	1.731031	2.086068	1.547396	1.167442	1.67519	1.085444	1.937789	1.840616	2.553538	1.553628	1.96822	1.741696	1.138661	3.158778
0.1521365	0.3258629	0.5532526	0.3290531	0.2378084	0.09959278	0.2413078	0.2443687	0.0454218	0.3866956	0.1253468	0.08949653	0.2697472	0.4949067	0.1871954	0.5224216	0.1950004	0.3047186
16.08452	6.007107	15.70231	6.120716	3.917673	3.280633	13.68665	4.411505	2.153294	6.272031	5.908584	6.654318	21.18552	9.967752	8.091178	4.931807	12.60148	6.372192
6.949179	7.204812	8.490675	5.248942	4.897989	2.377216	6.074037	4.73781	2.892814	4.047457	4.991113	5.08702	8.926368	7.35533	6.662859	9.29572	3.922177	6.457242
1.249024	0.05561953	0.1468475	0.06926899	0.05006106	0.06289584	0.03809829	0.1140672	0.07458161	0.01915372	0.08443765	0.05651975	0.09986238	0.04127999	0.03283876	0.02399454	0.07578376	0.09621949
0.2386999	0.2192314	0.7185324	0.1276515	0.1937343	0.1931781	0.3767882	0.1634946	0.2137984	0.3500306	0.1248081	0.3150421	0.3746759	0.367683	0.3388925	0.4298978	0.2240334	0.2758264
0.1538818	0.01843065	0.0293643	0.008197738	0.07464932	0.0223305	0.1136217	0.05002737	0.04752714	0.04760227	0.122413	0.0416199	0.07299574	0.07816545	0.09793603	0.02981652	0.1804959	0.05978298
1.131456	0.2664384	0.4927212	0.7332727	0.1199056	0.1479572	0.4106365	0.1707577	0.1460013	0.5591229	0.2686053	0.3290374	0.5803854	0.344291	0.2752925	0.3771563	0.4963337	0.3060846
9.069517	1.40954	10.73986	4.847268	0.7109685	1.661946	4.827536	2.607725	2.741943	2.887175	6.076711	4.021486	7.303553	3.530596	0.8713149	6.861334	2.022058	2.761662
0.5276507	0.1362162	1.10485	0.391064	0.167186	0.1950461	0.4325978	0.3809437	0.20437	0.2611968	0.4934852	0.3914934	0.6997058	0.4398403	0.1096697	0.4607658	0.2530906	0.3548114
4.264353	1.278633	3.948596	1.728912	0.8654388	1.123676	1.989905	1.616559	1.899194	1.805325	1.349085	2.321466	3.1546	1.941347	2.572793	3.86126	1.846718	1.526267
2.963634	1.520289	1.670198	0.8348701	0.6497777	0.9638646	4.613436	1.511297	1.208964	0.778733	1.970338	0.9877358	2.148347	1.476833	1.519411	1.653391	3.406614	1.175436
5.453255	1.026281	5.376581	1.863283	0.3889334	0.7901766	1.965061	1.433573	1.566216	1.147067	1.941839	2.995178	3.283412	1.463565	0.2551302	5.307732	1.870596	1.148574
1.252564	4.221137	0.891199	1.455936	1.174749	1.267596	2.078967	1.174666	1.036417	1.779142	2.059087	0.9883003	1.72309	1.164185	2.128337	1.08926	1.314368	3.292796
0.4116752	0.2283869	0.5921419	0.8082542	0.1653438	0.2446301	0.3400882	0.3833346	0.2970315	0.282113	0.3957141	0.280294	0.7813136	0.7159234	0.228648	0.4337342	0.3720703	0.3113559
8.507955	3.749234	9.379993	4.342237	2.769349	1.918083	7.153513	2.94175	1.997281	3.663806	6.194137	4.1948	8.962259	8.622666	2.462363	5.117604	9.132633	3.76848
6.52619	1.486561	4.430971	2.518015	1.047357	2.213499	3.299897	2.726889	1.371321	2.950346	3.450331	2.069345	4.408994	3.482331	1.216059	3.953281	9.820292	4.563994
3.085285	0.3886972	3.059325	1.520671	0.3498518	0.7933464	1.918145	1.245403	0.8536006	0.9931237	1.61403	1.599774	2.299809	1.329425	0.4935349	2.429643	0.7759144	1.134274
1.020135	0.6661419	1.245195	0.7754031	0.3635687	0.2919279	0.7863794	0.4531055	0.24853	0.4026694	0.786708	0.7041287	1.264886	0.8640741	0.2008351	1.918394	0.6638355	0.8121954
3.022639	0.5784671	1.438454	0.8218095	0.2098169	0.7601459	1.230114	0.8645031	0.6590171	0.9588693	0.6963244	1.442766	2.188471	0.540095	0.3976106	1.061534	0.7058363	0.8868358
0.05619251	0.05489365	0.01665873	0.10309	0.07136671	0.0738988	0.1169811	0.05886459	0.05662174	0.07351457	0.1041696	0.04820677	0.1223936	0.1034699	0.03601132	0.03289083	0.1454342	0.03297352
0.3352232	0.06367565	0.2567309	0.07282849	0.05731207	0.02204262	0.1246188	0.08779098	0.1125946	0.1487972	0.146728	0.05135418	0.01921458	0.1639602	0.04296598	0.01962142	0.09295771	0.0885184
0	0.02438065	0.06658978	0	0	0	0.03340054	0	0.02514817	0.04197987	0	0	0.02574963	0.05169988	0.02878954	0	0	0
0.1667819	0.04634364	0.1740425	0.08760574	0.03128414	0.05147062	0.0714251	0.04193107	0.01792596	0.01496192	0.2583371	0.2027647	0.6913601	0.3009612	0.0752458	0.09996439	0.06906485	0.1064792
17.3151	17.03889	39.84964	10.83763	6.740659	5.347933	23.66936	12.03861	10.34246	15.82732	11.18838	17.1057	11.82915	27.80667	11.34433	37.83369	13.25894	14.18333
5.552736	4.454043	4.24814	5.867849	3.557119	3.546272	8.513554	4.784772	5.090147	5.417135	4.870116	3.360674	4.226257	7.945733	7.14623	6.229613	7.658254	4.823463
16.95298	2.853666	16.79561	7.177658	1.181786	2.473912	8.065426	4.349567	4.002943	4.763174	8.61775	7.278028	14.50195	4.768612	1.487924	12.79621	4.310236	4.533726
3.702615	0.5007183	7.413776	2.391242	0.4150981	1.080295	2.825091	1.804224	1.467894	2.393636	2.725557	2.164728	4.237629	1.117671	1.882716	2.64331	2.390113	1.695402
55.17451	32.46364	71.96345	56.37363	23.5155	19.72068	41.14079	18.6688	26.37995	31.67529	30.48039	26.75221	76.32663	75.87408	48.6736	63.09867	50.97873	36.00283
3.256509	0.5849313	0.986655	0.4564958	0.5781534	1.005804	5.574918	0.6190673	0.7884206	0.5313878	2.499207	0.6361322	1.830586	1.004467	1.368703	0.7430939	4.461686	2.646168
1.04633	0.1547351	1.878316	1.108249	0.4678088	0.4550711	0.7555223	0.8136878	0.3273975	0.7719667	1.21595	0.7705187	0.9386378	1.169455	0.3232681	0.7873491	0.5405964	0.8837049
1.342578	1.207955	1.150896	0.5622746	0.9987397	1.145883	1.924246	1.338641	1.463305	1.535755	0.9062546	0.9832741	4.15371	2.53172	1.393225	1.151307	2.77504	1.230136
0.4737403	3.684435	0.3256677	0.6523354	0.289767	0.3178285	0.294031	0.2373454	0.1844865	0.4003527	0.984284	0.22214	0.5666965	32.18101	4.92095	0.3472173	0.9443308	1.005594
0.02220304	0.004819962	0.02194091	0.01715091	0.01735307	0.01167969	0.1188569	0.01744414	0.01491509	0.02489778	0.01371999	0.02721097	0.07635906	0.3730618	0.1536727	0.01559517	0.09851077	0.04690313
155.8607	70.58003	180.6399	83.08158	57.26364	36.34493	272.4165	41.25896	7.94572	81.13187	90.50953	64.02624	367.3709	175.5555	135.4144	51.24288	250.3797	65.18736
0.08091501	0.1288136	0.04797585	0.109381	0.0737802	0.02364698	0.06417076	0.05650848	0	0.03024518	0.1777779	0.01983311	0.06802326	0.006208023	0.0483979	0.06314867	0.04986179	0.03165372
2.344093	0.4122482	0.9461153	0.7640136	0.1391435	0.2046474	1.588399	0.07252708	0.141742	0.2772775	0.6559977	1.498223	2.381072	1.484292	0.522293	1.171745	0.9727429	1.174692
1.584466	0.4625734	1.82492	0.8440914	0.1281062	0.2778309	1.023681	0.5866573	0.354793	0.6024678	0.9678394	0.4955052	0.5511812	0.3772694	0.3221316	1.662971	0.4444241	0.3847273
6.20241	2.264187	3.671413	2.365754	0.6752839	1.13627	2.119902	1.551606	1.513216	1.94353	2.008498	2.38252	3.254461	2.997245	1.059961	4.522672	1.460375	1.651377
61.4319	21.02811	64.13367	29.15331	9.499018	12.73879	43.10282	15.84298	17.45438	19.02591	21.1756	24.3027	54.85164	30.8349	16.37423	45.85025	38.65264	18.51061
0	0.08457306	0.03849844	0.03761712	0	0	0	0	0.0654266	0.01820279	0	0.02387275	0.04466093	0.2017571	0.02496672	0.02280328	0	0.1600243
0.8558929	0.1039452	0.4021931	0.3837392	0.06081219	0.07556377	0.6764043	0.1191277	0.2090741	0.1118614	0.2465661	0.1936506	0.7574942	0.3471597	0.1595649	0.4035824	0.3009624	0.1573433
0.02051583	0.08610472	0.2649087	0.08980311	0.08284445	0.03357558	0.1952443	0.05731041	0.04900158	0.063905	0.08451602	0.03352428	0.1097546	0.2077721	0.06310895	0.05443812	0.07585411	0.08025733
13.19307	5.775165	15.60903	6.489047	5.501905	3.291665	14.61334	5.233577	3.992912	5.556731	7.971762	6.480814	13.40965	10.15261	6.536726	12.00411	5.917713	3.625145
1.946717	1.24919	1.171196	1.022045	0.6085927	0.8308124	6.802189	1.043839	0.7299616	0.7621953	1.908393	1.16783	2.024388	1.403454	1.444639	1.696444	1.054034	1.657339
0.04527528	0.05241928	0.06561979	0.01748662	0.0884637	0.02646291	0.1346482	0.01580941	0.02027605	0.0846171	0.08703981	0.05178805	0.06920329	0.08336746	0.007737319	0.02826743	0.2231978	0.07793086
26.41711	7.633925	31.58967	19.47628	3.107915	4.522852	24.29161	5.336512	6.930876	12.01965	18.15253	12.51819	13.40476	13.71425	6.722237	17.99647	18.05642	11.4022
9.54479	9.603582	4.304641	2.577667	1.101177	3.930565	5.532297	4.006125	3.396854	2.407532	2.539888	7.405472	10.24969	10.00678	4.898902	10.51632	5.436521	5.430516
0.6143607	0.2614559	0.3269966	0.2278864	0.03327852	0.2197187	0.3798925	0.4109959	0.3459617	0.1409681	0.1703966	0.2236428	0.7503108	0.1904085	0.1808759	0.2648935	0.1214467	0.3769232
1.220994	15.06166	1.277554	1.387009	1.68403	1.057893	1.068007	1.54239	0.4824787	1.691345	3.195497	0.9154394	2.140745	71.38265	8.874262	1.042588	3.452216	3.607658
18.95416	2.595079	5.293053	1.686806	4.81933	7.527607	12.74821	2.159883	3.842836	1.977673	3.949705	2.634566	10.09859	9.399873	6.286544	3.312343	20.43478	23.04299
1.766819	6.719428	2.789116	0.621931	1.03566	0.4784346	2.041939	1.00156	0.7862432	2.165169	1.612327	2.165328	2.42027	0.8287744	5.257206	1.28812	3.836834	1.973785
0.575583	0.4879032	0.6500431	0.3810972	0.3855894	0.1057326	1.97262	0.2584086	0.6505591	1.014264	0.7790902	0.3359079	0.5404356	0.6056271	0.8993303	0.3850315	2.513219	0.5017994
0.01838597	0.0114038	0.02907025	0.01217347	0.01642262	0.01473792	0.0593665	0.08254399	0.02587816	0.05694341	0.03246084	0.01802636	0.02167943	0.03143672	0.005386406	0.004919657	0.06603701	0.03452418
74.30065	33.35766	128.1038	83.00384	24.66212	24.03732	58.45739	33.4131	37.34113	45.53184	52.38044	29.41593	96.41111	83.61125	46.05834	118.1633	50.66191	42.30416
0.3667931	0.07350048	0.8966771	0.2048709	0.02205168	0.09103184	0.7652652	0.1832505	0.1263572	0.3543595	0.4370344	0.08852162	0.6365459	0.2285945	0.1446533	0.1743966	0.3004418	0.3761603
3.821493	2.513355	3.51343	1.853163	0.5309756	1.136665	2.508736	0.7339227	2.072714	2.153232	4.437149	0.8603648	3.095812	2.273894	1.306148	2.816726	1.894384	2.332133
0.09440085	0.4040063	0.0106613	0.02604309	0.0368901	0.1040467	0.02406404	0	0.03623693	0.1058581	0.02222224	0.01322207	0.06183932	0.4842258	0.08296783	0.006314867	0.009972359	0.06330744
0.1618827	0.6920353	0.2165769	0.1250475	0.389297	0.3318933	0.3407095	0.2087146	0.490752	0.1861847	0.2462342	0.07325377	0.3426062	0.3095468	0.5618112	0.09329609	0.4604125	0.2572093
0.6877095	0.07789142	0.1891035	0	0.01557937	0.2167082	0.9959432	0.1357297	0.1785411	0.1937253	0.04927053	0.0879468	0.1005462	0.1559949	0.2146129	0.2426865	0.1768835	1.029332
0.02058717	0.0446918	0.008137649	0.01590272	0	0	0.0122452	0	0.009219745	0	0	0.01009226	0.037761	0.01895405	0	0.0289204	0	0.01932874
0.02349523	0.009416268	0.004286372	0.001396082	0.004237616	0.001267635	0	0.005679804	0.008093915	0.08917381	0.03424871	0.003543953	0.006629995	0.0149756	0.03706355	0.01184816	0.01336461	0.05260233
0.01304848	0.0736486	0	0.005039699	0.1376758	0.004576018	0.01552241	0.01366897	0	0.05365122	0.01075078	0	0.02393353	0.1081205	0.1605542	0.03666043	0.05789369	0.02450173
0.1380447	0.04960143	0.2747115	0.2426836	0.02976293	0.08680662	0.1755427	0.09973039	0.02558146	0.1494611	0.1725657	0.03733648	0.3186849	0.5127587	0.09761855	0.1694032	0.08447983	0.1966443
0.01678492	0.05830026	0.006634705	0.02593128	0.01967771	0.02354545	0.1397709	0.05274926	0.02255085	0.4203598	0.02765855	0.03291326	0.08466402	0.9194788	0.5163227	0.08645665	0.1117074	0.04727674
0.01882585	0.005449097	0	0.004847391	0	0	0.00746505	0	0.005620638	0	0.01551081	0	0.005755065	0.005777485	0	0.005876919	0.009280757	0.01767509
0.07117896	0.03891598	0.02292518	0.02850956	0.02472482	0.03882978	0.09094634	0.03590104	0.059031	0.1261319	0.04995693	0.1395736	0.1063794	0.04126124	0.06487535	0.03703356	0.1871454	0.07672846
2.137226	0.2730139	0.9024984	0.8962944	0.2047747	0.1312629	0.4140918	0.646955	0.5531597	0.349769	0.7277905	0.3082586	1.973781	0.7029913	1.224294	1.314504	5.148104	0.8961102
0.5030095	0.3846312	0.3976568	0.1681799	0.1466916	0.1421749	0.9556184	0.220209	0.2151808	0.3086889	0.4639195	0.250266	0.5577033	0.857094	0.3079229	0.2812404	0.9215724	0.3594831
3.823312	2.519601	3.37337	1.977687	1.28064	0.7422346	5.197938	1.75224	1.43705	2.577509	2.503178	2.644055	6.4492	19.98869	5.110404	3.612572	5.149582	4.006256
2.373329	5.347269	4.107761	0.5532432	0.4664704	0.5614406	1.077532	0.6105132	0.5094229	1.091846	1.209115	1.769278	2.33757	3.671935	0.3743908	4.32039	1.080002	1.285532
0.03006309	0.1435779	0.06535798	0.09869548	0.117481	0.05271466	0.06258512	0.0708585	0.08751233	0.03933043	0.1548081	0.1842194	0.08271262	0.3459785	0.1001838	0.1126186	0.1111536	0.2258034
6.734877	1.376043	6.580587	3.758249	1.068054	1.092739	5.803643	2.009758	1.212039	2.222922	3.535475	2.732038	7.417585	4.88099	2.076743	2.296988	3.96312	2.662178
2.471902	0.7258554	0.4569183	1.079248	0.08213107	0.2010157	0.6278885	0.3952972	0.2139159	0.273174	0.46439	1.016254	1.931863	0.6288721	0.08816049	1.664103	0.7700119	1.879065
64.01374	31.7489	105.3976	79.27061	26.02692	15.50475	49.91504	26.28891	30.7429	32.71307	49.48923	14.65624	84.86762	77.17887	38.0078	94.89466	49.21202	31.26553
0.08277068	0.04161087	0.1325916	0.07907978	0.0459643	0.08555368	0.08809905	0.05476208	0.08193972	0.06513433	0.09152571	0.0726095	0.1078707	0.124334	0.0491356	0.1591124	0.06442775	0.07771117
0.2228063	0.7158473	0.4227377	0.1376867	0.1741371	0.2812922	1.033693	0.04668026	0.1796066	0.2498479	0.146858	0.06553455	0.2043357	0.3692372	0.2741507	0.08346489	0.7908402	0.2719429

TCGA-HT-7480-01	TCGA-R8-A6MK-01	TCGA-S9-A6UB-01	TCGA-HT-7689-01	TCGA-E1-5302-01	TCGA-TM-A7CF-01	TCGA-HT-7692-01	TCGA-E1-5305-01	TCGA-P5-A77X-01	TCGA-QH-A6CZ-01	TCGA-DB-5277-01	TCGA-FG-A711-01	TCGA-DU-6401-01	TCGA-CS-6188-01	TCGA-FG-7643-01	TCGA-CS-5393-01	TCGA-P5-A5F0-01	TCGA-FG-A87Q-01
0.01627432	0.00697978	0.03396996	0.03670172	0.1010219	0.01916652	0.04823032	0.08070989	0.02053543	0.01634365	0.1130423	0.002743569	0.09109511	0.04714159	0.04479185	0.0264582	0.01544823	0.6444192
0	0.03104071	0	0.03060396	0	0	0	0.7976348	0	0	0	0.1830193	0.9594975	0.09930773	0.2689197	0.9177932	0	0.1772711
0.2615714	0.6005281	0.2235422	0.5396436	0.9782228	0.1734245	0.481246	0.5637308	0.3251689	0.2309039	0.4689518	0.4076468	0.7877242	1.290289	0.4351538	0.7938063	0.3089879	2.910705
0	0.01857136	0	0.1391565	0.2035146	0.02039883	0.01833261	0.1288487	0	0.03044031	0.05614484	0.01313985	0.5357878	0.3208404	0.05720609	0.008447801	0.004932441	0.3075729
5.767552	7.825919	4.426162	8.28088	13.06779	6.50996	5.647395	10.61723	3.598596	3.05009	11.29632	13.76286	16.84649	13.21318	7.753958	11.06833	4.151498	11.14355
1.031558	1.069968	0.594246	2.474883	3.855538	0.838916	1.489815	2.07112	0.5685015	0.8142568	3.508335	1.120615	1.34906	3.382584	1.904066	2.902652	0.7272688	4.966795
1.394317	2.585369	0.9001078	2.695246	1.626691	1.192938	0.9413596	1.551958	0.6254315	0.7133091	3.403515	1.286961	4.241453	3.768415	1.637028	1.361605	1.048279	1.792657
1.013189	2.187295	0.9061578	1.988249	4.021074	1.134665	1.197078	1.581144	0.8919575	0.4417076	1.532405	1.1493	2.822924	5.431522	2.390848	2.375041	0.8469442	6.739495
0.03195222	0.009135844	0.07275818	0.009007302	0.01888968	0	0.009018392	0.1056413	0.03023871	0.02139222	0.1282331	0.02154637	0.188265	0.08768421	0.08794218	0.176619	0	0.06521763
1.034696	1.583803	0.8091654	2.56914	5.799793	1.069504	1.640138	2.67225	0.7121514	0.7417172	1.232528	2.29052	1.408151	3.709441	2.14208	4.571882	0.4894337	8.350959
0.01873531	0.004285471	0.03412964	0.02535104	0	0.02941982	0.02538225	0.01651818	0.02364078	0.0150521	0.08328735	0.02021408	0.05740278	0.05484158	0.01237566	0.01949389	0.01707292	0.02447399
25.0033	25.223	11.81161	68.80229	77.33408	28.794	19.94147	34.80581	12.28894	13.83631	20.97136	55.61426	33.60215	224.0987	31.2177	68.00327	10.89933	255.7368
0.280003	0.53283	0.1928853	1.040053	1.154562	0.5911735	0.401126	0.580865	0.3147188	0.2142445	0.5520584	0.4728301	0.4880082	0.7605005	0.3419368	1.202696	0.2036978	1.836528
0.2935579	0.2797822	0.174542	0.284121	0.2198263	0.1114011	0.6269406	0.2264661	0.1790365	0.1736094	0.1812506	1.36919	0.1614356	0.2207914	0.09426202	0.3849864	0.4309891	0.2836121
0.7444775	0.8746189	0.3882533	1.481922	1.46708	1.033848	0.5317473	0.8000053	0.5208262	0.3661954	0.9912408	0.9459911	0.6505181	1.34348	0.5798608	2.265897	0.3807459	3.101126
0.02158934	0.04938294	0.006554791	0.02921287	0.1327383	0.006780293	0.2144915	0.2093791	0.005448416	0.05781682	0.06931517	0.07570345	1.984415	0.2264517	0.03802905	0.1010856	0.05246324	0.12691
0.03618119	0.06502571	0.01569294	0.01165651	0.03055683	0.01623282	0.02917715	0.06835613	0.006522075	0.03460507	0.09573962	0.02788353	0.8040025	0.04412866	0.04552301	0.03361262	0	0.1012791
0.3682113	1.205931	0.53356	2.336408	8.734946	0.5203778	2.743652	3.398855	0.4561725	0.8919538	0.5580252	1.972826	3.467304	5.12781	2.15584	3.108495	0.3863792	10.78414
78.85214	177.5214	36.51132	164.5181	424.1454	56.12762	116.1614	89.9927	48.41739	23.31269	135.7947	107.0059	168.9773	330.0877	167.2179	310.2235	36.98469	527.3602
0.1150336	0.2346793	0.08967395	0.3435602	2.179874	0.06834871	0.2632525	0.5162401	0.0823841	0.04995619	0.3378481	0.2054576	0.3517167	0.9517759	0.1848303	0.3639261	0.07555077	2.736314
4.257193	4.294449	2.309687	17.15605	21.08567	1.998613	4.91091	14.53923	2.387491	5.41966	8.906209	7.41201	14.53879	22.9699	2.882966	16.78211	2.370035	39.89337
0.04884012	0.06383756	0.05295881	0.09047533	0.06599668	0.05478073	0.05513975	0.0666411	0.06162794	0.02335627	0.05600253	0.06586879	0.04522098	0.06382308	0.05376911	0.05898472	0.04238717	0.1424107
0.9218683	1.453865	0.6511141	1.847019	2.358967	0.9020823	1.48557	1.642661	1.082427	1.621604	1.883708	1.591409	3.38482	2.786054	1.64735	2.001686	0.5512044	4.287726
3.295923	5.287849	3.33773	9.128258	12.17132	4.570528	6.099302	6.160084	2.383306	2.467402	4.845712	8.093783	5.507636	10.39963	4.725877	12.60414	2.85591	20.43322
1.41687	1.53316	1.198564	1.452416	2.995171	1.015061	1.720808	2.590229	0.7163438	1.964281	1.375549	2.493149	7.297043	3.237998	2.379184	2.798322	0.9564082	3.715745
0.2546168	0.1888879	0.1149126	0.4888544	0.6834679	0.08644791	0.2486127	0.7482853	0.1562999	0.1566459	1.011225	0.06496604	0.3973563	0.4196558	0.1969767	0.662315	0.04180624	0.9214105
5.215248	7.297138	3.714285	12.15552	19.51585	3.223502	7.472667	7.793906	3.521406	2.176705	8.577626	3.322256	8.806703	24.87485	6.932648	15.52155	2.570057	37.27051
3.639804	3.825507	2.312188	8.086385	13.75385	3.511393	3.498159	6.703394	3.099561	2.879259	7.283601	4.470843	21.85954	9.802916	4.841443	11.91989	2.131852	16.30004
0.05215076	0.05964421	0.01979202	0.04900418	0.2723383	0.1910806	0.01471935	0.5364248	0.1809649	0.3666105	0.1180641	0.1465286	0.2663063	0.2650253	0.5262938	0.2034836	0.1716124	0.219986
0.2491622	0.2386572	0.09928897	0.2844663	0.2909194	0.3521306	0.2918491	0.2059467	0.1414802	0.1209414	0.5653601	0.09661021	0.403723	0.1063624	0.2228752	0.5630598	0.07095438	0.6051918
0.043203	0.03705807	0.01093081	0.05683481	0.02554097	0.02261371	0.04064627	0.01587099	0.05905776	0.0289247	0.08446989	0.03398864	0.03394074	0.01756429	0.06738105	0.02809516	0.06014799	0.1822419
0.1626443	0.2827419	0.118514	0.5281838	1.384602	0.1123753	0.2864518	0.5353492	0.1067194	0.1829377	1.140783	0.1403854	0.1226641	1.404462	0.2220326	0.5923038	0.07904687	4.59982
2.819926	4.25225	2.272259	8.205642	12.95661	2.257574	3.705222	8.189638	1.716177	2.538724	3.386357	4.83989	5.831663	14.33436	3.763666	11.60855	1.178768	24.04891
0.2951127	0.2711202	0.1689178	0.7255434	1.561616	0.3266673	0.2020914	0.6824636	0.08546476	0.1425167	0.2867576	0.1500683	0.5244989	0.8555865	0.3675051	1.075974	0.1322593	1.327154
1.66246	1.599682	0.849328	4.425099	3.392624	1.731993	2.950691	2.501599	0.806824	0.9953039	2.368726	1.940275	1.723604	3.041447	1.143898	4.750634	1.007539	6.877846
0.7499006	0.6944708	0.609495	1.5677	1.34176	1.615236	0.9665026	1.677273	1.143033	0.6708989	1.5898	1.145614	2.064562	2.687207	2.027423	2.321801	1.269964	4.139449
1.317736	2.880076	1.117664	3.791359	2.639262	1.207661	2.673631	3.76929	0.7929172	1.864934	1.887797	2.896614	3.591982	3.912155	1.600442	6.690772	0.6481257	4.755187
0.9146024	1.508861	1.204119	0.5019613	4.866294	1.02948	1.978335	2.194322	0.9906645	1.273435	1.449231	1.490011	3.819927	2.561705	1.875695	1.842211	1.432107	4.969305
0.3995791	0.4303729	0.3827969	0.8092653	1.022878	0.4081497	0.4160803	0.5358504	0.4846204	0.2908965	0.4742599	0.4028647	0.2834366	0.6576853	0.3630284	0.812342	0.2828151	0.9501906
2.65019	3.114657	1.736468	8.177641	13.55231	2.375797	5.19944	6.430204	1.938482	2.604711	8.163018	4.143752	3.291521	10.46754	4.821261	8.986939	1.489829	14.27458
2.420826	2.006961	2.139416	3.811348	5.756877	2.055965	2.281413	4.41566	2.208542	1.756203	3.011823	2.473948	3.383109	6.442998	5.417864	6.474491	1.860795	9.843317
1.123412	1.064469	0.679189	3.06378	2.89984	1.40511	0.866436	1.367652	0.6510864	0.865826	1.471937	1.427015	0.8043138	2.899678	0.8628752	3.682538	0.5902327	5.428531
0.553721	0.6459499	0.3698565	1.573422	2.02958	0.2695454	0.5813826	1.0903	0.1397404	0.5264223	0.6837647	0.4406016	1.546458	1.803184	0.6278912	1.685211	0.1934259	3.68897
0.809538	1.18047	1.607849	0.9660807	2.544486	0.7309442	0.6930833	0.2973907	0.6809997	0.5961902	0.5331527	0.9826136	0.6916301	2.155734	1.121474	1.456504	0.9323804	2.291254
0.0524233	0.07085699	0.1446942	0.0107477	0.0394442	0.04490163	0.1076093	0.1050445	0.04209502	0.03190705	0.0470801	0.06427391	0.09547271	0.1511273	0.1154278	0.1115711	0.05790518	0.2879303
0.04975371	0.06503169	0.06473933	0.2564668	0.1092507	0.1004498	0.1203668	0.1253311	0.1076241	0.03806907	0.09654633	0.02875754	0.4606688	0.1560407	0.01564997	0.2495966	0.04318005	0.1740895
0	0.02178738	0	0.02148083	0.02252427	0	0	0	0	0	0.0235241	0	0.06734685	0.0232346	0	0.07433034	0	0.06221302
0.01810557	0.2018945	0.1236842	0.02551972	0.668984	0.03553869	0.06643297	0.1928859	0	0.0424263	0.03912605	0.08546402	0.1600192	0.3422802	0.1893616	0.08241909	0	0.5986751
5.583337	5.908169	4.990455	29.41939	27.56971	6.063466	16.36891	30.96621	6.584321	12.82626	25.719	4.815602	40.67164	18.52	4.628575	27.16612	3.860665	44.09957
4.524284	4.9027	3.983356	6.191644	7.03453	4.476049	7.215847	5.527938	6.949636	3.920367	6.917028	4.634035	4.23134	8.071618	2.724583	6.257943	4.698397	8.47906
4.19887	7.051899	3.730289	14.21789	20.26934	5.001915	4.791835	11.06642	2.150625	3.090904	5.018971	6.812164	5.537879	12.19498	5.979809	17.08275	1.778069	28.67127
1.770885	1.089209	1.148785	6.333014	4.838966	2.23919	2.17366	3.948687	1.143262	1.323482	3.477243	1.70793	3.755098	3.553745	1.394194	7.090478	0.7740392	7.640994
22.13847	21.1354	15.23498	74.31647	140.1578	27.71728	64.94944	94.49101	18.13055	25.5442	64.69349	45.2555	42.44726	107.1022	25.18797	115.7348	9.450848	141.9267
1.233735	0.692677	0.3745774	1.890707	1.790262	0.5239568	1.190269	1.495637	1.142807	0.6607953	1.1807	0.6882461	1.268818	7.58865	1.830544	0.966413	0.9155954	8.355765
0.6324214	0.3900109	0.2870753	1.30738	2.316578	0.5257501	0.5459955	1.799383	0.4107397	0.3486909	1.757134	0.7191308	0.8913841	1.928344	0.8430038	2.157137	0.2966284	2.693035
0.8121501	1.317958	1.13293	0.965279	1.518252	0.930631	1.672732	1.225655	0.8863097	0.9699144	1.897354	1.169324	0.9441176	1.378731	0.5316348	1.35605	0.8167637	3.100307
0.06987563	0.5594115	0.04243022	0.3466826	0.2974279	0.2194496	0.1893324	5.934768	0.299782	0.5426735	0.1553152	0.3015635	5.187582	1.312455	2.338598	5.94362	0.7004311	1.48328
0.003766134	0	0.01143445	0	0.1558537	0.01182782	0.01700763	0.02213634	0	0.005042903	0.06510876	0	0.09763759	0.1791423	0.03316973	0.05877931	0.01143988	0.1906388
81.586	111.511	21.29238	129.3494	370.6391	21.02455	43.98382	84.0714	37.25409	12.66286	112.1941	51.52006	139.2432	452.4432	131.3317	209.0806	18.51665	464.8174
0.07320001	0.08371799	0.006945135	0.1341276	0.1135962	0.05028846	0.02066042	0.3294105	0.046183	0.04900789	0.02824729	0.03085064	0.4366916	0.1004388	0.2770193	0.4581729	0.0625359	0.3212281
0.285206	1.013098	0.5195525	0.8626372	2.035216	0.6428042	0.39018	0.981167	0.7197631	0.3639231	0.2983238	1.031757	1.000366	1.65742	0.8828639	1.34412	0.2598996	6.64591
0.4170428	0.6359559	0.2813764	0.9300618	1.665579	0.4802432	0.6173169	0.7217622	0.07016497	0.5584259	0.7095377	0.7249351	1.059348	1.243365	0.601972	1.157143	0.168906	2.19427
1.277137	2.352599	0.9455494	4.721624	5.712193	1.405475	2.381446	2.79193	0.8321837	1.633009	3.225254	3.211901	2.325347	4.111227	0.950797	5.520979	0.6280158	7.820288
13.12895	25.29753	11.29477	55.23154	81.1902	19.35052	39.72467	44.84792	10.09246	13.86461	27.9457	35.72658	31.28978	52.91602	21.67237	82.65665	11.79451	104.3551
0	0.3778869	0	0.07451399	0	0.02594199	0.03730287	0.02427584	0.02084611	0.02212122	0.02040045	0.02228061	0.03893613	0	0	0	0.1756378	0
0.1624376	0.1997113	0.09863616	0.4258565	0.5617796	0.2423201	0.229237	0.1909531	0.117857	0.1196284	0.1805281	0.4162395	0.220132	0.8618144	0.1877726	0.4859169	0.1541922	1.598077
0.06727893	0.01591989	0.03521848	0.04185571	0.4142013	0.03643009	0.1231025	0.1943149	0.0468384	0.06212923	0.171889	0.1032519	0.3417352	0.2631491	0.21199	0.09655585	0.1057056	0.3068457
5.491069	4.943363	4.060784	9.806422	12.85438	5.900912	10.84017	9.578841	3.503107	3.910441	7.068563	7.471789	10.96415	11.08596	6.794977	16.37016	2.860672	24.08866
1.018604	0.8884463	0.6864912	1.438873	2.514613	0.9350931	0.9907594	1.384925	0.889827	0.5305816	1.456868	1.473387	1.656732	2.563879	1.816426	1.811056	1.288632	3.845597
0.01535941	0	0.01554436	0.02309228	0.1392305	0.03215824	0.08670267	0.1278946	0.04522225	0.02056645	0.06322209	0.006904881	0.03619954	0.06244404	0.1747314	0.1198597	0.03110348	0.3176806
7.612848	15.87893	5.556994	20.35874	25.1413	6.114587	16.33339	15.6655	6.90095	8.690036	19.43183	14.86968	15.02058	27.07255	8.766485	26.10013	3.41109	54.4624
2.645043	3.888245	4.015339	7.075429	7.818549	4.582369	6.889387	3.876155	2.6211	6.44829	4.002921	9.112042	12.32385	10.01126	6.83683	8.198454	2.205119	14.42417
0.280643	0.2171255	0.07204979	0.6259235	0.4684573	0.5767857	0.2166638	0.3759994	0.580659	0.3840741	0.5300225	0.3478791	0.2553308	0.8506871	0.8064916	0.5904533	0.6362197	0.8457519
0.4142159	1.254001	0.3328971	1.181408	1.065941	0.3826107	0.4676435	17.3648	0.7686327	1.566044	1.955721	1.084413	17.74455	4.606249	6.706163	15.11624	2.183362	4.058187
4.488342	2.946091	3.788259	8.115325	16.37339	2.042994	5.148327	6.44187	8.804135	4.967021	5.163638	1.757586	4.068762	17.68049	5.547298	2.277876	2.88137	21.15901
0.6524943	0.6464608	0.3858448	2.54946	2.906543	0.7148398	0.4411371	1.49394	0.5361265	0.4114511	3.400953	0.5423219	58.51314	2.489249	1.357337	1.425915	0.7720561	6.88822
0.3998254	0.1595147	0.2964222	0.7234436	0.7915674	0.1168076	0.6928415	0.7651386	0.4341148	0.2614605	0.562618	0.1128619	0.7341341	0.8278709	1.729995	0.2902423	0.7908347	2.307807
0.01069259	0.01222899	0.02434803	0.01808539	0.0021071	0.05037133	0.0160957	0.01833074	0.04272542	0.0310213	0.04621327	0.01442069	0.05040129	0.02390905	0.03335315	0.009271271	0.04059932	0.0116398
35.57395	40.276	26.69798	118.5817	205.6261	59.97899	90.24066	143.8065	23.45406	35.78895	93.04601	67.83281	48.98219	141.724	45.48277	147.0628	11.86785	201.1927
0.3637262	0.1445016	0.145305	0.6821216	1.294704	0.3066199	0.09941844	0.3881953	0.2560518	0.07177347	0.3120403	0.175563	0.4737388	0.8078579	0.2023245	0.5328222	0.1221142	1.744252
0.955511	1.603515	2.850027	2.403916	7.164658	1.055577	2.759234	3.175012	0.7391685	1.806649	1.583104	1.832619	2.019991	4.860697	1.617559	4.271558	1.137635	8.796896
0.0366	0.04185899	0.006945135	0.03095252	0.08114016	0.007184066	0.02582553	0.3899144	0.1096846	0	0.1073397	0.01234026	0.2210414	0.1339184	0.09569757	0.279664	0	0.01494084
0.1605292	0.2125841	0.6412981	0.08574258	0.2297638	0.4245507	0.8107881	0.5462648	0.3198325	0.1923243	0.2921286	0.1025524	0.796506	0.2576192	0.3255552	0.4835046	0.5132821	0.8139662
0.1555346	0.04640429	0.09239134	0.03050092	0.00799563	0.4566115	0	0.1093055	0.2133242	0.2173178	0.2672175	0.03648062	0.14344	0.1154689	0.4318007	0.008795221	0.03081174	0.7508654
0.02095228	0.03993812	0	0.01575047	0	0.01096703	0	0.05131332	0	0.01870358	0	0.009419177	0.01646033	0.0340728	0.01537787	0.009083589	0.04242928	0.01140418
0.002452504	0.0168294	0.01303068	0.04977781	0.01884849	0.003851136	0.06506772	0.01801896	0.006189287	0.003283935	0.05602696	0	0.02167552	0.008973642	0.05400025	0.02551803	0.01303687	0.06207194
0.00885326	0	0	0.009982904	0.02093566	0.06951081	0.04497838	0.01300928	0.01117131	0	0.08199363	0	0.02086565	0.01619692	0.04386033	0.005757325	0	0.0289126
0.06782415	0.10712	0.0833496	0.2330766	1.042512	0.0557875	0.1130355	0.1898345	0.03260288	0.01729856	0.2193529	0.06969282	0.1560442	0.4057328	0.1600051	0.6216906	0.02943148	0.411352
0.0170826	0	0	0.03852455	0.06732651	0.02682459	0.1285733	0.1422433	0.007185113	0.01524922	0.3234492	0.03071819	0.295246	0.0486148	0.02507545	0.1110891	0.07783428	0.04648968
0.004257715	0.0146085	0.01292695	0	0.02013678	0.01337167	0	0	0.005372515	0.01140228	0.01051532	0.01148444	0.03512152	0.02077182	0.02343704	0.02768817	0.006466547	0.02780933
0.03040746	0.09410152	0.05702168	0.0907606	0.08459499	0.03089605	0.03231017	0.02365505	0.03159804	0.01916046	0.03092252	0.04100937	0.01475461	0.1287126	0.04332215	0.02559006	0.05433215	0.505279
0.5358461	0.322395	0.1349321	1.875653	0.9278325	0.6300774	0.8744712	1.291171	0.1281795	0.3060448	1.110137	0.6952751	0.6045158	3.246062	0.5423948	2.028026	0.3857033	2.276585
0.1884718	0.2679848	0.2010516	0.1493385	0.5781883	0.1679744	0.4198127	0.4191629	0.2571021	0.1705177	0.6856252	0.1167876	0.450199	0.391402	0.4262009	0.4770069	0.3249299	1.938003
1.852913	2.119155	1.336098	4.048092	6.408175	1.163842	2.484144	8.236269	4.179285	2.263981	3.146106	1.686793	2.838553	11.49734	2.090909	4.458351	1.160846	9.076747
0.5478761	0.7519193	0.4917937	1.283914	3.064339	0.8079555	0.7691457	1.42112	0.3606922	1.569295	0.8236225	0.5332927	11.34617	5.229556	1.164377	3.023792	0.2387779	4.527531
0.05099375	0.1399703	0.0232235	0.0632504	0.04823479	0.05605239	0.1899848	0.05994557	0.0836491	0.07510944	0.08186079	0.1375466	0.06009185	0.09329235	0.1122802	0.1260138	0.07744845	0.2581265
2.011445	2.790956	1.503825	5.946096	9.370253	1.70258	1.998111	4.380275	1.730178	1.083811	3.662361	1.67409	3.879352	10.88871	2.347442	8.712506	0.9724464	21.41301
0.1215333	1.234287	0.4354086	1.220575	6.033375	0.2010205	0.7226355	2.538292	0.1881148	0.394903	0.2581306	0.8413911	2.134886	3.687938	0.7528402	1.502696	0.2264216	9.731949
30.04107	24.7865	19.6494	96.137	171.4517	38.26525	84.1999	118.1587	20.94234	30.79518	85.01471	55.62704	41.75462	150.6077	43.23349	125.0616	8.575691	171.8376
0.09901728	0.07944045	0.06730495	0.08332204	0.06814818	0.05569633	0.08843011	0.07166388	0.06713356	0.08311327	0.1204469	0.06577384	0.07314492	0.09733473	0.03254036	0.1672257	0.02693477	0.07963502
0.1814061	0.2074721	0.2753858	0.05113823	0.5719713	0.09495327	0.2218718	0.4220603	0.2289038	0.2833895	0.5040228	0.1223278	1.31826	0.5715712	0.7655697	0.7668014	0.160718	1.727917

TCGA-HT-7609-01	TCGA-QH-A65V-01	TCGA-TM-A84H-01	TCGA-DU-7008-01	TCGA-S9-A6WD-01	TCGA-P5-A72Z-01	TCGA-DB-A64Q-01	TCGA-HT-7468-01	TCGA-E1-A7Z2-01	TCGA-DH-5140-01	TCGA-TM-A84G-01	TCGA-HT-7485-01	TCGA-TQ-A7RF-01	TCGA-19-5960-01	TCGA-QH-A6X5-01	TCGA-TQ-A7RS-01	TCGA-DH-A7UR-01	TCGA-DU-6407-01
0.01909348	0.0180661	0.01482329	0.0380975	0.04483213	0.01987312	0.04159935	0.09518358	0.005065119	0.2138205	0.01388716	0.1084727	0.1326075	0.09248937	0.04624666	0.02069046	0.03376857	0.01015698
0.09552729	0.04017208	0	0.09681625	0.07036904	0	0.1189299	0.1154464	0.1013659	0	0.07411133	0	0.08424809	0	0.4627568	0.1380229	0	0.1016335
0.575121	0.5993812	0.3694338	0.2522747	0.3189979	0.2607887	0.3565929	0.4841956	0.417301	1.692218	0.2301475	0.4617555	0.5352819	0.6658703	0.3386173	0.2414069	0.9488078	0.3531027
0.2552838	0.009613831	0.007888178	0.05213183	0.004210113	0.03172629	0.06641097	0.03798878	0.0283016	0.03519095	0.01773604	0.007068181	0.1260121	0.1519967	0.03230069	0.008257781	0.004492465	0.0689139
5.825947	18.32768	8.819149	3.177085	5.740527	4.361512	4.498318	8.090415	8.269174	9.873079	4.3913	5.959006	10.73336	11.31888	46.87744	21.36072	17.29261	8.13178
2.406062	1.778278	1.886642	1.165839	1.026097	0.7616143	1.956249	2.388961	0.8275423	5.70364	0.6033574	1.9946	1.73859	3.3761	0.9532212	0.8169347	0.812244	1.857666
2.874967	2.639709	1.862102	1.400422	1.459248	1.201721	1.339695	1.57631	1.643564	1.305068	1.030892	1.673573	1.632026	3.307778	1.513807	1.63722	1.678861	2.278154
4.054484	1.534529	1.445087	0.8195036	1.364373	0.5456213	1.210316	1.014784	5.817876	3.008534	0.8096145	1.598284	3.693596	4.279848	1.121539	1.402957	2.482593	1.475465
0.02811542	0.04729349	0.009701114	0.007123696	0.01035544	0.0520239	0.02333548	0.04247248	0.009944613	0.06059041	0	0.06954126	0	0.07477203	0	0.03046699	0.01104993	0.01994173
3.517139	1.892443	2.593567	1.225653	1.072624	0.3006313	1.452808	1.255893	1.125487	2.080045	0.3971673	2.636726	2.579175	1.753967	1.472621	0.8659052	0.4072224	2.32432
0	0.05546145	0.004550629	0.006683211	0.03886051	0.03253805	0.04378512	0.01992312	0	0.01218083	0.02046358	0.01631032	0.0174469	0.02505307	0.01064801	0.0190554	0.01036667	0.01870866
76.09042	37.21058	27.59552	21.99217	20.55193	5.132592	17.94119	21.781	24.35179	26.33966	5.066072	55.71635	44.7986	27.98077	22.31432	10.0423	6.195392	59.16794
1.098705	0.3134431	0.7486825	0.3986885	0.259276	0.1123774	0.4124228	0.5354589	0.2167676	0.4971818	0.2184517	1.129187	0.7997717	0.3052022	0.4045282	0.1615388	0.208312	0.5609717
0.1722051	0.1231096	0.4931751	0.4319585	0.2600483	0.08762683	0.09647659	0.1873016	0.05481909	0.3101439	0.136939	0.1969953	0.1632627	0.1373921	0.1703163	0.4603005	0.4466887	0.05191026
2.00535	1.075688	1.020993	0.6277885	0.4759923	0.2061466	0.4845306	0.7405144	0.2774174	1.100306	0.2350952	1.468733	1.108305	0.8499201	0.7231825	0.3766668	0.2837317	1.03674
0.222897	0.09586519	0.01048769	0.04620776	0.1007557	0.04218154	0.02522754	0.1147905	0.02687733	0.03743037	0.005895219	0.06108354	0.1809419	0.1212519	0.04294523	0.0109791	0.04778349	0.07006554
0.218308	0.01530084	0.01883157	0.03226615	0.02010174	0.01683126	0.007549714	0.03847507	0.01930425	0.04480635	0.02117077	0.02249864	0.08022153	0.0552936	0.03671998	0.06571312	0.007149956	0.03871042
6.334627	2.853825	1.784635	2.074692	1.345221	0.07630173	4.703543	1.68013	1.825263	1.606841	0.1462464	1.737538	2.374252	1.454748	1.726469	1.080949	0.1991092	1.616989
256.8077	83.88357	78.00719	72.39894	43.10831	32.17421	60.16742	53.73473	65.59047	258.6267	36.40025	158.2186	322.7191	172.9005	167.29	28.64663	69.16702	79.86212
0.6602128	0.207079	0.5625874	0.1303123	0.09269977	0.0944913	0.2588473	0.2942454	0.1354684	0.6367219	0.06791626	0.4127571	0.8203103	0.432371	0.09276626	0.07905337	0.1032174	0.4074781
12.34647	12.97774	3.665897	5.079445	3.698219	2.514278	6.268122	13.17162	4.462141	13.62778	1.085244	10.38512	7.75024	13.92217	5.847889	2.975895	3.237941	5.63653
0.05730057	0.08778048	0.06778744	0.04044421	0.04974724	0.003786686	0.04586029	0.06306583	0.04343057	0.04914245	0.0333409	0.07972224	0.1245325	0.06530957	0.03965393	0.0487875	0.05790932	0.05225426
4.982013	1.519607	1.072426	1.978271	1.270557	0.657265	1.204786	2.356882	1.080015	1.57515	0.6279922	1.307307	1.382592	2.172205	1.621438	0.9178791	1.581281	1.603707
8.61324	9.942763	6.291477	4.44814	5.027372	3.295725	4.39822	3.251036	5.688188	5.858594	2.750287	7.934374	8.807401	4.048935	6.224173	4.760002	5.764266	8.5156
2.840481	1.811179	1.610642	1.301845	1.796583	0.999405	1.693267	1.800937	1.912389	1.669709	1.074702	1.936395	1.836248	0.9090516	1.745414	2.080343	1.874176	1.851935
0.1695457	0.3259385	0.2841481	0.08591672	0.2051828	0.1718003	0.6935537	0.6805575	0.1199389	0.6338246	0.1785129	0.2620987	0.6194688	0.3864869	0.09777616	0.3499553	0.08567348	0.6957643
14.99075	5.157118	7.533026	7.723503	6.760871	4.046942	5.316142	4.631411	3.529513	15.5047	2.999666	11.8275	14.55258	11.19532	7.425841	2.828517	5.564212	6.703695
11.11159	4.718801	6.560282	4.790695	3.377975	3.314647	5.409171	4.152423	3.002291	7.342008	3.450585	7.328608	8.48823	4.098422	4.344151	3.611813	7.926844	8.99803
0.04588853	0.1865425	0.03166731	0.1123936	0.04507099	0.004717263	0.05078262	0.03234998	0.1839523	0.02354584	0.01186698	0.03783389	0.04721539	0.08135937	0.04322401	0.005525183	0.1683279	0.04339715
0.2630915	0.0875883	0.4311988	0.2138687	0.1897657	0.04394871	0.2001661	0.1755353	0.04652863	0.438733	0.131821	0.3897638	0.3915464	0.2707108	0.1991372	0.1385886	0.1637172	0.2799087
0.02534348	0.005328844	0.03060633	0.01284272	0.04200518	0.007815798	0.0210348	0.02679954	0.1299803	0.03120955	0.00491545	0.02350691	0.04470219	0.08184303	0.02046163	0.022886	0.05976301	0.01797564
0.4503324	0.3466585	0.3002362	0.09282891	0.185545	0.1765426	0.2850794	0.4911949	0.08909195	0.6274134	0.2487068	0.6017684	0.5048648	0.8612599	0.3974798	0.3391174	0.2969829	0.5034802
8.914943	6.000953	4.322351	3.69803	2.984894	0.6940168	3.70325	3.655045	3.257567	3.868612	0.8679052	7.601277	4.640533	4.749377	3.84915	2.07214	1.155421	7.270449
0.5959772	0.1861795	0.5464137	0.3149527	0.3073137	0.02625662	0.4522554	0.4372941	0.1264807	0.4718081	0.03302621	0.6528168	0.4955736	0.1811408	0.1787224	0.1168635	0.08030783	0.6280341
3.197603	3.229616	2.572257	2.02428	1.813233	0.5725883	1.902923	1.776359	1.741297	1.801424	0.9930244	3.174624	2.853132	1.699359	1.919209	1.656314	1.625263	2.035213
1.249633	1.114863	1.261305	0.6894702	0.795783	0.5726681	1.061415	0.8574288	3.213821	1.492135	0.6614222	1.086859	1.581029	2.236278	0.6788993	0.8437086	2.418936	1.085146
5.24321	4.837881	2.238181	2.965054	1.683951	0.1883365	0.7397611	2.707812	1.482868	1.366908	0.5826317	4.128404	1.659446	2.70271	2.11217	2.074763	0.7005907	4.952041
1.937164	1.796989	0.6978994	0.8986438	1.080734	0.6325532	2.730936	1.639628	1.506406	1.876863	1.65206	0.6341587	2.135546	1.060669	2.242519	2.438762	3.37007	0.7880255
0.4961058	0.6143978	0.4334449	0.3217457	0.301748	0.1347492	0.4759819	0.4166616	0.3235841	0.496035	0.1906772	0.4728192	0.4937254	0.3683189	0.2976507	0.2860624	0.3756489	0.5762412
7.732228	4.779014	4.147341	2.986156	3.814957	0.8987289	5.862349	5.76372	3.150288	5.793881	1.3302	9.628866	6.833022	5.398058	3.524562	2.058804	1.890511	6.495684
4.976315	2.829504	2.062126	2.136579	1.526411	1.088086	1.975498	3.289794	1.516791	2.371948	1.328276	2.93861	2.247292	4.936284	1.601929	1.658797	3.323591	3.489883
2.551744	1.706301	1.764904	0.9465166	1.041462	0.3757483	1.411946	1.260619	0.8741095	1.097001	0.4235794	2.949648	2.057845	1.148514	0.9001811	0.6103306	0.5240311	1.875125
1.695562	1.139216	0.6657432	0.7653975	0.4881211	0.240415	0.9543742	1.248313	0.572158	1.014011	0.2267999	0.7230757	1.039878	0.6886112	0.5271248	0.5983802	0.1455336	1.16116
1.638357	1.55769	2.072798	0.661779	1.801569	0.2123561	0.7488852	1.169338	0.8230551	0.7017655	0.626319	1.225982	1.172682	0.7036403	0.8243318	0.3602239	0.6812357	1.035745
0.07827841	0.03526972	0.05787783	0.1190019	0.02471265	0.05690298	0.09049419	0.03040741	0.02966529	0.05164122	0.1041076	0.02593064	0.03698348	0.1019652	0.04062848	0.08482561	0.145035	0.01189744
0.06671134	0.08416233	0.1640067	0.02535431	0.01842829	0.01543008	0.1038181	0.01511659	0.04424297	0.1001234	0.01940833	0.386731	0.1103147	0.09504462	0.02019785	0.01807277	0.03932837	0.1064634
0	0	0	0	0.02469591	0.02067797	0	0	0.02371615	0.06192748	0	0	0	0.05094804	0	0.04843894	0	0
0.21773	0.1272935	0.1539185	0.08073216	0.1408287	0.004913183	0.01983441	0.1925346	0.1014312	0.1765711	0.01235984	0.0935874	0.2107558	0.1755296	0.1800769	0.04603726	0.03130695	0.1242987
38.80464	22.42002	20.17914	21.26623	10.41738	5.121844	11.96618	18.04977	9.131749	26.98692	4.529004	30.73606	24.11103	18.50078	7.29758	8.785349	12.20906	36.13027
4.92563	6.377653	10.3383	4.611117	4.246668	7.015561	7.729957	5.892009	4.800298	7.679932	4.608269	3.853324	6.035937	7.468221	4.921314	6.952938	8.573879	7.378048
12.73054	8.361005	9.136089	5.506113	5.22876	1.733263	4.429106	5.56925	4.019631	6.676852	1.443072	11.61217	9.926177	6.508164	4.984366	2.719094	1.937063	13.27579
3.291628	2.255403	3.313511	2.277079	1.588315	0.4246746	1.789595	2.496281	0.7370156	2.047212	0.365482	6.268644	1.965501	1.810456	1.338538	0.7853812	0.6907522	3.938985
87.85331	61.33133	41.73187	49.74669	35.24759	11.7092	36.53397	35.98888	40.90801	58.21797	22.01672	67.99189	56.45476	53.50265	36.62206	29.67753	25.80819	65.76632
0.7171469	2.80969	1.249729	0.5001079	1.312213	0.3713127	1.417069	1.130069	2.907778	1.449281	0.7771343	1.986378	0.8616694	5.661694	0.9920107	0.645231	1.020102	0.7874875
2.084075	0.5414505	1.005236	0.6165178	0.3576795	0.1547907	0.4075347	0.840646	0.5518986	0.987617	0.2581885	1.663163	0.8083358	0.9741916	0.4052396	0.268011	0.3216298	1.168625
1.390627	1.072134	1.826021	0.9591687	1.067071	0.3335595	1.362602	0.7352612	0.8061269	1.213024	1.318615	1.289852	1.158296	1.174072	0.8264719	1.604611	2.034361	0.6712626
0.6066521	2.461518	0.2545819	0.7477756	0.2717532	0.1668626	0.877748	1.248336	0.9221009	0.1968629	0.4006874	0.1216626	0.9326749	0.6914449	1.628235	0.1599063	0.1159913	0.5407658
0.004418532	0.005574374	0.02286895	0.2216685	0.02441144	0.01635183	0.01100199	0.128157	0	0.146914	0.0154258	0.012295	0.06429756	0.04532505	0	0	0.01562916	0.009401941
242.5447	44.77216	55.78383	44.56762	27.39533	16.94137	33.38578	48.06292	34.95034	195.759	36.72512	110.4641	220.8448	165.7322	88.20506	17.49694	37.19407	39.49833
0.09124778	0.1083457	0.05000509	0.07751916	0.01186175	0.004965944	0.06682467	0.1994672	0.0569558	0.1536801	0.006246285	0.05476403	0.09230827	0.1101194	0.1105065	0.01744937	0.01265726	0.1085017
1.799029	0.730058	1.870393	0.5535741	0.2740362	0.1056205	0.4410907	0.6815059	0.9691132	0.8871444	0.06871659	0.7120096	1.791448	0.7358404	0.8915155	0.4137887	0.148528	0.280612
1.359128	1.261991	0.7428355	0.5950657	0.528626	0.1609529	0.500859	0.6011661	0.3922779	0.5623685	0.3416351	1.099275	0.6904234	0.8551008	0.7374011	0.3299084	0.2051194	1.665798
4.931119	3.098904	2.676149	2.361906	1.214586	1.284005	2.117742	2.014897	1.21853	2.915249	0.9004278	3.78205	2.672697	2.141752	2.22365	1.617042	1.122272	3.599905
51.75634	38.45217	27.52164	23.91425	20.67709	6.763681	19.65223	20.15902	20.24271	31.39211	6.983787	48.24185	42.91878	26.57263	26.3626	19.52933	13.18682	41.55971
0	0	0.2206975	0	0.08566666	0.01793225	0.04826139	0.03513587	0	0.01790148	0.04511124	0.05393329	0	0	0	0.02100349	0	0.04124259
0.4621475	0.4688367	0.2811137	0.2353988	0.1631984	0.09697504	0.2431959	0.1252337	0.4954497	0.3124279	0.07207765	0.2828252	0.2962311	0.3366809	0.3692787	0.1290726	0.1123505	0.1926203
0.1333704	0.05150777	0.06198468	0.07655034	0.09022565	0.03021851	0.0135546	0.2615071	0.03754666	0.3092082	0.0443445	0.0227214	0.05761123	0.1520118	0.03296315	0.008848499	0.1540426	0.0405416
11.29737	6.479301	7.838449	5.052834	6.199799	4.704968	5.28279	6.406811	8.000824	6.751163	2.760069	11.34996	8.198664	5.894687	4.790151	5.050576	4.769674	10.93588
1.699341	1.28566	0.9107925	0.7706296	0.7690731	0.8043271	0.922123	1.045093	1.270884	3.859482	0.7641256	1.042676	1.761603	1.095659	1.075124	0.9733912	2.588922	1.022744
0.04204685	0.01515596	0.0435243	0.1095794	0.0530971	0.0277865	0.05234761	0.1306655	0.01912147	0.1608852	0.01398021	0.05571405	0.07151577	0.2875428	0.05092122	0.01952728	0.08498709	0.01917195
22.82521	26.03301	14.50578	13.05794	9.717789	3.894237	14.40022	15.80329	9.368568	14.66525	4.758885	31.6456	17.29323	21.17906	14.40155	9.029691	5.628588	23.81092
8.727854	15.08556	5.394534	2.737014	6.364045	1.224885	11.42245	3.02675	8.617038	4.704209	1.462183	2.651527	14.16755	4.997909	8.129158	7.191622	2.813778	6.046952
0.4817821	0.9254614	0.4185163	0.3735732	0.1845832	0.1276735	0.1687909	0.4520423	0.5292116	0.366711	0.08170412	0.2268027	0.5092354	0.4884147	0.1817836	0.08919931	0.2854525	0.2627286
2.08681	7.60958	1.213228	3.063807	1.042364	0.4231638	2.847171	5.311636	2.062689	0.7656684	2.295396	0.4507527	2.306821	2.508815	3.912046	1.115187	0.5055775	2.28103
1.281365	9.741201	6.462845	1.810226	7.629247	1.976619	8.945628	5.802442	4.55033	8.53179	3.341719	10.88597	2.421096	5.984407	4.17319	3.382832	4.932695	2.199701
2.723842	1.353212	2.925517	1.133333	2.085172	0.9182561	0.5541074	2.206643	0.6517398	2.075891	1.854223	2.518197	3.29129	1.562425	0.6144701	0.8681366	1.810448	1.73309
0.4690862	0.3440664	0.225846	0.9287189	0.5665359	0.4238985	0.2308854	1.146982	0.1389089	2.216611	0.2285099	0.3440269	0.8803145	1.964532	0.4227655	0.2009639	0.836053	0.1392756
0.008363229	0	0.006492823	0.01907117	0.03465379	0.02321259	0.005206039	0.03979675	0.006655794	0.02896595	0.04136293	0.04072514	0.01659545	0.02859651	0.01012837	0.002265682	0.1158639	0.03781573
125.2337	95.95584	47.51293	63.94699	51.26371	22.17608	54.92979	57.61749	39.46261	96.27761	26.97341	99.81645	74.90804	75.87525	54.52535	41.07665	25.14385	119.4365
0.2290886	0.232346	0.3440831	0.1809636	0.2134259	0.04155867	0.1286248	0.2809291	0.05719777	0.692839	0.01568205	0.8999457	0.4040788	0.5375767	0.1414399	0.03407347	0.100629	0.372767
1.932231	1.854913	1.959307	1.198961	1.923396	0.4065256	1.92167	1.633914	0.98631	2.091575	1.009564	3.03578	1.788545	2.292192	1.951175	0.9400925	3.692972	2.774952
0.08051275	0.05417283	0.04444897	0.04895947	0.02965438	0.004965944	0.03341233	0.03892043	0.06265138	0.04957423	0.06870914	0.009957096	0.03550318	0.04894195	0.0650038	0.04071519	0.0632863	0.02284246
0.09912487	0.4251867	0.1026079	0.3013871	0.6681253	0.458544	0.2591584	0.2605523	0.3260686	0.3204301	0.484485	0.1287182	0.4720728	0.1807675	0.276106	0.2900223	1.110306	0.2109221
0.01586761	0.0100092	0.06570067	0.572911	0.02629954	0.0440414	0.009877451	0.09348439	0.06734976	0.007327639	0.009232724	0.2796368	0.1154511	0.9314007	0.009608306	0	0.3554687	0.05064566
0.01638786	0	0.04240924	0.006228368	0	0	0.0102013	0.007426881	0.01738949	0.01513578	0	0	0	0.009339208	0	0.008879268	0.009661143	0
0.01150938	0.01452011	0.007446123	0.02515196	0.3020375	0.04791735	0.01074674	0.02086393	0.01373944	0.005315015	0.008371057	0.01734741	0.009516029	0.02951564	0.03833098	0.01559003	0.003392565	0.003061269
0.01038689	0.006551995	0.005375932	0.00789528	0.005738533	0.004804895	0.03232875	0.01882911	0.01102174	0.0671531	0.01208743	0	0.06870357	0.3433217	0.006289568	0.005627823	0.006123388	0.01105083
0.2349303	0.09082784	0.05883515	0.07200611	0.05861662	0	0.1132197	0.106469	0.06031192	0.2939742	0.004409564	0.2917123	0.2155458	0.1295646	0.05506732	0.004106126	0.008935392	0.2620416
0.06680591	0.01685633	0.04840734	0.0304683	0.03690883	0.03708468	0.03326889	0.1211042	0	0.1480842	0.04664601	0.08675054	0.08837689	0.07614348	0.008090591	0.01447871	0.0945219	0.03553812
0.009990538	0.01260396	0	0.003797003	0.01655867	0	0.006219025	0.004527654	0.01060116	0.01845446	0.005813093	0	0	0	0.0181487	0	0	0
0.04406895	0.07148177	0.06516779	0.04306845	0.07651962	0.02135672	0.3265779	0.1635785	0.02894819	0.1918806	0.07570482	0.03892895	0.122149	0.1650366	0.04828724	0.02274042	0.08412569	0.03125727
0.4052087	1.443408	0.4687937	0.6363972	0.4411539	0.2508474	0.7975203	0.9505979	0.8757583	1.015427	0.2323074	0.7903786	0.5478713	0.5433475	0.2922741	0.2970385	0.2986037	0.7322534
0.4362644	0.2563444	0.3464282	0.4224661	0.2707423	0.2875138	0.2232089	0.4983437	0.3043916	0.9935322	0.3338232	0.3547612	0.5534123	0.9127451	0.3835907	0.3108516	0.4509653	0.2543293
2.608666	3.805295	1.406422	2.127485	2.70231	1.759842	5.176094	5.024487	1.153378	2.735624	0.8854289	3.629435	2.767973	2.818425	1.579625	1.648999	2.178675	3.180164
4.784544	0.8743927	1.695246	1.372307	0.7596565	0.3102747	0.8420067	1.079097	1.364141	1.321567	0.2471717	1.887111	2.225651	1.554442	0.8258325	0.7147164	0.4217771	1.635343
0.07179272	0.1207639	0.4706641	0.06821384	0.211541	0.02767564	0.1489679	0.04880407	0.2221937	0.1989227	0.1392444	0.06659011	0.1662043	0.1704736	0.2028722	0.1880105	0.08464801	0.05728636
6.006009	1.631651	4.526158	2.574876	1.487803	0.754002	2.289534	1.817804	1.387418	5.484952	0.6844989	6.44829	5.362486	5.125022	2.282934	0.9599338	1.583404	3.162917
2.110277	1.861214	1.133541	0.539023	0.4180372	0.1882039	2.374	0.6341303	1.589671	0.7058705	0.2212406	0.4390814	1.584458	0.6955675	1.114364	1.040382	0.1748426	0.7463668
118.0157	98.85965	28.32507	64.86559	41.07301	15.46051	49.04852	50.14564	38.13166	79.6915	22.20414	88.03898	50.29153	71.19509	54.47456	34.91062	19.03487	91.50327
0.1057665	0.07656047	0.2351192	0.07512343	0.07088684	0.05133302	0.0626008	0.1100097	0.05887529	0.08167163	0.04842594	0.1109678	0.1399175	0.09881123	0.06299486	0.03945688	0.1226609	0.03504948
0.2837743	0.04475083	0.2019501	0.3639979	0.1567792	0.09845384	0.08832353	0.3697389	0.1317394	0.3439972	0.1238376	0.1974075	0.2815517	0.8288095	0.1933129	0.05765796	0.5018808	0.2641743

TCGA-QH-A6X3-01	TCGA-TM-A7C4-01	TCGA-FG-A70Y-01	TCGA-27-2528-01	TCGA-FG-8191-01	TCGA-DU-7306-01	TCGA-TQ-A7RM-01	TCGA-HT-7602-01	TCGA-HT-7695-01	TCGA-DU-8164-01	TCGA-WY-A85E-01	TCGA-DB-5278-01	TCGA-QH-A6CY-01	TCGA-FG-7636-01	TCGA-P5-A5F4-01	TCGA-HT-A5R5-01	TCGA-FG-5963-02	TCGA-DU-6393-01
0.03586677	0.04053225	0.1995945	0.4110711	0.1257172	0.1872316	0.068023	0.035279	0.04318348	0.05261715	0.02770371	0.3021171	0.02538365	0.01095601	0.01901633	0.04770603	0.1965649	0.02667121
0.03680952	0	0.2803084	0.5523843	0.3964482	0.3019546	0.07778934	0	0.09218257	0.05200016	0	0.141928	0.2709289	0.07308591	0.2537102	0.08486393	0.4973725	0.09489046
0.4887711	0.305871	0.9580262	1.354849	0.6901362	0.5428267	0.2906237	0.5645069	0.2994282	0.3136851	0.3094415	0.3911001	0.3384744	0.3156614	0.395449	1.102623	1.139453	0.2895951
0.08809115	0.06968491	0.02096323	0.4910082	0.7334682	0.8671517	0.04964334	0.03003782	0.01378799	0.0155556	0.08845476	0.05094852	0.01620942	0.0568446	0.02023899	0.710826	2.732264	0.00851581
5.659648	10.61319	27.45433	45.64491	11.17968	16.51394	2.881315	23.19623	3.380914	2.679643	7.264506	9.853319	9.982139	8.440262	7.949477	15.91146	15.49811	5.765871
2.437466	1.050871	3.346803	3.49853	2.21442	1.592374	0.7232686	1.753366	1.077639	1.327818	1.370067	2.016116	0.9323513	1.350787	0.9627555	2.792455	4.230716	0.6057544
3.103545	1.810422	2.523653	2.747543	2.097583	4.202546	1.155076	2.474282	1.412064	1.136019	1.846645	1.685965	1.393007	1.646564	1.50114	10.50104	6.374412	0.7126008
0.841519	1.576945	2.930358	5.02392	1.006062	1.668188	0.7578739	3.300786	1.123739	0.7411185	2.688613	2.435564	0.9996366	1.242563	1.627478	1.430526	4.952582	0.7208229
0.3141776	0.01224294	0.02062496	0.1393515	0.07180425	0.08887073	0.03052642	0.04617674	0.02034825	0.01530459	0.02966844	0.0334176	0.03986964	0	0.08711678	0.2622585	0.2129247	0.05585597
1.807274	1.009166	4.400878	1.633335	3.260772	1.617313	0.5624941	2.438837	0.8933634	0.635773	1.000637	1.300765	0.9715725	2.035756	0.8358721	4.335494	1.581568	0.5937855
0.04065529	0.005742956	0.004837412	0.02723643	0.04210271	0.02652858	0.003579858	0.03032503	0.01590837	0.02153738	0.01391697	0.01567563	0.03272881	0.01009022	0.05254073	0.1405955	0.03121231	0.01637569
7.157117	25.14142	76.08254	33.51151	32.36589	44.70514	14.27836	68.49293	20.68733	15.30842	18.66157	26.36363	20.17362	34.74909	19.38662	103.4062	20.07214	6.986692
0.6797224	0.4147231	1.303153	0.4412603	0.8116584	0.5068978	0.1955735	0.8814	0.3356525	0.2299147	0.279653	0.5364713	0.2554326	0.5924301	0.3592565	0.8534399	0.1920777	0.1131142
0.2256096	0.3149462	0.2463368	0.07823922	0.3545858	0.1459787	0.1916467	0.2856578	0.1661756	0.07967869	0.6269246	0.2277072	0.1648343	0.2404446	0.2782262	0.2906667	0.1752452	0.2779678
0.9650431	0.5433465	1.366478	0.5485041	0.9104862	0.9783464	0.3435322	1.566283	0.4644328	0.4778826	0.485924	0.6620922	0.4644756	1.039881	0.674626	1.904226	0.6876334	0.1748517
0.6441665	0.04632463	0.1449322	0.3075776	5.496907	0.07860802	0.09075424	0.1098258	0.06232799	0.01654551	0.01603699	0.04515892	0.09159242	0.02325461	0.2152693	1.302854	0.1294813	0.03774052
0.2874119	0.1029847	0.01334557	0.07514051	0.6446543	0.06273228	0.03456669	0.05975814	0.004388839	0.02970892	0.01919723	0.02162315	0.03224751	0.0417557	0.02415843	0.2666662	0.111942	0.04517763
2.360717	0.7439042	6.300632	1.513401	8.045153	1.641993	0.7035614	1.644772	0.633832	0.4104546	2.121812	1.515886	0.7225898	1.825321	0.5684518	10.17872	6.864795	0.7987404
330.0432	58.76856	186.4077	174.1036	277.3593	204.6394	46.69431	198.5321	32.30737	34.40611	111.6488	49.05712	106.3192	267.4113	58.89357	455.7624	165.8131	70.43396
0.1517965	0.1048312	0.6181104	0.5423676	0.4331756	0.4622379	0.3623747	0.6470054	0.1108759	0.07445843	0.2232459	0.2243605	0.155176	0.3097666	0.1162511	0.7777004	0.5956431	0.1250028
2.678553	3.228772	27.84506	12.1013	5.194414	30.78139	4.607658	12.8207	5.573384	4.582778	2.143235	7.461525	5.360759	5.003153	3.297323	11.6341	17.55614	1.999036
0.1040896	0.01604037	0.09908171	0.07607277	0.1058355	0.07762406	0.02666328	0.08873268	0.0503573	0.05681308	0.03023285	0.09851143	0.04788314	0.02818251	0.03804604	0.1090807	0.07555394	0.02134447
2.62689	1.160071	1.688719	2.158371	4.413721	1.485923	1.064295	1.884807	1.254079	1.050448	2.2754	1.453324	1.181779	1.661928	0.9887483	5.968275	2.392611	0.6547887
5.429957	3.799869	18.71226	8.480917	7.806707	6.548425	3.688996	7.805121	2.880396	2.459312	5.13817	3.793102	4.196229	6.332305	5.50038	17.0037	5.895548	2.781984
1.98955	0.9614881	4.372744	2.857328	5.880298	1.640505	1.622551	2.007646	0.958009	0.8500359	1.045392	1.983284	1.166732	1.43238	1.28211	9.468375	3.266484	1.582428
0.9426335	0.08437619	0.2132155	0.3201287	0.8892059	0.5776815	0.2235317	0.4773631	0.0818047	0.2043609	0.119274	0.3958423	0.2747746	0.3520859	0.171541	0.2689642	0.4929682	0.2045045
15.89947	3.945569	13.1863	9.146385	15.24211	9.657239	4.070832	11.84749	2.656997	2.705301	5.955231	4.003782	5.482941	14.97009	4.659063	22.74306	9.69589	4.267449
7.817301	2.889411	14.32816	5.199101	17.74324	7.887454	2.624981	8.299748	2.906749	2.410372	3.623447	5.419224	3.037122	9.077596	5.118218	29.10012	5.684401	3.971025
0.0235763	0.09991145	0.044884	0.08213194	0.2343904	0.09670015	0.0290638	0.02512243	0.9262282	0.1373865	0.07532513	0.263622	0.3145203	0.02340556	0.2302083	0.02038308	0.3547649	0.04178407
1.398158	0.1288844	0.2452698	0.4301353	0.2729643	0.459907	0.1071198	0.4321014	0.1269399	0.2148203	0.2390651	0.2964202	0.1787686	0.2893483	0.1989476	0.6135244	0.477359	0.1306688
0.1318356	0.1048409	0.05577455	0.06804016	0.02831717	0.1492937	0.02407718	0.04162411	0.05808328	0.05863168	0.04011507	0.09789944	0.06289296	0.009694879	0.07291867	0.01688586	0.0599788	0.03461497
0.9441019	0.1096822	0.2183707	0.1607819	0.2485404	0.3092586	0.3418508	0.6694239	0.2485858	0.2991513	0.330229	0.5511352	0.1542389	0.2540251	0.2736689	0.2135932	0.4226965	0.1876513
2.070138	2.481029	10.83559	3.008781	9.14013	4.276261	1.57126	8.699679	3.157011	1.685275	2.937519	3.857607	2.79926	5.74247	1.577708	15.49099	2.764551	1.05633
0.2821387	0.1705417	0.6308145	0.2953906	0.7175483	0.4599504	0.1710152	0.3803464	0.3532819	0.254901	0.155727	0.5059788	0.2293945	0.3908311	0.1733599	1.021083	0.4594075	0.1014864
6.536441	1.102491	5.282993	1.882315	3.861673	2.449376	1.015581	4.435487	1.153723	0.5972184	1.642587	1.956041	1.396231	2.808718	2.266324	3.711193	1.651105	0.8453019
1.548067	1.561271	3.49977	2.267161	1.774685	1.372599	0.5801245	1.634245	0.9354018	0.6961296	0.8585665	1.148676	1.018525	0.8667157	2.765743	2.988043	5.500263	0.7308603
0.8649622	1.574022	8.518014	2.365598	3.56722	2.210207	0.824356	4.548794	1.732113	1.105116	1.852018	1.392883	1.732029	3.289573	1.402769	7.727373	1.164055	0.4590698
1.717926	1.736707	2.215195	1.570813	4.506265	1.592203	0.7075382	1.572083	0.999958	1.294855	1.518095	1.743795	1.15638	1.144255	1.841066	5.846084	1.294465	1.457336
0.4366965	0.3091815	0.5158518	0.2763446	0.4925645	0.3335105	0.2668535	0.4619713	0.3491695	0.2489953	0.35541	0.4463057	0.3436886	0.5484468	0.3565991	0.3881635	0.6398318	0.2475295
9.231509	1.742968	8.27223	4.708331	9.590996	7.012128	1.909273	5.91272	3.269581	3.239593	2.988738	5.633885	3.120167	4.401006	1.984176	9.009205	6.86919	1.755473
2.083996	1.930049	4.199289	4.279304	3.629336	2.972607	1.107539	3.716	3.352576	1.992443	1.770097	2.852788	1.928698	2.925847	2.882711	6.083968	3.472549	3.45702
1.085133	1.061111	3.060831	0.9495107	1.57052	1.317888	0.5397611	2.945014	1.098097	0.7383177	0.986513	1.823879	1.11246	1.486199	1.073559	4.125353	0.8106473	0.3082783
1.088918	0.4243318	1.179498	0.9659654	2.376695	1.08087	0.3861791	0.979478	0.2585941	0.2705278	0.5141439	0.9960787	0.5112862	1.006478	0.3882096	4.821869	0.7103082	0.3533072
0.4849177	0.5583343	1.654748	1.000335	1.114275	0.6754858	0.2449145	1.606702	0.3952482	0.5234699	1.052347	0.4938877	0.5471554	1.407886	0.7567479	2.594539	0.6855686	1.82202
0.07756202	0.02191278	0.04306772	0.1247078	0.08567829	0.1060424	0.04097784	0.03305942	0.04046659	0.03195806	0.08850247	0.09470208	0.02973329	0.08341704	0.1187994	0.01490153	0.1429122	0.0416553
0.2988307	0.04357446	0.2752776	0.2583192	0.5510559	0.244417	0.03395255	0.1890025	0.04224646	0.006808919	0.03519815	0.05946913	0.03547552	0.08612905	0.1550309	0.2666907	0.1894578	0.04348746
0.02583649	0	0.02459345	0.08308219	0	0	0	0	0	0	0	0	0	0.02564939	0	0	0.03173675	0
0.06752757	0.04856181	0.1519315	0.1908269	0.2339533	0.1419185	0.0648662	0.04186533	0.06149462	0.01734455	0.06164209	0.1893592	0.02259194	0.2864375	0	0.03538274	0.2714688	0.01582526
29.22781	9.962609	36.39617	22.19378	21.04862	20.37901	6.879614	24.88118	8.836134	8.640684	9.893256	23.52911	6.594769	16.8115	15.00504	49.26216	22.25436	6.365686
4.555181	4.089382	5.612584	11.37955	5.238748	3.112047	6.839838	5.077446	3.865076	4.297073	7.570873	5.361522	4.356366	5.786624	4.286058	7.038704	11.43937	7.995854
5.369261	4.523817	16.86289	5.872567	8.861809	7.53538	2.790269	10.96209	3.579361	2.054783	3.518484	6.543891	4.710214	8.285202	3.606029	18.23508	4.507225	1.988693
3.323343	2.303878	4.751823	2.305006	3.863915	3.576957	0.7524859	4.850672	1.787805	1.740834	1.383006	2.702775	1.496681	2.793295	1.475749	10.5593	1.389351	0.4589557
97.79501	21.51369	148.3532	58.22964	81.80378	117.1529	22.31213	61.63056	19.86792	16.11506	23.39888	33.30305	25.88643	73.03834	26.29471	146.4972	42.29536	25.51371
0.5247889	1.0228	0.8072284	5.584435	0.986127	1.758272	0.779538	0.8461009	1.145195	0.6124279	1.398965	0.9824001	0.4513364	0.5323144	0.991649	4.212714	2.036671	0.4656521
0.6516948	0.5701672	1.480813	0.7841786	0.5399173	0.84971	0.515348	1.175608	0.6528193	0.6860237	0.4029936	0.7457246	0.5067445	0.4591439	0.3525835	1.20198	0.723052	0.3034821
3.185331	0.8915075	1.756906	1.372121	1.319494	0.8436129	0.6710571	1.433822	0.5311832	1.240616	1.508204	0.7575699	0.8490584	1.093493	1.214019	1.372662	1.462715	0.9113114
0.1326753	0.02141905	6.007892	3.575672	2.009948	1.272106	0.7877395	0.9855935	0.3678598	0.281142	0.1211117	0.7892665	1.447355	0.09408176	5.748074	4.0857	4.330457	0.2320855
0.06129332	0.0404053	0.0291722	0.1478253	0.0126951	0.09141806	0.007196157	0.01306259	0.009593605	0.01803917	0.0139878	0.04726625	0.04229408	0	0.02347029	0.07065553	0.1317589	0.03291807
327.0494	38.53311	114.8978	143.4667	208.8157	149.7916	38.12273	127.0876	26.33742	30.85023	60.75602	36.55259	63.97583	151.8924	27.32822	294.2712	191.6921	57.89908
0.09307189	0.0280476	0.04134389	0.07315991	0.1490762	0.2544948	0.04370852	0.04760427	0.06992436	0.06135782	0.02832002	0.02392409	0.03996046	0.01847958	0.08553328	0.00715254	0.7088253	0.03998801
5.196871	0.8948155	1.425141	0.7511899	1.436428	0.5769174	0.2532455	1.454733	0.3447386	0.3375046	1.242062	0.6246459	0.632205	0.6189278	0.5645809	2.082569	1.570765	0.2404873
1.269479	0.8948562	1.112688	0.5793299	1.478685	0.8435982	0.4161414	0.8143179	0.4957623	0.2752204	0.3786295	0.7172558	0.5435097	0.9358562	0.5053577	3.317972	0.4786245	0.259213
6.00557	1.203327	5.878806	2.19144	4.969624	2.705164	0.8401018	4.121042	1.151098	1.073031	1.425617	1.434245	1.652379	4.312994	1.948984	7.274759	2.825256	1.189487
34.16142	15.13319	83.22571	30.26298	54.11365	36.85735	12.73561	53.63711	15.16225	9.948829	23.27768	21.33421	20.36649	44.80827	23.30432	91.14208	18.82518	9.822523
0.04481158	0	0.04265563	0.04803342	0.01856279	0.01670895	0	0	0	0.04747841	0.02045298	0	0	0.04448708	0	0.0516563	0	0.1877181
0.4846692	0.1182564	0.5609609	0.2479507	0.2509623	0.3984037	0.1047527	0.4741986	0.1275831	0.06613423	0.276517	0.2675725	0.1722848	0.2897892	0.3036901	0.685677	0.2503188	0.09938556
0.09124629	0.08889261	0.03294549	0.1180425	0.3519119	0.1243604	0.1086056	0.03486877	0.08470604	0.02889184	0.07180481	0.1941088	0.08973959	0.03123639	0.07590373	0.1196917	0.3478473	0.05474992
9.623114	4.236168	16.6807	8.75414	10.0855	8.978988	2.895581	9.927022	4.321307	3.708634	3.778847	6.56102	4.654561	6.187217	4.153234	12.15289	4.70172	3.871771
1.193226	1.084241	2.213834	6.115193	1.650126	1.372119	0.8255745	1.428714	0.8173864	0.6069197	1.24998	1.266676	1.265417	1.308168	1.335843	2.530472	5.396693	0.7924789
0.0277747	0.02354068	0.04626722	0.1042007	0.1035487	0.09838566	0.07826148	0.02367693	0.01304185	0.01471381	0.01901546	0.06425525	0.04471905	0.01378678	0.01595313	0.02401284	0.05117637	0.05817483
15.32902	9.097366	25.361	21.87992	25.67821	19.78019	7.259209	25.91569	10.17919	6.189152	10.00274	16.19852	11.70116	20.9125	8.38428	42.29507	13.02287	4.563503
11.96095	3.403987	14.73525	12.3507	16.51571	7.78573	1.229172	3.880735	4.412534	2.547636	5.55267	5.149317	6.008983	6.280718	5.677479	20.60883	4.226926	3.398762
0.1007521	0.6894716	0.3916109	0.3689859	0.825438	0.3694142	0.1439173	0.830246	0.5694604	0.2965223	0.4649638	0.4143723	0.5510239	0.4362091	0.8712591	1.232389	2.805244	0.09559382
0.7600495	0.1120324	14.34382	10.41393	7.966912	6.111596	2.374395	1.831064	2.296496	0.8636352	0.1809929	3.771497	3.496367	0.06561265	13.55217	12.11363	15.10031	1.213924
2.118592	4.41155	3.258119	20.24845	4.561603	11.98366	1.234662	3.267444	3.707652	3.755712	6.087341	6.020753	2.70388	1.757591	3.260803	22.12602	8.948968	4.577216
2.160895	1.354718	1.317415	4.632516	3.716926	12.63854	0.6342511	2.508745	0.6667814	0.9339683	1.235198	1.019661	0.44969	0.6333586	1.595787	16.48778	10.78815	0.8687367
0.5548694	0.3277739	0.3241066	1.662633	1.211934	0.7523448	0.5596511	0.6557586	0.4105538	0.1068892	0.5410435	0.9724698	0.6497275	0.2253482	2.071575	1.16295	2.277112	0.3494693
0.01450171	0.0628209	0.004601336	0.05181447	0.1221464	0.01802423	0.0289438	0.02266401	0.07414685	0.05292297	0.01765038	0.02795743	0.008894736	0.01199724	0.04720015	0.05572254	0.07422272	0.01090356
116.9463	35.65437	158.0203	55.37456	114.0219	133.3846	30.81728	69.08345	26.60261	25.88689	44.39377	52.59204	30.23642	98.13434	36.50608	173.7142	48.51016	36.44153
0.1453936	0.2112507	0.3163393	0.3784855	1.088405	0.3717468	0.1280248	0.4824916	0.2243184	0.1210363	0.2133025	0.1561672	0.07166112	0.3711618	0.1849164	1.664045	0.3508155	0.07027631
1.41963	0.9714068	3.334933	2.422152	3.560785	2.287329	0.8991116	3.386294	2.018147	0.8739508	1.040287	3.550392	1.366025	1.719666	0.8453252	3.775894	1.23188	1.397544
0.8748758	0.0070119	0.1653756	0.2527342	0.4369474	0.1203066	0.03059596	0.005289363	0.01553875	0.02191351	0.02832002	0.08612671	0.09704684	0.0307993	0.02851109	0.07867794	0.2134098	0.219934
0.171881	0.1553911	0.4472048	0.3193472	0.2848005	0.3503558	0.1775816	0.09768157	0.2439182	0.2023444	0.1464404	0.3534554	0.3584434	0.1592605	0.5396932	0.1849258	0.3518888	1.129875
0	0.7980581	0.1309522	2.300406	0.433105	0.2257034	0	0.03127314	0.1550343	0.2720816	0.04186023	0.488003	0.3459588	0.1365747	0.126428	0.06343364	0.07886101	0
0.08524886	0.02140841	0.02704913	0.03045934	0.0392373	0.02119123	0.02668977	0	0	0.03345266	0.06052578	0.007304393	0.008714676	0.03761401	0.0108811	0	0.01163523	0.006104478
0.03991416	0.007517687	0.04432613	0.04991453	0.02204544	0.01984379	0.002343066	0.02410132	0.0124947	0.007048256	0.003036282	0.05001709	0.009180624	0.02311465	0.01337336	0.001917118	0.03472911	0.07502678
0.1680999	0	0.01714418	0.2702787	0.01989538	0.08506518	0	0.01023565	0.007517407	0	0	0.01851857	0.011047	0	0	0.006920578	0.567844	0.01934558
0.4029851	0.03465035	0.4252922	0.09390406	0.1923358	0.1731275	0.03702723	0.1344249	0.07130225	0.08353713	0.04398354	0.1182244	0.06448019	0.1174109	0.03019111	0.09593737	0.3820221	0.1016263
4.270649	0.06109072	0.1764274	0.1821145	0.2367301	0.2188473	0.01632032	0.05266655	0.06769016	0.02727428	0.05639687	0.05359809	0.02842064	0.007666768	0.0266144	0.08012062	0.08537687	0.1294031
0	0	0.005496659	0.006189648	0.04305646	0.03445019	0.008135449	0	0	0.004078753	0.005271195	0.01781192	0.005312733	0.005732662	0.006633449	0.06656498	0.04965235	0
0.05822085	0.06853553	0.03001903	0.06500706	0.03617619	0.09407182	0.04784803	0.05997102	0.04556352	0.01713491	0.03100214	0.03180199	0.06918854	0.01926641	0.04180085	0.1174491	0.1370737	0.02814118
0.6509619	0.6616847	2.134326	1.620733	1.138544	0.8116518	0.2256397	1.101035	0.3838332	0.3722201	0.6193787	0.8844557	0.465818	0.533411	0.731968	2.481457	1.066162	0.2397287
0.3592395	0.2420198	0.348532	0.340637	0.6810997	0.4739772	0.3065915	0.453469	0.1686841	0.2000687	0.3846864	0.3036643	0.3813618	0.226328	0.301572	0.6052397	0.6704043	0.262685
2.952766	1.703926	5.113088	10.00025	5.387093	16.46817	1.482568	4.257694	1.376664	1.309085	1.978548	2.47081	2.54325	3.056123	1.587744	2.896838	4.167291	3.664226
2.326072	0.8616107	3.622613	2.957343	18.0827	2.279138	0.5461861	1.057543	1.072001	0.502028	1.038077	1.34531	0.6717447	1.969258	0.6160633	19.63356	2.762033	0.4747095
0.08299164	0.0547091	0.05266585	0.08895851	0.257839	0.07220545	0.112052	0.0353737	0.02597964	0.01954014	0.1767695	0.01066648	0.08908125	0.08239064	0.1350606	0.1036405	0.1104397	0.2183995
4.767856	1.971915	8.488181	3.683709	4.754352	4.148192	1.387887	4.476456	1.44893	1.238308	1.891982	3.221873	2.517398	4.452745	1.853928	6.086339	4.216413	1.24599
0.5472297	0.208621	4.980991	0.8904618	6.394518	0.9063258	0.5201737	0.7081711	0.1885036	0.1521286	1.49058	0.2237084	0.535823	0.9076266	0.4165631	6.447497	1.00965	0.3087659
87.97649	26.72921	123.6941	49.37853	124.264	141.9001	30.21067	65.07777	20.70633	23.40293	31.8104	47.75732	21.83791	87.76504	32.36979	200.6459	53.61687	32.88141
0.1683649	0.05209651	0.08585605	0.1181648	0.1046155	0.07174679	0.1073062	0.09055748	0.05897921	0.06512456	0.1152681	0.1112866	0.07929503	0.06566438	0.05295741	0.06007292	0.05416565	0.06846217
0.4920601	0.1853554	0.07806438	0.4834847	0.9512133	0.3822397	0.2166391	0.1747765	0.1026894	0.2172265	0.131009	0.2371572	0.2074936	0.1831863	0.5417031	1.25261	0.4029543	0.3435443

TCGA-S9-A6TY-01	TCGA-DU-5855-01	TCGA-QH-A65Z-01	TCGA-S9-A7QY-01	TCGA-HT-7680-01	TCGA-P5-A5EV-01	TCGA-HT-7482-01	TCGA-DB-A4XF-01	TCGA-FG-A4MT-02	TCGA-DB-A4X9-01	TCGA-HT-A5R9-01	TCGA-S9-A6TX-01	TCGA-S9-A6WE-01	TCGA-HT-8558-01	TCGA-HT-8010-01	TCGA-HT-7694-01	TCGA-DU-7299-01	TCGA-E1-A7YU-01
0.05352011	0.08501737	0.03281463	0.02429913	0.1268546	0.1031007	0.1007441	0.112573	0.3619518	0.02547782	0.09601366	0.07156743	0.2095293	0.0266764	0.07458634	0.03935563	0.0111696	0.0478856
0.06800469	0.06131222	0.1194008	0.0360213	1.429834	0	0.09600692	0	0	0	1.280985	0.06820222	0	0.2372723	0.03210027	0.2917061	0.09934762	0.3593674
0.2645866	0.9366845	0.312544	0.3805866	0.8165976	0.5323399	0.9435476	0.5563877	0.4379921	0.4298625	0.3915139	0.5745304	0.4401405	0.2074988	0.3758245	0.2936278	0.6063956	0.3791231
0.01220597	0.4108443	0.01428727	0.01293072	0.4294739	0.02165716	0.08807472	0.752866	0.146827	0.1801272	0.09580019	0.01224142	0.0366577	0.02484261	0.01536423	0.04537651	0.1545407	0.1003362
4.360498	18.48363	11.57091	11.91131	19.93507	9.150569	12.64508	8.925837	7.986203	3.129972	11.26165	12.62722	14.33414	6.979735	6.164084	13.46518	15.30021	13.24393
0.7325345	2.860072	0.8574429	0.8707875	2.498717	2.070284	4.977077	2.196392	1.762947	1.405147	1.054863	1.456951	1.858414	0.6604869	0.9609002	1.012121	2.189875	1.698731
1.537892	3.997436	2.391111	1.684539	1.180345	1.791671	2.059177	1.482616	1.276835	3.729572	1.024841	1.978876	0.9150232	1.457876	1.386118	0.7667209	1.805835	0.8927677
1.008578	2.501744	0.8523038	1.777299	4.640171	1.581769	2.532884	1.521214	1.563549	1.412271	1.152641	1.199006	0.6815298	0.8454152	1.258707	1.186038	1.750141	1.85459
0.01000751	0.009022651	0.04685577	0.02120345	0.120236	0.1065385	0.01883772	0.01194706	0.08154873	0	0.447708	0.05018288	0.01127068	0.06110436	0.03779077	0.1974651	0.04385971	0.01175204
0.4866493	5.398879	0.6896872	0.9039377	4.202568	2.687974	4.056494	3.480589	2.095841	2.19407	0.4085016	1.595506	1.034707	0.7766398	1.68113	0.5691432	3.358647	2.419193
0.01408306	0.04232374	0.005494813	0.02486545	0.004028621	0.03998032	0.01325469	0	0.01092947	0.02011239	0.06631967	0.01412397	0.01586066	0.01228415	0.02215877	0.008054572	0.02057385	0.005512683
6.014887	85.63458	16.07882	19.10938	88.13861	48.01169	84.12807	45.47479	36.96965	50.39537	5.472817	19.62638	16.98527	17.94793	39.12398	7.582404	65.97903	28.12451
0.2977296	1.044483	0.3346952	0.4559362	0.6223239	0.8441768	1.656332	0.5876952	0.4255157	0.5809601	0.1492294	0.4848464	0.3485894	0.1954171	0.5204058	0.09609947	1.205806	0.5677169
1.023632	0.5305262	0.1829552	0.6883095	0.1104657	0.221864	0.2105682	0.1756199	0.1997923	1.164248	0.3463809	0.05225237	0.2519674	0.02405959	0.1475596	0.1130583	0.1522281	0.3958926
0.3108961	2.162316	0.4381757	0.5847751	1.355811	1.526539	1.970632	0.8634933	0.5908842	1.227793	0.2203568	0.8049508	0.4287406	0.384638	0.7307668	0.3293148	2.14348	0.8121437
0.02163786	0.5120964	0.08231418	0.01719198	0.8913251	0.07486486	0.06618659	0.06457868	0.8060415	0.06180317	0.1846875	0.02170071	0.07919937	0.01415545	0.03064117	0.01392234	0.05926989	0.05717208
0.01295089	0.09341098	0.06821648	0.02743975	0.1000283	0.02757466	0.01828367	0.007730448	0.2035292	0.03699102	0.1143527	0.05195404	0.01458559	0.0112966	0.01833964	0.005555285	0.03783975	0.03041704
0.566139	5.629791	0.6234048	0.7063776	6.506814	1.446488	5.352066	4.706002	3.754126	1.790717	0.5035874	1.488854	2.229233	0.6108774	1.536105	1.269991	2.563914	4.712903
28.77083	543.257	73.66009	68.05507	275.6659	180.7883	123.1055	580.9583	210.3688	49.50987	34.22995	137.3483	28.96961	57.2701	136.5121	37.65255	227.9548	79.86957
0.05842508	1.376583	0.1413341	0.1114095	1.176607	0.3980703	0.6012074	0.3998909	0.154163	0.1279388	0.0779547	0.2304728	0.2105588	0.07814193	0.1103136	0.08687965	0.6458364	0.2332735
3.726695	37.16869	3.868729	5.695438	19.488	7.53803	44.5566	7.067287	8.320952	5.226216	5.157016	7.689487	10.63721	3.357243	5.536879	3.941382	12.15597	6.162748
0.06555787	0.07486785	0.05627343	0.07871056	0.09751863	0.08375033	0.1028362	0.09913389	0.03561433	0.03744996	0.03601778	0.08328117	0.07875492	0.1143676	0.09489892	0.08998735	0.06704115	0.03079442
0.7707605	3.143529	0.9733373	1.246683	1.663026	1.345997	3.462354	1.613875	2.504936	1.322975	0.681275	1.409443	1.374637	1.087992	1.68483	1.424681	2.227134	0.9165422
1.909803	15.99339	4.691997	4.524412	10.05441	8.462143	11.52058	4.885588	6.443665	9.913195	2.563426	6.288033	4.933986	2.896938	5.438629	3.330855	9.933696	10.60365
1.398538	3.06066	1.105332	2.31101	2.764572	1.507951	2.32317	3.531821	2.275094	1.698549	1.660565	2.823183	2.685688	1.128664	1.706804	1.848419	1.875039	2.688094
0.2155313	0.6140526	0.2220087	0.1278641	0.5400995	0.2845197	0.283995	0.2778875	0.471695	0.3570547	0.1725458	0.5187775	0.5825667	0.1052802	0.1302237	0.1627158	0.6360339	0.5365786
2.828957	25.42408	4.897851	4.440172	18.98073	12.50545	10.78761	22.21342	10.44603	3.216204	2.724969	7.479824	4.516812	2.449411	7.795578	3.279679	15.61734	6.211606
1.823992	16.27201	3.613095	3.904227	8.019263	8.623561	13.70282	7.526328	5.553307	11.90902	5.263826	8.039495	6.894807	2.468095	4.397618	3.462627	8.198948	6.634566
0.1252253	0.02454384	0.03823778	0.08075006	0.2943646	0.05796219	0.0204973	0.07149769	0.1901423	0.01555109	0.153837	0.03276236	0.02452723	0.8500913	0.2929807	0.8220792	0.05567766	0.1534486
0.1170573	0.5980455	0.2100932	0.2149468	0.3047187	0.3946218	0.844651	0.4005996	0.2316543	0.3510623	0.2618408	0.4930685	0.1098604	0.06466643	0.3904656	0.1305504	0.4959238	0.2886721
0.03157298	0.06506475	0.03167714	0.05733889	0.05806167	0.04801733	0.04669632	0.03769212	0.06300747	0.05797311	0.03186062	0.02261764	0.01523923	0.09048855	0.05961362	0.1006069	0.02635701	0.04767024
0.1793108	0.9552904	0.2385069	0.4058188	0.4616453	0.3644295	1.618597	0.2529835	0.3415699	0.4306774	0.2590794	0.6130625	0.413067	0.07820324	0.3000878	0.1818	0.130977	0.2297113
1.569632	10.19417	3.213882	3.310948	8.602738	5.752404	14.98659	5.477807	5.945205	5.772699	1.270104	2.985855	3.024477	2.427285	3.601846	1.779561	10.41011	5.845125
0.1090977	1.032792	0.08513367	0.1219964	0.7438061	0.7226724	1.23787	0.6729161	0.4445051	0.4933833	0.1213046	0.4619726	0.3344747	0.1480298	0.2860967	0.1299929	0.7614827	0.2847018
0.7810294	5.534029	1.429913	1.591162	2.956051	2.974113	6.351921	2.211462	2.168098	3.90393	1.485349	2.600951	2.029876	0.8646816	1.88116	1.07379	3.686311	3.527616
1.022622	2.548574	0.8417892	1.62061	1.990651	1.982616	2.155779	1.612867	1.08398	1.84632	1.081578	1.634278	1.423239	1.657084	1.605104	1.722519	1.393739	1.850149
0.7872484	6.308512	2.083659	1.605747	2.031855	2.795728	15.37178	2.237343	2.906626	1.594142	0.8230747	1.09003	1.965113	0.8096779	1.503059	1.466684	6.715988	2.918331
1.186382	1.759858	1.418351	1.707994	1.235685	1.964675	0.8875527	2.148682	3.423182	0.8254299	2.518875	2.954232	2.677971	1.127709	1.933704	1.739825	1.077681	2.214823
0.184686	0.727389	0.2218667	0.3861555	0.5297059	0.4760129	0.7959249	0.5221892	0.4695931	0.5066871	0.3318668	0.4386848	0.3065227	0.3391475	0.3349447	0.2876976	0.6212653	0.6905944
2.037101	9.025383	2.672153	2.634684	13.41008	7.832038	17.84754	6.584557	6.789298	5.592398	2.736675	3.425919	5.503333	1.566135	3.724376	2.054983	9.500034	2.671059
1.870959	5.663862	1.423324	1.57478	5.083267	2.655727	7.228568	3.205868	2.850956	2.53369	1.988103	1.747873	2.09108	1.818269	2.234085	1.71992	4.67194	2.411134
0.5564221	3.55591	1.024841	1.087902	2.492898	2.286679	3.858385	1.392019	1.51932	2.214477	0.7321404	1.177625	1.373112	0.8564952	1.378902	0.5931832	2.880919	2.046738
0.1873008	2.326629	0.3653975	0.6908031	1.291863	0.4652613	1.410269	0.4472029	1.138645	0.9807937	0.2205083	0.7722511	0.8984583	0.3207008	0.576313	0.2797116	1.403603	1.197514
0.3718721	2.285969	1.197025	0.8326727	1.407095	1.142681	2.0284	0.8576198	1.180634	1.07423	1.243759	0.6696188	0.5425502	0.6266255	1.077146	1.036844	1.253215	0.9726447
0.08358814	0.06459606	0.06289791	0.06325096	0.0768579	0.04449335	0.05057449	0.09266056	0.0486528	0.01705348	0.3163111	0.07784301	0.06051782	0.04166341	0.04509272	0.03585513	0.04361192	0.1051708
0.008904557	0.329158	0.02084584	0.01886656	0.3133119	0.1801135	0.3687546	0.08504275	0.3109759	0.4323722	0.06289975	0.07144337	0.1403992	0.00776713	0.04203221	0.03055688	0.3447282	0.1045682
0	0	0.02793569	0	0	0	0	0.05698325	0	0	0	0.02393547	0.02687859	0	0	0	0	0
0.05670706	0.4652504	0.0265506	0.04205191	0.8857053	0.09055426	0.1547774	0.4941912	0.2244447	0.01619697	0.02670438	0.06824613	0.2171402	0.04946356	0.09100933	0.04864899	0.1532595	0.08657007
3.969044	70.41533	11.68562	11.00151	36.18556	32.82876	78.94823	12.654	15.11141	17.48502	10.84075	11.59517	9.823539	2.802229	9.689346	12.154	24.68733	20.22298
4.422336	6.351946	6.318624	4.501629	4.858305	6.100446	8.650104	4.31277	5.599237	4.931942	4.766417	5.552652	7.739702	2.28187	3.247185	3.390187	5.995482	8.021494
1.775055	17.61107	3.163609	3.653843	13.72772	11.8044	20.24042	8.822788	6.760151	10.55449	2.556529	5.470576	5.477845	2.640052	5.536123	2.847785	15.612	8.867438
1.070594	4.948285	1.004028	1.619257	3.304243	4.524627	9.359954	1.547562	1.681737	2.238153	1.138921	1.70111	0.7553955	0.9282161	1.473439	0.9018643	6.958026	2.393268
12.32371	202.5931	20.92449	31.59713	80.6154	54.66026	160.3635	53.49276	46.81029	50.90103	19.83935	46.63284	49.96526	13.09269	32.94215	21.98845	86.49835	89.38497
0.6990486	1.691234	0.9374891	1.146187	6.412132	0.6694032	1.590273	3.413608	0.9446251	0.6923089	0.7899016	1.289422	1.028612	1.265472	1.827283	1.75695	1.149532	0.9075368
0.2369142	2.486153	0.4091485	0.3085838	3.943	1.153575	2.99742	0.7650331	0.6781813	0.5602079	0.3795113	0.3388755	0.4680252	0.2710189	0.8396468	0.3765099	1.509251	0.5381838
1.072467	1.487575	0.9334576	1.281807	2.997118	0.9953569	1.061148	1.493708	0.8803298	1.315621	1.100737	1.282425	1.114926	0.5996637	1.038435	0.7195417	2.058884	2.421966
0.2801306	0.5524804	1.393563	0.148382	4.222091	0.4286955	0.9886995	0.1881127	0.7133487	0.2000309	10.22369	0.5443296	0.8675959	0.6108701	0.925611	2.012714	1.457921	2.775631
0.009436493	0.08082439	0.01656834	0	0.3482248	0.02511488	0.0266443	0.02253075	0.03295529	0.006738251	0.1777526	0.03312366	0.02656897	0.01646224	0.06236035	0.0242867	0.02757143	0.01108148
17.47561	431.4609	40.3311	31.47254	371.6378	166.4424	75.73706	335.0821	156.9653	15.9177	24.29994	72.81034	16.39602	29.26244	115.2654	29.99646	190.671	35.61455
0.07451083	0.2170369	0.02012679	0.01821578	1.293637	0.07322135	0.3398514	0	0.100083	0	1.430531	0.114965	0.1613765	0.1349858	0.1352745	0.2261885	0.2679444	0.06730748
0.1975707	2.171638	0.605211	0.5655602	3.07719	1.154582	0.767536	2.704882	1.585487	0.4922718	0.4107599	0.3836415	0.3077245	0.4766682	1.321505	0.2921099	0.8075491	1.25385
0.2206006	1.318961	0.3397584	0.4796982	1.215608	0.9393925	2.10903	0.5544316	0.9731476	0.5803468	0.2460429	0.4890598	0.5230427	0.4152266	0.7234305	0.1593711	1.416309	0.9271483
0.8196768	7.407845	1.074578	1.340727	4.265623	3.483482	6.116158	3.084351	3.083949	2.434852	0.7720011	2.255726	1.920128	1.332713	1.634317	0.826964	5.16345	3.488334
8.602706	85.26455	17.09331	16.78696	62.37805	41.07803	76.89113	33.70782	33.79603	43.34678	9.190576	27.67484	23.68558	11.97565	23.9611	17.32612	53.78836	31.1052
0.1862737	0.03732048	0	0	0	0	0	0	0.02409363	0.02955804	0.02436657	0	0.04661903	0	0.03907859	0	0	0.02430504
0.07122617	1.463223	0.2143834	0.1563004	0.6811093	0.4387095	0.2968764	0.3522691	0.3079698	0.3051598	0.3893232	0.2245039	0.160433	0.1553202	0.2833762	0.1964087	0.5945935	0.466008
0.03778412	0.1572264	0.04422689	0.04002763	0.5736863	0.08663721	0.04923919	0.0867443	0.2402246	0.01660323	0.5474838	0.06704301	0.2094933	0.07098593	0.1536575	0.06981692	0.1337504	0.05802327
2.744018	17.70589	4.210578	5.964539	9.498651	10.48619	20.52346	9.607279	7.395283	9.665285	2.981669	10.12146	5.449274	4.1635	6.338427	3.257485	13.68523	5.442812
0.5889769	3.416194	1.214669	1.426168	5.167633	1.352563	1.73955	1.61719	1.501862	1.253689	1.225006	1.268572	1.143436	0.9810058	1.46686	1.077938	1.446346	1.505159
0.03848481	0.08096071	0.04504706	0.0475649	0.1266038	0.1365679	0.05433167	0.1072016	0.1120012	0.009160193	0.1359239	0.01286553	0.0505662	0.01678447	0.01816599	0.02751343	0.09838887	0.007532261
5.645729	39.2549	10.05163	11.2651	22.70444	24.2943	59.99775	13.82884	15.88823	14.01207	6.754241	7.879337	17.65522	3.76116	8.365717	8.730788	33.43166	14.78319
1.440754	24.88333	6.25031	7.269009	19.61291	2.636228	11.78032	6.256446	10.18895	6.056995	1.94002	12.76964	10.06018	5.647378	8.688014	5.523476	5.242238	10.45811
0.3076431	1.51037	0.3026059	0.3313875	0.1530841	0.2587072	0.7664489	0.3888712	0.8005253	0.3064396	0.3226201	0.1581575	0.1339313	1.395851	0.8371325	0.8827139	0.3625685	0.3885952
1.068393	2.50445	2.715523	1.067154	17.81363	2.567263	3.878546	0.2550914	2.842798	0.6975082	34.17658	1.867464	4.194177	2.715884	2.881792	5.970825	4.660126	7.169354
2.785624	10.28431	3.681752	4.784589	47.03735	1.999269	3.509378	26.31769	4.550027	2.27327	8.218008	5.663971	6.381905	2.315682	8.239348	3.82553	4.678732	5.319734
0.6986353	3.607892	1.379628	1.414784	4.03784	1.325607	3.90499	1.88935	0.9405358	2.27376	2.568214	0.8579567	0.7975238	0.72547	1.381923	0.7379865	1.447631	0.6753141
0.1863832	1.176286	0.5045052	0.7527784	2.019384	2.405853	0.5701152	0.5006386	0.8407594	0.08318085	1.988567	0.5023599	0.6953234	0.1727364	0.8138013	0.3697645	0.6211526	0.2325536
0.01339578	0.0161033	0.007839981	0.01892157	0.001916008	0.03565237	0.07564698	0.02665334	0.0363863	0.01275391	0.01051385	0.02239115	0.007543312	0.06621308	0.0337238	0.04979969	0.01630817	0.002621826
20.39703	223.2697	23.76693	39.17698	84.19327	81.52019	207.6682	93.65204	80.0949	97.37476	19.72633	78.74209	56.99806	18.7698	53.20941	26.34978	136.2781	81.88222
0.08154266	0.8995131	0.2358101	0.2083389	0.568062	0.561706	0.686202	0.3034917	0.2903571	0.06165169	0.08470568	0.1972329	0.1404538	0.1631732	0.1901889	0.1069907	0.6902251	0.1915147
1.660262	2.939511	0.9223929	2.600029	4.083426	2.375866	4.597228	2.592446	2.142803	1.683799	2.060848	1.665084	1.917247	0.645388	1.629856	0.7947397	2.610013	2.451231
0.02865801	0.1240211	0.05367143	0.05464734	0.3935018	0.006101779	0.0485502	0.04789708	0.1200997	0.04092725	0.06747787	0.09197201	0.05164047	0.01999789	0.05952078	0.1671828	0.1088524	0.04038449
0.3175459	0.353098	0.3469129	0.3812543	0.2543455	0.6310355	0.1593961	0.2653625	0.2587604	0.2418643	1.084151	0.5307805	0.3337855	0.2215873	0.3097753	0.2996649	0.1314386	0.4972016
0.09319161	0.137488	0.1090822	0.2692501	0.42896	0.02705739	0.07176275	0.566379	0.246557	0.0725942	2.663059	0.4163321	0.6678921	0.08128774	0.9357716	0.1090215	0.09901311	0.04974408
0.0262492	0.02366597	0.0102417	0.06488486	0.03003554	0.009314838	0	0.02089103	0	0.01249572	0.02060204	0.05265089	0.009854146	0.02289625	0.008260279	0.01501279	0.01917365	0.01027501
0.1060019	0.006925367	0.00359643	0.05207929	0.0210943	0.01635479	0.02168844	0.01467201	0.02682557	0.006581916	0.02712946	0.03851804	0.02941289	0.04288079	0.01160258	0.04481054	0.01122156	0.02345283
0.005545722	0.07499937	0	0	0.03807402	0.01180779	0.00521952	0.1324107	0.01291164	0	0.0130579	0.04449465	0.04372002	0	0.010471	0	0	0.006512465
0.04046224	0.5399081	0.02368084	0.05572419	0.5139162	0.2369153	0.3160825	0.4782116	0.1836994	0.2080267	0.190544	0.07710157	0.08658202	0.05647005	0.04583857	0.05206884	0.05024438	0.1140377
0.07847119	0.2444046	0.008350157	0.06045859	0.05509862	0.2278344	0.100712	0.2299409	0.06643558	0	0.1091808	0.3648777	0.01606836	0.01244502	0.03367343	0.006120039	0.0260541	0.01675463
0	0.0144275	0.006243651	0.005650827	0.05035411	0.005678609	0.00502035	0.006367904	0.006209473	0	0.03139908	0.0106992	0.006007388	0.004652751	0.005035718	0.009152257	0.0194814	0
0.02913127	0.08889492	0.1049188	0.08308729	0.1211539	0.05009747	0.06748985	0.09898121	0.06260666	0.06720505	0.05803956	0.02696851	0.03028457	0.03322869	0.1163533	0.0288366	0.02946301	0.021052
0.264071	1.064203	0.1824802	0.3977178	1.526283	0.3658026	3.758017	0.7368502	1.207406	1.499419	0.4906809	0.8008956	0.6234705	0.4162766	0.1922305	0.5131414	0.8993777	1.259098
0.15954	0.4660395	0.3809578	0.2839418	0.5531356	0.9171571	0.2462554	0.4037759	0.3268703	0.2278432	0.871511	0.3456073	0.359355	0.1614268	0.3012308	0.2080414	0.4195277	0.2248216
1.27674	3.845701	2.037878	2.059564	6.524269	3.923143	6.96406	3.775821	3.41165	1.947665	1.913037	3.172021	2.516314	0.936483	1.588831	1.593186	5.12925	1.976356
0.310365	10.0359	1.397258	0.7018477	7.667819	2.173081	6.426402	1.189928	5.065125	2.974825	0.8502372	1.173235	5.962346	0.4060806	1.267803	0.5274039	1.569497	1.541982
0.01916563	0.09215736	0.1196462	0.1218218	0.1864067	0.1564264	0.05411489	0.1144005	0.1115543	0.1186074	0.1278604	0.0832923	0.1007289	0.0278625	0.1326858	0.1041339	0.04666491	0.1050309
0.9118764	10.333	1.877854	1.891958	8.403571	4.781345	7.592391	7.403428	3.563368	2.734236	1.260639	3.024384	1.380664	0.8878088	3.286295	1.389311	7.500969	3.074767
0.1461678	2.648475	0.6534748	0.3225974	3.555844	0.3846976	1.364237	1.648022	2.467248	1.62358	0.3226548	1.184955	1.088304	0.4940507	0.7187042	0.7785083	0.7028878	2.865568
16.20504	173.032	15.1952	34.1247	74.70835	51.75493	213.5776	71.61727	74.83011	85.54837	19.71275	61.00333	53.054	16.01592	40.80648	19.81054	113.8048	80.56393
0.06480211	0.1235268	0.08018626	0.1118012	0.1144022	0.08869805	0.3014669	0.06630974	0.03448532	0.1084105	0.02397724	0.05756287	0.08132234	0.0549096	0.08390005	0.03653302	0.1054883	0.08914402
0.05681682	0.2902771	0.1773466	0.1605078	0.4875931	1.270213	0.05347482	0.1130474	0.4188924	0.08114158	0.8918688	0.2469214	0.2132946	0.1321581	0.1609156	0.2437157	0.3873489	0.266885

TCGA-P5-A77W-01	TCGA-DU-6400-01	TCGA-WY-A85A-01	TCGA-FG-A60K-01	TCGA-QH-A65S-01	TCGA-DU-7309-01	TCGA-E1-5303-01	TCGA-E1-A7YH-01	TCGA-S9-A89V-01	TCGA-FG-A713-01	TCGA-HT-7684-01	TCGA-EZ-7264-01	TCGA-HW-7487-01	TCGA-S9-A6U6-01	TCGA-S9-A6U9-01	TCGA-DU-5849-01	TCGA-HW-A5KJ-01	TCGA-FG-7641-01
0.03129375	0.04999843	0.0587718	0.07333475	0	0.07016472	0.05475232	0.01004553	0.07582033	0.03347624	0.02110419	0.06883948	0.0337205	0.02445243	0.1623724	0.3009303	0.008423791	0.05484911
0.1284652	0.3705911	0	0.714992	0.09538038	0.5081776	0.1739259	0	0.1123969	0.1030684	0.08044736	0.1836871	0.4217707	0.1087456	0.1494016	0.2712785	0.1873128	0.0770295
0.5869456	0.2980732	0.464283	0.3384937	0.4970664	0.4868941	1.296276	0.8635022	0.7435502	0.2428131	0.9045339	0.1854592	0.2810255	0.4140408	1.33854	0.457046	0.7622103	0.1594731
0.003842975	0.02660654	0.005519165	0.06304023	0.09701082	0.1664207	0.2663891	0.0841948	0.1075936	0.004110992	0.6593933	0.009419852	0.05383284	0.3860316	0.2145254	0.08115169	0.0179308	0.009217205
15.26696	9.090298	12.55787	28.56354	15.72188	9.481775	23.2737	14.0379	8.03707	17.03526	43.5082	10.46552	11.96929	7.471759	17.17828	14.42562	42.08111	8.313845
1.150742	1.611344	1.916251	1.573635	2.619382	1.576997	6.512679	1.812967	5.218413	0.6825024	2.351583	0.9687924	1.041916	1.796952	4.456991	1.333737	1.485028	0.6341023
1.20044	1.695122	2.243609	1.592833	2.018607	1.693584	1.407064	1.749912	1.464332	1.54803	5.293065	0.7121129	1.017401	2.122051	2.333176	2.523382	1.470606	1.018896
0.9340459	0.8472251	1.888706	1.285009	2.539356	1.594431	2.632345	2.327082	5.616161	1.14335	1.682001	0.9947838	1.143242	0.9545721	2.318458	1.452325	2.005607	0.7300374
0.08507168	0.1599718	0.05430106	0.1218309	0.05614436	0.2204129	0.06142748	0.009861452	0.01102682	0	0.1183856	0.06950888	0.04137826	0.06401164	0.109929	0.3513057	0.03307774	0
0.4163698	0.8562487	2.187651	1.543302	3.388279	1.790394	7.617383	3.698747	3.931454	0.7915918	3.49803	0.5321942	1.04127	2.061231	6.556243	1.170003	0.192349	0.5932541
0.02660379	0.03752004	0.08915099	0.03636743	0	0.05538856	0.009604866	0	0.3413846	0.0379456	0.02776635	0.007245654	0.007763938	0.01501339	0.04125268	0.05992421	0.01551621	0.005317335
5.06798	15.86349	44.64345	36.678	59.99384	42.8013	128.5565	37.61126	71.86116	14.7321	23.14763	8.986817	14.79951	44.64438	113.7619	13.08369	0.3518548	8.602636
0.1893329	0.2077626	0.7397667	0.3066672	0.9302581	0.6121497	1.700847	0.8249621	1.078358	0.2769997	0.7985629	0.193371	0.1803884	0.4493849	1.583419	0.2869241	0.0357258	0.166951
0.3271089	0.3874731	0.4074233	0.3256172	0.7694327	0.1783948	0.1442251	0.3895848	0.229631	0.08980303	0.2574119	0.1702949	0.08616931	0.4672133	0.1110958	0.1760502	0.4322112	0.1284451
0.2846619	0.5255559	1.230772	1.014672	1.566217	0.818495	2.447068	1.36611	2.932752	0.551332	0.8556515	0.3256212	0.4669662	1.099143	1.801632	0.6783135	0.09437164	0.2982535
0.04598469	0.1454289	0.1100697	0.1197355	0.07587076	0.2297741	0.1881562	0.02132206	0.05960441	0.1257123	0.1343837	0.02087354	0.05367991	0.1614708	1.711327	0.08631589	0.1191991	0.006127347
0.01834879	0.09880613	0.02635198	0.03583261	0.03632871	0.1171522	0.112617	0.00638094	0.06421499	0.01962848	0.3600315	0.02998422	0.005354834	0.158774	0.398331	0.08266016	0	0.02933918
0.2891537	1.069526	1.342529	3.745429	4.746605	1.72192	12.09829	7.479677	4.158685	0.5296573	3.98858	0.7864433	1.380221	8.275181	13.33596	0.5855094	0.1755745	0.7030226
33.10006	104.1183	163.9755	108.7111	119.421	121.0487	280.6711	152.5096	262.8566	62.48891	224.5745	22.02007	52.97707	123.2165	285.1448	105.8507	50.01332	22.24396
0.08461599	0.1528258	0.2219114	0.306059	0.3660185	0.1930197	1.43847	0.5181515	1.030015	0.05116194	0.5344893	0.0571128	0.1417219	0.2076155	0.6332223	0.189559	0.03433102	0.03970716
1.99658	8.815929	9.240409	10.87223	7.986015	8.318482	18.71873	4.882359	16.8088	3.981524	23.90018	3.54555	5.279424	4.93028	15.36795	7.754801	1.332747	3.079443
0.05779339	0.02540496	0.07114382	0.05804349	0.07355887	0.04469202	0.120721	0.09905498	0.1300234	0.05299201	0.07238266	0.04047498	0.06505525	0.06988864	0.2448444	0.0697381	0.1059363	0.04455478
1.15976	1.867369	1.434735	1.087127	2.571294	2.536044	4.80317	1.92154	2.451351	1.130091	2.35856	0.8537749	1.0194	6.713387	7.203234	1.441304	0.8572327	0.660982
3.062614	4.6967	7.857397	4.778877	11.41351	4.217851	23.78343	10.03007	9.428995	3.949473	14.75331	1.579151	2.911294	8.692007	21.67589	5.596612	2.456772	2.139479
1.532657	1.830421	1.212054	1.928128	2.229771	2.620936	3.463729	3.189127	2.700017	2.128694	3.832046	1.328381	2.186996	3.427856	7.001736	1.575928	4.349305	1.123789
0.1710039	0.3320022	0.1403375	0.5438559	0.4836715	0.2441326	1.525815	0.5097256	0.3134789	0.1045315	0.6527143	0.2395215	0.1069394	0.2757232	0.4166864	0.6740667	0.06649009	0.06835756
3.164431	5.646362	10.66392	7.569476	8.754444	6.61015	18.89335	9.205475	14.5822	3.104201	16.72461	2.789377	4.310916	5.387015	12.84235	7.913389	4.112396	3.621663
4.598599	4.709951	6.344255	7.774401	8.311035	6.147348	16.99961	8.203388	6.663172	3.090521	11.04865	2.674535	4.223352	10.21603	32.38113	6.212681	7.533456	2.140217
0.02057031	0.1819772	0.02954246	0.1265384	0.09163593	0.8651048	0.05569931	0.005365118	0.04199393	0.3245727	0.1996636	0.09664157	0.4502364	0.09867225	0.04784546	0.5646954	0.01799593	0.2960219
0.2211286	0.1927884	0.518711	0.1252316	0.262687	0.2977158	0.4311082	0.2537675	0.5116208	0.208953	0.604674	0.1053943	0.2613596	0.2578995	0.4629001	0.5385545	0.1590623	0.1325918
0.06816385	0.01966363	0.06118429	0.03494253	0.03795675	0.07095787	0.02768563	0.02666759	0.04472849	0.05924566	0.01067138	0.03828971	0.05594807	0.01923354	0.06440912	0.05397782	0.02981655	0.03065399
0.2617456	0.420472	0.3759111	0.2796303	0.7201859	0.2179796	1.459175	0.41764	1.041756	0.1070589	0.1639101	0.2956334	0.1887201	0.09557808	0.3491684	0.8258366	0.1706185	0.1477141
1.30326	3.014401	4.905367	5.242187	10.97501	4.461565	23.20077	8.157941	6.504778	1.866666	7.580662	1.733052	2.488787	6.698225	11.11312	3.180591	0.4591232	1.520948
0.1202206	0.2422147	0.3370926	0.5232104	0.5525564	0.4624532	2.157783	0.4240492	0.6411187	0.1531012	0.2222684	0.1730679	0.2856894	0.4975257	1.051929	0.2707931	0.006677772	0.1029799
1.011983	1.164093	3.246333	1.828502	4.817116	2.166	6.39209	4.114563	2.471411	1.112913	3.67204	0.8964015	1.068166	2.401801	6.984461	2.260819	0.7832848	0.7939425
1.087618	1.144759	1.736814	1.4216	1.836585	1.876917	2.262441	1.880216	2.171114	1.499491	1.583467	0.5325496	1.598488	1.418116	3.840277	1.691441	1.809114	0.645088
0.8101704	1.716042	2.741497	1.423934	8.23176	2.671086	8.636728	3.780377	1.935541	1.015076	9.528343	0.802513	1.690684	5.887306	6.950389	1.345227	0.04530995	0.9383057
2.154953	1.742468	1.004116	1.986327	2.431953	2.269207	4.325739	3.162477	0.826789	1.383146	2.746928	1.248163	2.238857	3.41592	7.418216	2.471477	5.20611	1.280654
0.2524816	0.3496073	0.5142418	0.7046289	0.7498309	0.3784763	1.422008	0.8333254	0.4819668	0.2750009	0.3794611	0.2550534	0.3697554	0.2072489	0.6459847	0.3877546	0.2302542	0.2202057
2.223149	4.128696	5.131116	6.210263	9.290113	5.5772	15.06959	4.531976	7.683928	1.809955	4.149432	3.337128	2.528576	4.74748	17.20134	5.726582	1.670681	1.066425
1.425579	2.369187	3.136731	3.438447	3.390992	4.213478	7.830936	3.207451	5.804901	1.346605	3.203704	3.072401	2.293687	4.292696	6.981995	2.274257	1.044797	1.345096
0.5371515	0.7461636	2.425321	1.448953	2.071538	1.760377	5.001714	2.302059	3.029416	0.9053264	1.495536	0.6472784	0.7037284	1.439333	4.013316	1.266496	0.2208768	0.5792869
0.2424344	0.5746133	0.451689	1.03645	1.138366	0.7311959	4.222585	1.565404	1.406429	0.3574717	2.199296	0.5085941	0.3614038	2.728878	2.880409	1.034961	0.0687871	0.243588
0.2953651	2.051078	1.077683	1.019544	2.062578	0.7379329	3.04347	1.823895	1.275809	0.9820517	2.049593	0.6652929	0.608046	1.090205	2.933691	1.254181	0.2141694	0.5648214
0.06203344	0.06073507	0.07289227	0.08590109	0.0334963	0.08923253	0.0427563	0.06471787	0.1249955	0.07239248	0.09181907	0.05068519	0.07899741	0.08910999	0.03279239	0.06668853	0.05262537	0.06086636
0.05046382	0.01294009	0.1811865	0.1872426	0.1373805	0.1961207	0.3643828	0.06142219	0.2256651	0.04498606	0.1474733	0.04123209	0.05154501	0.01898559	0.1858456	0.09235538	0.03924288	0.04034508
0.04508466	0	0	0	0.0334736	0	0.02441562	0	0	0	0.02823288	0	0.01973597	0.02544275	0.1310807	0.03808192	0.3155383	0
0.01606849	0.05768463	0.03846179	0.1004144	0.3499533	0.0847508	0.4234923	0.1341107	0.1874488	0.01145942	0.1073322	0.08752638	0.09847653	0.1874048	0.5855331	0.2035899	0.01249556	0.03211629
7.047908	14.81309	23.30972	8.785259	24.97859	21.52373	64.89035	18.58931	13.16108	7.199744	70.00895	13.0451	7.776832	33.00367	72.95837	23.13476	3.49836	4.210156
5.889686	6.471788	5.858113	9.359675	8.396285	3.696355	7.936736	7.153873	8.914347	3.454406	5.99287	4.384962	4.206438	5.171906	8.377585	4.527633	5.54337	4.037163
1.98744	4.40964	7.202539	7.370436	14.64693	7.04039	35.50726	10.36211	12.28047	3.440427	7.718804	2.373799	3.135032	9.37318	23.7994	4.562787	0.6966261	1.972463
0.8954695	1.813522	4.339313	0.9493083	4.097654	3.373584	10.71491	3.126777	3.503386	0.8471413	3.067015	1.090016	0.9759876	5.355945	5.136193	2.485253	0.2842271	0.5698096
18.65038	40.67711	51.41012	41.60639	76.67413	38.70989	213.8874	125.6002	37.74639	21.92381	140.8387	16.70253	28.60357	61.42299	134.0332	47.28265	26.25554	12.64168
0.4280165	1.135819	0.7147726	1.088557	1.847591	1.450663	1.556065	1.491923	3.576436	1.735636	1.018107	0.6641898	0.8453247	0.7339933	1.89462	1.84411	1.137864	0.608785
0.2274414	0.4938491	0.9430543	0.6662416	0.7789643	1.390035	3.214366	0.9299991	4.827192	0.3257129	0.8867303	0.5095444	0.5941681	0.7949582	1.744902	0.6073289	0.1026978	0.2991496
0.6493458	1.278776	1.641325	1.476046	1.079935	1.059909	2.658501	2.140728	1.848304	0.3889942	1.236164	0.7746119	0.8641306	1.055368	2.008758	0.8007904	1.302796	0.5918223
0.8103139	2.925926	0.5699992	4.882923	2.038163	2.83701	1.361256	0.241537	0.4629947	0.5660909	6.296325	0.7026124	2.577134	2.333095	0.7692846	2.081294	1.099522	1.5667
0.0267392	0.03428274	0.01280068	0.02610895	0.006617605	0.02597955	0.02896126	0.009298771	0.1247717	0.004767341	0.005581534	0.0109238	0.01950864	0.005029935	0.01036566	0.1242227	0.03119037	0
19.07969	76.56652	100.983	84.5354	56.7023	94.71949	195.4328	99.39111	201.0009	32.57591	189.6878	19.10432	44.27578	66.59878	172.0568	81.29433	18.20792	11.05078
0.05955048	0.1166082	0.09329957	0.2410451	0.3376335	0.7529143	0.02345425	0	0.04420774	0.06370365	0.04068181	0.04423315	0.3791773	0.1771968	0.1133274	0.256077	0.05051899	0.05193789
0.2421982	0.4001205	1.283008	1.037452	0.6898119	1.058102	2.322223	1.789465	3.663745	0.5776424	0.4226846	0.3698293	0.5457585	1.738754	5.642638	0.4997093	0.08336537	0.4190111
0.28513	0.4471197	0.9922382	0.5525355	1.416748	0.8493545	2.553742	1.155552	0.8059679	0.3284783	1.53831	0.3225725	0.4224565	2.054674	3.086419	0.880001	0.1279209	0.3024817
0.8609223	2.053547	3.12221	2.866609	3.577688	2.85756	9.499076	4.238882	2.897351	0.9275904	6.989246	0.870428	1.361077	3.837839	6.972594	2.113589	0.6718992	0.6833429
11.83062	18.13132	35.29868	21.29824	62.31715	23.84445	129.1905	46.48082	33.01577	11.02715	83.32049	8.707215	13.9228	38.23666	97.91076	26.26078	4.343261	8.534563
0.0390981	0.0751924	0	0	0.02902881	0.01628029	0	0.1223701	0	0	0.07345194	0.03194559	0.06846133	0	0.02273503	0.0165126	0.2508363	0.02344376
0.03844307	0.1996182	0.5659105	0.2477439	0.4067304	0.2361112	0.9472584	0.4662399	0.7623855	0.1953398	0.4152729	0.1138627	0.2019431	0.2711835	0.4862025	0.2070085	0.08407975	0.115255
0.04941457	0.1520524	0.03548386	0.2219493	0.1263711	0.1508909	0.05649436	0.02864052	0.1441128	0.04698742	0.006876523	0.09869394	0.03605235	0.04337863	0.2937245	0.1507252	0.2273594	0.03621403
2.482912	6.386307	8.153571	6.720237	11.78309	9.734141	24.20714	7.127272	11.86113	4.364662	13.08436	3.262521	10.72603	8.13263	27.20387	7.729798	16.9201	3.469487
0.9563189	1.0658	1.327782	1.198133	1.990443	1.1869	2.834793	2.412727	2.367184	1.258223	2.777009	0.571419	1.01353	1.820866	4.688998	1.884072	2.079614	0.6385476
0.01211671	0.1025311	0	0.09228267	0.02698855	0.03531743	0.05905628	0.04424366	0.06360698	0.03888525	0.007587715	0.03960043	0.03712893	0	0.09863988	0.1535203	0.2614739	0.02179604
7.714107	11.33742	18.51418	11.93033	31.26379	13.33714	64.22443	21.15507	14.71645	4.966031	54.44559	7.727054	7.871828	15.17333	35.46374	14.24173	2.03316	5.401574
1.309806	2.826496	6.2052	7.803155	9.636393	7.316832	13.80883	14.25938	7.272738	8.015697	19.70672	1.924958	5.4731	12.26824	26.54842	3.980027	7.053878	4.865264
0.1220919	0.3662939	0.3787445	0.844036	0.3444642	0.8540878	0.3411736	0.5757363	1.088148	1.104934	1.253883	0.09776156	0.808106	0.4795471	0.3691731	0.577517	0.3047703	0.4099647
2.162423	8.184347	1.490691	15.60787	6.165176	13.20622	9.493402	0.4211199	1.412656	1.542156	11.73597	3.038955	8.506854	5.271808	4.15787	9.449331	2.522392	3.423074
1.495768	6.099823	2.850679	5.1402	7.672542	4.725375	6.686615	6.344048	10.59557	5.45847	4.391589	4.200699	3.455335	2.522163	7.667745	7.556714	3.453489	2.003709
2.029029	2.869651	1.012174	0.6522193	1.486477	2.893525	2.562296	0.9881675	2.136572	1.051654	10.98015	0.9279521	1.28123	3.815135	5.345885	2.904481	1.392847	0.5706336
0.4291094	1.785929	0.7742928	0.8379901	1.029313	0.4673153	0.5839403	0.1607051	1.655773	0.4943468	0.9232824	0.3416192	0.2600919	0.4719024	1.791436	1.505596	7.85465	0.1319487
0.05061095	0.03244453	0.03331438	0.002470901	0.01252555	0.03863602	0.01370419	0.00440009	0.01476019	0.006767582	0.02377016	0.006892051	0.01292382	0.03332165	0.0441444	0.03562486	0.007379493	0.01517351
16.23747	55.7349	63.12264	68.86045	134.0097	68.40088	290.2567	135.482	48.24905	36.23284	139.2179	26.70714	33.47257	91.56302	258.2856	60.2509	16.97809	19.32298
0.08155019	0.3310964	0.6831996	0.1274048	0.4171074	0.4603083	0.966691	0.4112161	0.6553628	0.12601	0.243993	0.1443685	0.03966544	0.5778248	0.4267833	0.3558979	0.2695223	0.04346545
0.8181757	1.372436	1.574879	1.904024	4.387237	2.23338	10.02476	2.815633	2.936266	0.8326907	2.028081	1.740887	1.611717	2.828055	7.268531	2.615595	0.5169526	0.7767668
0.03248208	0.1790768	0.02332489	0.1014927	0.1447001	0.1668133	0.02345425	0.02823976	0.07578469	0.02895621	0.2373105	0.06634973	0.1611504	0.1160944	0.06925564	0.09145608	0.1199826	0.06492236
0.3499209	0.3614748	0.4020367	0.3865787	0.2375338	0.3163894	0.2598855	0.2086076	0.1282928	0.4384951	0.4507759	0.2123882	0.183815	0.2482503	0.534847	0.4391321	1.271161	0.3476975
0.08002039	0.1538931	0.3792455	0.03750445	0.1069416	2.558989	0.02600105	0.01669664	0.02800461	0.1968827	0.1403089	0.1699923	0.08407012	0.650277	0.7817175	0.6150806	0.1213434	0.0287888
0.008264401	0	0.01186908	0.08715174	0.03681598	0.04129516	0	0.008622035	0	0.00884077	0.02070131	0.01350506	0.02894217	0.05596646	0.009611281	0.006980739	0.03856057	0
0.0159615	0.02455738	0.004167896	0.01020128	0.01292814	0.03383573	0.02357444	0.0393598	0.005078202	0.01707469	0.001817345	0.06402213	0.05843845	0.006550981	0.0421882	0.1519822	0.05416305	0.01392108
0.047143	0.04432469	0.01504563	0.0245503	0.02333456	0.04798482	0.01134681	0.0710422	0	0.005603427	0.05248329	0.004279864	0.01375801	0.005912074	0.1218357	0.03539604	0.07332093	0
0.01146536	0.04409975	0.005488735	0.2328585	0.1759267	0.257803	1.088657	0.2671405	0.3388346	0.04497159	0.1483831	0.07806597	0.2275282	0.04744871	0.3955728	0.1355832	0.01783194	0.02749916
0.03369024	0.1140342	0.08709282	0.05526552	0.01000548	0.1627303	0.08757583	0.04920741	0.03144139	0.02883189	0.0843899	0.04954865	0.01179842	0.01521	0.1097064	0.02845732	0.03143881	0.01616091
0.005038231	0	0	0	0.007481381	0.03776218	0.005456913	0.01051251	0.02938703	0	0.03155037	0.0164662	0.008822016	0.02843239	0.02929665	0.01276701	0.005876925	0.02416795
0.02963198	0.04884636	0.0395168	0.02480018	0.01571471	0.0511172	0.03209443	0.03091427	0.2567873	0.06113296	0.06097009	0.1106797	0.02964916	0.01911119	0.03692273	0.07330042	0.04690923	0.02792074
0.2043469	0.4762167	0.4963211	1.024647	2.061602	0.7908291	1.969171	0.8245428	0.3120015	0.3696885	0.3838978	0.228348	0.439375	0.8106291	1.212714	0.8376508	0.2278476	0.3207383
0.3375469	0.7094403	0.3549243	0.353133	0.5012308	0.2811063	0.6332669	0.3773077	0.6187802	0.174096	0.9738502	0.2117726	0.184703	0.3401585	0.8061462	0.448042	0.9843423	0.1590268
2.110363	1.771023	1.456378	2.344291	4.354657	2.099862	7.658701	2.430434	3.964567	1.524575	8.581493	1.388396	1.295746	2.381894	4.143618	2.592833	3.165	0.591617
0.6595866	1.465821	1.983617	3.672679	1.741217	5.164343	11.15561	1.25863	2.091321	0.5306983	7.893764	0.7323832	0.5083735	4.154936	12.01762	2.052358	0.3024934	0.4867659
0.066376	0.2413793	0.07799499	0.1767586	0.2060867	0.06532783	0.1176413	0.4028995	0.1478241	0.05809516	0.1058042	0.07395455	0.174338	0.1498326	0.2807038	0.08155083	0.2815467	0.1591999
1.304453	2.449579	4.414075	3.580336	4.977852	3.35723	13.86145	4.187103	8.521656	1.280735	4.337262	1.211822	1.908645	1.484392	3.832095	3.49268	0.4377204	0.8700279
0.1399776	0.6269067	0.4268479	2.878107	2.52559	0.4135917	4.903437	5.201245	1.782796	0.2789675	4.985622	0.3666121	0.730272	4.501572	9.341484	0.3044973	0.1990663	0.4139137
11.87709	50.95917	51.79934	66.29548	130.1062	53.81717	266.2517	122.5663	37.09308	28.97903	134.0081	24.5792	28.42979	80.10318	169.6563	52.42595	19.32076	17.33375
0.06820281	0.02286993	0.1054855	0.09835605	0.08829178	0.1135975	0.1344823	0.1806222	0.2040071	0.04302735	0.04161478	0.0928766	0.05358774	0.09474234	0.1749065	0.0635179	0.07751635	0.06291593
0.1967727	0.7155729	0.3082899	0.4401632	0.1328146	0.3277414	0.2712497	0.1119752	0.4799629	0.153088	6.138739	0.3069353	0.2192603	0.3432309	0.8529547	0.6346167	1.690166	0

TCGA-S9-A7IS-01	TCGA-P5-A72X-01	TCGA-DB-A64U-01	TCGA-DB-A4XG-01	TCGA-DB-A64R-01	TCGA-CS-4943-01	TCGA-W9-A837-01	TCGA-DU-7014-01	TCGA-QH-A6X9-01	TCGA-S9-A7R1-01	TCGA-DU-5874-01	TCGA-DB-A4XC-01	TCGA-P5-A5EW-01	TCGA-CS-6669-01	TCGA-DU-A7TC-01	TCGA-06-2569-01	TCGA-DB-A64O-01	TCGA-TM-A84R-01
0.0250614	0.066596	0.03467558	0.0153903	0.01022673	0.03874433	0.06153402	0.1399798	0.01571446	0.1213103	0.1107874	0.03503908	0.0137339	0.02069221	0.01276998	0.2195597	0.07515071	0.1581778
0.1823791	0.03702099	0.03558701	0.1026664	0.04548066	0.3040677	0.1641937	0.27391	0.2446005	0.3936857	0.1642325	0.4090458	0.1099402	0	0.1362986	0.1237732	0.1307789	0.1406907
0.7811937	0.9170859	0.4877814	0.1563578	0.2402653	0.8617739	0.1852021	0.8337206	0.3766767	0.3278906	0.5080584	0.6007155	0.449983	0.2315732	0.3892098	1.575766	0.5695081	0.3264234
0.2546032	0.02214931	0.01277483	0.004094956	0.04897917	0.02910734	0.02750595	0.1132246	0.02090603	0.1727282	0.02947762	0.1538292	0.07454633	0	0.004077308	0.1875993	0.06259502	0.01262609
12.54121	25.21703	9.318585	3.963602	6.153124	11.24905	5.269606	35.51105	6.182069	11.90945	8.362054	22.6579	6.815673	4.587616	9.906336	7.839452	19.03959	9.930367
2.422262	1.131706	1.200854	1.615784	1.413003	2.46859	0.8012985	2.127755	1.244098	1.017771	1.080072	2.814648	2.50813	0.5118013	1.245638	2.840553	1.937618	0.9140832
2.557653	3.658455	2.028659	1.547833	1.335138	2.288653	1.07051	2.052737	1.46712	1.336384	1.181951	3.171554	2.257945	0.641986	2.09947	6.027681	3.987973	0.900467
0.8708345	2.292654	1.76102	0.6387685	0.8883974	3.370124	0.8410202	1.682592	1.299389	0.8671106	0.9544903	2.857836	1.696805	0.9686714	1.094539	9.60524	8.04217	1.05856
0.1252474	0.03268785	0.04189562	0.02014439	0	0.1252897	0.03866015	0.1026031	0.03085302	0.1158689	0.0886171	0.05159555	0.05392899	0	0	0.1457148	0.0513208	0.0517598
1.445587	1.660839	1.505149	0.4875986	0.7472555	2.632599	0.9146998	2.504301	1.946621	0.3509293	0.606079	4.905509	2.182657	0.6688633	2.26784	0.5428274	3.830207	0.6388432
0.06714453	0.06133329	0.03930506	0.02834818	0.01883714	0.0923549	0.01360112	0.03437812	0.01447263	0.04227388	0.03778981	0.008067534	0.02529718	0.009528508	0.01881734	0.005696032	0.01805529	0.02913558
39.69624	32.11574	27.89681	5.708932	11.62673	48.59495	17.18	20.84727	30.18261	5.56131	8.344139	72.58038	57.60776	7.443825	32.09674	3.872432	69.975	11.85314
0.3425776	0.6162276	0.4782061	0.1780124	0.2799477	0.7591973	0.2419899	0.8397628	0.7724874	0.09859897	0.2539121	0.9068152	0.8196845	0.1735193	0.5228733	0.1895716	0.5971251	0.2134496
0.1671253	0.1167897	0.2726459	1.304776	0.5780088	0.3864364	0.3818253	0.1167097	0.7495919	0.0867398	0.3528031	0.7057766	0.2543399	0.03732483	0.08599594	0.04834342	0.1257346	0.07925634
0.9103514	1.281452	0.9960774	0.1724174	0.2715733	1.392939	0.4289383	0.8479854	0.7889601	0.3224145	0.3728558	1.897289	1.576218	0.2446932	0.6199351	0.1809188	1.427591	0.3708209
0.1208952	0.3416028	0.2830785	0.03266658	0.02894224	0.2708967	0.1044869	0.02773056	0.03891372	0.4871364	0.2525696	0.2324124	0.4955637	0.01647004	0.0162629	0.09189225	0.02080573	0.01678696
0.1215637	0.06345289	0.115213	0.01955191	0.03464556	0.2026746	0.03126924	0.03793727	0.07320031	0.05831309	0.05212771	0.06677071	0.06979045	0	0.01297843	0.1257148	0.04981137	0.04688829
1.74324	0.7944705	1.114822	0.4938254	1.497678	2.43386	1.247434	2.195845	3.167577	0.685159	0.3578443	9.628549	2.006775	0.6682025	3.593171	0.6411453	3.672126	0.5379075
158.5122	216.5951	61.40445	9.759907	38.88268	169.513	45.71563	180.634	62.63525	37.5749	50.57854	358.4413	62.63676	28.67151	108.6591	37.4337	318.3063	51.40814
0.4665817	0.1993174	0.2079031	0.03528164	0.1979746	1.041456	0.1354217	0.2396038	0.09206327	0.03507554	0.08465853	0.7764815	0.2812608	0.04743604	0.2459071	0.2315798	0.5842536	0.1450465
14.65856	7.024204	6.381271	2.016815	6.57821	20.2634	3.215645	17.81117	4.043248	2.169209	3.526241	7.842953	6.41985	2.0337	3.134631	3.254281	6.547986	2.597361
0.02734932	0.1332388	0.08691002	0.0527853	0.02338361	0.04299201	0.05065151	0.08001657	0.08533706	0.01124506	0.04573786	0.06759913	0.08949803	0.04879164	0.06131745	0.03712173	0.05042937	0.07685623
2.340942	1.495711	0.9797236	0.7439298	1.294378	2.285201	0.9885983	2.394908	2.028928	1.507667	0.7144173	2.252236	2.662442	0.3257266	1.310886	0.5487439	2.163352	1.499005
8.705227	7.443392	5.277076	1.758449	3.459809	8.384426	2.721066	6.036286	5.208527	2.429898	3.851953	12.11858	9.420817	1.544203	9.947888	2.654727	10.15715	3.001297
2.82637	3.539946	1.591948	2.015123	1.279076	2.036315	3.065001	7.709877	2.036069	2.844844	1.554032	3.538447	1.165906	1.355674	2.353821	4.565106	2.605223	1.007727
0.5394897	0.2628255	0.1082765	0.37311	0.3459469	1.002248	0.06660975	0.7576312	0.1949142	0.1774551	0.402529	0.7111751	0.7154632	0.09624586	0.2159895	0.1464519	0.3094823	0.1337698
9.879705	8.801383	5.586964	2.035744	3.105249	12.49987	3.59811	12.45582	4.928691	2.745167	3.844038	17.46403	4.644618	1.460231	6.470982	3.559107	16.60169	3.688468
9.628688	11.58964	4.713758	2.635995	4.109998	11.90713	2.759797	9.542139	5.147523	4.30794	4.126366	14.53992	9.63768	2.335299	4.489999	2.57071	6.931443	2.723725
0.12168	0.02371175	0	0.09863591	0.03641265	0.06816381	0.2997207	0.05183391	0.05035671	0.2171323	0.04821209	0.5333401	0.03520804	0.7846425	0.5783526	0.07266974	0.3001513	0.9067484
0.2127756	0.3780997	0.2613604	0.1138868	0.2139828	0.6036492	0.1017462	0.6172159	0.2405907	0.1254918	0.2544257	0.5364535	0.5803399	0.0871202	0.2502531	0.3598216	0.4402174	0.1897018
0.02822477	0.03437596	0.0424857	0.02269791	0.03619818	0.03630125	0	0.04294052	0.01854078	0.00580251	0.05809469	0.06201155	0.01944482	0.1602156	0.03164012	0.1094572	0.06939151	0.07465075
0.7431883	0.3904593	0.4435779	0.1148445	0.4360756	0.728862	0.1259451	0.1850341	0.1507672	0.1153386	0.308376	0.7003561	0.5709834	0.09099042	0.1225173	0.0494482	0.7419074	0.2023446
5.807252	2.813012	4.976812	0.8947414	2.415353	8.322535	2.302942	4.747145	4.41567	1.185358	1.181561	8.617705	8.423583	1.531916	4.482267	0.9438522	7.556732	1.59971
0.3034212	0.1979719	0.3044857	0.06710176	0.1702473	0.9322512	0.2341427	0.4394248	0.2865176	0.07797248	0.1805276	0.9687038	0.8688023	0.07381479	0.1457729	0.07354271	0.4196085	0.1567399
1.924536	2.452983	2.578045	0.7558441	1.767928	3.814555	1.102441	3.29983	2.418194	0.7600167	1.475104	5.437821	3.809551	0.7723346	2.016932	0.947685	4.223536	1.025427
1.215186	2.860464	1.518407	0.7405453	0.9674918	1.750905	1.059892	1.902683	1.439662	0.9198286	1.301753	2.457557	1.402332	1.240357	1.387225	6.874999	3.874557	1.818935
1.17644	1.56076	2.176672	0.47895	1.626654	6.378039	1.259614	4.87895	3.09724	1.224392	0.7756213	3.180406	7.332327	0.5068099	2.749453	0.3849438	2.854655	0.7596501
1.450353	4.929461	1.506988	1.76046	1.396992	1.042793	1.268195	3.542122	1.141047	2.550257	1.889682	4.731226	0.8087175	1.342977	1.239713	1.070421	2.086514	1.384552
0.5213706	0.2857481	0.2899399	0.3473018	0.331542	0.8822831	0.3004056	0.4057529	0.3995682	0.1563107	0.2660469	0.8436005	0.502861	0.4143324	0.4286034	0.5838244	0.4922492	0.3519209
4.394409	3.197658	4.482078	1.320725	2.50746	10.78111	1.943249	5.067335	4.195466	1.377321	2.649291	12.46429	9.0066	0.6130428	3.544327	2.254421	7.354363	1.930538
2.749398	1.832422	2.819504	1.728281	1.900717	3.972693	2.491751	3.382642	2.300234	1.663233	2.386429	5.290414	4.250842	1.219637	2.137486	1.890092	4.611155	1.820472
1.217936	1.670459	1.567217	0.4323674	0.8482328	2.70378	0.7942169	1.510088	1.240332	0.3526457	0.5697843	2.910924	3.395334	2.275439	1.213611	0.3127538	2.926895	0.9183787
1.072845	0.4909401	0.4864446	0.3211678	0.8443747	1.451621	0.3215842	1.320856	0.6986385	0.5354581	0.4411659	1.692892	1.368212	0.1900898	0.9245918	0.286188	1.156184	0.2942093
1.367522	0.7545603	0.9199345	0.5954388	0.9722037	0.967414	0.5468816	0.5756124	1.042252	0.4675281	1.653279	1.525089	1.776241	0.6433115	1.007884	0.1538257	1.961223	1.057847
0.05337421	0.04550445	0.03749303	0.03004587	0.07187486	0.08542757	0.04036382	0.0568416	0.08590039	0.0460857	0.08170797	0.215477	0.01930475	0.02423794	0.03589966	0.0362229	0.04592777	0.04940867
0.1114436	0.09695083	0.2423083	0.02688635	0.01191051	0.1751848	0.08599831	0.1825899	0.00915088	0.05727705	0.0788504	0.07651514	0.2687179	0.06326005	0.02677047	0.06482764	0.1141615	0.08289941
0.02133522	0	0.02497842	0	0	0	0.02304941	0.1747787	0	0	0	0	0	0	0	0.08687606	0	0
0.07097086	0.04321897	0.02373995	0.005707361	0.05309494	0.09127906	0.1314395	0.1619601	0.1689996	0.02188551	0.04108455	0.4093092	0.0611172	0.02302058	0.1591174	0.1651372	0.1235929	0.01759766
13.23247	22.28829	17.60327	3.571731	25.50766	67.60445	3.833217	26.16666	15.85468	12.79513	11.02874	18.26435	102.8665	2.198893	12.59692	8.147464	26.77172	6.994104
6.316717	5.274582	7.28671	5.356429	8.451611	7.643271	3.234996	6.918147	4.146388	2.955883	6.178475	7.19567	5.325683	2.303795	4.480287	20.12029	5.953616	2.999587
8.791197	5.938991	6.603938	1.048445	2.74514	13.27583	3.148325	6.330729	6.135562	1.746831	2.19033	16.94943	14.95292	1.859345	5.676674	1.622436	13.97992	2.862788
2.133209	1.509714	3.11847	0.623141	1.664915	7.151549	1.009043	3.036926	2.240186	0.5061102	1.121423	3.746266	7.812851	0.4974502	2.68864	0.5634379	3.241235	1.227528
60.17166	31.29811	27.10041	21.28084	34.19016	115.9634	21.55729	112.3969	33.15568	26.06118	19.42888	91.23651	75.08136	16.42802	69.77009	28.94899	58.93823	14.29482
0.3391508	1.648431	0.7647162	0.7177092	1.155866	0.5434529	1.109378	1.293983	0.9385948	0.9851653	0.8483488	3.121116	0.427818	0.7629344	0.6758931	5.88633	6.210493	1.587934
0.7603618	0.5624089	0.8617472	0.2267188	0.3896193	1.88939	0.4951227	0.913004	0.6146566	0.2398286	0.4283323	1.374971	1.850163	0.1852586	0.4709439	0.5042451	0.5626616	0.4097869
0.835821	1.137738	1.597332	0.7611152	1.581633	1.131198	0.8498593	1.15796	1.257564	1.238186	1.007233	2.292061	1.022504	0.6698073	0.9507384	1.484828	1.322082	0.7964786
3.145948	0.38125	0.4214549	0.1233494	0.6557172	1.424766	0.6087244	2.551526	0.629737	5.811104	0.6060494	1.895598	1.075578	0.03553774	0.2631809	1.359619	0.897859	1.62997
0.07170417	0.07705682	0.004938139	0	0.006311005	0.07172838	0.004556782	0.04146371	0.0145463	0.03034932	0.04178036	0.05676017	0.04576668	0.02394252	0.009456558	0.02862511	0.01209813	0.01952258
110.7344	102.6113	29.89776	14.1955	29.15424	154.0124	30.43279	126.5077	30.47379	32.57203	39.95524	207.2271	29.64611	14.39814	56.63382	46.04893	174.8628	51.02869
0.1537136	0.08112573	0.07798339	0.02884325	0.01533289	0.2101461	0.04981913	0.2266607	0.06479168	0.1843378	0.1015074	0.4137046	0.09883763	0	0.04020653	0.2016836	0.07348244	0.1304354
0.75157	1.780383	0.8271135	0.008461606	0.5622669	1.033758	0.4465755	0.5202566	1.235495	0.3677323	0.2673318	2.362313	1.630998	0.3455642	0.4675952	0.3774443	2.020986	0.5044048
0.8095851	0.4171634	0.6683415	0.1635985	0.4814294	1.090194	0.3588233	0.9948216	1.038061	0.3435415	0.4860213	1.516462	1.313919	0.2710192	0.6864794	0.07044005	1.10152	0.4203564
3.194802	1.677798	1.599084	0.7787765	1.692808	3.776417	1.323599	2.530752	2.574225	0.9701274	1.076866	5.082463	4.021344	0.8585023	2.799398	0.5251376	3.3712	0.7868414
30.46655	27.76101	24.02323	6.860198	10.75349	65.43404	11.77792	37.97073	24.5264	11.16702	13.28768	50.5936	49.49813	8.573136	39.48534	13.54864	53.22878	10.26476
0.07400889	0	0	0.02083086	0	0	0.03997759	0.03031415	0.02126961	0.1863826	0.01666126	0.1067076	0.0223067	0	0.02074109	0	0.07960454	0
0.5002869	0.3268156	0.1384419	0.03072282	0.07485523	0.5596006	0.09335611	0.2719824	0.3032425	0.07854004	0.1761079	0.699466	0.3235116	0.1239202	0.2396249	0.1172902	0.7631412	0.1473554
0.2208512	0.12658	0.02737734	0.1257861	0.05053907	0.1299563	0.02807008	0.2660617	0.03584246	0.07478152	0.1474027	0.3096868	0.1096378	0.0176985	0.02330121	0.1128529	0.06707273	0.05411717
7.857236	8.987983	6.749372	2.560619	3.803981	13.77457	4.955045	9.82528	6.291512	6.427328	4.17968	13.86329	14.50959	2.878487	5.88869	2.452851	10.32732	3.824375
1.757567	2.610864	1.555742	0.8750621	0.9491119	1.279118	0.8505233	1.645197	1.608287	0.9489275	0.8895576	3.176358	1.269564	1.423207	1.264777	3.933991	3.257727	1.215597
0.09747694	0.04888497	0.04699145	0.01291119	0.05147629	0.09177398	0.01238927	0.09394516	0.03295783	0.04125783	0.05163414	0.1763693	0.04839076	0.01952893	0.02571109	0.02334834	0.05756303	0.02653962
18.6854	9.230363	11.72208	4.983438	9.628879	23.10322	5.84073	24.84733	10.02659	4.771511	7.633573	19.18527	35.76051	3.356289	12.43237	7.138617	27.31289	4.384763
9.217011	8.235468	4.316363	3.507349	4.131253	4.718771	9.24444	7.273637	5.274668	9.302156	1.080078	9.006536	6.230618	3.1803	15.14203	5.736218	8.185091	4.526918
0.5893256	0.2476978	0.1921061	0.1899421	0.6915891	0.5849008	0.4069728	0.3388906	0.3028695	0.36584	0.1540036	0.866363	0.373363	1.781514	0.6684092	5.38601	0.8617496	1.633947
9.605508	0.5982387	1.277925	0.6451791	4.613804	6.578719	1.827814	7.108799	1.756715	13.19473	3.513968	8.446051	3.750541	0.09294011	1.682472	4.629877	3.052565	4.799577
2.573063	7.232133	3.403257	2.935308	3.704105	1.633082	3.098316	5.297597	4.316431	5.480611	4.162343	11.79953	1.507007	1.66528	2.709604	2.146411	22.17929	5.402114
1.533752	2.882223	1.273374	0.6983679	0.8836208	1.802024	0.3901483	1.653229	0.8693681	7.239078	1.740778	2.205271	4.205355	0.6173939	0.4762727	1.395549	2.735817	1.091397
1.541216	2.295644	0.3779473	0.0234485	0.140232	0.770868	0.1012528	1.057827	0.3232221	0.4046215	0.7220653	1.121088	1.393593	0.2837378	0.5136437	0.876345	0.7616659	0.5181457
0.03991733	0.02430837	0.04907029	0.008988244	0.008958923	0.03793427	0.01509358	0.01308016	0.02064951	0.04882743	0.03774286	0.02685838	0.07940666	0.04758336	0.03803541	0.208595	0.02003652	0.1131643
84.52976	54.87315	39.18794	28.92101	49.39445	159.2886	31.20497	124.4456	55.71139	30.56817	27.94326	136.7904	104.8402	15.02344	82.59454	24.33311	82.12097	20.56682
0.3044452	0.3237925	0.3714928	0.02413816	0.1796437	0.5361761	0.06485472	0.3618091	0.2070312	0.06170698	0.1390072	0.5193278	0.6823955	0.04868051	0.2259208	0.09894218	0.3505244	0.14389
2.064969	1.499831	2.209822	0.4601287	0.7402413	3.883884	2.724695	1.502202	2.225464	2.344815	3.191184	3.101372	2.463641	0.8791221	1.597482	0.7809944	3.061721	0.7466987
0.1024757	0.01248088	0.02399489	0.0115373	0.01533289	0.03075309	0.06642551	0.2476478	0.04123107	0.4645312	0.02768383	0.2167024	0.0123547	0	0.00574379	0.09736451	0.02939298	0.00592888
0.2176346	0.5531791	0.3212669	0.2769854	0.1415804	0.3123638	0.1226716	0.4573447	0.1522874	0.3676608	0.7072326	0.6730573	0.3194255	0.2578194	0.371258	0.2054953	0.1492745	0.4379675
0.1590444	0.5442211	0.03546719	0.04263363	0.03399565	0.1590981	0.2127331	3.306874	0.2786022	0.1961801	0.1636795	0.5095857	0.0273925	0.008598115	0.7810781	0.04111883	1.173047	1.77024
0.01564371	0	0.009157513	0	0.03511026	0	0.008450306	0.01281537	0.008991773	0.03376871	0	0.06014782	0.02829062	0	0.01753668	0.01061675	0	0
0.008240066	0.00334529	0.01286285	0.01391571	0.006164583	0.002747616	0.01186949	0.01237552	0.01105129	0.1106754	0.03091744	0.04488265	0.00331147	0.01715048	0	0.02050472	0.02954359	0.034961
0.01983047	0.03019029	0	0.01116314	0.02225345	0.01487787	0.01071188	0	0	0.01426877	0.04017904	0.2382666	0.005977016	0	0.01111503	0.03364533	0	0
0.1265998	0.07489246	0.1312787	0.02036189	0.08659401	0.405256	0.04298533	0.1363057	0.09147937	0.02602665	0.06188743	0.5632485	0.2093233	0.02874532	0.1135352	0.1080113	0.1815622	0.07115337
0.01275447	0.1009718	0	0.01435971	0.06679339	0.1786229	0.006889617	0.125382	0.007331081	0.0917732	0.3560476	0.09807821	0.02306562	0.007239968	0.03574456	0.2943019	0.1097504	0.02213785
0.02861065	0.005807654	0	0	0.02140428	0	0	0.003906321	0	0.006862145	0.008587969	0.01833397	0.01149788	0	0	0.03236146	0	0.0165531
0.04206784	0.1073515	0.02345302	0.03834098	0.05994652	0.01803516	0.04328363	0.03282104	0.05526852	0.02882799	0.1118424	0.08087241	0.03622711	0.0795982	0.042667	0.02719022	0.08331469	0.06258589
1.302635	0.3048347	0.7691963	0.3746507	0.5702499	1.177889	0.1874347	0.4590025	0.3269588	0.2783234	0.2074562	2.394869	0.8606817	0.3842454	0.6121618	0.3706042	0.6118448	0.4475876
0.4735027	0.4794219	0.2938765	0.04495995	0.2902193	0.5649711	0.1417539	0.4626697	0.2688833	0.1641942	0.3082334	0.3838509	0.3301392	0.06476624	0.2302261	0.1935825	0.3272631	0.21124
2.568029	1.769201	2.247317	1.372606	1.97942	8.381349	0.8126911	4.037475	2.087365	2.725029	2.686251	4.138977	4.565928	1.501894	2.384439	27.42736	2.976099	1.650856
3.436134	1.969025	1.261838	0.1561856	1.421043	4.248582	1.400726	1.263204	1.398475	1.873517	0.5765667	1.405255	4.078354	0.3513311	0.7476561	2.636133	1.438582	0.3395695
0.1599097	0.1391141	0.05349024	0.1285968	0.09399667	0.03427793	0.1604179	0.1076059	0.098479	0.139717	0.02571407	0.2525194	0.06196845	0.02593469	0.09603192	0.1860414	0.05733336	0.07269276
4.275441	3.711786	3.465051	0.5026794	1.325984	6.817976	1.037803	5.719196	1.827542	0.5743799	1.245779	6.944728	3.874082	1.056017	1.789713	6.781036	6.10249	1.737795
2.689566	0.5570047	0.5396789	0.1389396	1.023713	0.7134657	0.3784025	0.4950734	0.9680272	0.475323	0.2484057	7.06844	0.665149	0.2905075	1.293894	0.4754151	2.071761	0.2456979
87.16515	35.83836	34.49624	24.79132	46.44107	128.8194	24.64977	122.9442	40.42921	31.98557	20.40463	122.5901	95.44858	13.00746	78.37513	23.54737	68.98465	14.27057
0.07944695	0.07861853	0.09688885	0.04285951	0.04457707	0.09934222	0.1162283	0.06101547	0.1084542	0.06907445	0.03875193	0.09227502	0.1396836	0.02066964	0.04453022	0.0269587	0.09969604	0.03064345
0.3894022	0.3917856	0.07928633	0.07624544	0.1266612	0.3387249	0.1280358	0.4022166	0.2919426	0.3411002	0.2591812	0.5533114	0.1632947	0.0384419	0.2467297	0.183841	0.1942465	0.2154988

TCGA-HT-7854-01	TCGA-HT-7688-01	TCGA-DU-6408-01	TCGA-HT-7481-01	TCGA-DU-A7TB-01	TCGA-HT-7681-01	TCGA-HT-7474-01	TCGA-DU-7302-01	TCGA-DH-A66F-01	TCGA-HT-A5RC-01	TCGA-P5-A72W-01	TCGA-HW-7493-01	TCGA-HT-A61A-01	TCGA-TM-A7CA-01	TCGA-DU-6406-01	TCGA-HT-A74L-01	TCGA-CS-5396-01	TCGA-DB-5273-01
0.2821914	0.03076279	0.01820072	0.03789912	0.05425298	0.1400912	0.02336628	0.02185546	0.3167045	0.06365401	0.08821327	0.186404	0.03974377	0.03208806	0.1587327	0.02477254	0.326628	0.02992877
0.5882675	0.1795622	0.1040694	0.03370924	0.05790616	0.04063161	0	0.3563865	0.078979	0	0	0.9879648	0	0.04281093	0.02861844	0.1502308	0.1013435	0.03071544
0.9092122	0.3424451	0.4853893	0.3657843	0.9301217	0.6352441	0.4268968	0.2567344	0.2762741	0.8574455	0.3592537	0.6517946	0.2970089	0.3789735	4.390508	0.512648	0.508247	0.8354379
1.263518	0.2946662	0.1079238	0.02420151	0.006928942	0.02430951	0.03391174	0.04264454	0.0236262	0.208079	0.1408274	0.2201301	0	0.0614721	0.1986168	0.1222391	0.06063288	0.4777957
30.9956	15.96579	8.874021	9.935903	6.706682	8.784433	16.05044	6.743481	29.70895	7.534846	12.12553	12.29464	7.503799	6.582395	13.095	7.928213	5.418004	18.41628
3.868731	1.106428	2.22158	0.8164242	1.335492	2.078733	0.8393571	0.9463245	0.7522402	4.205787	1.612415	2.646832	0.9582795	1.495103	1.553695	1.820627	2.577454	3.190199
2.776138	1.300302	2.007116	0.828481	1.087148	2.309295	1.775191	1.094876	1.853469	2.61594	4.25984	1.726915	1.552433	2.367395	3.419125	1.343573	0.6789015	3.313179
4.515512	0.9575982	1.500789	1.424225	2.048568	2.751418	1.353221	0.9047584	0.8856458	3.025205	1.432794	1.242198	1.38692	2.508744	4.960774	1.586823	1.143767	3.444333
0.06059824	0.1358959	0.08167868	0	0.05964992	0.05979311	0.03336453	0.0381422	0.244072	0.02380481	0.02388879	0.2005352	0.03601432	0.01260003	0.06738341	0.01768626	0.1491362	0.01808022
5.423541	0.7342672	3.154815	0.943274	1.298896	1.030062	1.182289	0.8109529	0.74006	4.404553	2.289488	2.827121	1.225138	1.692535	3.723077	2.437465	2.200069	3.414342
0.04872962	0.07082944	0.004789262	0.01396166	0.003997254	0.03365753	0.1643328	0.03131076	0	0	0.02241166	0.1081778	0.02252495	0.02364186	0.01580421	0.1576302	0.04197437	0.0466462
102.1212	14.99961	80.9892	15.63306	28.45744	36.26139	25.29935	16.10344	5.29074	124.8316	34.29223	75.54686	25.73565	34.21738	54.29543	34.3703	23.42714	140.1577
1.178091	0.2446253	0.911247	0.1490428	0.4166724	0.5847416	0.32432	0.2050422	0.2362232	1.00271	0.6473762	1.001233	0.2510655	0.649508	0.8113075	0.8960643	0.2767569	1.427294
0.2518588	0.1271652	0.1782235	0.4830974	0.1565794	0.2490357	0.3422956	0.2832624	0.08186496	0.1385127	0.05121105	0.1719571	0.6654324	1.412291	0.3662881	0.7420412	0.8243883	0.2491654
2.200896	0.47866	1.802757	0.4874847	0.6807311	0.7960927	0.5235443	0.6196733	0.3315923	2.150665	1.226796	1.013945	0.3805547	1.038504	1.991888	0.6615782	0.335664	2.536181
1.464652	0.2366956	0.2759415	0.02145134	0.009212343	0.0646412	0.07664825	0.06700656	0.3832277	0.1608433	0.0645643	0.5311472	0.02595626	0.0476758	0.04552933	0.1481822	0.09673701	0.1808022
0.3921063	0.03419604	0.09248909	0.006419627	0.01654158	0.04642759	0.02158882	0.01851018	0.04512256	0.07701555	0.0309149	0.136246	0.1398203	0.04076482	0.09810231	0.0457762	0.06754994	0.07019381
10.92648	0.9206411	3.478345	1.014423	1.38193	1.718853	1.219871	0.5474313	0.681852	5.456515	0.8358801	3.042023	1.292857	0.9979227	4.030811	8.226637	3.109135	4.064779
390.9091	83.23531	288.5192	49.80663	123.1281	179.3307	100.0517	41.7772	32.08388	295.3411	224.8391	182.2905	90.87733	77.59532	590.0547	47.37607	143.5024	460.6927
1.175898	0.1733677	0.4251915	0.1042586	0.2454289	0.2932269	0.1493362	0.1002055	0.1447541	1.278574	0.2882292	0.6009846	0.1074642	0.4315553	1.003151	0.3613911	0.3453681	1.833132
13.35473	6.512446	9.648453	4.815055	3.069247	10.84116	6.329424	5.763004	4.164903	17.91099	8.844901	18.07557	1.986055	3.761392	18.72198	1.436004	4.975553	34.36534
0.0831749	0.1186982	0.04904774	0.06499275	0.03349361	0.1201204	0.032785	0.02498645	0.05583404	0.09876337	0.0469477	0.08757863	0.03145667	0.05502744	0.05517748	0.1158604	0.08141432	0.07896078
5.813454	1.817782	2.842743	1.014805	1.393353	1.717129	1.382113	1.079607	0.821724	1.902807	0.7313056	2.906265	0.872086	1.053087	3.319312	1.654364	1.641404	2.727907
13.57953	3.530695	9.800955	3.401772	6.513744	7.523566	3.048029	1.804738	3.467148	16.24939	11.2594	5.819283	3.69448	7.378808	13.25227	5.920498	4.171372	17.47464
4.673723	1.133978	2.472533	2.319692	1.74303	1.960451	1.579126	1.716981	1.426406	2.790017	0.8524415	1.640755	1.605959	1.399643	3.340132	2.479557	1.527495	2.302636
1.237982	0.2601586	0.2902532	0.264955	0.2275715	0.6902402	0.2011997	0.3285867	0.2503128	0.2973562	0.2469561	0.7514915	0.09307665	0.3256396	0.6748235	0.7008711	0.8415283	0.7632102
21.32096	5.096902	16.03339	6.98042	8.321531	10.78107	5.718054	4.516855	4.060437	16.59986	11.8965	11.13564	5.553083	7.561214	25.08556	4.809211	6.924535	23.40754
15.90367	3.658854	8.927397	3.936462	2.767764	6.695304	4.02288	3.101611	3.351323	7.309075	7.831666	7.58647	3.276417	5.529294	8.301793	11.38984	4.627896	11.57173
0.381492	1.117227	0.1166478	0.1619294	0.03245251	0.2016885	0.3721151	1.224323	0.3035137	0.08418145	0	0.3436683	0.9796784	0.04113025	0.03665991	0.048111	0.2028437	0.01967307
0.5029205	0.288477	0.3980795	0.197282	0.1960266	0.4429512	0.2504188	0.275124	0.2764265	0.2552398	0.5588516	0.5394991	0.09829339	0.3733674	0.5615781	0.555115	0.3052766	0.50756
0.04682037	0.0884706	0.05061777	0.01788618	0.03840639	0.1024062	0.04887199	0.06016797	0.0314298	0.05364464	0.05383389	0.06326743	0.1244438	0	0.05694372	0.03188509	0.03696898	0.02852092
1.579312	0.2213576	0.3159805	0.1696849	0.4580508	0.4090612	0.2717334	0.3106444	0.3596998	0.7658076	0.4572144	0.3919762	0.08799426	0.3899539	0.665415	0.3168959	0.9352587	0.9497765
12.42441	2.445142	8.762636	2.676127	3.512867	3.386139	2.971584	2.726505	2.269255	9.141447	3.945795	7.668414	2.899323	4.465526	6.075489	5.13478	2.484036	13.30484
1.137729	0.1828989	0.7482059	0.2944281	0.1496673	0.6518396	0.3132087	0.1963549	0.2041333	0.6848183	0.2025533	1.032349	0.1963065	0.595228	0.6121548	1.49962	0.6277475	0.9362395
4.738012	1.548584	3.003398	1.250787	2.012198	2.249499	1.685867	0.9159331	1.442007	3.44175	6.023397	2.859251	1.273229	2.647511	2.562025	3.884341	1.439983	5.119612
1.388045	1.574316	1.747311	1.079746	1.313227	1.520046	1.503727	1.081341	1.13937	3.362001	1.567853	1.616019	1.979697	1.538768	3.323845	1.899113	0.873487	1.787454
4.162238	1.923556	4.830975	2.603482	1.465752	2.197387	1.880344	1.276326	0.9415835	2.746049	2.910002	5.756791	1.134647	2.544551	2.605881	0.7579488	0.4640236	6.14028
6.249708	1.04419	1.220713	2.060785	1.519628	1.19958	1.479026	1.086954	2.543697	1.784881	0.6474866	1.600125	1.739413	0.8489971	3.115076	3.817055	2.379998	1.080858
0.622226	0.3299901	0.5107171	0.3517332	0.3559056	0.4065404	0.4010373	0.3565832	0.3781795	0.6647479	0.3016421	0.6232969	0.5480324	0.6241606	0.392698	0.8073992	0.3223054	0.9834364
15.39829	3.117621	8.028388	2.709241	2.38374	5.022848	3.364546	2.258581	5.805741	8.412984	3.878256	11.24021	2.853433	4.557946	7.310494	8.834763	12.86325	10.39382
7.235006	2.101095	4.28823	2.792128	2.25996	3.120495	1.962924	2.811114	1.008722	4.660431	1.281382	4.904665	2.23411	2.380744	5.704438	2.549109	2.353194	5.587359
3.475465	0.845715	2.704788	0.6570835	1.114811	1.584038	1.009384	1.04087	0.5844446	2.724973	2.446478	2.795661	1.47727	1.911108	1.911153	1.977574	0.8170102	4.220651
4.170583	0.3611046	1.465005	0.5089179	0.6379484	0.779218	0.5203763	0.4759136	0.5317318	1.452103	0.6623753	1.549942	0.3994341	0.7511387	1.541405	1.483445	1.235381	1.516489
2.40532	0.726865	1.621032	0.6954391	0.7772318	0.9998418	0.9228079	0.4912363	1.266203	2.352153	1.593809	1.00768	0.8719013	0.9364171	3.06588	0.8215115	0.4219324	2.89353
0.1394493	0.0810769	0.1522823	0.08878671	0.06100757	0.1141542	0.02488202	0.06257906	0.04853862	0.1207186	0.04275688	0.1196413	0.05729735	0.06013847	0.2814114	0.05275901	0.093425	0.08090134
0.2850034	0.2754255	0.09993044	0.0353112	0.05307577	0.117047	0.04453102	0.05090768	0.06204923	0.1694498	0.2656994	0.3925542	0.0106817	0.1681703	0.05995692	0.09442209	0.1924144	0.329795
0.1032258	0	0.04869732	0	0.02032208	0	0	0	0.02771756	0	0.02848528	0	0.02862927	0	0.04017439	0	0.08891591	0
0.9124018	0.1368974	0.2082727	0.09557102	0.04345758	0.06098666	0.07089695	0.0810491	0.3622202	0.2427998	0.02707294	0.2386278	0.1836661	0.07853724	0.1431843	0.06514192	0.6380303	0.08708311
32.96853	15.14054	32.71824	8.897345	7.498848	23.39071	8.904757	5.161128	10.83516	21.94788	37.48539	45.19378	4.167425	22.82276	34.04255	16.46341	8.712213	59.34838
4.603766	3.268385	5.274292	3.883358	7.436971	4.581585	4.135891	4.609436	5.996011	8.617995	8.375813	3.889988	3.810583	4.496207	8.655789	6.843227	7.730003	6.564293
21.32655	3.49931	13.68914	2.892676	4.699881	8.07887	3.537809	3.17528	3.569265	19.76475	8.149673	12.30649	4.056578	6.681855	12.55828	6.617581	6.514688	18.21397
4.502218	1.459641	5.276963	1.291542	0.7303894	2.928571	1.585762	1.437979	1.250853	3.881291	2.586392	7.172565	0.9670633	3.24805	2.659806	2.518948	1.398418	9.682684
77.079	28.16303	69.57457	42.26699	34.2636	64.38251	31.72673	20.96501	30.52253	81.24027	70.65222	42.13383	26.39389	40.76071	76.35938	50.86412	50.6491	163.6465
4.062765	1.494657	1.297345	0.8741141	0.3499662	2.568981	1.203359	1.405799	3.129122	8.83636	0.3899199	1.851291	0.8975566	0.5882361	5.36917	1.831413	1.028665	3.204967
3.292482	0.9258837	2.595347	0.5698534	0.8730742	0.7100811	0.6700864	0.6143122	0.3157419	2.23571	0.5191795	2.190808	0.4099888	0.6845972	1.588673	0.9094676	1.565618	1.803318
2.093332	0.632743	1.360674	0.7224461	0.7258838	1.330852	0.6761462	0.6812595	1.213601	1.308242	1.575429	1.239843	1.039102	1.263741	1.481278	1.506577	0.7581383	1.37867
6.527606	2.734129	1.482557	1.284436	0.7155958	0.355668	0.2918567	1.851754	6.466072	0.8954015	0.08358702	5.61339	0.1680191	0.3747446	0.3094546	6.544268	1.421983	0.6642595
0.07346647	0.04627346	0.009627275	0.0187103	0.004017598	0.2931833	0.0353934	0.03147012	0.01643897	0.2020188	0	0.1890929	0.02829949	0.01188109	0.1151637	0.004169276	0.10547	0.1193401
311.2336	77.86743	219.1413	31.29408	92.66935	164.2008	60.31578	40.82884	33.92747	194.4585	113.848	175.5886	66.20782	37.37013	497.4496	41.00117	136.8688	366.0183
0.391687	0.3372708	0.1227971	0.06250415	0.07808758	0.102736	0.03344053	0.2129906	0.03328272	0.2454072	0	0.9360483	0.04812847	0.0721642	0.06271284	0.03545309	0.4441571	0.01553264
7.308978	0.7896451	2.148598	0.3625634	1.363752	0.6329222	0.5290548	0.3805116	0.6102494	2.154818	0.5970487	2.013201	0.8118549	0.386361	2.044982	1.894414	0.7611287	1.712573
1.546713	0.3591244	1.741258	0.2992723	0.3064789	0.6937104	0.4838627	0.4093309	0.3910424	1.463752	0.9146084	1.023694	0.5292545	1.008667	1.846936	0.5232428	0.3720049	1.269072
5.797217	1.538512	4.943902	1.39119	1.541089	2.209718	1.595933	1.387092	1.104265	3.111818	2.794081	4.375638	1.604688	2.130091	3.896497	2.740778	1.573315	5.455569
80.54822	16.11506	48.53893	17.94268	23.01741	37.98702	21.7859	10.47291	16.9824	49.4951	53.93441	32.16168	14.58256	29.55993	43.86831	36.00119	25.27851	84.51737
0.01790379	0.0156141	0	0.02051867	0	0.07419686	0	0	0.02403709	0	0.2964343	0.02073689	0.02482773	0	0	0	0.03084368	0
0.8581875	0.2110971	0.622853	0.1714868	0.4028844	0.2735768	0.2586667	0.1890584	0.1654407	0.7745157	0.5647194	0.4383738	0.2746326	0.1857613	0.6594309	0.3191905	0.2426156	0.7766871
0.2388502	0.2543502	0.127505	0.07491684	0.03217327	0.4792921	0.1356604	0.221552	0.09788965	0.2419763	0.05897299	0.3465354	0.09064989	0.03659416	0.1785771	0.02825132	0.3075258	0.05251022
13.56724	4.865066	12.5742	4.602521	3.969497	7.733677	4.968207	4.899645	5.739876	9.804485	9.389551	11.27828	4.909829	7.964607	10.54816	10.51744	6.494699	20.43764
2.712419	0.8675027	1.559438	1.120532	1.416184	1.337233	1.180509	0.7696463	0.6123639	2.868707	1.275387	1.472465	1.285199	0.9498981	3.5078	2.205713	1.19328	1.940687
0.1997453	0.09193899	0.03926288	0.06358844	0.02184661	0.206946	0.05880712	0.0305582	0.1042891	0.1220582	0.03827774	0.2120736	0.03077698	0.01615154	0.1511587	0.02833924	0.2007311	0.05794099
33.41201	9.510836	19.79607	10.95865	10.06588	13.18723	9.121946	7.97346	9.379002	23.46897	20.9131	28.37448	5.413604	12.23824	32.30909	23.5396	11.46307	42.97842
22.95546	7.669583	8.038418	3.454784	3.672748	5.14343	4.30805	2.925795	5.16618	9.060431	4.900866	10.16783	4.558376	4.94313	6.47239	6.22238	4.549112	5.938004
0.7245715	1.078533	0.6277258	0.2383545	0.1959188	0.6889065	0.7218453	0.44586	0.09307536	0.6733689	0.3610142	0.8495886	1.361422	0.3645558	0.3394391	0.2946931	0.1155789	0.9738322
20.86056	8.382474	4.204258	3.389385	1.507567	1.896799	1.526557	6.45707	19.81743	3.41271	0.5100691	24.8038	0.4760298	1.076135	2.003985	11.67967	6.027481	2.757469
8.738021	3.222172	3.353604	3.656029	1.443604	11.50722	5.245594	6.06167	15.46038	11.97181	2.095056	9.312379	3.547557	2.385076	19.0294	5.474584	13.29053	18.06993
17.36985	1.150923	2.211011	0.7774228	0.4653899	1.556105	1.192336	1.539685	1.490286	6.992164	1.333028	5.437934	0.5701133	0.6223179	18.41243	1.041514	2.843649	3.456025
1.380522	0.7294121	0.4991475	0.7506549	0.267816	4.148187	0.9806348	0.2774895	0.4870372	1.385465	0.500527	2.544356	0.4751095	0.2786674	0.9216196	0.2676333	1.354062	1.231177
0.01351919	0.03705505	0.006833303	0.02877399	0.009505448	0.03468288	0.0241913	0.01276403	0.01555753	0.02389835	0.04263584	0.06934465	0.02946031	0.06184223	0.02818675	0.007891448	0.01996297	0.0786555
128.303	33.2837	118.8128	49.12732	43.95954	67.41402	41.7539	28.23494	39.30226	109.8095	105.8508	84.50777	30.29195	70.98	117.6287	82.73937	71.27259	222.8437
0.6514358	0.2460667	0.5040411	0.1521689	0.191964	0.9858698	0.3198343	0.1691054	0.1448379	0.6788778	0.4007488	1.143793	0.143848	0.2717654	1.308031	0.1992112	0.1572591	1.273888
7.259016	1.175372	2.448687	2.075329	1.731291	1.365766	1.514166	1.364157	2.05394	3.791859	1.579532	3.441425	1.392685	1.666234	2.815006	4.943454	1.721179	3.934178
0.4610999	0.1902553	0.1754244	0.1079617	0.06344616	0.05479251	0.0238861	0.1365324	0.1597571	0.05453493	0.0410455	0.08039679	0.04125297	0.01443284	0.05306471	0.02532364	0.1921834	0.04659791
0.4303474	0.2874722	0.3347648	0.5771492	2.415494	0.4932932	0.1940918	0.2420562	0.3073251	0.4154397	0.2779369	0.3181568	0.3047365	0.2132313	0.2405393	0.3647791	0.3864636	0.334658
0.02198576	0.2556537	0.2765839	0.09238829	0.07213899	0.3340821	0.1129804	0.05650696	1.072466	0.03022833	0.05055828	1.41754	0.09146493	0.1600004	0.07843571	0.05988998	0.3535081	0.03826502
0.03784433	0.01980269	0.04463316	0.00867431	0	0	0	0	0	0	0.02088637	0.01753313	0	0.03304927	0	0.02319509	0.02607847	0
0.04385454	0.01738457	0.01567319	0.02893732	0.006540643	0.01101466	0.0217677	0.02195713	0.02497844	0.03837007	0.001833592	0.05387251	0.04422866	0.00193424	0.002586018	0.01900521	0.1167594	0.006938769
0.01918908	0.01673502	0.005657843	0.005497918	0.004722196	0.07952333	0.009244581	0	0.04508468	0.01978737	0.0661906	0.03333834	0	0.01396477	0.0560114	0.1176114	1.69835	0.01001927
0.4480185	0.1465208	0.2765779	0.1203404	0.0344537	0.2175793	0.0741945	0.1002405	0.1409756	0.5534221	0.1303923	0.4418868	0.1067826	0.09169977	0.2894716	0.1001124	0.2894332	0.5190224
0.1234194	0.0430542	0.08005768	0.0141445	0.0182232	0.5540974	0.04756709	0.07477044	0.06627966	0.016969	0.6811546	0.2787512	0.02567242	0.06287259	0.07805449	0.0630373	0.1116257	0.0837739
0.04614212	0.01207232	0.01088389	0.005288124	0.01362601	0.03187034	0.00889182	0.02033018	0	0	0.006366485	0.04275492	0.006398667	0	0.02244753	0.009426958	0.01192367	0.01927389
0.1802748	0.04902551	0.0320064	0.02665861	0.08777277	0.166021	0.03922243	0.01067593	0.06506222	0.07462474	0.04546772	0.1167492	0.06720229	0.008464145	0.1848329	0.07128508	0.02838522	0.0870427
0.8586836	0.4560413	1.006229	0.4825853	0.3928226	0.8820348	0.6974248	0.1970496	0.6133712	1.195744	1.101232	0.8829923	0.4808848	0.556805	1.582585	0.6241307	0.9814543	2.052053
0.8114895	0.2888609	0.2994885	0.1897977	0.2716974	1.418397	0.3989238	0.3222739	0.2964574	0.5616555	0.5103199	0.9015364	0.2143461	0.2490789	0.9077228	0.2932331	0.6181588	0.5130591
4.869537	1.729359	4.677349	2.330103	1.704845	4.022195	2.805479	1.963035	2.257863	7.074773	4.398371	5.087713	2.610593	2.192032	3.321436	3.589699	5.945794	6.762659
12.1951	2.228859	6.162298	0.4970371	0.5997045	1.426444	0.70641	0.881497	1.129873	4.00366	0.9403327	8.724877	0.6586963	1.134729	4.701996	5.701316	3.277661	4.000563
0.08289515	0.04819588	0.07169474	0.06966821	0.1142371	0.1068772	0.0745468	0.08522159	0.1261315	0.1595621	0.1448751	0.08961165	0.03831773	0.06434838	0.1451786	0.4629091	0.07140363	0.0634808
11.77977	2.443435	6.627992	1.965578	3.225068	4.571148	2.28642	1.903586	2.640987	8.536798	4.01927	4.575734	2.056104	3.163245	11.59516	4.002148	2.48665	10.38718
10.50864	0.5237852	1.480873	0.3501938	0.8816073	0.6355878	0.5042367	0.2224522	0.4526819	4.517536	0.7608288	1.000734	0.4432191	0.4421921	1.862441	4.610332	0.7820541	1.368062
118.0633	31.70434	101.7887	51.79521	37.31157	63.66738	36.64138	28.57544	33.66671	111.5712	88.1853	69.34283	26.07727	53.50788	107.6964	65.22065	71.06299	169.8886
0.08969024	0.05726813	0.1397802	0.0697501	0.06621499	0.08186119	0.04166621	0.04939681	0.04300549	0.08367841	0.02872782	0.2355914	0.07107209	0.1049009	0.1215494	0.06871483	0.06622006	0.07860801
0.9174443	0.5429343	0.3091494	0.2065325	0.1935186	1.086306	0.4104196	0.234595	0.1759618	0.3829246	0.20344	0.6072119	0.06815612	0.07153568	0.4144442	0.1004123	0.4939135	0.4448124

TCGA-CS-6668-01	TCGA-DU-5871-01	TCGA-DU-7292-01	TCGA-R8-A6YH-01	TCGA-HT-8107-01	TCGA-DU-A5TS-01	TCGA-DU-A6S3-01	TCGA-HT-7610-01	TCGA-DU-A6S8-01	TCGA-P5-A781-01	TCGA-HT-7881-01	TCGA-HT-8109-01	TCGA-FG-8185-01	TCGA-E1-A7YW-01	TCGA-HT-7607-01	TCGA-VM-A8C9-01	TCGA-P5-A5F2-01	TCGA-HW-8319-01
0.02422132	0.1796811	0.05893449	0.02088376	0.03914823	0.04397472	0.05417047	0.01737684	0.03030111	0.03279931	0.0175169	0.009308735	0.05923988	0.03135909	0.1653583	0.3404573	0.0280167	0.02339716
0	0.2207994	0.08424496	0	0.02748968	0	0.08030289	0.1159182	0.08085365	0.03366142	0.05842629	0.03104858	0.02634536	0.1521394	0.02865143	0.09463081	0.04153226	0.1418899
0.209934	0.5156652	0.6314889	0.4052751	0.7673243	0.411858	0.3038362	0.2648099	0.4069314	0.4205358	0.1710112	0.4122752	0.3611086	0.6027918	0.3865811	1.155211	0.2757408	0.4740563
0	0.1509739	0.03024178	0.03750719	0.1052596	0.01755078	0.009608893	0.0693528	0.009674796	0.01208359	0.01048677	0.02600651	0.07565846	0.03185826	0.01714188	1.460711	0.04969672	0.06451748
6.313872	11.92345	11.473	5.588838	7.493584	10.32399	11.74041	3.502339	8.251579	11.91069	15.86794	6.04552	14.1068	6.690783	23.60395	102.8981	5.632884	6.287665
0.7606799	2.745367	1.082598	1.909773	1.048555	1.301657	0.4352359	0.9684274	0.922281	1.279388	0.5273355	1.630707	0.8220877	2.870521	1.369659	11.23642	1.077475	1.51876
1.020356	4.602958	2.669617	1.438521	1.210488	3.50472	1.466513	0.9615218	0.8417837	1.826967	1.23652	1.531478	3.134064	1.713787	1.652853	5.378335	1.105812	1.588644
0.5985021	3.171987	2.22664	1.164095	0.9267708	5.051007	0.8191568	0.895653	1.164044	2.771351	1.627368	1.307319	1.368501	1.733561	2.201342	8.427666	1.225921	1.145599
0.08559897	0.05570167	0.04958964	0.03075163	0.04045356	0.01438964	0.1536249	0.02729348	0.03569505	0.01981434	0.03439184	0.01827633	0.007753917	0	0.02529791	0.1392578	0.0611185	0.08352158
0.5143551	4.079222	1.097596	2.369398	1.104451	3.250837	0.4947717	1.374853	0.7939506	1.365375	0.7649638	1.916988	1.623221	3.288152	1.463733	8.630362	1.524699	1.708389
0.01784579	0.0304835	0.01163083	0.009616714	0.02656652	0.0337497	0.07760616	0.01280291	0.05581315	0.03717829	0.05646421	0.01285968	0.003637232	0.01050216	0.05537852	0.04572645	0.01146786	0.05484999
4.711461	70.45966	40.42675	37.45968	13.37447	42.48636	9.769877	37.54151	12.97503	22.39249	10.37173	36.44784	20.53589	40.53258	36.07711	323.0011	12.91133	56.67577
0.1764986	1.19228	0.2458872	0.5525515	0.1715908	1.106281	0.2123356	0.3517319	0.2488398	0.5136142	0.1722182	0.7698386	0.4499478	1.586057	0.3949438	2.699108	0.3888671	0.7380652
0.6087568	0.2956799	0.07593333	0.1192894	0.09167691	0.1145763	0.0723802	0.4200166	0.1530409	0.2912677	0.2896407	0.3134357	0.1828451	0.6445079	0.1136286	0.2558835	0.1385083	0.6650335
0.3467266	2.165998	0.521382	1.049573	0.3821521	1.350221	0.3858502	0.6489084	0.6261206	0.8699465	0.2398508	0.9182932	0.6169615	1.408811	0.8260097	1.253722	0.5618675	1.450917
0.06169295	0.1053815	0.02680521	0.8920743	0.1049606	0.03889093	0.08942822	0.08114277	0.04502078	0.03213136	0.01859018	0.06915367	0.4275133	0.09076501	0.10028	0.3989549	0.2576888	0.112867
0.03077087	0.07208451	0.01604371	0.1392868	0.05758684	0.1210423	0.007646489	0.03090585	0.03079573	0.02564208	0.02781693	0.01182586	0.05518964	0.05794723	0.07093336	0.2342808	0.05536617	0.09187374
0.3228304	8.48858	1.014777	2.568689	2.508892	2.007972	0.1683684	1.867134	0.6132749	0.9424076	0.8394883	2.109958	2.138013	6.581426	1.848113	5.572966	1.885011	1.361481
45.70201	195.8903	108.1744	97.08913	60.92103	187.8116	32.78499	41.08149	75.83255	94.80065	34.79408	102.8636	140.8665	68.51168	177.249	2120.854	81.87913	133.7109
0.06663167	0.6070274	0.08041959	0.3431053	0.1542997	0.1736177	0.05979209	0.07967137	0.2130231	0.2159333	0.07027434	0.3236551	0.3923256	0.3921241	0.4299489	1.149047	0.1760299	0.3185715
3.756629	17.01358	11.39788	6.01991	6.603683	8.740599	2.936167	6.081627	8.011803	7.445188	2.089039	4.379218	6.035307	3.088911	8.257257	44.8276	2.962519	11.49776
0.0290758	0.085142	0.03609503	0.08953328	0.07420167	0	0.03612634	0.0715184	0.06755192	0.04759374	0.04881422	0.1476618	0.1083624	0.07333272	0.09943398	0.1155529	0.06939907	0.0510663
0.9936392	4.926106	1.534148	2.482997	2.288095	2.062701	0.8871785	1.614701	1.075385	1.119275	0.5681964	1.831669	0.8703569	2.268293	1.344007	3.440922	0.8048338	1.643683
1.949684	10.40605	3.219511	7.201335	2.447507	9.347225	3.258857	3.323349	4.797052	6.004388	2.262874	4.565761	5.195332	13.41642	4.155781	58.53193	4.325813	6.034055
1.337672	4.20815	1.495598	1.998769	4.684542	2.891796	1.369155	1.393662	1.115627	3.055993	1.586669	1.931945	1.88241	2.309827	1.500763	3.609457	1.357839	2.159828
0.4342564	0.4318719	0.1566415	0.4062085	0.04181984	0.5578365	0.08144278	0.3115438	0.2870047	0.1621614	0.05184868	0.1889359	0.120237	0.5882651	0.1162325	0.4798704	0.2000786	0.3021985
3.871058	10.71653	6.624477	6.165536	2.702949	15.05155	2.134016	2.696604	5.487327	6.942994	3.135167	7.045113	11.16956	6.686571	8.802955	88.48137	5.272303	7.842986
2.240211	11.00702	3.406753	10.14145	4.23106	9.959499	4.66115	3.354648	3.664414	5.782882	1.997028	4.360306	7.743869	10.82933	6.841408	29.85901	4.992826	5.761259
0.01034891	0.176776	0.2922749	0.005576803	0.3125241	0.04697207	0.1414423	0.4900171	0.04531303	0.3826893	0.06081029	0.5220193	0.01265554	0.09135421	0.6009981	0.06061052	0.9376919	0.2499193
0.1631661	0.7854667	0.09989702	0.5915068	0.0946366	0.6059335	0.2349847	0.1888897	0.2041223	0.3128857	0.1542071	0.2565326	0.3174399	0.3622665	0.3254991	1.009912	0.2049379	0.3777529
0.01714659	0.04602579	0.04097548	0.04157967	0.1859724	0.05188379	0.02663054	0.09225965	0.02681318	0.04018683	0.09687841	0.03294887	0.04193668	0.01009068	0.08741438	0.04393487	0.01101855	0.06022965
0.735881	0.4763382	0.1817446	0.2254076	0.09884073	0.597693	0.1251179	0.1611591	0.5426661	0.4115074	0.1750626	0.4391054	0.2210279	0.2826302	0.5013537	2.01882	0.2588415	0.4489527
1.342141	8.367159	2.875608	3.334067	2.144932	6.750877	0.9677512	3.58948	2.361653	3.786184	1.837848	4.659214	2.47897	8.297239	3.542594	17.31249	2.149109	5.417749
0.1094452	0.764668	0.2002243	0.4656131	0.1519027	0.3921748	0.05725657	0.3636626	0.07926774	0.2700096	0.1249752	0.6807397	0.1784522	0.664419	0.3115375	0.5144777	0.1776769	0.4856093
0.6280827	3.344	0.9261996	4.030754	0.6152437	4.736885	0.9577455	1.645367	1.464329	2.319595	0.9807246	1.874388	2.738689	5.253136	2.092483	12.03872	1.956904	2.640782
0.6242195	1.519383	2.272751	1.869242	2.376076	2.235506	1.556079	1.43418	1.018142	1.27575	1.139306	1.241255	1.214274	2.022751	1.698867	6.062268	1.462343	1.682653
0.7537735	5.553569	1.64968	3.273609	2.460354	4.249121	0.5411204	2.04692	1.152529	2.268272	0.6460801	2.714504	0.8421199	4.429349	1.623747	10.82402	1.37062	2.157409
1.402174	1.857791	1.507388	1.012662	2.426562	2.529661	1.63442	1.19953	1.356289	1.315024	1.267568	1.620354	1.453469	1.826155	1.508068	1.573285	1.572966	1.764484
0.2494275	0.4283167	0.3251242	0.5027971	0.3221997	0.7547416	0.1262599	0.4838116	0.33515	0.3512343	0.2547122	0.4659858	0.3841013	0.6632591	0.3235303	1.602848	0.4749164	0.5475918
3.642433	10.39554	3.715705	5.299638	2.253153	4.60033	1.377381	3.695336	3.159986	4.185943	1.299211	4.815821	2.443401	4.916195	5.142553	12.78221	2.177831	5.879224
2.02442	4.770981	3.229113	1.83764	1.79118	2.587856	1.261022	2.662008	1.459274	2.077734	0.9028066	3.549478	1.308425	3.115378	3.232243	5.765747	1.495699	2.740318
0.618248	2.744067	0.7805329	2.388698	0.6681557	2.029592	0.6377225	1.71838	0.8463997	1.450014	0.819013	1.501843	1.027083	2.581176	1.406576	6.251173	0.9495018	1.884852
0.3823878	1.460807	0.5729144	0.7602924	0.8188222	1.067293	0.2621309	0.8702954	0.3299109	0.4875935	0.2145595	0.8678196	0.5428658	2.056339	0.496858	2.422944	0.7117573	0.9321259
1.389591	1.615099	0.7189388	0.545384	0.7447804	2.527717	0.8383252	0.5071034	0.8239778	0.5940498	0.6825558	0.9106592	0.7727128	1.125022	1.018391	7.373983	0.9703881	1.333139
0.05106922	0.06646436	0.04930948	0.05504021	0.02413501	0.06868007	0.0634529	0.02035446	0.1206775	0.04728574	0.0512963	0.07632689	0.08789525	0.1202159	0.06540297	0.07477438	0.0437567	0.1096256
0.008462768	0.2230319	0.02941617	0.1276913	0.03599508	0.01280373	0.08411911	0.0485708	0.04234802	0.2468277	0.007650358	0.1057033	0.1103894	0.1892513	0.1575684	0.2849928	0.1196415	0.2229494
0.02268205	0.02213978	0	0.04889148	0	0	0	0	0	0	0	0	0.01849173	0.08008967	0	0	0.02915137	0
0.07545105	0.3840175	0.1404987	0.2729958	0.09169127	0.06523059	0.02008865	0.03093144	0.02696857	0.01684155	0.0633359	0.2640829	0.03954348	0.1141781	0.1099012	0.06312783	0.08311801	0.07572337
8.218793	41.04715	6.370753	66.93137	15.9158	19.95445	6.881353	12.41028	12.2582	17.0853	2.551488	15.40095	28.96528	51.84587	22.5848	81.68927	19.93954	16.93672
4.373938	5.12966	4.246718	10.64735	2.920668	7.761927	6.317172	4.638497	6.895339	5.583828	3.062157	3.406219	8.777983	13.16051	3.598102	11.71057	5.773621	5.671985
2.317454	20.57719	3.65744	9.090816	2.319155	8.659276	2.200101	4.749234	3.872732	4.853624	1.772164	5.80196	4.077805	16.35413	6.880298	35.28745	3.950141	8.246334
0.9623137	3.781159	1.214411	2.378156	3.556003	2.095798	0.7691847	2.088736	1.288211	3.102964	0.5984255	3.398018	1.254255	4.739748	2.434627	7.745	1.323436	4.04231
19.64769	88.59368	29.18408	88.23333	17.90032	104.9865	13.29335	30.70259	23.78922	46.29203	15.37034	33.5066	74.28545	114.0768	45.56276	434.1551	40.27056	37.57
0.6576891	1.13403	2.674847	0.6908384	1.684108	0.7172437	0.5641382	0.7448823	0.6264787	1.704017	1.167978	2.011198	2.803411	1.406729	3.12872	8.642318	0.4462357	1.80889
0.3912617	1.729392	0.6607579	0.7120907	0.872905	0.5249444	0.2338966	0.9665563	0.4063542	0.5305961	0.3270068	1.131319	0.4694411	1.086111	0.9065225	0.9295729	0.5408077	2.350023
1.071528	1.50509	0.5223494	1.211181	0.5891504	3.459805	0.6007359	0.5437389	1.193365	1.306725	0.9332242	0.8411961	2.460915	2.076329	0.8805227	3.195212	0.8229288	1.273753
0.2163137	1.42927	0.2602717	0.251067	1.104069	0.1258736	1.302487	0.6326878	0.8326479	0.363985	0.4362225	0.527579	0.162786	0.7246284	1.372022	5.091909	1.881915	1.052073
0.01345247	0.02188475	0.06234683	0.02899698	0.003814534	0	0.03342906	0.01286807	0.02243889	0.03269658	0.01216106	0.05600889	0.0329017	0	0.06361186	0.2626239	0.01152623	0.03150238
41.49529	130.4324	83.83439	50.27507	36.48911	93.23051	13.19402	23.5773	51.69833	56.50554	18.63178	85.27848	95.98671	35.26361	166.2126	1113.473	45.4141	101.7502
0.04902512	0.1967292	0.1893433	0.02348318	0.180718	0.03296554	0.054145	0.1914896	0.1090327	0.1021344	0.05909173	0.2512177	0.01776361	0.08334746	0.1255703	0.09570861	0.1330168	0.1148048
0.2317142	1.824994	0.4999204	0.4865461	0.7102835	1.239089	0.2531554	0.9085667	0.5497662	0.6824833	0.4478326	0.9788085	1.006418	1.434159	0.8536486	1.918634	0.8112569	0.908651
0.3862079	1.314026	0.4506763	0.7492289	0.2816016	0.8848161	0.2056538	0.6412258	0.4693456	0.5057424	0.1895292	1.02839	0.5757424	1.649414	0.352203	4.943883	0.6807239	1.317846
1.489466	3.814092	1.13186	2.982198	1.044383	4.403253	1.006626	2.351751	2.144006	1.545017	0.9126769	1.862198	2.179642	4.357045	1.475303	15.58526	1.257503	3.354794
9.929187	49.86415	15.58875	36.63886	10.22052	49.56257	10.02086	17.99384	17.80109	27.51025	9.977045	21.82842	30.40835	49.46151	22.92487	271.1164	22.24902	28.50821
0.1376915	0.0383999	0	0.02119971	0.0334657	0	0	0.02822357	0.04921527	0.04097912	0.01778191	0.05669742	0	0.02315165	0	0	0.02528051	0
0.0773627	1.113819	0.2563039	0.2553457	0.05347068	0.468183	0.1682139	0.1699733	0.2842956	0.2216092	0.1311301	0.1579514	0.3350624	0.3300749	0.2486435	3.157476	0.2672125	0.4670647
0.05800767	0.04583591	0.2232352	0.04763289	0.1785831	0.1546293	0.07207384	0.1565547	0.1002132	0.06042392	0.1623113	0.2866307	0.05179514	0.03251165	0.117556	0.2467116	0.02485082	0.06064273
3.217842	17.88644	4.142028	10.39235	3.004527	7.918795	4.438334	6.223366	4.710947	6.543963	2.344318	6.829117	6.277035	9.044977	5.968313	19.66419	3.304746	7.919224
0.594407	2.081421	1.663096	1.172088	2.095134	1.419534	1.215451	0.881562	0.9870325	1.16892	0.980715	1.101236	0.9844017	1.866676	1.498324	12.66058	1.339468	1.282721
0.05486314	0.05355149	0.06356721	0	0.04667039	0.01844558	0.03787045	0.06997308	0.07626036	0.06349824	0.0606178	0.1932791	0.02981841	0.007174821	0.07566649	0.2677645	0.03133825	0.03747217
7.206258	28.86471	13.27878	18.05967	4.963199	19.2045	5.134887	8.408883	10.29676	16.96633	3.233689	13.39708	11.53757	30.24797	13.9177	76.6808	7.476238	18.71022
1.429178	11.46915	4.261261	9.656892	8.364705	8.170022	2.79652	5.028363	1.5845	4.519614	5.136973	6.059966	7.465325	11.80512	5.288592	36.68889	12.14271	5.749154
0.1302196	0.733859	0.5871468	0.4660514	2.102612	0.806648	0.5525494	0.7385624	0.2551168	0.3173551	0.5530559	0.6893123	0.2423711	0.6709053	1.145839	3.611833	0.4357687	0.5652939
0.6092313	7.787292	2.26892	1.156872	5.083828	1.887363	3.676676	2.788949	3.447841	1.450533	0.7867803	2.843125	0.5203322	1.843867	5.375843	13.93254	7.457092	2.955245
5.691173	4.39453	8.538239	2.727641	2.445995	2.880601	1.277775	2.297011	6.565562	6.465202	4.770151	6.344165	8.631206	5.335593	13.51164	69.01844	2.786595	4.990925
0.9214324	8.319478	3.516859	1.767099	2.170913	0.8952176	1.189766	0.8230759	0.8984442	1.47736	0.449154	1.47986	1.355115	2.482693	4.397168	5.628031	1.288733	3.173189
0.4096271	0.6699918	1.144847	0.3698873	0.4143815	0.5192454	0.6327571	0.3177019	0.803296	0.6227363	1.020837	1.010516	0.3068746	0.1433351	1.305497	13.68116	0.3414875	0.2333301
0.01909674	0.02278245	0.05715999	0.04573699	0.04873503	0.03852317	0.005272772	0.03957883	0.03450808	0.0596766	0.03069065	0.08358599	0.01210905	0.01997927	0.06208234	0.009320337	0.01636231	0.03540324
30.63239	117.6174	39.94511	133.2869	27.01801	113.2546	26.45376	45.50876	42.69787	62.71958	19.43976	55.92364	68.82737	131.0667	73.06214	438.929	43.91376	65.14158
0.07293837	0.26253	0.174302	0.2014377	0.5080051	0.2827769	0.1019532	0.1733344	0.2281165	0.5365844	0.0782995	0.4555145	0.1523755	0.5365484	0.4850144	2.162592	0.1054594	0.6925559
2.10957	2.650648	0.8693995	1.719067	0.9872438	1.556912	0.745846	2.095856	1.330274	2.382052	1.01813	2.603627	0.6571762	4.31319	1.865318	3.247828	1.227046	1.460943
0.0762613	0.1807782	0.04260223	0	0.01853518	0.06593108	0	0.0312636	0.02044363	0.02269654	0.01969724	0.05233702	0.1110226	0.01923403	0.04346664	0.08773289	0.03500441	0.0287012
0.2917315	0.1865649	0.3496706	0.1517869	0.2139371	0.1369784	0.2999776	0.1587746	0.2894502	0.4401069	0.2455379	0.3769492	0.2952453	0.1776027	0.133787	0.6186262	0.3361517	0.3091905
0.03220655	0.7859142	0.8256195	0.03471083	0.4794496	0.2070891	0.160065	0.1963975	0.07050874	0.2096755	0.02911478	0.5028407	0.3675931	0.1231971	0.4426019	2.180969	0.05174053	0.1414122
0.04989376	0.008116818	0	0	0.007073847	0	0	0.005965788	0	0	0.007517341	0	0.006779381	0.05872447	0	0.01217555	0.03206216	0.007302433
0.02044056	0.03562834	0.02030011	0.006294272	0.04347038	0.02208964	0.01088449	0.01675936	0.005479571	0.02585456	0.05015539	0.0490982	0.004761236	0.01031072	0.038835	0.01710205	0.01125881	0.01666789
0.02108231	0.04115656	0.0137402	0.03976281	0.03586815	0.01594822	0.00654863	0	0.006593544	0	0	0.02531985	0.01289064	0.03722051	0.02803799	0.007717059	0	0.0138852
0.05768213	0.2177053	0.05346674	0.09117874	0.1112217	0.1512681	0.04777958	0.1103527	0.03848582	0.0801131	0.03476317	0.1145367	0.09091659	0.1312565	0.3136716	0.2984142	0.05930727	0.2431393
0.1966145	0.3110329	0.02356629	0.07306989	0	0.0102575	0.02527151	0.01945586	0.03392644	0.0776844	0.03064481	0.02605616	0.392438	0.09575727	0.2404446	0.4169275	0.01742706	0.0773987
0	0.009896521	0.004405298	0	0.03449947	0	0	0.003636926	0.01268389	0.005280623	0.0137484	0.004870735	0.004132918	0.01193341	0.01797873	0	0.006515358	0.008903573
0.07027974	0.03949669	0.08698167	0.01377172	0.0507265	0.04833163	0.03175336	0.03208546	0.09324915	0.06433357	0.02310291	0.07570956	0.03993366	0.04010597	0.09629943	0.1496756	0.04379381	0.01870203
0.4475117	0.8824797	0.5701854	0.4594969	0.5247272	0.7365336	0.3230973	0.7505707	0.5484933	0.9480531	0.2706121	0.3573392	0.3820933	0.6334838	0.7399274	6.450536	0.341981	0.6797595
0.3396391	0.5742384	0.2846014	0.2418534	0.2786019	0.5964404	0.2336058	0.1348852	0.2807321	0.3032455	0.2522068	0.372943	0.3362278	0.3854751	0.3925454	2.13125	0.1636913	0.2236926
1.185819	3.499325	3.091387	2.080506	1.548299	4.839852	1.130723	1.464046	2.518451	3.70564	0.9473336	2.649616	1.68619	2.823854	1.931588	24.67089	1.488593	1.695199
0.2836217	3.931142	0.8971284	5.428793	1.022978	1.965301	0.521547	1.062143	0.7238196	0.8094939	0.517917	1.111815	1.812803	5.020653	0.6739252	3.189292	1.720514	1.120791
0.1517896	0.1481606	0.03693292	0.1177864	0.06714382	0.03674396	0.1282458	0.07840564	0.0835517	0.04427143	0.06037595	0.1341722	0.08909833	0.2072396	0.06998141	0.3111455	0.2340991	0.06398171
1.639774	5.984589	2.381155	2.077326	0.8504026	4.216429	0.8578523	1.633035	2.073866	3.914343	0.8322012	3.558681	3.03134	3.758416	4.259542	18.08587	1.585221	4.29152
0.1408452	1.802279	0.5365162	0.5489641	1.244082	0.9662093	0.2589019	0.6422587	0.3161922	0.2090143	0.5005764	0.6840351	0.9437686	3.201925	0.7954438	3.805225	1.755614	0.6336704
25.63704	100.5863	42.52134	94.45303	26.04229	132.6874	20.8634	40.10795	31.91522	55.32668	17.59934	45.42666	59.3951	110.8287	66.42032	507.5784	39.15737	59.15728
0.05806777	0.1099239	0.04893109	0.1308552	0.06885568	0.06921793	0.05247165	0.08710502	0.1342801	0.1173072	0.1097589	0.106511	0.04447111	0.186396	0.06240489	0.04122254	0.04296862	0.2626896
0.3239875	0.3162417	0.3441059	0.1745899	0.2143604	0.1089281	0.4472785	0.3357391	0.3602769	0.374981	0.2440711	0.3285808	0.08804448	0.2754051	0.1755439	2.767188	0.09253209	0.3003185

TCGA-FG-6690-01	TCGA-DU-6397-01	TCGA-DU-A6S6-01	TCGA-DU-7294-01	TCGA-TM-A84S-01	TCGA-HW-A5KL-01	TCGA-HT-7874-01	TCGA-HW-7495-01	TCGA-FG-A4MY-01	TCGA-12-1597-01	TCGA-DU-7304-02	TCGA-HT-7605-01	TCGA-HT-7902-01	TCGA-VM-A8CE-01	TCGA-TM-A84O-01	TCGA-HT-7467-01	TCGA-QH-A6X4-01	TCGA-S9-A6U8-01
0.1243709	0.2222042	0.02511486	0.03851387	0.09027352	0.01064948	0.06099084	0.03681635	0.3508258	0.1145169	0	0.05705283	0.08938945	0.05015398	0.596297	0.02376152	0.01010241	0.09013612
0.3883513	0.5195657	0	0.4836146	0	0	0.4940452	0.1052556	0.173356	0.4583551	0.1615607	0.02455425	0.5714576	0.03521786	0	0.1219304	0	0.0308351
0.6225318	0.6741904	0.1704352	0.3560376	0.4548688	0.537586	0.3049773	0.2053857	0.3671471	1.519737	0.2306738	0.3470758	0.4806821	0.4450089	0.7220607	0.3568851	0.259796	0.6978099
0.08871428	0.05119904	0.01002361	0.04701809	0.01859568	0.06800514	0.1078007	0.03778405	0.2212635	1.20661	0.01933205	0.01762869	0.1129749	0.06742564	0.05262057	0.008753978	0.01209594	0.04058637
8.059439	13.50241	4.541699	13.14053	15.78189	8.239891	8.59523	5.206069	25.12182	18.56749	13.4509	7.171496	14.64686	26.49529	9.381272	8.817015	4.456935	8.840302
2.762147	2.496496	0.4540205	1.466861	1.515423	1.986407	0.8923434	0.6858461	2.177932	7.012727	0.4958339	1.020112	1.247468	1.188397	1.796884	1.015337	0.8986264	4.741008
1.175	1.371887	2.065955	0.6964409	2.201501	1.673246	0.7192118	1.323388	3.777352	1.616752	3.731742	2.066068	1.878586	3.690646	2.067447	1.32355	0.7418857	2.547193
1.767482	1.070172	0.7151229	0.8921879	2.175542	2.045547	1.269906	0.9290126	1.479918	2.932847	0.7636377	1.353625	2.106689	1.910913	2.741863	1.933612	0.8532199	2.806356
0.1350805	0.1619128	0.03698199	0.08006432	0.06860854	0.02090868	0.0769799	0.08260967	0.3061309	0.4796524	0.1585009	0.03613381	0.1901282	0.0932873	0.05883121	0.05741814	0.02975189	0
4.536267	1.50449	0.5107495	1.103809	2.456114	2.9568	0.8505209	0.7678574	1.209031	3.418957	0.3698288	1.024336	1.529245	1.22282	2.650473	1.122183	1.68035	4.330072
0	0.02109738	0.01156509	0.01669192	0.0107277	0.01961582	0.03209776	0.00484385	0.01595565	0.0140623	0.007435009	0.03728947	0.1989533	0.06807033	0.02207738	0.07743491	0.01395611	0.004257085
71.96765	6.356922	10.88452	19.43779	29.70203	48.98396	13.71982	15.09055	27.80501	71.60344	7.034333	20.51581	40.49297	20.88903	38.70178	25.29887	20.89168	136.9196
0.9671883	0.5458215	0.1706639	0.2227359	0.9734183	1.053168	0.2897393	0.2007519	0.3907545	1.015498	0.1820837	0.5300512	0.4243403	0.4121815	0.8837974	0.3699746	0.4936905	1.275133
0.8146304	0.2066053	0.8305451	0.1580141	0.3957098	0.4642331	0.08906052	0.2213657	0.2135453	0.1927957	0.04368634	0.2213172	0.2284257	0.136496	0.4324048	0.1274849	0.2460083	0.3779835
1.630488	0.6073674	0.4572125	0.4709515	1.181701	1.448243	0.544997	0.3895391	0.684702	0.8362815	0.1607847	0.6322896	1.01302	1.179608	1.510623	0.5460515	1.015451	3.046644
0.1572662	0.2333878	0.06663422	0.01923467	0.1297999	0.02260398	0.06472784	0.03907214	0.00919312	0.2916805	0.01713523	0.03515722	0.08696094	0.1568795	0.1717235	0.03491644	0.0107214	0.1962234
0.05378769	0.1978934	0.01595302	0.03453755	0.02219688	0.04735202	0.03320702	0.01336333	0.06602823	0.09698853	0.1025594	0.01870456	0.06624377	0.3420534	0.1065883	0	0.03208537	0.07633955
7.323835	1.413524	0.1291435	1.752095	1.063759	2.702995	1.161297	1.39335	1.788841	4.604092	0.1793324	0.8691386	1.078647	0.8947683	1.972246	1.341397	0.8145406	2.821823
222.1418	207.9919	25.19855	26.07701	141.3439	71.23504	39.62036	30.26928	111.3831	402.7724	110.9538	74.00168	119.2314	57.9199	290.2779	83.69487	101.8541	115.4701
0.4893467	0.1295366	0.047979	0.1419589	0.4628522	0.2075147	0.07989658	0.1044952	0.1654843	1.45264	0.08019681	0.1350104	0.0996147	0.2339861	0.8975858	0.1424661	0.1543958	0.2825756
13.18778	1.12572	1.384697	3.464856	5.584508	3.082562	3.863977	2.237882	14.06831	30.83717	3.938167	4.010289	7.47303	4.473706	8.791991	5.774875	1.398535	10.8146
0.1225238	0.09428173	0.03768554	0.05439164	0.04993839	0.08674768	0.06723803	0.08568447	0.08912992	0.05236899	0.1176761	0.06312209	0.04471044	0.04526762	0.06680189	0.09090018	0.03031789	0.122866
2.469007	1.711215	0.943439	1.415703	2.119755	1.724649	1.061916	1.141528	1.574323	1.817236	0.2464597	1.896277	1.341388	2.097777	1.712375	1.544995	1.091505	3.058251
9.849381	5.399773	3.160379	2.672468	9.466084	9.76162	2.843056	2.896383	8.675786	18.31809	9.090972	3.959327	6.456196	7.537334	8.599276	3.469376	5.693361	17.01934
2.52877	2.116593	1.074866	1.312278	1.52629	1.919881	2.288471	3.407263	1.929838	2.837713	0.5348262	1.763065	2.030761	3.652284	1.52486	1.506666	1.785719	2.682874
0.653456	0.7826622	0.06371836	0.4828157	0.906273	0.9816744	0.07368501	0.2935615	0.6153586	2.104789	0.0136545	0.2428022	0.1574917	0.4732604	0.6588635	0.08656283	0.3588285	0.8052746
13.06273	10.68968	1.361358	3.379791	10.31673	6.302639	2.887202	2.34528	11.90697	21.30043	8.190504	5.461082	6.059263	5.154649	17.71753	4.31701	6.529917	6.574351
8.833122	7.814846	2.86324	2.565157	6.268408	9.439221	4.005299	3.957192	6.660565	14.89966	3.592574	4.694517	5.437206	11.02515	6.736064	4.762992	4.704264	14.17853
0.1413278	0.2593719	0.3353339	0.1935953	0.02488429	0.04550143	0.7445489	0.1966289	0.05551678	0.5626834	0.008623231	0.2555615	0.2665548	0.02255685	0.07681701	0.1991442	0.0161865	0.08887372
0.3808274	0.5541366	0.1441921	0.2462669	0.6553833	0.509516	0.1434017	0.07247104	0.4774394	0.3039003	0.228657	0.3634834	0.2936714	0.8204028	0.541341	0.1427185	0.1778698	0.6935371
0.01404954	0.1418954	0.0277799	0.01202844	0.02576846	0.03769453	0.09637546	0.06981099	0.061322	0.02702267	0.07143701	0.03908559	0.06591662	0.01401499	0.07424333	0.03558307	0.004469767	0.03681261
0.3723572	0.9523792	0.09035859	0.3622634	1.117548	0.7492681	0.139323	0.3027619	1.01115	2.002066	0.4001762	0.2295442	0.3454293	0.3545579	0.9199557	0.3098082	0.6300076	1.049564
10.26887	1.1112	1.163513	3.543821	5.433373	7.124782	2.022218	2.603812	4.587493	12.12938	0.7993377	3.768251	4.50999	4.81968	4.518435	2.908944	3.51016	14.91446
1.20828	0.3105279	0.08959161	0.3448209	0.4640008	0.7597924	0.1813092	0.262668	0.1854062	1.261852	0	0.3326399	0.3321633	0.5398778	0.4133166	0.3129743	0.2342475	1.615946
4.189875	3.735112	1.134759	1.317362	3.317965	3.755242	0.9756331	1.126197	2.89288	4.874239	4.503984	1.532944	2.560874	3.33951	4.391303	1.12029	1.607515	5.36657
1.329395	1.617897	1.79222	0.646679	1.111468	1.928119	1.549086	1.599941	1.617798	2.68354	1.165357	1.203767	2.010877	1.618908	1.632445	1.493658	1.042241	2.318418
5.557105	0.3432441	0.564475	2.031548	3.846472	3.504401	1.215154	1.01843	2.645828	4.51414	1.274774	2.774614	1.845962	3.237223	2.914951	1.272469	1.240092	7.533433
1.800355	2.561108	1.274037	1.009549	1.697373	1.472395	2.140645	1.668839	3.567121	4.442376	0.401499	1.288752	2.096875	2.510098	2.384408	1.858073	2.095136	1.710363
0.7216219	0.275216	0.2933524	0.432037	0.3776246	0.6549541	0.2824673	0.2958812	0.4625377	0.8589801	0.1462547	0.2948136	0.3799982	0.3372712	0.6685719	0.2753662	0.5538799	1.154751
12.09383	7.773448	0.9236751	4.073492	6.802003	7.36769	2.22176	1.947228	6.506207	12.96582	1.345983	4.076249	4.115548	3.978218	7.106944	3.223727	2.671008	13.81168
4.458856	1.855751	1.090944	2.465633	2.821103	3.590682	2.261119	1.768929	1.805163	4.892299	0.3472925	2.539707	1.972671	3.306424	4.741044	2.983886	1.919001	6.117874
2.553073	1.092206	0.9222733	0.6764707	2.103676	3.004634	0.7098562	0.8105534	1.182015	2.181555	0.570235	1.418158	1.348309	1.669613	2.073264	0.9066864	1.297427	5.813946
1.743069	0.6235318	0.1452673	0.5734947	1.077993	1.507336	0.2193747	0.522533	1.096396	2.493663	0.1867801	0.6913099	0.6386954	1.516046	0.7095892	0.3830891	0.5568372	1.82436
1.360178	0.6077341	1.218063	0.5033688	1.342322	1.756971	0.8957184	0.540689	1.04851	2.329214	0.4818913	0.6896633	1.09928	1.129236	1.838334	0.8849667	1.222816	2.628885
0.07439112	0.1395316	0.02206383	0.06368958	0.03411046	0.09979466	0.05613299	0.01232144	0.07102698	0.1430827	0	0.03018092	0.08289294	0.04328812	0.07721846	0.04282031	0.04733408	0.03790104
0.1201929	0.3761776	0.03290612	0	0.08139604	0.2511579	0.05327448	0.03675253	0.1210629	0.8402404	0.01410321	0.1286057	0.05856015	0.09222876	0.3140837	0.07024868	0.05294573	0.2584038
0	0.150163	0	0	0	0	0	0	0.04055935	0.03574645	0	0	0.01743932	0.02471931	0	0	0	0
0.8419696	0.1172324	0.01397045	0.2772493	0.0647945	0.06516293	0.1550944	0.05266167	0.3469352	0.1528834	0	0.05323513	0.08287327	0.01762027	0.1600148	0.06100446	0.07305465	0.1337048
20.96408	28.44797	7.166846	11.50617	26.28224	31.58963	6.832499	8.653428	49.21613	41.61353	26.10642	20.04978	4.108098	47.27084	16.76671	7.253683	5.238183	42.59073
5.511495	5.107128	3.350321	4.361819	5.377256	5.595804	2.744591	3.299193	8.350604	10.83223	15.84984	4.692823	6.240413	6.336612	8.283435	3.682883	6.981777	6.583591
16.50666	4.183217	1.988623	3.701756	7.908538	10.22714	3.160488	3.072248	7.085755	15.89373	0.948067	5.058074	6.519371	5.842972	7.91539	4.443591	6.401931	28.94429
5.23657	1.194172	1.040714	2.023775	3.780378	4.150194	0.9040025	1.018177	2.893541	3.274668	0.194078	2.221535	2.346895	4.201667	2.282418	1.327492	1.604198	8.790477
88.20421	90.24687	14.75403	30.28009	46.31004	50.96064	20.22565	20.14585	73.20829	184.6869	112.2425	23.68833	43.64947	36.80122	48.3526	29.04762	36.69275	99.69052
1.247394	1.424012	1.25486	1.017988	1.926622	0.8733774	1.783404	1.105526	2.352119	1.378498	0.7065839	1.519494	0.8701644	1.509929	2.217892	0.8036707	0.5360957	0.9907207
1.609005	0.4852425	0.2009341	0.5558502	1.091685	0.835794	0.6904521	0.4247977	0.7260437	1.506648	0.1414799	0.7684749	0.9024762	0.9292388	0.6895252	0.551518	0.4041271	1.944182
1.213477	1.297662	0.779095	0.83113	1.696736	1.551258	0.5875819	0.5249357	1.355271	1.791676	0.2830991	0.7943232	0.7736197	1.29594	1.212447	1.04527	1.730456	2.182047
1.345227	2.18745	0.0215667	2.427928	0.1600411	0.4572475	2.977848	0.9213523	1.725748	3.697519	0.9428123	0.1770054	3.697309	0.5258873	0.2470209	1.104985	0.104102	0.1905278
0.07838335	0.1102647	0.0232479	0.0209711	0.03773806	0.01971566	0.01613056	0	0.04811058	0.03533469	0	0.03747927	0.08963989	0.04398222	0.06656926	0.03383871	0.004675714	0.03850877
198.2371	193.3419	10.40735	24.61088	116.8046	26.9526	26.53241	17.26542	76.5391	441.1584	37.61622	57.92159	54.55766	35.24575	216.8092	51.9688	42.62702	37.67283
0.1487782	0.3194114	0.007060235	0.4993128	0.006549031	0.08382524	0.4065959	0.07096958	0.06818405	0.3605587	0.009077824	0.02897289	0.5151438	0.04155547	0.02021665	0.1233191	0.01135987	0.03638402
1.540716	0.6423271	0.150164	0.280257	0.8213375	1.018787	0.3736516	0.303625	0.7929692	1.448115	0.1065247	0.4310531	0.9798568	0.8098258	1.200999	0.5245743	0.9206281	1.017823
1.362243	0.6157264	0.3432475	0.3509155	0.7827192	1.782958	0.2083921	0.4073305	0.927384	1.686846	0.3861696	0.5785221	0.8993034	1.274715	0.7508057	0.2747895	0.5522829	1.621475
5.133661	2.864518	0.9531422	1.422306	2.592446	4.123821	1.104098	1.529171	1.994777	9.772515	0.8620181	1.699984	2.956413	3.280453	2.390045	1.11929	2.098952	5.738475
52.34308	28.66073	10.97961	16.38204	39.85094	44.54863	13.94182	14.0063	31.25288	132.0498	32.47887	23.8115	29.54333	28.27943	42.19346	17.68757	23.63181	84.30072
0	0.09301688	0.05098966	0	0.02364885	0	0.0176896	0	0	0	0	0.02989213	0.01512364	0.04287391	0	0.0148437	0.02051051	0.01876919
0.4437252	0.3566883	0.1065378	0.126631	0.4069214	0.3348288	0.1913256	0.1627377	0.432305	1.417344	0.08863596	0.1836959	0.4014973	0.1686225	0.4964797	0.2408178	0.2319192	0.4290729
0.4043841	0.1985465	0.0250615	0.170522	0.04649379	0.06376113	0.1093019	0.07497577	0.07409109	0.08706561	0.01380998	0.04617495	0.5309496	0.03913482	0.09568324	0.2605609	0.008640813	0.06589349
13.48342	7.779288	3.346966	4.806771	8.435621	11.00445	4.056623	3.553135	6.165517	14.22944	2.873006	5.586635	6.144455	6.671398	7.482745	5.261722	6.337375	21.87648
1.589992	1.464663	0.7980573	0.700577	1.253016	1.69347	1.129039	0.9289641	1.582435	2.024775	0.6218888	0.8911472	1.629271	1.504745	1.579524	1.389921	1.481404	2.044898
0.1065568	0.1671933	0.007900984	0.05131581	0.08794685	0.03350262	0.0328926	0.0132368	0.05450255	0.01921404	0.01015883	0.04631865	0.1546676	0.01993028	0.09803774	0.09660299	0.03178158	0.05235005
25.06038	10.44483	3.80357	13.95164	19.20659	23.97298	5.809161	8.257539	26.51723	48.38319	6.25059	12.93021	8.117317	15.10145	21.11674	8.753288	9.161771	35.64007
10.69594	6.418384	4.259357	3.273887	7.29486	5.352452	5.271925	6.708445	8.738051	12.61049	9.526907	4.948485	5.132296	8.300683	7.156955	3.003004	4.36361	15.60512
0.4160592	0.1556889	0.6814862	0.1654639	0.3781039	0.2214546	1.062806	0.4748267	0.3734461	0.2865383	0.4872512	0.4984588	0.4854904	0.4337798	1.352608	0.4839204	0.1716494	0.5696958
6.370626	9.356209	0.4512188	8.818947	1.778828	1.466869	9.496708	2.771791	17.17116	23.27187	4.544607	0.7053921	13.82935	2.655805	0.9331425	3.130632	0.06050066	0.9411921
5.300693	9.927132	3.119108	4.218334	11.66649	2.238025	5.35574	2.930585	11.46914	4.502958	0.5223514	6.454028	4.92741	6.428191	7.886696	2.926352	3.427683	3.289927
1.070819	2.836508	0.8898563	0.5787944	2.052702	1.156804	1.173924	0.9513709	6.146482	2.34908	0.1656005	4.252284	1.739876	4.110302	3.252131	0.8180458	0.5745931	2.228231
1.632841	1.193643	0.2295884	0.6627311	1.504064	0.4745939	0.3683812	0.1442391	0.8512635	1.099203	0.3689967	1.219755	1.259783	0.3981574	1.835285	0.7686132	0.196247	0.5281939
0.01159071	0.018061	0.03300206	0.00396933	0.06377605	0.04897863	0.03625594	0.02073357	0.02276547	0.01672004	0.003536082	0.06287805	0.01305131	0.0393115	0.06037489	0.01120851	0	0.04859196
150.8505	114.3542	23.82508	42.42056	82.74686	101.3152	27.11948	35.66757	82.80287	226.3025	30.2574	38.23352	62.94357	68.69423	63.36672	44.5668	82.81381	195.6432
0.5777197	0.1250307	0.1713472	0.1108613	0.4603797	0.5862631	0.04509595	0.1534308	0.1793359	0.3951385	0.03798496	0.3740912	0.3750309	0.5266182	0.5639593	0.2889671	0.1093279	0.4741317
3.897403	1.903237	2.230371	2.149632	4.502045	2.878086	1.197933	2.277973	1.809462	5.054534	0.304876	2.063379	1.890094	2.610572	2.616864	1.350346	2.563323	5.095079
0.08331579	0.3760812	0.007060235	0.1069956	0.02619612	0.02993759	0.2498361	0.02957066	0.1266275	0.4120671	0.05446694	0.02483391	0.05025793	0.06530145	0.05391106	0.04110638	0.005679935	0.02598858
0.2857471	0.713558	0.4041942	0.2822781	0.3507392	0.2764369	0.3437778	0.1310634	0.23385	0.3329318	0.4191129	0.2751729	0.3944598	0.3617873	0.5226927	0.311245	0.5664302	0.1631814
0.3254686	0.2132185	0.5009201	0.1054345	0.1548835	0.1681545	0.1303363	0.0961594	0.2879538	0.01268922	0.04025419	0.3181305	0.117621	0.03509928	0.189256	0.06683593	0.01679119	0.06146256
0	0.007864617	0	0.02333386	0.0199952	0.009140408	0.01495663	0.009028379	0.05947899	0	0	0	0.09590327	0.009062518	0.01028743	0	0.01734172	0
0.02552159	0.09665967	0.01703139	0.02048455	0	0.004814559	0.03413869	0.06023697	0.07571314	0.0299129	0	0.009984466	0.1493013	0.03659708	0.02167493	0.1498431	0.01674652	0.03622217
0.01151626	0.6629679	0	0	0.01267328	0.03476003	0.004739876	0	0.01884937	0.09967587	0.03513369	0.004004755	0.008104672	0.00574397	0.01304067	0.03181862	0	0.005029151
0.4159186	0.243673	0.01495252	0.08992087	0.1340753	0.1183528	0.1348725	0.0876767	0.1306509	0.7454281	0.05126791	0.04090683	0.224704	0.06705389	0.1379621	0.1450952	0.03608775	0.6678176
0.1333255	1.577376	0.08787405	0.006341443	0.057058	0.007452264	0	0.02208278	0.1091111	0.07479391	0.8473923	0.005151519	0.0417018	0.155164	0.1761362	0.05116238	0	0.03881551
0.005538406	0.02397255	0.006570594	0.01896671	0	0.01114454	0.004559009	0.01100795	0.0181301	0.03994682	0.00844826	0	0.05846556	0	0.03135764	0.01912779	0	0
0.1163347	0.04229783	0.01656191	0.01991988	0.1203414	0.04681842	0.03638971	0.02081007	0.1789875	0.1308975	0.04968781	0.0534008	0.08678392	0.03249362	0.1211953	0.03374959	0.019986	0.06096406
0.8820171	0.8150235	0.3174466	0.4977666	0.6798062	0.5284579	0.4677135	0.3184412	1.503128	2.782954	0.7709739	0.3400338	0.9462029	0.474525	0.5798105	0.5225299	0.5990506	3.017941
0.5963418	0.5736041	0.1257745	0.3460429	0.8239653	0.3799926	0.1745374	0.2633931	0.5097252	0.5543808	0.3032191	0.373278	0.6248585	0.4098031	0.8253422	0.2883389	0.3541481	0.4803349
4.549358	4.016552	0.5361475	2.373058	5.139047	4.577176	1.636827	1.167695	2.514947	12.25603	7.353187	2.786887	2.035488	1.953518	3.172815	2.642937	2.24291	5.736447
3.079988	3.723159	0.3014369	0.4509817	1.029787	2.163563	0.9947476	0.8252568	2.028655	15.00072	0.397031	1.168037	0.9943782	2.200764	0.9824473	0.5950006	0.6801968	3.566904
0.2321631	0.07464967	0.09443352	0.09086411	0.1167946	0.05339032	0.1037443	0.1318399	0.09771317	0.1530991	0.0708281	0.0876543	0.1353777	0.0661692	0.135203	0.02290894	0.1899286	0.06952158
5.665098	3.754661	0.6851401	1.614473	4.280112	4.241206	1.435856	1.304008	3.973893	19.13818	1.312408	2.603932	2.001727	2.641392	5.663164	2.168198	2.722218	5.846892
2.449336	0.1459795	0.1875527	0.332053	0.8188319	0.8080063	0.4372487	0.9971051	1.362777	6.631708	1.006397	0.3327842	0.8233024	1.112318	1.248339	0.3494327	1.368029	2.034314
126.05	101.6932	18.87737	39.98849	67.90517	84.53951	22.69831	28.8107	104.5311	238.466	56.69837	32.36578	49.03706	49.61235	47.71641	40.24721	59.73322	153.4306
0.1364906	0.09319484	0.04105223	0.08558448	0.04865751	0.1392593	0.03639635	0.04967174	0.07236985	0.1053793	0.03225667	0.02139237	0.162349	0.1093075	0.0631293	0.04249172	0.1412795	0.2619282
0.5309366	0.7490159	0.1399749	0.3367102	0.3245995	0.1187074	0.1780563	0.2345049	0.2896723	0.7658965	0.2999586	0.4786758	0.761142	0.1373118	0.4008114	0.2988213	0.1313775	0.188923

TCGA-DU-5854-01	TCGA-DB-A75P-01	TCGA-CS-6186-01	TCGA-QH-A6XC-01	TCGA-TQ-A7RQ-01	TCGA-HT-7469-01	TCGA-DB-A4XB-01	TCGA-DU-7010-01	TCGA-P5-A735-01	TCGA-HT-7479-01	TCGA-FG-8182-01	TCGA-27-1835-01	TCGA-S9-A6TZ-01	TCGA-DB-A4XD-01	TCGA-P5-A737-01	TCGA-DU-8163-01	TCGA-S9-A7QZ-01	TCGA-S9-A6WI-01
0.1720691	0.1473288	0.006841827	0.09142338	0.02835185	0.01244984	0.03238973	0.07178286	0.01887253	0.1089176	0.09851109	0.3352214	0.3161547	0.01618707	0.04635154	0.03589258	0.033906	0.04715051
0.2295694	0	0.04564079	0	0.08729118	0.3654243	0	0.2189041	0	0.06054773	0.2564498	0.1064863	0.3059719	0.04319259	0	0.110508	0.0695944	0.08578198
1.940899	0.6038932	1.133874	1.508472	0.2492659	0.7470467	0.3470591	0.6407228	0.5464462	0.866652	0.787233	3.873203	0.7847908	0.3546007	0.3035153	0.8415015	0.327907	0.3102779
0.1098794	0	0.07645808	0.1287813	0.0104451	0.1709288	0.0904896	0.1178714	0	0.0253576	0.0613726	0.2166131	0.04184231	0.03101007	0.06937273	0.1719013	0.02914637	0.06843007
30.54121	19.46635	8.488419	16.47553	3.155117	7.717465	4.261477	9.075794	7.218767	15.81398	12.03145	14.65687	18.91473	7.902593	6.05949	10.37524	13.80784	7.708222
5.481244	1.490089	1.322756	1.524596	0.8710011	2.226921	1.719853	2.992173	1.605655	3.623539	3.070661	3.441154	3.061942	1.543694	0.9694069	3.845948	0.6928424	0.7184726
3.179328	3.702288	2.079867	4.181973	0.5363446	2.630836	1.475093	1.657919	2.690213	1.756782	0.984818	6.461067	2.674507	1.068928	1.530433	2.885733	1.03933	1.342081
5.257781	4.484542	6.967106	3.607622	0.6062592	1.442025	1.391218	1.726224	1.435437	3.127612	1.665151	9.144522	1.691819	1.643659	1.146452	3.086013	1.248656	1.042611
0.0579141	0.0206613	0.04029875	0.02111721	0.01712759	0.05866419	0.01589811	0.04832058	0	0.08019134	0.01886946	0.09402253	0.1543767	0.01271236	0.03412665	0.03252451	0	0
5.480995	2.040992	2.475212	1.793616	0.3772271	1.92102	2.017687	1.807098	1.987691	4.474703	4.927428	1.008064	3.687622	2.436691	1.505728	6.215347	0.6610563	0.5083432
0.06791633	0.03876748	0.03150577	0.004952864	0.01205139	0.01834559	0.03728768	0.06422139	0	0.020898	0.01770269	0.05145511	0.03319044	0	0.01600824	0.0305134	0.02882455	0.01973839
175.3646	45.1907	48.86794	81.28788	5.37882	40.16064	50.90145	62.83858	54.4296	88.14269	78.42971	16.61486	67.28141	31.27437	25.46615	147.901	11.61839	15.93395
1.194145	0.5112246	0.5025147	0.5722676	0.1412632	0.4924846	0.6275165	0.4863059	0.8687929	1.383153	1.258932	0.2584898	0.7995723	0.7564017	0.5662835	1.143266	0.2081542	0.1214682
0.10346	0.1233854	0.1974618	0.1002397	0.215056	0.1557029	0.4284496	0.2663644	0.7353175	0.120063	0.6067646	0.1919606	0.2127481	0.3854189	0.3866935	0.6009517	0.6021908	0.04896852
2.665106	1.064325	1.354131	1.148058	0.2388978	1.072411	1.572401	1.018631	1.503494	2.084513	2.634927	0.5861731	1.390651	1.055822	1.027734	3.557075	0.3376432	0.3281408
0.06782733	0.0781779	0.2831804	0.1027324	0.07869432	0.07927593	0.1031229	0.1567154	0.05608087	0.3106511	0.3926888	0.135528	0.1390781	0.02061465	0.03689368	0.1816684	0.04428735	0.1091771
0.07494766	0.02673816	0.01738377	0.07515243	0.08866046	0.05061224	0.01028701	0.04689939	0.0479515	0.103777	0.1648303	0.07097779	0.07075599	0.0164513	0.0441639	0.08418108	0.00662682	0.1905916
5.12071	1.142865	2.195322	2.56182	0.979698	2.650876	2.065241	2.382588	0.8819422	2.057311	4.337101	1.556297	3.606534	3.430644	0.5672608	5.92421	0.6737898	0.6171537
473.7577	341.0257	219.6977	412.3836	23.09126	96.0663	197.1917	209.9841	49.41028	184.2126	176.1478	173.544	133.7893	44.9232	83.90698	213.7329	38.03695	60.62837
1.059411	0.4623892	0.6535253	0.2835553	0.0933268	0.977996	0.6063924	0.7648088	0.1096034	0.3814695	0.4222887	0.4818256	0.46816	0.3364476	0.1903807	1.06756	0.0558048	0.06223399
30.02828	6.817442	9.660144	12.1825	0.7423737	12.98819	12.01529	27.38937	7.509906	9.350372	7.228384	9.509005	11.86678	2.584274	5.527816	15.33499	1.42549	2.743476
0.10117	0.06316309	0.05866484	0.09222405	0.05984031	0.04270015	0.04165858	0.0562741	0.06472857	0.1361948	0.1606948	0.02737463	0.1376494	0.1165878	0.03974386	0.1088993	0.09392654	0.1359882
5.060795	1.508469	1.413164	2.496052	0.7511171	1.624846	1.378948	1.909699	1.004678	2.46355	2.778218	2.223452	2.144168	1.244703	1.158032	4.103847	0.8037078	1.770696
14.44776	14.86728	8.944073	11.70788	2.155107	5.76681	6.092903	5.738015	12.8602	11.65512	12.45013	7.837731	8.521913	5.705076	7.538429	17.54069	3.473978	3.640049
3.326147	1.30488	1.155222	2.79031	2.066237	2.764868	1.908421	2.498613	1.057764	1.92631	2.386247	2.550232	2.85617	1.510817	1.545562	3.120823	3.235561	1.138394
0.6818538	0.4271823	0.4860309	0.3638404	0.07377522	0.3790344	0.219134	0.4023956	0.4213535	0.8213212	0.349496	0.2294953	0.4266832	0.3394942	0.372392	0.7751967	0.123519	0.01449994
25.81071	17.95952	11.23318	15.05572	2.272143	7.592236	9.220152	12.67497	3.95299	13.49111	11.57903	7.194294	9.408816	4.29993	5.499051	13.62179	2.845296	2.817377
11.78704	10.25933	5.511847	6.242082	3.377855	7.384065	7.97962	7.320368	7.965602	11.60524	12.43841	3.573821	9.781838	8.65602	4.327422	13.41145	3.393439	3.468909
0.1943002	0.08430578	0.02923268	0.1723321	0.03261389	0.1329843	0.06054552	0.1007737	0.008063559	0.1066464	0.1026592	0.06820387	0.1434805	0.02074847	0.01856659	0.08847471	0.1727275	0.5677433
0.8731165	0.4591807	0.2042613	0.131737	0.08013617	0.1410505	0.3285281	0.2103812	0.2369318	0.9553638	1.103575	0.1771862	0.4689897	0.5606875	0.4967542	0.5410664	0.2116354	0.1246886
0.05220426	0.06052889	0.1029226	0.09993498	0.01543895	0.0176268	0.05015743	0.06170516	0.01336011	0.1124436	0.02126136	0.0565018	0.0434864	0.02864758	0.02050804	0.09283992	0.06000624	0.07206712
1.792362	0.2944785	0.8095803	0.5589563	0.04882274	0.9555666	0.556765	0.6821379	0.5431988	0.8562979	0.61472	0.4849781	0.3509969	0.3209563	0.3613225	0.9447811	0.08341023	0.1233736
16.78717	3.828533	6.177729	5.793799	1.137157	6.50056	4.898825	5.373019	6.157961	13.3032	12.31416	1.776311	6.166411	4.952342	3.157882	13.07684	1.407311	2.550383
1.344553	0.2752944	0.3172868	0.3261323	0.07261249	0.3256879	0.5873439	0.326793	0.403941	1.133239	1.028535	0.2182858	1.090801	0.7006237	0.2618021	1.483935	0.1054453	0.1274233
4.741998	2.832208	2.459621	2.778492	1.02824	1.976151	2.354258	2.763897	4.701046	5.411535	5.494135	1.724726	3.501195	2.556638	2.913797	6.476057	0.9734908	0.8757337
3.436118	4.123873	3.280967	3.521894	0.7968351	1.364631	1.333986	1.515421	2.277951	2.309531	2.813897	4.940565	2.150902	1.668659	1.299449	2.954252	1.246474	2.579898
6.080144	1.534367	2.09765	4.035233	0.7591441	1.859721	1.987953	3.493867	4.176483	6.104314	5.793516	1.315521	3.542048	1.604523	2.938368	6.740103	0.7515406	1.615551
2.674874	1.753297	1.306635	2.530175	2.143266	1.505838	0.5557475	1.24033	0.5706698	1.214284	1.845014	1.937106	2.176857	1.577885	1.146855	1.796057	3.31547	1.70117
0.7687788	0.2458376	0.476231	0.4461186	0.237064	0.7360003	0.6640006	0.9112999	0.6046335	0.6966832	0.9713815	0.3881283	0.6653875	0.60503	0.2486048	0.8529607	0.2387411	0.2288782
15.4171	3.818725	5.558203	6.421471	1.507941	5.881219	5.731142	6.50705	5.386174	9.903163	11.8608	4.872804	11.11746	6.450713	2.812785	15.19985	1.381288	1.9478
7.594535	5.403139	3.191756	3.890893	2.266325	4.97743	2.456509	3.410019	2.349181	5.200243	5.321157	4.378901	3.644305	2.264507	2.59609	6.750093	2.319783	1.853557
4.482816	1.769625	1.779323	1.808673	0.5251665	1.443007	2.014869	1.586965	2.599472	4.291111	4.308461	0.4740803	2.479829	1.595527	1.571914	5.261139	0.4521939	1.097544
2.495697	0.4583082	1.331546	1.046629	0.3443057	1.335174	0.6722416	1.032768	0.6986301	0.8770479	1.615386	0.5322635	1.145918	0.9340759	0.7096839	1.766068	0.2413739	0.2508478
1.744463	1.535519	1.429407	1.747743	0.5641256	0.7761839	1.288937	0.8025607	1.514568	2.144593	1.56171	1.244009	1.423268	0.8051978	1.527512	1.620607	0.8130256	1.329072
0.1900368	0.05547035	0.04808528	0.07559246	0.1021849	0.05833275	0.1138197	0.05285235	0.03537033	0.02126354	0.04503085	0.06544393	0.08058988	0.06067461	0.02036031	0.09702227	0.1833046	0.03514638
0.1631823	0.1378813	0.107572	0.03757967	0.02285988	0.05219867	0.1273135	0.08599012	0.211006	0.7373171	0.09234397	0.04183004	0.1545328	0.02262261	0.2125583	0.3762188	0.03645086	0.01497643
0.1841532	0	0.03203514	0	0	0.02331729	0	0	0	0.02124914	0	0	0.03068011	0	0	0.07756523	0	0.02007004
0.393801	0.0819534	0.02283509	0.2632513	0.0291158	0.1662089	0.1081031	0.1825375	0.01679692	0.1009779	0.7912294	0.02663871	0.2587856	0.2233054	0.07090492	0.215015	0.09865556	0.02384367
26.03166	19.38701	23.69207	11.57752	9.677905	16.45086	21.67538	27.85206	26.43139	75.998	36.7056	11.5087	23.49429	17.91325	28.02511	44.35382	3.966254	10.54724
8.383929	5.522454	6.465534	6.016705	4.506878	22.40104	8.32274	6.081748	4.632887	6.02011	7.672938	10.23004	6.04081	5.600034	4.940342	7.842347	5.573924	2.758641
30.51966	7.468588	9.833124	10.82673	1.689727	7.607846	10.9708	5.953638	10.98382	15.20889	18.33477	3.765512	14.08749	7.320766	7.419865	25.52936	2.169253	3.469208
6.948279	7.023784	3.696487	2.354364	0.8223249	2.659042	4.251916	3.139983	4.240942	10.28997	7.618536	1.343146	5.853238	2.588835	2.734464	10.70383	0.409208	1.583674
82.99079	40.18039	91.27793	76.59704	15.40406	52.32796	39.22134	75.42772	75.77543	101.5499	97.86841	22.91824	93.04191	61.36954	43.6306	102.6806	19.59788	17.24537
6.654308	4.311594	4.36626	6.337691	0.931872	0.8030933	0.7533689	1.820519	0.5410658	1.579446	1.056449	1.490659	1.688887	0.5800936	1.134016	2.602999	0.8879463	2.073755
1.629513	0.6775632	1.068709	0.4179671	0.2658836	3.043208	1.789283	1.228312	0.3911404	2.593472	2.460561	1.070357	1.759929	1.011383	0.5209425	3.311308	0.2384778	0.2906814
1.830104	1.121291	1.347276	0.986459	1.09424	1.773206	1.092142	0.8077321	0.9570834	1.566962	1.335151	1.11956	1.14005	1.187678	0.9377503	1.444857	1.111609	0.7400063
2.093965	0.3614701	0.8225324	0.2586124	0.194771	1.95003	0.2225104	1.676651	0.1815102	1.449715	1.370005	1.973912	2.52077	1.023055	0.1194094	0.6828207	0.322514	0.3239137
0.2366416	0.1558592	0	0.009956146	0.004037578	0.01843896	0.1723964	0.1139087	0.02096353	0.07561573	0.02224099	0.08126958	0.1122088	0.02397403	0.02681619	0.3475787	0.01931417	0.007935542
388.6833	180.0297	253.4674	221.4153	11.53971	90.18153	168.7585	195.801	17.13906	99.06527	95.81676	112.2805	110.1582	20.99288	36.05339	136.4146	25.06595	30.35239
0.3703889	0.07691679	0.08462781	0.04233063	0.05885693	0.2239917	0.08194797	0.1937226	0.02546594	0.1122685	0.1783173	0.116674	0.1731484	0.05824605	0.02606049	0.3725554	0.02932794	0.03855955
2.063714	1.284453	1.309052	1.166436	0.4532475	0.5872953	1.449119	0.9471911	0.4856033	1.343625	1.926036	0.6648148	1.67788	0.2883492	0.5733929	1.94909	0.2796233	0.9403125
2.430076	0.647215	0.5610478	1.212743	0.1887761	0.7486742	0.940682	0.7755077	1.496011	2.046809	1.817041	0.4363339	0.8582138	0.5162041	0.5147118	1.509377	0.2732853	0.8201594
2.725945	2.098438	2.138864	3.618386	1.004444	3.735055	3.260593	3.899707	4.826713	5.272048	5.903907	2.084411	3.987203	3.240281	1.982717	6.826837	1.201216	1.384115
74.6579	38.36852	42.38344	47.61547	7.826949	27.13084	26.74669	30.31713	58.58478	74.65742	71.10728	20.28205	42.48792	29.45344	32.66928	79.35359	10.35391	15.18729
0.03992512	0	0.02778135	0	0.4427814	0	0	0	0	0.05528271	0.01951249	0	0.02660625	0.02629114	0.07057921	0.08968764	0.06354272	0
0.8930794	0.4359046	0.9150834	0.9930336	0.04788996	0.263441	0.4283582	0.2374622	0.5123691	0.4212632	0.3453406	0.3266255	0.3171961	0.2197308	0.3469839	0.5621799	0.07809771	0.1240725
0.7625038	0.1950209	0.05071702	0.1073284	0.04228197	0.1363023	0.1339009	0.3648762	0.02582734	0.1785559	0.1315257	0.1365344	0.5903338	0.0553806	0.03634169	0.8092127	0.1011301	0.07088102
18.45231	7.149	10.98269	8.016339	4.462815	6.484507	7.045538	7.775154	9.5338	17.89231	16.13411	6.493292	12.71416	8.889946	6.50474	18.20256	4.058859	3.968744
3.370576	13.05163	1.291271	3.050832	1.012819	2.468878	0.9802148	1.494142	1.212898	1.742984	2.136456	1.857693	1.483622	1.378765	0.9532277	2.591741	1.383444	1.344373
0.3464442	0.10594	0.008609589	0.02706941	0.03842168	0.0438664	0.07132729	0.1548511	0	0.05139722	0.06047023	0.2310046	0.2226263	0.04073882	0.02187287	0.2709981	0.06564075	0.02696957
50.52385	12.071	21.97424	16.17351	3.549177	18.51127	10.24604	22.70199	17.29119	40.7338	34.95	16.79401	28.0088	17.43018	12.9804	38.30065	4.057484	5.208675
7.582129	6.516127	4.532579	14.5929	5.702191	6.387079	6.437273	3.304377	8.028395	7.520239	11.87657	2.749534	8.554385	6.554277	4.984771	8.369907	5.612063	9.404968
0.6333461	0.8166264	0.3400718	0.8264632	0.2831721	0.4874762	0.8871021	1.113035	0.2373856	0.2370809	0.3534042	0.3278989	0.6846081	0.1247913	0.320312	1.030651	0.3651025	2.000111
7.30166	1.228936	2.376485	0.7085429	1.436699	10.46805	0.5819205	4.249755	0.7233446	4.593128	4.2592	4.684288	7.828531	3.295962	0.624571	2.943177	1.280802	1.334849
12.9133	19.70414	9.479456	17.11076	3.876528	4.053052	2.823182	6.887416	1.610672	6.399423	2.667304	9.295746	10.60792	3.150746	4.42122	7.621198	4.393268	4.077684
3.868858	1.780908	2.436918	4.272218	1.033016	1.044748	1.019264	3.71753	0.8305727	3.715239	1.4786	1.733979	1.979488	0.7848325	1.210118	2.708211	0.539873	1.538721
5.134642	7.599861	0.6410841	0.7620073	0.5382956	0.4097181	1.091839	0.8905646	0.1552715	1.908375	0.7467913	3.155641	1.108136	0.192367	0.6753105	1.728904	0.453008	0.4800084
0.06460186	0.02996138	0.04495234	0.02120019	0.009552718	0.01090643	0.03192117	0.04491709	0.006613161	0.1510738	0.04841144	0.08040799	0.02870062	0.01701642	0.015227	0.09191022	0.02284821	0.04130526
155.1074	64.91023	88.0507	135.998	20.63829	90.25584	79.4777	91.21285	130.7407	140.3615	176.2009	31.87704	90.16911	97.32168	75.25485	176.1459	28.39436	22.64469
1.535964	0.7476781	0.7082278	0.2732816	0.04104651	0.3842781	0.9296413	0.7295461	0.2060138	0.9779811	0.8094546	0.2905656	0.5672812	0.1706606	0.2835214	1.517338	0.09817511	0.2218526
5.15214	1.670404	2.373882	2.424441	0.8699505	1.933567	1.519443	1.18117	2.788524	4.627435	5.892324	1.403487	3.321279	2.689742	2.885536	4.503075	0.8002881	0.8700856
0.0773947	0.01775003	0.01538687	0.0181417	0.009809488	0.1175956	0.00910533	0.05534932	0.008488648	0.01020622	0.03242133	0.02692477	0.1657804	0.02912302	0.006515122	0.01862777	0.08211822	0.009639889
0.3369042	0.3715061	0.1989105	0.3908715	0.4076037	0.1758045	0.1849684	0.161843	0.2037939	0.3015743	0.229518	0.4143621	0.3809938	0.174795	0.360955	0.470146	0.7690934	0.3204452
0.269653	4.433981	0.01137178	0.2502781	0.3407393	0	0.04037616	0.2795265	0.03764158	0.8825291	0.2875346	0.1724575	0.2069247	0.3120919	0.1637116	0.03671197	0.1473902	0.2849772
0	0	0	0	0.02994988	0.008548516	0	0	0	0.007790294	0.01649789	0	0.03374354	0	0	0.01895782	0.01790854	0
0.1200209	0.01110106	0.03299354	0.06807607	0.00657317	0.01350837	0.01220265	0.0296709	0.006825717	0.03009167	0.02027665	0.004811154	0.03554771	0.005854452	0.06985075	0.01165001	0.01886606	0.01421095
0.04279128	0.04007356	0.007443933	0.01170223	0.004745679	0.02709094	0.008810037	0.06248002	0.008213355	0.009875228	0.01045663	0.06078702	0.04633894	0.02817854	0.02521533	0.06608675	0	0.00466363
0.3902627	0.09606818	0.1140548	0.1451475	0.01731252	0.1620801	0.04499535	0.25398	0.1558066	0.259383	0.2899126	0.05068677	0.3771052	0.2518526	0.05059285	0.4690264	0.06625298	0.04083167
0.06192516	0.1104614	0.06702856	0.1128988	0.02441843	0.006969689	0.2719872	0.04592641	0.01056526	0.03810902	0.06052901	0.05585245	0.5135469	0.02718562	0.08108939	0.123652	0.03650253	0.005999064
0.02572401	0	0	0.02251138	0.00456459	0.02084574	0.01694772	0.004292561	0	0.004749201	0.005028808	0.02505749	0.003428516	0.006775821	0	0.04045043	0.005458798	0
0.1210351	0.1272274	0.02105516	0.04019256	0.04218704	0.006568001	0.09967672	0.06852588	0.009956342	0.03192237	0.08239184	0.1052669	0.05905335	0.01138614	0.02292477	0.04855228	0.03669201	0.04145765
2.105105	0.6929896	0.9694217	2.108059	0.1715234	0.7180429	0.5559745	0.9242825	1.847103	0.9687856	0.5908977	0.3088794	0.4396687	0.2909882	0.7341505	1.926717	0.2604762	0.5083492
1.206404	1.561276	0.616746	0.4511126	0.2512046	0.274333	0.3852412	0.5443652	0.1323183	0.6363654	0.4452099	0.9593023	0.6480839	0.2594059	0.3119209	0.8780046	0.3134774	0.1126976
10.85897	4.942374	2.843264	3.55131	1.042892	2.749904	2.397027	5.884444	2.621454	6.820448	3.88456	4.952576	4.289655	2.58017	2.077959	4.998165	1.692623	1.390883
2.34875	1.478708	1.241782	0.9319911	0.8376325	1.457825	1.355892	1.244011	1.140306	5.733897	5.311839	1.813117	2.927107	2.054656	1.0923	2.489394	0.3787011	1.711551
0.1663691	0.1714656	0.1200533	0.07414386	0.3334817	0.06865788	0.07104271	0.07711664	0.05676956	0.09669629	0.1565953	0.2000721	0.2094193	0.1460746	0.09440428	0.1937865	0.2745912	0.06446872
11.32421	4.667546	5.66289	4.558959	0.6929039	4.306661	4.430024	5.422978	2.689816	7.987475	6.624214	2.316597	5.566847	2.427229	2.524664	7.169226	0.7318401	0.938657
3.324788	0.6706112	1.697663	2.73521	0.6792601	2.038957	0.6385632	1.091316	0.5171422	0.5675547	2.139757	1.071282	1.410553	1.71233	0.5399847	1.570294	0.419668	0.5206968
146.9299	48.16342	92.85476	117.6971	16.70095	91.84494	61.57147	91.63989	97.36892	125.7585	147.0814	25.63332	63.51334	73.94102	53.29755	135.8079	23.5529	21.10272
0.1696492	0.07836222	0.05716014	0.05078964	0.04277846	0.1628019	0.1205937	0.1370768	0.08500516	0.09066566	0.2094623	0.0376894	0.163037	0.09172466	0.07786985	0.3068853	0.07579081	0.0295829
0.8037398	1.094827	0.4067429	0.2797463	0.2431013	0.370068	0.6619085	0.2133726	0.02804905	0.4552799	0.3928094	1.067611	0.3164999	0.120289	0.06458377	0.6155176	0.174435	0.2388982

TCGA-S9-A7IY-01	TCGA-DU-A7TI-01	TCGA-TQ-A7RV-02	TCGA-S9-A7QX-01	TCGA-HT-A74H-01	TCGA-QH-A86X-01	TCGA-RY-A845-01	TCGA-HT-8111-01	TCGA-TQ-A7RN-01	TCGA-DU-A5TW-01	TCGA-R8-A6MO-01	TCGA-DU-8162-01	TCGA-DU-6394-01	TCGA-KT-A74X-01	TCGA-DU-8166-01	TCGA-DB-A75L-01	TCGA-P5-A780-01	TCGA-VM-A8CB-01
0.09398105	0.05127745	0.06019033	0.0203551	0.4979092	0.01454034	0.02770382	0.08679366	0.04393436	0.06073404	0.03494174	0.03889345	0.009499502	0.1456626	0.03580141	0.07966942	0.06012852	0.01438056
1.101883	0.2280428	0.1606082	0.03879595	0.2612398	0.09699639	0.2016087	0.1736962	0.1116493	0.08529424	0.05484496	0.2594523	0.1013916	0.3835632	1.343395	0.1714396	0.03208866	0.7674447
0.7215254	0.7949964	0.4958912	0.7284079	1.156281	0.2856354	0.3766086	0.713002	0.2849474	0.3897016	0.6656074	0.5186186	0.4885828	0.4490692	0.7949368	0.6755891	0.9071504	0.335572
0.1091166	1.214279	0.1537446	0.04178026	0.3885104	0.02901601	0.08845511	0.08313666	0.09017831	0.07144304	0.06562645	0.1517784	0.2790435	0.09485267	0.2822023	0.04923393	0.3839669	0.02295775
19.1972	10.56475	8.990783	10.237	30.41962	7.863874	3.677389	12.48838	7.065312	5.597967	16.85617	11.60374	10.73903	11.11679	9.039859	15.6892	14.95219	5.152772
2.248918	3.032191	1.653558	3.146151	3.838928	1.107518	1.423412	3.606811	0.9089631	3.14511	2.155415	1.859261	1.13241	1.588883	2.476456	2.657717	2.961704	1.183455
2.581022	4.437029	2.974189	2.377125	1.745271	1.734126	3.372147	1.976379	2.272404	2.998873	1.193488	1.44163	2.029018	2.011952	4.886309	1.66785	2.207129	0.9578203
2.589436	1.19336	1.533764	2.295919	5.367529	0.8982191	1.706522	2.835793	1.130839	5.621534	1.900592	1.697682	0.6711812	0.980479	0.8466309	2.25239	1.713266	0.846708
0.1789265	0.1006757	0	0.01141835	0.07688761	0.06423255	0.02966856	0.02556098	0.01643022	0.1506219	0.05649654	0.03393846	0.1044449	0.316091	0.228445	0.03027467	0.04722138	0.2258729
3.760315	3.024748	2.906828	3.187123	4.239522	0.968029	2.464942	5.274812	0.8777981	2.32928	1.490708	1.276635	1.241278	1.152087	1.49639	2.48009	2.310718	0.6033726
0.02622856	0.04197802	0.08315072	0.01071232	0.01030477	0.006695646	0.01391702	0.007993483	0.03853569	0.0117757	0.08707661	0.02387994	0.07698951	0.05648493	0.148375	0.009467559	0.0177206	0.0264883
67.06219	92.96808	73.97948	73.06628	105.7118	10.67869	44.82223	89.22018	17.57496	21.25563	23.69862	43.19216	20.9314	6.804176	68.40505	44.3666	49.55242	8.947312
0.9519786	0.8929481	0.8493555	1.062834	1.206818	0.357385	0.7894402	1.448128	0.3702367	0.8023083	0.6252509	0.4248698	0.2966659	0.2283362	0.574561	1.46548	0.5285634	0.1746499
0.2191826	0.6508894	0.1737151	0.1993195	0.1009139	0.1049121	0.1696034	0.3653051	0.535876	1.126279	0.1903207	0.05716449	0.2513178	0.3065391	0.5919739	0.4728475	0.1127989	0.4582701
2.031292	2.517698	1.296177	2.106637	3.941558	0.4841608	0.8245066	2.460582	0.489592	1.261332	0.82384	1.054342	0.6479967	0.4532936	1.868935	1.042891	1.446036	0.2953598
0.1873892	3.482835	0.4215966	0.06789292	0.1365572	0.1736015	0.1122594	0.03684465	0.07993078	0.03392385	0.06544001	0.2155551	0.06855454	0.2359495	1.913999	0.2400154	0.3063009	0.08393926
0.1085399	0.7817172	0.2752778	0.05171843	0.04264355	0.05541632	0.006399101	0.04410522	0.02126263	0.0243653	0.02088948	0.126271	0.04827284	0.04382774	0.3695434	0.03917899	0.1772191	0.0274037
3.092872	7.284446	5.293782	5.071924	4.271395	1.040771	4.148324	7.330077	1.631755	4.933703	3.094627	1.301298	0.7034042	0.8862015	3.486757	5.171875	2.991947	1.200891
242.8466	418.3016	261.1147	193.2313	369.0335	123.2393	77.61434	375.5961	77.00652	19.78079	149.6996	113.3758	88.26191	80.80553	146.9709	122.8163	156.8225	29.49685
0.5527648	0.4789134	0.2805672	1.062144	1.004635	0.08611057	0.3849083	0.8920529	0.2525937	0.2638048	0.3895181	0.1155799	0.2903629	0.2138296	0.6087101	0.5263142	0.5697476	0.1648343
10.97763	24.07867	15.45299	11.49456	16.64957	2.269961	6.037306	14.79292	4.403275	4.681834	6.661446	5.998032	7.537461	4.631177	13.36671	13.05441	17.69431	3.223174
0.07325773	0.07327925	0.05160984	0.09474684	0.1055331	0.04675322	0.09501791	0.10791	0.04664061	0.09318872	0.1057433	0.07781424	0.02606494	0.0755959	0.07290706	0.08373739	0.1031137	0.0184958
2.051331	9.944405	2.652963	3.159814	2.575253	1.366385	1.304696	3.078233	0.6147495	2.634843	1.088306	1.783128	2.024634	1.343954	9.081134	2.209109	3.290426	0.5007619
13.53168	12.7146	9.677592	12.5355	17.48898	4.591119	7.906902	13.35191	4.018428	6.935995	10.51929	4.466088	5.100326	6.830007	7.098594	9.72974	9.155181	4.100297
2.337519	5.317748	2.897148	2.867491	2.571443	1.41935	2.471742	3.167584	5.882541	2.77733	2.913752	1.19585	2.688864	2.128214	3.867305	3.902388	3.570302	1.121317
0.1734089	0.6938394	0.4581226	1.32795	0.7759192	0.4549762	0.1874313	1.145052	0.2689312	0.8109842	1.168093	0.2704445	0.347054	0.6548298	0.1059695	0.8693657	0.3742578	0.2918769
17.7945	21.15611	12.74684	13.12525	23.00889	5.710461	6.408596	22.20961	6.082422	4.236909	8.268408	5.887131	6.528834	6.011276	8.409374	9.686621	10.06893	3.186644
9.877539	26.61968	9.206465	13.20187	9.588873	2.875096	7.598176	14.43831	6.200377	9.039446	13.75222	4.044499	5.696964	4.807991	8.027633	11.45561	12.34948	4.915105
0.5901531	0.1582318	0.05143436	0.01863645	0.2270807	0.06212566	0.07532543	0.02781287	0.04916367	0.1434051	0.0702558	0.5400779	0.1014699	0.1187406	0.8221957	0.126277	0.04624338	0.2611328
0.723792	0.4928465	0.262634	0.4674594	0.7366105	0.3283566	0.3123283	0.7673956	0.1825741	0.3327874	0.4059426	0.122401	0.3025126	0.3755985	0.452203	0.2636235	0.614945	0.1431096
0.02016073	0.0403333	0.07456664	0.0102926	0.1287133	0.05789976	0.03120074	0.04224162	0.02962067	0.07354308	0.02546323	0.1147216	0.0336241	0.03391989	0.04752053	0.1000628	0.03830918	0.03181309
0.5555745	0.9292664	0.1636183	0.9764516	2.039629	0.5754476	0.4510463	0.5412635	0.1338139	0.3680164	0.8019394	0.2764077	0.2855715	1.244276	0.536695	0.3780715	0.5768832	0.1839594
7.212561	14.30515	6.626737	8.748549	10.108	2.024162	5.852183	7.478177	2.546597	5.331159	4.56691	4.447687	2.309178	2.023071	8.443394	5.887697	6.379604	1.972528
0.6637396	1.111074	0.6656178	0.6777143	0.6452784	0.1988326	0.5510361	1.640969	0.3632061	0.4409119	0.6110136	0.3494284	0.1174761	0.159532	0.5587464	0.5745173	0.4804686	0.179548
5.561063	5.685815	3.298952	3.793049	5.670938	3.47053	3.008123	5.583987	1.454901	3.993749	2.7053	1.443205	1.530241	1.927745	2.654986	4.40242	4.356287	1.864511
1.913842	2.123501	1.408838	2.096428	3.066068	0.8152664	1.980051	1.720072	0.9657259	1.887425	2.048421	2.319062	1.202389	1.000385	1.598589	2.724744	2.420517	0.879609
4.063743	6.172926	4.766095	3.425354	6.06132	1.101918	2.659021	3.68642	0.5015655	3.035883	0.97605	2.156757	0.7708196	1.210582	8.856451	2.808132	6.830625	1.334293
1.90927	2.958254	1.359044	2.499022	2.331606	1.956108	1.518101	1.985624	1.752178	2.581759	5.728551	1.762377	1.560961	3.847129	2.559523	2.305778	2.301424	1.644958
0.7060288	0.4672024	0.4476556	1.175612	0.8214924	0.3084801	0.6435828	0.7944781	0.3351329	0.6949221	0.6310651	0.416177	0.3840514	0.2704699	0.4181758	0.8919778	0.8393523	0.6581727
6.084261	10.50973	6.773865	8.175412	11.58167	3.948347	5.409389	11.42417	2.527175	5.240653	6.978113	5.121272	4.745261	7.988783	7.080948	5.914779	5.213026	2.332987
4.550547	7.87826	3.302246	4.168724	4.266423	1.697821	2.912651	5.275797	1.741576	2.521648	2.923649	3.812302	2.685484	1.586916	5.210496	3.336855	3.241074	1.799679
3.264317	3.361313	1.88904	3.164993	3.327485	0.8957577	1.74662	3.521656	0.7758267	2.026952	1.597014	1.717026	1.116297	0.7507444	2.051087	2.339272	2.428616	0.9407437
1.573623	2.845759	1.736644	2.5249	1.4771	0.5788266	0.7403702	2.734558	0.455568	1.444325	1.426639	0.6234305	0.982568	0.6834143	1.991618	1.643902	1.387888	0.4110015
2.587737	1.332037	2.205627	2.005773	2.70867	0.6027367	1.177631	2.266636	0.5966596	0.710226	1.438206	1.089158	0.5607432	1.480927	1.216934	0.9289656	0.9331888	1.096849
0.1267648	0.07341173	0.1057564	0.08174768	0.098297	0.07238561	0.06490207	0.06099976	0.09312312	0.1497709	0.1059344	0.09111615	0.08456746	0.05837095	0.1310506	0.1204145	0.112691	0.07580142
0.2388098	0.6171066	0.2418461	0.07111938	0.2736546	0.1651096	0.03519829	0.1137192	0.05847761	0.03350537	0.02872567	0.1283416	0.0796575	0.1071443	0.1329057	0.1077521	0.1176476	0.02512237
0.02666926	0	0	0.05446153	0.1047791	0.01702037	0	0.04063896	0.01959158	0	0	0	0.01779161	0.03589625	0	0	0.04504587	0
0.266143	0.0507088	0.4352607	0.1358733	0.441904	0.08088237	0.2689847	0.2607121	0.06517072	0.2062605	0.1829342	0.0721165	0.0422737	0.183376	0.2140859	0.08577499	0.05886709	0.03199745
22.60393	42.58012	36.1828	26.45765	28.54661	14.35927	16.03046	21.84962	11.50707	33.87475	18.22679	13.50772	9.537852	16.98907	61.37213	25.81298	59.6045	16.09854
5.676433	3.867916	3.669409	5.35375	7.603566	4.673987	5.443937	6.381306	6.493583	9.452781	7.903363	3.068731	8.265068	8.473095	5.426062	9.875055	6.988394	7.644179
17.78524	16.89254	12.17265	12.04536	18.76576	4.328077	6.870546	15.65764	3.258873	8.651876	7.814023	6.518462	4.456583	3.825414	9.767078	7.120657	11.15628	4.525434
4.734901	10.06372	3.122481	4.084016	5.294816	1.301638	2.83613	4.425703	0.9159048	2.620854	1.862078	2.980023	1.639473	1.333787	7.83674	3.563934	4.948234	1.401057
84.22063	74.04554	65.78101	119.7721	91.30905	41.20848	68.51384	89.11404	30.06523	87.35753	78.16015	26.87222	31.03974	58.66853	43.59576	106.3015	112.394	41.6249
3.501449	1.531352	1.563848	0.8697443	3.516264	1.052181	1.124731	2.264021	1.17941	1.317366	1.580834	1.763121	2.820351	1.49787	1.04553	1.253978	1.783525	0.9266466
1.762041	1.558519	1.064016	1.2851	1.172267	0.3323757	0.7829621	1.755845	0.5260567	1.003482	0.4667082	1.247978	0.7498842	0.3329684	2.339203	0.8420386	1.334152	0.2739363
2.135664	1.721328	2.029544	1.913934	1.99203	1.686571	1.766372	1.311106	1.230278	1.569321	1.973819	0.8976075	0.9224973	1.106395	2.088421	1.705402	1.102938	2.754204
7.39539	1.585192	1.591953	0.2397173	4.631163	1.198667	1.505252	0.6409725	0.445543	2.064189	0.4659641	1.588293	1.78811	14.32539	7.040258	5.579046	0.2974102	4.297423
0.1054482	0.04219168	0.1615763	0.01076684	0.2123229	0.0269189	0.09791498	0.1044442	0.01161955	0.01183563	0.03805209	0.04000248	0.2356614	0.04967587	0.1035626	0.004757873	0.0267162	0.01996734
153.5189	309.4967	186.4252	105.3015	286.2004	78.6144	48.24602	347.6559	58.89616	6.506551	80.31286	92.15247	100.1897	75.03491	152.0906	73.93075	101.6535	22.61376
0.9158845	0.3011134	0.3654844	0.08501528	0.08178088	0.1062762	0.09062443	0.09759697	0.06116553	0.2803635	0.03235728	0.3693141	0.1666379	0.0646553	0.9410227	0.1098151	0.02163609	0.242558
2.89357	5.774743	4.566794	1.031195	2.620628	0.77045	0.6854216	1.929051	0.3795811	0.7381311	0.4441133	1.350735	0.4637879	0.8092848	2.251318	1.23353	1.455906	0.2609129
1.310397	2.25818	1.124258	1.761904	1.771333	0.6541342	1.009683	1.986915	0.3049933	0.8737459	0.8240121	0.9548409	0.4847013	0.3754554	1.467905	0.9717697	1.17241	0.2620543
4.513796	5.988401	3.097359	5.484211	6.391135	2.11377	3.615897	5.996077	1.022759	3.051317	2.80818	1.923601	1.696271	2.140224	4.536713	4.159254	4.226648	1.554033
50.17043	68.09293	36.32332	57.00969	90.00876	19.26394	38.97539	70.97779	16.97729	32.83236	50.33558	22.48994	14.53443	28.69662	42.23182	49.19072	45.92563	17.09362
0.1156399	0.02313477	0.07332115	0	0.09086601	0.04428096	0.02045306	0	0.06796047	0	0	0.035095	0.1080041	0	0.01817153	0.02087091	0.01953223	0.08758879
0.983527	0.5573061	0.3784871	0.5282391	0.9381088	0.1378738	0.2463524	0.736361	0.1336438	0.350958	0.3897921	0.3191902	0.1327433	0.1721714	0.317141	0.3745128	0.2784725	0.100475
0.1721355	0.09746382	0.490796	0.1227002	0.2743449	0.29848	0.0832939	0.4553172	0.05964765	0.06197204	0.2953483	0.3893403	0.1928364	0.3344215	0.1582123	0.1436132	0.1920025	0.07790007
12.99959	14.51554	10.05221	16.04898	10.84062	5.402094	7.603299	14.67839	6.407461	8.348674	8.194983	6.119143	4.741545	4.453775	8.121706	11.95186	10.49252	11.67215
2.529188	1.369988	1.662577	1.836828	3.115243	0.9760802	1.729469	2.60277	0.8863944	1.150291	2.060607	1.666876	1.33602	1.1474	1.282919	2.203173	2.067154	0.700278
0.08600981	0.07886548	0.1211873	0.02927357	0.1478393	0.1052089	0.01901554	0.2948914	0.01053065	0.03217947	0.1241501	0.04894258	0.03825262	0.1784747	0.01689436	0.02587202	0.1089565	0.06333654
22.70361	34.61685	21.64021	27.53861	41.25041	7.987461	16.70139	33.89605	6.194396	23.14535	19.49482	11.55598	9.588868	14.99074	20.30849	26.54959	31.39889	7.886808
11.5113	18.85227	8.538553	6.750139	12.92736	6.447483	7.981995	6.025909	4.257744	5.104922	10.74804	7.435924	6.02137	4.476185	9.068086	7.600243	11.09828	4.58559
0.4593312	1.063419	1.059324	0.8554129	0.7859816	0.223086	0.2733589	0.7747707	0.1464322	0.1556404	0.3544435	1.078379	0.3469012	0.2158043	0.8488956	0.9176459	1.395529	0.2589267
25.58307	6.209983	8.651134	1.01004	14.00462	3.940289	3.800866	1.767266	1.703959	7.542406	2.240279	5.693663	7.350188	22.31341	27.17585	13.20542	0.748995	13.34882
22.13377	7.046987	6.182982	2.058767	15.13751	4.894565	3.972948	9.980939	4.459296	4.2015	8.540053	5.116294	15.57054	8.777183	3.142091	3.290016	7.703911	4.967666
3.775995	9.992593	1.537739	0.8296639	5.36761	0.9964769	1.432459	2.885048	0.8075898	2.324763	0.6630972	2.828633	3.57839	1.837102	23.36287	1.00164	2.834608	0.797808
1.405851	1.158866	1.719475	0.2392417	4.065803	1.619974	0.2993019	2.281103	0.1912512	0.1168847	1.888342	0.6222052	0.3386761	1.77836	0.3681898	0.1527082	1.000394	0.6573021
0.01746398	0.03992941	0.03163712	0.03821074	0.03185608	0.02069887	0.00661892	0.01710761	0.01649478	0.03360306	0.009002944	0.07003653	0.01497933	0.04365428	0.08232816	0.03602209	0.03792555	0.01889671
82.63454	154.1244	102.0308	178.5033	146.704	57.9407	82.33414	175.063	41.0364	132.5638	108.6484	46.70034	47.79987	76.1207	77.07661	142.4883	159.06	42.65699
0.4287998	1.688895	0.6910266	0.4159366	0.9634284	0.1505135	0.1943431	0.7881766	0.1023756	0.1203225	0.2669211	0.6140718	0.2789097	0.2525055	0.7622492	0.2998885	0.4390868	0.1150279
3.784466	4.363836	1.683507	3.678832	5.658232	1.334181	2.449142	4.41474	3.73316	2.187995	3.711307	1.626914	1.506746	1.76881	2.429449	2.820673	1.873373	0.9419086
0.3778824	0.1281334	0.09475521	0.02615855	0.04403586	0.171677	0.06796832	0.06831788	0.08469073	0.1006433	0.07395949	0.04373456	0.3033664	0.2586212	0.5736716	0.02311896	0.1352256	0.09702319
0.6387158	0.1774732	0.2624844	0.2536192	0.3136761	0.3547888	0.3451823	0.234309	0.3649404	0.3186232	0.5121939	0.1705081	0.2682856	0.6368133	0.2602111	0.3522342	0.1698154	0.5226026
0.04733504	0.00946979	0.1200506	0.028999	1.878313	0.2175071	0.07534872	0.007212975	0.118228	0.1275106	0.1503158	0.8188328	0.03157819	0.324931	0.2900889	0.3331816	0.1199273	0.1314604
0.02933221	0.02934082	0.03099667	0	0.02881035	0.01247991	0.05187945	0.0223484	0.01436522	0.04389709	0.007056555	0.02225473	0.01304542	0.006580088	0.1382769	0.02646966	0.04128646	0
0.04978416	0.006868798	0.03265397	0.01226988	0.0859938	0.009860383	0.006072587	0.02485126	0.01513328	0.02312207	0.03593027	0.1940694	0.01488816	0.0265723	0.05934712	0.006196647	0.09423683	0.01300271
0.06816783	0.03099448	0.07203603	0.02531021	0	0.2412541	0.005480338	0.09915342	0.01365736	0.00695567	0.008945116	0.004701802	0.02067099	0.09175244	0.01460703	0.02796149	0.01046721	0
0.3481523	0.3527774	0.1720091	0.2769994	0.2442559	0.124081	0.1239541	0.434061	0.09632416	0.1573231	0.2284262	0.2126904	0.1417689	0.08824392	0.2735414	0.1346468	0.2100175	0.09132468
0.1833471	0.1913749	0.1432074	0.06511561	0.1565957	0.5392748	0.09164528	0.1882823	0.08784076	0.06263202	0.05177951	0.036289	0.2712194	0.0536481	0.06263253	0.07193658	0.1211805	0.07044234
0.07152723	0.01788706	0.006298839	0.0243444	0	0.007608136	0.005271216	0.01816569	0.004378737	0.026761	0.004301891	0.009044775	0	0.01203427	0.07024821	0.005378904	0.01006779	0
0.1377233	0.0551055	0.07673846	0.03579494	0.1082184	0.02237334	0.06200452	0.09730083	0.02575321	0.04777997	0.03975911	0.05889576	0.09020756	0.06909343	0.02360912	0.01355812	0.03595073	0.02844964
1.013249	3.026414	0.8603536	0.8784873	1.868223	0.5717811	0.4904744	0.612183	0.2167744	0.9896351	1.606256	0.6635656	1.12897	0.7225806	0.4078287	1.029864	1.588331	0.5744745
0.7701617	0.5492581	0.4672187	0.4732825	1.225745	0.3504346	0.3594625	0.9453858	0.1833517	0.3121584	0.6999489	0.3408602	0.1950502	1.137404	0.4650397	0.4182877	0.6022437	0.3150764
4.182811	4.281343	5.208258	4.535739	7.707219	1.924507	3.125544	4.940947	1.786481	4.36729	4.095301	2.902941	12.67595	2.902274	2.522131	5.179114	4.929072	5.239359
2.827899	30.54228	6.822503	2.138339	5.325892	0.5618625	1.88742	1.66168	0.8770205	1.796642	2.998854	1.801464	0.5695246	1.140092	7.1739	2.371362	3.492238	0.7324988
0.1784722	0.09997377	0.1056158	0.07289193	0.1963325	0.1503497	0.1262644	0.1686141	0.09439775	0.176281	0.1494162	0.0324982	0.123825	0.0912835	0.09535275	0.1481706	0.07837688	0.4505989
6.456343	7.168773	4.258051	6.592278	10.08739	2.161415	2.766941	9.519337	2.317102	2.1498	3.014906	3.156605	1.906772	2.794263	3.760519	5.765217	4.88124	1.38241
1.960024	11.61837	2.88632	2.971825	2.24824	0.4169637	1.713271	2.663496	1.0299	1.163633	5.072223	0.6953552	0.3284066	0.5969427	0.9036692	3.214038	2.875693	0.8133091
55.60355	117.6466	84.88391	151.8332	152.6616	45.12871	60.45739	147.485	29.41388	129.8842	90.12668	42.28042	47.86253	83.25524	58.58151	126.6799	132.4148	41.28071
0.1820675	0.237999	0.1705355	0.1225256	0.03251423	0.06469995	0.08599401	0.2317228	0.06839446	0.1253995	0.07316697	0.1145909	0.1283622	0.03898666	0.09590816	0.07468155	0.2026851	0.04701234
0.7407171	0.5504086	0.9616634	0.1944804	0.9769805	0.3241557	0.2994504	0.7094775	0.07773435	0.7126196	0.4887688	0.2408536	0.2400146	0.726378	0.914536	0.076392	0.4289529	0.4007423

TCGA-WH-A86K-01	TCGA-TQ-A7RV-01	TCGA-DU-6405-01	TCGA-CS-6667-01	TCGA-HT-7472-01	TCGA-FG-8188-01	TCGA-DU-A7T6-01	TCGA-HT-7877-01	TCGA-CS-5397-01	TCGA-HT-7856-01	TCGA-DB-5275-01	TCGA-TQ-A8XE-01	TCGA-HT-7884-01	TCGA-WY-A85D-01	TCGA-S9-A7IQ-01	TCGA-HT-A614-01	TCGA-E1-5322-01	TCGA-FG-5965-01
0.04868374	0.02575322	0.05047144	0.03777643	0.3358341	0.03697757	0.2527585	0.03477173	0.1245556	0.1285264	0.06385921	0.04667082	0.04084272	0.02551817	0.03527744	0.07969921	0.03684749	0.04015686
0.09278912	0.06871835	0.05855433	1.037651	0.05856989	0.08969883	0.1568478	2.701617	0	0.1875519	0.3407961	0.3805193	0.160268	0.1702279	0.02941631	0.04430512	0.1092463	1.741222
0.755152	0.3777425	1.590546	0.9227883	0.8717922	0.7075897	0.7054262	0.4636583	1.203909	0.3768097	0.5028027	0.4395786	0.407314	0.5559737	0.1953007	0.4649473	1.002172	0.7133163
0.005551486	0.01644542	0.2452275	0.06385542	0.4905854	0.06439916	0.04692029	0.2938822	0.1251992	0.03847218	0.2854532	0.08278586	0.04602569	0.06110744	0.003519901	0.0477132	0.2516399	0.8141731
17.42854	5.259826	29.8004	18.47247	9.477315	10.22328	8.68813	9.454359	9.297942	16.72117	10.46838	6.840111	10.77147	10.25791	5.303058	13.78788	18.88764	21.36737
1.531878	1.422784	2.942554	1.676777	4.221084	2.379952	2.277615	0.7673719	4.823695	1.068164	1.28034	2.435013	0.8882674	2.482203	0.8818919	1.756269	5.664654	2.086108
2.826873	2.609599	3.128055	1.305491	1.654411	3.123115	2.507464	1.11316	2.725957	1.028909	3.591722	1.186728	1.36222	2.396932	1.445945	3.856516	1.981101	1.741955
2.128007	1.675403	4.579325	1.818845	2.093236	2.135007	1.282253	0.955544	3.290574	1.415021	1.019088	1.351433	1.442394	2.512335	0.9406483	3.301474	2.655599	1.732587
0.05461905	0.03033759	0.06893441	0.1483369	0.02585728	0.0264	0.04616317	0.33733	0.009057276	0.212914	0.1420952	0.0610875	0.03773584	0.03757587	0.01731551	0.0130398	0.01607658	0.3626735
2.742389	2.026565	3.119173	2.127293	5.659233	2.7921	1.021755	0.9667854	4.195053	1.14009	1.658659	1.332103	1.606683	3.362259	0.5399539	2.729779	5.910667	2.857421
0.02562088	0.009487234	0.04041997	0.01227923	0.01617228	0.01857569	0.02887249	0.04144271	0.01699446	0.01849525	0.04705021	0.004775853	0.01327593	0.005875402	0.02842845	0.006116751	0.01885313	0.05177687
34.1007	38.09869	173.5955	21.57167	90.97572	48.33862	19.04353	13.51959	140.3836	10.53579	23.67049	28.75475	14.40269	77.42843	16.22751	36.79228	99.13025	48.2368
0.5952791	0.7089433	0.9492759	0.7016776	1.497919	0.9564972	0.2878212	0.2626052	1.051159	0.2787382	0.453026	0.68677	0.5752268	1.081011	0.2295211	0.6721772	1.827914	0.5318243
0.2718122	0.4180854	0.1979151	0.09352758	0.2771547	0.1576558	0.117811	0.1106852	0.1220455	0.2680618	0.4633195	1.593285	0.5315979	0.2339856	0.07954229	0.3833661	0.2486317	0.2269641
1.493367	1.057881	1.647125	0.8215232	2.144838	1.452	0.3487736	0.3971856	2.253878	0.3599699	0.9565351	0.9682457	0.7237233	1.905865	0.2799424	1.645195	2.565998	1.149685
0.02952381	0.005466232	0.2189133	0.05659912	0.1164742	0.3068108	0.1913068	0.02604865	0.1077081	0.04688797	1.066279	0.3962432	0.06119325	0.07447469	0.02339934	0.06343687	0.2693913	1.508653
0.07951891	0.02617361	0.03902904	0.0282302	0.2119283	0.1195764	0.02987029	0.03637872	0.02344236	0.04592263	0.2379726	0.09223015	0.006104326	0.0324184	0	0.06750015	0.1040249	0.2703819
1.945478	2.292559	3.05839	2.150012	5.222677	2.638491	1.209035	2.366052	6.194142	1.642329	1.908932	4.684539	1.897573	3.637344	0.4535015	1.710325	9.967173	4.334661
230.9267	97.13465	438.5917	59.59608	419.2747	82.05623	120.1824	31.87199	316.443	37.29066	102.9126	140.5662	27.31487	108.367	22.10512	174.3238	516.7529	196.0536
0.4304783	0.09446113	0.9558133	0.1562213	1.180827	0.2568774	0.1946435	0.1000317	1.177406	0.1534591	0.2049525	0.4398516	0.0734355	0.5118694	0.03032704	0.3552639	2.940855	0.5431419
16.46339	4.889371	21.07294	9.833314	14.1804	9.130021	8.299443	1.402296	23.99138	3.331306	4.873062	5.991646	1.94085	14.15345	1.136349	10.57947	47.59961	9.255614
0.06559707	0.07507863	0.03763169	0.1905363	0.1468026	0.1441191	0.04704138	0.05261439	0.2096433	0.08609702	0.03650381	0.06224963	0.03708037	0.06017112	0.08696426	0.03416881	0.08074192	0.08263743
1.731765	1.388169	1.827656	1.454315	3.371488	2.899189	1.564603	0.7181046	3.692528	1.182115	3.793513	1.625177	0.9810067	2.358434	1.228436	1.65694	5.702703	2.794779
11.51865	7.300541	8.262684	7.566535	10.05432	10.60907	8.099709	3.503405	8.44763	4.853666	6.533855	8.994196	4.486034	15.55658	2.446558	11.06826	17.69583	7.560194
5.243577	3.563234	1.286526	5.555075	1.909712	2.668487	2.407732	3.393631	2.39626	2.232294	2.710584	2.654104	2.20576	2.348821	0.8789958	2.242828	3.626859	3.602068
0.1764492	0.3484689	0.5121999	0.353299	0.6088631	0.5685755	0.3115204	0.1522203	0.9987387	0.1902141	0.3528344	0.2894405	0.2438145	0.615045	0.08950162	0.1460356	0.6994059	0.2988511
15.72442	8.245822	23.31692	7.678615	21.76066	9.039758	8.202737	2.950988	16.17985	4.699307	6.949135	10.25357	4.501495	9.090967	1.166576	12.30826	29.13782	11.30795
8.180965	5.444332	6.334425	14.11348	9.39742	9.407539	4.46579	5.525172	7.877281	4.79826	10.92585	7.075293	5.206864	9.722764	1.89358	7.812524	13.07676	9.904203
0	0.0330103	0.03281576	0.2943271	0.03282449	0.06463306	0.1255752	0.9132514	0.02463803	0.5791788	0.2773951	0.04985193	0.05645794	0.08177252	0.8195831	0.04966008	0.2186614	1.964544
0.9742857	0.1340571	0.3225288	0.5919712	1.132511	0.4426122	0.2729838	0.2411279	0.289576	0.1506564	0.3030851	0.3056631	0.2758712	0.483475	0.1080206	0.7321239	0.2946067	0.4734016
0.08000537	0.02734656	0.0271854	0.03146167	0.2058871	0.05354365	0.05201484	0.02533934	0.08164305	0.0355412	0.0376723	0.04588732	0.0340154	0.0395164	0.1092584	0.05877094	0.01811446	0.07817578
0.3225077	0.1729565	0.6245885	0.2558354	0.7300503	0.709538	0.3759703	0.2027801	0.7892949	0.1798276	0.2723005	0.2321758	0.2305012	0.9895036	0.09871685	1.093871	0.4779081	0.7384374
4.390774	4.978214	7.3077	4.113832	9.538825	8.708945	1.890214	2.140426	11.25578	2.477322	6.353898	4.606935	4.168401	11.85675	1.249776	7.577619	11.15353	7.820867
0.3307967	0.4470949	0.6419013	0.3487877	1.780053	0.6635429	0.2376466	0.3161812	1.195835	0.1480537	0.2379279	0.5364601	0.3028216	0.4551516	0.06292217	0.3869761	1.416686	0.3676764
3.880157	2.226021	1.991613	3.44861	6.381754	4.081118	2.509625	1.711718	2.582791	1.514916	3.010786	2.709752	2.756282	6.065686	0.5977248	3.940259	6.297559	3.202807
2.115666	1.419812	3.794077	2.544436	1.747172	2.719629	1.498896	1.704829	3.12646	2.456722	1.180494	1.865065	0.8934763	1.196657	2.236917	1.912053	2.200408	2.004304
4.641344	2.998004	3.3083	2.924943	4.973889	4.788984	1.260171	0.7921291	6.029644	1.310111	3.631142	3.19171	3.195588	7.897199	0.6607387	4.661968	14.8222	3.97587
2.075335	2.05441	1.449329	5.136759	0.995587	2.501084	1.087505	3.120929	1.578479	2.720636	2.22317	1.820785	1.27611	1.154894	1.127253	1.347671	2.577806	4.154073
0.4310444	0.6777404	0.7114092	0.4958046	0.7660147	0.8974833	0.2316651	0.5148777	1.033689	0.2680791	0.4871203	0.5883998	0.5268856	0.9854333	0.3700092	0.6459451	1.042316	0.6049779
4.604122	3.885431	6.761377	3.962804	16.7411	4.857399	3.449354	2.945502	13.95009	3.033124	4.58241	4.903567	2.61152	8.08681	1.254187	6.253183	14.47531	7.007022
1.93891	2.103537	4.835328	2.66672	5.812766	3.850088	1.749299	2.381477	6.260342	2.436931	3.134865	2.259793	1.680511	2.997483	1.820634	2.998163	7.001643	4.255967
1.898028	1.351908	2.448333	1.241421	3.664666	3.054427	0.8146736	0.876711	3.310355	0.8994648	1.496732	1.910528	1.130059	3.732973	0.7149839	2.340319	4.229429	2.07579
0.8140153	1.023441	1.176691	1.022202	1.583279	1.839162	0.5333279	0.5511767	2.831549	0.4427672	1.593357	1.298581	0.5689357	1.588872	0.3060733	0.8044721	3.805698	1.655963
1.672119	0.8796542	2.103095	0.6042672	2.278352	1.393474	0.9486635	0.5426385	2.432426	1.092193	0.7129628	0.8082467	0.8285949	2.559871	0.9285901	1.211375	2.606813	1.305055
0.06517256	0.07239885	0.08739482	0.09370513	0.07713357	0.02362576	0.07803401	0.1677121	0.07024762	0.0517516	0.1047226	0.06074237	0.1407099	0.05978172	0.04648775	0.08557646	0.08152733	0.4045266
0.08504887	0.01799601	0.2606822	0.1941005	0.2684207	0.117452	0.1026885	0.08575773	0.1450629	0.04911622	0.1041226	0.0362366	0.1259133	0.2006073	0.01540713	0.1160265	0.1001332	0.364794
0	0	0.04109911	0.1872833	0.02055502	0.06295934	0.0183485	0	0	0	0	0.02428049	0	0	0	0	0.0191699	0
0.07737405	0.1203345	0.1708934	0.0988875	0.595844	0.1944726	0.1395101	0.1774931	0.5748133	0.1027731	0.09472655	0.2711504	0.1176056	0.2626036	0.0196235	0.08127835	0.2915109	0.4601574
39.2342	10.98459	30.90206	19.7706	48.85481	42.5961	17.246	3.395274	35.99782	11.86251	28.25985	34.68837	17.17601	45.47995	4.362848	19.62801	98.44663	24.00009
4.948121	9.322123	8.414061	5.738595	6.407592	5.340164	7.209556	4.199063	7.378495	3.081157	9.265928	6.611372	7.046	7.05944	2.604471	7.601923	11.69036	7.104397
7.27654	7.588459	15.18788	5.34803	16.12482	12.44828	4.474552	3.152828	15.18328	5.090473	6.713789	8.797367	4.177202	13.37525	2.506355	10.52792	23.18282	8.827526
3.009548	2.196244	5.314336	1.664496	9.29284	5.920714	1.110666	0.921333	6.502401	1.026556	2.090033	2.959161	1.678008	3.55166	0.4686791	2.689131	10.21554	2.261043
45.13629	50.58173	54.11677	97.52806	104.8158	100.5259	52.61053	23.99296	80.3557	33.26775	74.95457	68.49228	34.39538	111.7823	14.01576	42.21943	244.6097	75.54457
0.9394389	0.745431	23.38958	1.212896	1.219256	1.130556	0.8912235	1.014931	5.980187	1.57777	0.9506133	1.093581	0.7252144	2.484074	1.73832	1.025291	1.410789	2.186318
0.7366044	0.4474042	1.765694	0.6942077	2.88005	1.173126	0.3792132	0.3833947	2.801504	0.4559971	0.551461	0.8297663	0.6956374	1.059692	0.2721609	0.7894615	3.038491	1.315715
1.894825	1.570008	0.8997528	1.390662	2.30919	1.450866	0.8350933	0.8165841	1.866605	0.6825659	1.504407	1.23097	2.281236	2.357609	0.5590707	2.991927	1.060226	1.310711
0.5733371	0.3715299	1.23616	5.633019	0.3920573	0.6466162	1.049914	12.36525	1.711339	2.290146	2.705309	1.567467	0.6766943	2.235129	0.4846967	1.43723	2.179767	12.04167
0.04506474	0.0238388	0.1421899	0.02468347	0.8086662	0.006223412	0.09068575	0.03029356	0.1110262	0.0334609	0.01970404	0.0240008	0.0133435	0.005905306	0	0.01229577	0.02273891	0.0780606
124.2361	48.68045	341.8888	32.33417	409.5319	34.21274	104.3518	26.79163	342.0548	22.26036	62.56786	84.47129	11.2724	43.83068	10.38065	100.7651	447.6765	147.3046
0.01564099	0.03475051	0.07402654	0.6246839	0.04936415	0.1663208	0.1101627	3.624783	0.1400591	0.2167863	0.7850991	0.1166222	0.1242716	0.1004306	0.0842955	0.03734144	0.423547	3.142804
1.330671	0.9514949	1.856787	0.7220463	2.541542	0.792882	0.475073	0.2361578	2.218017	0.5995393	0.9514774	1.466878	1.200702	1.557321	0.2800237	1.70893	1.701738	1.304827
0.5544716	0.5983524	1.809472	0.6782692	1.649957	1.271098	0.4641676	0.2329554	1.83892	0.3476571	1.105509	0.8268491	0.3611894	1.845519	0.4319176	0.8623289	2.499341	0.8506834
2.10261	2.676997	2.580293	2.550009	4.947346	4.39384	1.794738	1.326212	4.000038	0.9869167	4.01459	2.982053	1.953384	5.589095	0.7828726	4.588301	10.1128	4.628845
47.7733	28.27673	46.09865	38.1137	62.56834	60.34684	21.85701	16.75404	48.10362	17.5207	43.45188	30.60257	27.78889	70.55701	8.517629	44.23009	100.03	43.51038
0	0	0	0.0180461	0.1604306	0	0.0477363	0.01661073	0	0.01630886	0	0	0.01951089	0.02590424	0.01790558	0	0.01662443	0.03261152
0.3748552	0.2107799	0.6527065	0.2661565	0.5258094	0.2415805	0.1838348	0.2531529	0.7873708	0.1924276	0.2974501	0.2691473	0.1870442	0.6303889	0.1496476	0.404377	0.7478256	0.7334907
0.02776018	0.04111761	0.1051079	0.09123106	0.3504529	0.09584157	0.4312625	0.4035449	0.1893957	0.08473879	0.3107281	0.06505248	0.08493676	0.07275415	0.08549177	0.07574274	0.1050549	0.9594271
9.160936	7.677667	9.890877	9.416062	17.87385	13.71717	11.27793	3.957265	13.55473	3.676393	7.129218	7.983984	5.144523	12.14059	2.919192	8.122405	15.08273	8.420187
1.614915	1.181813	1.149492	2.029697	1.384105	1.616621	1.770274	1.262762	2.594188	2.015526	1.344607	1.147044	1.232063	2.021917	0.9851093	1.604311	3.183163	2.156556
0.07001422	0.03888868	0.08284179	0.05033323	0.1767761	0.008460304	0.07396866	0.08236407	0.0754663	0.01516261	0.01071452	0.02610197	0.01813959	0.02408359	0.01109807	0.02507289	0.05667199	0.1920232
23.25956	13.20084	30.23675	21.60208	31.29911	31.73915	9.565928	6.495985	38.31416	8.609487	23.15004	23.66275	13.38291	41.91658	2.766171	17.18873	66.62775	21.74994
5.946663	5.259342	7.668072	11.58858	6.165559	11.03401	4.236816	6.879968	7.77481	7.138094	10.46168	7.493733	3.259635	12.58881	7.836951	6.5471	7.789744	14.56434
0.5467517	0.3213211	0.2648911	0.2209025	0.4586729	0.3307652	0.5624774	0.4772081	1.48107	1.163191	0.6024333	0.3287649	0.2315218	0.5015263	1.341933	0.5187594	1.746361	0.5295483
1.915926	0.9562211	6.33432	14.4523	1.814043	3.663972	3.989607	34.88611	8.205233	7.913584	10.68271	6.024759	2.53229	5.272344	1.980631	5.528695	9.439766	50.35835
5.091354	2.927782	64.76464	3.876144	4.387494	4.213541	4.417339	3.845654	15.52884	4.426538	7.057289	2.822894	7.4848	10.78687	4.027531	9.983379	2.060151	6.40341
2.833824	0.4562372	8.32756	1.810877	3.065848	2.068688	1.227696	0.8238851	2.955028	1.09353	7.764377	0.9670265	0.9811724	1.683377	0.4358314	0.9970206	3.439503	3.396037
1.017244	0.3413647	0.9628934	0.7312966	1.725643	0.3841269	1.352328	1.028393	1.054286	0.798584	1.255108	0.6162619	0.2635519	0.3499128	0.1814006	0.4553582	0.6362583	4.267483
0.03350947	0.009024237	0.05767108	0.01557331	0.09229825	0.03239345	0.03604578	0.01075097	0.03030956	0.03518529	0.01678277	0.01589973	0.004209347	0.002794335	0.08305476	0.01163648	0.01793306	0.0175893
51.84514	81.15112	89.69839	83.43519	155.2072	122.7554	55.57469	33.6081	147.4435	39.88997	109.4787	84.80318	44.3389	149.4898	21.02453	63.41778	323.7368	100.314
0.3141488	0.3102057	0.6071184	0.2467529	1.652462	0.2404183	0.2433875	0.1116384	1.541118	0.07559281	0.3365269	0.4343113	0.1175648	0.3301874	0.07884406	0.2062505	1.479466	0.3816711
1.165505	1.507485	3.579033	2.113717	3.585166	2.515213	0.9480687	2.510444	4.061421	1.422012	1.396744	1.689086	1.468708	2.883554	0.6817392	2.335763	4.242284	4.63014
0.03128198	0.03475051	0.01480531	0.2848559	0.07404622	0.03780019	0.1145692	0.5243975	0.05706113	0.153557	0.2202107	0.1341155	0.05943425	0.129125	0.05950271	0.1269609	0.08747166	1.995861
0.404391	0.2673986	0.2187342	0.7660156	0.1914433	0.09773075	0.341785	0.526692	0.153277	0.5671641	0.5039236	0.3661334	0.1796081	0.264958	0.2289311	0.3310104	0.2040489	1.042383
1.190637	0.08560871	0.02917859	0.1181894	0.9558528	0.0223492	0.09769983	0.2515739	0.8587639	0.2336505	0.0495321	0.2413333	0.1197963	0.06362052	0.1759036	0.05519493	0.136098	0.0333723
0.01193859	0.008841556	0	0.03814513	0.02260746	0.02308196	0.04708811	0.07724444	0.00791893	0.03447302	0.007308015	0.008901639	0.09073097	0.02190214	0	0.136811	0.01405603	0.1033994
0.006288456	0.009314288	0.007936627	0.04688208	0.03043182	0.01418438	0.03307052	0.06411322	0.0361501	0.07505347	0.01154813	0.01562931	0.04054998	0	0.00664529	0.01601399	0.004935853	0.1028763
0.01513374	0	0.004775054	0.0338478	0.458527	0.02194458	0.3624061	0	0.07026803	0.004369912	0.01852775	0.08463004	0.03659521	0	0.009595498	0.02890435	0.03118127	0.01310724
0.07729229	0.09403978	0.2821988	0.1552304	0.7213664	0.1547732	0.1057663	0.1753571	0.8898727	0.1084035	0.1182831	0.1440765	0.1068012	0.1569905	0.04900692	0.3163343	0.7572575	0.4048421
0.1265374	0.04325164	0.1351327	0.04976016	1.536007	0.009409476	0.4277899	0.005725287	0.1097585	0.02248496	0.05362467	0.07983354	0.04034937	0.02678555	0.006171588	0.04647641	0.1489802	0.0955429
0	0.005390082	0.01837138	0.03255621	0.004594065	0	0.004100907	0.08561922	0.01448286	0.004203161	0.01336557	0.005426711	0.005028397	0	0.004614673	0	0.008568982	0.138678
0.03669066	0.02037945	0.09068471	0.0468923	0.1138685	0.03842442	0.03790157	0.04316254	0.1784721	0.06533296	0.02245959	0.01823817	0.06337316	0.05328825	0.04846581	0.04379781	0.1529934	0.04943176
0.4210876	0.5497592	4.484472	0.5187503	2.583984	1.670219	0.8120804	0.5183443	1.523245	0.6092048	0.688251	0.5470215	0.5548586	1.696344	0.3770878	0.8208291	1.241985	0.4010463
0.6095169	0.3546713	0.6318988	0.2782107	0.945352	0.496626	0.6525287	0.34315	0.3754175	0.1458284	0.5703191	0.415513	0.3489198	0.2635756	0.08833421	0.4407075	0.8560196	0.8144727
4.909416	2.726884	3.347918	3.036026	8.422003	5.932388	4.439371	1.74659	4.123075	1.349014	3.22334	3.247265	1.559168	5.157035	0.9288189	4.537078	6.805663	3.154721
1.02612	1.067521	2.2458	1.883876	3.413289	3.054563	0.8718493	0.9005482	2.290377	0.9500256	6.545438	2.307428	1.389743	2.599624	0.3201404	1.290989	4.755747	7.777444
0.1656203	0.1678452	0.08251127	0.2673729	0.06602656	0.07583894	0.09331981	0.2307247	0.04625551	0.08054459	0.09604592	0.2404799	0.1324929	0.05597082	0.08290343	0.08324284	0.1949948	0.2617202
4.501877	3.383733	9.91382	3.625692	10.40853	4.162295	2.364982	1.081057	9.047392	1.848524	2.897963	3.2566	2.981901	5.095573	0.6612257	5.502096	13.54867	4.545909
0.8476151	0.6933773	2.020678	1.118692	0.7728175	1.405809	0.7007834	1.575525	2.416109	0.8942221	1.348003	1.999261	0.8611913	1.758276	0.2458819	1.18771	4.953869	2.947653
34.75013	61.82071	81.01882	87.86522	133.3617	109.7752	49.11538	27.74171	145.0504	29.6735	94.101	73.04147	38.12333	139.0002	17.40766	59.38917	306.7006	85.32867
0.113682	0.0991587	0.1307239	0.05973063	0.2710851	0.1636228	0.0412798	0.09955788	0.3217318	0.04230909	0.05103165	0.04520719	0.148357	0.1459901	0.03203543	0.09649974	0.1561522	0.08022612
0.4651429	0.3444784	0.3587554	0.7265786	0.5056534	0.1498838	0.2766481	0.3495934	0.2742504	0.2835462	0.4429128	0.2504806	0.1249746	0.2370377	0.09830743	0.3454846	0.5172164	1.029525

TCGA-HT-7860-01	TCGA-DB-5279-01	TCGA-FG-5965-02	TCGA-S9-A7J2-01	TCGA-E1-A7YO-01	TCGA-HT-A5R7-01	TCGA-HT-8012-01	TCGA-CS-4938-01	TCGA-DU-5853-01	TCGA-HT-7686-01	TCGA-DU-A7TA-01	TCGA-HT-8104-01	TCGA-HT-7677-01	TCGA-06-2565-01	TCGA-28-5209-01	TCGA-DU-7015-01	TCGA-FG-A4MT-01	TCGA-E1-A7Z4-01
0.1597644	0.1287376	0.01560166	0.02894851	0.0165046	0.02899812	0.05924528	0.02177149	0.1385218	0.08264785	0.2355692	0.0340877	0.03290714	0.6362979	0.3483445	0.04904638	0.004675092	0.125792
0.06558551	4.320781	0.04163053	0.2457778	0.3302991	0	0.08468922	0.03227429	0.3696231	0.875644	0.6360616	0.02675223	0.2469584	0.1920655	0.2178519	0.3523488	0	0.1748208
1.470178	0.4464007	0.1307667	0.2907603	0.4113362	0.3559828	0.3022948	0.5322323	1.146639	1.764214	0.9615796	2.135179	0.5269459	3.8008	3.138967	0.7258695	0.320602	0.6265118
0.2707504	0.407833	0.02490716	0.02520798	0.05708873	0.08744391	0.02364542	0.2432985	0.1889759	1.614356	0.1925134	0.2400841	0.2462548	0.6297121	0.9427837	0.0542075	0	0.01673498
15.23624	10.61318	5.464527	6.683308	9.277344	10.37603	7.998585	4.637291	11.28055	33.01086	11.21671	12.60032	10.82388	18.27313	16.57727	19.11178	6.470288	7.911281
3.237889	1.29997	0.6570779	1.348819	1.023683	1.146414	1.833477	1.993698	3.352855	7.026591	3.765592	3.259054	1.167373	5.07511	2.59862	4.793097	2.079286	2.307341
3.548446	0.8748646	1.350009	1.941604	1.42814	1.716823	1.243076	2.902942	2.305492	2.789751	4.24023	4.986016	1.723456	3.619007	5.794191	3.410643	1.181668	2.213951
6.424559	0.6096531	0.9457108	0.9551008	1.216038	1.66694	1.433708	1.788555	2.207339	4.612794	2.658144	5.469515	1.095815	4.129666	4.181864	2.982465	1.525228	2.306931
0.1737271	0.4739197	0.03675787	0.08267071	0.03240434	0.03795568	0.0664682	0.01899782	0.3164707	0.1049962	0.1321444	0.03936833	0.3230415	0.3504759	0.3526478	0.09629532	0.01835771	0.123487
5.608898	0.9507753	0.2778739	0.6715267	0.6642268	1.785029	1.134184	2.251587	3.87756	9.216665	4.46168	2.48617	0.7854499	3.04966	2.183495	5.131736	2.265793	3.682426
0.02263679	0.1926667	0	0.01454229	0.02026711	0	0.03897389	0.004455779	0.1902029	0.00447745	0.08264892	0.02954723	0.2424533	0.1378858	0.1503829	0.02779724	0	0.004827141
115.0821	13.84823	6.092884	17.21135	8.61501	30.55074	17.00398	38.93258	106.586	239.5224	103.0451	156.5692	17.46007	48.66023	91.78328	152.4573	36.43749	73.93327
1.017777	0.3629551	0.06496453	0.3287458	0.1590841	0.3242273	0.261868	0.9009578	1.074941	2.091841	0.8109277	0.4568967	0.2402672	0.8591933	0.5162331	1.488062	0.4380041	1.291292
0.4610957	0.1040144	0.04127558	0.1519058	0.1257005	0.2789713	0.18829	0.2298118	1.172103	0.517399	0.6340108	0.1929028	0.2052806	0.2492874	0.2683568	0.3334653	0.2979653	0.1481186
3.016721	0.3830965	0.1165235	0.6224128	0.2225658	0.6176458	0.7664435	1.216518	2.066009	5.694606	1.710514	1.564976	0.7475571	1.44074	1.002726	5.175313	0.913653	2.211735
0.4747497	0.9777262	0.01324608	0.04468687	0.1050952	0.2051659	0.05838423	0.1283637	0.1817568	0.1857427	1.446445	0.06384054	0.6810064	0.3972264	0.7913636	0.1481466	0.0595385	0.2280617
0.206088	0.07666348	0.007928167	0.05349281	0.03494586	0.04911912	0.03763273	0.03687813	0.2175736	0.08029121	0.2850172	0.06113671	0.1724472	0.2340939	0.2005252	0.05272278	0	0.05992754
9.941233	1.287557	0.6931484	1.125896	0.9776853	4.299716	1.08628	1.078714	6.241254	16.09142	9.076849	3.629385	0.7952974	3.600588	2.857007	4.516356	3.615594	3.26437
370.4519	78.50674	36.15324	24.36813	36.37319	144.7447	79.76059	79.36889	316.6407	672.5138	598.9902	244.1671	45.75298	502.6678	248.596	333.6203	80.37815	336.8875
1.619028	0.273606	0.0429194	0.03619814	0.08408026	0.1920447	0.2101936	0.3253409	1.093161	3.585009	0.8743386	0.5454804	0.2105985	1.403684	0.8276799	1.562583	0.1643343	0.5206737
14.27043	4.142044	2.662852	7.557646	3.072811	6.982196	10.64644	3.508304	11.18734	104.5082	17.7812	17.97698	5.433103	22.03755	23.1853	40.74315	2.532765	23.58319
0.08430098	0.08278895	0.05886123	0.04964346	0.05660709	0.05525393	0.03991391	0.06637453	0.05182895	0.09587742	0.08656603	0.05157935	0.03879704	0.1678738	0.06533756	0.07116921	0.06413809	0.06291813
5.055661	1.013262	1.143127	1.343228	1.044478	1.503137	1.326244	1.442408	2.943431	5.579698	3.068775	2.234365	1.638397	3.213783	3.188305	4.121274	1.520922	3.28276
15.01828	3.259039	1.65091	4.50761	2.219879	5.803967	6.660284	6.987034	11.23061	28.09407	15.44951	9.609954	4.293363	14.14257	9.563615	14.28351	10.01914	17.5935
3.619822	2.405782	1.225678	2.221758	3.751148	2.572795	2.049305	0.916176	3.661911	6.19645	2.393874	4.413101	1.48553	3.129527	2.913403	3.443919	1.913138	2.565838
0.9146016	0.1769176	0.05277684	0.2225594	0.1861042	0.1525906	0.5654515	0.5400849	0.6560222	1.940605	1.214288	0.5697713	0.1252319	1.246668	0.6444208	0.7848944	0.3637396	0.5939628
19.43342	3.999329	2.484952	2.526448	3.278457	7.864745	6.08811	6.940578	17.38528	35.95892	30.31081	14.84749	4.223761	28.7869	12.06852	23.65576	9.409809	18.04711
12.47612	5.208502	1.769375	4.095339	5.446729	7.958532	8.165741	7.977269	12.08614	23.63781	18.19751	8.518446	5.10656	9.246275	7.386314	11.64875	6.623203	7.430209
0.1837813	1.589987	0.3399677	0.04497696	0.2879495	0.3235131	0.09944547	0.005167878	0.5326686	0.7270216	0.1198218	0.1156589	0.166963	0.2829389	0.279066	0.02417973	0.0099875	0.03359153
0.7563837	0.3603218	0.06210456	0.2296612	0.1474012	0.1578544	0.2494398	0.8666441	0.6362365	0.7108294	0.8887689	0.1811257	0.3589668	1.644207	0.5916525	0.6296068	0.3077783	0.7222105
0.09134945	0.05695936	0.1049239	0.01863002	0.04381432	0.0912362	0.04119156	0.04281199	0.1248053	0.07313435	0.1836372	0.06387651	0.02547933	0.2140116	0.04334723	0.07344735	0.008273895	0.03246608
1.265548	0.2444528	0.119748	0.2609043	0.1143624	0.4224714	0.7172783	0.4177587	1.296783	2.021213	1.174888	1.17992	0.3617586	2.080955	0.8007058	1.713318	0.1495121	0.7459128
7.229596	1.776078	1.099773	2.400146	1.714148	4.55415	3.55196	8.635919	10.66591	28.36112	7.234822	8.564496	2.992974	6.632662	5.067844	17.09466	4.977235	9.855591
1.151538	0.2583237	0.02968288	0.2190518	0.08504374	0.2911764	0.2012802	0.9549903	1.203917	1.803651	1.040421	0.6962207	0.293472	0.7942761	0.3041881	1.458016	0.4558472	0.5048266
3.582744	1.699178	0.6497582	1.964546	1.178027	1.645681	1.853854	4.524052	4.314365	6.895486	10.3021	2.032561	1.721174	8.913935	2.320245	5.655007	3.673628	5.096737
4.730763	1.164759	3.247162	0.9744101	1.588325	1.618678	1.846426	1.096915	3.294671	3.104537	2.310005	2.351582	0.8453821	3.390688	3.098396	1.938459	1.319224	1.55595
2.82733	1.205613	0.467545	2.281028	1.00189	0.7575944	2.497324	4.633999	4.186006	14.16573	2.870327	2.778002	1.80636	3.305269	4.786663	10.04511	2.899023	7.182954
4.185541	2.248913	0.9683592	1.235539	3.666398	2.743356	2.685514	0.5486072	1.773657	2.712855	2.1702	3.031611	1.726608	2.027601	1.667481	0.9644457	1.246253	2.210492
0.7359005	0.3414035	0.3332281	0.336249	0.3514643	0.4178197	0.4519251	0.4290237	1.263176	1.960856	1.053557	0.5621068	0.2549389	0.4941552	0.332148	1.266278	0.4903493	0.6996691
17.16744	5.070089	0.9945837	1.798108	1.267969	3.33378	4.340556	7.686839	15.7427	25.12757	12.71603	10.90782	4.161948	16.88128	8.416394	16.77407	5.104727	9.646866
7.635065	1.807101	1.108739	2.499503	2.747846	2.790167	2.742552	3.492282	5.940797	13.11358	5.010589	4.830574	3.180588	6.659471	9.29599	8.007631	2.533571	3.853621
3.488083	0.8040672	0.3907178	1.292774	0.6491406	1.256902	1.256797	2.951392	3.832934	6.899262	3.295475	2.085891	1.162199	2.130771	1.481039	4.921015	1.283376	3.521317
2.916967	0.4051681	0.1274002	0.780797	0.2470847	0.7103797	0.598973	0.9350012	1.754985	4.062555	1.908347	1.713786	0.5486234	1.489018	2.022273	2.058999	1.03075	1.911723
2.836542	0.2734968	1.510765	0.6807243	0.6932829	0.7906827	1.340208	1.283538	1.86253	1.862121	1.283398	1.423003	0.9957863	1.55632	2.382572	3.177902	1.209283	3.137345
0.2821511	0.2733205	0.05848031	0.07398328	0.0966638	0.09812724	0.104096	0	0.3422175	0.1594519	0.09197844	0.08925268	0.1108197	0.2428229	0.1275111	0.1944487	0.09857154	0.08595257
0.2404581	0.105422	0.02180446	0.03677969	0.09610994	0.0788025	0.06653541	0.4395047	0.08799741	0.1273968	0.9700384	0.1681414	0.07904554	0.4426245	0.1901707	0.3756843	0.06533784	0.2655367
0	0	0	0	0.02575955	0	0	0.06795969	0	0.0227634	0.07878525	0.05633192	0.01925993	0.05392408	0.07645481	0	0	0
0.8094088	0.1253207	0.05554312	0.04098934	0.1162912	0.1362136	0.1224076	0.1076503	0.2689899	0.6652689	0.361915	0.2766177	0.06864372	0.2178144	0.1756048	0.3609703	0.1560346	0.2040889
15.65365	15.80335	4.825168	20.28983	8.819845	8.736918	21.53133	60.53436	44.41916	73.9583	66.74481	17.45065	18.02729	39.43726	45.17482	49.00936	41.52618	41.15886
6.200627	3.539594	3.677424	5.352644	4.825467	3.954749	8.515243	5.833265	6.709303	8.673635	8.567316	6.528615	6.15467	14.06541	9.917555	6.100964	8.721677	4.931719
20.13056	4.080297	1.015253	4.209067	2.654261	5.107367	6.273456	9.039129	20.59155	41.12724	16.14765	11.11956	3.801785	10.88139	9.872962	22.75368	8.793981	17.27474
4.584093	1.30312	0.1895096	1.917985	0.5916867	1.736705	1.959729	6.586839	6.271474	12.3335	4.42836	4.597229	2.076645	6.040069	3.388311	10.63575	2.697393	3.727069
82.62294	18.98361	10.27371	28.511	30.80247	55.05564	66.13691	53.20346	109.9636	461.1512	172.6783	49.19903	21.32074	88.35206	50.70144	181.2765	67.43859	117.3399
9.807807	1.261517	2.073024	0.613023	1.129865	1.838587	0.9624235	0.5034764	0.9824225	6.824962	1.866994	14.17825	1.47978	2.476335	9.332671	1.697866	0.6379431	0.8560849
3.31867	1.033044	0.1046133	0.5694891	0.4527319	0.7266958	0.6158752	1.161234	2.890107	5.030556	1.534254	1.570636	1.018631	2.79932	1.111469	3.067338	0.6697021	1.026381
2.081892	0.9875485	1.010052	1.405596	1.48404	1.025584	1.255683	1.174573	2.364266	2.91137	3.479836	1.027695	1.242739	3.308564	1.380126	1.577455	0.6053314	2.106638
0.8273814	24.18278	0.278668	1.084745	2.39994	0.3984215	0.4942169	0.3157491	2.802936	7.748427	6.83928	0.9918002	3.066004	3.461379	4.3561	1.52918	0.1284674	6.895307
0.2548225	0.1079956	0.005776752	0	0.02546283	0.04771998	0.003917226	0.008956916	1.268257	0.1260067	0.08826143	0.09651727	0.04188376	0.309157	0.09572724	0.4819429	0.00865512	0.02911026
398.3767	63.4697	23.68738	11.99242	22.63443	107.3703	58.92614	58.51329	267.6155	727.6761	576.8927	229.7014	45.85069	593.1154	202.3758	256.1141	37.38317	203.9093
0.171359	2.144282	0.01403489	0.2544956	0.1299126	0.04347674	0.166549	0.03264188	0.7703224	1.399494	0.2333561	0.04509487	0.4486629	0.4985828	0.2019721	0.1357569	0.02102803	0.02357492
3.86355	0.50099	0.2058675	0.1953318	0.5989003	1.286085	0.2268485	0.4029896	2.637885	2.309415	4.218527	1.29316	1.370498	2.806613	1.22543	1.250796	0.8173779	1.741983
1.847592	0.4490314	0.1279377	0.4435989	0.1754434	0.4697131	0.5590865	1.553889	1.85877	2.580262	1.826966	1.224077	0.7495755	1.796983	1.326594	2.518005	1.001021	1.253593
5.75497	1.863214	0.4415688	1.333935	0.87055	2.055956	1.998005	2.925353	5.13882	11.47062	6.573435	2.636359	1.444667	5.252298	5.7558	8.003669	3.223739	4.672831
64.10727	14.17753	6.110371	14.37759	10.14455	32.23485	26.37622	53.32461	57.99404	163.3634	80.94364	40.61041	15.45185	69.08352	36.78631	91.80453	36.9761	70.00958
0.03992161	0.3430488	0.02534032	0.04274396	0.04467814	0	0.06873328	0.01964522	0.02045345	0	0.06832376	0	0.033405	0.04676377	0.02210092	0.03063902	0.07593317	0.02128253
1.295342	0.1646353	0.11835	0.1260836	0.2141569	0.3923476	0.1942976	0.2221351	0.5128251	1.436343	0.8061493	0.5243653	0.2011778	0.8448884	0.4237478	0.7531429	0.2799789	1.030604
0.7372093	0.3119841	0.07472887	0.02100873	0.05960402	0.2902837	0.08928237	0.04689892	3.185335	0.1635584	0.1535142	0.141778	0.1852957	0.3940195	0.2979467	0.6755096	0	0.02390948
12.57968	8.089829	1.736274	5.786048	4.525531	4.67891	8.283072	10.13372	18.75035	30.87892	15.87116	7.157117	4.165212	18.09618	9.192594	21.83817	9.771122	17.11556
2.36677	0.9939233	1.064495	0.7703647	1.147293	1.755148	1.117682	0.8465527	1.693471	3.247526	1.987496	5.148074	0.9936098	2.189412	9.853174	1.73341	1.368526	2.261045
0.3402278	0.1974378	0.0157062	0.006623291	0.04846097	0.08919908	0.06390242	0.04261711	0.3422862	0.263064	0.08469558	0.0756973	0.07764289	0.1376772	0.05479354	0.2990987	0.02353209	0.04616899
33.18035	10.25318	3.44497	10.50879	4.693475	10.14041	15.09325	36.57514	28.32971	86.31472	31.10236	27.67218	10.66037	42.16969	40.24965	46.67049	18.13223	48.58524
8.840699	7.66154	5.865223	6.220591	5.753703	10.1774	5.25561	5.213717	11.24798	9.885531	25.95056	5.823615	2.623308	9.084305	10.77896	5.685317	3.9974	14.43544
0.5759453	0.271381	1.500312	0.4217869	0.3013561	0.8987989	0.3240971	0.1104233	0.958052	2.21674	0.7652334	0.3742563	0.194024	0.6191652	0.7978098	1.243797	0.1233008	0.9011854
3.091158	84.73498	1.158584	4.129237	5.633975	2.817184	2.432944	1.854346	14.8116	26.0871	30.43213	2.906024	11.89823	19.65659	22.32825	6.959041	0.8959324	12.96364
23.16409	9.409748	2.876617	2.932751	10.86443	5.816661	3.790395	4.046283	4.234302	27.8378	4.822799	34.39108	7.828382	8.769647	32.62711	8.612588	2.014973	3.531477
5.656104	1.256625	0.6691664	1.555708	1.436307	1.970773	1.957099	2.070588	1.737771	4.628219	1.621199	9.991271	2.43545	3.844295	4.471059	3.778099	0.7236086	2.047677
2.550244	4.404026	0.3137709	0.1443457	0.4903514	0.4270851	0.4738937	0.9730102	3.844952	2.66657	1.948372	2.40126	1.720324	2.868889	0.9329307	2.336641	0.1923188	0.3473754
0.04521734	0.008810777	0.01913453	0.02305432	0.01204877	0.0423387	0.03707188	0.03602579	0.08163492	0.02981258	0.0294808	0.04567106	0.03783627	0.1639459	0.09536257	0.06940677	0.02457312	0.02295783
115.8782	21.80528	7.365891	39.90364	33.9769	72.37301	85.45969	81.02322	156.992	515.5966	198.2687	79.39864	29.43912	160.7489	82.20524	250.0449	101.043	164.4545
1.272148	0.2309369	0.04110902	0.1584972	0.06212597	0.2850122	0.1314159	0.4826026	1.810743	3.051523	0.6967088	1.056684	0.2554773	1.414318	0.6402466	1.217772	0.2287714	0.6954554
5.488172	3.133567	0.1914891	1.08703	1.766007	1.490573	2.197446	3.597129	5.04105	7.315309	2.627843	3.535405	1.888381	4.226963	3.115377	5.253944	1.213816	3.451564
0.08291562	0.4252374	0.007017447	0.03551101	0.23508	0.02898449	0.09992942	0	0.1529317	0.6122785	0.0567623	0.04509487	0.1387617	0.1424522	0.07956475	0.05090884	0.01051401	0.02946865
0.4389578	0.685057	0.1425545	0.2841806	0.6055042	0.3746907	0.7206048	0.09042233	0.7949805	0.4139273	0.3261255	0.5163308	0.4270981	0.5261486	0.05651407	0.1645282	0.2718353	0.2938754
0.3431646	2.06619	0.07260813	0.05248934	0.5669331	0.214212	0.0773704	0.1929938	0.4018676	0.008080515	0.01864471	0.06665538	0.08887914	0.1244222	0.1447456	0.1191444	0.4817684	0.200367
0.01687695	0.07596527	0.02142535	0.0180701	0	0	0.007264285	0.008305059	0.1642881	0.02503635	0.08665204	0	0.04942711	0	0	0.01295271	0.01605045	0.008997235
0.03704022	0.07638932	0.009404542	0.003172709	0.01658138	0.01165321	0.01530537	0.002916369	0.09716327	0.04542356	0.01183325	0.02900861	0.03471322	0.03297529	0.01476415	0.01023394	0.002818104	0.00315943
0.2139376	0.02626255	0.006789866	0.04008593	0.02394275	0.0280445	0.02762532	0.00526388	0.1205698	0.132237	0.6712623	0.008726482	0.02685232	1.271817	0.4026877	0.01641928	0.02034607	0.005702593
0.7297273	0.2522927	0.0297238	0.02924716	0.05240674	0.0613848	0.1041381	0.1689859	1.123605	1.636329	0.6366891	0.222843	0.1469382	0.8365107	0.1252995	0.6049747	0.0445342	0.1123383
0.0687997	0.03378285	0.0174683	0.007366366	0.08469664	0	0.07699435	0.02708479	2.072633	0.2041238	1.23242	0.03928861	0.06908299	1.950309	0.09141134	0.1425664	0.01308609	0.05134874
0.06173221	0.1431423	0	0.005508043	0.005757282	0.01348718	0	0	0.08434107	0.09157754	0.005869525	0.008393491	0.0387415	0.00602604	0.04556725	0	0	0
0.343623	0.03891046	0.06584615	0.04165089	0.03627967	0.06515887	0.055813	0.01701586	0.09079407	0.228694	0.1898664	0.1710168	0.06148474	0.1316406	0.08614297	0.0464419	0.03699568	0.03917231
0.9235876	0.5047382	0.3661622	0.4566601	0.3914744	0.3298269	1.315436	0.8969059	2.103421	3.896391	1.344359	3.499458	0.3312109	2.573511	0.5605673	1.749715	0.6653342	0.860424
1.747901	0.9972919	0.1797051	0.2240496	0.1446455	0.2662399	0.4132588	0.2362338	1.929783	1.034745	0.9550143	0.5824231	0.6076926	2.119568	0.7359606	1.284796	0.2926591	0.5971523
6.744287	6.229402	0.8526372	1.528114	2.223639	2.824687	1.879068	4.241491	5.849755	12.59262	5.268358	6.369498	1.803072	6.851201	5.329417	8.397742	2.661414	4.714344
4.938849	3.745106	0.3288403	0.9244776	0.7343959	2.34671	0.8423961	2.787292	4.022642	4.668079	9.050239	1.760969	1.859213	3.533224	5.946376	2.067527	1.291945	2.853886
0.2834188	0.277327	0.03910884	0.03958119	0.06895374	0.07268989	0.1538147	0.04244708	0.2714738	0.1096805	0.2249538	0.100527	0.1340441	0.2670386	0.04093119	0.09930177	0.1640674	0.2036469
10.49487	1.493195	0.722681	0.847841	1.257841	3.099728	3.402087	3.914653	9.875177	20.4155	8.68926	7.418533	2.012226	12.80652	5.159542	10.65357	3.255249	7.773715
4.871228	0.3893614	0.3380345	0.7148406	0.4952033	2.225194	0.5275473	0.6262537	1.15959	4.449631	2.953194	2.047662	0.5013175	1.821	1.838299	1.487614	2.27165	1.795278
107.8721	13.27646	7.664126	34.25077	26.97811	70.64072	84.13023	68.44912	144.52	561.127	161.1991	96.80449	25.78198	134.1654	72.65881	230.2508	86.91898	147.3463
0.185705	0.02922663	0.03853658	0.139566	0.02198213	0.025748	0.06456073	0.07908271	0.2744542	0.2436998	0.1731735	0.09468595	0.07321323	0.4350658	0.1265326	0.2932721	0.1086835	0.0837699
0.8401997	2.765381	0.04637552	0.2151216	0.3679461	0.3830939	0.2987496	0.1438115	1.197825	0.7044906	0.6043591	0.402319	0.6419147	1.433511	0.5864824	0.4625998	0.1042244	0.2531706

TCGA-P5-A5F1-01	TCGA-HW-7489-01	TCGA-HT-A4DV-01	TCGA-RY-A843-01	TCGA-QH-A6CW-01	TCGA-E1-5311-01	TCGA-CS-6290-01	TCGA-DU-7301-01	TCGA-WY-A85C-01	TCGA-S9-A6WN-01	TCGA-S9-A6U5-01	TCGA-HT-7476-01	TCGA-06-5417-01	TCGA-DU-6395-01	TCGA-HT-8019-01	TCGA-E1-A7Z3-01	TCGA-DB-A4XE-01	TCGA-P5-A5EZ-01
0.4893072	0.08631063	0.05394018	0.03946683	0.05756964	0.02829655	0.1071763	0.3136447	0.07579865	1.860286	0.6870711	0.05207287	0.3349304	0.05031901	0.01643002	0.05356183	0.006417034	0.1838312
0	0.1818206	0.1439307	0.1238952	0.04042509	0.5932521	0.09976141	0.2479736	2.696754	0	0.06866439	0.2315802	0.3191814	0.1751325	0	0.5464633	0.04280707	0.1486437
0.7946616	0.4261775	0.364766	0.4223382	0.6060421	0.3118632	1.614294	0.7877671	0.6593779	2.415907	0.8235184	0.956513	1.276022	0.3959146	0.2347323	0.6421905	0.3942191	0.8568829
0.1652434	0.06164288	0.0430562	0.007412533	0.1402785	0.05485392	0.2029335	0.2781755	0.3025205	0.5589783	0.02875688	0.05937954	0.4010228	0.2584576	0	0.04526915	0.1024443	0.08893211
50.96819	29.09522	15.70366	6.904407	15.13177	7.024932	27.10528	14.01673	23.19899	13.15658	14.14197	27.23251	9.59013	10.71024	8.199918	12.16434	5.360288	11.09052
1.81258	1.425267	1.096702	1.278385	3.500117	1.534921	3.883687	5.924279	1.616039	2.982415	3.095591	1.848586	3.069016	2.254528	0.6874199	2.852166	1.557096	2.786024
2.842642	1.494723	1.817843	1.887269	2.118168	0.8514504	3.870764	3.608036	1.58793	2.404084	3.492359	3.032136	1.661531	5.430164	0.7910134	3.493958	2.929752	1.864682
2.385674	1.407484	1.572343	1.452066	1.503249	0.8193748	3.820397	1.551809	1.536415	2.564393	1.867825	2.273755	1.870421	0.8404564	1.200725	3.132359	3.152681	3.150872
0.1477973	0.08918851	0.04236143	0.009116152	0.1070806	0.03174634	0.07829759	0.4470216	0.1785833	0.09445851	0.0909413	0.09736895	0.1409114	0.1975879	0.02764964	0.08660296	0.03779669	0.1312456
2.782469	1.789295	2.050732	2.725426	3.328742	0.9138037	7.356733	3.192955	1.34136	5.485951	2.234302	1.643413	1.956336	1.292608	0.6712742	2.436171	2.351753	4.18091
0.173323	0.01255105	0.02980655	0.01710493	0.1116215	0.01116875	0.01377303	0.1540582	0.01861565	0.06400174	0.09005796	0.04110672	0.1101652	0.1410426	0.02161663	0.01741028	0.01181986	0.03591298
47.56536	24.97638	35.06233	41.24967	55.00322	8.476974	147.8416	63.88968	33.57058	274.8181	25.68313	36.89544	25.124	39.42251	10.63488	53.50273	52.08827	65.36183
0.7292157	0.4623771	0.6363786	0.7894664	0.7605059	0.2127403	2.312105	1.088334	0.4851223	1.227337	0.6339763	0.5564126	0.4565764	0.5845467	0.1547454	0.8600456	0.5974935	1.085696
0.2225403	0.1474939	0.123244	0.1005045	0.4117352	0.1118054	0.5125379	0.1117542	0.2673761	0.9321119	0.3218277	0.4920122	0.2876909	0.3893728	0.03669298	0.2879518	0.5054467	0.3282472
1.175208	0.9245276	1.020581	0.9478697	1.286132	0.2968786	4.613522	1.729406	1.402705	1.726749	1.025019	0.787089	0.7295986	1.206422	0.1840653	1.156964	1.218136	1.844499
0.2130411	0.173556	0.05724518	0.04434885	1.942242	0.09867106	0.4073591	1.41035	0.1608859	0.07375139	0.5134224	0.0789478	0.177726	2.851209	0.07472875	0.4681241	0.08172258	0.1832709
0.1115725	0.01731306	0.04111551	0.005898687	0.1693692	0.02054175	0.1139921	0.3482798	0.01925899	0.4074681	0.1503801	0.2709148	0.1823559	0.2668194	0.01192729	0.04002658	0.04891337	0.07784666
2.394785	1.255774	1.70414	6.566755	5.85827	1.024349	6.254486	3.712061	1.313769	12.31899	1.482006	1.954427	3.700377	1.32478	0.7145017	3.509835	2.909026	2.452001
228.6222	156.3752	238.0925	207.469	167.3703	19.10125	630.239	375.3006	162.961	855.7751	379.928	147.2732	211.1098	58.21871	49.14735	183.7767	120.5297	393.711
0.6663189	0.21522	0.1813616	0.390289	0.3241517	0.1297374	2.125567	0.8699227	0.1583195	2.234437	0.5269939	0.35623	1.115159	0.1036517	0.0573945	0.7174689	0.2500834	1.089747
11.40614	2.776816	7.603018	5.058753	8.527157	4.753032	38.81257	42.48818	21.59821	48.68172	9.166541	12.661	23.14016	10.28438	0.6470443	19.84852	7.360588	15.66389
0.07530449	0.05063606	0.01850028	0.05175622	0.09352942	0.03812712	0.0854862	0.09562057	0.09099035	0.2291797	0.08384552	0.1063085	0.1025657	0.04126984	0.1167277	0.08104637	0.06602698	0.1050833
2.109641	1.735704	1.106403	1.528252	4.382294	0.9544938	6.448851	3.056546	2.564746	3.312394	2.764339	2.164591	2.78451	3.116209	1.576431	1.298532	1.576428	2.484559
11.84257	5.377347	8.117445	7.857325	14.1535	2.767048	24.12181	9.747606	7.615016	15.99576	12.30105	7.968649	5.639252	10.88605	2.488495	10.15104	6.446793	10.80303
1.662364	2.138587	1.897424	3.239374	4.224278	2.647216	5.59667	2.626105	4.589046	8.283203	1.747028	3.709998	1.641017	2.085574	1.431113	5.456545	1.602672	1.81912
0.6578414	0.1767181	0.3466878	0.3691082	1.014723	0.1572555	1.138247	2.923612	0.2649564	1.989141	1.061996	0.2013149	0.6878883	0.7252752	0.05557897	0.4050071	0.4124397	0.3486178
14.1747	7.941508	12.9311	8.946636	13.14754	1.548981	37.91779	25.5608	8.737632	35.95968	20.40186	9.691092	10.57002	6.652937	2.214881	12.31365	7.613036	19.94063
10.41828	5.26018	6.453876	7.566358	19.02745	5.003547	19.01388	22.29377	9.486509	24.0782	5.562837	7.144181	5.378029	13.77481	2.110555	9.534276	5.540571	9.435558
0.02680301	1.062653	0.1382803	0.06943493	0.02589205	0.4490611	0.1064944	0.1439358	0.3238606	0.2341105	0.2143985	0.1324338	0.1788797	0.08412854	1.273621	0.2625051	0.1987783	0.06545376
0.4225898	0.2781954	0.1734247	0.1812729	0.6912028	0.09283378	0.7937301	1.447698	0.2437026	1.129429	0.4294338	0.2695442	0.4578422	0.5325749	0.06468324	0.221893	0.1866669	0.8784598
0.077715	0.06029646	0.04773119	0.04519557	0.02681203	0.02146231	0.03528905	0.1356869	0.04024413	0.0851457	0.1229629	0.05266152	0.2540374	0.0309752	0.1620035	0.03903232	0.01703513	0.2563295
0.8626637	0.4939417	0.5347616	0.3341042	0.6395433	0.0711024	2.1761	1.812926	0.5332984	1.444585	2.164374	0.499596	0.7650902	0.6227022	0.1351135	0.3425875	0.3386136	0.7571473
4.695529	3.829385	5.487832	4.534111	7.139705	2.272984	22.79069	9.653189	4.392912	7.473248	4.768601	4.338293	5.085255	9.404146	1.5735	5.466511	7.070751	7.625862
0.4699404	0.2754838	0.4361508	0.6018041	0.8358785	0.2211101	2.169484	1.265275	0.1502192	2.053138	0.4467438	0.3007514	0.4551576	0.6711804	0.1060569	0.3671536	0.381522	0.8015045
6.457484	1.61523	2.543131	2.508367	4.857078	1.191165	8.86245	5.002186	2.739835	4.515584	3.225196	2.552517	3.712775	4.349405	1.03284	3.862212	2.634667	5.001112
2.046442	1.926489	2.049985	1.018634	2.495823	1.031887	3.130425	1.602976	1.693809	3.522607	1.401907	2.762456	1.190169	1.106238	3.158467	3.10403	0.9681853	1.89904
2.986182	2.115297	2.101398	1.937319	4.819459	1.076284	9.399889	3.577585	3.319895	2.76647	2.343132	2.812332	5.156409	8.386983	0.7683119	3.544342	4.459959	3.377305
2.190157	1.572429	1.856392	3.117441	2.724516	3.276983	3.039497	1.451259	2.543325	7.864111	4.597035	4.114048	1.237402	1.301327	1.671607	4.086628	0.8872177	1.446195
0.6220754	0.4548023	0.5966136	0.6773724	0.4969252	0.3314794	1.468728	0.824081	0.5589216	0.8716039	0.5152671	0.3310114	0.707149	0.1794012	0.4252225	0.6248741	0.7648338	0.6586424
6.97784	4.15448	3.43861	4.770068	5.413348	1.836903	15.43692	13.73975	5.5548	21.23743	17.17417	4.863841	10.5388	6.39765	0.9591486	4.295151	4.279573	8.782405
2.565484	2.347597	2.139646	2.544799	4.100013	2.276497	10.29072	4.890872	3.015176	8.150328	2.938614	4.713003	2.967226	4.370921	2.011576	4.390097	2.721118	3.597778
2.391764	1.400166	1.766487	1.375237	2.928223	0.6716508	6.702128	3.53201	1.439913	2.557316	2.073776	1.35344	1.17137	2.054603	0.6480912	2.357003	1.813071	2.830393
1.033048	0.8779044	0.6900626	0.9668363	2.647422	0.517144	3.588922	2.447335	0.8596633	3.543052	0.9876151	0.7964389	1.693082	3.04301	0.2108298	1.62944	1.152807	0.917358
2.173934	1.250351	1.055377	1.278011	1.346443	0.3284314	3.893088	2.057116	1.600357	3.421376	4.360683	1.019664	0.7933589	1.538095	1.206465	2.131473	1.149136	2.669416
0.08817736	0.05321077	0.06318316	0.01631637	0.1277707	0.04735047	0.1576568	0.04354247	0.1894127	0.200373	0.2351115	0.08132783	0.1120922	0.08200553	0.04398948	0.05166821	0.03758315	0.1631301
0.3287705	0.07142295	0.01884634	0.04866864	0.07410586	0.03530938	0.6618482	0.2597579	0.05296708	0.1214027	0.08091845	0.1646116	0.1253812	0.03057587	0.008200769	0.1431082	0.1569448	0.4963181
0.05874508	0	0	0	0.02837425	0	0.02334074	0.04351297	0.07098163	0.07508897	0	0	0.2240325	0	0	0	0	0.05216628
0.3280154	0.2173144	0.1320215	0.1962937	0.1415789	0.1169279	0.6544126	0.1395749	0.05621855	0.184362	0.1603201	0.09931256	0.4524649	0.04867923	0.03655758	0.217324	0.1998949	0.2231091
36.95193	20.27483	16.02996	11.89295	52.19505	9.939331	58.98308	108.1884	19.36873	16.96358	23.97927	15.16519	25.64198	138.0661	4.971266	30.71053	21.1747	35.17141
5.996291	3.780892	6.781824	4.501266	7.199479	5.060454	8.088187	6.283718	7.903979	8.012952	6.624481	5.077112	6.626433	5.340932	2.147944	4.988019	6.334201	6.30804
9.312439	6.004511	7.859271	5.330045	16.35289	3.074402	32.16756	11.86564	6.378823	28.79353	12.42557	7.102361	6.032562	12.91309	2.405604	10.47878	10.47534	10.36526
3.57975	1.977235	1.965595	1.938726	4.769243	1.273573	14.84759	5.6441	1.771084	4.423513	2.656867	1.86994	2.572969	5.159877	0.7008759	2.375975	2.817429	4.031722
135.4808	54.07628	52.98643	50.76951	114.1998	40.36777	250.7173	165.3343	73.15427	115.4783	60.31486	61.51513	75.58402	81.66101	14.49954	88.96373	55.92326	78.32914
1.301308	1.380625	1.115219	0.9727774	1.432487	0.5488179	3.088492	2.091096	1.577593	11.649	2.887164	2.043855	1.599281	2.270679	1.578761	2.28862	1.614187	1.00585
1.046802	0.7718789	0.5260851	0.6049828	0.9004054	0.47434	3.433699	2.01456	1.035753	3.209669	0.7176375	0.7141964	1.604142	0.7934961	0.168113	0.8978663	0.8165531	1.426193
1.844485	1.090592	1.338635	1.290067	1.275047	0.4361714	2.622144	2.093208	1.676629	1.917846	1.860317	1.551822	1.677883	2.124558	0.5445029	1.376839	0.917428	1.65295
0.3016671	1.310699	0.4817245	0.5263083	0.270599	3.124143	2.277324	2.13871	14.56281	1.39549	0.7778324	0.6813891	2.876123	2.735386	0.6933481	2.142815	0.6171707	1.339418
0.09290944	0.01681991	0	0.01719199	0.03365689	0.01496747	0.1891895	0.1247341	0.08419681	0.6977059	0.4239975	0.0688599	0.02214519	0.0445533	0.004345331	0.03499779	0	0.7992634
177.9033	114.2828	133.7059	120.5714	94.18181	11.31658	529.9523	416.3345	90.79971	1203.848	335.5633	106.7176	209.5763	30.73565	22.15197	138.0297	59.78335	245.4594
0.03526999	0.3115938	0.0970467	0.02088438	0.08858528	0.4909164	0.1345302	0.1828734	0.3636625	0.5349814	0.04629763	0.1338387	0.3497182	0.2214089	0.01055719	0.1133717	0.09380501	0.3445215
1.557221	0.6968198	1.245568	1.07984	1.914104	0.2000247	3.831536	2.283902	0.5542666	3.090404	1.409144	0.6503037	1.341629	1.266596	0.2748686	1.507063	0.8943714	1.966283
1.471802	0.7036309	0.3931767	0.6240087	1.145706	0.5248505	3.009064	1.418288	0.7942269	1.680369	1.230935	0.9941004	0.7629215	2.24255	0.2780152	0.818157	0.8477887	1.383111
3.696216	2.489575	3.372513	2.544649	5.394869	1.461324	8.991105	7.765122	3.386981	5.302243	3.522386	2.647739	4.985933	4.446991	0.706579	3.19402	3.252636	4.314264
73.34786	25.87352	35.42854	30.60293	63.37469	14.55361	141.9439	67.85419	30.18699	76.47402	66.80303	33.02686	39.1921	57.8452	10.80216	39.01157	33.19997	63.69054
0.1783062	0	0.04380501	0.01885362	0	0.03282818	0.06072433	0.05660266	0.02051878	0.04341219	0.06269357	0.02013741	0	0.01776706	0	0.02558691	0.02605648	0.1130984
1.271062	0.299253	0.5276213	0.2456253	0.5988098	0.09683459	1.776283	0.3571156	0.4035004	0.3148011	0.6318433	0.3019506	0.2626655	0.2795105	0.08902406	0.6666934	0.3778942	0.5170971
0.1538132	0.06993792	0.1476359	0.07678027	0.2626163	0.1751811	0.2728787	0.4928167	0.2478032	0.6065851	0.6544305	0.6164772	0.09549108	0.1996009	0.07227232	0.2299613	0.04024995	0.5209397
7.572501	7.182409	7.597144	9.277271	16.54929	5.659895	25.2312	18.66285	9.525137	16.89595	9.34411	9.123744	7.630059	12.8104	2.962487	9.758835	8.457399	12.23928
1.242627	1.399748	2.119151	1.451152	1.937302	0.95421	3.033658	1.805054	1.557075	2.861966	1.41593	2.125744	1.066259	0.984711	1.211418	2.64206	1.115765	1.673591
0.1657741	0.04001466	0.05430162	0.02921418	0.1143856	0.04578132	0.2070067	0.1110959	0.04451215	0.2959807	0.3885814	0.1185731	0.06020976	0.04955496	0.02362873	0.03964759	0.03230016	0.5257472
25.18482	14.98071	13.68925	15.06246	24.13336	10.12853	66.40025	39.20799	15.23351	27.76532	27.46939	14.41707	31.81517	36.87404	3.5673	15.96944	15.98649	29.7726
7.513784	8.265896	8.519066	7.382405	18.62778	5.255999	8.920961	4.917033	11.70348	15.49712	8.773842	6.746135	5.240711	6.640021	6.866609	8.616274	5.520842	4.271074
0.244991	0.8939535	0.585462	0.3320511	0.424152	0.3341916	1.385521	0.9968897	0.8560304	2.423876	0.5977622	0.9533096	0.2669449	0.2995991	2.007105	1.057881	0.4100883	0.5198711
1.502734	5.114505	2.196628	1.418138	2.032324	12.68533	9.254585	13.27357	36.46635	5.922529	2.835589	2.702705	8.453053	11.97528	2.389593	6.037978	2.190505	4.437027
8.121303	4.374902	4.984253	2.81063	5.644974	3.067339	13.55753	6.656576	4.718005	83.95247	19.39681	10.70329	3.722746	3.874704	2.507722	8.231972	5.675626	4.349127
2.380602	1.10973	0.5733539	0.8831795	3.904392	0.9762042	3.54642	11.20821	2.407668	28.43554	3.647031	2.432279	1.851442	25.36417	0.398306	2.896602	1.035109	1.65692
2.809973	0.2803067	1.577907	0.350176	0.8448096	0.3787725	2.130399	1.051305	0.9007907	1.930263	6.304408	1.416744	0.546746	1.529977	0.1609238	0.5472417	0.19065	11.1906
0.01923419	0.03183608	0.004725322	0.0142364	0.03185225	0.01239431	0.03493567	0.05698771	0.006640188	0.009365895	0.1082058	0.06733996	0.05239447	0.07091285	0.09663996	0.005520208	0.03091833	0.04636047
132.2374	73.78261	76.03608	85.41404	216.586	45.59868	338.7031	194.1307	115.0103	149.5877	104.3732	69.8314	85.5784	111.3313	22.53946	89.16926	89.72605	97.68868
0.5253941	0.1538939	0.2436475	0.1966229	0.2908361	0.1065128	2.298606	0.4241448	0.0855955	1.338105	1.017063	0.3406852	0.3376961	0.6711662	0.03534013	0.391371	0.2355088	1.761379
2.731827	1.479652	2.215295	2.082269	2.967958	1.922562	9.330463	3.701501	1.705596	3.842001	5.873361	1.867036	2.42809	3.144788	0.62602	2.320229	2.067459	2.853217
0.01410799	0.1685672	0.06065418	0.02610548	0.1022138	0.1909119	0.08968679	0.2089982	0.6250449	0.5169483	0.1157441	0.05576614	0.1883098	0.03444139	0.01583578	0.1133717	0.01443154	0.03132014
0.5471342	0.4622366	0.3584429	0.3856834	0.3146063	0.6463738	0.2794998	0.2315811	0.6715955	0.8214661	0.5985026	0.2883619	0.04968028	0.3725424	0.253455	0.3271403	0.1732348	0.4280205
0.06255969	0.1887587	0.03586153	0.123478	0.1410114	0.04031281	0.04971275	0.03089233	0.2855654	0.07996488	0.0513249	0.651187	0.2385801	0.1309071	0.1014308	0.02094705	0.2666433	0.4629475
0	0.0155958	0	0.03188163	0	0.01387818	0.02567134	0.04785779	0.05204615	0.2202311	0.03533845	0.008513132	0	0.01502212	0	0.03245076	0.03304629	0
0.00756282	0.01506053	0.02601173	0.008396561	0	0.1230534	0.0150244	0.163854	0.0060921	0.009666927	0.03102325	0.0254102	0.01442093	0.006593881	0.004244515	0.03228659	0.01353846	0.05876384
0.1365046	0.004942436	0	0.01515532	0	0.004398107	0.02711818	0.7128264	0.06047742	0.1454021	0.07839329	0.04316608	0.2342607	0.009521265	0	0.04799157	0	0.1636438
0.1344538	0.1550606	0.1884028	0.03685832	0.2116629	0.109103	0.8982728	0.4352497	0.2446934	2.075064	0.257385	0.1220411	0.5507401	0.1563036	0.05216993	0.2801217	0.1630071	0.1636173
0.1053557	0.02543083	0.09059067	0.04548846	0.07633122	0.03960256	0.2302311	0.5527681	0.1131566	0.9202277	0.3169297	0.1179943	0.3348238	0.05511459	0.01970975	0.08819141	0	0.1169463
0.006564789	0.004753839	0.02257908	0	0.006341671	0.02115141	0.01565003	0.01945038	0.03172892	0.1286655	0.0215434	0.02594933	0	0.01373692	0	0.006594324	0.006715342	0.005829605
0.1158309	0.03195354	0.0426848	0.04490807	0.04262636	0.01599434	0.1271091	0.04902683	0.04443125	0.7144343	0.1900588	0.07848978	0.08414045	0.06540358	0.04746638	0.0664869	0.08745495	0.1224514
1.491855	0.5103851	0.6599079	0.4434244	1.91397	0.2447496	1.474869	1.644493	1.078726	1.85186	1.288189	1.204167	1.11996	1.693327	0.5157004	0.7355173	0.8811946	1.22047
0.7696882	0.2445575	0.6888338	0.3720447	0.7814639	0.2834166	1.1234	0.9017118	0.4428637	1.311772	3.376397	0.533977	0.5990434	0.5916431	0.1234216	0.504915	0.1928179	1.987705
7.606568	1.758498	3.74623	1.982425	2.518343	1.426755	10.58501	9.813645	3.48079	43.27351	5.888961	3.529023	6.400981	4.558349	0.9085965	4.268798	2.849385	5.391088
2.18165	1.239391	0.8084703	1.859436	7.883619	1.382165	3.72411	19.71262	2.786973	8.681975	1.566879	1.631812	2.465193	7.521457	0.5496822	1.933212	0.6086408	2.465692
0.1887002	0.05693572	0.02704248	0.1862248	0.1139293	0.3445232	0.1686934	0.05823826	0.2723408	0.1473999	0.2709217	0.1181	0.08995429	0.05484138	0.02941806	0.0868767	0.03217129	0.4049556
3.860404	4.144325	4.292383	3.408795	4.035093	0.8011944	18.14317	11.42739	3.308491	13.31325	7.006659	3.512059	5.931495	3.712545	0.8258658	5.220257	3.205831	7.807258
1.481607	0.5807739	1.392127	2.908931	4.344439	0.7341615	5.511515	0.9475356	0.9016533	15.25485	0.9265102	0.4839268	1.553122	0.9288655	0.2954054	3.342967	1.198667	0.974005
118.4852	70.05392	72.99608	64.96941	167.757	43.93804	316.9659	190.9505	117.1806	147.9943	80.5893	69.39335	97.64221	101.6264	19.52411	70.84519	70.58027	80.44812
0.05924527	0.05115237	0.06661695	0.09782159	0.08144511	0.04845547	0.2897167	0.1670948	0.1468431	0.281554	0.08225579	0.07205691	0.09559003	0.07311146	0.01705153	0.0709564	0.1468481	0.1133148
1.095501	0.2869377	0.4409233	0.1897728	0.5178759	0.2553362	1.407673	0.5697392	0.4318431	0.8739387	1.740162	0.681792	0.5333419	0.3739298	0.1220945	0.3043742	0.1907446	2.587279

TCGA-S9-A6WO-01	TCGA-S9-A7J3-01	TCGA-RY-A847-01	TCGA-E1-A7YD-01	TCGA-DU-6407-02	TCGA-TM-A7C5-01	TCGA-VM-A8CA-01	TCGA-DH-A7UU-01	TCGA-HT-8108-01	TCGA-HT-7687-01	TCGA-HT-8564-01	TCGA-HW-8320-01	TCGA-HT-8113-01	TCGA-FG-8189-01	TCGA-DH-A7US-01	TCGA-S9-A7R2-01	TCGA-HT-A74J-01	TCGA-26-5133-01
0.09263335	0.03147973	0.2175792	0.1727561	0.04038365	0.02266355	0.1156003	0.008718872	0.04526046	0.4575016	0.121025	0.08655468	0.03237487	0.01738108	0.1035124	0.5666478	0.1083458	0.1593664
0.7176113	0.4199931	0.04268935	0.1646328	0.3315609	0.1007901	0.4712596	0.03877485	1.74446	0.2322116	0	0.280894	0.6838982	0.1545954	0.03634292	0.08446977	0.2891034	0
0.7172157	0.5220564	0.6399873	2.283021	1.076979	0.3885474	0.896123	0.5148676	1.26691	0.7838742	1.129182	0.5882154	0.2826591	0.2703929	0.3917683	1.510572	0.3637599	2.380052
0.1001794	0.07686148	0.03575689	0.2560955	0.2082882	0.06030176	0.2768238	0.05567671	0.09232677	0.1071746	0.007886099	0.1083029	0.5211532	0	0.004348725	0.7681696	0.05189036	0.1221213
19.13889	14.92896	12.85511	19.48226	14.63703	6.530387	40.87478	7.875341	11.9041	12.09427	9.480575	7.858236	10.36786	2.854455	26.49985	22.47317	9.232339	25.13976
3.10465	1.684139	2.395052	2.951304	5.120425	1.033123	2.83497	1.902843	3.358038	3.137022	3.107207	2.028485	0.7460702	0.5416525	1.081308	5.398063	1.760326	8.669484
2.755769	1.931211	1.719502	1.49626	5.941877	0.7138993	3.729141	2.011846	1.929561	1.460753	3.352026	3.121538	0.9399405	1.226932	2.28885	3.424018	2.448639	3.389424
2.353551	1.422732	2.699279	5.854054	2.860477	0.6708452	2.324586	2.008537	2.786225	1.415972	5.802663	1.350262	1.348914	0.7940937	1.49343	6.306636	3.142566	1.655148
0.08213569	0.02181381	0.1130783	0.09690887	0.0487922	0.08899306	0.1260912	0.02282429	0.2073452	0.08787099	0.04849279	0.07348641	0.1165327	0.02275012	0.1176602	0.2361794	0.01063605	0.05006279
3.876429	2.3245	2.748039	2.501548	3.739909	0.5821873	2.317915	2.575686	5.931734	1.67983	3.705327	2.433765	0.4689606	0.5729953	1.544117	7.950342	2.630139	1.694853
0.0165122	0.02728664	0.03536208	0.02841145	0.0514971	0.02087257	0.01774417	0.01070649	0.05557837	0.05953827	0.01364829	0.02154452	0.0496941	0.00533585	0.05017493	0.02332374	0.009978383	0.03522542
86.05683	28.56527	78.74468	17.98679	111.7304	7.43518	32.05695	55.1166	119.8313	22.42872	64.7422	54.8898	8.794242	13.6256	17.59139	160.0159	38.15818	30.22186
1.209697	0.4626353	0.851215	0.6601157	1.174932	0.1660211	0.6796894	0.7630762	1.753746	0.5377982	0.8170486	0.7900866	0.1060984	0.1407271	0.5954886	2.306767	0.8365008	0.5972344
0.3844936	0.1870514	0.2116269	0.2077462	0.2353436	0.05450767	0.2046595	0.2201807	0.8738644	0.1973415	0.6118512	0.208171	0.1492397	0.07315508	0.26861	0.2398283	0.1237757	0.08049072
2.495857	1.599679	1.860016	1.286416	1.856072	0.3855511	0.7674482	1.544751	2.976586	0.8356607	2.050672	1.595735	0.2048557	0.2920801	0.9494195	2.904151	1.571193	0.8173075
0.1014804	0.2279642	0.08149786	0.2291764	0.2571481	0.1282783	0.4089443	0.1603869	0.3308984	0.147771	0.1572739	1.241325	0.05153778	0.03074341	0.01734548	0.1478221	0.160978	0.169131
0.04555425	0.08939385	0.04877886	0.1489261	0.08682141	0.0959729	0.1142238	0.02215299	0.08305424	0.03158761	0.1192352	0.1545376	0.03427431	0.022081	0.02768475	0.1126057	0.06882149	0.08908231
3.545133	2.845906	1.711132	4.081248	4.825316	0.5718157	1.500605	1.975062	10.69958	2.876217	4.019452	2.659858	1.167546	0.4242356	0.5737336	10.18354	2.085881	1.688785
157.3426	109.9599	217.6653	243.8603	533.4777	47.84756	246.6495	111.0751	367.0616	160.2009	341.6948	148.6766	29.27956	29.23162	101.7376	813.8164	137.2622	241.2592
0.3607805	0.3339446	0.3423078	0.9853759	1.305585	0.1212289	0.3435301	0.3686612	1.064478	0.3154011	0.7813751	0.3146168	0.06184838	0.03541815	0.1790142	1.083724	0.3270315	0.9157881
22.53516	4.06606	11.48224	13.34808	30.2779	4.844251	11.72337	17.64698	16.78077	9.64383	19.21276	10.34061	3.156042	2.388802	6.640884	23.75962	5.902177	23.47169
0.06149258	0.08891519	0.093281	0.04761279	0.06392625	0.03886545	0.06608053	0.08971135	0.07761664	0.09380658	0.1270679	0.0922683	0.09253213	0.05464546	0.04671373	0.1357176	0.05109526	0.0437273
3.56066	2.57574	1.993352	2.460447	3.14738	1.106312	1.439848	2.046246	3.878993	1.923792	2.363427	2.530838	1.889232	0.9258333	1.177252	5.137206	1.454866	1.818404
13.12158	6.469243	12.46949	8.243461	19.0512	3.196009	10.55725	10.09953	10.00617	5.809787	7.545749	8.365236	2.382723	2.284093	6.869645	25.11012	9.133885	9.540712
2.242417	4.019012	2.671279	2.296941	2.699051	1.492579	3.015919	3.148776	3.275603	2.862971	2.678868	1.961217	1.999284	1.063165	2.143196	3.266432	3.077561	1.965818
0.2527075	0.2756178	0.3030672	0.6365722	0.8196516	0.3322175	0.2389745	0.4915657	0.4593173	0.4626046	0.3843344	0.4035818	0.06388476	0.009799368	0.6081702	1.274323	0.1924172	0.636138
11.40954	5.014938	10.09662	14.07588	29.75438	3.234749	17.34027	8.195847	26.27199	10.3753	18.05294	8.90653	1.97195	2.986425	9.167652	40.50046	8.768738	12.47421
10.74787	7.306024	10.90975	5.780051	10.40935	4.138201	12.87563	8.389351	9.685443	6.059453	7.301885	10.33727	3.208207	1.60012	5.763663	14.51734	8.156827	5.89587
0.210662	0.2808711	0.1708894	0.08567523	0.05309074	0.03227775	0.5762385	0.03104386	0.3867636	0.1168595	0.2216128	0.2098963	0.9567565	1.033496	0	0.04733963	0.0636519	0.03404579
0.9424368	0.1871136	0.6711326	0.321169	0.3424317	0.2371045	0.5752036	0.4138103	0.7622387	0.4339692	0.4424295	0.5336475	0.1982656	0.0887023	0.5254829	0.8966259	0.3732278	0.444066
0.01586524	0.01310877	0.1075925	0.05459655	0.07147026	0.0267397	0.03409789	0.02571751	0.03115045	0.06160595	0.07868125	0.09936191	0.2148617	0.1589304	0.03856724	0.1400619	0.01917485	0.1128175
0.3822538	0.3790078	0.6446665	0.8287256	0.7748951	0.4952753	0.6469684	0.2044786	1.101674	0.644089	0.6872018	0.4713196	0.2502136	0.1111714	0.8711538	3.938183	1.0828	0.5198561
10.82508	5.487018	7.568674	4.691062	11.00518	1.674429	5.104637	5.596871	13.85084	3.722223	7.93344	6.914755	1.298892	1.210469	3.855197	19.06532	6.038055	3.468598
0.9025155	0.2774393	0.6011472	0.3814995	0.7239916	0.2066093	0.1832791	0.2833793	1.787981	0.5262717	0.6813687	0.745485	0.1219062	0.1033385	0.2915187	1.912226	0.4058242	0.2653018
5.764507	2.117143	4.387417	2.921039	5.833982	1.068524	3.83535	3.722474	5.058148	2.61809	3.085894	4.338163	1.02819	0.8649961	4.152556	8.668981	3.203271	3.243408
3.299717	1.703506	2.604302	1.580001	1.798759	1.022745	2.650265	2.569335	2.345983	1.415411	5.188923	1.480482	1.945053	2.060129	0.9997091	4.368305	2.107429	1.79881
7.218986	2.578269	1.91037	1.728068	7.160963	1.14937	3.160775	3.249302	6.341224	1.639268	2.738629	3.208596	0.5037569	0.6210374	1.97801	7.47256	2.466371	3.335738
1.30185	4.954678	2.457009	3.46127	1.792137	2.096486	3.903091	2.740492	2.265978	3.462453	2.063665	1.023818	2.152198	1.141042	1.457713	3.702956	1.869789	0.9194029
0.9972325	0.3707866	0.7932398	0.505937	0.9063716	0.3097404	0.600116	0.6429093	1.256321	0.5168374	0.6829668	0.6870088	0.3447103	0.4861395	0.394798	1.334152	0.6198492	0.8144873
10.16933	5.602662	9.2626	5.456052	9.601954	3.093287	4.172818	4.707807	15.28957	7.079928	6.407104	5.705104	1.755156	1.302159	4.550567	21.63965	3.896195	4.933813
4.958306	2.915374	4.322509	4.305355	4.286771	1.865521	2.332228	3.091556	7.083053	3.500632	4.697206	3.230788	1.959482	1.485728	1.65276	7.081503	2.242702	2.26865
4.44206	1.527963	3.759995	1.753139	3.351191	0.6791448	2.031049	2.253481	3.495684	1.269316	2.55348	2.831574	0.619342	1.083551	1.705458	5.68667	2.35678	1.320123
1.691793	1.144158	1.437046	1.293136	1.428983	0.3906955	0.9352299	1.344826	1.396223	0.9204323	1.008436	1.693748	0.2203059	0.220781	0.8749211	2.765944	0.6930398	1.067112
1.238679	1.136049	1.888738	1.063952	2.019117	0.6638893	2.193647	1.14691	1.734415	1.500684	2.04768	0.8688605	1.002047	0.8741986	1.237578	4.0417	1.320422	0.7187517
0.1120068	0.09110039	0.1049435	0.1011794	0.1309946	0.06194319	0.1128409	0.08851182	0.2238458	0.1514492	0.1272977	0.1041265	0.15169	0.0610783	0.1021051	0.185404	0.1332571	0.1194719
0.07308333	0.06469887	0.1341542	0.1832352	0.1302441	0.01319749	0.05609718	0.1116982	0.1142101	0.1042487	0.1035559	0.2125087	0.0188526	0	0.1808327	0.154847	0.05678295	0.1113631
0.02798274	0.0173407	0	0	0.0872706	0	0	0.02721595	0	0.02328409	0	0.02190652	0	0	0	0.02964454	0	0
0.2194113	0.2760554	0.09967263	0.1441465	0.3525103	0.04202291	0.135753	0.1616659	0.889576	0.3208797	0.3242428	0.09889672	0.07803861	0.01289125	0.07273264	1.01429	0.1566985	0.1489311
32.88215	8.013665	52.586	17.13735	41.09307	9.799617	29.62861	28.6797	21.77256	15.78054	21.75768	60.27912	5.911901	4.288507	21.45084	29.3017	10.88825	34.29115
5.615195	5.249714	5.621676	7.975086	15.0997	6.790281	6.025423	5.327162	6.527659	4.800622	7.572268	6.35246	2.337064	1.832882	5.680977	8.18371	7.634886	8.343667
18.41204	9.937453	13.58278	9.620169	20.20199	2.350454	7.509445	10.70018	16.99145	7.149535	12.58926	11.61922	1.794023	1.888023	4.949449	29.16808	10.47099	5.930639
5.762087	2.549178	5.48981	1.452036	5.148997	1.481588	2.137132	2.853579	7.222067	2.246271	4.050169	5.173917	0.4505986	0.9603201	2.254113	9.701157	2.289379	2.13743
110.6271	43.47305	82.59376	65.51862	181.3351	22.94416	75.07261	77.5466	127.6399	55.266	61.05821	68.92587	16.24128	12.21353	47.16768	89.35102	49.271	75.74594
1.079105	1.222571	1.186362	2.891417	2.423456	0.4113015	1.996133	0.8933064	1.084794	1.816382	1.280039	0.9898828	1.959719	1.385516	1.903589	4.258597	1.209633	2.662294
1.93079	0.7760297	1.126378	1.118886	1.121952	0.3511317	0.9150853	1.284401	2.268463	0.7957152	1.313614	2.003497	0.3248011	0.2648746	0.5230495	1.56786	0.9081099	1.267742
2.192483	1.598431	1.691711	1.115095	2.094903	0.7132413	2.07888	1.458189	3.201476	1.810924	2.171992	1.527095	0.7132053	0.5157405	1.528389	2.305609	1.548994	1.410043
4.885686	2.264357	0.6594359	2.076894	2.176733	0.830366	9.044471	0.7986232	6.21859	1.656874	0.2884501	1.687412	4.559369	3.184113	0.318127	0.8916347	1.656094	0.4160287
0.01106416	0.01371276	0.04738941	0.02855606	0.06901227	0.03496468	0.03566897	0.01076098	0.09310208	0.2439684	0.1920486	0.05197003	0.02996822	0.005363008	0.02017212	0.4805703	0.005014585	0.1003133
80.41084	52.4195	150.1617	121.607	376.4044	39.97271	173.202	51.54723	348.1338	189.6781	296.2218	96.93891	20.88848	19.11295	66.72804	643.9898	82.38019	182.2958
0.2620889	0.4622569	0.02158778	0.2358862	0.2864339	0.08494836	0.5416216	0.0980412	0.9104387	0.04473455	0.1499757	0.6365794	0.6552829	0.03908905	0.03063071	0.1139091	0.06700746	0.1433624
1.764475	1.313341	1.55161	1.322956	1.183589	0.3862738	1.811379	0.7382214	4.309123	1.25494	2.240621	2.365245	0.5028426	0.4634727	0.8806262	3.216383	2.113198	0.5677765
1.865012	0.5652131	1.778376	0.646485	2.49075	0.3097451	1.243457	1.284299	1.603725	0.5776972	1.158968	0.991122	0.172072	0.3167321	0.781816	2.797804	1.542473	0.784108
5.158971	1.986795	4.132825	1.682736	7.297395	1.27316	3.693166	5.421406	6.456719	2.654959	3.57785	3.804003	0.7844045	0.8646063	2.361965	7.941448	4.132774	3.879027
65.48294	28.19104	46.73736	37.04547	92.00476	13.55599	49.59567	37.95626	75.62184	33.02218	36.71722	38.78657	10.25796	8.211114	30.17913	110.9562	40.76175	44.66241
0.02426705	0.01503812	0.05196965	0	0	0.03067524	0.07823282	0	0.0204201	0.1211539	0	0.03799533	0	0	0.04424355	0.05141638	0.065991	0
0.5726519	0.2180961	0.4854403	0.7266766	0.7813517	0.06786299	0.6730689	0.3422984	0.6726128	0.2233578	0.5719403	0.396937	0.2746697	0.08674224	0.2664518	1.396582	0.6272258	0.3308598
0.1465353	0.0886951	0.1824512	0.2884878	0.1452494	0.1809232	0.2123991	0.1226335	0.4731498	0.810978	0.2675906	0.1627372	0.6061236	0.112324	0.04349156	1.270782	0.1853411	0.1926509
18.05577	7.296465	13.63069	4.749655	11.96394	4.317545	8.740266	12.08278	18.38231	6.703989	10.49569	13.53542	2.855978	2.184171	5.356453	18.90846	7.213298	9.617561
1.624478	2.339404	2.260605	3.354158	1.805001	0.9477477	2.109658	1.53199	1.718381	1.680058	2.693604	1.202228	0.9471062	1.048824	1.058192	4.236186	2.095505	1.543337
0.07520488	0.06058509	0.05636985	0.2173921	0.06254499	0.0950642	0.1131422	0.02194324	0.1582074	0.3316584	0.1243224	0.09419963	0.169749	0.007290644	0.02742263	0.7011053	0.03408494	0.07219542
30.37706	15.04097	19.77332	26.42878	44.22881	9.04455	20.97194	23.23153	34.9533	18.21753	18.23812	24.36791	4.874063	3.032091	17.63438	47.25032	13.26284	21.45637
10.10391	5.849135	15.25079	10.25668	12.0404	3.042871	19.072	9.673018	5.961301	5.095359	4.921869	8.339731	5.910041	6.4789	5.726611	16.39707	7.771059	11.18274
0.3667698	0.30242	0.8179213	0.3098011	1.625977	0.16859	0.5928302	0.3537715	0.7243815	0.4237274	1.305326	0.3867933	1.652975	1.795433	0.1547388	1.358323	0.5907355	0.7597963
17.25114	7.651846	4.943827	4.951254	6.994958	2.624036	25.03816	2.15822	23.28043	8.60672	0.976243	8.994126	17.96663	11.1377	1.663964	4.512033	3.568699	2.061511
2.588598	3.465725	3.654716	8.835474	16.56156	1.466416	10.75947	2.890653	2.506319	8.105199	6.857369	3.059773	3.528801	3.3119	9.887681	15.95966	5.625683	4.363256
1.515689	4.924216	1.509609	1.369171	1.604639	0.718476	2.131777	1.452477	0.7221089	1.557936	1.423223	2.9228	1.393612	0.5672184	1.513772	4.651809	1.474927	0.9688352
0.5463296	0.482443	0.7020027	2.058673	1.221096	1.346667	1.658532	0.3719514	0.9194446	2.76166	2.890067	0.5667008	1.899051	0.1986122	0.7470489	11.53208	0.5571264	1.252894
0.04188366	0.03244375	0.02803028	0.01351245	0.02721329	0.02316294	0.01125215	0.01018399	0.06608253	0.0217818	0.03678297	0.0573807	0.04017858	0.1065844	0.04056734	0.07210286	0.007118562	0.03350634
150.0349	64.73489	126.0529	81.68656	223.9164	31.50154	71.14752	94.28842	190.6319	72.02978	88.30089	110.4564	21.17315	19.37527	64.28918	213.549	96.87839	113.5867
0.2811991	0.2788112	0.7587823	0.3657835	1.08746	0.135073	0.2538306	0.2133251	0.7903167	0.4586053	1.08776	0.5855698	0.04062141	0.06542519	0.2255795	1.459703	0.2548944	0.3539297
3.653467	3.125057	3.519364	3.487793	3.416787	1.5513	1.803861	1.797257	4.759822	2.640931	1.719771	2.048484	1.012497	0.54016	2.456076	11.03597	2.608447	1.670127
0.1948866	0.2415396	0.02158778	0.1248809	0.09780668	0.04247418	0.5705081	0.01307216	0.2035763	0.2907746	0.05554657	0.1578296	0.7644967	0.01302968	0.06126141	0.04271592	0.06091587	0.02150435
0.5212457	0.4998998	0.4518299	0.2434369	0.4128571	0.3765091	0.8802132	0.5431742	0.4177284	0.5266626	0.3282589	0.3206198	0.5714582	0.3248455	0.4864797	0.3812815	0.33749	0.3044684
0.09933266	0.03077786	0.2233645	0.3486668	0.2065278	0.08789438	1.835994	0.08694967	0.1671717	0.570309	0.3612584	0.2566192	0.05381017	0.1348155	0.06338597	2.430852	0.09004063	0.09535769
0.03077684	0.01907218	0	0.01059113	0.04265985	0.01296802	0.02204873	0.03991133	0.0431632	0.03414537	0.01695923	0.05621911	0.04631203	0	0.01870406	0.02173638	0.01859856	0
0.02161493	0.02567301	0.01735872	0.003719137	0.005617593	0.02959967	0.0116138	0.003503772	0.03789251	0.09742153	0.02679898	0.02961249	0.0308992	0.003492387	0.01313607	0.1011353	0.008163736	0.01344905
0	0.004029419	0.006962558	0.03356416	0.02027887	0.008219342	0.006987419	0.01897233	0.03830054	0.102799	0.07524321	0.03563258	0.01761199	0.006303561	0	0.01377687	0.04715226	0.1040348
0.4981351	0.2234333	0.3606775	0.122444	0.3846883	0.05996929	0.1172564	0.03691316	0.6107875	0.3434363	0.2980195	0.2228396	0.154199	0.03219406	0.06487124	0.2462683	0.1591131	0.1315686
0.07527798	0.01036649	0.2866013	0.1208908	0.139124	0.0740107	0.02696482	0.0325401	0.1548421	0.5776605	0.04839458	0.281564	0.01510346	0.00810859	0.1677456	0.1240532	0.01516359	0.1784336
0	0.01550265	0	0.03874007	0.02600674	0	0.05376631	0	0.04210176	0.01040803	0.02067772	0.0734419	0.06775975	0	0	0.01325116	0.005669124	0.006670995
0.01839171	0.05698604	0.07877435	0.1735976	0.09559795	0.0232484	0.09034928	0.03066469	0.09727864	0.1136832	0.6102441	0.03496682	0.05218765	0.06367719	0.08861939	0.205973	0.0523954	0.07006242
0.6267001	0.5085686	0.8348329	0.3697101	1.268104	0.3206494	0.348755	0.4317485	0.586993	0.8753237	0.7585056	0.5957497	0.4041595	0.6147793	0.4896841	1.557045	0.4057683	0.4337087
0.3666347	0.6445085	0.6810189	1.251388	0.9489729	0.4350772	0.9568308	0.2037647	0.9255409	0.9650244	0.9709802	0.3045959	0.432353	0.1595806	0.4228946	2.829829	0.3933797	0.7901214
3.606316	2.066143	4.298755	2.845002	11.24712	1.763245	5.37435	2.878793	8.788951	4.472847	3.205797	3.009674	1.443699	1.088408	3.070408	9.551207	2.066232	14.84234
3.268092	1.756341	2.203066	3.149949	2.044279	1.910742	3.301358	3.328278	1.360286	2.131216	1.613819	5.423701	1.377384	0.2713671	1.282261	8.592416	1.573169	3.0455
0.09737627	0.1949555	0.05614494	0.2010587	0.09344317	0.07574793	0.3139244	0.07285228	0.1386671	0.2056804	0.2971832	0.07623183	0.09468014	0.05083089	0.1365661	0.2777361	0.1629549	0.1118559
5.532142	3.38974	4.968912	5.312813	8.910262	1.671145	5.935358	3.192949	14.18945	4.189786	8.485227	4.386452	0.8571596	1.062291	3.963291	15.8431	4.318944	5.389789
0.6783782	2.786348	0.9557869	5.393879	2.655114	0.4242454	1.62936	1.3844	1.876754	0.9625701	0.688602	0.9559332	0.4491531	0.2515205	0.3927431	4.334684	1.818867	1.061266
114.8654	49.87883	105.4826	78.58153	223.6834	24.35997	59.47911	77.81985	171.6585	67.01734	81.93554	97.44638	20.25237	18.54455	54.47792	198.0196	69.83838	106.4646
0.2453056	0.05784608	0.1603913	0.1165392	0.0812434	0.109764	0.02799375	0.1393498	0.2100718	0.07767238	0.152518	0.244723	0.03527952	0.016836	0.1088416	0.1908806	0.09248544	0.09493668
0.3997018	0.5917094	0.2139976	0.2521715	0.3462672	0.5333204	1.073809	0.1727774	0.3363388	0.8314674	0.8626501	0.2259905	0.7217506	0.08610799	0.2833966	2.023096	0.4025687	0.4026555

TCGA-DU-6404-01	TCGA-E1-5319-01	TCGA-TM-A84M-01	TCGA-TQ-A7RK-01	TCGA-TM-A84T-01	TCGA-CS-6666-01	TCGA-DU-7019-01	TCGA-TQ-A7RR-01	TCGA-E1-A7YV-01	TCGA-HT-7477-01	TCGA-HT-8011-01	TCGA-HW-8322-01	TCGA-HT-7879-01	TCGA-HT-7676-01	TCGA-FG-A87N-01	TCGA-TQ-A7RW-01	TCGA-FG-A6IZ-01	TCGA-S9-A6WH-01
0.1668134	0.5956649	0.04094437	0.0296631	0.0183635	0.1134831	0.1617558	0.0897879	0.07702029	0.01672917	0.01844389	0.1183072	0.05434314	0.04487957	0.04770002	0.1281697	0.05163655	0.0969104
0.1279065	0.2128709	0	0.07915122	0	0.6599738	0.8056897	0	1.383283	1.376373	0.03075911	0.5170683	0.1208383	1.021431	0.09091418	0	0	0.6225312
1.07017	0.5166873	0.1671326	0.36446	0.6005028	1.089188	0.7970288	0.5986314	0.7420465	0.7090519	1.537108	0.4759845	0.785016	1.174249	0.9627264	1.237253	0.3114822	0.3794663
0.5142498	0.05943405	0.0793722	0.02841326	0.009772091	0.04645366	0.1962578	0.08958827	0.7472091	0.08902387	0.06256981	0.0683842	0.1084446	0.1770119	0.4931641	0.04977135	0.02289855	0.06876084
12.16045	10.74325	2.331638	8.768704	7.703986	19.27568	13.80403	9.840214	14.96635	11.589	12.79407	8.377751	11.53105	13.06583	11.19095	15.76349	5.948955	10.98
1.217093	2.069339	0.7415099	1.496991	2.392784	2.841888	2.79808	2.22421	2.100855	2.788899	2.678555	2.125461	2.412846	6.048075	3.115929	4.460586	1.157837	1.270572
2.920921	0.9385727	1.385198	2.641043	2.467117	6.606083	2.760051	2.498198	1.113007	3.263386	4.425602	2.113376	1.763363	1.929688	2.529241	1.885227	2.978734	0.948084
3.260959	1.242302	1.247718	2.989131	2.499208	1.86493	2.094231	3.623696	1.075097	4.155261	2.603612	0.8702285	2.307944	2.634803	1.762719	5.028741	1.113111	1.122482
0.06023234	0.2192813	0.02296806	0.02329564	0.01201801	0.2056683	0.1609091	0.02203566	0.3605967	0.2518136	0.06337075	0.3364036	0.02667368	0.103664	0.05351539	0.05440913	0.03379358	0.1691282
2.466011	1.511944	0.4106989	1.821165	3.385948	2.332148	2.63349	3.673575	0.6797984	1.396666	1.743158	1.557979	3.833131	5.621977	2.213375	7.756554	1.358387	1.041885
0.0671032	0.03428705	0.0484827	0.005463797	0.03946211	0.1125548	0.06356188	0.02067311	0.005456446	0.02054287	0.01698637	0.05260042	0.004170725	0.04376434	0.01255158	0.0191418	0.010568	0.02644507
38.89413	19.35766	1.152816	26.14707	50.57306	38.52928	52.86642	86.32284	10.82136	53.20784	29.00619	13.48584	53.35129	134.5931	80.40435	133.7025	26.27221	12.46529
0.443552	0.3168066	0.1657545	0.5935613	1.040769	0.498117	0.8157356	1.106688	0.2535517	0.3257223	0.4239969	0.500175	1.259742	1.755782	1.053529	1.983315	0.3384448	0.3570328
0.3066641	0.140704	0.200466	0.6099754	0.2208285	0.2274467	0.127085	0.4082751	0.3526685	0.3285859	0.1441669	2.349894	0.5990401	1.060342	0.3960654	1.191377	0.200084	0.2632911
1.725605	0.4170768	0.1893045	0.8750972	1.62676	1.064892	1.393341	2.556645	0.2581198	1.332741	0.9470393	1.124808	2.426767	2.359476	1.320406	4.092053	0.507066	0.3976382
1.058136	0.05644305	4.03493	0.05036896	0.04547354	1.803458	0.5584895	0.05955584	0.2389307	0.3018213	0.01468048	0.09524942	0.0624789	0.08965536	0.04339086	0.3602767	0.08524506	0.09142067
0.1558955	0.08107879	0.1783403	0.03768413	0.0544345	0.1035063	0.2082352	0.06416266	0.02258006	0.07792698	0.09958261	0.1088365	0.08054406	0.04024603	0.05771268	0.1232207	0.05831049	0.01823932
3.126632	1.890294	0.6352311	1.029889	2.457625	1.206587	2.746826	6.69926	2.208114	1.968213	1.604703	1.741976	3.97548	8.93564	5.774566	11.14351	1.51524	0.8209431
250.0024	91.8662	80.46671	52.23551	97.46784	127.6441	231.1158	372.7908	60.65903	200.8441	268.4227	152.5159	137.2788	198.7624	211.5463	570.0491	137.6084	53.84907
0.5274659	0.2072691	0.05363617	0.1768037	0.5472684	0.707089	0.7350449	0.6989816	0.1856205	0.8692879	0.3699664	0.2743318	0.6644236	1.178129	1.117803	2.027649	0.2455178	0.2358769
12.7777	3.174531	1.114704	6.868943	13.85902	11.0419	29.03501	15.07322	5.453675	18.15303	12.46251	6.107196	8.472911	65.76505	49.95215	13.96709	4.799946	5.403343
0.04932177	0.1231271	0.03510751	0.06104268	0.09447406	0.0898204	0.1183544	0.05774108	0.04572041	0.03825155	0.06325843	0.08395184	0.07377733	0.07696345	0.09738119	0.2554394	0.03935596	0.06770725
2.114618	1.600832	0.5468708	1.066892	2.169479	2.626154	2.432084	4.960274	0.9184967	2.354122	1.933365	1.833144	2.376231	3.757777	1.185357	3.936024	0.5829439	0.9314038
7.760409	6.04931	1.481122	8.262355	12.77166	6.289925	6.202755	15.50964	4.305524	8.518176	8.728385	5.04751	12.56267	22.17971	8.468515	16.82291	12.50245	5.460603
4.790996	2.319838	1.241382	1.57212	2.329055	2.992244	2.857642	3.20448	4.258464	2.478125	2.370789	2.540028	3.026698	4.203686	3.56624	3.89929	1.224382	2.045378
0.4410552	0.6836599	0.2967973	0.2207556	0.5590755	0.5512228	0.4377462	0.673905	0.1402918	0.3489775	0.920274	0.6210094	0.4902143	0.5983387	1.129507	0.6327758	0.3202367	0.2914006
9.465083	6.454733	5.368037	6.132252	8.639306	10.03902	15.16407	18.83275	3.748729	10.6916	12.43093	8.678447	12.08533	12.83045	12.65616	27.21831	13.76993	5.648287
12.49461	6.368323	2.672736	6.134808	9.699674	11.8117	10.99886	10.97781	5.153481	8.601049	7.704299	4.933545	11.10275	14.57264	9.512079	14.90053	8.412441	4.749003
0.3645593	0.2670044	0.01874364	0.04435894	0	0.5408197	0.23959	0.08391942	0.9555982	0.08934722	0.1526831	0.1917355	0.05804721	0.2368731	0.1989526	0.03700157	0.02451384	0.3373851
0.4021717	0.3542033	0.2731337	0.313361	0.7965856	0.3964934	0.5976633	0.5455705	0.1859495	0.4012641	0.3953302	0.7057826	0.391734	0.3961003	0.4555645	1.230413	0.3645374	0.285752
0.01018011	0.04235614	0.05693492	0.0472475	0.08124849	0.05149754	0.0954242	0.05462361	0.05766925	0.05427948	0.03264168	0.06136941	0.09216822	0.01401654	0.008039871	0.06130606	0.00507697	0.02540894
0.484423	0.6208161	0.3460621	0.7684005	0.3523651	0.6979335	0.6207651	0.9242537	0.2273679	0.5617587	1.201811	1.324236	0.680687	1.409946	0.4721704	1.285073	0.9632968	0.2525314
4.343886	2.623338	0.5791527	4.677746	7.478713	5.24948	6.763007	8.441673	1.73834	5.511279	5.910034	2.697832	8.531839	18.05302	5.905263	13.54965	3.674297	2.451855
0.4787912	0.4490117	0.3894929	0.444429	0.9607788	0.7681303	0.5898503	0.5872132	0.1972582	0.4641587	0.208349	0.2376974	0.8562013	1.255669	0.7022437	2.001867	0.143268	0.2987583
1.905931	2.476153	2.183207	3.869275	4.858035	2.938156	3.618275	4.034818	1.943673	2.431931	2.509097	4.343677	4.554896	7.831082	3.034263	7.281358	3.212215	2.157611
1.929653	1.392328	0.5581289	1.441834	2.141627	1.765599	1.751904	1.560647	1.444726	1.559911	1.334976	1.626951	2.411732	3.246785	2.323	2.638657	1.810832	1.182607
1.228756	1.47734	0.1011269	3.651464	5.016295	2.528816	5.180547	6.616872	1.072113	2.635216	1.58907	1.641035	3.998265	15.14117	1.96354	6.492073	2.182267	2.7056
2.727273	4.14745	0.6050769	0.8261438	1.19336	2.355983	1.956476	1.618739	3.429777	2.318518	3.302221	1.700232	1.999989	1.790875	3.976427	1.922644	1.055766	1.428064
0.4424352	0.3549807	0.189626	0.7466948	0.9980517	0.754672	0.5963749	0.7598168	0.6327068	0.6274193	0.316554	0.4706751	0.9413317	1.3442	1.017934	1.327799	0.4431211	0.3901523
5.071028	7.584996	2.538438	4.305617	7.984413	7.595869	10.10018	8.406906	2.116017	5.309517	6.549653	8.165407	5.977395	14.72794	7.551898	17.00942	3.554347	2.798536
5.144047	1.827386	0.9574916	1.690394	3.525852	3.947176	3.672695	4.009956	1.913202	4.423975	3.225285	2.4228	3.289107	7.955764	3.129418	6.750427	1.695671	1.524023
1.003453	0.8452534	0.3521236	1.819062	3.06589	1.70034	2.091237	3.166583	0.6657751	1.763547	1.510047	1.290171	3.151522	5.666291	1.783101	4.131808	1.777623	0.9795425
1.403919	1.216022	0.3423047	0.6297806	1.691136	1.10885	1.197587	2.001001	0.3950991	0.7968758	1.04799	1.149295	1.922942	2.148568	2.578182	1.357764	0.7105658	0.5568775
1.634135	1.119956	0.4655331	0.580379	1.938567	1.032508	0.7795937	2.093599	0.3536531	0.8506564	0.7186768	1.122883	1.681995	2.784005	1.491446	2.550232	4.785153	0.987934
0.1167895	0.04360848	0.06166341	0.06949209	0.07887071	0.1772388	0.06063156	0.131467	0.145737	0.09797907	0.1350273	0.1481371	0.07426435	0.0804011	0.06385566	0.0811526	0.07392584	0.1345382
0.0937895	0.09291132	0.2145854	0.04145635	0.203176	0.1321675	0.1657815	0.1078388	0.1138516	0.1461265	0.08860738	0.05701482	0.1265809	0.2490452	0.09523467	0.1210314	0.05011518	0.06270342
0.01795544	0.02490225	0	0	0.02866083	0.02724904	0.02019683	0.05255117	0.08322189	0.0522201	0.02158973	0.01910151	0	0.04944408	0	0.03243905	0	0.03361175
0.05546185	0.3905147	0.006507372	0.3036084	0.1974884	0.1424389	0.2543396	0.1685664	0.3031999	0.1302812	0.2770102	0.1316196	0.2821376	0.2290888	0.06570252	2.790176	0.07659588	0.207644
14.11532	20.13184	10.1381	20.44582	45.05108	32.17993	39.46021	35.52345	8.434358	37.94585	17.24268	18.86565	27.08213	92.92692	39.58588	26.36448	29.40197	14.77017
5.784672	6.123546	4.622131	13.017	8.701698	7.45129	4.855191	5.455492	5.293108	7.639544	7.788182	5.998805	8.362296	9.534645	10.89289	6.697561	9.464858	5.565393
8.716454	7.230012	1.019962	7.871919	15.00877	9.041565	9.1028	13.04075	2.777067	10.77284	7.231717	4.739068	13.2588	27.06264	12.74858	21.94648	6.19239	5.357428
1.203324	1.608315	0.8437042	2.582225	5.3828	3.622855	4.393531	4.736013	0.9670316	4.565061	1.633578	2.838998	4.824639	11.31035	3.558026	7.039119	0.5880157	1.208029
41.75352	110.4983	24.07313	59.67934	79.25233	72.40789	88.50584	99.76194	29.34082	72.28986	88.39679	51.65544	65.82975	234.5871	69.14705	122.7867	107.2638	49.33793
2.235828	1.565087	0.9876176	0.7727426	0.7846459	1.564184	1.337728	2.343673	1.20451	1.767818	1.639717	1.71221	1.407559	1.535567	1.978668	1.241405	0.6959114	0.8509257
1.002234	0.6119199	0.1426198	0.510809	1.142704	1.121891	1.53161	1.706099	0.3972629	1.980034	1.071584	0.8765887	1.366445	2.039724	1.208059	3.441872	0.2754158	0.3172475
0.87927	0.9899081	1.262257	1.888384	1.386996	0.8791165	1.047208	2.588545	0.9109581	1.835163	1.330878	2.233937	1.905679	2.02246	0.9680953	2.990166	1.578616	1.25867
1.567478	1.004754	0.1004567	0.1630232	0.3153831	3.498224	3.555927	0.3855145	14.9169	8.351271	0.3009256	3.307029	0.544433	7.145603	1.778881	0.5711336	0.5715123	5.917807
0.1064917	0.09353852	0	0.005491607	0.08499206	0.1292888	0.1317636	0.01558375	0.02193687	0.01032372	0.02134104	0.07552592	0	0.02443727	0.02523094	0.2950016	0.06904162	0.04651442
236.8326	115.3641	85.93214	24.80841	49.22726	107.1946	223.2528	245.8009	34.33956	146.8224	218.9324	166.1123	86.93509	121.5422	168.5679	437.3542	92.1579	36.54932
0.2889114	0.05980429	0.01315451	0.01334212	0.03441538	0.2748491	0.3831811	0.233479	1.812087	0.4452048	0.1140679	0.2752407	0.04583047	0.8371383	0.2094409	0.1324375	0.07741842	0.3148108
1.119545	0.3772066	0.7476955	0.4745862	1.357949	0.5087441	1.33756	3.683899	0.752453	0.8737831	0.8061676	1.147267	1.774001	1.180038	1.517334	6.056424	0.2649718	0.3433693
0.7512168	0.6178449	0.3330899	0.5675722	0.9899605	1.087016	0.9923739	1.533926	0.2699088	1.168601	0.9347768	1.003604	1.361641	2.850384	0.8071405	2.067332	0.7841358	0.3597366
2.738038	1.724207	2.310146	3.190272	3.709027	3.34664	3.301798	5.963246	1.08994	3.579799	3.209185	3.253941	5.220071	10.24321	3.88063	8.636589	2.826952	1.422205
33.42729	40.72712	8.534903	33.56422	53.28734	43.81141	40.82102	54.71063	16.43224	30.95397	40.0702	22.11677	58.62603	113.7337	30.77263	79.94963	52.19785	20.56639
0.03114246	0.107978	0.1187538	0.192716	0	0	0	0	0.1683996	0	0.01872294	0.1159558	0.01838844	0.04287865	0	0.08439489	0.06989036	0
0.4286901	0.270731	0.05838214	0.2368592	0.2260579	0.1916878	0.4047066	0.8345799	0.2956757	0.4230099	0.3865945	0.3298238	0.5152905	0.7799653	0.2448538	0.5670368	0.4352233	0.3654186
0.4438905	0.4791587	0.05336474	0.08795451	0.08376907	0.4280797	0.2656383	0.06079806	0.4020194	0.1271895	0.1393499	0.3512597	0.05680996	0.1655887	0.03367525	0.1422179	0.03271546	0.1514526
8.165209	5.934809	3.748661	6.991438	11.88427	10.90396	13.36367	13.17158	4.190112	8.724334	7.648501	6.333307	13.40525	19.37148	9.248132	22.95275	5.697201	6.170247
2.160691	1.998738	0.4151807	1.260042	1.714365	1.99817	2.031672	2.21704	1.248567	2.00363	2.42804	0.9382978	2.337317	2.472354	1.917237	2.325384	1.145968	0.8176327
0.1640706	0.2275481	0	0.01493093	0.06162182	0.08787963	0.1139878	0.07767851	0.1267421	0.05613756	0.1392561	0.1283404	0.03419205	0.05979745	0.04001637	0.2005173	0.02165939	0.05419987
20.28905	21.82847	3.836906	21.32953	27.05398	23.53096	26.81091	24.21911	7.220621	16.4308	23.2387	13.29263	21.96318	54.11003	19.86657	30.09886	14.62238	10.98749
9.714775	5.693754	3.079318	6.026378	6.077326	2.38521	9.502484	11.2125	10.34096	7.516571	4.015888	3.344053	10.91239	8.562774	6.725356	13.67127	6.44635	3.931338
0.3325908	0.0701342	0.1068	0.3911671	0.2638916	0.4663653	0.4222391	1.391808	0.5919711	0.7947526	0.3063631	0.4531373	0.3766863	1.65765	1.663562	1.029565	0.2415268	0.2767087
8.451318	9.141153	0.4553807	0.5684622	0.6598448	14.1849	11.46956	1.680362	31.82653	24.01138	0.7731891	11.50719	2.006921	21.31203	4.108102	1.410677	2.130303	21.19428
8.011778	12.65291	2.890095	2.997935	2.189799	4.795603	8.712034	9.04751	5.937113	3.509729	2.290616	8.291917	4.717331	3.27233	8.274044	4.241925	7.507453	3.846346
13.60152	1.452972	1.036228	1.548852	1.19285	4.297613	8.401805	3.453403	1.46943	2.703722	2.300127	1.570973	1.786115	2.239993	3.744441	1.104626	0.5724057	1.539465
2.278631	1.470715	0.1069413	0.2847245	0.6994607	2.513724	2.050459	0.2564997	1.96331	1.197954	1.433147	1.062863	0.3829343	1.448005	0.6021661	1.504168	1.088308	0.2132746
0.01175787	0.0256251	0.0358685	0.03897864	0.03485511	0.1554946	0.04345557	0.03195436	0.02076064	0.04885085	0.06462961	0.02680361	0.05355699	0.03700315	0.0198984	0.1122805	0.01005226	0.009432935
71.02995	108.2986	30.00748	72.26501	105.505	83.78326	94.41114	159.7356	33.44622	116.8133	148.3878	75.42641	112.0367	345.2584	93.08177	170.5031	81.35165	45.1887
0.5340864	0.2051994	0.03302598	0.2065649	0.5702662	0.5366992	0.462745	0.5492112	0.1393831	0.3515897	0.3427898	0.3839029	0.3963279	0.9738545	0.5087266	1.147452	0.1079824	0.1148402
2.611303	3.006487	3.106337	1.596324	1.661506	2.78158	2.331492	2.470282	1.985729	1.888752	2.356244	3.967164	3.281495	4.031567	2.12307	5.110147	2.457883	1.719778
0.06468166	0.167452	0.2170493	0.01334212	0.006883076	0.03272013	0.3492283	0.03786146	1.445672	0.05643441	0.2177661	0.2890028	0.05092275	0.1128059	0.06129977	0.08569482	0.1419338	0.4681801
0.2866828	0.5743081	0.5587423	0.1724774	0.08897947	0.3746415	0.2866396	0.2330695	0.6889812	0.3242416	0.191505	0.3642832	0.1974878	0.4714719	0.1132056	0.3309017	1.262927	1.028592
0.01912138	0.7602193	0.009721932	0.01972118	0.4680028	0.1547651	0.3871496	0.09327263	0.3446563	0.018537	0.09963051	0.1017093	0.1957011	0.1404126	0.1510135	0.02303032	0.04768053	1.479499
0.01974831	0.009129587	0	0	0.04203019	0.1698293	0.0370225	0.0385323	0.1525528	0.01914478	0.007915161	0.007002938	0.0155475	0.01812704	0.03119294	0	0.009848766	0.0616132
0.01386946	0.07373585	0.0246809	0.005364196	0.003689785	0.005262047	0.01950099	0.02198761	0.06071242	0.03025259	0.006948634	0.1094309	0.009554289	0.00954812	0.01506117	0.01461667	0.1020243	0.0865433
0.04172266	0.06365127	0.362745	0.01290943	0.0133197	0.03799079	0.02815855	0.01831679	0.05156822	0.0182014	0.03511729	0.3195774	0.02956277	0	0.01482794	0.03768894	0.04993845	0.007810288
0.2252659	0.2575349	0.09750743	0.04709434	0.1943643	0.1894099	0.3389894	0.2806471	0.09876505	0.2434659	0.1866745	0.3367974	0.2839964	0.5071511	0.3678338	0.8304486	0.1229704	0.09687412
0.09123895	0.133982	0.7858818	0.01660605	0.1370705	0.07330422	0.4708831	0.08639335	0.1907126	0.03121785	0.07098639	1.193299	0.06338016	0.0960645	0.1144436	0.1648361	0.3774004	0.07032739
0.01203917	0.01669702	0	0.006208409	0.006405721	0.02436074	0.02708406	0.01761785	0.09300084	0.01750686	0.009650647	0.02561523	0.009478232	0.02210159	0.07131064	0.02175047	0	0.02253677
0.1129548	0.04910115	0.02314339	0.0286898	0.03229266	0.05628694	0.06826837	0.02467097	0.01823258	0.05393419	0.09324784	0.06815302	0.0358364	0.04178213	0.065907	0.2741223	0.2043093	0.06311826
0.7515987	1.463988	0.2300126	0.5850843	1.062169	1.075234	0.9315787	0.8021362	0.536222	2.00837	1.479349	0.7333655	0.7575531	3.720819	1.412126	0.5102822	0.8021905	0.3629414
0.7393739	0.8323305	0.1391399	0.2599659	0.3601918	0.9180556	0.6318534	0.3302155	0.6304963	0.3281352	0.8659358	0.6486623	0.5442978	0.403238	0.3241947	0.7893267	0.6464858	0.5212745
1.72461	5.722266	3.063418	3.512384	4.739094	5.0026	11.00092	3.322411	2.158559	4.69826	7.034765	2.577854	4.150607	9.918955	5.482609	14.59276	4.997218	2.574539
5.06066	1.961717	1.739689	1.333796	2.171793	2.609979	4.712507	3.311845	2.122877	2.024215	1.031259	0.8885212	2.34914	1.323077	3.989626	3.342354	1.444424	1.109564
0.1153525	0.2066426	0.04398672	0.1040995	0.1380959	0.08752901	0.04325066	0.06330157	0.2376216	0.104838	0.1213629	0.1073758	0.1759541	0.09926477	0.05124434	0.1476171	0.0934829	0.0899727
4.301275	1.823968	0.9161569	2.08745	4.257601	3.564034	6.576886	8.177289	1.517302	4.185001	5.579193	3.721827	6.124957	8.234686	6.761372	11.23715	4.446378	2.701692
2.845416	0.8282322	0.1584151	0.318986	0.8971672	0.4566192	0.8452499	3.424112	3.013312	1.103827	0.4811553	0.6304291	2.066995	1.970429	8.793401	5.695274	1.903492	0.689041
58.6575	92.72726	18.49496	62.17915	97.02562	67.94119	112.9485	141.1537	31.27792	101.3529	133.009	61.29208	83.75547	315.8037	86.45092	131.4981	90.3605	39.43156
0.05571793	0.03477362	0.08498643	0.1292978	0.2290155	0.06130389	0.1049777	0.2283018	0.08177843	0.05671587	0.05192156	0.1348491	0.1398221	0.1821993	0.1353121	0.4932454	0.02709265	0.1069089
0.7551711	0.3161782	0.4781309	0.110216	0.3411563	0.5838326	1.041765	0.3544661	1.05665	0.4558309	0.3940473	0.6214768	0.1346113	0.4119808	0.1181558	0.2574195	0.2558138	0.480106

TCGA-DU-6392-01	TCGA-28-5220-01	TCGA-FG-A6J1-01	TCGA-14-1034-02	TCGA-HT-7880-01	TCGA-HW-7486-01	TCGA-FG-A60J-01	TCGA-DB-5280-01	TCGA-FG-A60L-01	TCGA-DU-A6S2-01	TCGA-FG-A4MW-01	TCGA-32-1970-01	TCGA-HT-7873-01	TCGA-DU-8168-01	TCGA-HT-7475-01	TCGA-14-1825-01	TCGA-FG-6691-01	TCGA-TM-A84Q-01
0.09769894	0.4514652	0.05422153	0.9931794	0.02672086	0.09777384	0.2982827	0.08403653	0.07278176	0.01960633	0.05884656	0.1695128	0.02014922	0.04780298	0.06098613	0.4368345	0.03607255	0.2191909
0.9200964	0.2151183	0.9787275	0.1798738	0.4838238	0.05929405	0	0	0.211094	0.7473765	0.06039332	0.403855	1.075299	0.04555521	3.80676	0.4162939	0.09625382	0.06357345
1.165908	3.968281	0.4982075	0.9587979	0.3163118	0.5714489	0.512889	0.5813494	0.8197987	0.5308784	1.793555	4.327516	0.6247797	0.3642408	0.3277734	1.733602	0.9345217	1.11873
0.3149998	0.3912579	0.142572	3.250027	0.02437619	0.04257009	0.07585346	0.04236616	0.2071247	0.5142206	0.1228514	0.25612	0.1322424	0.03815736	0.1703815	1.228719	0.03839184	0.4107823
20.24056	34.36099	12.9713	7.326757	36.33175	8.83808	6.715415	7.012821	15.66145	24.19945	11.47543	11.15807	6.29724	4.649171	18.32076	17.93508	11.48201	13.08278
7.984534	4.642238	1.414697	10.00193	0.9516702	2.203869	2.343238	1.700853	3.410322	0.6050668	2.478923	2.106439	2.800225	2.797579	0.6431757	3.744737	8.035608	4.128995
2.695817	5.07407	3.544254	2.99837	1.360345	2.013888	2.470436	1.928704	6.297793	2.276925	4.555984	2.47452	1.458021	1.115735	0.9869799	3.410455	2.929689	2.023605
2.762474	7.078065	2.155007	3.661051	1.831204	1.548585	1.401329	1.566782	2.895549	1.346342	3.635205	7.415388	2.026965	0.7613192	1.820618	4.919167	4.563489	2.492825
0.09026703	0.2026023	0.6011631	0.2647006	0.1423981	0.09598224	0.1243824	0.05210315	0.07455456	0.2309648	0.1066491	0.2139514	0.2637334	0.2614507	0.3250011	0.2858867	0.03777233	0.05613246
4.233711	4.948206	1.982773	1.473831	1.653928	1.824745	2.436587	2.242073	4.346229	0.4628808	1.581435	3.670212	2.975232	0.7864892	0.8019643	2.627261	11.68186	4.706475
0.02469993	0.2732324	0.1292479	0.1903888	0.03164048	0.06548895	0.06806999	0.01222036	0	0.1341374	0.01667577	0.07248294	0.02061882	0.01257868	0.05215482	0.03831561	0.02214796	0.03510772
119.9076	125.0043	49.23334	31.43114	29.35091	47.75324	60.70842	45.26902	92.52715	10.16431	77.22516	87.90641	40.56478	3.123962	12.87106	50.51802	165.4359	131.2497
0.7910262	0.9175597	0.6935594	0.2962875	0.4282794	0.3392711	0.775508	0.8083038	1.405494	0.1360663	0.5654634	0.7492576	0.8623089	0.2744826	0.136041	0.9022604	1.930403	1.04718
0.3639791	0.329621	0.2646498	0.2810208	0.1629598	0.3981585	0.6824765	0.1489266	0.1788514	0.2694561	0.1687482	0.2875684	0.3069165	0.4845166	0.09953053	0.4877889	0.2255693	1.054344
1.82897	2.099372	0.7225795	0.6433165	0.7270016	1.20047	1.225603	1.351629	1.851331	0.8646522	0.8564712	1.623989	1.647004	0.4887832	0.3090789	1.061629	2.924634	1.607405
0.2846258	0.4928165	0.1286272	0.8775659	0.05671618	0.04716572	0.3641828	0.05632773	0.1477658	0.5112735	0.03843211	0.1349243	0.0807832	0.1087113	0.08783814	0.4856762	0.137818	0.5663816
0.0973468	0.1310956	0.04862348	0.1370215	0.02909683	0.05646014	0.2347413	0.07304658	0.1929647	0.1280981	0.03450412	0.04614638	0.02844184	0.1344716	0.03320445	0.0264265	0.05499207	0.1029095
5.692841	7.391609	2.0993	1.878282	0.9987141	1.791669	3.865727	1.157186	9.200742	0.723585	2.636772	4.537581	3.768625	0.471952	1.27231	2.875203	15.82445	7.919132
411.7655	594.4425	101.8919	128.4662	101.6676	110.1143	263.6032	101.5994	243.7153	27.73457	163.7778	371.2522	24.99194	105.1331	47.45567	261.8295	310.2333	473.6889
1.168162	2.478989	0.2144795	0.6318785	0.0845923	0.2614996	0.4195592	0.4562771	0.3820605	0.07277087	0.3389879	1.295337	0.2018729	0.1408968	0.1165068	1.223964	0.8563511	3.41909
43.53357	57.03365	6.521809	12.35649	2.43863	15.5649	7.510979	3.403422	9.861303	1.765836	7.118326	19.62083	11.85517	0.8962668	5.325611	25.95462	6.825486	82.80165
0.1116952	0.05530089	0.06016603	0.1156012	0.05564258	0.07240348	0.06790108	0.125151	0.08682615	0.09126154	0.03881357	0.0519099	0.05375013	0.06441032	0.04482189	0.07134499	0.2062013	0.1062292
3.636932	4.682452	1.010237	1.320531	0.9832834	1.753226	2.017179	1.419914	2.00157	3.265367	1.433766	3.254623	2.494715	0.9854051	1.097159	2.698976	6.832391	2.832143
15.0454	30.33905	18.43782	15.50597	5.088406	6.990092	9.710994	6.574175	33.64764	3.182929	9.505336	16.94389	9.697302	2.681705	2.425827	17.34372	19.2784	18.53536
4.144258	1.901932	2.554314	1.101317	2.065641	2.761891	2.024222	1.765885	4.066643	5.205451	3.768486	2.718779	2.590977	1.062973	1.389321	3.487903	4.142268	4.01162
0.8165123	0.6326984	0.3236805	0.6080901	0.2518023	0.5412215	0.2946701	0.5984764	0.9741114	0.104223	0.7043818	0.5119846	0.9466691	0.3753901	0.1252548	1.477711	1.301602	0.5641638
20.83052	26.99152	11.36927	7.856475	5.91337	7.466464	14.34799	7.195472	27.84155	1.382647	12.91349	21.0105	4.69544	5.17769	5.83336	13.47397	18.11522	26.23177
13.60477	4.964995	10.55893	4.189476	4.563809	7.246328	8.762917	8.347942	20.89956	5.525446	6.587362	8.537856	8.114155	3.850432	3.449407	5.783235	18.78226	12.79292
0.3069358	0.1653385	0.3815728	0.5568395	0.3669709	0.08544942	0.06767019	0.03307114	0.06084205	0.7359868	0.2997823	0.3621338	0.6791576	0.17142	1.005063	0.4666095	0.07706257	0.09161651
0.4458026	0.4986513	0.4834342	0.3921838	0.300981	0.4831007	0.4203028	0.6331484	0.5329258	0.4030932	0.1732595	0.426365	0.3016322	0.4286671	0.2767773	0.4458667	0.7879138	0.4523094
0.03729342	0.1084351	0.01693417	0.1033948	0.07093517	0.03932691	0.04671641	0.1213294	0.03360205	0.03469894	0.07210081	0.07500017	0.02773534	0.09668671	0.08094913	0.03681438	0.0340483	0.1054131
0.8576969	3.269201	0.7344148	0.2443264	0.2868841	0.6822246	0.8272976	0.1909565	0.7185205	0.1343618	0.7527785	0.8131671	0.8233787	0.9336395	0.1671746	0.8980852	0.3999218	0.4419252
10.32613	17.10136	4.994942	2.996414	3.249095	7.727025	6.344681	2.603514	13.11355	1.964201	4.745194	7.589162	8.712354	0.908793	1.954791	7.615055	23.45737	10.33207
0.8473794	1.380429	0.0834374	0.3099408	0.3086574	0.8191189	0.6779827	0.8993442	0.3838046	0.1998319	0.5274959	0.6118974	0.6335894	0.1055639	0.1864751	0.6183764	2.470669	0.7309196
3.755738	6.030846	5.413543	2.295376	2.167182	2.846182	3.930645	4.014224	9.983557	1.144476	2.276474	4.07928	4.59157	2.240363	1.959673	3.146541	7.114684	6.084474
2.124766	4.334169	3.553252	1.612652	1.917722	1.504182	1.330425	1.666514	4.345995	1.110164	2.488227	3.90547	2.384576	1.495387	1.534756	1.917028	2.941909	2.296701
4.892927	10.85321	4.727625	1.626464	1.051549	4.31824	3.876129	2.151326	16.83603	1.020152	1.450447	4.061127	6.058889	0.2361334	0.9991608	4.243754	12.40666	9.226844
2.084578	1.520392	2.303247	1.153284	1.841515	1.32618	1.480846	1.456209	2.467697	3.872525	4.529568	2.986807	1.897301	1.647213	2.209807	3.000176	2.415951	1.564082
0.5187531	1.100784	0.3831917	0.4970684	0.513209	0.6441192	0.7248777	0.5609044	0.8267382	0.2830915	0.4834132	0.554164	0.9179242	0.387433	0.3239816	0.6347027	0.8988648	1.249453
12.17139	32.09579	4.509671	4.260586	2.336739	7.035671	9.466558	7.511661	9.817408	1.785537	6.163518	9.598912	4.753688	4.420364	2.40672	7.378418	23.95881	10.48372
5.972073	11.13772	2.541052	2.470596	2.060159	3.714521	3.188427	2.651462	3.984922	1.602395	2.291275	5.912307	4.257713	2.222409	2.43374	4.462724	11.93349	5.244475
2.500219	2.930086	2.355312	0.8296616	1.406381	2.357975	2.1315	2.279226	3.921749	0.7733828	1.228096	2.356806	2.900393	0.616662	0.7377738	1.636294	4.848897	4.260765
2.283873	1.895956	0.8681625	1.259943	0.4987374	0.9798581	1.070567	0.9029298	2.454809	0.9072427	1.195164	1.713783	2.023168	0.3857032	0.2964294	1.896796	3.364549	2.840449
1.550075	2.630719	2.252919	1.17735	1.240571	0.8179677	2.240953	1.092733	4.281521	0.9567065	1.486128	1.907266	1.648207	0.3340351	0.8450883	1.207199	2.528064	2.583602
0.1840019	0.2190852	0.1569137	0.05264102	0.09837031	0.06767562	0.09894376	0.04144703	0.05189334	0.0524937	0.02120931	0.1843769	0.2150396	0.107989	0.1020521	0.194929	0.06760607	0.0669784
0.1405574	0.3718132	0.06686343	0.2512291	0.08002364	0.2173915	0.1291196	0.2704377	0.04422515	0.02935852	0.01581584	0.1374904	0.1720889	0.06561517	0.0152201	0.1090193	0.05041403	0.4328651
0.1076355	0	0	0	0	0	0.02471918	0.02070947	0	0	0	0.08503939	0	0.03197507	0.02039659	0.09739848	0.02252009	0.04462201
0.370833	0.7390484	0.04258077	0.1799896	0.04671481	0.09888705	0.3700237	0.07381001	0.2886809	0.08725007	0.1208644	0.4512618	0.05977731	0.05318197	0.3343964	0.277708	3.157018	0.2756623
71.22193	28.2655	48.22267	23.84532	7.826215	26.88724	51.08165	36.5072	104.291	5.922067	13.75091	26.73195	17.98272	8.80218	6.64042	28.01265	51.21656	103.3941
15.61129	11.52405	8.886347	18.68374	4.058236	5.756672	3.490854	4.810278	8.756328	5.11878	6.773022	9.198088	6.894167	6.114977	5.530161	8.670799	7.052814	7.692537
19.45263	18.21722	7.439433	8.211209	6.10721	11.09819	12.0509	8.715492	19.29856	3.163997	9.057878	16.11527	14.1498	1.697694	2.664172	11.06173	41.25492	16.50428
6.006344	5.997949	0.9362676	2.843116	1.60837	5.353356	4.148206	4.454683	3.211133	1.977524	2.531566	2.589109	5.450168	0.8813458	0.9755859	3.790088	14.29512	6.945544
196.9638	148.7678	119.519	40.6296	33.11122	60.75253	83.38316	49.35927	333.1291	15.21781	53.76278	104.8945	66.79145	38.78206	20.21225	96.74425	156.8561	227.0482
1.819263	1.435672	0.8089044	13.65842	1.254866	1.179199	0.7749715	0.7407086	2.067424	1.011477	3.362971	6.608853	1.120157	0.7953719	0.9997095	4.372442	2.718041	3.789632
2.209933	2.702854	0.3839843	1.225892	0.4770127	1.273273	1.230934	1.19977	0.8149764	0.2561021	0.5518631	2.071211	2.023177	0.437089	0.325284	1.394806	3.67945	3.122459
1.085177	4.366784	2.409032	0.5818867	0.74139	0.7792469	1.594995	1.121513	1.741352	0.8159881	1.001275	1.894367	1.703047	1.584226	0.8813047	1.346699	2.101805	1.658121
6.158975	3.898987	12.68657	2.469837	3.12063	0.5342948	0.1813395	0.1975017	2.543452	5.618548	1.306081	3.389572	6.598054	1.208028	22.99799	4.001277	1.073846	4.451911
0.1028491	0.8835698	0.07085769	0.1331185	0.04240203	0.02468335	0.043982	0.06960114	0.03515029	0	0.04190161	0.1176838	0.02901326	0.01896405	0.0564526	0.03851062	0.01780855	0.1235024
384.6288	772.0795	65.31856	150.3411	61.73802	67.5475	173.748	48.20302	136.0683	10.72199	111.6438	304.9045	7.337283	109.48	27.50643	249.1655	176.274	488.37
0.835795	0.2828389	0.4590723	0.3537381	0.137357	0.1998979	0	0.09947017	0.1280989	0.5669166	0.3257662	0.5241836	2.371455	0.2111728	2.018127	0.2105176	0.1622502	0.2786226
1.203848	3.244539	0.6891615	0.8820826	1.31276	0.4654795	3.935907	0.5763264	2.416588	0.5035605	0.7018309	2.701087	1.562007	0.6589306	0.3987704	1.183705	2.947143	1.984508
1.701804	2.791291	0.9154164	0.8803577	0.6695276	1.032584	1.310785	1.178761	2.753489	0.6252443	1.092972	1.668611	1.560495	0.6066621	0.3671386	1.374108	2.804664	1.812618
6.353419	5.915898	3.545206	4.532694	1.9447	3.213225	3.178559	2.583303	10.73112	1.088204	2.422568	4.112285	5.149773	2.191959	1.064784	5.164875	10.59932	6.810572
62.3569	145.1187	69.91917	23.21661	25.58126	41.80466	49.72006	35.13373	143.8265	12.27156	34.11204	56.78531	43.70472	13.65903	13.62542	58.80377	114.2132	79.13254
0.09334311	0.05237663	0.07770615	0	0.01550008	0.09023008	0.02143685	0.0538787	0.07709519	0	0	0	0	0.06932315	0	0	0.07811904	0.03869688
0.5200827	1.68016	0.6430697	0.6010686	0.3276685	0.1907446	0.468979	0.2737096	0.4295537	0.1230084	0.5150702	1.184361	0.5631188	0.2113015	0.3087061	0.4360143	0.6480868	0.6515819
0.3582876	0.3089184	0.2327936	0.1896293	0.3308524	0.06335462	0.4214498	0.06557317	0.06495843	0.2970637	0.07227266	0.2485508	0.0459575	0.1732827	0.3552036	0.177921	0.060336	0.6357974
16.14525	15.56585	7.599254	4.987745	5.517415	8.977052	13.15962	12.07547	17.22634	2.289226	6.20487	10.08326	11.26582	3.926068	3.603053	11.19842	42.69007	15.62123
2.725834	2.168297	2.039296	1.196829	1.724435	1.291175	1.490212	1.068391	3.736592	0.9524409	1.798317	4.197263	1.650713	0.7083038	0.949182	2.781347	3.62037	2.343977
0.2362415	0.243477	0.04013592	0.06786206	0.08166045	0.02237022	0.1660848	0.08348648	0.04778442	0.04934427	0.06835485	0.220929	0.02253803	0.05585739	0.05481676	0.07852885	0.04841901	0.1918779
26.97274	67.87599	16.34906	20.32959	7.388905	21.8728	23.97673	17.96298	36.18422	4.999902	16.12865	28.86223	24.29995	6.388977	7.29212	41.75606	60.54858	41.36877
6.903859	7.975654	9.387282	9.749354	6.912233	4.027721	9.783564	2.539452	17.31882	10.49002	8.876521	7.604244	3.599143	1.550251	4.340344	5.40203	10.61256	8.392702
1.787763	0.6771254	0.5014839	0.3008721	1.016446	0.3223358	0.4739421	0.127868	1.357806	0.7443927	0.5601961	0.8014149	0.3133987	0.242453	0.7114288	0.6013745	0.5488714	3.173232
23.38078	10.38995	28.346	13.9412	6.172361	3.220479	1.264662	1.086009	10.4609	14.02296	5.042266	12.32703	20.51361	3.496698	65.2196	17.56515	7.489003	14.18261
10.16601	7.05628	4.182031	32.99513	4.582609	5.962722	4.477408	4.086977	12.86372	2.842749	18.36262	17.04783	3.601119	2.888533	2.329416	8.198022	6.288375	11.32865
5.558591	1.136563	0.7315827	18.11868	0.8328635	1.20172	2.072369	2.185727	1.410354	10.58736	2.287613	5.797227	1.498807	1.741485	0.4711579	2.831757	3.88364	2.434729
2.477965	2.741563	2.361712	0.739481	1.596477	0.954744	1.628818	0.5862753	2.184041	1.369844	0.9724443	2.089202	0.2660591	0.319941	1.174746	1.426192	0.9562996	1.491916
0.04195451	0.05084962	0.03632328	0.0275583	0.01839222	0.04477306	0.04393616	0.1007411	0.02494917	0.02208309	0.01982744	0.05038334	0.01569006	0.01944282	0.05342567	0.07289144	0.05477443	0.02922009
262.7503	216.953	88.73197	53.74116	50.01546	97.02043	114.8679	69.44589	278.1419	28.44834	76.34638	141.6898	92.15036	49.95267	23.04696	135.6299	288.9733	297.3291
0.998706	1.068187	0.2881392	0.5328613	0.2263087	0.6189705	0.630945	0.357949	0.3632978	0.2108611	0.3578205	0.9229275	0.2486027	0.02891863	0.2172637	0.5089548	0.420927	1.69498
2.7039	5.480227	2.348806	3.245837	1.288423	3.194163	2.697785	2.223637	3.674766	1.401535	2.403976	4.836964	2.731971	1.70051	1.341786	3.682381	8.355323	1.338386
0.5514523	0.0362614	0.1004221	0.3739517	0.03863166	0.01998979	0.1009199	0.009947017	0.0853993	0.7432906	0.1018019	0.1974198	0.4481093	0.3071604	0.4114629	0.1871268	0.03785838	0.1071625
0.5489798	0.5893001	1.602861	0.1493183	0.4597673	0.1292069	0.3179327	0.2571758	0.4994199	0.3373525	0.4136071	1.093758	0.3998266	0.3687125	0.7327328	0.3887744	0.1697939	0.2572738
0.1209928	0.5038259	0.4559085	0.8515236	0.3108887	0.1551228	0.1579459	0.4190307	0.5049197	0.05586451	0.1504749	0.1408733	0.007442212	0.198633	0.3982191	0.5186153	0.1598829	0.102959
0.0197305	0.02214233	0	0	0.02621076	0.007628991	0.009062472	0	0	0.009616022	0	0.08313837	0.06148975	0.01758391	0.1271215	0	0	0.03271838
0.02771391	0.01749466	0.001922603	0.01083583	0.01725761	0.07501098	0.07319379	0.01333065	0.01144492	0.02194869	0.02182904	0.02736985	0.1079624	0.04322282	0.06695917	0.03761714	0.007248071	0.03015926
0.1042126	0.1964783	0.02776152	0.1564645	0.008306401	0.03384764	0.05743941	0.1588031	0.03442906	0.02437916	0	0.07245485	0.0194866	0.2154693	0.00947902	0.02263227	0.01569883	0.1192402
0.7147263	0.6706868	0.0860843	0.2996656	0.1606019	0.2152048	0.356222	0.1263977	0.165791	0.04002147	0.1149873	0.2691254	0.4976178	0.1843139	0.2074801	0.3467681	0.446708	0.5484725
0.2573835	0.2346872	0.1071331	0.4025364	0.02136989	0.0746399	0.6428189	0.1485647	0.07971812	0.03920019	0.1267061	0.1355671	0.00626665	0.8936313	0.03048336	0.02911303	0.0403884	0.2934317
0.02806607	0.01349864	0.006675544	0	0.01198416	0.004650866	0.01657428	0	0.01324612	0.03517332	0	0.0316773	0.02342877	0.02143937	0.09117313	0.02176865	0.005033261	0.00997306
0.09937795	0.7910771	0.08693664	0.06717423	0.06209299	0.06252278	0.1903188	0.009722387	0.1112943	0.01477638	0.06368191	0.1756615	0.01377945	0.04053023	0.0172359	0.08230543	0.03806067	0.2199593
2.905633	0.8614991	0.748558	1.150096	0.6957451	1.431411	1.110515	0.7564494	1.53275	0.2937125	1.587921	1.205452	1.542764	0.4347824	0.4595201	1.168571	1.834305	1.231345
0.7578942	0.9528182	0.9024706	0.5176367	0.3536601	0.3672364	0.9187475	0.2436515	0.9666881	0.5891277	0.510061	0.8564946	0.4036268	0.29497	0.381771	0.9115227	0.4757124	0.7636184
7.044893	17.54997	7.008617	8.084343	1.347306	3.617914	3.215774	2.996289	11.38503	1.785824	4.329216	4.618186	2.19213	1.691097	1.562305	7.223533	7.529576	9.955266
3.719169	1.570838	4.474261	4.220379	0.7330574	1.451931	4.685872	1.921455	5.773031	2.472927	1.049505	2.856874	1.431364	0.4118185	0.6937191	2.313998	4.798405	2.906993
0.1536646	0.1940044	0.09594187	0.03379565	0.03827512	0.02228097	0.1985066	0.06652271	0.1586459	0.1614843	0.04538809	0.1821083	0.1346887	0.05991411	0.1201157	0.05214369	0.2652421	0.113473
8.082858	18.12584	4.062855	2.488659	2.541769	3.981974	5.878439	2.892555	7.68598	0.5988003	4.072598	9.423973	4.962456	1.81481	1.571558	7.226611	8.683012	12.85761
2.797071	2.522488	1.417681	1.886546	0.4804309	0.6814784	2.674595	0.4580145	8.429122	1.066467	3.869001	4.27026	2.337974	0.1332737	0.6315326	2.684318	4.836914	4.645874
270.6793	216.1391	87.89522	47.39237	41.44193	92.35398	91.15681	46.93905	296.6635	23.31068	78.14096	127.4651	75.95082	47.79951	22.64954	128.0013	219.7533	273.6712
0.1294273	0.1686756	0.02317108	0.08488524	0.03605116	0.106546	0.1073893	0.1590535	0.02988556	0.0447656	0.0394623	0.1165504	0.2797483	0.1041837	0.06171078	0.1511197	0.3371835	0.2042396
0.6406047	0.8866575	0.9006672	0.4675428	0.4680523	0.2807223	0.7061712	0.2793775	0.4703083	1.040702	0.3363843	0.8772774	0.59893	0.2917983	1.246297	0.3091616	0.3038033	0.5842605

TCGA-12-3653-01	TCGA-41-5651-01	TCGA-HT-7691-01	TCGA-E1-A7YI-01	TCGA-RY-A83Z-01	TCGA-DB-5281-01	TCGA-VM-A8CH-01	TCGA-TM-A84F-01	TCGA-VV-A86M-01	TCGA-QH-A65X-01	TCGA-WY-A859-01	TCGA-DU-7304-01	TCGA-DB-A64X-01	TCGA-FG-8181-01	TCGA-DH-A669-01	TCGA-QH-A6CU-01	TCGA-28-5207-01	TCGA-S9-A6WQ-01
0.6868827	0.2937195	0.402368	0.02799619	0.04108494	0.1022602	0.0314767	0.04201279	0.1432855	0.1068583	0.00898243	0.1772231	0.02187102	0.07378968	0.09406807	0.08957386	0.3462309	0.06920222
0.1238403	1.003574	0.2840356	0.1697803	2.969107	0.3968946	0.08399052	0.1724683	0.1033336	0	0.02996022	2.571347	0	0.09527222	0	0.04268098	0.3519473	0.1420423
5.755399	0.7062084	2.633992	0.7078361	1.076116	0.5578254	0.9683566	2.360898	0.7229364	0.9437556	0.2309943	0.8228837	0.3779361	0.5871882	1.174479	0.4966546	2.996184	0.8492491
0.5112381	2.87634	0.720528	0.2641027	0.1694402	0.1632526	0.03015044	0.1135048	0.1421941	0.04652542	0.03943482	0.1874388	0.02992785	0.1748015	0.04896987	0.06128551	0.1684534	0.2591969
40.07771	14.58337	29.10189	13.54246	24.58159	23.00079	15.33886	56.33599	7.787924	35.63482	4.646766	19.29856	11.41921	11.62078	13.11603	9.843622	36.99638	17.08315
3.575082	6.168656	4.722706	2.91845	2.399091	4.070516	4.554131	6.152245	3.894648	2.600173	0.7745429	3.29361	1.359367	0.795078	3.274244	1.931926	4.04053	2.758931
3.170475	1.427379	1.880943	2.868756	2.377844	3.75958	3.304245	6.601047	1.746014	5.957829	1.227234	1.906796	4.233214	1.837451	3.701977	2.600601	2.387733	2.357645
4.336348	1.915387	5.872889	1.405174	2.392588	1.722833	2.090285	4.086367	2.130606	1.356476	1.122772	2.163907	1.489793	0.9971294	1.610655	2.16147	8.688945	2.995526
0.5285025	0.2531743	0.2340712	0.0799511	0.4570972	0.1022116	0.08651978	0.1269014	0.01520648	0.3178796	0.01763567	0.3827464	0.1349559	0.1028146	0.02408984	0.05024715	0.2330651	0.02090282
3.04413	1.343404	4.801655	1.534237	2.471397	1.644591	4.250445	8.714137	3.183356	1.100775	1.121027	3.473219	1.281504	0.7602497	2.166046	2.703672	5.135973	5.155287
0.1880709	0.0593799	0.07058497	0.01875186	0.06937007	0.06849394	0.01159571	0.04762184	0.0142662	0.1013961	0.0206815	0.05712633	0.0115101	0.03945976	0.09416781	0.01178505	0.03036859	0.03922064
56.16913	32.26331	82.14253	33.70215	68.52403	31.14853	47.94505	116.1225	68.34501	0.6314842	21.95226	74.7863	15.91209	16.76639	43.56566	41.92114	75.36816	103.2039
0.6978582	0.4515942	1.085934	0.3297063	0.7167752	0.5892474	1.015772	1.547539	1.209392	0.2696683	0.3194787	1.112047	0.4625769	0.1954768	0.4683306	1.06936	1.426432	1.477716
0.9432075	0.5039698	0.1740886	0.1346662	0.4899458	0.1207362	0.2195416	1.259166	0.2747591	0.233639	0.1512243	0.2211098	0.09768864	0.1001848	0.8213544	0.1885033	0.2260222	0.6753499
1.814019	0.7624414	1.531725	0.658963	0.779909	0.932697	1.759248	3.74926	1.952301	0.5280283	0.4220873	1.94958	0.5561584	0.3348141	1.242212	0.9158883	2.245201	2.372199
0.7191181	0.4561699	2.919111	0.07562942	0.1889432	0.1815342	0.05344852	3.038774	0.641138	0.08934995	0.03336479	0.2162941	0.04642218	0.2425111	0.9158479	0.05432125	0.5249214	0.1864306
0.1532979	0.04550519	0.5409208	0.04526643	0.1130881	0.04251661	0.03998814	0.1642253	0.04919743	0.2221418	0.06276225	0.1463442	0.07144725	0.07257502	0.07274186	0.1300515	0.268101	0.04733874
7.193883	1.668003	11.17491	3.124184	3.740771	1.87235	8.535677	7.99793	5.926181	0.7139879	1.034626	2.795872	0.8328708	2.052375	2.453034	3.30052	4.563143	4.300805
974.4585	185.5912	337.7439	106.2702	240.1375	118.8939	390.5458	837.4159	255.0004	287.5287	60.43498	331.0997	154.0261	105.682	322.9172	219.6015	596.769	426.7358
2.971975	0.4653162	1.43161	0.8051688	0.7901121	0.5313688	0.6013254	2.21272	0.8226697	0.1187725	0.1063912	0.8599522	0.1193772	0.1273246	1.2095	0.3031275	1.713428	0.7118608
43.87873	20.35854	26.5682	12.97077	17.83435	18.28698	20.30482	52.29235	23.26364	2.227782	1.593389	12.8299	5.804556	2.105506	30.1233	7.453938	28.84341	18.19669
0.08754864	0.03685581	0.08031935	0.113479	0.06458474	0.05101524	0.167335	0.1108419	0.07305146	0.07218944	0.04621159	0.1215671	0.06429662	0.1428689	0.08767187	0.1152069	0.2714273	0.06390143
5.038782	1.698391	4.675515	1.585557	0.8329214	2.667807	2.864837	6.323627	3.184712	1.204818	1.360411	2.195878	0.9866462	2.629692	2.29237	2.05095	4.850911	3.478802
21.11556	7.956644	13.9592	7.188692	16.162	6.629512	11.90386	29.98577	13.45744	13.5059	4.64611	9.235169	5.795426	3.48902	9.476532	15.38458	16.67905	19.28763
2.639328	2.625002	4.201231	3.975037	3.315982	3.041229	4.55075	6.529113	2.284016	4.370192	1.313909	3.114442	1.857418	3.379939	1.560976	2.085589	3.626687	3.885499
1.632776	0.7875984	0.7129669	0.7490279	0.4285245	0.2893173	1.693007	0.8089886	0.812203	0.4600619	0.2126985	0.6444678	0.3065078	0.04026025	1.13449	0.4761551	0.6915773	1.062432
37.08709	11.4005	21.25089	7.191182	21.69127	6.084987	19.39276	50.08108	18.9266	13.62683	3.441949	17.91386	11.63156	5.011705	14.56773	13.5933	29.55142	22.32164
10.77388	5.922489	15.81477	11.0178	12.22029	8.011296	13.95554	15.58562	10.3614	9.584941	3.556356	12.78125	5.813927	4.55158	8.124617	7.009518	14.03259	14.14021
0.1982976	0.3366962	0.1591829	0.7557665	0.2121124	0.4250055	0.05379548	0.103561	0.02481923	0.02075307	0.3550031	1.372445	0.06007311	1.36281	0.06553032	0	0.4860626	0.1592104
0.937938	0.4058168	0.4791355	0.1753469	0.3931353	0.318817	1.122857	0.9203022	0.498034	0.6841525	0.116894	1.024275	0.3779005	0.211369	0.4164045	0.6122272	0.903668	0.7538722
0.1642747	0.0507141	0.04898069	0.01351286	0.01211857	0.08884393	0.1336966	0.06863399	0.09595073	0.03438473	0.0755105	0.0705704	0.03870695	0.09689059	0.04342957	0.03396991	0.1867439	0.03768396
1.499092	0.6414947	0.7421203	1.172074	0.7117064	0.3151419	1.026775	3.493341	0.5201586	0.7249009	0.1579948	0.9847617	0.4896133	0.07612373	0.8959651	0.6650017	1.138902	1.710917
7.495886	4.574826	10.30377	4.831955	8.154634	4.41827	7.38271	17.13262	8.105004	0.9118566	2.52129	9.55055	2.849542	2.136295	5.008119	7.573002	11.81963	11.82779
1.445897	0.2300001	0.886023	0.4660604	0.2035573	0.2741455	0.8608595	0.5072562	1.160423	0.08470956	0.1068093	1.238065	0.1040266	0.1754854	0.5495512	0.2662788	1.207654	0.9747936
4.668054	2.39245	4.993569	2.379897	6.564419	2.556744	7.543868	9.472226	5.751316	6.755602	1.385188	4.578161	5.033035	1.112899	3.141494	5.266382	3.433187	6.180295
3.694223	1.092554	2.343947	2.150396	2.797832	1.598222	2.079345	3.974586	2.065526	2.894393	2.076822	2.435413	2.053802	2.01504	1.197461	1.748036	4.71692	3.229996
4.332965	3.14596	4.456119	1.660359	4.814382	2.09586	3.141856	11.68881	4.302855	0.432944	1.273439	3.237642	1.051558	1.393724	2.229743	7.993957	4.956024	6.485313
4.016254	2.301614	4.396126	5.341581	2.12504	1.560873	3.976253	3.709583	2.02613	3.439908	1.349124	2.344515	1.541465	2.552312	1.033307	1.641928	5.982496	1.927344
0.7169	0.5464641	0.6292831	0.8251024	0.992425	0.6949489	0.8763846	1.497605	1.307153	0.2902318	0.5652759	0.713953	0.3396242	0.2723566	0.4874691	0.3050324	0.911795	1.339995
16.9325	4.578588	11.89432	6.036422	4.628208	5.057121	12.71872	19.45037	10.80537	5.420013	1.974795	11.74311	3.023421	2.665204	10.19173	6.201744	18.95635	11.37691
7.955132	3.065836	4.729108	2.303522	2.280074	3.608653	3.615626	7.727751	4.738225	1.128886	1.999831	5.170944	1.476769	2.782062	3.852505	2.60944	8.93121	4.359953
2.54809	0.9832926	2.576919	1.368733	2.363402	1.67446	1.84943	5.857332	2.567546	0.7225652	1.15359	3.271793	1.158646	0.5922886	1.726783	2.264799	3.244925	4.512096
2.652885	1.267478	3.314636	1.246973	1.388564	1.315818	2.690277	2.700566	1.992234	0.3173037	0.5134419	1.881322	0.5698021	0.3952227	0.9518285	1.541256	1.687379	2.122719
4.047807	0.7483463	2.915781	0.936855	1.805268	0.9680258	1.983289	3.836758	1.553921	0.8161148	0.7149049	1.998216	0.8289039	0.7498954	0.7934388	2.546113	1.869889	2.612652
0.4784014	0.1174805	0.1496242	0.08347437	0.104271	0.09147076	0.08111492	0.2195609	0.03628936	0.1365482	0.09995542	0.2439198	0.04391786	0.06134024	0.07665196	0.02997796	0.1853987	0.0685896
0.1783726	0.08760554	0.7661495	0.1600639	0.05981168	0.13642	0.1869617	1.095278	0.08118321	0.04525526	0	0.6037254	0.07641589	0.1081165	0.4501317	0.06706395	0.1612944	0.2324884
0.08692314	0	0.0797455	0.02383366	0	0	0.02947635	0.09079124	0.03626477	0.1819409	0	0	0	0.02229043	0	0	0.1235154	0
0.4337201	0.15143	0.2131639	0.2378455	0.11427	0.2564928	0.1891004	0.8269427	0.1378668	0.007204999	0.01498976	0.3943299	0.02085603	0.1218151	0.07735222	0.37014	0.8730978	0.1302893
41.13921	21.80157	54.58493	22.39224	57.20914	33.83374	36.82878	99.04929	32.69505	26.70971	12.15384	34.56488	17.92924	8.425782	39.75292	64.87264	27.99637	49.47784
11.19146	8.676346	7.503942	6.925116	10.25487	5.377084	5.897818	7.749113	7.203508	8.371139	6.073602	8.000829	6.906349	1.926208	13.02208	5.594512	11.42563	6.439876
19.67326	7.273117	14.72603	5.599988	11.06663	6.756508	15.32382	29.25158	16.16357	3.140989	3.81078	14.66115	3.443689	3.221333	8.649706	11.52114	22.33261	25.17097
6.048494	1.586267	2.952306	1.558622	2.105362	3.754622	4.173877	14.22188	3.439749	0.5572147	0.6478253	8.481807	1.043672	1.216759	5.749336	2.080538	4.848103	7.36857
173.9393	61.7119	127.6866	60.63587	172.3458	92.01711	148.1875	358.32	133.6908	168.5794	37.5296	73.56434	71.6879	24.10647	116.3837	115.8276	126.5913	148.1103
4.503499	3.47081	5.044221	2.360901	0.8211211	1.368491	1.41871	3.207209	1.81483	2.709192	1.012134	1.749611	0.835467	1.97509	3.255539	5.626398	3.776888	1.570171
2.404714	0.7205373	1.245823	1.074359	0.5269706	1.02569	1.261562	2.191602	1.333744	0.2023215	0.2635046	2.616348	0.333298	0.4352901	1.081378	0.8092728	3.145679	1.123544
1.452247	0.8116335	1.975522	1.482934	1.847112	0.9128035	2.10573	2.336333	2.214614	1.380109	0.9692049	1.481985	2.208178	0.7705076	0.9818975	1.190869	2.081393	1.938529
1.179683	5.512008	3.597822	1.398747	13.7359	2.61843	1.383923	2.442158	0.2660378	0.4893956	0.6016465	13.04229	2.232273	1.17736	0.8288565	0.5494222	2.786279	6.783651
0.3007266	0.05305078	0.06306154	0.0942365	0.01267694	0.07572682	0.01165473	0.9393355	0.05735526	0.02997419	0	0.1189354	0.05784341	0.01762693	0.09086122	0.02369006	0.1770343	0.05420287
775.5798	174.7342	318.5719	80.15473	153.541	80.00799	250.5907	715.8952	250.7396	136.6326	26.28597	253.725	93.84497	89.50012	211.7256	93.3231	670.8568	277.9579
0.3444396	0.7250029	0.430906	0.1373712	0.4157895	0.5017689	0.05663142	0.1308245	0.1306381	0.04369423	0.06565315	2.251889	0.2599867	0.4603739	0.2713406	0.05755613	0.5042707	0.1676035
2.56444	0.6676121	2.861845	0.7682175	0.9995431	0.4109375	2.502437	4.920018	1.628799	0.4593247	0.4444695	1.187519	0.6235672	1.044341	0.8331213	1.350797	1.849193	2.440891
2.769794	1.060688	1.803973	0.7884433	1.21661	0.9825562	0.8603907	2.120098	1.534882	0.2065275	0.4910553	1.665212	0.711699	0.3470078	1.071365	0.9764575	2.493675	2.279606
6.937961	3.668064	6.151433	2.540814	5.681932	3.458735	6.827341	11.08417	8.200401	3.3993	1.831589	4.4573	2.789558	1.384131	4.340835	5.300831	5.285663	7.218088
78.36538	32.01478	97.20018	30.79572	84.13268	29.26901	55.66701	125.9802	74.8608	45.27559	18.17155	54.35252	29.56829	17.12141	43.42652	71.42076	94.26988	84.79309
0.03769053	0	0.03457825	0	0.0556087	0	0	0.1312259	0	0.05259395	0	0	0.05074723	0	0.1494646	0.05195945	0.0803358	0
2.251339	0.4504777	0.8754729	0.2794363	0.6014478	0.3414647	0.5466662	2.154758	0.7344087	0.2133154	0.3317259	0.8181177	0.3742278	0.1473023	0.5715132	0.5683647	0.842559	0.7438554
0.9632977	0.2982017	0.4345932	0.1886635	0.1288498	0.2205189	0.04666602	1.006165	0.3003154	0.1107858	0.07170677	0.6012782	0.02850554	0.211737	0.1679133	0.04742801	0.5640739	0.1062388
11.23277	7.713263	13.23628	5.162019	7.191899	10.26443	16.96425	17.44178	15.61539	11.88031	4.395412	14.55489	5.875279	4.978533	10.10845	9.70122	19.56646	17.97706
1.670128	1.371764	2.436555	1.926974	2.019517	1.677796	2.258489	4.321114	1.585348	2.170166	1.089817	2.133141	0.907729	1.286165	1.856605	2.235518	4.949623	2.249516
0.3387343	0.2073419	0.09644385	0.1409189	0.04308358	0.1076248	0.1742819	0.422943	0.1267022	0.114094	0	0.06690396	0.02359025	0.04193458	0.1543995	0.0724613	0.4232403	0.1004797
42.82164	23.03431	58.10432	18.39207	28.88961	25.08578	28.90694	65.79916	35.23107	8.983991	7.308926	24.99763	13.01401	7.064771	24.9668	39.01139	52.88182	40.12821
23.40005	6.859465	22.58106	13.74047	7.645229	7.344428	8.730334	24.54984	13.54589	10.09102	9.298942	7.083548	10.48737	12.63211	10.5056	20.4472	12.5477	10.97462
0.3577972	0.4323828	0.8897365	0.9010195	1.892781	0.7487505	1.021743	3.776532	0.7070911	0.4697135	1.357634	0.5011152	0.4563899	1.668455	0.7094357	0.2985476	0.4783162	1.266257
10.45066	17.50428	12.26512	3.962914	33.42136	11.46876	7.917235	7.19973	1.855348	4.537801	1.828975	47.81345	5.613418	3.934396	5.43754	2.912071	10.42667	14.66458
23.62663	9.243792	20.38014	5.759647	3.214918	6.299459	5.201057	9.684567	3.002703	11.10625	2.452074	5.027471	3.308112	5.827116	6.5349	31.45245	9.165512	11.14998
10.29054	6.018374	5.538235	1.642169	0.6767726	1.955504	2.435975	7.237968	0.8016021	1.654552	0.3517618	3.631237	1.171123	5.042521	8.542164	3.454119	2.496537	2.099532
2.227405	1.817318	1.595859	0.7794175	0.9858959	1.291745	0.5323288	13.58986	1.221346	4.114607	0.1231698	0.8505476	0.4427125	0.7833494	3.383624	1.242886	3.873457	0.7664368
0.02439445	0.02510313	0.01305505	0	0.01499652	0.01303026	0.02481708	0.09342671	0.01017749	0.0170202	0.03540995	0.0329912	0.005474191	0.13971	0.04657756	0.005604956	0.06355038	0.06994986
284.1789	76.45253	169.7345	107.1824	146.0278	118.9947	216.7207	308.0006	162.7856	119.2862	57.91582	119.8641	65.65226	28.99449	168.8408	114.0712	188.471	218.7083
0.7599383	0.4247151	0.6851663	0.3113564	0.5541637	0.3114385	0.5331751	4.202952	0.4373105	0.1279829	0.06762263	0.7421445	0.1999347	0.1209584	1.170028	0.09031351	1.582541	1.157169
3.757377	1.910721	3.527513	2.38488	1.721277	1.465752	2.518597	6.994832	3.6105	0.5350105	1.272081	4.820932	1.010326	0.8371244	2.369931	3.080727	7.751856	6.030978
0.09393808	0.410835	0.2872706	0.2117807	0.354191	0.2592472	0.1628153	0.09448434	0.01741842	0.5461778	0.04040194	0.3138695	0.14756	0.1124169	0.05518791	0.03597258	0.4449448	0.04788671
0.9637812	0.4016708	0.4951505	0.1902682	0.3839306	0.3861017	0.2353148	0.9798252	0.2895079	1.976967	0.2984499	0.4968343	0.3633429	0.2965806	0.3821946	0.2790166	1.1093	0.2210875
0.03085583	0.3572124	0.757237	0.0253813	0.1934803	1.013618	0.05231733	0.1718876	0.01287321	0.1506985	0.06718352	0.3313822	0.6127868	0.7200484	0.01359568	0.1807834	1.74285	0.9997963
0	0.03689243	0.02192704	0.008737825	0.04701739	0.02553296	0.06483918	0.04438081	0.01329528	0	0.02312875	0.09887141	0.05363375	0.02451615	0	0.01098298	0.01132071	0.01827569
0.005595227	0.01943248	0.01539961	0.05216173	0.002063803	0.0302604	0.07779288	0	0.01167178	0.046846	0.004060897	0.09881635	0.01695042	0.02008763	0.02342097	0.01157021	0.03379032	0.06096732
0.1009907	0.1558868	0.4493594	0.04984357	0.007450095	0.3317557	0.5137015	0.3375513	0.1601085	0.4509567	0.004886459	0.009640968	0.1359757	0.02071831	0.1601944	0	0.2511337	0.01158343
0.4273671	0.10805	0.3244779	0.400031	0.2826552	0.1269302	0.7895843	0.7029282	0.2028928	0.1902166	0.09269567	0.7210018	0.1091304	0.09825609	0.1883062	0.04063174	0.7800366	0.4901815
0.05196377	0.09023629	0.2443235	0.03562017	0.08625092	0.1092907	2.466988	0.7327283	0.1300773	1.25988	0.0125714	0.1984267	1.609195	0.05330202	0.09730903	0.04477268	0.2584368	0.0894021
0.0388548	0.07496918	0.06238117	0.01065369	0.01433162	0.01167426	0.01976397	0.0541118	0	0.03388662	0.004699998	0.05563849	0	0.02490966	0.008560088	0	0.0276058	0.01114142
0.2407638	0.04724203	0.0823631	0.05818324	0.03010369	0.03106105	0.07472592	0.3040467	0.1770607	0.04270749	0.0177703	0.06038239	0.07417424	0.0460442	0.2355451	0.09844862	0.176858	0.06552742
9.07893	0.7467585	1.023216	1.671232	0.9360312	1.005025	2.365541	2.001081	1.377812	2.073751	0.4793743	1.30532	0.5889695	0.6240193	1.970818	1.289943	0.6092325	1.551702
1.638596	0.8520682	0.9382231	0.369629	1.020188	0.4096931	0.6384193	4.25653	0.7175694	0.9648799	0.1012134	0.4659522	0.2190589	0.2622518	1.87412	0.5607291	2.559588	0.7864308
4.385823	4.73074	8.241244	6.693764	7.36742	3.641034	6.164102	14.86523	5.687774	10.39671	0.894858	4.363435	3.30831	1.924175	17.48544	4.880679	8.672428	6.394128
6.369277	1.64417	11.93098	4.202106	1.747952	1.0189	3.464645	4.790861	3.292143	1.16785	0.8992939	3.667933	0.9073282	2.60329	1.834235	2.427392	4.726064	3.422084
0.2326778	0.2244725	0.1440886	0.1339768	0.1201527	0.0745707	0.2051477	0.469862	0.1844413	0.1055217	0.1125817	0.127721	0.1488088	0.1491688	0.2511797	0.2726505	0.1405172	0.08673496
12.20972	4.451082	8.151438	5.025498	7.741454	3.157849	6.589151	20.80409	5.841378	2.187394	1.380243	9.028047	3.312001	1.50191	5.209822	4.255518	13.17381	10.40444
4.76905	1.572142	7.037706	6.055679	3.880857	0.9774834	4.322627	3.925811	2.736184	0.2605177	0.8997528	1.445225	0.5599831	1.058009	1.415551	3.136914	2.033007	2.397898
251.0476	79.49066	163.9664	94.20847	151.3538	102.876	226.938	286.7233	113.5898	121.0212	48.28364	104.6349	71.0234	26.98225	151.5227	99.62055	184.1306	188.058
0.1180082	0.111895	0.07269138	0.1516155	0.06964386	0.08644651	0.1669304	0.1878242	0.1800544	0.03293411	0.06362413	0.191514	0.04539671	0.04150195	0.07130977	0.04415707	0.1101939	0.1546892
0.5518217	0.5323618	1.740253	0.1891316	0.3561946	0.5664817	0.2806909	2.545669	0.4316677	1.68442	0.0834376	1.41575	0.1625276	0.4422134	0.5926618	0.2377285	1.372216	0.4944753

TCGA-DU-7290-01	TCGA-DU-5870-01	TCGA-DU-6397-02	TCGA-HW-8321-01	TCGA-06-5859-01	TCGA-TQ-A7RI-01	TCGA-06-0221-02	TCGA-DH-A66D-01	TCGA-S9-A6WL-01	TCGA-12-5295-01	TCGA-DU-7012-01	TCGA-12-3650-01	TCGA-DB-A4XA-01	TCGA-DB-A75M-01	TCGA-S9-A6U0-01	TCGA-76-4932-01	TCGA-S9-A7R3-01	TCGA-27-1837-01
0.5232067	0.04186917	0.0630173	0.0891849	1.382779	0.08208678	0.3879372	0.1434937	0.2033176	4.630181	3.31817	0.5707146	0.3868022	0.07105827	0.9166427	1.68645	0.4603391	0.2937129
0.06177403	0.03103366	0.08407578	0.5382782	0.7990928	0	0.4349363	0	0.07978243	0.7336637	0.115966	0.5588479	0.04734494	0.03511252	0.1860011	0.2276571	0.4178031	0.09330068
3.197249	0.5671598	1.053369	0.6512407	4.256172	0.3544333	0.6272049	0.9219501	0.5837993	4.441456	3.911695	2.009746	0.6489441	0.4988243	4.076489	2.091124	0.6024979	3.443561
4.882261	0.1188297	0.1006035	0.2101779	0.8564042	0.05678696	0.114496	0.260141	0.02863985	1.217338	0.4058811	0.9612662	0.02266083	0.01680599	0.2726426	1.07602	0.02499677	0.4354032
11.21933	16.50259	16.38464	23.39657	17.9853	6.838505	14.58663	12.87577	15.9631	41.80498	16.40938	33.74646	20.09021	16.19618	39.66651	38.40441	13.18449	21.04834
6.666798	2.064851	2.042001	2.203652	8.253925	1.87925	2.459901	2.828596	3.372475	5.612442	10.3615	4.647928	2.271877	1.624381	4.973073	1.727783	2.757149	3.406484
5.282199	1.811466	1.456492	4.588708	5.247878	1.850507	2.360608	3.638549	2.376149	3.865074	5.027308	3.493352	2.626201	2.283274	3.484103	4.170488	1.725671	4.649848
5.88584	1.549169	1.630131	1.172373	6.489965	1.848749	2.039001	3.281827	1.760451	7.0629	7.349395	4.406937	3.116964	1.996647	6.681153	6.00612	2.260945	6.034469
0.163631	0.04566885	0.2350778	0.1917777	0.337443	0.0214887	0.2816212	0.02865037	0.07044426	1.036467	0.3583745	0.8018356	0.1532793	0.03100276	0.1505446	0.08933816	0.1475605	0.08238026
8.914588	2.549443	0.7905165	1.349148	6.763075	1.567443	1.493419	5.879023	0.8243954	6.055366	15.33578	3.846744	2.522052	2.510439	6.079234	3.148697	3.88546	4.191652
0.2004199	0.01713799	0.05803739	0.07822582	0.01918652	0.02015998	0.1321038	0.02687881	0.03304421	0.04726838	0.8565475	0.01928859	0.045755	0.03393337	0.102717	0.1204827	0	0.03864319
241.7615	41.86601	6.021766	28.22079	72.94949	37.62711	29.41197	107.8083	21.87201	100.0079	272.2228	49.28566	20.78178	45.85073	158.4753	66.05982	46.18198	212.0462
1.828894	0.610732	0.4300459	0.4298165	1.162649	0.4240917	0.5278925	1.223694	0.3492935	2.196481	2.837635	0.8418026	0.7141915	0.9680125	1.036049	0.9703844	1.035928	1.5692
0.5623619	0.62657	0.1553507	0.3038706	0.4478102	0.6844079	0.3998659	0.4416288	0.2049466	0.1675243	1.659331	0.2833379	0.1792303	0.2341975	0.3269178	0.2051964	0.6853803	0.479345
4.85863	1.120532	0.4667327	1.89519	2.901167	0.8957755	1.156514	3.121266	0.4987264	1.369012	5.74791	0.9059385	1.426778	1.765758	3.010892	1.26734	1.044688	2.959675
2.456922	0.1530524	0.1404448	0.4822612	0.6466967	0.06388533	0.1453083	0.3045715	0.1142339	0.1789695	0.5165759	0.6223533	0.06778935	0.08379124	0.1775465	1.611709	0.4918682	0.3265524
0.8646775	0.08274121	0.06404596	0.4154366	0.1852628	0.05561782	0.2402063	0.2039232	0.02279079	0.2794395	0.4306517	0.146338	0.04508213	0.02674748	0.1771113	0.1589694	0.1193504	0.1066097
12.14436	3.142365	0.762147	4.682076	10.65677	3.457839	1.206948	4.494197	1.19062	13.94678	13.81262	3.721927	1.267108	1.580649	9.675001	3.902801	5.333257	3.613224
889.3175	131.2592	118.446	137.7456	929.8448	56.8549	209.4682	379.6735	107.2186	1511.12	1401.202	472.3877	338.5238	175.6449	769.1695	491.434	222.2268	736.3305
3.969794	0.2239612	0.2455898	0.2823395	2.531196	0.28018	0.4185083	2.222758	0.1919224	3.272034	5.469701	1.264328	0.5314946	0.3821068	2.056089	1.225683	0.4068083	1.496277
105.4256	5.798978	7.220777	11.87044	68.06759	5.686792	12.38625	15.3332	9.102613	52.17445	79.41669	25.5168	7.043509	8.889107	35.79233	29.50545	2.694953	52.58773
0.1310131	0.05983413	0.04322705	0.1274518	0.1161094	0.07038483	0.1006289	0.05422005	0.06152948	0.1320231	0.2533987	0.1167271	0.115625	0.1083173	0.3048248	0.0682783	0.1664785	0.04796998
9.729112	2.629678	1.999005	6.669056	6.486756	1.474901	2.002916	2.974618	1.260813	4.96994	9.559104	2.917211	1.655517	1.627003	7.361806	3.557001	2.29448	3.656088
22.70048	7.063231	8.225051	8.200955	19.01019	6.680765	5.96631	17.72949	4.912002	36.05283	19.84452	28.02563	10.51997	11.44285	32.99156	33.46575	11.07575	19.98263
8.320052	3.439281	2.541845	7.940125	2.677865	1.944265	1.739229	2.880268	2.699527	2.91444	6.074305	2.799549	3.3038	1.95646	10.53557	2.520991	2.441816	4.345906
2.145793	0.3855587	0.6608365	0.797329	2.50178	1.110723	0.8050259	0.7486772	0.2022873	3.459962	2.984408	1.948308	1.704603	0.3828176	1.084688	1.05824	0.9004342	0.5440941
47.26601	7.517518	9.942542	7.980416	50.8091	5.559587	9.246601	20.81459	6.703046	54.63106	72.93077	25.68964	20.00494	12.28104	31.6821	25.00622	15.26228	29.65776
36.24735	8.174312	7.181447	15.41165	18.74723	5.819638	4.785916	15.98253	4.120659	23.21668	24.8552	10.23299	9.169079	10.83763	22.29627	8.835606	11.48772	8.63673
0.3907136	0.1142921	0.06731261	0.3946644	0.2725969	0.08183642	0.9889389	0.04676165	0.1149755	0.7126932	0.1531934	0.4474238	0.06064836	0.04497876	0.3350607	0.1640398	0.2475306	0.3734911
0.7478709	0.416665	0.6367707	0.8582727	1.594768	0.2597305	0.8185358	0.5399183	0.1785295	1.17306	2.897725	0.7134472	0.9670787	0.8501835	0.9658334	0.3744524	0.4554739	0.2997858
0.208956	0.01234989	0.1003742	0.0338224	0.1198261	0.04358272	0.1788529	0.03443424	0.02645794	0.3244027	0.7076154	0.01853286	0.1507279	0.05123461	0.1295339	0.04529822	0.05542177	0.01237638
1.947183	0.4537721	0.453449	0.2784274	2.356844	0.7875545	1.355327	0.7233522	0.1051829	2.122068	5.212031	3.114523	2.099521	0.597579	1.917162	0.982265	0.9313858	1.498424
25.28203	5.671197	0.9216288	6.245818	13.86201	5.363815	3.133073	12.95081	2.705178	17.60322	19.16678	12.92743	4.641895	4.7192	7.997386	6.954473	8.221078	15.46196
1.794848	0.6084989	0.1049067	0.6968935	2.099439	0.6442174	0.4574165	1.174146	0.2559846	3.583288	3.147184	1.120677	0.4388449	0.5695575	1.881549	0.6492849	0.92348	0.748396
8.477266	2.540632	2.104523	3.470647	5.944419	1.945833	4.034596	7.061553	1.785602	7.561866	7.513068	4.793177	6.21831	4.82884	6.025208	4.603531	5.52436	4.423587
4.538912	0.872634	2.153408	1.246887	2.820387	1.825399	1.579259	2.796741	1.789842	4.700019	5.111017	2.784149	2.141641	2.215047	6.242076	2.458539	2.007407	4.037935
15.51383	1.849908	0.6319145	5.433433	3.95597	1.709348	2.08163	9.446912	1.550808	5.095904	8.520659	4.912428	2.961286	3.791754	3.575309	4.254737	4.295233	9.349986
2.334984	2.022155	1.259902	4.097556	3.73512	2.694438	1.335958	1.983698	2.379234	8.715125	2.775273	7.291244	3.332081	1.251295	8.777869	3.462432	3.071274	1.558127
1.505469	0.3637378	0.2884189	0.3644494	0.9137529	0.5948528	0.5222116	1.085299	0.4789588	1.125572	1.085709	0.3794275	0.3789686	0.4818089	1.934149	0.5640334	0.8420393	0.8801776
22.11848	6.516863	5.176089	6.539264	35.00639	7.178487	8.877596	12.12513	2.497428	44.17595	48.98479	13.65083	5.319095	6.461401	20.12199	7.442452	11.90628	13.88852
16.97729	3.896051	3.890391	4.674223	9.875777	2.727207	2.553487	5.36178	3.354293	7.279193	14.79907	4.639454	3.084915	2.029089	6.788017	6.822633	2.890292	7.470209
5.834884	1.325617	0.5816951	0.9033492	3.515782	1.677966	1.460113	4.591512	0.7151879	2.919067	7.998921	2.151792	2.455316	2.741974	2.719245	2.054466	2.473394	3.873116
6.793003	1.17131	0.617505	3.063346	4.380513	1.578941	1.038194	1.760924	0.5859719	4.400595	4.21723	2.508322	1.110807	0.8094826	2.001728	2.771277	1.866736	2.950418
8.759789	1.180202	0.3134687	0.9154373	1.735791	0.8892446	0.8756745	1.935687	1.060946	3.367577	5.959479	1.840524	2.318312	1.728062	3.005113	2.829328	1.921213	3.316293
0.1898243	0.0435944	0.08857882	0.1641628	0.2440266	0.04487133	0.1527437	0.0683724	0.08405552	0.1631801	0.5192526	0.257591	0.06650761	0.06782073	0.2857796	0.2598378	0.05869072	0.2621275
0.8654923	0.02438135	0.1321069	0	2.183655	0.04780096	0.1366817	0.3144105	0.01044674	0.794147	0.842747	0.1280575	0.2231771	0.1563199	0.1704853	0.1291745	0.2188291	0.3665047
0.1083975	0.0653473	0.05901254	0	0	0	0.03052805	0.04555072	0.02799952	0	0.1831415	0.09806342	0.06646255	0.02464537	0.0326384	0	0.02932548	0
0.2369529	0.6831801	0.09114075	0.2456885	0.5330716	0.1522059	0.1668329	0.1461113	0.2261958	0.122356	0.7204193	0.2796039	0.2210853	0.1112612	1.543252	0.423968	0.8988551	0.1867215
174.1618	17.23464	18.85451	45.86183	82.6467	17.08523	25.68338	68.35578	13.33386	73.5492	127.7611	49.2321	42.14448	43.18512	16.10492	48.28145	29.80106	46.12596
10.68099	5.476386	10.0108	5.385699	16.44123	5.193765	4.665285	10.69914	5.19636	19.03424	10.13762	9.739489	6.533491	6.2319	13.03261	7.359779	5.901646	10.23565
35.95115	11.42806	3.206915	7.38832	22.28372	9.648387	6.858018	33.44827	3.994709	24.13187	44.59986	15.77422	9.40648	13.44922	24.29268	16.78012	9.405777	25.59052
16.30411	2.690031	1.124263	2.729763	6.840295	1.807223	2.763621	8.364103	1.286276	2.569766	15.507	1.894732	2.828735	5.134811	3.845475	4.022998	4.532896	7.220263
171.0384	50.13463	43.38006	62.96937	271.8928	32.37902	58.18401	105.8178	35.56032	408.9987	214.4376	162.6067	80.93916	81.27533	119.2048	102.3357	81.4475	150.3466
8.487954	1.317711	1.081398	1.164881	3.348856	3.458421	1.676023	1.612435	2.130655	2.622518	9.806067	2.244448	1.271723	2.114833	8.997848	7.545814	1.044558	9.060624
7.751013	0.9110004	0.3841346	1.258796	6.222543	0.6963572	1.033336	2.064427	0.3371797	1.776582	7.642915	1.380391	0.7462858	0.8582785	1.821806	1.391193	0.8399179	2.312581
2.635354	0.9411847	1.002937	2.348484	3.79327	1.077622	1.424595	1.823842	1.016245	4.628095	6.905048	2.062088	1.416726	1.419851	1.936749	1.488272	2.213215	2.490058
4.023725	0.3355707	1.060642	3.690672	2.701333	0.1127836	2.508277	1.603965	0.9859395	3.92882	1.462947	1.546692	0.4631898	0.2531173	3.280256	2.989187	2.968811	4.900233
0.3814501	0.03014413	0.04083295	0.04324318	0.3760415	0.05065647	0.2112348	0.373716	0.0110708	0.6786995	2.321231	0.09693384	0.1708121	0.01948919	1.34857	0.08424064	0.04638029	0.07767975
1039.328	107.3412	114.4241	108.9287	1039.128	51.16057	156.2294	237.3635	92.37297	1635.627	1754.49	526.017	269.9248	78.83485	627.6581	367.875	148.807	429.1531
0.5727114	0.07323661	0.09920559	0.9216748	0.5212266	0.01230723	1.422311	0.1312714	0.1210367	0.2638292	0.3323126	0.2708312	0.06384557	0.0295937	0.3056942	0.1535	0.5704583	0.28309
8.793983	1.243065	0.436552	0.9771768	3.388926	1.110233	1.016256	2.418939	0.5276875	2.763361	7.601988	2.659929	1.381342	1.614807	2.466201	1.580795	0.8935802	2.306909
3.691375	1.292817	0.5311129	1.238247	2.218478	0.722995	0.8910889	1.938983	0.5176128	2.20457	3.751925	2.576153	1.08316	1.246923	2.746918	1.982282	1.512243	3.504465
10.72787	3.171996	2.983407	3.513079	8.28825	2.414799	2.633777	8.3165	1.107805	7.319665	6.099851	6.924533	3.643093	4.123852	6.277363	9.636947	3.529145	7.449188
122.8978	38.23011	17.31006	39.07444	157.9447	26.72844	29.06257	84.18933	17.47411	247.66	128.6844	131.1372	49.53607	51.17586	91.72765	89.62578	57.88752	75.23882
0	0.05667016	0.07676485	0.03448922	0	0	0.07942314	0	0.02428161	0	0.105882	0	0.02881866	0.02137284	0.02830451	0	0	0
1.612888	0.199666	0.1320879	0.2373799	0.8577407	0.4806716	0.6637866	0.6699982	0.1551863	0.8708742	1.704771	1.045217	0.4462897	0.5411304	1.203661	1.669091	0.6438899	1.619368
0.8686183	0.09549764	0.2838737	0.3317652	1.366106	0.1809874	0.3792127	0.1164926	0.2250496	1.944088	1.117646	0.2746747	0.1295033	0.04802188	2.488209	0.1135155	0.2107079	0.08772727
27.18828	10.08792	11.84522	10.69497	20.24314	8.466821	9.010896	19.85592	7.682928	25.77258	38.52212	11.45647	9.915826	12.43376	19.851	10.68276	12.06542	17.13634
2.940031	1.277332	2.38561	2.299421	2.561988	1.117139	1.732778	3.088643	1.836038	3.844756	3.637224	3.566682	1.999464	1.445216	7.057378	2.528782	1.654181	3.363074
0.262191	0.07024954	0.1348091	0.08016295	0.8388969	0.09640962	0.1148637	0.07957276	0.037625	0.8488341	1.09378	0.6193423	0.08931056	0.05298845	1.499964	0.04294478	0.08669491	0.1232004
92.69405	21.51173	9.690606	21.86051	75.01223	14.33947	15.02759	39.77131	10.65335	92.19727	97.77018	58.82005	26.30552	21.75425	34.90636	50.84861	26.58129	62.91892
22.715	5.358478	4.809341	8.357941	16.94806	9.426026	3.697359	5.568366	8.155592	17.20144	6.080746	9.08335	5.015272	6.00697	14.38332	23.3823	18.31178	9.16684
3.790276	0.1415714	0.4155044	0.4243369	0.4384992	0.394133	0.6514532	1.189132	0.5095396	0.2677501	1.717116	0.4036534	0.3923661	0.4618484	1.336407	0.3115624	0.1905962	1.532251
13.58708	2.173109	5.170296	14.59886	11.22861	2.195795	11.28437	2.650862	3.939344	28.14607	7.027299	16.61895	3.867844	1.670674	6.554039	21.4938	13.05284	17.42216
23.48917	6.870992	8.501998	3.485976	4.68166	14.57906	7.670278	4.616927	11.774	15.96919	36.51912	7.677218	9.389362	12.33944	26.71503	17.18163	4.477812	49.40674
13.01198	1.006341	1.21041	33.79723	2.335817	2.056329	1.750824	4.426556	2.932833	3.260983	10.11439	2.421473	3.249222	2.120167	2.794784	7.0217	1.640976	6.403105
7.386001	1.201405	1.872243	0.8929332	5.439557	0.8004258	1.609264	1.578549	0.3963266	7.40638	15.6334	2.847925	3.292665	0.9262553	15.15005	1.260897	0.8874448	1.885883
0.08923525	0.03871635	0.08280756	0.0651072	0.03421909	0.009588064	0.105666	0.1406189	0.04714738	0.03211542	0.3159997	0.009173635	0.04352206	0.05072155	0.1343433	0.01743957	0.02743339	0.07351464
314.4646	79.52223	56.53508	94.70152	437.4542	70.99417	87.24984	191.6388	43.01444	475.1943	263.4886	312.4955	106.9901	145.0431	195.4035	165.7005	112.0531	206.4966
3.594653	0.2801819	0.1541847	0.2238042	2.50938	0.2883887	0.4417588	1.698216	0.05627355	1.697336	9.430987	0.5617015	0.3740147	0.396259	2.099097	0.8885157	0.3831	1.658374
6.518912	2.283942	1.02631	2.526053	4.148889	4.030012	2.469957	5.241086	2.124234	8.151084	11.56581	6.0309	4.916662	2.907144	6.433109	5.524442	5.484465	4.902301
0.04165174	0.04184949	0.1629806	0.8930217	1.522685	0.06153615	0.131967	0.03828751	0.06724259	0.5936158	0.2736692	1.354156	0.07182627	0.02367496	0.1567663	0.1662917	0.1197258	0.09436333
0.2019164	0.4250716	0.3795031	0.5379717	0.5083276	0.1250064	0.419724	0.2828305	0.2607792	1.21807	0.4332	0.8915874	0.7221811	0.1311655	1.751529	0.3189115	0.3121471	0.2033103
0.03078302	0.2938271	0.1780595	0.2399979	0.1558178	0.1910107	0.1300415	0.00808476	0.5963535	0.1706118	0.7223472	0.08702598	0.1769457	0.6211488	0.2896481	0.1418066	0.3122973	0.1394798
0.04768845	0.02395743	0.021635	0.03645098	0.1877477	0.009393969	0.0447684	0.01669967	0.0102651	0.2265501	0.1417458	0.01797586	0	0	0.1076921	0.009763733	0.04300487	0
0.1004764	0.009814919	0.04368421	0.05119987	0.0156973	0.01484435	0.11594	0.004398137	0.03063951	0.03314765	0.1964797	0.03471779	0.04064273	0.004759255	0.01890835	0.01714295	0.02831514	0.008430833
0.02015047	0.01012307	0.3633842	0.0323445	1.065311	0.01786215	0.1702496	0.03175355	0.1106052	0.7259328	0.704541	0.2962281	0.1698813	0.05154111	0.1516822	0.03713047	0.115843	0.06086871
0.8086112	0.1994196	0.07003418	0.2056478	2.369	0.07819459	0.3260673	0.3745456	0.06171087	2.857777	2.818603	0.8229623	0.1633852	0.2172733	0.6861487	0.1760904	0.2237305	0.3663868
0.6933754	0.03906546	0.617373	0.3863449	0.1239159	0.007658995	1.268379	0.3676159	0.04184619	2.390949	0.9062869	0.2638061	0.3575895	0.07366661	0.1463374	0.119407	0.2542016	0.1370224
0.2131971	0.01460518	0.01978403	0.05777623	0.04905289	0	0.04093828	0	0.006257919	0.06138305	0.1273449	0.05479316	0.01485444	0.005508263	0.05106298	0.02380908	0.03932562	0
0.197449	0.04704008	0.08311308	0.0485438	0.1717267	0.0962344	0.09459034	0.1026456	0.0920137	0.2610947	0.3993229	0.09667855	0.09672584	0.02776838	0.4259685	0.125028	0.04956229	0.09223235
4.511413	0.5459144	1.003032	0.7926073	2.694993	0.9871761	1.721471	0.622414	0.5188308	4.022799	3.979555	2.05896	1.28471	0.9166423	5.921712	1.001181	1.020342	8.258661
2.197029	0.4310081	0.7968617	0.5742455	3.090782	0.3699794	0.7591529	0.8952202	0.1572234	2.386711	8.265121	1.061963	1.137376	0.5206067	4.939609	0.7762062	0.4861654	0.4552803
12.62561	5.005366	10.22414	2.683597	15.03206	2.834951	2.52392	6.507299	5.140381	22.33061	22.1924	9.955456	6.060465	2.277281	10.83326	5.342628	5.989934	10.90803
24.4953	3.143181	0.8264555	5.694023	18.58243	1.435396	1.412401	3.440245	0.6792186	31.98963	10.23395	7.197842	2.144144	2.163364	1.583499	5.827718	3.622924	2.440235
0.3830124	0.09912311	0.102678	0.1383947	0.561385	0.1920499	0.2451545	0.09754496	0.03747488	0.2664997	0.8061678	0.09187443	0.1334313	0.07916555	0.6377806	0.04990235	0.3218463	0.105179
22.2446	3.905761	2.217325	2.91927	31.11096	3.18079	4.854412	11.28371	2.45034	30.73503	36.57455	18.87393	7.433797	5.431089	11.05266	9.567532	5.658122	15.43853
10.12046	1.452644	0.2083192	1.982399	6.044622	3.607209	0.4855986	4.637951	0.7383277	12.67085	2.814502	4.104015	0.4975039	1.436028	4.342126	3.481882	2.01056	3.386871
324.3166	57.57436	41.79669	86.05274	431.1514	51.80482	68.86967	140.3764	34.30605	519.3612	278.7401	316.5149	98.28254	98.46213	183.1886	149.587	93.20611	191.9606
0.3481437	0.06590371	0.05722597	0.1172409	0.2062093	0.1411347	0.1563082	0.1696193	0.08905809	0.1065315	0.4309686	0.08368326	0.1031207	0.1013328	0.08608879	0.144624	0.1501509	0.2286176
2.150467	0.5531333	0.7258543	0.8205468	2.457649	0.1830006	0.5329606	0.4518337	0.1555328	1.743543	4.42454	2.256724	1.028454	0.2738022	3.47062	1.01442	0.255983	0.6236097

TCGA-RY-A83X-01	TCGA-S9-A6TW-01	TCGA-P5-A736-01	TCGA-TQ-A7RG-01	TCGA-19-2629-01	TCGA-DU-5872-01	TCGA-HT-7473-01	TCGA-S9-A7J1-01	TCGA-RY-A840-01	TCGA-12-3652-01	TCGA-TM-A84B-01	TCGA-S9-A7R8-01	TCGA-12-0616-01	TCGA-S9-A7IX-01	TCGA-DB-A64P-01	TCGA-HT-7611-01	TCGA-TM-A84I-01	TCGA-TQ-A8XE-02
0.02099223	0.1346225	1.162279	0.1680462	0.04667119	0.6344138	2.085741	0.1075853	0.0145953	0.7191088	0.1459415	0.06791764	0.6780942	0.1620864	0.1245883	0.1456256	0.1833938	0.1665551
0.03111912	0.3741859	0	0.3018106	0.1383718	0.9248914	0.1932453	0.03879378	0.4283975	0.04657346	0	0.04530684	0.3185538	0.2073637	0.1511108	0.08447361	0	0.1129896
0.3621157	1.012416	1.035688	0.5417284	1.585459	1.224505	1.722154	0.4597299	0.3058296	2.097975	2.717598	0.7374474	2.442416	2.193037	0.7497438	0.6814469	0.5497092	0.8146896
0.01489462	0.0582067	0.1625963	0.01547747	0.6871282	0.2448172	0.3738278	0.009283983	0.02330057	0.7857779	0.07090916	0.2710665	0.5870103	0.198502	0.0949286	0.07412499	0.4090275	0.1577347
8.002393	30.1734	39.87515	11.01501	38.50932	17.96166	22.81413	4.219597	4.41911	27.62733	4.907208	11.43788	97.91572	27.05996	10.43757	10.92752	4.578148	13.59219
1.127716	2.267355	3.804764	2.262535	5.659256	3.060969	7.536138	1.870342	0.8248914	1.322326	1.919808	3.857035	6.518948	4.15835	2.109207	4.435374	2.239064	3.773448
1.604003	1.583836	4.058614	1.464403	3.572021	3.228878	4.232719	1.807573	2.326448	4.173197	2.282918	0.8892679	2.207394	5.632368	2.024586	3.604393	1.854662	1.639186
1.539927	1.840828	4.059509	1.703122	3.463701	4.035029	5.778646	1.32471	0.732562	3.443548	6.112366	2.832006	4.203629	6.785196	1.842096	2.265277	1.530667	2.152588
0.07327137	0.09911675	0.3646435	0.07613858	0.04072533	0.4784339	0.1611475	0.04567086	0.1146229	0.2604409	0.1328856	0.02666925	0.3000198	0.06974962	0.100068	0.008287373	0.04236077	0.09976464
0.9666628	1.155141	6.599817	1.856784	6.587301	2.72336	9.142754	1.675636	0.8723326	0.9415675	1.086662	3.855757	4.767753	5.245524	1.966341	3.160764	2.565684	3.287444
0.02577778	0.020664	0.03310609	0.02976277	0.08596605	0.4836751	0.1511832	0.02142343	0.02150707	0.08358893	0.0506467	0.01876514	0.07036711	0.06952654	0.04694021	0.03498721	0.01490304	0.02599884
14.01764	4.589691	116.9628	28.85611	191.6862	59.15629	114.8093	25.60639	8.291827	27.4014	5.304254	61.51579	71.03711	147.2766	28.76114	71.24578	66.70601	90.35339
0.3965851	0.4833543	1.680441	0.6503955	1.607475	0.8285583	2.448776	0.5246608	0.189075	0.3795406	0.1541251	1.323688	0.9721143	1.181366	0.5272946	1.132686	0.6488465	1.322403
0.3702455	0.1888705	0.2125349	0.3186683	0.32739	0.29809	0.6096289	0.2167916	0.7160989	0.1889033	0.3535446	0.3716154	0.3330653	0.2055957	0.2655943	0.139589	0.2951323	0.7841833
0.4790618	0.5550919	3.176944	0.8809694	3.934337	2.071011	3.648015	1.096693	0.2289155	0.3172062	0.3685948	2.016334	1.295835	2.965615	0.7930461	2.75585	1.433491	2.270021
0.01980307	0.08929437	0.1780299	0.123468	0.6549073	0.4993904	0.3176835	0.1234348	0.05576249	0.08891297	0.3950653	0.1009107	0.5574692	0.1036819	0.132222	0.07615424	0.200355	0.3595122
0.02963181	0.04275625	0.07611154	0.1149543	0.08564297	0.1868124	0.3496177	0.05910347	0.0296671	0.2217376	0.08061075	0.1121677	0.3033288	0.2369436	0.1366942	0.02144967	0.09593469	0.02869049
1.681055	1.222964	2.77017	2.531185	8.788914	2.754962	13.05286	2.420991	0.9750588	1.780659	0.9988567	10.46603	6.105403	7.03674	2.129278	2.781716	2.600548	11.62211
19.96231	140.8786	384.1181	104.1013	1232.386	337.6163	565.2168	100.3749	130.3493	198.1831	82.54433	270.512	1041.051	658.082	102.6566	135.8713	303.2366	405.2026
0.1425891	0.1843126	0.897315	0.2420091	2.615355	1.16542	3.361052	0.3244025	0.1382978	0.6135296	0.3652738	0.8355815	2.656522	1.021411	0.4500577	0.6386519	1.244781	1.803402
2.941021	5.210439	30.61189	10.14829	71.95628	25.77787	38.19326	6.816887	2.840962	9.564667	2.808896	20.99424	46.70443	36.26254	9.84891	24.36528	34.34235	23.10286
0.05999889	0.1539083	0.1130153	0.1274644	0.08448232	0.1080744	0.2401101	0.09972794	0.07008209	0.07183638	0.0290172	0.1747068	0.1228369	0.1523069	0.08254851	0.08686319	0.06937496	0.07745719
0.7009534	1.75794	3.546605	2.247581	5.199619	2.661498	6.275892	1.694941	0.7268522	1.172283	1.218791	1.982743	3.968097	3.762089	1.585715	2.696073	1.510345	2.817086
5.185963	5.993262	29.23735	7.630944	31.57335	8.683983	18.24899	6.70438	4.188567	12.66978	4.911661	12.00363	31.26212	16.99963	5.928366	11.13159	8.444898	14.81376
1.977357	5.357081	3.382483	2.474396	3.374661	3.470623	4.356281	1.564928	1.276682	1.072406	1.359068	3.14565	2.480483	3.803686	4.140204	1.846113	1.555864	3.435849
0.6154365	0.74002	2.350926	0.8964202	2.227833	0.8030021	3.503292	0.5016418	0.2863609	0.4723456	0.2790402	1.298086	1.518455	0.5708339	0.5651309	1.599224	0.620379	1.079088
3.250658	7.488474	21.86246	8.30253	55.73038	17.86962	38.03498	9.127685	8.23186	10.57732	5.330107	18.69921	41.16807	26.92324	8.317149	14.09651	15.40234	18.96289
7.687463	10.47373	17.81083	5.888913	17.3672	12.7524	24.0861	4.546228	3.144429	4.734045	6.773072	18.11583	9.578167	11.2744	8.287924	11.24336	8.572038	12.03416
0.004982906	0.08388243	0.1791856	0.1173656	0.2049485	0.3365841	0.3197458	0.04348262	0.1434292	0.2684704	0.4202225	0.1233298	0.5304832	0.2276835	0.1693749	0.04959615	0.1037086	0.1145846
0.3285362	0.5839754	1.201598	0.5244577	0.4882709	1.193216	1.13096	0.3828501	0.3530609	0.2458468	0.3270623	0.6394948	0.5702636	0.7036769	0.5765746	0.8918198	0.4252969	0.5532166
0.01651186	0.09927187	0.05301495	0.03431598	0.03671016	0.3160126	0.2606131	0.04631412	0.05682713	0.03088995	0.04866233	0.04807978	0.1183177	0.1296754	0.09521337	0.07470308	0.0859148	0.0649485
0.5967497	0.6906423	2.069276	0.6511064	1.252101	0.8330547	3.975941	0.6416323	0.2987303	0.7144856	0.2570392	0.6190327	1.374454	1.796513	0.4074958	1.262166	0.7245044	1.011137
2.923921	1.569378	13.20291	4.550167	20.58535	5.209532	18.67904	4.268377	1.081867	2.283128	1.694606	8.982854	11.39113	11.80316	3.77076	9.670171	4.98797	9.370113
0.266258	0.1734183	0.9759878	0.4380719	1.91154	0.7693637	3.570937	0.5186299	0.1874356	0.2075452	0.1207219	1.025656	1.385501	1.050805	0.2087524	1.671392	0.538766	2.01406
2.373509	2.490347	7.861946	3.466275	7.558844	4.027732	8.327528	2.399084	5.000399	3.26422	0.9639683	4.629746	5.403426	4.431619	2.035873	4.776241	3.58152	3.793212
1.517999	2.401696	2.814385	1.623523	2.080769	2.042182	3.303626	0.8584441	0.7856146	4.646222	2.166565	2.271009	2.827423	5.026845	1.985982	1.593867	1.596852	1.920102
0.9355664	0.459033	7.83761	3.449177	14.48833	2.760259	8.842594	2.399625	0.3364515	2.116374	0.7411098	3.107795	6.098468	5.983391	2.251919	6.660417	3.133397	2.869814
2.009163	4.167811	2.776998	2.417265	2.481033	2.666229	4.009997	1.671706	0.9349322	1.444448	1.981809	4.728917	3.60041	3.03453	2.895334	1.10422	1.180346	5.267685
0.4403561	0.3048624	1.062537	0.4252331	1.57732	0.6249467	1.160112	0.4824174	0.3618339	0.1131694	0.2299097	1.411763	0.9835086	0.8087415	0.4265837	0.7486078	0.9669103	1.103602
3.519036	4.556891	20.72682	7.944728	17.00369	11.03271	38.48098	7.366965	2.819918	4.365105	4.912807	10.66871	16.16545	15.55542	8.553274	13.64053	6.736044	11.32365
1.748799	2.087141	4.826641	3.329334	9.042143	3.910586	8.259484	2.826646	1.904834	2.355947	4.301325	3.737576	4.876959	7.571978	2.850693	4.572647	2.673009	4.769347
1.198212	0.7383936	6.650717	1.919529	4.699353	2.141446	6.921428	1.759787	0.6138782	0.5940199	0.6519266	2.267847	2.230809	4.455551	1.200041	4.485846	2.24704	2.383743
0.98407	0.6565276	3.090267	1.389826	3.493492	1.349447	4.133095	0.9972401	0.3337111	1.11171	0.483601	2.73604	2.87258	2.326819	1.364193	1.901494	1.512241	1.990141
1.167982	0.2883243	5.761688	1.253875	3.929486	0.9683306	4.0588	1.263181	0.7550591	0.9608346	0.9188491	1.632916	1.868645	3.497522	0.8263239	1.658747	1.261066	1.750615
0.07650028	0.1182681	0.07719517	0.0908501	0.0911143	0.2854385	0.1922845	0.1089908	0.02051556	0.1226698	0.1288315	0.07160016	0.1342461	0.2756883	0.1194032	0.04944331	0.04422752	0.0462938
0.07334546	0.1959841	1.13036	0.06774717	0.4348428	0.3890059	0.7169355	0.1015934	0.05099502	0.1341636	0.05172999	0.3915445	0.5005386	0.1706715	0.3066908	0.3981961	0.2544216	0.009863261
0	0.183848	0.1122078	0	0.04856137	0.01967208	0.04521276	0	0.02733554	0.03268977	0	0.0954022	0.2683104	0	0.07954819	0.0790557	0	0
0.01037972	0.2308966	0.4665691	0.2948143	0.2711526	0.2570797	0.859421	0.2264428	0.0129901	0.1165086	0.06118043	0.181344	0.2550072	0.4347562	0.1827098	0.314632	0.1200176	0.288937
15.89748	12.38405	78.74792	38.43908	71.63858	61.10489	97.09343	31.66642	15.42438	25.8903	11.16845	23.04585	49.34578	34.08264	30.8474	99.88087	31.21939	24.2783
7.752658	8.362416	6.588957	6.204372	7.723891	6.098127	9.852776	5.424514	4.803623	11.05297	6.392607	8.179548	11.89116	7.548855	6.320471	7.204056	7.719148	6.853249
4.581913	3.504708	35.89177	9.985725	26.67704	10.92107	35.06311	7.438498	2.323959	4.43671	6.283962	17.01559	13.26982	21.33659	6.9788	14.51496	12.06294	15.70923
0.938496	1.44076	9.339173	3.03402	9.336852	5.140571	12.2362	3.384761	0.376732	1.492909	1.107948	2.853053	3.782381	6.0402	2.608225	9.335752	2.750425	3.814752
35.20482	79.10085	149.62	68.51195	233.0616	131.3011	168.5664	49.98518	54.19284	96.06399	14.74382	100.0936	285.3183	94.09619	64.21415	76.19693	111.3196	114.235
0.6622773	1.774376	5.272614	1.937631	3.327229	1.841532	4.638375	1.775462	0.3017595	2.463508	0.4868585	1.057834	7.299444	9.052705	2.73199	1.917038	1.315938	1.105047
0.3092424	0.3675686	1.364939	0.876615	1.995406	2.090463	4.410921	0.8330527	0.2090762	0.8032818	0.9315178	1.278242	1.85569	2.473458	0.7378774	2.794937	1.907029	1.639059
1.384205	1.271005	1.599947	1.237871	1.510748	1.575333	3.998312	1.254968	1.385854	2.052795	0.8900534	1.483985	2.344417	2.431708	1.649833	1.69655	1.455015	1.736211
0.3525183	2.215731	0.4733139	1.753861	2.600592	5.498365	4.494266	2.936373	1.744638	1.942474	0.2906047	0.3732635	4.72397	2.043955	1.575623	0.3044745	0.8152132	1.454488
0.008636326	0.1038459	0.188556	0.06581136	0.1104046	0.4550242	1.376512	0.01076624	0.005404133	0.1357155	0.231028	0.02514754	0.4862362	0.1109865	0.07338975	0.08595958	0.09486634	0.02613117
9.482918	102.8047	248.3957	82.93236	899.1491	336.3978	583.3885	70.34883	117.9842	218.4448	69.95804	197.2124	1081.801	530.0829	81.37891	148.1026	278.4598	314.1339
0.02622796	0.3342955	0.0673685	0.04360686	0.186597	1.218888	0.2497369	0.01961781	0.1116016	0.03140261	0.00951345	0.16038	0.6228852	0.5892288	0.1400959	0.1613786	0.02426129	0.165066
0.3000804	0.4070857	1.729312	0.9008217	1.680722	1.62158	5.013017	1.237364	0.5392474	0.8406343	0.596558	1.478711	2.110879	3.973564	0.8079741	1.486426	1.396793	2.342075
0.5525541	0.3960895	2.429147	0.7361234	2.126201	1.358968	2.804413	0.5960996	0.465443	0.5566096	0.443244	1.531592	1.957941	1.699371	0.657885	1.249935	1.093505	1.556133
1.632375	1.905088	7.746036	3.093165	13.26924	2.572809	7.615035	1.773104	1.689898	4.805251	0.9197108	4.089153	10.23485	5.221613	2.302218	4.121595	3.129587	4.467
19.62089	23.31496	129.1443	32.35596	162.2601	49.44441	122.5787	24.43769	20.04018	56.8681	14.79469	66.37027	133.6097	66.23522	26.7966	64.67916	35.92119	62.2283
0	0.04555307	0.02432707	0	0.04211315	0.05117976	0.03920919	1.227908	0.2607637	0	0	0	0.1551219	0.01803163	0.04599026	0.03427915	0	0.02292542
0.2328091	0.1903571	1.178036	0.2838519	2.836422	0.5074168	0.8385126	0.2031575	0.2563951	0.4250804	0.1224451	0.4880889	0.648223	1.657712	0.2430562	0.353901	0.5599159	0.7551349
0.0478803	0.3358411	0.1093193	0.2911526	0.1774173	0.4240404	0.7323125	0.09284902	0.03328982	0.378198	0.7742889	0.03485486	1.127302	0.324117	0.09364633	0.09868265	0.1414822	0.04185213
5.683927	14.75599	20.54424	9.259124	29.02722	12.73769	25.868	7.423334	4.388634	3.247907	3.801868	16.59161	13.09862	17.69872	10.39288	14.02564	7.702765	13.78395
1.329619	2.333347	3.036123	1.244728	3.737911	1.627495	2.916958	1.158353	0.6521094	8.028879	1.133548	2.036715	2.306999	2.682917	1.620351	1.681545	1.199053	1.941633
0.01761075	0.2399914	0.1357036	0.146399	0.3654305	0.2854959	0.6136324	0.08781579	0.02203965	0.1054263	0.1384023	0.07691933	0.5167857	0.09499766	0.07126312	0.1062329	0.08823875	0.1278848
11.4878	10.77718	50.03781	19.34531	85.15312	25.79805	72.37597	19.42812	7.994115	20.87167	11.236	26.00086	67.64761	44.85682	14.51424	46.51108	18.98677	34.82977
4.887006	8.670745	13.06068	8.323862	20.57587	7.75629	7.028227	6.760035	2.887841	29.03393	2.667903	14.87806	12.77231	15.66656	6.973155	4.228086	4.726388	6.004467
0.1987458	0.250358	0.8690546	0.6261252	1.699071	0.5220767	1.26112	0.2802058	0.05033784	0.3151519	0.2853539	0.7647299	1.007552	1.236512	0.3274401	0.580177	1.058739	0.4524447
1.815911	7.894221	4.44903	13.04428	17.82598	24.80891	28.53834	8.428135	5.768889	26.75916	2.077345	2.318422	15.90053	7.792133	9.597895	2.350914	3.52101	6.931462
3.96382	12.13602	27.66399	7.776456	16.56037	4.52929	18.6689	10.3057	3.555829	12.86147	2.119548	3.497461	31.13297	24.72711	17.10685	6.624769	6.328287	3.378391
0.5350036	2.196651	6.904504	1.578927	1.871211	4.352264	8.067148	1.361007	0.4735849	4.673985	2.078623	1.146216	3.223634	2.910814	1.731944	5.887837	2.861695	0.8054393
0.2771906	1.999816	3.190242	1.240777	6.399695	3.072591	3.409527	0.5847804	0.4536398	2.521004	3.006625	0.1707396	8.534293	1.644098	1.061274	1.340888	1.257375	1.48386
0.01021657	0.04176805	0.07347769	0.06228262	0.03407113	0.06625019	0.07190257	0.04585033	0.02301466	0.01223225	0.04632214	0.01784937	0.02928321	0.09725514	0.01736367	0.133119	0.03071411	0.01731103
52.37998	63.24466	273.5965	111.3019	462.3542	154.5976	265.524	68.05267	45.53045	115.728	25.96097	161.7335	324.8799	131.7019	85.35538	129.2289	135.4694	178.3624
0.05267876	0.1530779	1.020458	0.3953456	2.264293	1.024009	2.094526	0.3611879	0.005493908	0.2102401	0.7603285	0.4921319	1.455978	1.512761	0.1385594	0.7705993	0.7207791	0.8979059
1.613071	1.059171	8.003773	2.85277	5.520174	2.43454	13.60094	3.006041	2.900648	1.697323	1.981938	4.151951	3.392642	4.826731	3.468659	3.0935	1.776027	4.864056
0.02098237	0.3342955	0.0404211	0.07267811	0.4606614	0.0661412	0.1302975	0.09808907	0.0722128	1.452371	0.08562105	0.02291143	0.4403154	0.139817	0.1783039	0.004746429	0.01213065	0.01269738
0.271245	1.013405	0.4230045	0.4429215	0.3230615	0.9335488	0.3910198	0.2415286	0.2788422	0.4784415	0.4919323	0.2256629	1.11065	0.4241984	0.6585672	0.2366683	0.1456151	0.4338057
0.03876792	0.2424019	0.9360365	0.6230741	0.1551441	0.1606127	0.5135857	0.9085844	0.009703526	0.4757706	0.2109295	0.1015971	0.1904888	0.5092829	0.1223642	0.09822072	0.01793048	0
0.0160156	0.009628829	0	0.02218975	0.02670513	0.02163635	0.05801519	0.009982703	0.08017339	0.01198463	0	0.04663476	0.08197262	0.03049164	0.01944248	0.01449158	0	0
0.02108988	0.05240888	0.07945076	0.02922021	0.003125887	0.07091213	0.215365	0.02629112	0.01231709	0.01262541	0.04717352	0.02251708	0.02878515	0.02141462	0.0648597	0.006361002	0	0.01191162
0.02537736	0.1220581	0.01955511	0.04219266	0.06770458	0.379406	0.08404791	0.03796319	0.3938177	0.4253788	0.1840984	0.1403998	0.3013423	0.04831523	0.06777626	0.101035	0.04694896	0.2088552
0.09998437	0.08905505	0.2901086	0.1539213	0.6380582	0.6403525	1.513895	0.1292591	0.05097862	0.2549397	0.1007403	0.5445355	0.697496	0.2643852	0.2697292	0.2781115	0.2440634	0.4930044
0.03917303	0.3375706	0.3605511	0.1990067	0.1016071	0.5938912	0.6959909	0.02441699	0.5474415	0.6351269	0.05920379	0.07604355	0.3742653	0.08079543	0.3645878	0.02363026	0.09813836	0.07111615
0	0.005870028	0	0	0.01628027	0.06595091	0.1010509	0.01217152	0	0	0.02213419	0.01421499	0.0199892	0.04182442	0	0	0.03386809	0.0118168
0.02871194	0.1011064	0.1422298	0.05967095	0.2256992	0.1422246	0.316265	0.05880269	0.06929873	0.1043577	0.08926666	0.03881631	0.2309313	0.1425164	0.04979352	0.1206203	0.05691218	0.1290707
0.6522423	0.5847061	3.130032	1.307119	7.635705	2.121578	2.977476	0.7876904	0.4008489	1.90438	0.3089302	2.361308	1.73476	1.225154	0.9544058	1.79424	1.114419	1.638716
0.2569808	0.8216635	1.447664	0.6069017	1.356921	1.204573	1.988723	0.5096604	0.1681136	0.5769034	1.519999	0.3741425	2.690395	1.11751	0.4112295	0.3857832	0.2971393	0.8341031
2.071395	6.130978	8.969877	4.599255	15.52796	6.768685	13.44026	4.568591	3.689082	7.551499	2.287734	4.253021	16.96323	7.12894	4.868034	4.70974	3.654053	8.260213
1.207202	1.018075	6.790873	2.096412	5.386066	4.496593	7.999718	0.9192983	0.4580255	3.270085	1.089743	7.046249	9.483513	4.31072	2.612717	3.420318	2.096937	3.074189
0.1520175	0.1124865	0.1276532	0.1296132	0.1624877	0.1474443	0.1875912	0.06559907	0.1024414	0.2012609	0.1696617	0.06809994	0.4189613	0.1447109	0.02129364	0.06877588	0.09464721	0.07783998
2.043099	2.793959	11.84325	2.590838	17.07997	7.510059	20.53989	4.118325	1.976192	3.959515	2.04738	8.469994	21.97073	12.7704	3.754025	6.009779	6.294328	7.75771
1.044176	0.388729	2.293101	0.7027636	5.675635	1.037478	5.501559	0.7944498	0.2883276	2.624948	0.6874027	10.78771	6.923001	2.555717	1.055581	0.9464952	2.090306	6.856219
44.44245	63.5723	208.8854	93.25589	372.0713	145.9054	213.2592	53.52021	37.75153	105.3908	18.71687	146.9997	354.0571	137.7209	81.50226	110.3089	140.4949	158.026
0.01863941	0.04686273	0.09140106	0.09625705	0.1224371	0.1327726	0.3647819	0.1436426	0.04241276	0.06086421	0.02612174	0.3009787	0.1595816	0.1806613	0.07405432	0.2821174	0.1645805	0.05537237
0.1039981	0.937879	1.090768	0.3842406	1.175342	0.7493158	1.076355	0.151254	0.2169207	1.297046	0.8330355	0.1009417	1.951742	0.6764963	0.3366684	0.2666217	0.4409164	0.1678239

TCGA-HT-A617-01	TCGA-28-2499-01	TCGA-DU-A5TT-01	TCGA-19-2624-01	TCGA-KT-A7W1-01	TCGA-S9-A6U1-01	TCGA-DU-8167-01	TCGA-FG-8186-01	TCGA-06-5416-01	TCGA-02-2485-01	TCGA-S9-A6TS-01	TCGA-DU-A7TJ-01	TCGA-CS-4942-01	TCGA-P5-A5F6-01	TCGA-TM-A84J-01	TCGA-VW-A8FI-01	TCGA-DB-5274-01	TCGA-DU-A6S7-01
1.948751	0.7916408	0.2071055	0.1535535	0.1856804	0.01345555	0.08230336	0.05950766	0.4730022	0.2456135	0.2364956	0.1652081	0.0220422	0.3840773	0.04294715	0.09483884	0.1944175	0.02540925
0	0.1582297	0.7948756	0.3819543	0	0.3590397	0	0.381088	0.1838054	0.4915353	0.07424125	0.03104447	0	0.2372335	0	0.4866582	1.715294	0.3013356
1.426854	1.829935	0.9295458	1.990521	3.210198	0.5767099	1.15756	0.9431263	2.974257	1.579064	0.8188535	1.748613	1.11689	1.636009	0.6866221	2.421527	0.4689071	0.8900634
0.1835878	1.571478	0.6657932	0.6647845	0.2626578	0.03866581	0.3241017	0.2964018	1.107022	0.6077673	0.03997606	0.2340275	0.08093495	0.3122561	0.2595586	0.3785119	0.1001214	0.03605725
15.98107	33.46512	12.91033	14.29134	10.56934	8.485529	17.64517	14.43532	19.76625	16.9826	13.18453	11.14104	16.58591	59.92233	5.55089	47.42801	25.12632	12.94864
9.781659	2.797047	1.922753	2.234739	1.001264	3.34153	3.942727	2.748198	6.22459	2.782985	2.872235	4.257862	6.354238	1.639784	3.72555	3.026127	2.582489	4.906486
2.335528	2.065954	2.8296	2.417965	5.228965	3.174266	4.210546	4.449982	3.273049	3.747221	3.383199	4.795168	2.268692	1.425246	2.368035	2.657939	2.077872	3.304415
7.349924	2.41539	6.517388	3.180308	5.903019	2.436475	3.278271	2.283743	4.583259	4.344343	3.598627	5.345372	5.246575	3.748396	1.421266	5.414983	2.13686	3.041175
0.231884	0.2910618	0.2673672	0.1021965	0.06460487	0.06340314	0.0754089	0.1775884	0.5409728	0.4942815	0.02185055	0.0182739	0.06924257	0.04189328	0.02409154	0.1002626	0.8619257	0.05543034
7.90954	3.259834	3.233407	1.502038	1.50742	3.016369	3.739839	3.640222	0.9280075	2.594735	3.55933	3.478811	6.320969	1.452536	0.9324986	3.395127	1.535865	3.256345
0.01717466	0.0819193	0.06793446	0.02876318	0.03030506	0.02974135	0.03537304	0.05261289	0.1691742	0.0169653	0.02562431	0.1114357	0.3451056	0.01310096	0.01130093	0.02687515	0.02887963	0.03120173
49.58816	64.25503	40.92076	18.90664	50.93066	51.41989	70.91086	74.69004	14.39753	37.92375	66.16833	193.5768	223.6093	6.380442	12.18624	44.41221	17.62222	78.30598
1.272609	0.9053653	0.5906691	0.3040409	0.3207923	0.9960572	1.621512	0.6525056	0.3293219	0.5823836	1.033028	1.108851	1.649538	0.320843	0.2803086	0.6412974	0.5585567	1.511936
0.1794031	0.4314217	0.4128134	0.2785463	0.5935509	0.4142294	0.2045445	0.7986138	0.2540291	1.425113	0.451687	0.6099992	0.4320582	0.07270153	0.2729843	0.2851189	0.1772316	0.8385829
1.477895	1.468897	1.062984	0.6058159	0.5997694	1.393529	2.441708	2.157028	0.3486962	0.9707054	1.641625	2.389563	4.450382	0.3497144	0.4467696	1.330368	0.7806655	2.758738
0.08576078	0.6293227	0.2468932	0.2430618	0.3342491	0.1142399	2.177832	0.666904	0.7115498	0.9058027	0.0767722	0.1876779	0.3883198	0.5434804	0.4297409	0.348403	0.392692	0.04194729
0.2369093	0.1582006	0.07929274	0.1454797	0.1254095	0.05470075	0.3067051	0.1935329	0.1283484	0.2418221	0.09897013	0.1596279	0.08960803	0.1355371	0.04676592	0.1760915	0.07967381	0.1004267
11.85479	4.185967	7.875451	2.141259	2.189006	2.129943	3.638505	5.945608	0.9218863	2.995771	3.030775	4.632869	9.085615	1.281536	0.7167806	4.969753	2.925622	4.041664
860.6281	550.528	255.8015	144.0903	252.1213	63.5343	243.758	277.9501	165.3637	428.0839	217.2807	624.9936	678.4637	118.6183	59.75972	434.0538	182.443	53.25212
1.496269	2.002855	0.8585078	0.4057123	0.7471584	0.3125757	0.6624662	0.4801945	0.624635	1.02289	0.6973621	1.831431	3.119438	0.4076304	0.8157665	1.042473	0.5415423	0.3797015
21.54683	54.44299	10.39854	12.33419	10.31066	5.844613	18.36807	15.63435	9.522222	22.88858	32.20087	36.36779	46.29717	3.239476	20.95788	26.53798	12.32023	12.39179
0.2025388	0.05084553	0.1216311	0.06694756	0.03627583	0.09229903	0.127027	0.08572116	0.1023776	0.07370996	0.1240547	0.05985496	0.09449979	0.1158736	0.03682478	0.03753185	0.09141729	0.1404052
4.699813	4.186404	1.935251	2.232183	2.096588	3.083449	3.656413	5.456939	1.607883	4.135799	2.468593	3.347625	5.713564	3.701537	2.27985	5.226913	0.9543259	3.162131
22.35637	14.87517	9.426746	7.543968	8.559657	10.53735	16.77422	11.21188	6.334179	14.05049	14.25669	11.8512	21.90392	4.129178	5.589064	16.96363	23.49174	16.94831
3.050318	2.360573	3.576086	3.402618	2.149633	1.79861	3.470951	4.613563	1.760777	2.833138	3.330893	2.523746	4.137872	5.504269	3.143753	2.326712	2.764981	3.826819
1.556049	1.384103	0.4414679	0.3785721	0.2464752	0.6281349	1.104373	0.51533	0.3417599	0.8620112	1.280017	0.3542078	2.594817	0.4090217	0.4462182	0.4071921	0.6788848	1.241553
39.62824	30.34798	17.74992	10.76781	13.91019	6.311957	18.45116	18.52768	9.593763	17.79326	14.86527	24.47693	31.42859	6.071606	3.982424	16.78272	22.33835	8.061768
8.846292	6.034052	9.990892	4.384407	7.500654	9.017635	17.90774	10.48783	4.264195	7.205986	10.56392	11.67434	18.50575	3.726418	5.567537	8.139675	9.310707	15.74493
0.09959712	0.1963565	0.2181915	0.2112797	0.03012707	0.09198519	0.03516529	0.3305315	0.1765896	0.196766	0.1783167	0.02982572	0.04238031	0.1291548	0.1703908	0.1714361	0.5426193	0.03618822
1.027832	0.8397848	0.603758	0.2948619	0.2446962	0.7292625	1.423886	0.4810455	0.513249	0.6157621	0.847689	0.623434	0.3678416	0.2831246	0.07984258	0.7595059	0.8305562	0.7823611
0.1650175	0.1626666	0.1506294	0.0368483	0.03327736	0.04762677	0.1116719	0.05055149	0.1137821	0.05976884	0.05908881	0.04941675	0.04291089	0.1132889	0.02714539	0.06455543	0.03329773	0.01998615
3.180717	1.384382	0.8982352	0.4910727	0.3006665	0.5852297	0.8685956	0.7916849	0.3671572	1.433512	1.290204	1.019482	0.9375463	0.2502095	0.2060211	1.329854	1.754962	2.175968
10.8133	8.629385	7.468571	3.688099	3.091562	9.206234	12.75959	8.778682	2.394464	6.464666	9.508091	8.125065	24.63608	1.427866	2.909224	10.07958	5.600122	10.83287
1.559612	1.121139	0.4655465	0.1485471	0.3074305	1.25439	0.8677473	0.4472036	0.3276367	0.5987168	0.3639252	0.5091036	2.075847	0.2875538	0.1021362	0.5812099	0.0596594	1.201843
5.617224	4.880969	4.124255	1.728102	1.745323	4.842525	8.482935	4.142969	2.147079	2.702	5.870208	3.556295	4.988299	2.836391	1.446314	5.001651	3.81927	6.788531
4.02524	6.896343	3.331836	2.835741	3.189663	2.229859	3.34719	1.514188	1.183492	2.033156	3.267526	2.823764	1.923496	1.519773	1.330976	3.153343	2.664795	3.789934
3.904763	5.016748	1.020241	2.843775	1.359947	5.248207	11.63653	5.316991	1.630256	3.050344	6.009703	4.248225	11.00075	0.9755536	1.499172	5.162862	2.060145	6.866115
9.769156	2.38292	4.932774	2.811373	1.80951	1.702431	1.82832	3.276801	1.096272	3.200403	2.346827	1.629397	1.86361	0.5588987	1.257143	6.828316	2.813398	2.263422
0.9305533	0.4749511	0.6005434	0.4814288	0.372023	1.000734	0.8004615	0.5492511	0.4729034	0.6264678	1.034645	0.3860513	1.895764	0.2102371	0.4797034	0.8486438	0.7415116	1.37291
37.93	12.02886	5.564881	3.747912	4.406648	10.67156	10.95085	10.74639	3.895823	7.989347	7.635909	8.717533	29.35341	3.162276	2.58739	9.247633	8.144668	16.23495
10.2832	10.29341	3.601208	1.663431	3.555976	4.026364	6.544785	5.947109	2.996677	7.869677	3.637401	7.615416	12.1793	14.11462	1.816701	4.683107	2.095299	5.296649
2.160452	2.065734	1.771683	0.6601345	1.02253	3.356758	5.271791	2.583136	0.7077256	1.099094	2.764792	3.58975	5.265331	0.8906094	0.8667357	2.512106	1.449786	5.094287
2.309563	2.897274	1.57535	0.956356	0.9980258	1.626157	3.255825	1.859486	0.812489	1.429014	2.082644	1.304723	4.475814	0.9873549	1.118895	1.538944	0.8962126	2.943244
4.535078	2.625254	2.032196	1.052957	1.753738	0.901355	2.00153	1.507681	1.035567	1.384664	1.430142	2.091152	3.399	0.5896688	0.4679584	2.794268	1.809406	2.275091
0.08009409	0.2917327	0.1129891	0.1402343	0.1101255	0.06934942	0.02570841	0.1784439	0.1398583	0.151043	0.202062	0.1471823	0.1187686	0.06665056	0.05749302	0.11109	0.07346195	0.07936873
0.07601539	0.2486238	0.4956252	0.1363997	0.1067571	0.09402555	0.2204646	0.08316632	0.3690357	0.3647168	0.16526	0.1788588	0.2695489	0.2609329	0.05359088	0.1529357	0.5587639	0.2170134
0.2619485	0.05553048	0	0	0	0.05040177	0	0.02229038	0	0.143753	0.07816457	0	0.1444899	0	0	0.170792	0.1468244	0
2.247562	0.1187487	0.08206378	0.1158183	0.06275664	0.155684	0.1587118	0.4501852	0.04087195	0.2322633	0.4271621	0.0569515	0.8435734	0.1661704	0.1023852	0.1379753	0.1883852	0.2324291
22.82369	48.29341	22.21634	15.21134	18.94789	45.02632	178.6518	29.47272	20.96733	31.14064	27.31342	37.38883	89.37285	32.1096	22.25721	24.43365	63.8188	76.25616
11.01419	10.5473	6.217208	10.88382	7.033536	6.255444	7.814927	5.326148	20.68833	8.195377	7.623395	7.228567	8.595329	3.06131	12.02892	7.706294	13.36892	10.3269
19.97869	15.10051	9.484712	3.929685	5.29342	16.32444	17.71306	14.60366	4.547875	8.309865	14.23819	15.74981	34.98049	3.901575	4.229505	15.73284	5.716727	22.77823
4.192135	6.197495	3.202011	1.396248	2.617043	7.988192	9.40708	7.204158	1.045894	3.014486	2.767043	4.009954	9.789292	1.133926	1.855339	2.676887	1.817181	8.901979
158.2614	171.3523	80.95566	43.84935	52.44662	74.52461	185.262	180.2161	51.24838	88.24127	108.5079	65.17117	234.704	52.45882	30.10078	142.4144	219.2002	124.5544
3.088736	8.917195	4.109869	4.821292	7.230857	1.053431	1.739445	3.372738	2.588844	2.957986	1.338401	13.08228	1.315526	1.887175	0.7145778	5.42489	0.90333	1.743347
1.50619	2.670349	0.968457	0.5830268	0.6447259	1.1729	2.521026	2.227229	1.112722	1.48315	1.081205	2.233969	3.70505	0.3251695	0.6124085	0.7115189	0.4539738	1.802715
2.448125	1.743553	1.607126	2.55547	0.9383449	1.509929	2.205334	1.656587	0.8919083	1.937938	1.861305	1.958346	3.377265	0.4796525	0.9764501	2.302452	2.131601	1.401293
0.5551434	2.159065	3.293809	1.430344	0.4843989	1.294113	0.433478	3.270437	4.13276	1.455306	0.7836658	1.454646	0.7268405	1.441418	2.107411	3.357845	8.918398	1.74556
0.4718301	0.065869	0.03151395	0.01445479	0.08702658	0.03487484	0.03047407	0.1189815	0.1105229	0.1818843	0.08756609	0.1163107	0.2285209	0.06583818	0.005679226	0.0472709	0.1219118	0.01045351
724.4559	528.005	207.7262	95.9893	183.5009	26.08115	153.5164	253.039	191.315	357.9035	153.4072	467.5309	551.6145	49.98043	36.42189	166.4378	142.6241	11.60018
0.0698984	0.2400478	0.1850312	0.1053558	0.0264294	0.2118252	0.05552866	0.433607	0.2272097	0.1104741	0.03754341	0.05233008	0.04957164	0.1119699	0.06898976	0.09023685	0.634694	0.0952399
2.701633	2.543005	2.073	0.5237142	1.147513	0.8566738	2.715029	3.930012	0.6059584	2.243329	1.376741	2.306611	2.734007	1.049965	0.3845446	1.738752	0.5534192	0.5727714
1.515054	2.512379	0.9047364	0.438697	0.6638745	1.422147	2.124714	1.355496	0.7949958	1.007048	1.406962	2.300319	2.851863	0.4212342	0.3214322	1.296173	0.7428519	2.032178
5.685824	6.774306	3.876128	1.566962	1.233684	5.497772	7.807039	5.732494	2.930303	4.099818	5.555213	4.74169	9.425858	1.427429	3.528163	3.500714	5.413785	7.68546
127.4341	114.9922	54.73512	28.47709	25.03685	48.82298	107.0913	58.18364	31.84204	53.07994	56.58305	36.20449	113.3853	23.00574	13.67401	106.8668	69.4102	77.16393
0.0757219	0	0.09215949	0	0	0.02185459	0	0.1546444	0.07458771	0.02493295	0	0	0.01790055	0.08664181	0	0	0.4074505	0.02292771
1.091982	1.47969	0.5493565	0.3896572	0.4081991	0.3169547	0.5914716	0.746013	0.1833453	0.9438367	0.5554155	0.7803621	0.888833	0.298166	0.2388277	1.711176	0.281689	0.5974052
1.548954	0.1149646	0.1682443	0.1365318	0.2171162	0.1319678	0.1752075	0.3583233	0.3980227	0.8753275	0.2189382	0.2202518	0.4399074	0.09328054	0.3323519	0.5033459	0.1466208	0.02897745
17.47253	11.91531	7.478271	4.085539	5.008091	19.04465	15.85719	11.32102	4.251923	9.750159	17.4915	11.68796	34.89082	3.748549	3.52957	10.04395	8.213617	26.14019
3.522957	1.523755	2.644438	2.339973	6.533616	1.462984	2.569255	2.229173	2.769413	1.611617	2.345385	2.294364	2.54698	9.709636	1.134297	2.175672	1.652952	2.41408
1.220264	0.208937	0.1071027	0.1179018	0.3016821	0.04063712	0.09666399	0.1018409	0.1618059	0.3554352	0.0350118	0.07613017	0.3439434	0.1163533	0.05404365	0.2570463	0.2525422	0.04263251
54.88039	49.35635	28.80022	20.03899	16.47941	31.94887	61.10847	29.19757	15.14425	38.60608	35.54477	32.37893	67.523	12.36518	15.26264	71.52108	50.15417	32.10084
16.74676	14.48809	18.91528	7.715101	11.30236	9.983726	9.363657	10.03334	5.760051	20.03555	11.33458	5.968727	5.101153	14.91481	8.757692	22.38643	16.06511	13.9154
0.7125246	0.3157975	0.9928653	0.6732096	0.5674381	0.2593328	1.185519	0.6543418	0.6055804	0.2927471	0.620911	1.550747	1.446644	0.2416975	0.6316912	0.7474235	0.6616212	0.4295791
5.621224	8.807115	12.47096	3.335466	2.252133	5.608486	3.384532	10.94786	14.46594	5.92043	3.632417	4.737913	4.329761	9.796881	15.57892	7.951505	52.3566	9.130156
25.18605	20.66198	9.563765	11.57354	24.18931	1.962861	5.146356	14.42138	3.938894	15.80674	4.439631	41.65341	5.289952	9.808898	2.759156	16.57967	2.84054	2.623874
2.637156	7.867872	1.69828	2.334469	6.223903	1.676151	4.251408	5.064703	1.284555	5.175666	1.924924	7.884306	2.688241	2.745559	1.950725	0.516966	0.321619	2.442884
10.68306	3.130531	1.348625	1.094421	3.631155	0.5289186	2.16936	1.969249	2.686736	5.599178	0.763035	1.4039	3.032568	1.381666	0.5748827	4.601623	4.342855	0.1935663
0.04628676	0.05194764	0.01739745	0.01367974	0.03500313	0.08251222	0.1225704	0.02293742	0.04022952	0.01613736	0.01949903	0.02649936	0.03861926	0.04984641	0.04568505	0.01917269	0.01648215	0.04946503
162.9406	202.3749	103.4394	58.25765	62.6379	127.4155	211.2295	199.0917	62.49945	120.6482	133.3319	105.7012	355.0116	50.66441	39.87609	171.5904	106.7493	251.4131
1.222569	0.6305704	0.5926932	0.176339	0.8670283	0.2988282	1.043004	0.8108671	0.3284337	1.265449	0.2042241	1.983853	2.128191	0.1204774	0.2482635	0.82382	0.548864	0.318815
6.492351	3.779031	2.59149	1.505024	1.536698	3.734895	3.988842	4.5508	1.517483	3.565309	2.521768	2.757062	5.65008	1.418572	1.563975	3.788222	1.65051	6.357224
0.08387808	0.08668393	0.1850312	0.2399771	0.0264294	0.04236504	0.01850955	0.3961347	0.1962266	0.2761853	0.2064888	0.02093203	0.04461448	0.0639828	0.02069693	0.1066435	0.2538776	0.03809596
1.187584	0.3447962	0.2120942	0.313468	0.3221369	0.9053237	0.1937014	0.2965799	0.2670186	0.5100466	0.4160008	0.2705943	0.219712	0.2510912	0.01274072	0.393889	1.719116	0.2110617
0.7852155	2.641424	3.319692	0.08651552	0.01562627	0.1252408	1.887787	0.2611158	1.038057	0.4898803	0.305214	0.06187988	0.03663628	0.224613	0.02039495	0.02425098	3.085473	0.3847863
0.07469371	0.01017921	0.08766139	0.008935209	0.008069305	0.02771722	0.01883751	0.04086015	0.01576606	0.01054046	0.02865646	0.007988591	0.03026998	0	0.02106364	0.01252305	0.08612529	0.01938547
0.1517542	0.005361731	0.02907264	0.001568825	0	0.01297741	0.03968939	0.02726172	0.01660902	0.01295469	0.006708586	0.004207859	0.001328684	0.01500582	0.001849154	0.01099385	0.07749867	0.01872013
0.7709996	0.1161314	0.02469387	0.06229608	0.01022891	0.01756762	0.05372782	0.08287304	0.1698773	0.2137828	0	0.04556968	0.0383712	1.501264	0	0.01587462	0.334349	0
0.5526614	0.2306563	0.1306228	0.04545195	0.1268733	0.1153579	0.2221359	0.4799421	0.1895621	0.2632136	0.1281015	0.3915896	1.392805	0.02823041	0.01948133	0.1216142	0.3385359	0.3092792
1.444165	0.04149605	0.02382373	0.1384143	0.1184217	0.06779437	0.09982964	0.1665684	0.7455448	0.2234372	0.6775529	0.4559224	0.1419066	0.2986318	0.008586696	0.01021016	0.8426255	0.07902584
0.006505081	0.02482223	0.02968948	0.01089435	0.01475788	0.01126484	0.00574196	0.02989152	0	0.006425784	0.005823284	0.009740178	0.02768025	0.1339773	0.01926156	0.007634432	0.01312615	0.01772697
0.2705468	0.2111642	0.1546603	0.08466963	0.2479925	0.05205618	0.09166389	0.144411	0.137285	0.1592695	0.07339116	0.153445	0.08721393	0.08129927	0.03236726	0.1186678	0.01930012	0.02234139
3.057454	2.239325	0.595008	1.816202	3.303864	0.8163613	1.863117	1.286665	0.739538	1.027169	1.184413	0.9266347	3.249748	0.4661536	0.8731441	3.269506	1.518868	4.204696
4.03135	1.106203	0.8098515	0.6582044	0.9004555	0.3234479	0.5564334	0.8761572	1.069401	2.65993	0.5225131	0.7749194	1.407431	0.6322445	0.3533425	1.735395	1.107119	0.3393301
11.85457	13.50296	3.714657	3.733622	3.773204	5.852169	11.27602	5.203389	10.61388	3.775185	5.670343	7.9213	14.75654	1.133718	3.213263	5.52353	14.03099	5.400212
3.173563	8.637916	5.627608	2.535553	4.287934	2.590269	8.827846	3.088263	2.054145	5.212811	2.280572	1.858229	4.465219	3.348056	1.048892	3.442624	2.269205	3.484429
0.3428039	0.2304001	0.1635689	0.1565752	0.3240454	0.09444163	0.07564725	0.250603	0.1841832	0.4309774	0.1115908	0.1283216	0.07182944	0.03565817	0.03075887	0.1645847	0.1572091	0.1344643
14.40137	13.31128	5.547224	3.647546	4.934119	4.171123	9.722321	8.127967	4.404328	7.47072	6.873512	10.5019	15.98931	4.514215	1.808081	5.24215	7.41153	7.812975
3.520545	2.635739	6.180839	3.949335	3.113527	0.7395572	1.791973	3.737153	0.7681864	2.247497	2.369208	3.130575	2.990136	1.090629	2.260317	7.508043	1.788455	4.841888
189.1455	185.5942	102.7741	61.04876	57.95311	89.9361	221.8231	211.2029	59.48966	114.9029	121.0758	88.89266	339.235	40.14359	36.61432	190.35	168.1453	173.0684
0.04064291	0.09908277	0.06595415	0.1985273	0.09220536	0.3597266	0.1793751	0.1296933	0.09007683	0.1427466	0.08287268	0.1943991	0.1889559	0.03875338	0.08022915	0.1006978	0.01366842	0.2953488
2.40204	0.6609919	0.758978	0.2514253	1.013036	0.1799831	0.4281279	0.7606052	0.8872729	1.870829	0.2481095	0.4495773	0.6388186	0.3699823	0.2735555	1.138466	1.654474	0.1468606

TCGA-28-2514-01	TCGA-TQ-A7RJ-01	TCGA-06-0747-01	TCGA-27-2521-01	TCGA-14-1829-01	TCGA-HT-A5RA-01	TCGA-12-0821-01	TCGA-DB-A75O-01	TCGA-S9-A7J0-01	TCGA-DH-A669-02	TCGA-27-1834-01	TCGA-HT-7478-01	TCGA-28-5204-01	TCGA-DU-A76O-01	TCGA-DB-A64V-01	TCGA-CS-4941-01	TCGA-QH-A6X8-01	TCGA-S9-A6WP-01
0.1851182	0.09460236	0.2508521	0.3267495	3.234864	0.3489744	1.060759	0.05912457	0.06107569	0.01991059	0.2540583	0.125524	0.3982341	0.005746836	0.5212078	0.4000371	0.1177473	0.08593613
0.721939	0.1893234	0.3238832	0.6065243	1.667274	0.0372473	0.2378542	0.3067642	0.03259414	0.1138462	0.1857298	1.703088	0.4601915	0.4983716	0.1075329	0.7412742	0	0.1592408
1.722476	0.5991937	1.878001	2.714324	6.131507	2.962187	4.813893	0.431736	0.4165096	0.7937748	4.836257	1.71836	3.339029	1.360188	1.021	1.69129	0.4412811	0.7889444
0.8092991	0.01132704	0.5210428	2.739727	3.910257	0.3654692	0.8158874	0.03670683	0.07410291	0.04540873	0.1611246	1.348399	0.4805729	0.09633219	0.05575782	0.170303	0.06946968	0.03810892
31.55807	10.09508	126.7558	6.59207	55.3963	8.702977	17.18105	20.40549	10.70144	10.95619	25.57961	24.45154	28.38416	20.16707	19.42172	22.94424	15.46607	19.0206
2.75563	3.091238	10.10491	3.552092	8.423991	4.285888	2.946892	3.011237	2.828005	1.743541	7.288679	6.640377	4.777792	2.453367	4.135833	4.987942	1.979524	3.401739
4.358	1.744895	5.325201	3.836666	2.813107	7.755783	3.788949	2.954438	2.102821	4.520887	5.959509	4.587825	7.668691	4.455825	3.517703	6.341048	2.65208	2.505858
3.82651	2.182367	2.988853	1.453519	3.404965	6.370286	6.82006	2.308101	1.414841	2.399526	8.277116	4.016718	7.724018	3.300454	3.677166	7.619537	1.689962	2.953855
0.5144247	0.04643444	0.04236654	0.4909056	0.7010131	0.1534758	0.1050072	0.09028628	0.02877914	0.122859	0.163991	0.4594789	0.2216335	0.1241137	0.2531912	0.4887019	0.02010256	0.1687229
2.697146	4.441137	2.358164	1.836866	5.656049	2.949934	2.737627	3.943275	1.744649	1.651313	6.836242	9.422078	3.399241	9.027346	6.015814	6.999275	2.080046	3.409439
0.1363915	0.004356323	0.02981014	0.08373656	0.05261335	0.5296621	0.2845967	0.006050254	0.01349981	0.03667433	0.2628284	0.1449955	0.121292	0.06351234	0.02969193	0.1228081	0.004714886	0.05276338
61.45058	75.88967	82.38826	26.29481	194.7682	57.1224	56.60371	97.4123	26.07374	47.39926	193.063	185.2728	69.70421	143.526	37.86376	182.7741	26.78603	30.80575
0.5599995	0.9984765	0.5678179	0.5159644	2.886743	0.5715574	0.5808155	1.645078	0.6075329	0.3915038	1.976491	2.55924	0.9357448	1.887775	1.684269	1.503788	0.4973995	0.751009
0.3493298	0.4351441	0.1394771	0.123004	0.6011123	0.5002298	0.3001425	0.3673484	0.5141219	1.173221	0.8412134	0.6728683	0.1470615	0.8292963	0.2713864	0.8071131	0.1108142	0.2755778
1.187605	2.007788	1.534413	0.6083268	0.7955589	1.563823	0.7989023	2.661754	0.9275123	0.8001768	4.40578	4.1896	1.975044	3.333544	1.836001	4.38202	1.570838	1.595655
0.2538876	0.09537885	0.2805352	0.2412312	0.6517527	0.4325765	0.6180606	0.1394383	0.08815233	0.2596038	1.684232	1.192161	0.9317926	0.1829686	0.05132254	1.122694	0.2879556	0.08613481
0.1664315	0.06009163	0.06168069	0.0938496	0.1814387	0.1205881	0.09814402	0.04172896	0.03103633	0.07227	0.2829648	0.324338	0.1274755	0.07300806	0.1023935	0.1806965	0.1886093	0.1031084
2.867998	4.238005	2.973727	1.631677	10.26977	6.403803	2.987317	3.55642	1.503459	1.310496	6.614302	15.58202	4.623111	7.513016	6.719614	5.660963	1.255165	3.020586
424.6971	215.8208	290.1049	213.9325	603.1665	292.3504	203.3288	373.8248	118.478	201.3159	986.6007	868.0087	480.0525	528.3252	602.1095	747.5407	56.05987	311.1077
0.9140404	0.4988057	1.071812	0.6556988	7.754116	1.331216	1.176139	0.6927634	0.4891139	0.9041901	3.951933	3.020652	2.659733	1.185695	0.8663671	2.560999	0.2699308	0.4268443
23.36893	8.129941	26.30987	12.13727	75.83294	26.88994	25.99074	27.49578	9.703918	22.22798	106.4612	59.92	46.6265	20.09238	17.48539	53.65865	12.40097	11.81652
0.04395559	0.1216744	0.04625627	0.0389801	0.09184489	0.03830094	0.07643206	0.03943024	0.06284279	0.0634111	0.0596824	0.1021571	0.01613213	0.08376905	0.1428258	0.08384676	0.08779281	0.09824724
3.795724	1.985579	1.937725	1.981513	3.522175	4.602458	2.888585	1.266016	1.608199	1.212987	7.181736	10.08976	4.358698	3.560987	5.062193	4.849047	1.851078	3.709854
10.85841	15.34193	9.723227	11.87415	39.92062	13.57541	13.48185	20.74629	7.629631	18.25794	22.14619	18.32112	16.88092	23.64408	23.02529	34.27339	8.932075	13.84362
4.721891	2.989452	1.752166	2.272125	2.009555	4.762965	3.048508	3.115838	1.56119	2.401316	3.770624	6.341339	3.919428	4.686587	3.383329	3.705596	1.764823	2.549896
0.9826717	0.920052	0.5748411	0.9419236	1.449378	0.6988563	0.593025	1.466702	0.6198151	1.116135	1.047787	2.432562	1.166809	0.9914516	3.226339	0.8796019	1.047734	2.139891
14.16755	14.26149	13.58739	10.43029	46.76106	14.39824	14.48129	16.72531	8.261984	15.31325	46.12974	48.47339	23.27862	35.51591	35.71322	32.40343	4.132845	19.3489
9.396931	12.49089	5.474575	4.640531	4.658628	7.756576	5.152771	21.85064	4.075237	7.537266	10.96583	32.8697	10.35781	15.41831	9.766843	13.16911	8.309626	10.89456
0.6996812	0.06063032	0.3284552	0.2245874	1.128902	0.1133193	0.8696323	0.05613738	0.05741002	0.0546884	0.05947946	1.354433	0.1473751	0.08593962	0.03443713	0.2991127	0.07108911	0.05099644
0.5842785	0.5829713	0.627708	0.6177102	0.4701167	0.6283191	0.2820457	0.7493093	0.2393799	0.2612863	2.051401	0.9608951	0.6241047	1.170199	0.9172619	1.391651	0.462439	1.180471
0.03528204	0.05859896	0.07637911	0.09554116	0.4044154	0.09387654	0.05258578	0	0.07350166	0.02013566	0.1970971	0.08283563	0.1609358	0.005085324	0.09985005	0.11013	0.02718092	0.0633701
0.7013142	0.8319952	0.569331	0.5179383	2.523517	0.8124771	0.8362137	1.890837	0.8203587	1.146153	2.882679	2.44942	1.183322	0.8637992	4.433482	2.23174	0.7694972	1.79402
7.124757	10.33374	4.528007	4.212187	20.9782	4.914822	6.197132	9.136316	4.536069	4.1909	19.80307	23.23002	11.74122	12.58625	13.09574	18.61758	6.868691	7.563454
0.5350669	1.046165	0.3784705	0.4392135	2.411524	0.4979551	0.4239797	0.6717988	0.4183176	0.2164618	1.911911	2.292018	0.8426718	1.059195	0.8370005	1.55389	0.4748243	0.7153015
3.043545	6.597439	2.628219	5.001153	12.72361	3.049323	3.18703	7.08505	2.821959	3.430832	7.452157	9.395416	4.484471	10.20569	7.473387	5.370045	2.856186	4.680033
3.335359	3.140139	4.474434	1.603525	1.939343	5.350618	2.990309	2.319882	1.067937	2.01817	3.048366	3.321001	4.004782	4.490004	2.532908	4.310147	1.240029	2.367333
5.219299	5.659116	5.104899	2.089378	18.93063	3.044066	5.917953	2.173135	2.179459	3.088277	9.811743	11.90783	4.92979	9.525043	5.809266	6.890636	3.316184	4.094072
3.121752	2.197202	1.405876	1.786139	2.095371	3.526699	2.057021	4.260437	1.764193	0.8714006	1.668547	3.36475	2.414108	2.172168	6.744963	1.887847	2.815978	4.1742
0.4399198	0.8704664	0.4063634	0.5201649	1.252848	0.6548494	0.6232953	0.989704	0.670878	0.4339391	1.048625	1.910945	0.5262234	1.287714	0.9068493	0.9663074	0.6052955	0.9787335
9.329092	10.25617	6.018293	5.368844	40.90627	12.19343	8.062366	9.003979	4.548393	4.463373	22.84599	17.87838	12.3372	14.39108	21.20227	19.50052	4.083653	11.29608
5.320754	3.995719	2.579941	2.244593	8.832969	14.05334	4.837369	3.589513	2.009127	2.533061	14.72675	13.87842	11.31018	5.342608	4.578046	12.54562	2.067914	4.09433
1.828788	3.728389	1.619474	0.9806709	1.467356	1.783752	1.464383	4.519018	1.596212	1.753416	5.508776	8.565839	2.567277	5.618415	3.79973	4.588138	2.301171	2.479393
2.108545	1.699512	1.644603	1.608639	2.624036	2.044156	1.876353	3.075616	0.9708663	1.130361	2.292472	6.068958	2.099655	2.307273	2.83739	3.181938	1.553732	2.625021
1.02945	1.30979	1.556421	1.319132	2.261541	1.509087	1.359787	3.80158	0.8020598	1.160204	4.708838	3.922768	2.048539	2.830078	4.267641	4.517846	2.962523	1.1241
0.2068313	0.08310978	0.1706151	0.1065016	0.1087402	0.3400994	0.07657025	0.08464612	0.09157296	0.1066166	0.3016693	0.4535602	0.2938418	0.1346318	0.08182224	0.2707385	0.06596365	0.08947715
0.15921	0.2479008	0.2638805	0.1092004	1.659182	0.06828043	0.2595393	0.2295303	0.1877871	0.1689467	0.5228697	0.5872409	0.2410305	0.2509883	0.1032563	0.4503709	0.2951356	0.05838294
0	0.02214759	0.05051836	0.0532147	0.06687171	0.02614377	0.05564972	0	0.06863319	0.07990829	0.1303632	0.09961602	0.02936427	0.02690812	0.07547705	0.2081193	0	0.04470826
0.1774325	0.7840932	0.08402378	0.1390846	1.604792	0.1739326	0.4561808	0.07308613	0.22287	0.05063088	0.2168243	0.3171676	0.2511752	0.1790178	1.093956	0.4203259	0.1309967	0.6267506
31.9551	32.42985	21.8437	33.8272	88.47127	35.68283	40.74286	51.41928	15.46932	52.18706	52.1977	100.1401	34.18385	82.80213	49.44075	43.05626	26.91995	35.37396
8.476165	7.905933	11.4631	5.354632	22.04811	13.03205	13.66832	5.449916	7.08521	14.43605	9.431789	9.110861	15.11424	8.481672	7.850088	8.44907	7.277679	7.389775
12.40534	18.36008	10.89625	7.849289	11.4502	14.25271	8.948643	19.18375	10.1546	8.222172	28.1285	35.336	11.86045	22.3474	18.07838	27.33789	11.96961	12.71099
3.812507	4.698193	3.453858	1.970093	5.529661	4.295427	3.233226	5.294856	1.891773	1.864254	14.73417	14.38029	6.49887	4.966366	4.867897	10.89935	5.259789	5.279635
62.35834	107.2571	65.72808	64.41978	404.2579	74.91632	100.6719	195.935	58.85425	121.8081	184.5122	210.5165	170.1638	262.1219	108.7329	157.5043	63.23708	103.6402
2.335658	1.639708	12.55516	1.245377	7.436163	13.16031	4.894101	1.833611	1.34693	0.6860894	5.343825	4.571696	7.714886	1.255494	2.251486	5.792631	0.7479723	1.40823
1.905302	1.153335	1.340034	2.338161	3.98837	1.629469	2.073851	0.9360536	0.9121328	0.5418258	4.842223	3.757696	1.87798	1.637699	0.8679778	2.729772	0.7138513	1.069508
1.014078	1.594938	2.342893	1.594198	16.08445	1.506175	1.346541	0.903769	1.83204	1.135559	3.154362	2.915413	1.894718	3.363956	3.319223	1.210991	0.9804901	3.180988
6.652082	0.7473822	2.668334	2.459408	41.52673	5.389306	13.00262	2.617559	0.5370581	1.152872	2.677758	11.80943	2.972739	1.57918	0.8120911	8.015488	0.8088981	4.821293
0.05272525	0.01751398	0.02996187	0.02630086	1.500502	0.6202228	0.1870296	0	0.01356852	0.02106342	0.5927631	0.2875282	0.2147926	0.01063927	0.2337706	0.4237873	0	0.08396722
275.645	113.9675	203.2045	173.7346	2338.289	312.0218	178.0908	179.423	87.0701	139.4296	897.5345	944.4473	469.2074	373.9856	413.0076	607.9484	23.55015	224.7764
0.6276827	0.1489287	1.025179	0.1214085	0.6102669	0.1004574	0.1804225	0.2068387	0.06593074	0.04477777	0.6026699	2.483249	0.2538724	0.02584861	0.07250513	0.1699359	0.06332338	0.1342121
1.413934	2.254741	1.570494	1.021646	6.701886	2.187279	0.882161	1.018545	1.261245	0.703727	4.563567	5.512869	2.001578	6.203908	2.853789	2.126095	0.5741943	1.07095
1.284477	1.239068	1.044501	1.346188	1.935665	1.220985	1.001695	2.065047	1.157489	1.529056	3.678356	2.849557	1.757095	2.186103	1.725773	1.538958	1.212779	1.587748
6.125999	5.872245	3.206355	4.430213	11.36403	4.755284	4.296548	5.197632	2.96063	4.588688	6.563709	11.32036	5.841282	7.533692	6.387281	7.393689	3.286461	5.662903
42.59804	61.82958	38.24853	54.99009	256.0066	41.71525	49.92185	83.46895	27.25229	49.86965	111.1378	109.9102	86.51609	131.5195	108.1697	122.9075	41.0172	63.53389
0	0	0.04381028	0	0.1159843	0.09068907	0	0	0	0.04619845	0.05652646	0.0518331	0	0.04667025	0	0.03609683	0.0207876	0.0969292
0.8812138	0.6326458	0.5922993	0.487786	0.6414816	0.7690889	0.5279008	1.521239	0.3121204	0.6075523	2.577497	1.248636	1.445974	1.095585	1.201354	1.650382	0.5109836	0.3526297
0.3150473	0.08361264	0.05537013	0.1166508	0.6107837	0.2483399	0.2676972	0.03371369	0.1058718	0.1005579	0.4842153	1.715388	0.5292547	0.1081387	0.266561	1.056896	0.02627265	0.2776782
8.748182	16.92625	6.776427	9.057599	8.043953	9.202902	8.817889	18.65378	7.069593	8.432583	27.25859	28.33189	15.51429	23.3896	15.26955	19.55508	9.047774	12.89047
9.173598	2.717213	4.698908	2.135596	3.053024	2.430196	3.286214	3.564131	1.381888	2.810889	2.313282	3.586862	2.991817	4.287852	3.199025	2.783244	1.346487	2.248683
0.08601167	0.04761813	0.07467372	0.01430169	0.6200366	0.1475514	0.1121709	0.05786739	0.07378193	0.05726858	0.350357	0.722851	0.2919959	0.08678015	0.3651264	0.5761099	0.06442189	0.3484509
34.95452	33.14764	22.54744	28.15069	101.6207	31.62311	45.81975	28.46988	15.97302	21.26837	74.47639	74.93509	50.26555	33.12445	56.70362	57.48548	23.9112	34.7745
8.231161	7.863279	16.48743	10.24979	10.24368	12.19198	9.427507	10.1665	4.782007	8.683041	13.08351	11.89947	12.02089	9.786962	16.82073	10.37296	4.820503	9.749967
0.886904	0.5014075	1.039731	0.2392201	0.8004287	0.7532996	0.8801037	1.809247	0.2552514	0.5453187	2.400611	2.768878	0.4357705	1.661408	0.741277	1.341366	0.3816919	0.2276164
25.03784	2.86107	12.82597	16.77757	132.1452	24.81148	34.52115	6.806231	2.574993	5.110253	10.12936	48.95162	9.577232	4.370867	8.302214	22.7593	2.023495	9.663959
8.034974	6.604239	20.40386	3.394253	64.95404	15.40662	15.82847	5.128877	4.331984	2.451857	26.38575	33.57737	24.54821	2.950149	16.93244	21.69351	4.823618	8.853731
11.03082	1.706823	3.445568	3.322157	2.610054	8.309062	5.058884	3.83447	1.152615	2.418724	8.495434	7.97455	4.040622	1.666459	2.730493	6.614486	2.56332	2.158989
1.21062	0.5729371	0.7643909	0.9090844	2.953899	6.09959	1.996434	0.2852583	0.3573283	2.067151	5.933465	8.440797	3.640471	0.9456282	6.041758	8.167195	0.4445962	1.789395
0.0573829	0.01864676	0.05907363	0.01991251	0.01876714	0.09782786	0.04164735	0.005754988	0.03210248	0.0423598	0.1280497	0.09505254	0.0439515	0.0352408	0.04001078	0.05451358	0.01793916	0.05855315
104.7661	150.8875	120.2397	89.2676	389.7461	97.84348	121.4088	352.6152	84.74764	110.8915	231.5118	295.278	221.8542	380.1617	208.2047	219.5733	107.9702	159.2272
0.4931304	0.249269	0.812256	0.2192498	0.9273533	1.050877	0.5760019	0.6862095	0.2712805	0.4818	4.847082	2.995122	1.699673	1.032929	0.8697127	2.421835	0.5010311	0.6065202
3.979489	4.476991	1.9418	2.112504	4.129465	3.986656	5.996315	4.775799	2.22003	2.007368	8.978431	7.442051	2.708854	4.489796	9.511965	5.581349	3.649218	5.442773
0.6212778	0.06914546	0.2001829	0.2747666	1.750502	0.06906446	0.447715	0.08125808	0.1373557	0.0255873	0.09392258	0.3827745	0.1057802	0.08400797	0.1812628	0.08996604	0.09786341	0.2147393
0.3430215	0.4518428	0.1904457	0.306816	0.7414561	0.3594454	0.3578772	0.2046324	0.1116114	0.8623757	0.4769929	0.4152982	0.4948868	0.4892944	0.4686472	0.8399564	0.5847138	0.6245992
0.04733607	0.03930958	0.6545518	0.07556026	0.8189613	1.791131	0.6321423	0.05459488	0.0243633	0.05673143	0.09255222	0.1555907	0.1250842	0.1719324	0.3929599	0.1551435	0.07658117	0.1587047
0.01955523	0.01623937	0.03704178	0.009754706	0.159356	0.05750849	0.0306032	0	0.08387364	0.009765246	0.02389668	0.1753004	0	0	0	0.01526001	0	0
0.04120158	0.009979456	0.008129645	0.02569065	0.08178588	0.00168287	0.04119487	0.007919948	0.002945271	0.04457858	0.01678291	0.0628402	0.005670519	0.02424899	0.0696377	0.03617082	0.07560596	0.08489696
0.117747	0.03087832	0.05869419	0.3709614	2.113281	0.09112452	0.387936	0.007147529	0.02126419	0.07427244	0.1893263	0.06481314	0.1501128	0.01875774	0.04092301	0.2031129	0.01670993	0.01558314
0.20347	0.2553308	0.1798607	0.1443508	2.6586	0.4299397	0.2217168	0.4328385	0.1706606	0.06773756	0.8840619	1.354476	0.2290049	0.6204259	0.2516595	0.4939785	0.1503651	0.284241
0.3108996	0.112541	0.03020053	0.3976554	1.519117	0.05470178	0.2328768	0.07355383	0.136766	0.03184681	0.1656072	0.4704587	0.06144019	0.1528172	0.07520192	0.136858	0.02865978	0.1469995
0.07748951	0.01485002	0.02822725	0.0178403	0.08220238	0.02337262	0.01243776	0.02062437	0.01022639	0	0.02185222	0.1647556	0.05250354	0.01804197	0.005623063	0.05581781	0.01607231	0.004996169
0.1051728	0.02703359	0.08537989	0.03247721	0.853916	0.5768592	0.1828794	0.03176922	0.04081317	0.04501704	0.6120104	0.1010152	0.1764546	0.06568868	0.210241	0.2188589	0.07652271	0.04617579
2.591839	1.204611	2.091082	1.798332	3.173604	3.188963	2.151401	3.440347	0.6984071	0.8202436	3.792884	3.962673	1.143043	1.517344	2.284026	1.922675	1.032144	1.221805
0.7487845	0.4500776	0.1688521	0.483791	1.072855	1.188405	1.339223	0.4770406	0.5811441	1.089706	3.041359	2.647664	1.460426	0.8130347	2.590011	2.938282	0.6345416	1.141664
6.420268	3.715979	8.107573	8.993196	107.4021	5.657907	8.017686	3.291019	2.308653	14.18445	26.74647	13.92819	10.46055	8.080681	12.4802	11.6649	2.564649	5.734663
4.855554	2.713999	19.82254	2.827989	7.324933	1.183406	3.200943	2.753913	1.498999	1.172385	5.216295	11.64906	2.768521	2.82631	3.30324	3.903559	1.726463	1.956649
0.1070856	0.183784	0.1352287	0.1210794	0.2506052	0.2169449	0.1564127	0.1646757	0.1224792	0.1140802	0.401303	0.3466507	0.1650662	0.09363674	0.2222425	0.3899689	0.1411627	0.1136923
6.519906	6.439654	5.970815	4.015829	52.12369	5.661813	5.227428	7.74891	4.10941	5.51084	20.35766	25.41417	13.5239	17.78065	14.65844	16.11809	4.362708	9.497937
1.882934	2.27578	3.863187	1.371548	4.670059	2.795376	2.539625	4.066005	1.436171	1.821644	3.335012	7.668573	2.687623	3.126589	2.405453	3.556551	2.33464	1.192236
107.8533	124.0724	110.008	70.46228	485.3137	97.97691	128.5955	319.9346	66.6703	130.6158	202.8722	338.6816	223.9771	301.842	196.4939	192.887	74.48468	133.7512
0.1634505	0.1683804	0.2488639	0.07843756	0.2256683	0.06490184	0.09281974	0.05249786	0.177481	0.01859739	0.1441148	0.5162315	0.1321253	0.1669984	0.128818	0.09202928	0.06694513	0.1144565
1.608449	0.2636278	0.3006657	0.8234545	2.467566	0.6431366	1.280662	0.3417287	0.3267825	0.634111	1.241394	3.320105	1.118495	0.2135289	1.137999	1.750619	0.2853266	1.064345

TCGA-DH-A7UV-01	TCGA-S9-A7R4-01	TCGA-DU-7013-01	TCGA-FG-6688-01	TCGA-DU-8161-01	TCGA-HT-7858-01	TCGA-QH-A6CV-01	TCGA-TM-A7C3-01	TCGA-HT-8114-01	TCGA-S9-A6TU-01	TCGA-06-0211-01	TCGA-HT-A618-01	TCGA-QH-A6CX-01	TCGA-TM-A84L-01	TCGA-RY-A83Y-01	TCGA-HT-8015-01	TCGA-DH-A7UT-01	TCGA-VM-A8C8-01
0.02456119	0.156818	0.2066086	0.7212852	0.4774663	2.512853	0.2086046	0.2808769	0.23779	0.09462526	0.1559274	0.1726038	0.1484605	0.08455833	0.0320191	0.06831933	0.006949618	0.08606643
0.163844	0.1207049	0.1120533	0.4318089	0.2132289	1.314074	0.03662026	0.07570454	0.3440085	0.1147693	1.128318	0.4040048	0.04042275	0.208917	0.09493092	0.189895	0.2781591	0.0459309
0.88427	1.232235	1.395094	2.212902	1.826668	1.214728	1.680989	1.183582	1.338438	0.8848747	3.652426	1.897776	1.849764	0.7844983	0.5286093	0.7130693	0.8704229	1.042712
0.01960527	0.1877632	0.1850315	0.4354996	0.1530874	0.5039731	0.3505529	0.2219372	0.6151643	0.09613151	0.5484879	0.3045575	0.1644549	0.234987	0.06815553	0.06362293	0.09985199	0.07694407
13.59699	14.60136	12.69962	27.11833	17.14413	17.70245	11.92496	13.06318	18.73455	18.44262	21.31862	16.47884	54.97331	10.67462	9.704179	25.69108	22.17792	8.172306
2.419584	3.972718	4.061679	3.826014	8.338824	4.968185	6.228426	1.450689	4.040186	2.699849	4.407439	5.828755	2.390722	3.060567	2.434815	2.444283	2.096345	3.408112
2.181204	2.376024	2.849586	2.421544	3.598351	3.243436	2.650076	1.531127	1.745105	2.722773	4.61581	3.737291	3.435787	2.824035	4.625779	2.171498	3.315333	1.787355
3.06295	2.526621	4.734281	5.395561	4.674179	2.808176	6.464396	5.24144	3.312838	2.34702	3.714151	5.927495	4.509485	2.472714	1.483468	3.022407	2.327653	2.046927
0.03857782	0.1421026	0.04617103	0.1452448	0.2274946	0.3272549	0.03233402	0.06684367	0.2193706	0.1125956	0.2490634	0.2259213	0.0237943	0.1106785	0.2933689	0.1453128	0.1228009	0.1216647
4.435469	3.026451	4.104978	6.82548	9.275085	4.504503	7.365849	2.043103	5.600605	4.144567	3.380917	8.408836	2.817462	3.966198	1.547547	3.261385	2.594543	3.87644
0	0.02221931	0.006188015	0.01277473	0.05151705	0.1255989	0.01516735	0.1724538	0.02902393	0.01584501	0.08275559	0.06135446	0.005580752	0.06345464	0.06553067	0.05767706	0.01280085	0.04438844
35.16937	80.35621	94.3501	138.2212	194.2867	40.19692	100.7573	46.15338	81.62223	86.91752	137.1888	198.2152	116.8012	60.90308	13.59554	83.67927	50.62402	45.31604
1.366462	1.426654	0.6897229	1.668553	1.555116	1.413814	1.879468	0.4266062	1.562429	1.631779	0.8620302	2.140029	0.9216657	1.735126	0.5226046	0.816558	0.8118677	1.17866
0.3367084	0.3989192	0.2403754	0.236304	0.8168146	0.6954713	0.1122248	0.1637652	0.4926645	0.168962	0.3146337	1.201681	0.0692258	1.239048	0.1240693	0.2293543	0.7897551	0.1987169
1.794647	2.012096	2.124362	2.305029	5.373918	2.703547	2.835827	0.9005595	2.321603	2.509227	1.621843	6.049812	2.323201	2.225975	1.031844	1.236657	2.019944	1.114188
0.06777185	0.4864773	0.6025409	0.05397611	0.3392277	0.3698704	0.1048671	0.04215366	0.3709384	0.4808136	0.1851147	0.3342221	0.1543414	0.3988415	0.1132698	1.450107	0.04425259	0.04384313
0.08112689	0.1915599	0.1493769	0.1703423	0.177658	0.2374202	0.1185581	0.08650357	0.2365761	0.1602831	0.120869	0.17696	0.100076	0.2864621	0.1265513	0.09402594	0.05297295	0.0262414
2.663339	7.438506	5.378706	10.07688	11.79475	5.713975	9.186558	3.30526	9.164386	3.968083	5.448953	12.96007	2.567518	2.772468	1.691827	3.147699	2.435747	4.877402
79.06563	374.2737	359.3561	803.207	782.4517	464.8993	802.817	277.8916	418.4873	341.9398	476.6935	908.1619	308.4949	197.9619	239.5235	347.5914	205.9552	105.1146
0.454198	0.6590811	1.252776	1.469793	2.12808	0.7603623	1.430458	0.7544672	1.808323	0.5258777	1.967024	4.382639	0.5927001	0.4499154	0.271861	0.4481051	0.6638214	0.3840847
10.35058	16.20781	24.60661	26.88868	42.2482	24.63966	29.34549	10.65047	30.72612	16.02255	45.21498	67.89988	19.90765	10.84067	8.809741	13.53167	9.612425	4.662711
0.08423941	0.1396343	0.1065813	0.06739648	0.1096303	0.1602439	0.08472637	0.04865379	0.1228263	0.07375989	0.04985387	0.12463	0.1143071	0.1611201	0.05490917	0.04881668	0.065548	0.1180755
2.458031	3.691319	3.80921	3.923655	10.43879	4.893464	4.284065	1.90553	3.312688	2.478471	3.729074	4.827411	2.691081	2.420138	2.874832	1.406775	2.347073	2.361485
13.72299	12.63416	9.734447	15.78656	20.51861	12.87833	26.78982	10.78791	13.65388	16.93019	27.03516	22.6979	13.49152	18.5813	13.38851	8.925279	15.609	13.6077
3.591259	4.342318	2.784989	4.445566	4.314845	2.742116	3.517719	2.075851	4.961657	4.306982	2.968702	5.599353	2.366027	3.315974	3.766908	2.229665	3.48767	1.570269
0.7809979	0.856928	1.238718	1.212151	1.777351	2.041806	1.940569	0.1823505	0.7995433	1.008786	1.135394	1.905289	0.8814256	0.7839654	1.504351	0.4044401	0.3761434	0.6288688
11.71584	18.95	19.79529	33.49228	31.79761	31.11203	46.89173	15.14279	28.67582	17.11098	22.78396	46.99178	18.47655	16.75814	16.84421	15.55062	18.1956	11.08863
13.05745	13.42038	8.359756	13.72845	14.49946	12.2444	11.78655	6.455993	15.56734	17.04899	10.05865	24.6117	7.978609	13.83341	7.697345	10.00372	11.2165	8.851003
0.01049412	0.07731083	0.4090862	0.3506529	0.08962533	0.6096619	0.1055478	0.01212209	0.1866732	0.09188636	0.5645949	0.5110567	0.02589055	0.1003577	0.03800171	0.5594829	0.03711654	0.007354623
0.9814526	0.7295061	0.4989125	0.7114229	1.406961	1.93714	0.9203091	0.3518399	0.8092658	0.7946046	0.8335217	1.330567	0.5751958	1.198704	0.5501325	0.4488993	0.303239	0.7326355
0.03477437	0.1547784	0.05053731	0.04909684	0.2863846	0.08939102	0.03886155	0.1205069	0.1546448	0.03552312	0.07951318	0.06966884	0.05362096	0.03325552	0.03148157	0.04534138	0.03074828	0.1096696
0.8561741	0.5593796	1.584716	1.74482	2.344753	2.754131	1.694157	0.4355201	0.9437106	0.9720423	0.9804252	2.575984	2.548335	1.01158	1.422207	1.310934	1.022362	0.6165495
8.268472	8.853893	9.567121	15.11592	14.55787	10.14456	17.10927	2.33496	9.42615	9.205134	9.521239	16.60882	9.010808	11.92571	4.679565	6.345667	8.535088	8.524915
0.5081758	0.9036668	1.354217	1.220536	2.014448	1.519532	1.005255	0.1551865	0.9538692	0.8387713	0.8107844	1.238651	0.518791	1.437458	0.1945986	0.4874276	0.3470767	0.8596633
6.870756	5.13045	3.022622	6.221829	5.510069	9.604935	9.037105	1.783511	6.855006	7.210215	3.966512	9.172861	3.714021	8.687052	3.452539	2.96383	4.86022	6.911778
3.116818	2.892359	2.189135	3.133545	4.099283	2.0885	4.257682	3.854857	2.899611	3.634098	2.724394	3.45251	3.543368	3.085404	0.9516841	2.377304	1.99506	2.100138
7.037686	7.494118	2.664042	5.398475	6.3046	7.026848	5.669275	2.622614	2.30489	5.499532	7.389932	11.81095	5.049661	7.854722	2.845806	3.779759	5.494957	6.102804
2.228052	3.395627	3.983187	7.542362	2.440194	4.491513	9.369979	2.867184	2.918076	2.253208	2.607733	2.89153	2.730386	2.342565	4.805598	2.061317	2.260423	1.157422
0.6978923	0.8569026	0.8969207	0.8332339	1.062923	0.6742559	1.198667	0.5843287	1.608991	1.137389	0.987823	1.524516	0.6297873	0.9376592	0.4884856	0.711142	0.9343352	0.7156044
6.439613	11.34759	13.67896	20.52665	24.57295	20.00889	15.03904	4.257252	12.66041	8.713216	12.97274	23.45676	9.379951	9.501158	7.228424	8.966439	5.600332	8.424061
3.597935	4.68391	4.275612	6.726584	10.7439	4.955297	7.011125	2.181074	5.811831	3.588495	8.876884	10.2421	3.977899	4.129294	2.305663	3.699246	2.923661	3.127415
3.426391	3.141991	2.127591	3.83001	4.675953	4.634021	3.573542	1.011117	3.923126	4.520353	2.621963	6.582059	3.1372	4.363953	1.524915	2.353463	2.75566	2.989915
2.22624	2.429755	2.372952	3.007866	4.203418	2.777177	3.683285	0.6873042	2.592897	2.177587	2.805519	3.684352	2.144205	1.721957	2.120747	0.7903341	1.257942	2.164631
2.362058	1.840151	1.559712	6.5855	3.915394	4.179148	2.694295	1.166664	1.824147	1.930334	2.524097	6.226016	2.451589	3.281882	1.002833	2.369459	2.223983	2.089239
0.1956353	0.08477985	0.1456009	0.11915	0.3744154	0.1538283	0.07073296	0.166165	0.3020278	0.08061081	0.2414648	0.2411977	0.04258776	0.08804258	0.07501152	0.1067017	0.06512381	0.04839092
0.2917716	0.1685882	0.04108242	0.09692778	0.2582624	0.5647271	0.07672111	0.06938945	0.3703666	0.3306144	0.175444	0.708867	0.2117187	0.2297876	0.07458172	0.6365418	0.3035188	0.1082557
0	0.141204	0.07864984	0.1082448	0.05612426	0.09459944	0.0514073	0.05313679	0.05365745	0	0.02474884	0	0	0	0.1332635	0	0	0
0.1912744	0.5301015	0.2915261	1.172807	0.5467508	1.213772	1.355822	0.01893832	0.3155442	0.07018193	0.09996748	0.107804	0.4449365	0.2787354	0.1583201	0.1013425	0.100511	0.4366245
62.13688	66.66917	19.19193	36.11328	63.13247	62.05929	26.382	20.959	44.70132	52.26346	58.39651	102.7176	21.16103	91.28038	41.73029	37.56252	32.7747	38.62764
7.65675	5.388545	8.789783	7.520771	8.332766	6.933432	20.69852	8.875368	6.138265	8.806612	12.29365	9.456411	6.853506	6.437862	6.43419	4.900887	9.530274	6.515979
13.91253	10.75355	17.06722	30.75271	34.27368	21.27711	26.74455	3.98669	16.93904	13.6155	17.17097	22.07897	17.22067	20.74582	6.091612	11.82066	14.15709	15.8043
5.612501	6.074699	3.710879	4.428611	11.75404	5.886035	5.313625	2.354907	6.883823	6.269402	4.079617	9.915825	10.17431	9.145715	3.160042	4.862452	4.097661	4.556325
150.5663	84.41549	69.16861	114.853	201.4652	140.3148	174.7949	34.99133	238.5603	138.8112	109.3869	227.3886	67.85416	104.6798	95.75828	65.66986	108.1781	88.17331
1.364304	1.978591	6.39475	10.4038	6.501258	0.9329006	2.667202	4.954661	2.314034	0.9920269	5.558805	6.327485	6.456256	2.084953	1.436782	2.985888	1.748157	0.9632664
1.302544	1.934809	1.820022	1.941188	4.161759	1.454787	0.9411447	0.5058599	3.012508	1.59059	2.04999	5.366841	0.660259	1.78973	0.3307196	1.379504	1.027298	0.6295622
2.120124	2.115084	1.368397	2.394669	2.974717	2.588746	2.843173	1.943911	2.97535	1.60886	1.853555	2.891615	0.6211408	2.027522	3.800349	1.750769	2.193353	1.820169
1.586062	1.160175	0.6808293	3.239854	2.388015	8.64005	0.4148354	3.527786	3.326175	1.221315	6.517901	6.011961	1.561057	1.850265	1.05094	1.310238	1.456142	0.543957
0.01818831	0.0446648	0.1990243	0.05991883	0.5732703	0.2244234	0.05081514	0.0735346	0.8221103	0.02123421	0.2006029	0.2578791	0.01682747	0.0985654	0.01317284	0.0843209	0.01286601	0.03186741
29.9834	298.0528	377.637	564.5223	693.2839	424.2042	517.6987	219.9254	453.1556	168.412	488.8932	866.1184	139.7186	105.1415	170.7267	258.7015	112.2111	65.68879
0.06628405	0.1831195	0.1851048	0.07798696	0.426822	0.8803473	0.283953	0	0.4735667	0.1354225	0.5289793	1.457362	0.143091	0.2958151	0.0720091	0.1536462	0.1172197	0.07742338
0.7413571	3.969376	1.388059	2.249735	5.130508	3.253284	2.453785	1.562983	3.097527	1.144552	1.739283	8.016361	1.474224	1.869942	0.7569778	3.657604	2.137798	1.890884
1.611266	1.442571	1.346825	1.685108	2.803179	1.817843	4.038946	0.4782311	1.97735	1.672085	2.323716	3.255687	1.642544	2.439743	0.9886708	1.582149	1.329736	1.866363
4.619572	4.003844	4.187955	5.906586	7.520108	6.601956	5.897007	1.423476	7.548321	5.762077	7.031141	10.71305	4.443492	6.043504	6.325276	3.27397	5.230241	5.022405
66.34364	47.6579	57.58282	103.9469	84.37686	95.50407	136.9679	27.23861	81.19582	75.31267	62.58712	100.5763	61.59339	74.35731	53.53863	36.00593	61.18775	64.13665
0	0	0	0	0.06489574	0.04101903	0.02229059	0	0.01163314	0	0.1717006	0	0.0492103	0.07630012	0.02889202	0	0.02821904	0
0.5785559	0.3612082	0.5029773	1.190655	2.257226	0.9326734	1.808165	0.2378723	1.16956	0.5208928	1.081529	1.759066	0.9798138	0.4438792	0.4403237	0.7501016	0.5271793	0.6666216
0.09803604	0.07222376	0.8045645	0.2161895	1.993523	1.437186	0.1095586	0.08736004	0.6812238	0.1144536	0.4129136	0.1588552	0.03455278	0.1607213	0.08926013	0.2726967	0.02773941	0.03533496
18.39648	19.09543	11.60872	15.27622	25.24225	18.26858	17.61168	5.921175	17.83174	21.18753	11.97584	25.04627	9.384393	14.97545	8.970029	9.645538	8.83445	12.2569
2.246114	2.920495	1.733913	3.475237	4.331041	1.867646	6.104609	2.076269	3.201671	2.612791	2.413958	3.482354	1.513742	2.185059	1.62087	3.452194	2.263778	1.818512
0.05563298	0.01517969	0.5368926	0.1920024	1.21172	0.7309408	0.1312515	0.1856498	0.2487568	0.02886646	0.1928896	0.2210109	0.03050107	0.1103471	0.1163993	0.09313567	0.01749046	0
38.08639	33.40295	37.11418	60.49356	51.77463	51.00288	64.26734	22.10926	40.96504	28.94407	49.60253	48.8886	31.88634	34.93543	26.37248	18.81599	23.24216	34.01002
10.1533	23.49488	6.718337	14.62918	6.910343	9.735109	19.82272	7.518204	12.52659	30.9106	9.599211	10.04646	14.07708	8.000239	10.47089	10.62107	11.02502	5.972379
0.3338541	0.6760632	0.5282111	1.163152	3.033676	0.6865645	0.5234445	0.8259717	0.504217	0.596279	1.034809	4.168288	0.3718798	0.5273552	0.487195	1.882706	0.743731	0.3562021
5.08933	5.020794	4.0037	13.90021	8.709863	27.43572	2.268425	3.364194	10.29442	4.49914	35.8962	21.72537	6.568381	6.601922	3.068062	4.671087	4.078703	4.164667
4.652024	4.892739	11.62489	23.59453	24.35913	5.431628	4.10972	14.7223	23.70008	2.014802	24.70334	25.08573	44.99192	7.432311	6.072582	11.56275	7.447364	2.201755
1.511468	2.991849	2.224012	7.169356	4.399773	3.616935	0.5272092	2.142749	1.800449	3.309934	4.489771	5.172569	2.63856	4.164066	3.655558	2.988658	1.969881	1.54078
0.482733	0.2481156	3.247677	2.451487	8.59258	5.067545	2.458982	1.685828	2.376738	0.7601692	1.207979	2.394477	1.440248	1.302636	1.707438	1.639427	0.07941271	0.4248608
0.01936471	0.1241679	0.0647463	0.01012608	0.08400498	0.05088512	0.06011311	0.08698969	0.002509773	0.04772717	0.03704327	0.05836023	0.013271	0.065845	0.03116632	0.04488733	0.01217615	0.04222219
172.6414	126.1119	124.3741	174.8008	302.2346	193.1793	260.0285	42.02022	277.669	226.4165	191.0195	311.6544	146.0082	179.7327	128.295	99.5003	147.9719	132.5948
0.3189604	0.9365149	1.343601	0.9659255	3.365163	0.8745811	1.017688	0.6140685	1.685015	0.6853858	1.153975	2.735614	0.5645314	0.9607679	0.3749668	0.9536534	0.3858523	0.2008601
3.14209	2.676395	5.475286	8.212185	3.409204	7.689596	5.727068	1.245557	4.145203	4.311242	3.449573	4.174054	5.828303	5.861863	3.09858	2.465859	3.871174	3.461571
0.04418937	0.09495085	0.4570956	0.2079652	0.07188581	1.482391	0.02469156	0	1.014786	0.103179	0.2139692	0.07491114	0.01362771	0.05634572	0.2960374	0.01920578	0.06251716	0.007742338
0.3978338	0.2880766	0.4185835	0.4032638	0.3567803	0.4824912	0.5243915	0.4949002	0.6246869	0.2977287	0.471983	0.4024509	0.1761693	0.559305	0.5614844	0.5674938	0.2453395	0.1286838
0.0898108	1.744328	0.07817318	0.1229586	0.5711231	0.3693883	0.01824848	0.02829362	0.819031	1.210552	0.3426268	0.1610578	0.01007166	0.4372485	0.02365282	0.861113	0.02310188	0.2975456
0.05059386	0.04141423	0	0.03968437	0.03429351	0.05202264	0.009423395	0	0.09344073	0.03937769	0.01814669	0	0	0.05376006	0.01221416	0.02931911	0.01192966	0
0.007402634	0.01272499	0.0172131	0.1017284	0.1372829	0.1293987	0.009927236	0.005130609	0.05180881	0.008642313	0.07965393	0.06936267	0.04200581	0.01887815	0.01072268	0.0360345	0.04189168	0.004150406
0.005344534	0.03937351	0.1096541	0.08048831	1.42152	0.08792743	0.0238908	0.02469455	0.6483496	0.04991643	0.2472858	0.04612473	0	0	0.3096613	0.01238861	0	0.0149825
0.2144689	0.2824859	0.5867029	0.7267246	1.579525	0.577376	0.4488484	0.06306103	0.9483612	0.1957554	0.5454638	1.399009	0.158738	0.4226334	0.0847247	0.2530886	0.1875694	0.08198554
0.04812462	0.3714197	0.2491948	0.01294202	0.7157719	0.3534548	0.1767087	0.3097171	1.271056	0.1685514	0.2367228	0.508565	0.008480755	0.1051947	0.2788331	0.02390416	0.09726369	0.01927275
0.005140594	0	0.02109397	0.01935425	0.08362549	0.05814341	0.01723437	0.01187612	0.04497183	0.02400584	0.04425111	0.08239153	0	0.02621902	0.0297845	0	0.007272684	0
0.02591487	0.1007615	0.06203131	0.1199286	0.4865674	0.1176896	0.2027253	0.2020618	0.2896888	0.02773347	0.1394248	0.4499668	0.1625036	0.1018856	0.2002004	0.1351588	0.02138687	0.03632398
1.094618	0.8922482	1.281237	4.87735	2.122361	2.604167	1.319216	0.8110751	1.648774	1.045252	1.530852	1.852321	3.559173	1.735883	2.078534	0.9914741	0.8025045	0.7950791
0.4612565	0.4606321	2.132471	1.186692	3.926868	1.808597	1.896929	1.385311	0.9218292	0.5313206	0.635292	2.024503	0.4703685	0.7763537	1.086821	0.5274677	0.4959496	0.5172218
5.033548	6.832786	5.221054	9.659857	7.642516	8.511144	10.15654	6.105128	9.557302	3.460599	5.927735	9.239796	9.796822	4.742381	9.032826	7.195111	4.154057	7.447313
4.198982	5.169818	2.549118	5.863441	2.704235	5.695633	4.152546	1.136184	4.216892	5.5334	3.39793	4.489012	2.284769	4.180605	2.099611	1.919979	2.042567	2.475163
0.2647415	0.08315518	0.1642147	0.1796463	0.3906102	0.2468953	0.1238474	0.1991329	0.2908537	0.1365686	0.112622	0.2201291	0.03797422	0.1099069	0.2051155	0.08562838	0.1306551	0.09492719
6.54903	8.878435	9.264529	11.71413	17.65639	15.30142	13.72472	4.283738	16.63081	8.64618	12.40662	23.67432	7.008149	8.029827	5.688575	6.749296	7.268807	4.656219
1.88407	3.963673	2.395067	6.992208	5.574505	1.740129	7.263254	1.446379	2.74538	2.859689	3.197787	5.24151	3.533272	2.504654	0.9436961	1.03853	2.510496	1.606988
161.6317	104.6902	121.025	191.5435	310.1088	179.2564	238.5913	39.42315	255.9417	181.4087	178.6992	345.7146	134.8544	147.5996	128.3106	84.00091	125.2469	103.6246
0.2444519	0.2234684	0.158639	0.07557677	0.3773476	0.1981487	0.1196423	0.2679462	0.2393518	0.2124793	0.2015965	0.2199879	0.1078535	0.1865649	0.05427618	0.1178774	0.1792308	0.2125867
0.3650374	0.4482089	0.661572	0.5325643	2.21202	1.482617	0.7138981	0.9065822	1.277393	0.362243	0.8837732	1.012618	0.5178458	0.4421852	0.7138193	0.4230779	0.3615072	0.1534981

TCGA-DU-8158-01	TCGA-12-0618-01	TCGA-TM-A84C-01	TCGA-TQ-A7RK-02	TCGA-WY-A85B-01	TCGA-HT-A615-01	TCGA-FG-7634-01	TCGA-41-2572-01	TCGA-DU-6542-01	TCGA-HW-A5KM-01	TCGA-14-1402-02	TCGA-P5-A731-01	TCGA-HT-A4DS-01	TCGA-06-2563-01	TCGA-06-0219-01	TCGA-FG-A4MX-01	TCGA-HT-7690-01	TCGA-VM-A8CF-01
1.325718	0.4002589	0.02588982	0.02966062	0.1274679	0.1976582	0.3215465	0.9409135	0.1385909	0.1521889	3.23307	0.1999148	0.1362372	0.1290678	0.4099646	0.03836672	0.1481075	1.539696
0.2520215	0.497528	0	0	0	0.1180789	0.1751011	0.04719317	0	0.04952337	3.542458	0.648779	0.06058786	0.3359968	1.893332	0.07312531	0.05566212	0
2.047772	1.245502	0.7808631	0.7113953	0.9769048	1.095843	1.510456	5.596046	0.8943765	3.275579	5.545056	0.9649127	2.954193	5.519903	5.854078	0.4802729	1.43251	1.250828
0.4523462	0.8731545	1.012624	0.06026552	0.06152182	0.047097	1.372373	1.118115	0.8308255	0.7436971	2.032149	0.7461266	0.1159973	0.4221499	2.386353	0.01750007	0.5394944	0.195197
11.60641	9.542692	46.63479	9.879065	10.46246	7.227735	13.12823	136.4575	14.04899	67.18354	40.04932	14.08449	19.24157	17.92068	34.85966	15.46579	11.63223	27.15886
4.491126	3.014738	3.051007	3.388916	5.804673	3.875578	5.588322	4.15247	4.561132	15.82177	5.495803	2.609052	5.116711	3.579296	4.791666	1.499104	5.033969	5.398325
3.175206	3.17019	4.971763	2.652489	2.470562	3.15733	3.222648	5.630247	2.634172	5.270086	2.854118	2.153096	8.758709	4.272308	3.798752	2.246525	4.19907	4.568167
6.43785	2.73557	3.198651	3.2429	4.137411	5.569762	2.415352	4.99164	2.566223	6.524345	5.344275	2.049574	6.119103	5.329562	4.882422	2.317174	3.294047	4.327321
0.0741745	0.1659557	0.05083083	0.01058805	0.06984125	0.09267405	0.1288386	1.277862	0.04442495	0.4226932	2.299875	0.09547373	0.1961532	0.2472249	0.965886	0.02152211	0.03276475	0.2489501
7.05223	1.720046	4.160348	2.992307	4.805036	4.299451	4.360366	4.434275	4.458677	14.72567	3.005785	1.864497	6.952733	3.49613	4.377232	1.182689	5.710509	6.661818
0.06642493	0.05037162	0.07153166	0.03476672	0.03822158	0.0271699	0.1974243	0.1694024	0.03646824	0.1880225	0.0863066	0.04976124	0	0.0985737	0.02323492	0.02019132	0.07684696	0.02502392
174.8509	24.61663	89.06121	54.41015	111.2061	50.37093	134.0114	128.5003	151.1792	232.1224	82.9743	30.15111	166.1103	71.59454	59.16768	21.55477	166.8058	112.7511
0.9653237	0.460085	0.661297	1.169558	1.635513	1.300073	0.9512995	0.9041975	1.138468	3.602168	0.9755318	0.6968206	1.381444	1.026801	1.032268	0.4881466	1.208814	2.351827
1.210127	0.3378262	0.3995472	0.5058385	0.4562913	0.4186216	0.3288033	0.3998486	0.4795761	1.406078	0.3192959	0.282639	0.2348241	0.3104185	0.2009919	0.1647769	1.397248	0.08168588
3.1444	0.8417352	3.32207	2.48041	2.409557	3.117734	2.760933	1.417801	3.235532	11.08	1.239414	1.096282	2.328966	1.575257	1.075586	1.064321	3.80666	2.536647
0.3171097	0.4115913	0.4670941	0.1316352	0.09438006	0.1690675	0.3621409	0.1952081	0.2521416	1.733318	0.9033743	0.2236322	0.2795304	0.4142688	1.305251	0.02326714	0.3453582	0.09611973
0.2225235	0.1263331	0.04385405	0.1027663	0.1054466	0.2023838	0.194521	0.1348129	0.07186388	0.2970856	0.2182626	0.06177712	0.05769212	0.05598918	0.1682652	0.1253346	0.1219041	0.1380732
8.403618	3.223539	7.185381	2.91504	7.399625	2.257275	5.931646	5.232622	4.598193	13.26327	4.234106	2.129317	6.590747	3.019911	4.327735	2.23666	7.3936	7.242923
828.6075	215.8833	446.9273	132.6453	270.0277	415.5523	277.7143	469.2892	555.4742	2055.157	265.8517	173.3229	1207.377	375.9912	307.5783	146.0432	757.2913	930.631
2.558873	0.5699237	1.045243	0.6346271	1.227756	1.185785	1.607985	1.632621	1.582086	4.997874	2.643618	0.5037132	0.8189678	1.929251	3.561704	0.1675317	1.868214	1.231931
40.06195	16.85949	46.08098	17.71065	31.29508	14.08016	54.49587	32.56083	34.93098	98.36805	48.28881	18.78699	32.4078	35.64841	41.06379	17.08033	84.36372	31.29658
0.1501896	0.04689685	0.02589892	0.1017293	0.1321727	0.1517739	0.1087828	0.05459419	0.08245623	0.1432245	0.05356863	0.08339144	0.08566492	0.06478152	0.08112041	0.04699617	0.121628	0.1475521
4.495524	1.641284	3.418848	2.587903	4.168621	3.194466	5.546817	4.106966	4.425323	10.44504	3.173853	1.646935	2.803117	4.450868	3.086831	2.494235	4.161358	3.940155
13.56839	7.349522	10.5976	15.79836	18.54	13.41607	11.19932	28.87359	13.14195	47.38603	19.7586	6.768513	51.65309	16.31847	36.95366	9.320221	21.61829	53.53142
3.791533	2.113411	4.477666	2.219502	2.443535	1.944048	6.027331	4.201538	2.855437	8.243465	1.835946	2.724221	2.241751	3.561985	2.396121	2.789183	5.434924	2.946998
1.399985	1.000771	0.7663189	0.9303798	1.825057	2.205488	1.280103	1.471789	1.320354	4.601989	1.413322	0.7128206	0.7527367	1.064894	2.613615	0.4078982	0.6774268	2.87996
42.44631	9.146635	18.81744	11.16333	22.87177	32.19161	15.13496	29.26003	25.63323	96.71039	23.40705	10.44777	60.71305	19.18871	22.88665	23.09507	39.83025	56.20787
13.75242	6.587838	7.688781	11.95982	17.54377	12.338	11.83755	9.217581	13.3808	45.82274	5.381276	7.074532	12.37546	6.637655	2.939865	5.904121	16.12763	19.51258
0.3741972	1.003792	0.04148173	0.02304168	0.0126657	0.1764674	1.140205	0.823685	0.0725081	0.07929863	1.292954	0.681023	0.08731394	0.2891806	2.061537	0	0.1381487	0.04837184
0.8728732	0.3996536	0.657325	0.6027258	0.7488517	1.327904	0.4822456	0.8231727	0.5369573	3.256365	0.7891108	0.3929308	0.7926084	0.7711403	0.5986928	0.3692229	0.8048195	1.532243
0.1063706	0.05279785	0.04200106	0.01908831	0.2150979	0.07309503	0.05032568	0.04382137	0.1401576	0.252918	0.442267	0.02868694	0.05625905	0.1949943	0.06697368	0.1261014	0.09229507	0.03205796
1.378458	1.105137	0.8072716	1.845387	1.867604	3.028524	0.69954	1.640296	1.546753	3.757153	2.072903	0.6738975	2.222041	1.620862	3.882844	0.4907957	0.9339697	4.330254
10.82277	4.697306	7.310161	10.8263	12.64535	12.01644	11.80673	8.200099	12.055	32.06976	11.10195	4.451648	15.03222	9.522449	24.55016	3.674374	11.97963	20.13216
1.866359	0.467076	0.4207333	0.9939501	1.297166	1.375122	1.071615	0.7907536	1.244388	3.58402	1.244328	0.2826904	0.2807974	0.9433001	1.252463	0.02606945	0.8780863	0.4092464
5.458299	3.252628	3.297406	5.614855	9.294167	5.169515	3.970484	6.754285	5.556275	14.9631	6.520017	2.324515	9.206599	3.846533	9.974106	5.943479	8.417132	13.789
3.07552	1.232739	1.541361	1.957177	3.302445	2.62252	1.723281	4.373405	2.029896	5.839609	2.426566	1.546688	9.659052	3.732939	2.515326	1.796946	2.371558	3.959162
5.177865	2.750842	6.137226	8.646174	8.364177	4.046375	6.116409	6.612995	7.608846	10.89701	7.443882	2.578236	8.469002	6.175511	15.8696	3.916765	11.17542	12.98631
4.543562	3.76861	2.394931	1.110373	1.668899	1.789991	2.349802	2.976593	1.243489	1.875616	1.367126	2.44532	3.062565	1.828639	2.239661	2.671368	1.933818	4.504402
0.5927411	0.7727792	0.7446959	1.095267	1.571557	1.305211	1.622665	0.6475778	1.402377	5.38333	0.4616673	0.4842768	0.7707565	0.4742571	0.8359312	0.5957776	1.623048	1.571738
22.90217	7.960777	6.534751	11.5565	16.80419	20.53296	16.24333	12.10739	15.48779	72.00223	17.20721	4.910479	11.52052	13.34266	21.85621	3.15359	14.13007	15.6631
9.396476	2.750119	6.545137	4.082518	5.803204	6.029132	8.434757	6.181672	7.727139	32.14415	8.464233	2.454171	3.638269	10.7671	8.749454	1.439215	7.374903	4.189745
3.567268	1.097528	2.923196	4.475854	5.877156	3.765085	3.416701	2.021556	4.694907	13.85744	1.278741	1.626343	4.104409	2.486377	1.599771	1.601385	4.93275	6.579189
2.790527	1.272188	3.916983	2.15781	3.195258	1.67025	3.084142	3.225455	2.725337	8.80531	2.083142	1.308915	2.138448	1.987926	3.656713	1.238263	2.577821	3.660924
3.314362	1.220169	3.190992	1.153508	2.103727	2.37733	1.516058	2.94432	2.166675	6.647158	1.696286	1.433429	5.632345	2.129646	3.085093	1.417733	2.241335	5.391302
0.3097728	0.1048351	0.1061417	0.1073879	0.1180593	0.1382257	0.1178618	0.3148984	0.08613922	0.1130476	0.2012455	0.05063164	0.1489434	0.3097435	0.3102925	0.2953271	0.1661562	0.02121802
0.1259992	0.1389792	0.3241386	0.1601589	0.2900048	0.4226087	0.4891263	0.3460515	0.4348165	0.5511916	2.100971	0.235976	0.06346716	0.2749721	1.928216	0.03830021	0.6413807	0.2373351
0.04824358	0.02328089	0	0	0.02775985	0.08287923	0	0.03312475	0.02648642	0.03476031	0.2925225	0	0	0.0294794	0.6792277	0	0.03906908	0.1696292
0.3744547	0.2378609	0.1056112	0.2039886	0.4946901	0.242874	0.282289	0.2282472	0.1950922	0.5285895	0.4691567	0.1202216	0.5052239	0.3432176	0.4069776	0.1097586	0.3527538	0.8363221
75.4173	31.54764	38.39066	75.20185	86.05311	60.32167	50.13638	59.39123	48.43214	185.3253	47.43156	21.64026	39.7049	32.49527	57.21306	25.10532	92.28541	98.02169
11.28035	7.527367	5.571618	9.01361	8.806492	7.650616	7.769397	12.38152	7.557652	9.716933	17.09239	5.634138	14.62555	11.50621	20.65539	10.86507	10.84777	8.263774
27.10572	8.139442	20.6885	22.67066	22.50018	15.8019	20.66169	17.23489	24.0764	80.88258	9.321943	8.232344	23.37331	10.78988	14.14693	3.477481	26.05658	31.63271
5.379892	2.28371	5.465136	9.546067	9.827046	7.63721	7.450591	5.988435	7.479528	43.40645	5.157918	3.069578	2.608672	6.24553	7.557397	1.733747	7.073635	4.652659
174.8186	66.1318	78.20914	102.8585	198.1676	98.41518	84.65316	165.0878	131.0969	762.8774	161.6566	49.80246	403.6315	73.18636	120.8798	34.54366	269.2665	319.3629
8.878454	2.590559	8.99559	0.9365796	2.390259	2.248649	2.632069	16.56228	1.87127	4.504898	4.477799	2.785296	2.234597	4.881386	3.51648	1.692237	3.318035	1.061113
4.276115	0.9888246	1.975995	1.77515	1.581118	2.122001	3.670096	1.423101	2.913728	7.636537	2.731247	0.8110413	0.8304612	2.3171	2.772953	0.5763274	2.613088	0.683211
2.5107	1.180292	1.350198	2.327552	2.17502	4.838439	1.07389	1.564853	1.327546	7.63986	4.508066	0.9036424	2.621501	2.700368	5.410307	3.637085	2.172039	3.200509
2.147079	2.886322	5.795186	0.3334287	1.547708	0.9322678	7.768907	4.884353	0.8355069	5.954275	27.73628	5.679073	1.466274	4.044069	22.9209	1.581426	3.367669	1.306617
0.8615608	0.06443563	0.04793049	0.004991953	0.1426884	0.2348504	0.2956189	0.2292023	0.1204341	0.8040218	0.3108394	0.04001161	0.1261097	0.2272906	0.1634722	0	0.2857809	0.03353505
679.9947	194.3366	369.2065	70.85653	220.2167	410.5533	286.4565	422.3058	471.4028	1727.186	869.0709	136.0169	515.6132	387.899	612.7618	47.64457	825.4341	452.7002
0.8032956	0.7436098	0.2280468	0.05457685	0.1133339	0.0862505	0.04919317	0.1034165	0.5088702	0.2086976	0.7727619	0.2369497	0.07149091	0.9274354	0.8652492	0.07395817	0.04691341	0.03055312
5.322153	0.9513274	3.138652	1.965788	1.911772	2.929296	1.500882	1.686137	1.576821	5.054091	3.902871	1.122899	2.23212	2.28981	4.442094	0.5062575	1.868294	2.210928
2.057523	0.9060672	1.523473	1.510943	2.917035	1.760624	1.903335	1.837081	2.242039	7.846435	1.33414	0.935367	3.703248	1.778325	3.419285	0.324605	1.710592	3.775395
5.478861	5.539466	4.574144	5.404555	8.885734	6.269817	9.325752	7.472159	6.782495	24.32079	9.473954	3.816105	9.113894	4.608729	18.08623	2.453809	9.408826	10.49822
90.46836	40.3107	35.57448	61.59604	84.22839	69.07742	48.61793	114.468	69.09328	228.191	132.8136	30.0744	201.5944	58.55115	241.2757	43.43337	108.9422	246.6461
0.04183756	0.1009477	0	0	0.1203688	0.02395804	0.05329163	0.02872628	0	0.01507233	0.09512996	0	0	0.02556498	0.07683085	0.08902212	0.06776258	0.03677624
0.9975635	0.625316	1.124092	0.3283456	0.8580535	0.4652441	0.4235571	1.129801	0.7565843	3.427083	0.6469588	0.3829	2.085048	1.351096	1.309424	0.1641201	1.011903	2.088247
1.308213	0.1672767	0.1894553	0.202955	0.263691	0.1682202	1.092619	0.2541422	0.3677154	0.2116593	0.8683356	0.2926881	0.1864426	0.218994	0.08991069	0.08125839	0.5400236	0.06713788
17.95596	7.209173	11.34464	14.72337	22.36424	21.8608	20.26603	11.7243	20.18674	75.13175	6.959743	7.552903	17.38029	13.32976	9.938947	7.512968	20.37434	23.94875
7.088626	1.622374	11.3015	2.056374	2.492339	2.408935	2.544384	3.121934	1.814632	4.946801	1.250429	1.861096	3.6482	5.844272	1.655399	1.483792	3.663808	3.363884
1.14098	0.1439075	0.1574657	0.1085795	0.06714526	0.1781934	0.181669	0.2225607	0.05694676	0.2242078	0.3636024	0.05439306	0.1828668	0.3327543	0.1507985	0.08276528	0.3359998	0.05698577
53.87346	27.9107	30.1765	37.23126	53.3421	33.60684	31.80228	52.4931	36.681	165.0953	67.01511	20.66529	55.76936	49.71786	122.9663	29.35223	62.22719	108.9861
9.131503	11.13798	21.29074	7.678823	10.85086	4.919874	6.499718	25.72086	7.345106	16.25615	15.07915	8.69568	20.90696	7.496629	9.961222	24.45862	11.50518	22.67242
1.722791	0.5043681	2.683124	0.8643252	1.470428	0.6224519	1.626421	0.7786285	1.629632	3.360546	0.6060084	0.8769314	1.4741	0.8462183	0.4926361	1.273194	3.182504	1.993647
10.387	14.17383	14.21266	1.970074	9.728551	3.215484	34.63562	31.01303	3.828091	39.79121	153.5397	17.73204	7.071034	19.83284	151.6539	1.969441	11.49322	6.942743
34.24654	8.556085	43.68588	3.042384	8.766002	14.20428	7.792468	67.87925	4.706762	58.01921	33.80875	12.5134	7.052044	13.00904	10.81286	6.969673	16.79693	4.373385
4.819541	4.418181	0.7040787	4.354527	2.664496	3.454897	7.052725	10.55416	2.336565	4.097852	6.101888	6.906946	1.016228	8.118798	2.275881	1.246958	2.540196	1.528516
9.623062	1.238599	0.7691861	0.5176378	1.449791	3.263205	2.249563	3.637807	1.357429	1.73905	8.049156	0.8643712	6.725252	3.654743	4.324276	0.3507307	3.890164	3.642988
0.06167889	0.01742304	0.02457027	0.07086435	0.06492194	0.1059602	0.05173794	0.1146539	0.05203277	0.2325006	0.08209466	0.02129975	0.007956525	0.104794	0.07182826	0.04321336	0.02010158	0.01190135
231.42	116.2923	118.0544	186.7405	257.8877	165.0612	150.4232	252.2431	229.4976	1053.785	166.4593	67.24147	298.8699	115.705	206.1893	49.73912	324.7976	358.0001
2.7569	0.3322092	1.372469	0.8576548	0.8591944	1.188206	1.181535	0.4593623	0.7878411	9.763131	0.7642866	0.4118474	1.324782	1.730036	1.151444	0.08252486	1.966956	0.5966124
7.231001	3.632249	1.929997	3.653165	5.037729	8.160887	2.772494	3.406419	4.566302	10.0317	3.188825	4.98643	5.916752	3.952897	7.272227	0.5304083	2.520916	8.187562
0.4595778	1.800318	0.07278088	0.03032047	0.0133334	0.1061545	0.1180636	0.3659352	0.03180439	0.03756556	0.2107533	0.3827648	0.1327688	0.02831864	0.09219868	0.1109373	0.06567877	0.02036875
0.4421941	0.1858554	0.1164873	0.2575746	0.1969881	0.6493867	0.3452213	0.7198661	0.2584336	0.2929141	0.3405584	0.2244041	0.5469656	0.4314551	0.4191221	0.06829131	0.3725415	0.2821206
1.621207	0.3305685	0.0143438	0.4033541	0.01970829	0.07845422	0.5817051	0.16462	0.02820631	0.06786536	1.142231	0.9609113	0.1358637	0.648802	0.3669083	0.01821977	0.1109494	0.1956977
0.2240343	0.03414069	0.02962817	0	0.08141785	0.01012831	0.03754858	0.02428819	0	0	0.02681093	0.06492445	0.01559091	0.03242294	0.0433072	0.07526858	0.04297016	0
0.01759744	0.06443924	0.007803075	0.004876135	0.02144276	0.03556614	0.1015276	0.006396702	0.03750837	0.02013763	0.110624	0.05536801	0.01094968	0.04933717	0.0817407	0.003303874	0.04903996	0.010919
0.2653093	0.3354032	0.01877881	0	0.3289751	0.1348092	0.6568496	0.5080101	0.3323478	0.04846305	1.384946	0.08230033	0.02964532	0.1918017	0.102933	0.1669725	1.348141	0
1.281397	0.5091633	0.4144626	0.3810043	0.4047466	0.9414304	0.8543066	0.1403978	0.7094928	2.425052	0.5331328	0.231611	0.2883943	0.3598477	0.3554787	0.01740359	0.9637488	0.3594829
0.3701215	0.8559335	0.0181171	0.01509515	0.4646654	0.1981851	0.7347295	0.1386168	0.3641802	0.9766675	0.1858033	0.06049548	0.2923628	0.16742	0.08827198	0.03068361	0.344501	0.3168953
0.04312993	0.0156099	0.0767645	0.005643537	0.0124087	0.01234906	0.04578153	0.03701706	0.01183948	0.02719136	0.01634476	0.01696283	0	0.05929805	0.02640139	0	0.004365987	0
0.4001271	0.07213523	0.2257423	0.1043178	0.06516147	0.2101083	0.1115508	0.1368482	0.08206746	0.3639086	0.861741	0.1211439	0.267527	0.2158972	0.3909674	0.0867456	0.3521585	0.1154709
1.828637	1.284873	4.301277	2.272146	2.860598	1.44368	3.301396	5.307834	2.549299	8.843742	2.64685	1.453568	3.877711	1.27721	1.551115	1.306877	2.583302	4.584814
3.508776	0.7968139	0.3889663	0.3375899	0.5567056	1.802443	0.8982601	0.9831569	0.6657303	1.459253	1.388369	0.2435272	2.331096	1.694745	0.8212364	0.8097303	1.305841	2.347244
9.306639	2.915443	6.484908	5.679519	10.26026	9.169693	8.168691	21.50609	5.635454	27.42812	40.27774	5.16743	14.21868	14.26492	31.16556	3.307384	9.523908	18.04581
10.44827	3.819361	2.59201	3.018472	3.977945	2.521764	4.938274	5.865067	2.993979	6.185085	5.651255	2.682568	2.903413	2.705651	4.549414	1.052549	1.866181	5.366341
0.5509952	0.1246377	0.05408189	0.06759144	0.1263239	0.1700873	0.1644947	0.195072	0.0638095	0.09304718	0.3132123	0.1151241	0.2618222	0.2525156	0.1027663	0.233566	0.3398882	0.1475718
18.3564	5.196836	7.924441	7.233808	8.357544	18.7546	7.128238	11.32764	11.63161	40.46357	20.55629	3.8143	18.31156	10.60449	32.8156	2.074999	16.10435	20.32429
6.449659	4.208191	5.281188	1.550766	2.122822	0.8906365	3.988353	7.875374	2.744147	2.844432	2.836618	2.070187	7.213084	2.472477	4.428705	6.14288	2.650334	5.017698
210.1682	114.4526	124.7592	146.5073	185.2126	137.8279	140.5953	234.2302	200.0482	1053.5	166.4284	58.37858	451.9095	106.0476	224.8453	53.94373	302.125	466.8684
0.0785955	0.1282321	0.161439	0.2311495	0.2476588	0.1628834	0.3273536	0.2261383	0.3945148	0.842699	0.1843832	0.07261699	0.03958942	0.1738085	0.2176457	0.07167249	0.2273177	0.03618863
1.926942	1.016098	0.3527186	0.2003759	0.3965181	0.7015326	0.9752942	1.261732	0.3993468	0.7033916	1.886062	0.3814386	1.484859	1.099486	1.195174	0.16292	1.209125	0.706697

TCGA-E1-A7YY-01	TCGA-14-0871-01	TCGA-HT-8105-01	TCGA-DB-A64L-01	TCGA-DU-5852-01	TCGA-28-1747-01	TCGA-HT-A61B-01	TCGA-DU-A7TG-01	TCGA-WY-A858-01	TCGA-08-0386-01	TCGA-S9-A6WG-01	TCGA-DU-5870-02	TCGA-E1-A7YJ-01	TCGA-DH-A66G-01	TCGA-76-4931-01	TCGA-FG-A70Z-01	TCGA-TQ-A7RH-01	TCGA-VW-A7QS-01
0.00944513	0.4647014	0.1525396	0.04803944	0.2604284	0.03055574	0.1145976	0.02461223	0.1508233	0.246803	0.111913	0.04342933	1.297249	0.13022	0.3244711	0.3780167	0.1904204	0.01618325
0	0.2479963	3.546073	2.00824	0.06260463	0	0.123967	4.081157	0.0457327	1.084205	3.793304	0.1448553	0.8442684	0.1974269	0.1159553	0.2076688	0.2032425	0.1439414
0.3306046	2.390074	1.109463	0.713783	2.927387	4.144254	2.424874	0.5726545	1.423462	1.91208	3.01338	0.6928537	5.740874	0.7498086	2.872449	1.522771	0.9068324	0.3981895
0.04523582	1.038617	0.2914869	0.5061686	0.1910244	0.3902439	0.2472277	0.2582047	0.0985012	1.85952	0.9174567	0.01733311	0.5411977	1.875724	1.493874	0.8981233	0.08511865	1.601809
8.143547	26.89128	17.10109	16.7112	13.02002	69.23608	40.65974	27.05522	18.83802	38.1503	48.05237	15.05077	24.5571	13.87864	18.98355	19.03258	10.34829	10.43766
1.508859	2.811971	3.655788	1.974775	4.129959	7.112983	8.043966	1.463788	5.289232	3.358414	3.640409	2.138508	3.957735	3.166242	3.038039	4.814367	3.430659	0.9547883
1.919545	7.44953	1.457809	1.101199	2.500307	4.132962	7.623978	1.633297	3.192429	3.207485	10.66872	1.860423	19.32429	3.073068	5.356687	3.336797	3.451506	2.616413
1.344707	6.207804	1.26265	1.823839	5.702285	7.001666	6.840024	1.459316	4.936423	6.641109	4.262451	1.336168	5.815665	1.148363	5.855481	5.202626	7.013739	0.6612658
0.01854412	0.2919592	0.8440144	0.6413648	0.07370272	0.1919734	0.2675621	0.621289	0.1480596	0.3191013	1.140192	0.138559	0.6212089	0.3486379	0.1137591	0.5238918	0.1694842	0.2330051
1.688293	1.321069	1.06076	1.335516	4.528275	7.223917	8.759834	1.595667	7.519836	2.639065	3.221904	1.041249	5.408873	4.852969	2.522856	4.779136	5.272024	1.002346
0.0260962	0.4108595	0.2681991	0.3126516	0.06914541	0.07879504	0.1996732	0.1036218	0.05682459	0.09424613	0.6128433	0.07499502	0.02081419	0.1199295	0.1547512	0.04095814	0.0607958	0.0596175
31.54816	16.85988	9.300173	14.91239	129.6642	234.1312	177.339	37.07336	117.3639	46.33945	56.92496	5.74836	95.7862	97.84144	68.38071	180.7415	52.63981	1.083328
0.3113549	0.300999	0.2994054	0.4185893	1.27003	2.102872	1.827035	0.4636185	2.026001	0.5187114	0.948093	0.3013959	1.309641	0.3286241	0.549547	1.363142	1.929392	0.2589379
0.110742	0.2682347	0.1500832	0.5391808	0.3554967	0.374794	1.054047	0.1353015	0.2555684	0.209925	0.4328379	0.7507419	0.3696153	0.2562461	0.3065776	0.8022008	4.988889	0.4216553
0.7230602	0.5553115	0.5264792	0.6059569	3.151216	4.921738	5.856295	1.011012	4.757535	1.105223	1.472132	0.5135682	2.444683	1.031513	1.262169	2.43577	2.569414	0.278666
0.13031	1.025803	0.2698099	0.7205582	0.4780717	0.8366408	1.288506	0.3507578	0.1382375	0.3896932	1.22622	0.0518516	0.3309916	1.840557	1.094547	0.5286115	2.182547	0.2747971
0.06599524	0.0472287	0.1526807	0.2278435	0.19076	0.1707998	0.1731284	0.1072028	0.06967511	0.1605934	0.3996262	0.06206941	0.2985979	1.022671	0.125135	0.4011368	0.2709402	0.05482472
1.697935	1.712828	1.650517	2.656005	4.258262	9.779215	11.06943	2.79442	10.23726	2.580274	6.395484	0.929005	11.75141	7.538548	3.146253	5.674996	4.390828	0.9675258
90.38906	96.42724	61.51313	97.20888	440.0349	1034.345	835.1771	106.981	811.1509	151.5562	278.3438	117.5721	442.7369	448.9668	200.0463	623.7204	277.6155	64.20519
0.1948732	0.4261236	0.3002396	0.4209354	1.477314	2.451672	4.137095	0.2579316	1.03203	0.7359814	0.6517906	0.2115649	1.758084	1.135296	0.841244	1.648216	0.5587595	0.05358808
6.299982	10.87227	7.324304	10.6406	46.276	49.55151	103.4091	13.14843	51.43005	23.26097	12.89885	4.567689	41.22259	40.65854	27.37606	57.79201	5.836695	3.782392
0.1052827	0.573776	0.1070134	0.06041367	0.08449294	0.08907946	0.04780267	0.08441445	0.1116877	0.09290624	0.254162	0.02792867	0.07751348	0.07612937	0.04968141	0.1105849	0.2612397	0.07400657
1.851749	1.542822	3.437536	2.09601	3.064299	10.64599	8.545441	2.660042	5.444931	2.928864	5.918434	1.033233	5.739604	3.933134	2.67268	2.609334	3.851339	0.8105643
4.919466	6.84978	4.753283	5.703118	19.05469	21.09191	35.03733	7.875377	28.65044	21.91599	19.53362	6.018224	19.29922	9.230545	10.83684	22.94366	16.69462	4.975217
1.733217	2.244926	5.335992	2.204325	3.532328	4.001997	6.362659	3.149757	6.462976	2.728015	12.69675	1.190325	4.61891	5.42741	2.758321	5.616148	3.10504	1.967142
0.1757289	0.5659122	0.2892763	0.5525218	1.238119	2.542731	4.704274	0.3806058	2.423453	0.7127006	0.5218208	0.4682802	1.949504	0.4104701	0.7840081	0.7597243	0.9018077	0.3010932
5.699352	5.829968	6.580721	6.804221	27.38693	36.65904	50.16737	4.353545	39.87209	9.152625	18.20603	6.203566	20.34041	24.6668	12.79202	29.99511	19.40236	4.192988
4.2682	3.172324	6.48071	7.305306	14.3417	9.218393	30.28866	6.226455	21.41871	5.89334	19.17729	5.603717	15.77242	29.94151	6.124506	24.66307	15.85997	3.494457
2.244784	0.6353611	1.347932	1.696778	0.1854529	0.293746	0.09263357	1.757662	0.02196866	0.7522964	1.085562	0.1275711	0.2655466	0.5879965	1.05214	0.4892896	0.07593583	0.03457262
0.2241398	0.4553374	0.5909279	0.8139432	0.7938472	0.9964414	1.593281	0.6728872	2.27239	0.7234617	1.055827	0.5194604	1.339065	0.9741884	0.5322536	0.9906824	2.5111	0.4831474
0.09193703	0	0.06135498	0.06801549	0.06228399	0.005407701	0.09318425	0.09956181	0.1031299	0.07990016	0.04817714	0.1152907	0.1119926	0.09427955	0.04614455	0.0314827	0.1303077	0.08592249
0.4681939	0.5350109	0.4804389	0.9320393	2.12343	3.469024	1.723491	0.1236892	1.940328	0.6449076	0.6384233	0.5815995	2.854924	0.605747	0.7782577	1.137806	1.494019	0.3105297
3.603525	2.161297	2.653726	3.479431	12.51474	27.81192	30.1549	3.366386	14.93985	4.904891	10.57815	1.099685	12.62574	10.09767	6.237015	11.1176	10.86745	1.205452
0.1740824	0.1326174	0.2143624	0.3198904	1.500938	2.972086	1.487889	0.1337883	1.630388	0.3149448	0.7480959	0.03873108	1.574796	0.7038343	0.4822823	0.9994687	1.261955	0.03207227
2.05029	1.606689	3.205446	2.818576	4.07439	7.323146	15.10152	3.97851	10.03337	2.471484	8.568188	2.794084	6.579678	3.360455	2.891125	5.949552	8.279522	3.380494
3.262224	5.184568	1.873603	2.241024	4.113923	2.442144	4.885204	2.655317	4.120649	4.325087	6.462974	0.9297471	5.772788	2.862611	4.753773	5.172066	2.803646	1.117456
2.667186	1.028382	1.347933	1.004049	4.862216	19.05326	18.17066	3.987744	13.10905	3.482968	7.2235	0.3879396	2.625737	4.918919	4.153916	5.197678	6.877004	0.2611398
1.517265	1.220279	4.873664	3.542359	3.318532	6.242727	2.54469	2.839815	4.998397	2.395012	8.436023	2.176102	10.05746	2.478668	2.50126	3.136313	2.626434	0.9497271
0.3917598	0.5671615	0.5288989	0.6107443	1.078289	1.287768	2.415738	0.5229983	0.8759377	0.459176	0.778691	0.3468092	0.6637199	0.5756759	0.5745715	0.966829	0.9247795	0.4269188
2.941093	6.471717	6.939909	7.721553	15.16379	24.9579	24.21475	3.787427	16.79783	5.94797	10.60009	4.188331	13.8827	9.041497	6.444987	13.89908	13.37576	1.807608
4.543413	1.931604	3.664564	4.022298	8.090259	13.84133	17.80921	3.893389	6.120595	3.749839	4.988457	1.140387	5.356156	5.291104	4.920427	6.258806	4.757583	0.7413969
1.64515	0.6863246	1.035167	1.167079	4.43312	5.621409	8.731041	1.377243	5.183634	1.294908	3.520338	0.862772	3.312454	2.228259	1.711487	3.85169	4.675018	0.3983548
0.5784537	0.8095286	0.8178247	0.8804532	3.295287	4.707469	4.931827	1.244155	3.974692	1.761381	1.753624	0.3989652	3.56794	2.175039	1.963521	2.384715	1.99723	0.3891065
1.550445	0.5547786	2.176708	1.285095	3.252256	5.066476	4.76579	1.947209	4.44463	0.9382262	2.808535	0.7471097	3.020366	1.746982	2.036747	3.362561	3.031083	0.6261173
0.05531804	0.04354646	0.26531	0.1650624	0.2583343	0.3722331	0.3482843	0.156504	0.06424282	0.2326855	0.3046955	0.0763068	0.2276663	0.1317339	0.1696746	0.09376775	0.124908	0.03791269
0.1650033	0.2597817	0.0888272	0.1566568	0.4098736	0.1708155	0.6168268	0.03685435	0.08383558	0.2103203	0.5072645	0.04741849	0.2289939	0.7858748	0.1923208	0.2719223	0.2749973	0.4806171
0.04422445	0.3481357	0.04328657	0.02999102	0	0.2575253	0.02900403	0	0.09629894	0.0281852	0.02832456	0.02541837	0.02116391	0	0.0542591	0.04164632	0	0.025258
0.02101587	0.1654373	0.3548356	0.2565362	0.1618328	0.305946	0.2618767	0.2425236	0.3279621	0.2209988	0.2422817	0.3019761	0.3922342	0.0790216	0.3545359	0.3958144	0.434993	0.04200993
25.55116	15.1212	16.24376	23.75289	58.69409	51.46027	266.9833	42.32408	109.0397	38.06104	57.65649	27.05398	27.78453	52.21805	50.46804	70.03372	69.87939	14.7957
2.327596	8.480011	6.464412	5.844254	8.919622	8.952975	12.27947	3.628187	7.707245	10.96837	9.297757	8.926072	8.855862	6.308817	9.550582	8.049064	7.149637	7.243757
6.703274	3.912263	4.67879	3.70572	23.71716	36.82517	38.47037	8.726585	24.07641	12.07278	19.77178	4.349536	22.06923	11.09486	12.08896	21.87443	17.29619	2.191214
2.115292	2.257847	1.427078	1.629003	6.180665	14.5523	15.66772	2.304474	10.40917	3.823485	4.416454	0.8448652	4.140656	5.235058	5.21984	3.781382	6.624147	7.023427
48.21237	42.7197	46.39882	55.12463	91.79349	148.7658	532.1458	45.63761	192.8872	64.39706	112.9974	67.64655	174.4486	93.43935	58.82134	257.4297	102.4241	71.76263
2.538628	3.663757	1.548216	1.284565	2.422165	12.64329	3.662837	2.79631	2.225324	4.073846	5.08205	1.133606	4.17108	4.128214	9.260093	4.113118	1.165341	1.513092
0.7448724	0.6231897	0.6938547	0.5563847	2.846046	3.417846	4.337634	0.6536968	1.64547	2.192442	1.428907	0.3185059	1.59806	1.41618	1.262664	1.728208	1.632782	0.275393
0.7516085	1.805088	1.708248	1.485925	1.670825	2.851876	4.661973	0.8536055	2.626007	0.9742705	2.300857	1.230082	3.438367	1.197507	1.359781	1.379595	2.479262	1.949894
0.2595439	3.703181	22.9112	20.63728	2.192033	2.896775	8.000268	38.18807	1.106768	6.182308	32.02021	0.8764031	7.825009	7.766609	5.891049	11.09026	2.372105	5.78112
0.01311451	0.1032377	0.2182186	0.1482278	0.1129332	0.2262745	0.2293595	0.05858391	0.02538391	0.07243739	0.2351857	0.07035161	0.1506249	0.1205399	0.1072682	0.1728997	0.07520644	0.01498024
54.7318	109.6946	54.50699	74.23326	441.4044	572.8171	635.1089	66.95597	581.2265	148.4486	151.8772	77.35055	251.3555	368.0977	194.5224	510.2248	164.8832	41.80985
0.005310388	0.5016417	1.855604	3.831746	0.3271413	0.2611288	0.5293777	1.423324	0.107925	0.8664128	4.292263	0.1342963	0.1372314	0.07321434	0.2280365	0.05500892	0.2797864	0.03032933
1.273569	0.7971393	0.8348523	1.014226	2.852067	5.110432	5.491729	1.287457	1.469995	1.146768	2.948445	0.4790422	3.179708	5.799205	1.61033	1.712797	3.433941	0.2580295
0.6561952	0.3387266	0.6528083	0.5106579	2.704214	3.591433	5.065514	0.8649719	3.341833	1.288901	1.653542	0.4204335	1.657649	1.280867	0.9634656	1.08393	2.290196	0.2703289
2.065674	2.200021	2.039707	3.065381	4.38265	11.28969	19.82709	2.958255	6.094368	4.375421	5.626675	1.655184	6.198741	5.787268	4.88241	6.768334	7.309995	1.866817
24.41526	21.95328	21.9251	22.67023	69.6316	135.7593	178.9333	23.30804	135.477	31.48768	75.61684	25.64593	84.03589	41.10981	43.15572	97.0846	77.26695	18.16772
0	0.3019085	0.01876938	0.1300433	0.03810716	0.04962884	0.1006109	0.08566147	0	0.02444263	0.09825394	0.04408638	0	0.02403458	0.02352715	0.03611632	0.04123761	0.06571236
0.3629547	0.2226379	0.2860515	0.6009639	1.606472	2.818049	1.89813	0.6597731	0.9990389	1.003383	1.424966	0.08669557	1.254209	0.3899266	0.9715858	1.260649	0.5423127	0.1615286
0.08078625	0.1059918	0.2266759	0.2447089	0.2568649	0.7387491	0.3284921	0.1403425	0.09381995	0.1613254	2.880271	0.06191008	0.3092865	0.6986566	0.1453714	0.507178	0.138983	0.03383572
4.664555	5.101483	9.911955	7.374571	15.03645	27.93646	35.3558	6.659377	31.7842	8.609836	14.79621	4.960858	11.44755	11.52318	7.649538	20.42404	17.35134	5.759288
1.514948	6.995864	1.114023	1.695247	2.922759	3.739201	3.9197	2.15134	3.681686	1.589861	4.397063	1.015601	9.928489	1.924837	1.527126	4.348413	2.260382	1.092304
0.02971381	0.09356305	0.1221514	0.217626	0.2893355	0.4921677	0.2728237	0.06194287	0.07764233	0.0681741	0.5024154	0.1229634	0.3469617	0.134072	0.1312414	0.2686233	0.07028865	0.02715281
10.12309	14.39202	15.65793	11.6866	53.42071	104.8844	101.496	15.50215	67.77567	22.20321	34.2176	14.96419	49.57888	24.90905	31.81857	51.45878	39.60727	10.113
5.489665	10.37679	4.107511	9.686179	7.518732	11.56257	10.23193	13.88887	19.29317	11.11284	39.28495	5.715858	29.84852	9.076469	13.91072	12.94766	7.831382	16.68678
1.832364	7.429041	0.7056798	1.279987	0.6449664	1.038341	6.135903	2.728455	2.089742	0.5037592	2.199887	0.3056248	0.765703	2.60884	0.8404784	1.946594	0.5614501	0.2435045
1.357545	18.92422	74.35493	72.23075	13.82597	11.67478	39.47121	96.8182	5.583669	32.15633	91.22198	5.494332	20.65385	25.9124	28.48828	20.58765	6.690212	14.21452
5.49264	6.540393	11.84732	4.055387	7.991875	60.30882	4.408854	4.56505	4.406503	6.357371	23.92487	7.341518	19.60226	26.97687	16.60412	15.78842	3.119939	10.64136
1.862618	4.228919	3.146277	1.403492	4.156467	2.974396	3.026499	2.655482	1.623622	8.419054	30.41897	0.885802	12.23472	9.807268	5.98055	3.835653	3.683555	1.976708
0.2266503	1.954119	0.9507587	3.820642	2.080444	6.550203	2.052725	0.6910516	1.770448	0.2751412	6.940192	1.091779	4.142332	1.515069	5.256996	1.900609	0.998021	0.1849247
0.05998807	0.2768228	0.01012343	0.02805599	0.1027671	0.05888903	0.1790757	0.1416869	0.03903714	0.02900335	0.07949116	0.009511347	0.01781857	0.03888977	0.04822037	0.04675115	0.1334511	0.01417701
59.46897	54.17539	48.57629	68.14094	155.2229	272.1967	658.5356	73.24374	315.1685	100.7469	117.4512	76.10293	259.4386	130.9326	90.01475	252.9248	165.1352	61.6041
0.2088738	0.2448896	0.1130968	0.2411043	1.713307	3.243472	2.68728	0.5095442	1.458016	0.5891259	0.8368242	0.1123891	1.016594	0.8745065	1.134122	1.397806	0.6642096	0.1015273
1.822491	1.842694	3.409483	4.641356	7.736268	8.32278	5.13193	2.249013	6.059926	3.203929	4.997428	1.447909	5.585061	1.271354	3.644644	3.831376	4.36268	2.50194
0.01593116	0.2508208	1.746451	0.6986453	0.04748825	0.1924107	0.05572397	0.600959	0.06167143	0.6024276	1.918254	0.06714816	0.3456198	0.1663962	0.5081957	0.160026	0.09135881	1.049395
0.5001562	0.07720076	0.5375443	1.170515	0.5651723	0.2791921	0.4888155	0.7593532	0.4413315	0.5125166	3.127992	0.5072981	0.5256383	0.221251	0.276741	0.5171752	0.3585243	0.1792347
0.2119328	0.185371	0.1920725	0.2448617	0.7565253	0.06094393	0.09266221	0.2571355	1.97128	0.9104673	1.478027	0.2075283	0.3606111	0.02951432	0.885997	1.493138	0.0337597	0.7621138
0	0	0.08728276	0.1319426	0.02416483	0	0.1169671	0.1689966	0	0	0.1453797	0.04659403	0.0310362	0.06096401	0.009946153	0.007634125	0.008716643	0.01852003
0.01992704	0.03361415	0.111454	0.1042479	0.01414269	0.01105124	0.0112019	0.1292854	0.02686123	0.05624256	0.05469741	0.06381085	0.02724634	0.03746375	0.02444854	0.05227494	0.007652247	0.02926536
0.01027634	0.161791	0.06537963	0.06968946	0.04084282	0.03324476	0.0471772	0	0.193932	0.3536639	0.1777063	0.23035	0.1278631	0.2704798	0.05043226	0.7596651	0.03314849	0.7747272
0.2174343	0.05902232	0.3229041	0.3762622	0.7524348	0.4560084	1.003128	0.2177057	0.3755061	0.2484804	0.3169384	0.112044	0.4592758	0.5450488	0.2437733	0.624867	0.2700717	0.06423296
0.03304743	0.20812	0.1358557	0.06275156	0.1379127	0.1282933	0.7889236	0.06889231	0.2782489	0.1853442	0.2455254	0.3115063	0.0442822	0.5467501	0.1135288	0.3547781	0.2771638	0.4378872
0.009884205	0.03890432	0.05321024	0.06032718	0.01964215	0.03837142	0.01296485	0.09566655	0.0143486	0.0377965	0.07596678	0.01136206	0.0709523	0.05574828	0.02425392	0.08842591	0.02656966	0.01693556
0.04775228	0.0653751	0.04064312	0.07321472	0.08045411	0.1880655	0.1225475	0.09274543	0.1145296	0.1190878	0.2127585	0.06921169	0.2921104	0.1118953	0.1604785	0.1603224	0.2232392	0.0403164
0.9314935	0.7657602	0.4241318	0.8116103	2.513028	11.09257	6.167075	0.9788195	2.511878	1.397733	1.155433	1.060602	2.683099	0.8608468	1.381549	4.608027	1.445313	0.9023896
0.1773785	0.6516192	0.740765	1.154785	1.409965	1.683245	2.737665	0.4490089	1.150144	0.331605	1.931304	0.5845119	1.312899	1.030082	0.5585738	1.18597	0.8519002	0.1688443
2.903514	4.867591	4.131336	4.156844	5.983641	10.40198	37.13251	3.442735	13.19121	4.420572	8.60936	7.447875	8.362708	6.537009	5.640354	7.240697	7.833821	11.23952
1.874646	2.263649	2.522297	3.81765	4.428657	3.649511	3.104482	2.602026	5.434706	5.434538	7.352715	1.004366	12.21042	18.60978	3.751931	11.87843	3.603267	1.004346
0.02959525	0.1863795	0.1390446	0.3130945	0.1646749	0.3446747	0.1319857	0.07932308	0.1288876	0.08299145	0.5989766	0.1020607	0.3569078	0.1112809	0.09440729	0.1839417	0.1909315	0.1014168
2.496026	5.464122	2.552894	3.813311	16.19022	20.7723	20.55429	1.492933	14.18828	4.732098	7.175904	1.46685	9.10631	8.062736	6.800034	12.43925	9.178512	1.113219
0.9912424	1.095688	1.215077	1.34188	3.504192	8.192723	11.61286	2.215393	9.39827	3.011255	4.570538	0.3113486	18.79281	7.244511	3.433858	5.521032	1.533002	0.7842033
49.22148	48.7851	57.54578	80.32161	143.1159	264.2866	644.4086	62.12183	249.7833	99.36512	108.748	65.07566	226.0116	132.0594	78.42317	281.2414	140.1536	54.74528
0.1080717	0.05401538	0.1175331	0.07677928	0.2812371	0.2264208	0.2160073	0.04086922	0.2116694	0.1180738	0.1977628	0.07493248	0.08866017	0.3203574	0.1641633	0.09046353	0.3578304	0.02155413
0.03509424	0.552525	2.181224	1.880147	0.6450969	1.226153	1.496046	2.377674	0.3056715	0.8946527	2.11283	0.2622192	0.9404969	1.319576	0.6028011	0.8096851	0.4905502	0.5010862

TCGA-76-4926-01	TCGA-32-2634-01	TCGA-16-0846-01	TCGA-HT-A74K-01	TCGA-76-4925-01	TCGA-DH-5142-01	TCGA-28-5216-01	TCGA-15-0742-01	TCGA-06-2559-01	TCGA-41-2571-01	TCGA-DB-A64S-01	TCGA-32-1982-01	TCGA-19-1390-01	TCGA-DU-8165-01	TCGA-27-1831-01	TCGA-VM-A8CD-01	TCGA-DU-7006-01	TCGA-P5-A5EX-01
1.581551	0.6941565	0.621643	0.2046157	0.1247861	0.1959011	1.549411	0.2877821	0.9082685	0.3298651	0.1818005	0.3952557	0.4097618	2.524781	4.226387	0.2956285	1.121457	2.525949
0.20486	0.1665688	0.1330552	0	0.9323208	0.03394354	0.6923563	1.047137	0.8169334	0.3242813	0.2165649	1.757794	0.4906209	0.1602134	0.1663339	0.2045134	0.2192256	0.5124045
3.968193	2.05718	2.919268	0.7470663	4.043376	1.79317	2.531356	1.220986	2.68273	0.6515122	1.419264	5.118098	0.6854898	2.081626	9.087905	2.600893	3.004865	2.158229
1.850743	1.008524	0.4139495	0.2885472	0.8725567	0.8001393	2.639238	0.8927513	2.775091	2.483385	0.6374783	1.832914	0.9686617	0.642223	5.698949	0.2272371	0.9181243	2.608176
12.95234	14.38231	41.13915	29.25037	18.65639	16.78509	32.93875	25.5741	14.55061	17.46495	19.7025	59.85386	10.7059	9.035912	65.59325	18.44915	26.90859	30.68557
2.607796	5.746133	6.21979	1.797391	4.245158	6.019488	9.113266	4.365393	4.147771	4.387125	5.964109	6.998702	4.07214	5.546146	5.693266	8.145401	21.19348	4.553278
2.65148	4.110864	8.53868	4.007002	3.847398	2.601205	3.907992	4.639288	2.471018	3.257558	4.479833	3.707295	2.739394	3.247159	2.64965	8.751285	3.367479	6.942571
6.743498	6.11691	7.50191	2.337082	1.734429	4.233613	4.200359	6.565568	3.703736	4.075437	4.161338	6.867181	3.655948	7.08781	4.460473	4.193552	6.070483	4.334409
0.3617641	0.3137551	0.3916056	0.1205198	0.1763993	0.09990202	0.3493252	0.2439849	2.110514	0.2999602	0.2039648	0.5173508	0.1031419	0.4125951	2.464072	0.3783497	0.1613052	0.1740115
3.900572	2.659785	6.532745	1.509704	2.169081	8.94932	4.586399	2.329796	4.075062	2.646161	6.249091	8.090514	2.080473	7.413726	3.932096	5.51844	9.558797	8.722466
0.09427635	0.2989538	0.1408333	0.1067861	0.1792829	0.1359008	0.1980006	0.150591	0.2882298	0.0319787	0.05979778	0.1213403	0.02419106	0.4479101	0.06889201	0.07260442	0.1172816	0.1360431
63.81956	78.65729	113.4006	46.06123	50.05173	313.6964	44.04876	38.53898	62.91226	57.91739	144.4824	199.3636	35.32288	180.1791	144.8086	105.7035	166.735	183.8346
0.8495317	0.5920651	1.403445	0.5206724	0.4791895	1.745033	1.21762	0.6014246	0.9836622	0.8273379	1.44192	2.340293	0.3979982	1.496608	1.768883	0.7396024	1.986092	1.274589
0.2769727	0.2942648	0.7675389	0.2255538	0.1950784	0.6455475	0.8023478	0.1769674	0.7854236	0.2672344	0.5348442	0.3281909	0.3758837	0.7689659	0.4197853	0.5846086	0.4989318	1.140416
1.347493	0.9417739	3.546269	0.5943019	1.224007	5.130415	1.333459	0.9362684	1.545565	1.115126	4.255267	2.752885	0.8566424	4.368514	0.9569989	2.635909	4.709461	2.809592
0.2878903	1.208381	1.968612	0.4632593	0.4555659	0.3510071	2.911043	0.3748274	1.523498	1.650887	0.8406657	0.4993733	1.644695	0.9876793	0.4586782	0.5902999	0.2441376	0.595715
0.1040367	0.1078533	0.1013568	0.08664823	0.1268231	0.1680705	0.1977797	0.09970899	0.3025123	0.1588025	0.2062144	0.1673782	0.1001083	0.4424129	0.1478251	0.06120363	0.621025	0.2927488
4.37524	3.176023	10.23993	1.992084	1.946553	9.980307	4.787973	2.534508	8.250725	3.548101	11.1967	10.54551	3.284823	10.4825	7.740808	7.282331	11.31533	4.564736
344.4596	333.7166	968.5745	208.6951	126.456	1639.294	425.1776	325.8718	229.2991	204.5759	459.1585	801.9494	137.3161	844.5474	685.1292	313.0844	941.4704	1115.676
1.110768	1.011274	2.997518	0.3491989	0.9497426	6.543958	2.277335	0.8246608	2.131558	1.15155	1.428926	3.969614	0.8349885	3.110769	7.373791	1.298538	4.896944	2.433647
46.43329	28.16232	70.24542	19.4318	25.10912	143.9615	52.74492	25.19551	45.60017	28.64969	34.75538	94.72639	11.73678	82.63792	49.1722	31.72731	73.57397	91.51335
0.07021834	0.0727943	0.1482206	0.08772327	0.05991838	0.07417043	0.06992283	0.05047304	0.1458407	0.05359091	0.1169128	0.1613856	0.06306243	0.2007836	0.2209255	0.06384064	0.2465608	0.2279853
2.490333	3.265972	8.385392	1.207047	2.297661	4.764626	3.752047	3.104321	5.108227	1.56357	3.55249	6.563095	2.483379	5.822743	5.399926	4.247294	5.367161	3.669711
13.4662	12.2857	23.30954	23.3577	7.259855	23.36511	15.0025	12.04428	15.624	13.19575	19.54222	43.01019	6.662038	14.4807	61.78674	17.71267	14.46125	22.80758
2.04049	4.186773	3.64843	3.818755	2.393758	4.725328	3.889952	3.079125	4.168214	2.760407	6.299931	8.132557	2.928992	5.146428	1.934504	3.964519	4.616821	5.490583
0.6579317	1.131853	2.305291	0.6921679	0.7513785	1.781513	1.604992	0.652688	1.82967	2.067273	0.9444479	2.610449	1.661579	1.462389	1.377675	0.9111505	1.869024	1.009375
18.44004	19.33358	38.41424	13.21649	8.481029	64.60657	19.90538	15.2247	16.22658	13.07798	27.3147	50.71659	9.284235	41.32333	63.20962	13.17505	50.3038	72.38732
6.109879	6.744985	12.13984	8.993009	4.579908	27.82614	13.37998	5.845217	9.931932	8.882518	26.47535	15.31839	9.510306	14.14762	3.195383	7.145043	19.80722	16.93675
1.098898	0.9068347	0.2840704	0.03642707	1.370238	0.09239779	1.385783	0.4261647	1.467983	1.669021	0.1179024	0.703661	2.222126	0.1859912	0.4261437	0.2666576	0.04826691	0.2777012
0.6151396	0.6460686	1.486147	0.2767215	0.7030616	1.495742	0.6866812	0.7059591	1.083292	0.3349135	0.7554921	1.123836	0.3579119	1.801744	1.075262	0.6537722	1.880489	2.315836
0.06793692	0.07954355	0.1235488	0.04828336	0.04858573	0.2296339	0.1246419	0.05208869	0.04816292	0.06145152	0.06320036	0.216517	0.06043243	0.8713476	0.3088997	0.0658842	0.2071977	0.3137109
0.8511658	0.9582525	2.615295	0.7525284	0.5347578	0.8136398	0.4504641	0.5020055	1.316359	1.165972	1.13167	2.377618	0.982831	2.112207	3.21625	0.4622134	1.944321	0.6708153
5.444425	6.074262	19.50149	3.364247	4.992107	16.92178	10.10815	4.402503	9.770915	11.58206	16.39064	19.28583	6.213241	10.33019	16.70067	13.57842	14.45234	8.721922
0.5781807	0.7779097	1.154244	0.1946454	0.2848936	1.863554	0.7316676	0.4588581	0.7685496	0.437657	0.8338272	1.812839	0.224882	1.813455	0.7906487	0.6145253	1.670558	1.306823
2.925922	3.306112	7.862687	6.538567	2.323912	9.765976	4.034087	3.623308	3.154206	4.501961	9.068838	12.09071	2.982495	7.206826	12.0498	4.017949	6.915858	9.831435
3.539753	4.072818	2.667761	2.375188	2.37327	3.021064	2.279367	4.261969	3.02955	1.665108	3.791425	6.324242	1.563803	5.871234	2.548699	3.264684	4.492637	3.739406
5.132432	4.270963	14.30715	3.867783	4.861409	16.36287	6.707635	3.475292	5.089287	5.194775	10.91123	17.83241	2.857973	6.027749	19.38956	5.729512	6.164678	5.931792
3.884457	2.637656	5.429423	1.716403	0.9830429	1.79168	3.701735	2.1794	4.567913	5.146122	3.171024	3.688129	5.026816	2.197792	1.355824	4.208242	2.516993	2.292103
0.5173129	0.504744	0.9635977	0.5202719	0.5044926	2.217251	0.7280822	0.2681651	0.7849483	0.5164878	1.176287	0.9511684	0.6511828	0.9274606	0.9430787	0.6180547	1.167237	1.064812
9.052278	11.29756	20.91478	3.968931	7.147205	22.83097	10.11842	4.233623	12.58327	7.264883	12.80472	22.21273	7.131525	28.01029	29.80038	11.56884	23.97145	15.82489
4.715379	7.374133	9.386547	1.42896	3.856733	11.82607	5.56247	3.814897	6.26794	2.661571	6.787065	8.717352	3.108164	8.906221	8.302655	7.702796	9.207724	9.045034
1.750137	1.775759	4.386711	1.538373	1.394258	7.278158	2.433781	1.385967	2.140705	2.307708	4.732173	3.958201	1.471635	5.098955	1.754574	2.703377	5.466916	3.998101
2.020091	1.862264	3.755137	1.21601	2.289308	3.988841	3.248812	1.771382	3.111147	3.01496	3.92345	5.558617	2.094849	2.94994	2.895786	3.802869	4.752119	2.026456
1.76523	2.923011	5.545116	1.413647	1.258007	5.332198	1.745509	1.431523	2.323878	2.809608	4.209465	3.99468	1.567915	2.85728	2.191233	2.570745	4.556795	4.418534
0.1498834	0.1637908	0.2102723	0.07190334	0.07016109	0.1430461	0.1823596	0.1608864	0.2629879	0.1220177	0.1825312	0.6614073	0.1538387	0.147695	0.3504852	0.1282541	0.2983326	0.3806624
0.2861273	0.1570362	0.6155877	0.09532181	0.2354369	0.8800267	0.725259	0.1942427	0.4397637	0.3518239	1.474569	1.939972	0.0734195	0.734244	1.873064	0.2677905	0.4162294	0.3922443
0.0479302	0.04676567	0.1556517	0.03193539	0.02337119	0	0.06942326	0.03062426	0.03185573	0.2601281	0.121605	0	0.02459752	0.05622666	0.03891641	0.1025338	0	0.02766578
0.2220745	0.1944554	0.9541762	0.106232	0.1166151	0.2434187	0.2886675	0.2328469	0.4919895	0.2704084	0.16614	0.6281879	0.1519566	0.6145469	0.7767245	0.5700826	0.6078288	0.2958083
40.29575	55.34717	88.69307	86.4005	60.8982	154.87	99.8065	38.91012	44.13957	55.76779	161.9303	89.52261	38.5871	94.71747	101.1911	51.04311	83.35528	115.8571
8.269796	12.74068	11.07636	12.59446	8.363046	8.325038	11.37423	13.83572	9.228298	10.62649	9.626818	14.02989	9.893191	12.50565	15.91184	7.284533	13.59806	10.99087
10.53837	14.33432	25.47881	9.362139	10.3787	41.74902	18.49397	7.724207	12.85294	17.08056	24.65432	26.08425	12.57915	24.43197	11.12558	19.67842	26.06115	18.35068
3.782046	2.792891	10.82673	1.777764	4.073569	15.82512	7.935605	2.275794	5.500764	4.999703	6.868035	6.76342	3.762205	7.931356	5.531631	7.4811	9.64689	6.810767
120.5918	64.53563	414.565	163.3848	73.33084	284.9319	174.7285	77.32568	117.2704	90.74268	253.7999	263.5793	55.28665	151.0402	410.6418	127.4729	208.6153	267.753
9.435721	9.939541	7.221247	2.317348	4.877856	3.948581	4.981403	7.757442	5.040435	5.197555	2.407387	10.13063	1.58214	6.128291	6.23209	6.211757	11.83943	6.038877
3.958443	2.895749	2.244227	0.3533948	1.163808	6.447657	2.067451	1.086429	3.644366	0.9947964	2.681451	3.700089	1.296927	7.84613	2.19123	1.741994	6.498021	3.19204
1.684404	1.737777	3.64072	1.508667	0.9155812	3.376548	1.439842	1.305583	2.037125	1.760891	3.345277	4.721756	1.218701	3.984324	7.734979	0.8624365	3.279998	3.506492
6.276331	2.075587	7.627616	2.553625	15.01908	17.53032	15.02399	6.986898	5.44505	5.772594	3.612975	26.82995	3.735254	1.711783	68.60329	5.084779	2.553946	5.215963
0.07580496	0.06009507	0.1969393	0.01894052	0.05082435	0.5652105	0.1578342	0.0181629	0.3904609	0.03214146	0.1021736	0.3658736	0.02917702	1.645138	0.453924	0.06081163	1.308072	0.6891471
344.108	329.0739	494.0888	104.4555	111.4513	1437.116	352.1362	204.4481	284.0245	185.6566	306.6037	971.7141	111.2153	941.7644	2342.321	218.2487	1042.501	1295.816
0.2992791	0.252699	0.1270945	0.06135585	0.5275971	0.0629386	0.2917675	0.5442408	0.7497344	0.2576944	0.5037725	4.719674	0.6734262	0.4321018	0.1401903	0.1723689	0.5219717	1.023193
1.772845	1.363225	4.18909	0.3881179	0.7409614	2.929061	1.669091	1.278369	2.766157	1.122524	4.771024	5.960558	1.481698	5.134916	4.715889	2.07686	5.03765	2.631353
1.305774	1.251298	3.240922	1.36718	0.7504046	3.569867	1.688674	0.8194059	1.162304	1.312944	2.514266	4.158684	1.112872	2.338725	1.987892	1.745846	3.209516	2.570675
3.707132	4.40729	9.733669	6.238672	3.024489	11.58655	12.1982	3.340461	10.08301	11.13181	9.923351	13.31761	5.649994	8.033337	16.03889	5.059688	6.75392	7.076899
56.08119	61.0923	136.5534	76.83739	34.95227	135.8932	87.35826	49.51224	51.71059	83.97563	84.30245	179.6264	34.02431	87.13093	279.4452	56.53137	93.47948	145.7578
0.0207829	0.06083383	0.02699671	0.1107794	0.06080353	0	0.09030734	0.1062313	0.0828773	0	0.02636443	0	0.04266268	0.0243803	0.0337489	0	0	0.04798437
0.9348881	0.7377142	3.11897	0.5446174	0.5231176	1.396666	1.006336	0.5940682	1.086518	1.67742	0.4795706	2.667663	1.211247	1.018804	0.7051492	1.066636	1.312061	0.7253997
0.201378	0.4129038	0.5686682	0.01944581	0.09677059	0.1508751	0.3846805	0.1864745	0.3491514	0.08315729	0.1036653	1.341551	0.09585728	0.794299	0.4597146	0.1348573	0.1475703	0.2459516
8.68297	11.07009	19.78097	8.912043	7.730146	35.35734	14.13053	6.400985	9.197596	9.958967	29.81856	24.65016	8.773474	23.72646	8.898722	12.02992	24.76589	18.89819
2.306534	2.415365	3.808302	2.323055	1.647892	4.81006	6.885638	2.50839	2.317261	2.418787	2.733063	5.68568	1.335457	3.016336	1.861332	10.83719	5.004366	3.641311
0.1932219	0.1445376	0.7613445	0.07724503	0.1004978	0.2241065	0.102618	0.1069953	0.1797888	0.06117178	0.1797505	1.634249	0.04627487	0.6648915	0.2300972	0.08266927	0.2688028	0.7955777
50.4168	38.9883	110.4859	26.35544	31.0606	78.06452	68.6668	35.94075	45.43387	44.40905	49.50378	70.03096	25.1204	60.30589	84.10523	45.57466	61.81537	53.28843
7.613404	17.90938	23.6202	10.45874	14.24983	5.95081	15.75535	15.11335	12.75236	17.04068	16.91886	20.51569	9.243538	8.644849	21.36404	12.42596	6.574919	20.43823
0.9007965	1.187922	2.151407	0.6019209	0.7746761	4.715059	1.052812	0.8359574	1.135276	1.00383	3.135064	3.064336	0.8179895	2.049488	1.315241	1.421657	1.762638	1.771122
25.44122	13.39848	36.31277	16.25683	48.57521	113.2671	49.41408	41.08863	42.21121	27.28234	17.26454	86.00396	15.85632	10.10414	194.5216	17.46835	7.183533	23.31895
9.822954	9.944803	40.3275	10.15385	10.68454	5.975198	22.68758	11.49621	32.34652	8.255172	8.121031	18.62984	3.660372	16.69893	48.04941	38.35724	48.63639	69.31535
5.254332	17.47072	5.548275	0.8903304	20.54766	4.768125	6.124345	3.939572	6.914567	2.913807	3.844291	4.001282	5.192161	6.544519	3.061123	5.904951	4.247733	17.18892
1.076147	1.164131	11.33515	1.558751	1.562809	3.872393	1.897569	1.569493	1.539314	1.618833	1.469032	11.82907	0.5642789	9.042786	5.698475	1.651526	5.614104	11.53202
0.02466075	0.08312184	0.09901416	0.02389993	0.04372654	0.05126168	0.06169685	0.04870224	0.06258082	0.02129265	0.03412771	0.1236628	0.04602097	0.2603646	0.08009214	0.05946934	0.05577901	0.1526966
126.0559	111.7086	464.4057	159.8232	96.6272	417.7715	213.7068	128.9848	141.7616	155.1075	355.8521	316.1304	114.2209	200.0321	282.3626	212.4767	251.9773	193.116
1.17523	0.8036129	2.577715	0.1668781	0.6716925	3.725322	1.765018	0.5231644	1.075599	0.888771	1.020379	1.275262	0.6723323	4.22072	1.384394	2.052485	3.742	3.202721
4.777944	3.942834	5.406155	2.696512	2.615466	2.420031	6.964234	2.045487	4.88176	4.810837	3.172321	5.353228	4.941229	5.257769	2.550301	4.25385	6.622328	5.452994
0.5985582	0.168466	0.3214743	0.1073727	0.1515438	0.01716507	1.34213	0.8457797	2.907133	1.366561	0.05110735	1.206374	2.197496	0.1012739	0.3364566	0.1181958	0.1339573	0.2790526
0.2657188	0.425191	0.5246511	1.062274	0.2487683	0.4015293	0.2463191	0.5704505	0.353208	0.2884233	0.4584305	1.211653	0.2618217	0.6857693	0.4660147	0.5184119	0.2047335	0.8711729
0.4763971	0.124506	0.0442024	0.1587092	1.078514	0.01691463	1.589522	0.3152575	0.6332533	0.2423921	0.4532551	0.3441184	0.1397054	0.7085533	0.3177331	0.7789017	0.03413867	2.052538
0.008786013	0	0.03423874	0	0	0.008734603	0.01272587	0.01122737	0.09343081	0	0.03343685	0.03230918	0.06312509	0.06184095	0.01426742	0.02255438	0.007051594	0.03042824
0.06016254	0.0587008	0.01603084	0.0164454	0.04663637	0.02760485	0.03351572	0.01577023	0.1045779	0.02720965	0.1213293	0.02836388	0.07125022	0.07781495	0.03006051	0.03960049	0.01114293	0.04986356
0.1893364	0.2879711	0.1519075	0.5565566	0.3475654	0.1107226	0.3468321	0.5692873	0.36271	0.02266702	0.5439489	0.4300397	0.3429403	0.4311537	2.007528	0.03335577	0.0983271	0.6107205
0.3981741	0.1942499	0.3852783	0.08662832	0.166417	1.547024	0.5178755	0.1505675	0.7020994	0.2315337	0.9586767	0.8366987	0.3753199	2.130529	0.771945	0.2572743	1.884821	1.26172
0.1862464	0.1956998	0.05583037	0.124094	0.02794319	0.5483484	0.4980254	0.06407652	0.2190033	0.03887698	0.209004	0.05268399	0.02205707	1.680651	0.2326473	0.03677761	0.5461772	2.621426
0.03213733	0.0679391	0.01391532	0	0.02089392	0.07987321	0.04654848	0.0273782	0.1495155	0.03633679	0.05435767	0.1378766	0.05497569	0.03141677	0.02609356	0.03666623	0.0515864	0.03091664
0.3195225	0.08123303	0.4910515	0.03898068	0.05705424	0.279624	0.1368857	0.06038356	0.1525427	0.04579545	0.1712682	0.7447143	0.06235754	0.3880283	0.668679	0.4370757	0.4551023	0.7143475
1.635722	1.832883	7.847581	2.290418	1.557798	2.09621	3.812962	0.9226444	2.399365	2.097988	6.091311	2.525877	1.731353	1.738964	5.021904	1.101697	3.930765	2.574297
0.6151737	1.469306	3.937245	0.6746004	0.5061894	1.885687	2.088357	0.5732063	1.013635	0.6433929	0.7397419	2.709933	0.644562	3.239939	1.092621	1.019899	1.91322	5.363253
7.954439	8.471905	16.88055	9.007989	7.444743	16.27926	17.38761	4.356314	8.404559	8.104361	13.71302	23.14381	1.854175	8.750239	79.11098	5.983784	22.42647	48.90746
6.724489	6.47924	6.041334	2.140396	3.378281	2.12709	10.2434	6.770255	9.424524	8.180548	12.41552	12.27597	5.173382	9.364911	5.450144	2.179581	9.264418	5.334387
0.3143366	0.1877751	0.4166521	0.08548537	0.1313771	0.2806098	0.1115003	0.1311611	0.119381	0.1827832	0.0732409	0.7312985	0.1185178	0.5418317	0.1666757	0.1262538	0.3552576	0.5924512
8.978001	7.896114	14.30273	3.134004	4.068364	24.30991	8.700487	5.078483	9.772489	6.959335	14.29068	26.21096	4.687405	21.75493	41.07838	5.656958	27.09068	19.98312
3.41453	3.88252	8.566335	2.330753	3.785162	3.554651	5.208473	3.323939	8.79575	9.401242	10.55346	12.60137	8.953039	2.48943	4.333205	4.556244	4.198249	2.706311
149.8636	98.77504	411.7705	167.7016	95.09527	364.8339	216.794	122.4617	133.0983	149.6316	368.8858	328.7668	97.13711	194.258	283.9203	216.4983	258.7348	198.3964
0.100395	0.1251656	0.1328267	0.03220731	0.08884146	0.2587602	0.1400287	0.2209467	0.2471305	0.1185589	0.2594317	0.2871452	0.2976835	0.7371703	0.1086861	0.1002251	0.4386932	0.1051666
0.3993668	0.7978826	2.890302	0.3547919	0.4822003	1.58812	1.514995	0.8991678	1.693697	1.419167	0.361873	4.47576	0.8588503	2.208619	2.347036	0.9601132	1.480539	2.393001

TCGA-DU-A7T8-01	TCGA-DU-6403-01	TCGA-FG-A710-01	TCGA-S9-A89Z-01	TCGA-19-1787-01	TCGA-FG-A6J3-01	TCGA-HT-A74O-01	TCGA-DU-5847-01	TCGA-DU-7007-01	TCGA-DH-A66B-01	TCGA-06-2558-01	TCGA-26-5139-01	TCGA-06-0125-01	TCGA-14-2554-01	TCGA-02-0047-01	TCGA-DU-6396-01	TCGA-32-5222-01	TCGA-19-2620-01
0.2052803	0.03898189	0.06250043	0.2224999	0.7781002	0.6180281	0.31451	1.374654	1.261915	0.1694745	0.4905991	0.910126	0.4856344	3.336913	1.668825	0.3282027	0.6514522	0.5606117
0.7088628	0.03250529	0.09621484	0.04497767	0.1827672	0.398978	0	0.1428366	0.1163115	0	2.108594	0.1704228	0.2832432	1.19517	0.4300099	0.4802535	3.570359	0.5853532
1.094911	1.881947	1.176837	1.637569	1.933655	1.417806	1.291664	3.876896	1.403272	1.17027	2.819316	4.842207	1.71116	4.831372	3.049341	2.295072	3.452534	3.050516
0.2506081	0.3772836	0.2264201	1.463889	0.7654354	1.501188	0.3178413	1.121206	4.397965	0.5997333	2.864774	2.569452	0.982878	5.367412	3.081533	0.8788953	3.141595	2.544869
26.02549	17.27423	25.43106	19.14609	31.24284	36.81882	21.85378	26.91045	15.3259	20.26706	22.76999	39.94869	19.24333	33.35226	17.86837	28.46109	39.78396	32.09642
6.155361	4.483282	5.075133	6.234132	7.006215	7.260383	4.483733	6.112982	5.63799	4.279808	6.588878	5.615508	4.595838	4.663496	6.392689	9.735237	6.711256	2.669621
4.624945	1.536756	5.008561	7.024075	7.810948	8.224453	9.159103	8.430155	8.610593	3.962015	3.553866	5.61379	6.393316	5.666992	6.067083	2.169728	2.339006	4.473542
2.009387	3.762623	3.336541	4.165083	9.187025	6.818461	5.091105	8.184298	3.576253	4.787465	5.148532	6.553115	5.655686	6.290418	5.032851	4.222491	3.15703	7.293297
1.232821	0.06696828	0.4719629	0.05295099	0.3012336	0.3914216	0.07931115	1.631129	0.179721	0.07763892	0.6335266	0.6269817	0.1979886	0.6155806	0.4640523	0.166291	1.82567	0.2871335
4.729422	4.808696	4.868043	6.572982	5.212845	5.562315	6.144652	9.16181	5.684534	5.582164	4.161409	4.656771	2.804058	6.023858	6.991545	11.18507	6.018003	3.506365
0.4359458	0.02692602	0.4073574	0.01862881	0.1917692	0.1407671	0.02657395	0.5639914	0.317144	0.08844641	0.5761588	0.2058746	0.332388	0.1856305	0.3166242	0.08580473	0.1842236	0.2514203
81.36617	106.4126	101.827	147.9792	147.0667	205.3305	146.0072	120.0035	106.6843	160.4593	115.1943	175.5113	86.75602	188.8382	178.7203	281.2735	62.19464	162.7557
0.9218183	0.9102242	1.231732	1.883371	1.349988	0.8801035	1.808882	1.691539	1.207508	1.515913	1.485279	1.337113	0.8103362	2.331332	1.586451	1.981354	1.710235	0.9303605
4.068799	0.3838079	0.2948557	0.4013477	0.4349014	0.6632894	0.2255387	1.102051	0.6106706	0.5638466	0.5780868	0.6605182	0.3669914	0.6194214	0.7708636	1.015975	0.2275429	0.3634592
1.743604	2.623315	2.833682	2.308022	3.338807	3.403976	3.318726	3.79542	2.129671	3.976557	2.483731	3.112504	1.228833	1.784145	3.026816	5.100149	2.274593	1.231833
0.897063	0.1241111	2.081739	0.3291548	1.174695	0.804001	0.2143545	0.3454049	0.3700819	0.6355	1.684279	0.5422545	0.5069409	0.9388198	2.059921	0.3955029	0.9524235	0.3104146
0.1227242	0.198091	0.6107756	0.21414	0.2506061	0.6838365	0.1906134	0.2611389	0.3211821	0.1865944	0.3680995	0.1541637	0.114625	0.1707072	0.4367551	0.1990601	0.2747246	0.1362476
4.307738	3.936824	3.164399	7.577539	7.848878	4.680159	5.331867	9.016132	6.05721	5.71676	4.838197	6.9257	3.628699	5.983694	8.179106	17.38612	9.967793	4.050861
240.6074	465.6978	500.9339	839.5925	847.7018	691.6642	817.5095	659.9507	975.0307	1017.146	502.4663	442.7676	247.1291	700.3439	897.2689	960.4408	368.8436	320.7353
0.9153568	0.9346025	1.113173	2.163941	2.952002	1.670712	1.234742	4.76464	3.124385	4.308182	4.453425	1.971734	1.123436	3.581001	2.539529	4.815305	3.842013	1.020693
24.58129	30.90715	16.71106	75.89972	66.97743	128.5568	53.67425	73.23484	81.96251	112.1337	70.43627	69.17946	27.23327	60.58379	70.73726	139.8796	76.15712	26.1062
0.07454813	0.1128087	0.09481419	0.2139062	0.1221593	0.1253585	0.1632893	0.1101579	0.1794025	0.08234514	0.1355151	0.0657164	0.1228734	0.06720979	0.2026631	0.2759688	0.2085998	0.07523894
2.421551	3.72829	6.714956	4.509167	5.63022	2.171442	3.983263	7.617513	3.353885	2.985764	4.982028	3.524946	3.114052	4.593667	6.313777	8.084442	5.650285	2.041697
17.03853	15.89479	20.06735	28.59385	40.02399	53.33501	34.55386	22.00065	11.59849	22.22457	23.39919	16.36988	9.609786	29.22482	17.86355	30.94984	29.49938	13.719
5.943901	2.553943	4.873438	5.095297	3.970694	3.860999	4.205425	6.382116	4.873409	4.017397	5.215868	4.661834	3.625058	3.003451	5.437826	7.994607	3.331187	3.623968
0.8251298	1.96976	1.024596	1.049171	1.288263	2.337928	2.030223	2.035341	1.253351	0.6879522	1.938038	1.663604	0.7540674	1.903431	1.841369	2.327911	2.816364	1.022418
17.05276	19.60859	24.87375	49.09773	29.2165	71.03792	47.79094	30.21476	62.199	62.42138	25.80843	22.24395	11.37022	42.43473	33.65692	43.38108	29.25307	17.77584
13.34442	8.409927	19.28195	16.76547	14.97781	20.31096	21.33368	32.60169	18.054	21.05493	13.71598	20.4406	6.228586	11.8516	26.767	49.49237	11.80105	9.250079
0.4333854	0.6662227	0.2875837	0.1368378	0.6262786	0.2058543	0.02465668	0.2927554	0.1350256	0.01810261	1.596736	1.794234	0.657632	1.495117	0.5890906	2.098916	2.171301	1.22889
1.364376	0.3879319	1.177712	0.9496925	0.9270194	0.9309486	1.223678	3.353616	1.615013	1.09409	1.058618	0.8947199	0.6581921	0.9771986	1.644847	1.322665	0.9849673	0.6567897
0.04274135	0.03018289	0.02552588	0.1252925	0.0775813	0.02940256	0.07149174	0.2311575	0.3240041	0.3499219	0.2214338	0.16955	0.1080205	0.2031292	0.1204199	0.2248442	0.02870362	0.08627484
0.3938988	1.885577	1.62979	1.229071	2.120404	2.954107	3.02669	1.294215	1.031571	0.8581495	2.116522	1.491062	0.4243405	1.423482	1.282711	0.7787436	3.829617	0.9354093
6.177766	18.12137	14.79631	12.5128	17.5559	15.80997	13.50374	15.25301	7.360904	11.02783	12.77141	12.96105	5.153905	13.29967	9.271287	24.72403	26.03297	7.722914
0.5398853	1.541238	0.6059835	0.8017356	1.76576	0.118531	0.9881338	1.736433	1.212865	0.7456267	1.558256	0.5619966	0.5995525	1.131782	1.533001	2.054546	1.99284	0.4637347
5.826018	4.259689	5.931225	16.64931	6.157271	14.24279	9.409383	7.756474	8.922656	7.968062	5.420378	4.579569	3.013223	9.172966	6.176812	9.076331	7.221867	4.730819
3.017447	2.261193	2.99169	3.524734	3.972843	6.373603	2.908566	9.433338	2.161411	3.487639	3.114949	2.661705	3.584026	3.438318	4.597079	4.444997	2.411767	3.188535
6.290948	4.947981	9.520113	10.60009	12.62523	17.45617	9.604714	14.16608	6.898127	12.26714	8.022778	7.869436	3.56645	14.37137	6.150215	14.19919	10.88598	4.629891
2.652031	7.347729	3.89743	2.253389	3.640866	2.855552	2.680624	3.552512	1.504499	1.393529	2.882068	3.144654	1.705187	3.213332	4.189027	6.128929	9.566489	4.111888
0.5710877	1.050036	1.12771	1.755097	1.097209	2.369843	1.51319	1.294406	0.972568	1.637493	0.9920883	1.035281	0.5819811	0.8167945	1.529768	2.002834	1.139228	0.5298975
6.320124	15.18503	14.86848	18.84043	16.52978	9.140823	18.09225	15.11637	12.07694	15.79935	20.13957	14.14072	7.300429	21.30987	19.46356	32.41683	29.15303	8.016194
4.034324	6.571754	8.665133	6.588353	8.256564	4.637233	5.22305	15.84341	4.734224	6.558431	8.925499	8.278389	6.867491	9.82199	9.155499	16.3726	10.64999	4.553777
3.326468	3.762579	4.024961	4.616392	3.971666	6.71566	6.42469	6.025707	3.299369	5.513845	3.092175	2.911875	1.84039	3.145824	4.173776	8.379057	3.514943	2.13646
3.056747	3.22961	3.356644	3.129093	3.430467	5.38134	3.974081	7.162893	2.847546	2.383379	3.325007	4.337459	2.094761	4.152822	4.659409	7.250523	4.8561	2.414982
2.442707	2.843994	3.204637	2.303012	5.151615	4.616921	4.411153	7.13621	2.414021	4.046506	3.887171	1.980348	2.07689	3.537014	2.553327	3.735643	3.971115	1.875277
0.1471027	0.1883544	0.1689467	0.1500577	0.1540449	0.03113681	0.1554729	0.351136	0.2144468	0.1124918	0.2005547	0.1571067	0.2300271	0.1901891	0.1510136	0.1885007	0.3672928	0.1941476
0.3375227	0.1872752	0.2183723	1.342781	0.2967519	0.2554069	0.2318731	0.6059796	0.9823262	0.3947552	0.6787458	0.3124131	0.08344791	1.388902	0.513007	0.525271	0.8027847	0.2214228
0.0452317	0.06844609	0.2025987	0.0315697	0.102627	0.06223143	0.02702045	0.1002566	0.04081933	0.02645074	0.1233346	0.02990482	0.02485094	0.0524304	0	0.05948621	0.2531338	0.0913017
0.1988243	0.449946	0.1497639	0.9526405	0.1036348	0.155258	0.2824881	0.30015	0.1260852	0.07541781	0.4835305	0.2486933	0.3247584	0.3550446	0.438255	0.1460534	0.8420406	0.5098021
72.38154	41.88901	126.2366	81.33452	86.62167	312.4654	101.8941	144.597	107.0336	94.80863	63.51197	69.19325	34.77403	106.346	78.44764	143.667	57.06405	36.74496
6.413873	8.878682	9.184529	8.165896	21.85115	22.34984	10.60913	14.37923	9.2623	7.309694	13.70679	10.12	8.640336	13.31091	8.499421	10.19465	11.81801	6.380643
15.48481	19.67912	18.22137	27.29862	34.39277	26.60876	29.96884	49.49678	18.99357	24.34552	21.32571	13.46525	10.1643	20.69885	30.42978	52.29733	24.18094	10.32469
6.802096	5.46872	10.23187	5.587871	9.803605	3.178383	6.70301	12.11031	7.931028	7.01914	6.899062	8.0812	3.948706	6.729944	5.781821	16.2036	6.279534	2.787995
132.4087	139.5203	162.8397	386.732	186.8609	481.0218	212.0152	98.16387	217.3381	274.8236	140.2166	156.9694	43.68725	309.9598	131.355	479.7568	195.6834	101.7331
4.423947	1.732803	1.934992	6.33342	4.94326	7.552183	2.239097	9.644884	37.44255	3.846509	2.9	11.54107	10.99888	18.87222	4.323998	6.044316	2.500027	12.17528
1.604643	1.681917	2.190657	2.481399	1.86633	1.012719	1.011345	6.699015	3.802949	3.396204	4.174721	2.370005	1.455876	3.297705	2.903354	7.931512	3.291364	1.552652
1.576542	1.656171	3.216275	3.182846	1.655492	2.043581	2.210488	1.698117	3.809677	1.996256	3.783658	1.808996	1.889836	3.549182	3.110588	2.159057	3.777088	1.578001
15.72822	2.477123	2.51013	2.755978	11.80124	33.41791	4.360874	8.340352	2.799857	7.664666	17.91466	19.87594	4.794651	53.34799	4.821962	4.494816	45.94181	8.77422
0.1564874	0.0811892	0.1290597	0.09361814	0.2485399	0.05536313	0.2884594	1.066335	0.9966238	0.6065884	0.4327945	0.1773621	0.1326493	0.5908217	0.2651964	0.4351273	0.1801569	0.0361
160.7373	292.1348	326.3954	882.7559	615.5104	679.5192	542.5846	664.9563	1353.504	1112.372	619.7958	386.5656	210.3655	1080.61	803.874	902.1962	614.8765	258.8628
0.7875438	0.2027323	0.1243414	0.2122864	0.3943442	0.2092337	0.1427608	0.2118797	1.328309	0.05081846	2.206658	0.2441821	0.608747	0.1196191	0.4349075	0.2666717	1.690008	0.2740834
2.071374	2.166	3.029214	2.874771	2.277585	1.139949	1.389689	4.99384	2.020294	2.208302	3.551772	1.991019	1.930295	3.227548	4.500975	2.965139	4.409493	3.626333
1.540323	2.419662	3.110164	3.440244	2.421443	3.405909	3.483447	2.926409	1.73758	2.41917	2.640028	2.269533	1.571654	2.639942	2.82245	1.919636	3.485036	1.088218
4.995964	5.027501	7.681856	11.46706	4.68785	21.85199	12.22747	11.83389	5.787166	8.888215	7.777107	7.575695	4.206045	10.37208	13.60944	11.2013	12.78339	4.277141
58.62836	98.09329	92.55344	113.5381	130.1629	198.1637	158.4734	105.1187	78.86552	102.3166	88.01034	66.96358	33.48515	163.4774	89.47461	163.8415	173.0335	72.50663
0.05883842	0	0	0.05475543	0.02224992	0	0.04686507	0.06955522	0.123897	0	0.1069576	0	0.04310222	0	0.05816559	0	0.02195214	0.0395891
0.6701268	1.366668	0.9933307	1.110411	2.072861	1.71131	1.284478	0.7309174	0.4959868	1.263034	0.9136275	1.644713	0.7893297	2.162686	1.651394	0.9130154	1.651204	1.294285
0.385589	0.3000785	0.4715268	0.349861	0.4999257	0.5191395	0.3290609	0.6544274	0.7630589	0.8149707	0.5970434	0.5244299	0.4781715	0.5842356	0.8045616	0.4467355	0.3730089	0.2223784
11.89057	16.30502	20.72015	24.90996	26.045	15.76322	25.70195	18.84686	17.35636	25.99913	15.9381	14.35521	7.963472	15.10428	20.47784	36.88576	15.1472	8.755221
2.187306	3.115518	2.655979	3.509605	3.171861	4.647528	3.537321	3.501535	2.302522	2.850387	3.119787	3.72515	2.488165	3.222416	6.534172	4.695337	4.506708	3.277833
0.1397966	0.3556405	0.1088987	0.322411	0.4688851	0.443212	0.1888086	0.4311106	0.4772116	0.1492839	0.2071671	0.3214823	0.08682445	0.2677273	0.2343357	0.5542226	0.1700771	0.1349576
23.37362	54.47812	67.55533	54.98334	75.52268	90.47198	55.45857	77.94979	48.29634	61.66012	66.33011	73.32929	24.82748	100.8884	67.85562	75.93081	105.7507	39.84126
13.14078	7.867941	11.38965	24.40618	19.10893	35.36082	11.86678	11.60528	8.347429	11.2573	8.10859	17.17289	12.24542	24.44069	12.84289	15.71093	16.5419	14.33045
2.089681	0.5783107	1.336265	2.123635	1.717545	4.776036	1.299551	4.252786	1.72002	3.317908	1.673327	1.797845	0.7187405	1.397128	2.346712	3.952126	0.2632324	0.5241706
52.47238	11.87688	8.637658	14.17288	31.66719	88.62996	19.97565	44.47056	22.37161	25.81326	89.08854	52.93682	24.69701	195.1782	23.41976	30.48457	141.7769	22.53807
21.85882	6.209274	14.56667	27.16348	32.2757	65.30105	11.32834	26.30733	321.1154	9.267925	14.65251	15.15033	24.7712	61.71701	28.58329	15.67531	24.02373	23.27875
5.593523	1.89913	6.737583	4.457256	6.156583	3.606233	3.357593	12.2903	7.69374	7.600725	3.450741	23.96945	6.725325	8.493598	7.179079	3.312897	3.276532	7.308964
1.501262	1.893132	2.010712	4.283707	5.798121	14.1395	7.029498	6.586619	6.515057	2.633737	2.302614	4.554077	0.5458332	3.672313	1.685975	3.910038	2.261027	1.893967
0.09520502	0.01494032	0.1431982	0.05906562	0.09840564	0.03201894	0.07330354	0.2869913	0.05155064	0.02721854	0.1874877	0.06434331	0.1069387	0.04169044	0.1505861	0.0241142	0.03788824	0.02989381
177.8819	245.4652	258.455	379.9477	369.6731	280.0158	359.8657	246.21	232.3774	351.664	204.8483	218.1517	72.75552	329.7075	226.4529	707.6856	258.6132	125.9539
1.263605	1.274753	2.36619	0.8248357	2.495748	1.069374	1.341353	4.029922	1.825365	2.200857	1.54924	2.289918	0.7391937	1.870403	1.893954	3.550802	1.516073	0.5688454
2.377028	9.672499	4.936268	3.700059	6.919438	2.956694	7.273594	6.059648	2.901351	1.960065	5.432347	6.617912	3.326003	7.122906	5.570318	5.297686	16.67788	4.452922
0.2607042	0.2301285	0.2432767	0.4018279	0.2341419	0.2092337	0.08435866	0.02407724	0.2548784	0.02540923	0.09626362	0.3519096	0.22082	1.063981	0.2335614	0.7285851	1.690008	1.129223
0.5115477	0.1416637	0.6589329	0.5880615	0.625846	0.7728067	0.4074501	0.4624335	0.3439716	0.527902	0.9025516	0.4509446	0.2645192	0.6045881	0.3272164	0.5100656	0.7185104	0.2632057
5.459133	0.3077605	0.2956636	0.08965247	0.009107593	0.04418162	0.02877502	0.5907761	0.3405145	0.2441254	0.4049754	0.4989315	1.420269	0.4280681	0.3690395	0.05631018	1.401769	0.9560987
0.03316542	0	0.06602323	0.01157399	0.009406202	0.03422265	0	0.02940461	0.1197205	0.01939458	0.02260829	0.01096361	0.02733233	0.03844375	0.02458962	0.07269552	0.1206441	0.03347275
0.1557683	0.06021346	0.005796108	0.05486766	0.009909124	0.02403496	0.008696506	0.05808149	0.1064151	0.02213418	0.1587806	0.01539974	0.03519232	0.006749871	0.04533263	0.05998949	0.03910602	0.08815611
0.1261247	0.005301555	0.04184659	0.7555853	0.1788541	0.5711928	0.08162285	0.5218393	0.4268294	0.2458519	0.1934482	0.7921764	0.3637971	1.139123	0.467559	0.9445496	0.1176402	0.2439791
0.4294362	0.5686073	0.3015012	0.6369202	0.7264168	0.174085	0.196983	0.9008583	1.076128	0.8744602	1.014132	0.5475612	0.2317252	0.8844514	1.142807	4.282843	0.5536145	0.3250617
0.3109611	0.1363932	0.2758756	2.302478	0.1533794	0.2697199	0.99342	0.3236473	2.135203	0.9329428	0.1566784	0.1698361	0.04456864	0.2429127	0.250602	0.4623011	0.06809698	0.122808
0.04549197	0	0.04024978	0.02116757	0.04587446	0	0	0.3630005	0.05017737	0.02955882	0.07580486	0.1938289	0.07775891	0.0820277	0.1648964	0.1063618	0.07920587	0.08162405
0.1380257	0.04498632	0.08031777	0.3883073	0.4552997	0.1928224	0.04566662	0.9300444	0.2702022	0.2086176	0.2692414	0.2470914	0.1026666	0.3938284	0.2424565	0.3518768	0.2543125	0.2143151
2.402877	4.428423	3.147966	5.214372	6.296741	7.362722	3.115795	2.370974	2.658219	3.014839	1.668827	3.017848	0.616192	4.31599	4.005693	2.038026	2.146186	2.781156
0.7740521	0.7991825	1.938195	1.688292	1.955212	2.679063	2.413161	3.131144	1.86642	1.428685	1.228453	1.031521	0.4717897	1.507089	1.452686	2.094302	1.644764	0.8666714
6.489221	5.742029	9.989753	13.16534	16.90708	28.63804	10.11838	12.28687	10.59574	17.30166	15.33261	10.43496	3.897627	33.46649	9.010923	12.5121	25.9445	9.60202
3.523612	1.831553	6.080022	5.779212	7.430148	5.890645	4.304464	13.3236	8.66684	4.326161	6.292198	10.14117	7.780981	10.0176	14.03112	18.67012	14.40186	7.592025
0.2542622	0.07939449	0.1626963	0.2366181	0.4258072	0.333165	0.1952881	0.7406987	0.3168717	0.1062059	0.1898333	0.2081297	0.1197388	0.2947285	0.09874648	0.3343915	0.1693985	0.1221992
5.733213	12.67804	11.73088	18.44355	13.75663	15.56874	15.82007	11.84466	18.62488	19.81419	15.88223	10.78597	5.228179	21.99428	17.26348	24.92271	28.70214	7.389436
3.131873	4.933325	3.368192	3.875016	7.845804	11.37053	4.663494	13.35322	3.267325	4.308671	3.858109	6.7995	4.217184	5.416485	9.291031	16.27812	6.935497	8.818664
154.6243	242.1017	234.0465	384.4606	311.0445	370.6703	307.2871	182.0018	245.5226	363.1132	200.32	230.0162	69.69427	348.1702	217.4118	684.1143	298.4453	118.095
0.2561561	0.09203852	0.1851139	0.127354	0.1293761	0.03862235	0.07965535	0.3235528	0.1979178	0.1969917	0.2415935	0.1832767	0.1966445	0.1138886	0.3200024	0.7568312	0.1413909	0.1345771
0.8255515	0.4526274	0.7859959	0.9018749	1.079073	2.543258	1.608152	2.816369	2.008311	0.7136579	1.46808	0.8068513	0.3352472	2.26753	0.6386957	0.9283686	2.591273	0.5796185

TCGA-HW-A5KK-01	TCGA-06-0158-01	TCGA-06-5858-01	TCGA-HT-8110-01	TCGA-DU-A5TU-01	TCGA-FG-5964-01	TCGA-06-0174-01	TCGA-14-0736-02	TCGA-HT-8018-01	TCGA-06-0157-01	TCGA-06-0686-01	TCGA-06-0743-01	TCGA-32-2616-01	TCGA-28-5208-01	TCGA-HT-7693-01	TCGA-P5-A72U-01	TCGA-02-2483-01	TCGA-06-5408-01
1.399579	0.8810092	0.7229435	0.2777366	0.205455	0.0430819	0.3093855	10.52113	0.6191463	1.705291	0.7809096	0.5818195	2.427457	0.7982672	0.926559	1.208602	0.8266042	1.112633
0.1358019	0.7788901	0.4096457	1.756908	1.271244	0.2155446	0.6538976	0.8647005	0.548772	1.022113	0.6667943	0.3320839	0.9235664	0.2290374	0.8337919	0.7059247	3.615837	0.6155
1.749909	2.620986	1.850365	3.15613	1.49459	0.9822392	1.9256	6.785186	1.771185	2.162009	1.933828	3.289374	2.243908	5.423015	1.182711	1.397809	1.535884	4.399175
0.7068663	1.6183	2.887573	1.112299	0.7748339	0.1332569	1.398614	4.11287	0.8052606	0.9104861	1.316491	1.668934	1.127225	0.8404565	4.537365	0.08891539	2.548393	2.19649
23.50165	33.50521	15.38996	17.51202	27.57993	29.07683	17.73994	41.97085	46.48365	23.51098	31.47881	25.8503	18.62368	35.1644	12.65689	66.90481	28.45315	63.27719
6.559657	4.112337	6.418373	7.971361	5.00898	3.138108	5.937072	8.216282	3.57493	5.135434	6.698711	4.556158	4.361979	13.27414	3.469268	3.5556	8.668854	5.166108
3.256516	5.958013	4.067867	3.549264	5.519177	2.624224	3.264425	6.370673	3.59366	4.018801	4.296155	7.219432	5.94791	5.205627	2.648163	3.4116	2.375969	9.442209
5.084321	6.925911	8.637658	8.472185	5.213542	3.123972	2.956695	6.77711	3.105088	6.151225	4.498394	8.300659	5.968957	9.367589	2.643669	5.671579	3.426864	5.52162
0.2597983	0.760665	0.0876845	0.8743519	0.1987673	0.623814	0.2886809	2.523763	0.6120515	0.4122436	0.8585924	0.6475164	0.7248597	0.2584046	0.1813827	0.3389874	0.9471439	0.3729618
7.885639	5.919208	5.57852	11.96681	6.629101	4.175484	2.242633	8.671379	4.350647	4.86409	4.167175	4.435825	5.273487	5.705939	5.214037	3.772365	4.293219	3.299666
0.03281035	0.2346182	0.05655557	0.714444	0.01645383	0.02479836	0.1015615	0.5770047	0.4944552	0.3501683	0.1611005	0.4126266	0.157259	0.2055355	0.07507373	0.1077187	0.1198083	0.9347333
219.0015	229.9946	127.821	263.4466	164.9769	37.01627	57.95041	237.0897	97.81408	210.2927	51.30393	102.0668	50.81068	164.6254	56.20455	214.4924	41.72201	129.3954
2.448844	1.789427	1.640104	2.569958	1.935562	1.040218	0.6661	4.39169	0.6435219	1.18477	0.932145	1.317136	0.8914258	1.300588	1.445704	1.095155	1.291511	0.6434649
0.2631679	0.4786668	0.4095096	0.5067427	0.6194604	0.2525627	0.4162524	1.734141	0.3410344	0.5254657	0.3305546	0.6547634	0.23586	0.4300845	0.9018358	0.2344182	0.3096143	0.7016268
4.18436	3.428713	2.897758	6.30641	4.180885	1.856847	1.387942	2.816948	2.147717	2.504157	1.796363	2.017789	2.139851	2.824286	2.103781	1.989743	1.622685	1.530233
1.091045	0.9631523	1.374514	0.6301636	0.4297671	0.1828864	1.228844	1.180775	5.458837	0.5119168	1.021029	0.9773832	2.728019	0.3583048	0.8535656	0.1004845	5.930795	1.100165
0.5560389	0.3708319	0.09219767	0.4380055	0.1891384	0.4173272	0.09339677	0.4803009	0.7315615	0.2739552	0.4285747	0.2608751	0.3283188	0.2108211	0.2761536	0.176891	0.4200486	0.2206429
12.13039	6.789915	8.919601	18.57584	5.306251	3.362856	4.198704	6.638738	7.031791	6.050908	4.163297	4.51551	3.364039	6.172177	8.083673	3.551297	5.578875	4.572562
594.3235	797.8872	455.3827	1199.674	773.6107	653.9282	203.6143	1538.336	282.0037	948.0824	523.8215	694.8648	384.4288	543.2141	289.5325	602.1606	488.934	555.7478
1.555626	3.01735	2.606491	3.066405	2.894244	0.3744782	1.212519	5.637731	1.02565	2.515141	1.174373	2.120762	1.678623	2.759204	2.047274	1.034214	3.371572	1.264964
49.03193	78.04548	122.0332	66.81884	86.56716	6.415899	36.06159	128.0848	29.67342	65.33754	35.11801	35.17359	44.15686	100.1573	117.406	21.0348	71.95115	42.38173
0.1309157	0.1228689	0.1627494	0.2135081	0.02553134	0.1800841	0.0735432	0.1759798	0.1744861	0.14111	0.05356691	0.08536945	0.1187115	0.1275714	0.1584286	0.1432685	0.1533725	0.251303
6.576672	5.291162	6.532277	11.43023	3.979865	3.360045	2.805513	4.802327	3.692812	4.534206	4.410471	3.505573	4.744009	4.381084	2.965563	3.150945	3.777552	2.840123
27.2832	22.51306	15.54395	29.93915	26.29751	18.48674	17.31054	55.67247	14.1635	35.29346	21.24665	25.4929	22.78741	38.26895	15.2935	27.81086	20.20858	19.21835
4.991849	6.313282	5.704699	5.042109	4.922861	2.892019	3.581002	3.38178	6.236819	4.258579	4.043529	3.301612	2.38365	3.962094	4.173695	5.90368	3.46064	2.733329
1.506417	2.351886	2.341687	1.603662	0.7856604	1.439145	1.782295	2.594384	0.4833299	1.410957	2.092177	1.315616	1.545516	1.693771	1.397124	1.017396	3.300447	1.744191
41.97404	35.74503	27.80159	57.73123	47.88871	29.23488	9.950036	84.35965	19.33037	41.19991	28.76057	40.76467	20.95628	33.0782	21.31287	24.95718	42.2202	27.0514
17.39022	17.61373	12.01692	23.16387	19.98812	10.84601	7.189683	13.72725	16.20472	11.93436	10.69629	8.884169	7.173147	9.540247	12.06687	13.76229	17.86466	11.28466
0.217451	0.5839104	1.633886	0.8235076	0.3689456	0.4199178	2.113721	2.619183	2.17366	0.6910276	0.8941949	0.5184512	2.188695	0.9718692	1.172564	0.1903751	3.462307	1.229053
1.344105	1.751052	0.5584613	2.008792	0.9938104	1.393384	0.4314652	3.481246	1.097071	1.112099	1.214713	2.105466	1.512025	1.436803	0.3827241	1.142636	1.543548	1.134253
0.07205674	0.0610528	0.06421963	0.1694942	0.04215776	0.06671489	0.08673981	0.1911715	0.08812009	0.2309945	0.07739425	0.1872171	0.2613579	0.1367186	0.05770584	0.05914183	0.08153922	0.1777009
3.401709	2.83449	1.874606	3.506927	1.675971	1.980564	1.214749	2.038175	0.8723362	1.470026	1.558373	1.731339	1.372571	2.003892	1.216558	0.9262177	1.698791	0.8071181
21.3958	16.89782	16.20734	26.74295	14.2598	10.79147	8.370027	21.63627	9.215097	10.047	8.659956	7.551853	10.12822	14.23239	9.354666	4.755781	12.30944	6.315643
1.137727	1.779659	1.208151	3.376565	0.5877479	0.7364071	0.5536528	2.65882	0.8494864	1.133649	0.6091439	0.4513587	1.399333	2.170601	0.9628314	0.7285034	0.7412093	1.168132
8.228291	6.109632	5.384695	8.165258	10.58745	10.2406	3.466141	17.20954	5.026779	6.599647	7.216276	7.35899	6.083217	6.958407	5.401872	8.041851	7.666644	4.776625
4.694397	4.536136	4.234186	4.791838	6.138994	2.626386	2.193439	4.475272	5.134215	3.540526	2.669774	4.882072	2.916889	3.420822	2.459768	5.81869	2.289865	5.98449
13.38627	5.701217	10.74559	11.01101	10.8863	6.144964	4.561634	23.94208	6.926693	8.522709	6.696579	5.816702	5.914933	14.20689	7.22826	3.671968	8.577832	6.011747
5.674811	3.135739	4.186846	6.996937	2.41679	6.545716	2.571593	3.072914	4.082692	2.240916	5.157627	2.840995	3.364735	1.673411	4.670286	5.645194	5.143482	1.738341
1.543173	1.07283	1.053956	1.442829	1.436617	0.5494287	0.7301992	1.349265	1.011454	1.017266	0.7982121	0.5399341	0.6600544	0.8948303	1.295422	0.6744505	1.617688	0.6158397
19.30422	17.68435	17.50206	29.08618	11.27229	10.78758	7.9913	48.35284	11.89682	16.98896	11.30432	11.72767	13.19354	19.63326	13.58195	8.521291	17.5871	9.847521
8.149146	9.346086	7.909609	13.96519	7.808759	5.544998	4.963041	13.28899	9.402492	8.880517	4.495498	5.892744	6.396814	11.0489	5.206957	6.988832	6.925723	6.258625
6.24203	4.835106	4.064243	7.618097	6.390971	2.762126	1.642453	3.919038	2.700325	3.288735	2.336762	2.657211	3.093072	4.280828	3.371832	2.713626	2.775787	1.895069
5.139452	4.102692	5.060076	7.422958	3.274366	1.905577	2.242896	4.924888	1.909196	2.857616	3.502968	2.52372	2.820073	3.683693	2.29276	2.501415	3.688467	3.065451
4.987299	5.112834	3.073165	6.399607	4.532351	2.562713	2.031664	3.241866	2.426538	3.895538	2.672537	4.25096	3.259782	2.685182	4.397255	4.386624	2.606387	2.474822
0.113268	0.3170527	0.08500938	0.3253284	0.08370817	0.1135445	0.2368162	0.5946892	0.1977929	0.3323631	0.1463557	0.2915584	0.1189261	0.2480074	0.2482574	0.3196749	0.1587291	0.1716527
0.7112779	0.5470273	0.204804	0.6859675	0.603407	0.1034861	0.2568647	2.283354	1.104319	0.505602	0.2182757	0.4674441	0.4837284	0.3898731	0.3132905	0.2140577	0.6249659	0.3034119
0.04765948	0.09940011	0.104556	0.08968496	0.1673029	0.1765052	0.02868555	0.2528878	0.02027271	0.02657104	0.05850262	0.05827217	0.06482485	0.2143476	0	0	0.02537945	0.05082561
0.4756127	0.1535165	0.670761	1.47036	0.1656331	0.4433493	0.2317379	0.9013097	0.2504782	0.2272825	0.3614128	0.3530663	0.5288255	0.8021472	0.5924943	0.3965638	0.6512698	0.2837954
64.85315	60.05496	54.06684	60.76546	103.0659	59.60503	62.81387	185.602	83.28645	96.80984	77.12426	67.7566	36.63732	123.9675	65.5752	53.67581	122.063	72.56239
9.798601	11.21977	10.33885	9.668057	10.69746	6.529492	10.2564	16.58781	5.937474	9.045762	9.78858	12.38608	12.92045	14.8632	7.998362	7.993219	12.71729	14.06046
31.68233	29.71472	28.70174	43.74442	21.41689	17.55738	14.7414	22.92184	14.61788	25.35925	16.23586	15.16274	27.79742	22.69436	14.47131	17.61762	15.66853	14.65164
12.62791	8.38062	11.7679	8.470352	6.917188	3.679492	4.046395	9.731337	6.40962	7.862439	4.96409	3.747769	7.170564	10.9113	5.507702	3.389674	6.92689	5.953966
131.4828	108.6374	167.7007	211.3239	390.6472	165.8982	108.0798	545.3093	235.4516	161.2074	188.9582	142.4029	141.4536	196.8185	202.7245	126.3228	219.5331	138.1533
9.722647	9.414721	7.075268	5.930342	7.756862	2.197117	2.368635	10.52639	4.090922	6.44522	4.055723	4.652999	6.240097	7.272129	2.756469	9.166098	2.96229	17.25847
2.675762	3.328172	3.083942	3.356808	2.708979	0.5744689	1.419109	3.209974	1.794691	3.057081	1.589904	1.849162	2.665438	3.025988	2.368527	0.7935949	2.601968	2.890739
1.510147	2.963497	1.355311	4.895602	5.092356	3.529985	1.322088	9.032891	2.429307	2.648267	1.786316	5.807838	1.568549	3.380493	2.096266	1.081728	1.915406	1.537266
3.094218	4.995004	7.459284	12.71445	12.39605	3.070618	6.58667	48.42022	6.275995	7.231706	5.965522	9.896248	5.944426	16.17659	11.19987	8.321963	20.29398	8.743469
0.4287055	0.2554634	0.09818409	0.2038994	0.1488382	0.02492458	0.09073639	1.109888	0.2685251	0.6933952	0.08096023	0.4953681	0.1238844	0.4078662	0.2012156	0.3866676	0.1003484	0.1456964
421.0144	693.9427	451.0381	1223.409	722.8595	379.4428	179.5806	2871.35	221.0643	844.0325	403.517	728.5628	359.1295	660.6225	406.2746	316.1714	773.2433	487.6648
0.366263	1.032445	0.86001	1.674611	0.368306	0.0544999	1.102242	0.6680583	0.5501537	1.454913	0.5479409	0.363855	1.06382	0.3474691	1.069387	0.2129369	1.115391	0.7140559
4.696688	4.236865	2.071782	7.53495	3.369137	2.029635	1.192388	4.846175	3.831368	3.074795	1.246818	3.833488	1.712676	2.770187	1.819582	1.258552	1.604793	1.57557
3.025733	2.611268	2.657701	6.010104	3.282754	2.612822	1.660661	2.558951	1.716057	2.456026	2.533005	2.069449	2.775205	2.059475	1.609227	1.814199	2.469352	2.225337
9.997897	7.824595	11.90041	12.46252	13.54518	8.112723	6.313149	13.21566	5.575659	8.103672	13.24503	6.772545	7.985331	5.830478	8.857888	5.137451	17.81655	6.612305
123.88	96.79198	81.11831	146.1854	147.5872	109.8877	61.26911	341.7388	72.52994	101.1172	98.63116	90.11107	110.8548	119.0338	79.10963	88.80199	118.1417	68.58784
0.02066551	0.1508522	0.02266814	0.05833211	0.1692689	0.02186685	0	0.1754465	0.03516159	0.0230428	0.1014687	0.1516035	0	0.02323568	0	0.2035389	0	0.06611509
1.605224	2.600984	1.526754	1.529463	2.377615	0.9030213	0.9906209	1.929924	0.665521	1.297099	1.396763	1.167665	1.400307	1.370784	1.285526	1.539881	0.9900626	1.489754
0.6442523	0.3298657	0.31196	1.283339	1.090036	0.2149518	0.1991231	0.7822495	2.00471	0.5759864	0.3633534	0.4364355	0.4263036	0.3980817	0.4555167	1.248115	0.9365019	0.4796981
19.91559	21.3005	21.31941	29.00307	10.92897	13.80121	11.27554	21.6888	15.58478	22.57031	15.31801	11.40572	15.48065	22.34504	15.32842	21.45041	15.61177	11.87153
5.17229	3.358315	7.621011	8.036301	4.551465	3.828412	2.288915	8.303305	3.299577	2.604182	4.186868	2.369376	3.707404	3.520199	2.146962	4.06367	3.453723	2.135282
0.3394306	0.2137139	0.1685995	0.3856519	0.03746952	0.2100762	0.06938435	0.9243205	0.4140771	0.4713117	0.1100599	0.2897269	0.3890908	0.3096371	0.2940543	0.6518039	0.3205797	0.1912346
78.70073	67.30759	74.1407	103.5381	60.09108	56.28935	38.56266	142.6838	32.40571	66.02925	72.33145	59.37704	52.38991	67.99727	53.31597	32.8615	75.32817	51.45855
37.0608	13.5576	14.93147	12.97695	10.40486	8.724012	11.64563	17.04107	17.39361	9.684409	13.7736	12.56507	17.46334	12.15124	15.45669	16.89414	22.66564	18.61082
3.456351	1.703918	1.35909	1.816683	3.63661	0.6445986	0.6059206	2.410619	2.305786	2.210486	1.096325	1.843156	0.6507034	0.9722802	1.193463	1.861579	1.182139	0.9139209
10.05805	24.79146	31.05885	45.76917	48.53901	12.31979	39.44139	210.2431	25.82567	40.06848	32.92369	32.9058	26.83614	58.05272	42.76408	21.31374	72.1285	60.65195
24.85004	15.17776	33.85854	19.97046	61.77299	7.899045	6.41126	42.68661	25.0373	15.81317	12.27365	18.89033	13.40508	34.61139	4.327004	52.61752	14.06057	43.71151
9.789702	13.66789	4.83566	4.728317	5.036287	3.836222	2.210676	13.79842	5.777	4.10073	7.444356	4.96076	13.83844	4.583242	3.080759	9.789048	3.411434	10.59187
4.3268	2.66842	1.620307	3.764633	0.9799171	3.236829	1.260115	13.15799	4.116325	4.83751	1.570517	4.650327	3.005867	2.367074	2.546019	11.634	3.580014	1.835772
0.01783379	0.07903878	0.0855838	0.06082639	0.01565085	0.05661155	0.03756865	0.03312	0.1744753	0.14914	0.04925522	0.1035736	0.1293703	0.0927395	0.03808532	0.05854958	0.06647751	0.1188658
291.4196	215.5935	239.2178	352.4175	374.1698	214.1323	186.5491	603.343	255.406	233.5358	302.5156	172.9536	214.3815	261.3517	197.5042	156.8636	305.6293	167.1341
1.479897	2.831807	1.875474	1.973729	0.8238028	0.572653	0.8128978	2.703916	1.637916	2.638087	1.140514	1.428811	1.71108	1.852426	1.012563	1.200238	0.8008213	1.581995
7.988334	7.967082	8.064099	12.49493	3.521075	5.14154	4.395943	7.12615	4.087775	4.607679	5.367375	3.17249	6.851509	3.957415	5.82337	3.431105	5.814756	5.002521
0.1888544	0.1193578	0.4080339	0.942305	0.2879483	0.7629986	0.7302353	0.3158094	0.5306792	0.08295559	1.095882	0.2938829	0.1089766	0.2187768	0.6477432	0.2818282	1.627374	0.292946
0.3910426	0.5069764	0.1970793	0.3878174	0.667804	0.5032398	0.5343373	1.547783	0.9620521	1.131313	0.4410901	0.7494838	0.7283446	0.4990924	0.552971	1.630799	0.7766662	0.2592289
0.3129845	0.1058547	1.048502	0.3899937	0.128676	0.2058681	0.5600512	1.526085	8.103115	0.3489893	0.6022477	0.6102181	0.3835237	0.5611546	0.6142059	0.3425167	0.3693754	2.769445
0	0.009110443	0.01916601	0.05754005	0.01022268	0.009244256	0.01051661	0	0.1189171	0.02922418	0	0.03204535	0.007921964	0.01964587	0.03731434	0.009560723	0.1860908	0
0.127315	0.03839021	0.04711178	0.0534003	0.01974364	0.02110013	0.03508319	0.2181298	0.1513743	0.1180157	0.08284767	0.06189099	0.05563686	0.06381351	0.02948207	0.05035954	0.07841621	0.1014207
0.03876084	0.08084084	0.1154034	0.2969685	0.0194379	0.4921693	0.1399775	2.291756	0.09421447	0.8458726	0.5845473	0.7176504	0.08535817	1.799295	0.08868912	0.8362442	0.4776867	1.251885
0.3151241	0.5729721	0.819839	1.136576	0.8083809	0.5728381	0.4231069	2.863993	0.6186599	1.112687	0.6942902	0.4840887	0.3187185	0.7813125	0.4443326	0.1901141	0.9939441	0.4782371
0.06410576	0.1039897	0.0859443	0.1139315	0.3083822	2.698222	0.1371887	1.360616	0.7816939	0.8101099	1.381458	0.470284	0.1614714	1.665817	0.1597194	0.7950851	0.1213773	0.3798025
0.02662984	0.08331006	0.08763139	0.1653685	0.04985647	0.01127116	0.0833462	0.02260826	0.04530969	0.08907976	0.02615078	0.07163136	0.04829471	0.04191852	0.09099185	0.06994211	0.09642963	0.1135957
0.239407	0.3266546	0.1693449	0.3473581	0.1151964	0.08996567	0.05656089	0.8168083	0.178926	0.2195457	0.1043669	0.2626251	0.08724139	0.1937105	0.2245768	0.2301653	0.1167649	0.3674575
4.04397	4.316498	3.759853	5.861348	2.832484	1.939525	2.15294	5.414192	2.41607	3.524454	2.168105	2.645079	5.245544	4.882671	0.6682358	1.442735	2.787852	2.625515
1.580222	1.980115	0.9365721	1.744616	1.155663	1.220352	0.7670266	3.17814	1.897261	2.415654	1.783312	1.830823	1.583136	1.354059	1.190662	3.116972	2.069805	0.9241421
12.02361	6.797936	8.358196	11.8854	14.13696	6.867127	4.045655	41.63075	11.75703	6.945678	6.330427	11.33571	12.50532	22.28191	8.662214	6.880271	23.01912	4.974462
19.07621	10.51512	6.295099	13.86663	4.323456	2.560192	5.717532	6.615257	9.485772	4.565122	6.773973	8.321171	8.835379	6.533117	8.374257	2.282619	10.96764	8.17934
0.2360155	0.299336	0.09795729	0.2940867	0.1194241	0.1484915	0.05375029	0.6904745	0.2279191	0.4623187	0.09396068	0.3431654	0.225582	0.164959	0.4904055	0.3211113	0.3057133	0.1700639
17.32945	17.6026	16.4229	27.76178	19.30952	8.742685	5.939406	55.52027	8.440014	19.78214	14.16286	18.49994	8.489034	19.24259	9.766404	6.776318	16.35207	9.826549
11.94112	10.4781	8.522456	10.83099	4.613268	3.56259	4.997237	7.004047	3.90931	3.815204	6.07614	4.11659	5.372617	2.79419	6.971573	6.155707	4.185978	3.791569
270.9901	200.0372	240.9456	360.3312	406.8737	173.8361	167.2967	653.3141	250.0147	231.6019	312.1755	175.6457	201.6323	247.9786	230.1151	110.9906	278.3885	165.3947
0.2236884	0.1869982	0.1703363	0.3130913	0.1600748	0.0723769	0.1045923	0.2001091	0.1934442	0.2288075	0.12254	0.3390481	0.1341063	0.174601	0.1855538	0.03236958	0.08269332	0.1833472
1.172423	0.985985	0.5393068	1.690272	0.5753061	1.280598	0.614611	5.458461	1.463951	1.855515	2.971178	1.988397	1.337483	1.190666	0.8480587	3.124845	2.154961	0.5243235

TCGA-28-2510-01	TCGA-HT-A61C-01	TCGA-06-0745-01	TCGA-06-0187-01	TCGA-06-0125-02	TCGA-FG-6692-01	TCGA-DU-A5TP-01	TCGA-E1-A7YE-01	TCGA-28-5215-01	TCGA-26-5134-01	TCGA-DB-A75K-01	TCGA-06-0238-01	TCGA-S9-A7R7-01	TCGA-HT-7471-01	TCGA-HT-7601-01	TCGA-DU-6402-01	TCGA-06-5856-01	TCGA-DU-A7TD-01
0.4389312	0.4084624	4.645987	0.9436513	1.525964	0.8361769	0.03360767	0.2590266	2.008857	0.8967969	0.05435663	1.36647	0.7114614	0.2295476	0.1891238	2.064598	2.219335	0.639584
1.068629	0.113533	2.884637	1.381338	0.156909	0.3255072	0	0.1757215	0.2627605	0	0.4285331	1.019918	0.3835193	0.07753303	0	0.2828054	0.8245737	1.143195
1.623421	2.239689	5.082106	4.569991	2.268326	4.132085	2.834316	2.193217	3.0787	2.005412	1.009557	3.428191	1.878893	1.524898	2.618663	5.180561	2.351951	4.87339
1.18152	0.4392529	2.32579	8.089683	2.708357	0.3363828	2.965532	2.113163	2.900472	1.30719	0.2603317	2.374724	5.897019	0.06958092	0.8906797	1.130255	2.179642	0.8109848
36.57806	16.00793	40.51143	101.5168	37.99248	35.94184	43.95377	21.43354	24.89762	25.73014	23.78161	32.39925	21.30831	14.54998	22.7651	24.52313	15.87925	15.70764
4.338831	5.211085	9.105344	4.555598	5.979738	1.907162	10.11646	6.490119	9.499749	7.416405	2.788338	12.48592	6.886062	4.937186	10.37125	29.7822	7.10403	4.659064
2.414827	5.283541	4.214168	8.99258	6.280033	5.757631	9.872116	4.098816	3.666223	2.367648	3.240668	2.197697	4.033808	2.686296	3.192222	7.578066	4.286005	5.003316
3.25945	4.950122	7.9999	5.516401	5.32967	7.16754	3.031563	3.055312	5.484318	3.98933	1.993719	4.501029	4.626684	3.191654	5.412511	7.660203	5.835335	6.96503
0.7674215	0.1447975	1.222966	0.9873429	0.2655418	0.5312695	0.9237716	0.6981933	0.2513394	0.4593204	0.4753945	1.150692	0.04232877	0.3765198	0.04641458	0.4161738	0.6067176	0.3604963
3.462173	3.482991	5.827413	5.278762	4.592141	2.993137	14.73927	10.39068	8.172752	4.451624	3.683343	6.824074	8.015071	4.36404	10.12745	7.11139	5.677031	5.371786
0.8143911	0.08882119	0.1564562	0.6157116	0.2762009	0.1675015	0.3038908	0.3113373	0.312886	0.07181982	0.1911425	0.1056072	0.01985571	0.2622526	0.05225352	0.1756981	0.367394	0.1070984
61.86625	99.90699	64.90873	176.9808	351.3545	59.76211	155.7925	198.2455	177.3552	75.28364	34.73104	84.10915	402.3445	70.37532	387.9396	93.32608	78.95605	162.9254
0.7670951	0.6627412	1.542175	1.313878	0.9454197	0.4694733	2.395953	1.899184	1.563271	1.022249	0.8516264	2.446324	1.920132	0.8603766	1.875786	1.419574	1.33224	1.408761
0.3621625	0.5594138	0.4735784	0.6794444	0.5798563	0.2507173	1.230808	0.5200299	0.7549164	0.7971038	0.4011098	0.3294132	0.5833371	0.4717144	0.8983447	0.6245142	0.6655208	0.7176053
1.387863	1.641852	1.758995	2.021551	1.778708	0.9607878	10.78938	3.612312	3.242172	1.989947	1.980647	2.145021	4.642746	2.7814	6.715267	2.451228	2.59822	4.586372
0.7412401	1.709875	2.360158	1.274598	3.501033	0.1788945	1.614736	2.147929	1.489225	0.3034549	1.001659	1.352164	0.1601629	0.4193832	0.2157651	0.2204597	1.103123	1.123709
0.3418985	0.1729708	0.3139164	0.217967	0.1120573	0.3381272	0.2095952	1.059712	0.3815582	0.06604607	0.1381098	0.2508861	0.1643352	0.1624203	0.04204615	0.09694402	0.3069277	0.2565885
3.82213	5.946352	5.90412	6.425196	6.753888	4.664223	11.6081	12.35322	7.700744	4.191724	2.455604	6.551557	14.28482	3.485497	19.07259	6.860764	6.332976	6.420267
218.4575	412.6803	711.5495	488.7829	423.9125	279.0939	841.6667	695.7997	810.3917	606.7261	138.4319	834.6361	817.2075	222.7521	1141.449	1027.757	477.4776	679.7607
0.6267527	1.291862	2.533361	2.423237	1.289642	0.8372672	5.253014	5.693176	2.844403	1.747986	0.532426	2.91595	3.432231	1.407717	4.834178	1.778522	2.631882	2.4741
23.75484	40.89983	62.87402	58.79278	68.21168	16.62574	100.6815	134.1255	101.9054	40.22235	21.65371	94.64239	154.9492	30.0972	203.2976	36.7173	68.15777	51.14521
0.1043915	0.08755843	0.1191792	0.1369684	0.1563055	0.07607162	0.07859099	0.143048	0.1097661	0.133731	0.08897848	0.1201715	0.1910221	0.139521	0.1378386	0.1417676	0.1445283	0.1154542
3.808521	3.174315	5.44168	5.782628	4.230003	3.296755	12.25672	4.613594	5.481788	3.521484	4.238205	6.032063	3.668026	3.936375	6.479691	4.422703	4.851061	5.298957
10.49387	17.60623	45.75119	21.15023	10.38685	8.744101	28.00209	25.72272	17.89053	19.23816	20.57491	40.72808	25.23233	13.91975	26.11052	21.97616	17.22304	18.36451
5.289435	5.068802	4.844028	4.918445	3.26136	4.743647	11.15924	13.46669	5.793448	2.727902	5.023538	2.513557	7.937757	2.691556	7.645066	4.496203	3.462017	4.829778
0.6502789	2.571562	3.378353	1.541041	1.70077	0.8028106	4.960888	2.324231	1.857105	0.9232872	1.053108	3.103187	1.227665	2.162424	2.495074	1.233325	2.774924	1.77019
11.00983	25.11149	42.02152	26.38171	15.9655	17.60894	36.12177	42.00358	38.91541	30.62619	7.398888	42.75814	43.01164	15.66703	50.43869	39.01089	27.47516	34.43617
9.148419	9.026082	13.23989	13.86444	10.87747	8.791665	40.41299	62.82957	17.08151	7.465345	15.09853	8.002154	19.52473	8.195587	29.21826	18.45363	14.04311	15.03351
2.101265	1.108938	2.518447	1.16912	1.821552	0.3932797	1.931986	0.4595736	2.187857	0.6386155	0.2322465	1.177215	0.1688789	0.09931897	0.2222161	0.2354759	1.29033	0.7125977
1.19196	0.4516503	1.540838	0.9284404	0.7382442	0.9202492	1.959923	1.293905	1.673483	1.333212	0.8776011	1.799487	0.6656575	0.9786956	1.831412	1.820618	1.367736	1.175992
0.1644348	0.04016053	0.09110677	0.1570587	0.08845973	0.03532801	0.1135494	0.198131	0.1132797	0.09200784	0.0306089	0.4340637	0.09538877	0.1337023	0.07112545	0.05627139	0.2585347	0.04874316
1.178311	2.258786	2.024986	0.7095259	0.6770106	2.177616	1.807605	0.456312	2.070622	1.122264	1.098325	3.534748	1.470897	1.245193	1.708641	1.172759	1.464433	3.76789
6.525021	14.43455	12.29396	9.582362	7.574491	5.250511	26.73207	19.72898	13.71744	8.35889	7.2539	22.65557	13.65678	7.047373	21.91705	8.318225	12.99945	17.81885
0.7467019	0.7622779	0.6611041	0.9426944	0.8880264	0.596047	1.663897	0.9501213	1.697861	0.4636407	0.2409123	1.20444	1.247626	0.642649	1.450512	0.9174728	1.703653	1.455548
4.430917	6.029231	10.75002	5.7066	3.696595	5.132715	10.87549	12.29864	7.186589	3.574523	6.426328	8.972988	10.27762	6.313146	13.35642	6.412721	7.339938	7.057721
3.334103	3.507044	5.898744	4.35221	3.65918	4.786291	3.89704	8.750765	2.208534	2.946743	4.384713	3.080917	3.756874	2.630395	3.508973	4.245779	2.863902	3.988233
3.596782	6.394959	11.68304	8.898988	6.777759	1.927558	21.72047	6.998321	10.41936	7.370371	2.528844	13.21675	10.95032	5.673333	18.78481	7.564091	5.938466	5.234371
3.754069	2.996368	5.202422	3.189563	3.714169	5.003581	7.165897	3.388716	4.471372	2.895837	4.143511	3.352044	2.094425	2.300593	1.481419	4.668047	3.157586	5.162478
0.6598608	0.9358116	0.9326251	0.7615656	0.8186201	0.4991045	2.050884	2.528436	1.309833	0.6692085	1.149668	1.135892	2.048849	0.7702171	2.245113	0.5295422	1.896495	1.464798
8.044018	10.68263	18.08732	13.41806	14.18728	7.131273	14.0632	20.77169	17.94733	12.55563	6.611263	30.5155	18.43671	14.39111	31.82187	12.54963	18.75831	19.59345
4.739855	5.822372	5.297752	8.765881	9.01164	3.957883	22.68516	7.14503	9.647081	5.40243	4.229507	8.531239	8.993637	7.373335	16.69982	7.264262	10.99292	7.273107
2.093332	2.782265	2.648662	2.877804	1.939932	1.210608	8.421389	6.103487	4.058795	2.065608	2.978774	3.175428	6.16063	3.125274	8.983017	2.851598	3.278671	5.104281
1.909845	3.458962	5.149515	5.257899	3.593365	2.263947	16.22487	7.050544	4.275214	1.591973	3.994795	4.144279	4.156744	2.609983	5.134963	3.254122	4.588018	3.423508
1.657462	4.533982	4.088646	2.687909	3.256616	3.596738	9.696183	2.737117	4.049336	2.284357	2.187682	3.084387	4.027597	2.187332	3.527865	4.098154	2.13341	3.46058
0.1275979	0.1063233	0.3075317	0.1871128	0.1033206	0.0727451	0.1646237	0.06685357	0.1730211	0.1522418	0.150495	0.268636	0.09259716	0.2858998	0.08307424	0.3376792	0.1974403	0.2652601
0.3134357	0.4063386	1.142139	0.3927526	0.226003	0.2014859	1.323677	3.482033	1.109592	0.2724651	0.4402655	1.702742	0.326418	0.4771535	0.2973537	0.2221838	0.3828026	0.8553737
0.06000541	0.1328141	0.07231137	0.2216131	0.0275335	0.04154046	0	0.04111279	0.02305385	0	0.04627478	1.759865	0.1009467	0.0816303	0.04427624	0.05955008	0.07892262	0.1146294
0.3493103	0.3155724	0.6929878	0.2961916	0.7261716	0.1036372	0.07477868	0.2197933	0.3943946	0.4627052	0.1429363	0.9922255	0.7195618	0.1616312	0.6627751	0.3065698	0.331292	0.4357835
36.57069	58.87244	139.2528	105.9154	64.22368	22.12301	286.6057	162.5895	132.8624	102.7769	88.48241	79.31066	79.30457	105.7503	175.9403	145.9883	95.22185	53.55683
6.307642	12.5631	17.20147	12.16186	7.137851	11.86523	11.73965	7.773079	11.15085	12.95281	8.487377	14.60917	9.240367	8.411161	9.385485	17.8785	10.14613	9.132762
11.13452	19.16209	25.29975	24.20051	22.41537	6.819652	48.97374	25.17872	37.28906	19.25432	16.48503	24.6236	36.88214	13.735	36.42804	23.98807	21.22021	23.6539
4.183548	6.331074	4.32502	9.117743	12.20225	3.047725	19.80815	4.194001	7.525669	5.525524	5.177014	8.229773	9.483958	7.235356	18.16855	5.085154	8.317652	10.95789
75.65182	178.0639	241.9781	147.8961	87.09365	110.8419	194.7639	376.749	254.3769	133.8866	170.8345	449.6099	290.283	177.1143	439.3517	221.1103	178.291	155.2099
3.475419	3.905815	5.342965	19.96676	15.23776	12.36608	3.853216	4.027143	7.488897	4.066562	6.1436	2.98544	11.67848	2.419018	6.601646	10.57357	5.843075	10.5366
1.332914	1.12822	2.584936	2.677755	2.477796	0.5272845	6.467129	4.917488	4.884651	1.941041	0.7041011	2.708574	4.73659	2.152016	6.488339	2.144906	2.889769	3.035959
1.469219	2.540326	3.16612	1.70764	1.268991	1.914558	1.450508	1.421135	1.56723	1.121907	1.359633	6.014544	2.481349	2.602229	2.538055	1.864043	2.864503	1.964672
21.17356	7.17101	22.10313	11.42089	6.524125	7.054735	35.15632	15.66826	7.762732	7.232241	5.414563	21.42242	7.553539	4.471328	18.35175	10.67391	8.896917	14.96836
0.2728454	0.04201095	0.20967	0.2628724	0.1905144	0.2381594	0.0905	0.01625569	0.2916902	0.07218536	0.02744505	0.3774034	0.5122238	0.24207	0.3282467	1.181207	0.1612279	0.1359707
172.6384	265.0282	762.6722	426.8124	352.0074	156.3141	662.1133	650.3929	754.6791	396.3425	69.55555	1403.67	815.178	174.6267	1060.855	692.9471	551.0816	459.2497
1.059184	0.1786183	0.764105	1.676485	0.4430268	0.2045121	0.3504243	0.3505089	0.5259699	0.05845926	0.1000186	0.5874015	0.694965	0.1176241	0.06379928	0.0715066	0.7897398	0.4955211
1.654044	1.646869	2.610975	1.605253	1.474271	0.7243507	6.425142	5.655491	2.582516	1.757866	1.071797	3.535777	4.000523	1.988935	3.314378	2.541779	2.905294	4.769908
1.67853	1.8996	3.893088	3.261306	1.942228	1.07107	6.193854	2.340095	3.308539	1.006582	1.891013	3.003783	2.946551	2.131213	4.038711	1.371261	2.278091	3.485352
4.360803	9.779785	21.55692	10.63291	8.51068	5.472809	21.4135	14.18221	9.501346	5.75186	10.04435	12.75227	10.00341	6.526717	15.35255	5.830276	9.613515	6.71638
46.01727	77.41922	174.5518	78.3266	54.21579	81.12069	109.5082	105.2075	105.7216	83.08843	64.83755	243.9901	103.1372	74.25784	142.9704	96.47231	114.2636	89.81962
0.07805639	0	0.08361268	0.9369077	0.1671422	0.05403677	0.02481173	0	0.09996324	0	0.1003255	12.15772	0.02918082	0.02359701	0	0.1032854	0.1140715	0.02485207
0.9657543	1.217424	1.818938	1.46449	1.778416	0.2169535	0.8782578	0.6134855	0.992714	1.790226	0.4192399	1.882125	1.248099	0.2726197	1.349696	1.341373	1.603894	1.612758
0.5590306	0.3493668	0.384539	1.049179	0.3621336	0.6753616	0.2229944	0.6083268	0.9152607	0.1926889	0.1549747	1.594688	0.4015878	0.2617827	0.1995061	0.3674417	0.2402842	0.1710077
12.61944	12.60124	18.23496	16.75473	15.74856	6.103946	24.37668	27.09725	20.66299	13.85656	13.83766	20.34651	33.03103	16.51878	42.15493	18.21975	18.67785	16.84823
3.307196	2.381746	5.707647	2.366663	2.17434	4.696163	7.820538	4.002821	3.928332	4.820101	2.645598	2.639485	3.993822	2.13601	4.651628	7.261677	8.891311	3.748243
0.1370772	0.2712774	0.2591201	0.2307931	0.3921869	0.4688959	0.2076107	0.176788	0.6381702	0.2616829	0.09327418	0.5691695	0.3617316	0.2340108	0.7080168	0.645509	0.2474595	0.3388788
36.84208	56.71553	108.5953	69.34776	42.89485	34.61618	99.83524	119.0516	63.78389	64.85121	40.58063	140.5741	50.82496	44.14082	90.87552	67.85188	72.98737	59.03762
11.00308	14.01099	33.57643	24.10195	19.55098	17.06816	25.90771	18.61426	14.49995	15.5215	17.26745	13.20119	11.99748	5.636247	10.60796	11.49627	9.866295	10.47725
1.73548	2.529187	1.73369	1.296305	1.297384	0.8887025	8.727318	5.773876	1.718106	1.582081	1.50879	1.163183	4.443164	1.405939	5.822472	4.637983	0.6069848	1.427946
78.62903	25.00533	138.3626	64.76824	28.70117	19.44612	138.289	60.2618	26.65585	18.52495	21.27766	100.2995	24.66072	44.51242	56.17757	40.13964	43.67526	43.87434
17.17016	18.92989	16.75905	52.10924	54.37145	37.70908	7.02519	12.64923	41.53123	11.0506	29.81456	15.38746	99.28923	11.28044	30.13239	53.26757	33.7163	45.11615
6.858881	4.961671	4.084753	24.33287	19.61791	7.126521	11.13853	4.547911	10.3019	3.199296	3.805795	3.284759	13.36794	2.703844	4.884628	13.81922	3.740498	7.253245
1.713369	5.756542	3.611838	4.056311	3.440376	12.37832	2.010931	0.8628791	4.287193	2.910919	0.7001815	3.974611	2.38146	1.952325	6.180753	9.330221	1.926088	2.81149
0.08700747	0.01739429	0.07666527	0.09847441	0.04636266	0.02331616	0.09635341	0.01730708	0.1293985	0.1480155	0.01731565	0.06417852	0.03462564	0.06872715	0.05384539	0.06313561	0.1452	0.07506341
102.4171	183.2437	379.9431	240.4865	128.0827	104.3252	472.3384	408.2203	348.4567	213.354	299.8107	468.3459	393.5499	270.5001	647.7287	288.235	222.8867	218.5725
0.6572652	1.558873	0.9737222	1.575598	1.173143	1.014381	3.898644	0.5701376	2.437154	1.663382	0.5580194	1.522702	1.880049	1.585922	3.701841	2.54129	2.194229	2.298066
4.431424	4.619644	5.352375	6.640048	5.857187	3.596527	15.11495	3.533594	5.822326	2.822296	3.54571	7.788834	4.282993	4.718499	3.838968	6.263985	5.384204	9.101054
1.224907	0.6060264	1.22141	1.397071	0.3570664	0.0698334	0.6939775	0.1777228	0.6588465	0.5261333	0.2611596	0.4656232	0.2989966	0.09148544	0.1169654	0.1573145	1.080364	0.1376447
0.4125011	0.3416451	1.346973	0.4300079	0.2808615	0.4698017	0.15227	0.1732222	0.5010056	0.2159198	0.6875309	0.6482262	0.5074025	0.7723483	0.2454617	0.5106133	0.361697	0.5719412
7.572371	0.1131507	0.8813013	3.274549	2.775758	0.1032218	0	0.03648537	0.9411161	2.160237	0.1067725	1.30237	0.860013	0.8789687	0.7308455	1.078088	1.970446	0.4170821
0.0329985	0	0.08836859	0.02031178	0.05047124	0.02284416	0.01048921	0.02260897	0.0507116	0	0.01696512	0.0328066	0.09869002	0.009975685	0.01623242	0.04366414	0.03857916	0
0.2066458	0.01538859	0.07447472	0.1248204	0.1949559	0.008021857	0.04235848	0.0463124	0.1899485	0.1253522	0.03276569	0.2841656	0.04331945	0.04378773	0.05415102	0.02299937	0.07451011	0.03320402
0.8784293	0.0432064	0.7785312	0.9397971	1.158019	0.1206583	0.03988938	0.5254308	1.028539	0.3959437	0.01612915	5.413203	0.1329216	0.0632275	0.2469209	0.8625386	1.222605	0.05327233
0.4120151	0.2386809	0.71514	0.3522365	0.6535173	0.07042697	1.23691	0.6795935	1.450057	0.2888852	0.05884009	1.031633	1.74566	0.7011992	2.867489	0.6730677	0.731462	0.5975973
0.7174402	0.03969899	0.4250819	0.3726089	0.139909	0.3352512	0.1624872	0.1413221	0.4823661	0.1455207	0.05532732	1.025323	0.5833563	0.8051942	0.3970336	0.3144649	0.3302667	0.436859
0.08717322	0.02968407	0.1346805	0.08667876	0.1169215	0.02321083	0.6202706	0.4088992	0.1288139	0	0.06722596	0.01999991	0.04512334	0.03648889	0.03958312	0.09760297	0.03527855	0.06404941
0.1324013	0.152138	0.2919503	0.1066407	0.1835496	0.1111604	0.3760902	0.1158063	0.2078014	0.1599895	0.07820791	0.2912692	0.2337955	0.1813938	0.1891542	0.3783476	0.6768066	0.3175058
1.707845	4.486535	3.104007	1.0377	4.566065	3.43616	5.961416	11.26013	7.707816	2.433779	1.792053	4.405827	5.494992	3.213839	8.709039	1.413067	1.511533	6.788652
0.8022463	1.583895	1.888427	0.7629391	0.7362218	1.810532	1.139892	1.709443	2.601377	1.7574	0.8847034	2.504404	1.070693	0.9895012	1.687069	2.807786	1.48426	1.80074
7.14964	10.78669	22.44821	7.241216	9.373183	10.42939	15.02732	20.36912	18.55535	8.582725	4.022704	31.62362	14.23697	13.10066	23.76671	21.98605	15.96057	9.477034
5.567388	4.390993	11.3929	8.770538	12.21524	2.991927	16.47826	49.20812	17.29625	7.396443	6.862561	8.697874	5.797984	1.78967	2.818031	4.715979	8.394949	5.289071
0.1927486	0.3270782	0.2903471	0.2076266	0.162145	0.1501152	0.1301963	0.08804131	0.3085554	0.2606388	0.1362563	0.1836432	0.1801442	0.1602406	0.2607432	0.520723	0.09858886	0.1917765
5.317885	10.65025	20.41772	11.24739	6.403659	3.292896	13.34926	18.96083	18.18332	10.38122	3.95797	32.35543	18.16988	8.489692	24.90175	15.55566	14.00547	15.63808
3.708197	7.777174	8.881997	6.604886	7.688986	5.174847	25.97975	26.03975	7.802868	5.021206	7.100971	5.008543	8.53809	2.571467	7.056044	7.165942	4.786608	9.56293
95.71815	221.4286	395.1491	238.826	129.3558	89.42383	421.5307	416.3724	368.8082	184.1336	266.9244	609.6056	359.6812	231.3021	596.2682	259.8081	247.7136	224.1991
0.1792215	0.07006352	0.1458542	0.3202068	0.1901036	0.06767519	0.4372555	0.4018707	0.230713	0.292705	0.06102842	0.152725	0.5273038	0.2680851	0.5616004	0.1016351	0.1612301	0.2245412
0.6904494	1.391206	1.951007	1.384903	1.092458	0.8900379	1.884437	0.3751873	1.536725	0.8692515	0.5691798	2.224993	0.9612726	1.036442	0.8256805	1.921739	1.273455	1.3872

TCGA-12-5299-01	TCGA-14-0817-01
0.7641794	2.240835
0.5347265	0.7443125
2.522009	7.049358
12.03759	2.107825
24.03079	43.08919
6.198951	13.02696
4.593656	5.84365
5.472712	7.233622
0.4091873	1.679496
5.905149	8.40226
0.1673348	0.1598482
117.0127	166.1155
1.189852	2.939602
0.5751512	0.4509794
1.722862	3.244584
1.179639	2.190647
0.07467822	0.440993
5.825548	9.302252
566.1964	1064.686
2.168374	7.256726
60.10541	106.2513
0.1191348	0.1328763
4.249397	6.058653
23.71907	63.72939
2.594122	5.265878
3.036974	3.84778
32.88735	64.52102
15.91126	15.07225
1.849443	0.7415771
0.6299838	1.134094
0.06620293	0.2523186
1.418481	5.580411
12.27661	31.19477
0.6354401	2.329185
5.721381	18.95987
3.713226	5.171657
11.51192	11.98158
2.86503	3.754914
0.8305573	2.086407
21.32225	33.11232
8.076027	14.62493
2.565064	6.025367
5.461946	7.128638
3.198745	7.492769
0.1690098	0.588133
0.8402072	2.696408
0.2752368	0.3482871
0.4756185	0.8137539
138.8368	210.5282
18.74886	17.28722
21.707	27.53326
8.134186	11.80395
248.7088	404.515
14.1422	5.361323
1.571738	4.789313
1.470352	5.183524
39.31802	36.79242
0.03957329	0.2639444
619.0256	1278.871
0.8112251	0.5367113
1.471982	6.962667
1.412026	5.436103
12.01039	21.87421
101.9187	346.068
0.7811656	0.05033997
1.461483	2.938861
0.484501	0.4453592
16.83406	24.90625
2.193559	6.442951
0.1681162	0.3666147
92.65508	189.044
29.56808	29.95029
1.73736	1.188409
144.2387	191.8489
47.30776	35.51381
19.39765	11.27624
1.795295	7.692377
0.03979213	0.07873876
297.1556	551.8891
1.106344	1.685806
4.975951	10.21238
0.9854883	0.1603164
0.2330431	0.6822367
3.401845	0.3193888
0.02751995	0.01064066
0.09824851	0.01681439
0.7151462	0.2832574
0.6532843	0.8217512
0.05235367	0.7287369
0.1118467	0.09730311
0.2067428	0.4114973
4.483048	4.097438
0.3813607	2.359267
11.2864	39.69871
11.25725	7.918959
0.1406542	0.365152
11.77771	54.90427
5.850937	7.394177
300.9905	607.6975
0.1785832	0.254433
0.7148086	2.971114
