# Supplementary material for: HK3 stimulates immune cell infiltration to promote glioma deterioration
Source: Cancer Cell Int. 2023 Oct 1;23:227. doi: 10.1186/s12935-023-03039-w (PMC10543879; doi:10.1186/s12935-023-03039-w)
Supplement: Supplementary file 4 — Supplementary Table S4. Genes positively correlated with HK3 in leukocyte migration. [file 12935_2023_3039_MOESM4_ESM.pdf]

Table S4. Genes positively correlated with HK3 in leukocyte_migration.

Gene Name	TCGA-E1-5304-01	TCGA-E1-5318-01	TCGA-FG-7638-01	TCGA-DB-A4XH-01	TCGA-E1-A7YM-01	TCGA-TM-A7CF-02
FPR3	0.5434228	2.770934	0.3782211	0.06986406	0.07867353	0.007082217
TREM1	0.03314013	0.01175046	0.0084978	0.004970693	0.05541495	0.004534974
CD74	85.11394	95.62198	114.0177	23.47125	129.1032	83.49108
ITGAM	0.4178552	0.894937	1.136403	0.5013933	1.620705	1.753529
PTPN6	1.031082	1.222017	1.314607	1.026719	3.038969	1.889115
LYN	1.101782	2.398775	2.227441	3.284255	2.563401	1.066503
ITGB2	2.757089	3.764378	3.907635	1.766532	5.993513	5.118101
SLC7A7	0.9156955	1.926583	1.197315	0.5409937	2.992213	1.132536
THBD	0.4233381	0.9656082	0.7072697	0.3770517	0.2069904	0.04777781
FPR1	1.050813	1.627611	1.517427	0.8212394	2.345611	1.021707
ITGA5	0.9940399	7.442968	1.988157	0.9343362	1.666492	0.4016029
FCER1G	5.748218	8.653848	10.16984	4.397377	12.01266	7.188861
ICAM1	0.4500174	1.166691	0.9727803	0.7780436	0.5943439	0.3920024
SLC16A3	0.4744906	0.7257395	0.5398422	0.5023688	1.052229	1.10218
C5AR1	0.522614	0.6952442	0.4176089	0.4739676	0.6503323	0.5521681
DOK2	0.04866473	0.9739489	0.09982897	0.03892923	0.05918128	0.02663759
FPR2	0.009693488	0.00458268	0.01491364	0.01163142	0.04715308	0.00530592

TCGA-S9-A6U2-01	TCGA-HW-7491-01	TCGA-HT-7855-01	TCGA-CS-4944-01	TCGA-DU-7018-01	TCGA-HT-7608-01
0.347074	0.8969299	0.7572288	1.848103	0.2887982	0.3024977
0.04630058	0.2310168	0.01551611	0.01552	0.026801	0.088354
185.0563	55.85619	152.0695	266.5974	46.27379	193.1181
2.137032	0.7429189	2.021598	1.942052	0.3891278	1.877495
2.953108	0.779209	2.326584	1.965016	0.6439207	1.853185
4.235551	1.61244	2.476085	1.906412	2.783766	1.909925
10.82533	3.649748	5.232618	6.904442	1.211936	6.806813
1.703172	0.9420543	1.943993	2.523898	0.6353937	1.628496
0.8487643	0.5340964	0.4740588	0.2330012	2.292759	0.4260409
2.565323	2.961105	2.757719	2.376378	0.5859222	4.230671
1.751196	1.596686	2.566384	2.498309	1.964572	1.454203
12.45443	5.84989	16.40908	23.55695	4.958597	16.23697
1.124947	0.5359562	1.250587	2.6559	1.621672	1.571919
0.8244695	0.6593643	0.9203565	0.6610306	0.3843737	0.4857309
1.969712	1.962753	1.05272	1.644932	0.9121313	2.333013
0.1450458	0.1633365	0.5544271	0.5469696	0.3463329	0.07984228
0.02166867	0.2552736	0.02269231	0.004539601	0.03449289	0.02783151

TCGA-P5-A733-01	TCGA-HT-7470-01	TCGA-E1-A7YS-01	TCGA-E1-5307-01	TCGA-FG-7637-01	TCGA-TQ-A7RP-01
0.1234172	0.03607451	0.252058	0.4568308	0.2405728	0.2353812
0.1289405	0.07919893	0.03228019	0.004062828	0.03005788	0.1261861
40.62643	122.71	53.70305	108.4279	83.67519	258.2561
1.719542	3.280749	0.3515107	4.625966	0.5483898	2.448177
1.434291	2.327206	1.10197	3.690352	1.279384	3.377694
1.903941	2.926392	0.7314653	4.131825	1.888168	2.288961
2.715326	7.358181	2.08676	11.42947	2.658598	7.369275
0.9334584	1.776292	0.7489526	2.72852	1.331383	1.984344
0.6134897	0.2746544	0.3995999	0.2953446	4.03361	1.059845
1.096737	3.265731	1.447779	4.732619	2.489295	4.545143
1.021849	1.155736	1.809698	1.648076	4.928678	2.234927
11.41247	10.5363	4.095105	23.07967	7.3564	14.90629
1.676212	1.83869	0.8578268	1.381032	0.8470475	2.595854
0.6765983	0.7639053	0.6563707	1.227933	0.5424703	1.261819
1.574222	0.9100899	1.675139	3.403165	0.4795191	3.064598
0.02443137	0.04522774	0.08690357	0.03977382	0.2574751	0.2196128
0.02919879	0.02702663	0.02832586	0.009507018	0.07912736	0.02870733

TCGA-DU-7298-01	TCGA-TQ-A7RO-01	TCGA-E1-A7Z6-01	TCGA-DB-A64W-01	TCGA-HT-7603-01	TCGA-IK-7675-01
3.470291	0.9179987	0.2330488	0.08209468	0.08151856	0.6513263
0.1155241	0.1383116	0.04974292	0.01051358	0.01739967	0.1033752
157.7608	103.1588	69.43859	21.50589	32.20633	96.39308
3.673989	1.728836	2.757587	0.2811942	1.244527	1.152249
4.122849	1.938176	3.681511	0.5042898	1.260148	1.725214
4.846696	1.887736	2.307353	1.048609	2.050836	2.083304
12.78078	4.766325	8.104627	1.215339	3.628189	6.116265
2.468212	0.7876769	2.666867	0.5676964	1.318265	1.717276
0.2112016	2.794644	0.7336869	0.9359632	0.4399503	0.3492632
7.915836	4.410341	3.712969	0.5877776	2.584333	3.396817
1.851963	2.873021	2.645629	0.9070221	0.5010277	1.370641
29.67727	7.16496	17.48446	2.903597	11.37661	14.83914
1.9686	1.627167	3.512711	0.7245774	0.406493	0.9535337
1.090702	1.450164	1.290565	0.5009245	0.5861724	0.8886578
3.887825	1.811587	2.155958	0.1927876	0.4441284	0.9987806
0.09978927	0.2031042	0.02656187	0.0617548	0.02725397	0.397825
0.02782772	0.0231178	0.02116335	0	0.04885826	0.02085327

TCGA-S9-A7QW-01	TCGA-DU-6399-01	TCGA-DU-A76K-01	TCGA-F6-A8O3-01	TCGA-DU-7009-01	TCGA-CS-5390-01
0.1703411	2.558067	0.08106147	0.4487442	0.2355539	0.764979
0.08536302	0.02905567	0.2627757	0.005986366	0.02246446	0.03991297
45.82454	373.1289	64.91334	49.06672	18.96279	55.74608
1.098064	4.979102	0.4784594	0.697623	0.9000192	0.8401401
2.061588	3.96757	1.045793	0.7527709	1.370129	1.166543
1.07545	5.88491	1.6008	2.103366	1.810427	1.909797
3.956752	14.16829	1.799652	2.42107	3.084307	2.756971
0.9482681	3.876297	1.075668	0.5252695	1.007225	1.187787
0.1698743	0.9221667	1.045859	0.4477886	0.3144361	0.8027718
3.743484	7.043113	1.007003	0.8941367	1.00687	1.955877
0.4335184	2.776434	1.616244	0.9918627	0.8305897	1.03031
9.306561	26.96028	3.918889	4.139945	8.47395	6.810958
0.4930255	1.561245	0.8526383	1.031425	1.083373	1.051127
0.817084	1.604612	0.4746354	0.5041825	0.4185562	0.4749815
1.954888	3.116845	0.7233727	0.7113227	0.8144464	0.910198
0.01857061	0.1493341	0.1270366	0.1289302	0.09425147	0.1847114
0.01664579	0.05949149	0.007591297	0.007004048	0.003754775	0.04245289

TCGA-HT-7620-01	TCGA-F6-A8O4-01	TCGA-DU-6410-01	TCGA-HT-A616-01	TCGA-FG-6689-01	TCGA-QH-A6CS-01
0.05617849	0.3044991	0.05152367	0.1085955	0.1030917	0.433556
0.02055595	0.09926289	0.01832905	0.2135787	0.07261424	0.04307897
139.0488	72.80068	21.19727	76.01304	284.2488	319.4312
4.084122	1.909878	0.1288592	1.597935	5.637212	3.547989
2.704777	1.213279	0.285134	2.219303	5.071862	5.038563
2.906276	1.821264	1.193083	3.462555	5.195609	6.52619
8.899327	4.567674	0.7587935	4.542702	13.25003	16.95298
2.094162	0.7746655	0.3958818	0.896786	3.759399	3.226675
0.1732523	0.3772261	0.5291051	2.480386	0.2225515	0.4538547
3.833565	2.467076	0.4098853	8.78644	2.979985	6.278603
1.429871	0.5549703	3.292401	1.364975	1.906517	2.953499
13.45417	10.44343	1.991892	8.052064	24.93077	26.41711
1.548744	0.5093499	0.4967182	2.135106	3.265613	1.766819
0.8681029	0.5903253	0.4216521	0.8509928	1.245379	1.677029
0.9197205	0.6864694	0.2312367	5.71247	1.956135	2.373329
0.03018546	0.2012916	0.1148388	0.1944994	0.05169969	0.0656024
0.02405047	0.04562548	0.004288998	0.03486796	0.0270323	0.02240107

TCGA-QH-A65R-01	TCGA-DH-5143-01	TCGA-HT-7606-01	TCGA-CS-5394-01	TCGA-HT-7875-01	TCGA-S9-A6WM-01
1.116444	1.524647	1.414679	0.5783841	0.2883617	0.4268328
0.07065826	0.01892017	0.06285596	0.01122298	0.0134289	0.05694062
91.14001	272.4642	104.3914	63.05985	31.66408	311.8282
0.4414856	4.776973	2.339459	0.5660303	0.9928399	2.414921
2.130126	3.751069	2.153743	0.6498594	0.8849464	2.765636
1.486561	4.430971	2.518015	1.047357	2.213499	3.299897
2.853666	16.79561	7.177658	1.181786	2.473912	8.065426
0.540033	2.451895	2.451916	0.5807513	0.7336111	1.844756
5.591861	0.2352115	0.3116298	0.7764338	0.7286169	0.7078735
1.484381	6.71915	3.733108	0.2622127	1.669608	0.8647314
3.393412	1.312303	3.210934	1.897973	1.271699	5.253939
7.633925	31.58967	19.47628	3.107915	4.522852	24.29161
6.719428	2.789116	0.621931	1.03566	0.4784346	2.041939
1.000162	1.311215	1.133505	0.2754639	0.4911799	1.222203
5.347269	4.107761	0.5532432	0.4664704	0.5614406	1.077532
0.5696537	0.09631575	0.07963226	0.1684665	0.1248915	0.3679044
0.004862951	0.0221366	0.02162984	0.0218848	0.01963977	0

TCGA-HT-A5RB-01	TCGA-P5-A5ET-01	TCGA-DH-5144-01	TCGA-CS-5395-01	TCGA-QH-A870-01	TCGA-S9-A7IZ-01
0.1722724	0.1205152	2.363821	0.2709879	0.2784982	0.5689999
0.02005666	0.01286164	0.03220493	0.07493012	0.1548668	0.2677747
75.28357	19.65519	109.0957	93.2646	102.7909	466.2176
1.835362	1.264591	0.623451	2.133653	2.542602	4.03546
1.542713	1.344988	1.719456	2.154053	2.717414	3.376496
2.726889	1.371321	2.950346	3.450331	2.069345	4.408994
4.349567	4.002943	4.763174	8.61775	7.278028	14.50195
0.8460853	0.9368292	1.171378	2.129455	1.26701	2.41105
0.8135247	0.1355027	1.161133	0.4778066	0.05933038	2.335514
1.832569	1.209148	1.039075	4.080488	2.404365	2.776493
1.140176	0.9038404	1.011988	5.29494	0.7078458	2.274769
5.336512	6.930876	12.01965	18.15253	12.51819	13.40476
1.00156	0.7862432	2.165169	1.612327	2.165328	2.42027
0.8639091	0.7118371	0.5493599	0.9319031	0.8943882	1.043646
0.6105132	0.5094229	1.091846	1.209115	1.769278	2.33757
0.06872195	0.1091233	0.1751534	0.077215	0.04594236	0.1117331
0.01759972	0.01504812	0.004186641	0.05998354	0.0329444	0.03081604

TCGA-VV-A829-01	TCGA-P5-A730-01	TCGA-QH-A6XA-01	TCGA-14-0790-01	TCGA-DU-A76R-01	TCGA-HT-7480-01
1.176844	0.605515	0.1120092	0.9839142	0.9053444	0.2485173
0.0528822	0.0392639	0.0941366	0.1911334	0.02246983	0
138.2502	118.8052	97.56427	263.5533	66.0679	78.85214
0.9462857	0.4913177	5.573326	1.06027	1.792619	2.126839
2.212794	0.9458992	7.016075	1.68245	1.747748	1.319169
3.482331	1.216059	3.953281	9.820292	4.563994	2.420826
4.768612	1.487924	12.79621	4.310236	4.533726	4.19887
2.531984	0.8985635	2.167102	2.221782	1.262582	0.9672225
1.053915	2.187234	0.2503033	0.7383453	0.7243903	0.3216218
2.618158	0.1556233	5.640637	0.8624091	2.894907	1.528385
2.986093	6.065272	1.249825	5.435787	1.838371	1.52335
13.71425	6.722237	17.99647	18.05642	11.4022	7.612848
0.8287744	5.257206	1.28812	3.836834	1.973785	0.6524943
1.202535	0.4747729	1.781992	0.9232697	0.5990953	0.5501678
3.671935	0.3743908	4.32039	1.080002	1.285532	0.5478761
0.8542054	0.8264199	0.1228757	0.360367	0.05279343	0.06994516
0.02578007	0.01148469	0.03146852	0.02484735	0.03680558	0

TCGA-R8-A6MK-01	TCGA-S9-A6UB-01	TCGA-HT-7689-01	TCGA-E1-5302-01	TCGA-TM-A7CF-01	TCGA-HT-7692-01
0.800474	0.5697466	0.3431353	3.663997	0.02389246	0.8016348
0.01857136	0	0.1391565	0.2035146	0.02039883	0.01833261
177.5214	36.51132	164.5181	424.1454	56.12762	116.1614
1.668932	0.9945973	3.800207	5.170269	2.587623	1.476565
2.211717	1.167462	3.509845	5.177031	2.005364	2.325877
2.006961	2.139416	3.811348	5.756877	2.055965	2.281413
7.051899	3.730289	14.21789	20.26934	5.001915	4.791835
1.59506	0.8235812	2.759033	6.991279	0.9852951	1.302332
0.2230489	0.2181506	0.2237686	0.4530948	0.2525193	0.1390619
2.188095	0.6252978	8.250327	11.02854	1.251066	0.9606639
1.519417	0.5999645	1.25534	2.672128	0.4924048	1.015982
15.87893	5.556994	20.35874	25.1413	6.114587	16.33339
0.6464608	0.3858448	2.54946	2.906543	0.7148398	0.4411371
0.6846348	0.5172477	1.334142	1.992163	0.9301884	0.6528935
0.7519193	0.4917937	1.283914	3.064339	0.8079555	0.7691457
0.04363388	0.09652827	0.1290598	0.1503656	0.06989437	0.1363976
0.0173828	0	0.03856099	0.03144876	0.01789997	0.00428983

TCGA-E1-5305-01	TCGA-P5-A77X-01	TCGA-QH-A6CZ-01	TCGA-DB-5277-01	TCGA-FG-A711-01	TCGA-DU-6401-01
0.4620642	0.1023956	0.3191856	2.724366	0.1573227	2.880754
0.1288487	0	0.03044031	0.05614484	0.01313985	0.5357878
89.9927	48.41739	23.31269	135.7947	107.0059	168.9773
1.8416	1.189976	1.209595	0.9071125	3.249945	1.985739
2.610697	0.9314497	1.740077	1.982505	3.391671	3.066063
4.41566	2.208542	1.756203	3.011823	2.473948	3.383109
11.06642	2.150625	3.090904	5.018971	6.812164	5.537879
3.297507	0.5428156	0.818166	2.077475	1.810718	1.375498
0.5027681	0.2072334	0.4673074	1.753401	0.1568915	6.051972
6.713693	0.6975653	2.155386	1.57947	4.846188	12.39037
1.878563	0.8592577	0.4472124	0.9921103	1.096058	5.072745
15.6655	6.90095	8.690036	19.43183	14.86968	15.02058
1.49394	0.5361265	0.4114511	3.400953	0.5423219	58.51314
1.102412	0.4536087	0.6864537	0.6446362	1.570406	2.913786
1.42112	0.3606922	1.569295	0.8236225	0.5332927	11.34617
0.196216	0.05616477	0.04257159	0.7459406	0.06002968	0.989094
0.08375166	0.01438382	0.01526364	0.02815262	0.02049817	0.1746286

TCGA-CS-6188-01	TCGA-FG-7643-01	TCGA-CS-5393-01	TCGA-P5-A5F0-01	TCGA-FG-A87Q-01	TCGA-HT-7609-01
0.8845744	0.1395908	0.1187355	0.06162348	0.87785	1.6899
0.3208404	0.05720609	0.008447801	0.004932441	0.3075729	0.2552838
330.0877	167.2179	310.2235	36.98469	527.3602	256.8077
3.247816	2.092853	5.022396	0.8292247	5.187018	4.91483
4.039453	2.230982	5.483024	0.8397783	8.799497	6.315875
6.442998	5.417864	6.474491	1.860795	9.843317	4.976315
12.19498	5.979809	17.08275	1.778069	28.67127	12.73054
5.383752	1.324257	3.503102	0.5118616	9.14506	2.619941
1.076651	1.190312	0.4583554	0.4469015	0.3128668	0.6663584
9.208157	3.257861	4.906146	0.5926687	9.265831	12.5075
7.850523	3.987424	1.650669	0.9806893	4.126554	1.806828
27.07255	8.766485	26.10013	3.41109	54.4624	22.82521
2.489249	1.357337	1.425915	0.7720561	6.88822	2.723842
3.162545	0.7726006	2.101941	0.4700169	3.276963	1.866735
5.229556	1.164377	3.023792	0.2387779	4.527531	4.784544
0.2559274	0.1190063	0.1571325	0.1545186	0.1765094	0.104442
0.06024669	0.06693113	0.07412945	0	0.05584039	0.04903734

TCGA-QH-A65V-01	TCGA-TM-A84H-01	TCGA-DU-7008-01	TCGA-S9-A6WD-01	TCGA-P5-A72Z-01	TCGA-DB-A64Q-01
0.157645	0.7760891	0.4522981	0.3024447	0.2697536	0.5407905
0.009613831	0.007888178	0.05213183	0.004210113	0.03172629	0.06641097
83.88357	78.00719	72.39894	43.10831	32.17421	60.16742
2.027651	2.685419	1.580216	1.393059	0.301705	1.555094
2.887273	3.153007	2.285271	1.335354	0.7555531	1.701361
2.829504	2.062126	2.136579	1.526411	1.088086	1.975498
8.361005	9.136089	5.506113	5.22876	1.733263	4.429106
1.760118	1.617218	1.143568	0.9981267	0.4907342	1.636894
0.1671213	0.2036077	0.4332816	0.2838737	0.2376885	0.3748215
6.080696	2.613093	1.991339	2.283467	0.2647323	3.309068
0.8019359	1.327249	0.8538281	0.7528846	2.334267	1.154547
26.03301	14.50578	13.05794	9.717789	3.894237	14.40022
1.353212	2.925517	1.133333	2.085172	0.9182561	0.5541074
1.890057	1.188249	0.8251608	0.9421785	0.3868103	0.7967529
0.8743927	1.695246	1.372307	0.7596565	0.3102747	0.8420067
0.03764656	0.1467233	0.07938845	0.08243139	0.1656483	0.1300285
0.07311318	0.02307292	0.02033141	0.01970333	0	0.03885042

TCGA-HT-7468-01	TCGA-E1-A7Z2-01	TCGA-DH-5140-01	TCGA-TM-A84G-01	TCGA-HT-7485-01	TCGA-TQ-A7RF-01
1.893059	0.01894212	0.8023764	0.4708689	0.8830636	0.8343965
0.03798878	0.0283016	0.03519095	0.01773604	0.007068181	0.1260121
53.73473	65.59047	258.6267	36.40025	158.2186	322.7191
1.097852	1.152417	1.55972	0.42354	4.005037	2.66157
1.632625	2.037127	2.363135	0.7395893	2.703071	3.097265
3.289794	1.516791	2.371948	1.328276	2.93861	2.247292
5.56925	4.019631	6.676852	1.443072	11.61217	9.926177
1.858955	1.459965	2.113959	0.5574021	2.230308	1.756337
0.3456509	0.7369033	0.9565377	1.004354	0.2382918	0.8390363
3.187171	2.509999	1.703125	0.5697773	3.951574	2.666546
1.274634	2.074338	2.832906	0.9195888	1.01765	2.007222
15.80329	9.368568	14.66525	4.758885	31.6456	17.29323
2.206643	0.6517398	2.075891	1.854223	2.518197	3.29129
0.5850483	1.490487	0.999239	0.5676293	0.9082499	1.249298
1.079097	1.364141	1.321567	0.2471717	1.887111	2.225651
0.2839951	0.05541277	0.4892019	0.2170379	0.03459764	0.1875101
0.04040625	0.009460821	0.008234681	0.01037559	0.03721397	0.005897366

TCGA-19-5960-01	TCGA-QH-A6X5-01	TCGA-TQ-A7RS-01	TCGA-DH-A7UR-01	TCGA-DU-6407-01	TCGA-QH-A6X3-01
0.779935	0.3963426	0.3352981	0.4911081	1.943527	2.531305
0.1519967	0.03230069	0.008257781	0.004492465	0.0689139	0.08809115
172.9005	167.29	28.64663	69.16702	79.86212	330.0432
0.9192501	1.625558	0.6783589	0.6865977	2.487489	0.6193095
2.161849	2.309026	2.497859	1.226898	2.718668	4.297667
4.936284	1.601929	1.658797	3.323591	3.489883	2.083996
6.508164	4.984366	2.719094	1.937063	13.27579	5.369261
2.608746	1.199085	1.187882	0.6974099	2.585623	2.099599
0.5764853	0.1798734	0.2348976	2.570017	0.3032269	1.953601
2.44963	2.017589	0.7303934	0.3748628	6.061548	1.190789
1.989898	0.8357248	1.316309	4.661126	1.396579	0.8455355
21.17906	14.40155	9.029691	5.628588	23.81092	15.32902
1.562425	0.6144701	0.8681366	1.810448	1.73309	2.160895
0.7398708	0.9482613	0.9240016	0.7524027	1.353954	0.6634879
1.554442	0.8258325	0.7147164	0.4217771	1.635343	2.326072
0.425143	0.08131208	0.1374299	0.3166548	0.1428661	1.72477
0.0304862	0.01079766	0	0.01576855	0.05217188	0.005153332

TCGA-TM-A7C4-01	TCGA-FG-A70Y-01	TCGA-27-2528-01	TCGA-FG-8191-01	TCGA-DU-7306-01	TCGA-TQ-A7RM-01
0.03109317	0.4910706	1.032233	1.27652	1.754331	0.751047
0.06968491	0.02096323	0.4910082	0.7334682	0.8671517	0.04964334
58.76856	186.4077	174.1036	277.3593	204.6394	46.69431
1.711634	4.466206	1.33128	3.446604	1.985433	0.5903751
1.574448	4.612679	1.91366	5.689294	2.398747	1.016288
1.930049	4.199289	4.279304	3.629336	2.972607	1.107539
4.523817	16.86289	5.872567	8.861809	7.53538	2.790269
0.6971149	3.480719	1.919942	2.081218	1.817944	0.9607108
0.3513476	0.3798728	4.874532	4.228919	0.4152636	0.6374221
1.819166	11.22303	2.308557	13.53762	4.544261	0.8595413
1.023773	2.313959	13.57603	4.715939	3.210935	2.829204
9.097366	25.361	21.87992	25.67821	19.78019	7.259209
1.354718	1.317415	4.632516	3.716926	12.63854	0.6342511
0.5629437	3.428215	3.642297	2.637798	0.7398155	0.8810072
0.8616107	3.622613	2.957343	18.0827	2.279138	0.5461861
0.08771059	0.1067162	0.6008522	0.8287854	0.3858702	0.3158953
0.017471	0.05886476	0.03314306	0.0811194	0.065332	0.01089051

TCGA-HT-7602-01	TCGA-HT-7695-01	TCGA-DU-8164-01	TCGA-WY-A85E-01	TCGA-DB-5278-01	TCGA-QH-A6CY-01
0.4280511	0.499555	0.9328514	0.4897647	1.395052	0.2531406
0.03003782	0.01378799	0.0155556	0.08845476	0.05094852	0.01620942
198.5321	32.30737	34.40611	111.6488	49.05712	106.3192
4.068575	1.277001	0.8582459	1.219767	1.832452	1.470919
4.886973	1.205919	1.053997	2.41849	1.690828	1.705583
3.716	3.352576	1.992443	1.770097	2.852788	1.928698
10.96209	3.579361	2.054783	3.518484	6.543891	4.710214
2.470919	0.9259758	0.8530722	0.9226841	1.77958	1.046199
0.328328	0.3399134	0.4850986	1.198772	0.2648034	0.1878502
4.311064	2.917681	0.9553275	2.952357	2.516755	1.034705
1.379366	0.7909062	1.082491	1.084254	1.156583	0.6112258
25.91569	10.17919	6.189152	10.00274	16.19852	11.70116
2.508745	0.6667814	0.9339683	1.235198	1.019661	0.44969
1.140234	0.5879239	0.34138	0.6946714	0.6702095	0.7098968
1.057543	1.072001	0.502028	1.038077	1.34531	0.6717447
0.05146065	0.08098808	0.1340104	0.06297778	0.2527102	0.1031453
0.02635818	0.04516946	0.0327601	0.02822511	0.03576586	0.01422376

TCGA-FG-7636-01	TCGA-P5-A5F4-01	TCGA-HT-A5R5-01	TCGA-FG-5963-02	TCGA-DU-6393-01	TCGA-S9-A6TY-01
0.5940999	0.1027227	0.5709028	2.306685	0.5009306	0.4511321
0.0568446	0.02023899	0.710826	2.732264	0.00851581	0.01220597
267.4113	58.89357	455.7624	165.8131	70.43396	28.77083
2.151887	1.562061	4.135997	0.7152617	0.6507492	0.6871175
3.125273	1.757889	9.96088	1.697362	0.843917	0.7700727
2.925847	2.882711	6.083968	3.472549	3.45702	1.870959
8.285202	3.606029	18.23508	4.507225	1.988693	1.775055
1.69334	0.8964658	2.37318	1.447025	0.8070867	0.5938581
0.5896676	0.6023636	5.103112	3.790568	0.4844748	0.3429197
2.787577	1.579022	30.07176	2.627495	0.3031812	0.5228286
1.211652	1.098507	5.601545	12.63666	1.960746	0.5718425
20.9125	8.38428	42.29507	13.02287	4.563503	5.645729
0.6333586	1.595787	16.48778	10.78815	0.8687367	0.6986353
0.8880649	0.7110467	10.85915	2.085692	0.4549204	0.3230884
1.969258	0.6160633	19.63356	2.762033	0.4747095	0.310365
0.05992978	0.1684134	0.3479383	0.6991558	0.4168356	0.04779705
0.005116014	0	0.2851428	0.1645851	0.009963498	0.01428098

TCGA-DU-5855-01	TCGA-QH-A65Z-01	TCGA-S9-A7QY-01	TCGA-HT-7680-01	TCGA-P5-A5EV-01	TCGA-HT-7482-01
0.8363854	0.245435	0.3702189	1.057859	0.5546764	1.363492
0.4108443	0.01428727	0.01293072	0.4294739	0.02165716	0.08807472
543.257	73.66009	68.05507	275.6659	180.7883	123.1055
5.785709	0.9753278	1.231858	5.269707	3.336412	6.10708
7.60547	1.596969	1.49004	4.659245	3.301692	6.178803
5.663862	1.423324	1.57478	5.083267	2.655727	7.228568
17.61107	3.163609	3.653843	13.72772	11.8044	20.24042
3.840755	0.8437873	1.134597	3.202188	1.929527	4.881727
0.7999835	0.1304527	0.2497558	1.419941	0.2692371	0.423608
12.98427	1.692877	1.43863	9.439833	2.884177	18.24514
3.883515	0.8434904	0.7092254	2.543503	1.588755	1.592741
39.2549	10.05163	11.2651	22.70444	24.2943	59.99775
3.607892	1.379628	1.414784	4.03784	1.325607	3.90499
2.063765	0.7471919	0.8048587	1.164532	1.079811	1.968254
10.0359	1.397258	0.7018477	7.667819	2.173081	6.426402
0.2082841	0.08392078	0.08439182	0.1230561	0.2798622	0.112464
0.06866969	0	0.02017193	0.1266424	0.02533888	0.0985671

TCGA-DB-A4XF-01	TCGA-FG-A4MT-02	TCGA-DB-A4X9-01	TCGA-HT-A5R9-01	TCGA-S9-A6TX-01	TCGA-S9-A6WE-01
0.9936917	0.5769434	0.05444565	0.5909596	0.8284158	1.15927
0.752866	0.146827	0.1801272	0.09580019	0.01224142	0.0366577
580.9583	210.3688	49.50987	34.22995	137.3483	28.96961
2.475708	2.370681	3.934042	0.6149408	2.098521	1.306086
2.480904	2.513328	3.399717	0.9397207	1.914904	1.489017
3.205868	2.850956	2.53369	1.988103	1.747873	2.09108
8.822788	6.760151	10.55449	2.556529	5.470576	5.477845
2.094076	1.84617	2.083493	0.7112702	1.525106	1.871139
1.161618	1.172637	0.2571096	1.468523	0.8898821	0.695167
2.107546	3.841476	5.769686	2.006447	1.716046	3.976456
2.944365	1.696688	1.161919	2.208514	1.5783	1.555077
13.82884	15.88823	14.01207	6.754241	7.879337	17.65522
1.88935	0.9405358	2.27376	2.568214	0.8579567	0.7975238
0.7643991	1.401864	1.325508	0.5048553	0.8974563	1.656157
1.189928	5.065125	2.974825	0.8502372	1.173235	5.962346
0.2757927	0.2874781	0.307171	0.3094918	0.3515298	0.1884054
0.0227317	0.01662461	0.04758844	0.1737336	0.01909662	0.01608356

TCGA-HT-8558-01	TCGA-HT-8010-01	TCGA-HT-7694-01	TCGA-DU-7299-01	TCGA-E1-A7YU-01	TCGA-P5-A77W-01
0.01108469	0.1859546	0.09266825	1.596586	0.02984645	0.4681193
0.02484261	0.01536423	0.04537651	0.1545407	0.1003362	0.003842975
57.2701	136.5121	37.65255	227.9548	79.86957	33.10006
1.261072	3.102513	0.8081984	5.448257	2.661666	0.5785241
1.012161	1.981809	0.9230935	4.471724	3.361238	1.039557
1.818269	2.234085	1.71992	4.67194	2.411134	1.425579
2.640052	5.536123	2.847785	15.612	8.867438	1.98744
0.7006493	1.121276	1.007162	2.883521	2.458973	0.6322484
0.6057114	0.5746331	0.3934802	0.4226928	0.2265178	1.534469
1.75903	2.48073	1.001922	4.200884	2.623323	0.4040413
1.072618	1.450583	0.6202095	1.424597	1.766171	1.449112
3.76116	8.365717	8.730788	33.43166	14.78319	7.714107
0.72547	1.381923	0.7379865	1.447631	0.6753141	2.029029
0.6524527	0.7837962	0.4804411	1.315873	2.294855	0.4161372
0.4060806	1.267803	0.5274039	1.569497	1.541982	0.6595866
0.07643474	0.1278494	0.06834182	0.06982641	0.1216131	0.2332535
0.03737039	0.01797615	0.0285872	0.04868033	0.03913111	0.004496281

TCGA-DU-6400-01	TCGA-WY-A85A-01	TCGA-FG-A60K-01	TCGA-QH-A65S-01	TCGA-DU-7309-01	TCGA-E1-5303-01
0.9602926	0.2068612	0.6258556	1.007034	0.3498617	2.398597
0.02660654	0.005519165	0.06304023	0.09701082	0.1664207	0.2663891
104.1183	163.9755	108.7111	119.421	121.0487	280.6711
0.6935407	3.513226	2.184999	2.786505	3.895897	8.015392
2.273906	2.866714	1.825152	3.856688	2.364867	7.885217
2.369187	3.136731	3.438447	3.390992	4.213478	7.830936
4.40964	7.202539	7.370436	14.64693	7.04039	35.50726
0.9652558	1.643742	1.80455	3.02836	1.539092	9.825843
0.3955497	0.313992	0.4696522	0.1503013	2.184895	0.4385186
1.692223	4.034271	5.124975	6.742518	4.310178	18.40077
1.820769	1.239242	1.178272	1.08895	2.400817	2.829918
11.33742	18.51418	11.93033	31.26379	13.33714	64.22443
2.869651	1.012174	0.6522193	1.486477	2.893525	2.562296
1.047153	1.415751	1.198399	2.293212	0.7886066	3.713921
1.465821	1.983617	3.672679	1.741217	5.164343	11.15561
0.347293	0.1296742	0.1851433	0.07821105	0.2193163	0.1140942
0.0276708	0.02582969	0.05268362	0.08679615	0.1872233	0.04869926

TCGA-E1-A7YH-01	TCGA-S9-A89V-01	TCGA-FG-A713-01	TCGA-HT-7684-01	TCGA-EZ-7264-01	TCGA-HW-7487-01
0.3631519	0.5880969	0.147662	0.3081784	1.073894	0.2784823
0.0841948	0.1075936	0.004110992	0.6593933	0.009419852	0.05383284
152.5096	262.8566	62.48891	224.5745	22.02007	52.97707
3.008605	5.590917	1.473354	2.626091	0.7558247	1.326675
4.563397	4.924445	1.296809	4.596453	1.058337	1.517869
3.207451	5.804901	1.346605	3.203704	3.072401	2.293687
10.36211	12.28047	3.440427	7.718804	2.373799	3.135032
2.442242	2.677898	0.7006217	1.644616	0.8848199	1.175204
0.1055984	0.5714945	0.5587117	1.191635	0.2084082	0.2977543
3.412351	3.366707	1.262357	10.72316	0.8226974	1.662022
1.672165	2.353862	1.585407	4.24032	0.9543876	0.7785252
21.15507	14.71645	4.966031	54.44559	7.727054	7.871828
0.9881675	2.136572	1.051654	10.98015	0.9279521	1.28123
1.838846	1.374362	0.6944487	3.604292	0.5047261	0.6266495
1.25863	2.091321	0.5306983	7.893764	0.7323832	0.5083735
0.07064921	0.1141083	0.1851285	0.1130848	0.08606959	0.07246342
0.01407256	0.03671632	0.009619721	0.0732071	0.0257162	0.04330179

TCGA-S9-A6U6-01	TCGA-S9-A6U9-01	TCGA-DU-5849-01	TCGA-HW-A5KJ-01	TCGA-FG-7641-01	TCGA-S9-A7IS-01
0.1490218	1.570415	2.200096	0.05600464	0.07916921	1.630205
0.3860316	0.2145254	0.08115169	0.0179308	0.009217205	0.2546032
123.2165	285.1448	105.8507	50.01332	22.24396	158.5122
3.828269	7.636545	0.9822916	0.1507232	1.000173	1.642635
6.3014	14.63906	1.851561	1.026649	0.9399783	2.495876
4.292696	6.981995	2.274257	1.044797	1.345096	2.749398
9.37318	23.7994	4.562787	0.6966261	1.972463	8.791197
1.127117	4.434354	1.673341	0.589997	0.6571152	1.828933
3.061675	3.705643	0.7455306	4.939952	0.2184908	1.302856
12.00148	14.30552	1.338049	0.1271763	0.6537411	7.059326
2.126231	8.886014	1.479963	2.289641	0.7398878	5.440337
15.17333	35.46374	14.24173	2.03316	5.401574	18.6854
3.815135	5.345885	2.904481	1.392847	0.5706336	1.533752
3.824236	4.727718	0.7326842	0.280459	0.4369401	3.451908
4.154936	12.01762	2.052358	0.3024934	0.4867659	3.436134
0.1188939	0.8488052	0.584715	0.7460319	0.02707007	0.291977
0.07104716	0.04706151	0.06456428	0.005244758	0.02156826	0.04681063

TCGA-P5-A72X-01	TCGA-DB-A64U-01	TCGA-DB-A4XG-01	TCGA-DB-A64R-01	TCGA-CS-4943-01	TCGA-W9-A837-01
0.3597393	0.4256063	0.5052085	0.3994488	2.233055	0.08591144
0.02214931	0.01277483	0.004094956	0.04897917	0.02910734	0.02750595
216.5951	61.40445	9.759907	38.88268	169.513	45.71563
2.474545	2.333139	0.5476145	0.7485643	3.594999	1.366242
2.519142	2.31484	0.5945592	1.241679	3.474652	1.07313
1.832422	2.819504	1.728281	1.900717	3.972693	2.491751
5.938991	6.603938	1.048445	2.74514	13.27583	3.148325
1.502456	1.480187	0.8222649	1.014721	3.827935	0.8486999
4.134999	0.3499289	0.5737891	1.049232	0.6401481	0.3146253
1.131096	2.309589	0.4783711	2.661057	9.387277	1.324639
2.624577	1.335661	2.491662	0.7835375	1.56738	1.037196
9.230363	11.72208	4.983438	9.628879	23.10322	5.84073
2.882223	1.273374	0.6983679	0.8836208	1.802024	0.3901483
0.7589736	1.098462	0.5064876	0.6652564	1.76506	0.5200531
1.969025	1.261838	0.1561856	1.421043	4.248582	1.400726
0.2602018	0.133399	0.03207067	0.05327676	0.5627797	0.03846781
0.01554882	0.03487527	0.004791099	0.0891421	0.07236812	0.01838969

TCGA-DU-7014-01	TCGA-QH-A6X9-01	TCGA-S9-A7R1-01	TCGA-DU-5874-01	TCGA-DB-A4XC-01	TCGA-P5-A5EW-01
0.6700608	0.1893625	0.4087085	1.253171	1.004612	0.7464456
0.1132246	0.02090603	0.1727282	0.02947762	0.1538292	0.07454633
180.634	62.63525	37.5749	50.57854	358.4413	62.63676
2.119799	2.632791	0.3719803	0.7909057	3.884527	4.312643
2.817158	3.55938	2.162289	0.9171313	4.870658	5.32085
3.382642	2.300234	1.663233	2.386429	5.290414	4.250842
6.330729	6.135562	1.746831	2.19033	16.94943	14.95292
1.686807	0.984218	0.6977374	0.7461038	4.689956	2.389986
1.456555	0.365621	2.823389	0.6004137	1.112358	0.3926881
3.510586	3.140015	1.257852	0.7160426	6.499634	6.725281
4.199686	0.9794352	2.269768	1.800177	3.403658	1.112373
24.84733	10.02659	4.771511	7.633573	19.18527	35.76051
1.653229	0.8693681	7.239078	1.740778	2.205271	4.205355
0.8718612	1.151023	0.6574705	0.7708062	1.420087	2.194811
1.263204	1.398475	1.873517	0.5765667	1.405255	4.078354
0.595054	0.03274616	0.3279429	0.3783561	0.3833301	0.1802999
0.05577803	0.02446005	0.024496	0.02682464	0.09817099	0.04104433

TCGA-CS-6669-01	TCGA-DU-A7TC-01	TCGA-06-2569-01	TCGA-DB-A64O-01	TCGA-TM-A84R-01	TCGA-HT-7854-01
0.006448587	0.184657	0.7941074	0.3502848	0.09859009	1.132266
0	0.004077308	0.1875993	0.06259502	0.01262609	1.263518
28.67151	108.6591	37.4337	318.3063	51.40814	390.9091
1.107554	1.941106	0.5017495	3.053339	1.328267	5.217668
0.7815393	2.526558	1.297053	3.304449	0.9966318	11.84154
1.219637	2.137486	1.890092	4.611155	1.820472	7.235006
1.859345	5.676674	1.622436	13.97992	2.862788	21.32655
0.6897964	1.809443	3.086394	2.556938	0.6391543	5.018331
0.2479685	0.6787066	4.010084	1.203522	0.9887897	2.869984
0.4410296	3.762844	0.7662107	4.387394	1.116766	13.93806
0.5756391	0.8991893	10.75768	2.688162	0.7694633	5.043185
3.356289	12.43237	7.138617	27.31289	4.384763	33.41201
0.6173939	0.4762727	1.395549	2.735817	1.091397	17.36985
0.4491227	1.293139	1.278252	1.292862	0.5509383	3.477462
0.3513311	0.7476561	2.636133	1.438582	0.3395695	12.1951
0.1212719	0.04789869	0.7346155	0.1225571	0.09888439	0.3790082
0	0.02385225	0.005776084	0.04882411	0.0393934	0.1029468

TCGA-HT-7688-01	TCGA-DU-6408-01	TCGA-HT-7481-01	TCGA-DU-A7TB-01	TCGA-HT-7681-01	TCGA-HT-7474-01
0.4553832	0.298192	0.2708657	0.1244398	1.237622	0.4024928
0.2946662	0.1079238	0.02420151	0.006928942	0.02430951	0.03391174
83.23531	288.5192	49.80663	123.1281	179.3307	100.0517
1.224383	4.491534	1.186696	1.453439	1.946817	1.689606
1.199041	4.236725	1.554757	1.943203	2.445489	1.600219
2.101095	4.28823	2.792128	2.25996	3.120495	1.962924
3.49931	13.68914	2.892676	4.699881	8.07887	3.537809
0.8804882	3.043331	1.157059	1.318254	1.411075	1.273197
1.823852	0.4548085	0.2889692	0.2481974	0.7017432	0.6037933
2.873687	5.971297	1.272244	1.266189	4.66543	1.890228
0.6015109	1.603175	0.7697114	1.456817	1.486108	0.8524975
9.510836	19.79607	10.95865	10.06588	13.18723	9.121946
1.150923	2.211011	0.7774228	0.4653899	1.556105	1.192336
0.521459	1.522247	0.5765461	1.050422	1.081022	0.6054949
2.228859	6.162298	0.4970371	0.5997045	1.426444	0.70641
0.1081759	0.1137812	0.08687257	0.06783218	0.5711581	0.1261545
0.09696359	0.03399599	0.03775435	0.004053431	0.03413055	0.03967674

TCGA-DU-7302-01	TCGA-DH-A66F-01	TCGA-HT-A5RC-01	TCGA-P5-A72W-01	TCGA-HW-7493-01	TCGA-HT-A61A-01
0.6780835	0.7674525	1.057991	0.5839482	1.878028	0.007622077
0.04264454	0.0236262	0.208079	0.1408274	0.2201301	0
41.7772	32.08388	295.3411	224.8391	182.2905	90.87733
1.3272	0.7510603	4.086107	2.159732	4.775463	1.917034
1.289936	1.241463	4.286671	3.315529	3.17077	1.151538
2.811114	1.008722	4.660431	1.281382	4.904665	2.23411
3.17528	3.569265	19.76475	8.149673	12.30649	4.056578
1.573263	1.168092	3.788727	2.19194	2.527892	0.9882704
0.3594222	0.2738028	0.4843227	0.2046448	1.692128	0.3342287
1.403939	0.820115	7.461899	1.928785	9.714746	1.56386
1.266943	1.147369	5.275072	0.5604423	1.644298	1.252033
7.97346	9.379002	23.46897	20.9131	28.37448	5.413604
1.539685	1.490286	6.992164	1.333028	5.437934	0.5701133
0.5597298	0.7208908	2.02845	1.04468	0.902464	1.296595
0.881497	1.129873	4.00366	0.9403327	8.724877	0.6586963
0.08349535	0.7863965	0.06632185	0.6940822	0.1676115	0.1242285
0.0635016	0.00552853	0.03963179	0.01136332	0.2527827	0.04568302

TCGA-TM-A7CA-01	TCGA-DU-6406-01	TCGA-HT-A74L-01	TCGA-CS-5396-01	TCGA-DB-5273-01	TCGA-CS-6668-01
0.9680026	0.48131	0.6513035	1.56238	2.525491	1.153397
0.0614721	0.1986168	0.1222391	0.06063288	0.4777957	0
77.59532	590.0547	47.37607	143.5024	460.6927	45.70201
2.84981	3.17945	3.140255	0.9915387	6.305258	0.3457201
2.262321	3.681671	2.644218	1.482966	5.511057	0.8321221
2.380744	5.704438	2.549109	2.353194	5.587359	2.02442
6.681855	12.55828	6.617581	6.514688	18.21397	2.317454
1.326847	3.059635	3.097008	2.83914	4.00013	1.145101
0.2374657	1.551343	0.5908907	0.8432058	0.4143183	0.541819
2.197088	3.663226	9.443944	1.851727	9.666577	0.4259049
0.6614477	9.948768	1.770633	3.898917	3.586551	1.025084
12.23824	32.30909	23.5396	11.46307	42.97842	7.206258
0.6223179	18.41243	1.041514	2.843649	3.456025	0.9214324
1.089696	1.888683	1.489775	1.247472	2.136743	0.4149946
1.134729	4.701996	5.701316	3.277661	4.000563	0.2836217
0.1905677	0.2011445	0.1196682	0.4451828	0.12953	0.2346992
0.02397412	0.02403949	0.05889046	0.1064107	0.09890371	0.01357245

TCGA-DU-5871-01	TCGA-DU-7292-01	TCGA-R8-A6YH-01	TCGA-HT-8107-01	TCGA-DU-A5TS-01	TCGA-DU-A6S3-01
1.491272	0.6297098	1.041325	0.04623264	1.014127	0.2776128
0.1509739	0.03024178	0.03750719	0.1052596	0.01755078	0.009608893
195.8903	108.1744	97.08913	60.92103	187.8116	32.78499
4.59746	1.484159	2.732417	1.279432	3.321633	0.9986193
4.84401	1.4404	3.965481	1.196825	3.144866	1.071272
4.770981	3.229113	1.83764	1.79118	2.587856	1.261022
20.57719	3.65744	9.090816	2.319155	8.659276	2.200101
3.047471	1.097138	2.099094	0.6993555	2.309971	0.5553279
0.5447711	0.6088982	0.7639636	2.31494	0.4376077	0.7339438
8.53491	1.811277	4.527626	5.599247	3.426888	1.026292
2.003309	4.492323	2.078627	2.055983	1.343667	0.9113218
28.86471	13.27878	18.05967	4.963199	19.2045	5.134887
8.319478	3.516859	1.767099	2.170913	0.8952176	1.189766
1.554899	0.7729994	1.46213	0.9086783	1.388061	0.6196759
3.931142	0.8971284	5.428793	1.022978	1.965301	0.521547
0.3029875	0.1052649	0.1631927	0.09016516	0.06872666	0.1881361
0.06623982	0.03145145	0.0146278	0.1462451	0.06160324	0.01124241

TCGA-HT-7610-01	TCGA-DU-A6S8-01	TCGA-P5-A781-01	TCGA-HT-7881-01	TCGA-HT-8109-01	TCGA-FG-8185-01
0.06498447	0.460825	0.3899965	0.01637706	0.1798623	0.6892371
0.0693528	0.009674796	0.01208359	0.01048677	0.02600651	0.07565846
41.08149	75.83255	94.80065	34.79408	102.8636	140.8665
2.950876	0.8908745	2.003749	1.031087	2.847558	1.623655
2.18653	1.525953	1.827555	0.8398502	2.045314	1.953448
2.662008	1.459274	2.077734	0.9028066	3.549478	1.308425
4.749234	3.872732	4.853624	1.772164	5.80196	4.077805
1.163231	0.9182901	1.304943	0.7373031	1.492026	1.279313
0.2922641	0.4280973	0.2630981	0.4014194	0.3209588	0.5911772
5.411979	1.388538	1.707363	0.7992058	1.822842	2.314816
0.9097586	1.456507	0.9757326	0.6410853	1.103768	1.228347
8.408883	10.29676	16.96633	3.233689	13.39708	11.53757
0.8230759	0.8984442	1.47736	0.449154	1.47986	1.355115
0.7850327	0.7077369	0.7172372	0.3818851	0.6275922	0.5734882
1.062143	0.7238196	0.8094939	0.517917	1.111815	1.812803
0.1031992	0.2273118	0.1419535	0.1095063	0.09456408	0.1604791
0.05193138	0.005659755	0.0188504	0.0204492	0.07824243	0.03319515

TCGA-E1-A7YW-01	TCGA-HT-7607-01	TCGA-VM-A8C9-01	TCGA-P5-A5F2-01	TCGA-HW-8319-01	TCGA-FG-6690-01
0.4051289	0.7816921	1.114063	0.2638767	0.04772662	1.253494
0.03185826	0.01714188	1.460711	0.04969672	0.06451748	0.08871428
68.51168	177.249	2120.854	81.87913	133.7109	222.1418
4.531647	1.676703	6.53295	1.416525	3.876699	5.962506
5.514531	2.287401	9.712741	2.164686	3.178256	4.085459
3.115378	3.232243	5.765747	1.495699	2.740318	4.458856
16.35413	6.880298	35.28745	3.950141	8.246334	16.50666
2.841458	1.4665	4.198742	0.9308213	1.678831	3.254098
0.3260501	0.7874019	0.9364742	0.424096	1.352281	0.3827579
8.795291	2.420173	12.46256	1.476269	4.040461	8.354303
2.475433	2.23885	2.59747	1.476459	1.478322	2.05397
30.24797	13.9177	76.6808	7.476238	18.71022	25.06038
2.482693	4.397168	5.628031	1.288733	3.173189	1.070819
2.479459	0.4999396	3.907332	0.9399543	1.562313	1.077548
5.020653	0.6739252	3.189292	1.720514	1.120791	3.079988
0.08019834	0.08726312	0.387982	0.1362243	0.1595636	0.1654257
0.0266244	0.0240672	0.01987247	0.0348871	0.05562086	0.05931184

TCGA-DU-6397-01	TCGA-DU-A6S6-01	TCGA-DU-7294-01	TCGA-TM-A84S-01	TCGA-HW-A5KL-01	TCGA-HT-7874-01
1.279311	0.1017494	1.169193	1.502854	0.411536	0.005430681
0.05119904	0.01002361	0.04701809	0.01859568	0.06800514	0.1078007
207.9919	25.19855	26.07701	141.3439	71.23504	39.62036
0.6064307	1.179594	1.061306	2.124422	3.647449	1.251608
1.943772	1.199804	1.231557	3.262455	3.434551	1.226187
1.855751	1.090944	2.465633	2.821103	3.590682	2.261119
4.183217	1.988623	3.701756	7.908538	10.22714	3.160488
1.308233	0.4101582	1.330435	2.678911	2.438463	0.8948399
2.381078	0.707539	0.1524169	0.3428479	0.2328502	1.069779
0.653033	0.5185653	2.752352	2.769732	3.582043	2.100807
1.978673	0.8303906	0.793493	1.439599	1.403589	1.045047
10.44483	3.80357	13.95164	19.20659	23.97298	5.809161
2.836508	0.8898563	0.5787944	2.052702	1.156804	1.173924
0.7638532	0.675365	0.4664905	1.096313	1.376649	0.6543696
3.723159	0.3014369	0.4509817	1.029787	2.163563	0.9947476
1.217255	0.1668176	0.04248853	0.2366596	0.0915405	0.06808615
0.0727392	0.03518287	0.05924279	0.01087847	0.04475588	0.1464699

TCGA-HW-7495-01	TCGA-FG-A4MY-01	TCGA-12-1597-01	TCGA-DU-7304-02	TCGA-HT-7605-01	TCGA-HT-7902-01
0.3737104	0.9070545	2.826523	0.2817793	0.7249704	0.0510723
0.03778405	0.2212635	1.20661	0.01933205	0.01762869	0.1129749
30.26928	111.3831	402.7724	110.9538	74.00168	119.2314
1.273633	1.104317	1.997775	0.1871234	1.697377	2.683094
1.048577	2.653257	4.202823	0.9857495	2.828724	1.916786
1.768929	1.805163	4.892299	0.3472925	2.539707	1.972671
3.072248	7.085755	15.89373	0.948067	5.058074	6.519371
1.098028	1.872623	7.419141	0.663288	1.090704	1.404662
0.6590277	0.8450243	1.316155	0.3258738	0.3002562	0.1941965
1.527355	2.13476	34.37447	0.3441314	2.044667	2.381536
2.045998	1.070581	3.641864	0.7437011	0.9803293	1.245067
8.257539	26.51723	48.38319	6.25059	12.93021	8.117317
0.9513709	6.146482	2.34908	0.1656005	4.252284	1.739876
0.6505913	1.251221	9.845538	0.6326643	0.89366	1.283445
0.8252568	2.028655	15.00072	0.397031	1.168037	0.9943782
0.0246596	0.3249151	0.4891977	1.085061	0.1725794	0.2561238
0.05403119	0.09707935	0.1782492	0	0.02062557	0.1078316

TCGA-VM-A8CE-01	TCGA-TM-A84O-01	TCGA-HT-7467-01	TCGA-QH-A6X4-01	TCGA-S9-A6U8-01	TCGA-DU-5854-01
3.501153	1.927423	0.3964586	0.3463183	0.4091102	2.610731
0.06742564	0.05262057	0.008753978	0.01209594	0.04058637	0.1098794
57.9199	290.2779	83.69487	101.8541	115.4701	473.7577
1.928944	2.569839	1.750417	1.780877	5.802573	4.639126
5.178944	3.046961	1.374413	2.048954	5.848217	5.446785
3.306424	4.741044	2.983886	1.919001	6.117874	7.594535
5.842972	7.91539	4.443591	6.401931	28.94429	30.51966
1.262173	2.022023	1.230948	1.078359	5.058555	6.217837
0.8923857	0.3729461	0.3627585	0.1316835	0.6608272	0.8847557
5.281565	1.700435	2.201102	1.002587	8.811398	7.347991
2.518414	1.992419	1.332052	0.7809233	1.882475	4.185938
15.10145	21.11674	8.753288	9.161771	35.64007	50.52385
4.110302	3.252131	0.8180458	0.5745931	2.228231	3.868858
2.299877	0.9001731	0.9170305	0.8285766	2.890869	2.460872
2.200764	0.9824473	0.5950006	0.6801968	3.566904	2.34875
0.8580987	0.3184495	0.1428312	0.05526056	0.101138	0.3303889
0.0049305	0.02798458	0.05803887	0	0.09928903	0.05968805

TCGA-DB-A75P-01	TCGA-CS-6186-01	TCGA-QH-A6XC-01	TCGA-TQ-A7RQ-01	TCGA-HT-7469-01	TCGA-DB-A4XB-01
0.3804303	0.5629031	0.160893	0.2446798	1.123621	0.8075232
0	0.07645808	0.1287813	0.0104451	0.1709288	0.0904896
341.0257	219.6977	412.3836	23.09126	96.0663	197.1917
2.545152	2.145093	2.368408	0.7715729	1.916743	3.378426
2.726022	3.242565	3.891739	1.697098	2.91326	2.50815
5.403139	3.191756	3.890893	2.266325	4.97743	2.456509
7.468588	9.833124	10.82673	1.689727	7.607846	10.9708
1.187089	2.833714	2.611274	0.6932484	3.387301	1.750497
0.4601904	1.179507	0.3979842	0.6272474	1.105611	0.4153861
1.54904	1.357998	3.460162	0.8134614	7.522766	5.123679
2.466813	2.221599	2.099403	1.247237	2.505029	1.395895
12.071	21.97424	16.17351	3.549177	18.51127	10.24604
1.780908	2.436918	4.272218	1.033016	1.044748	1.019264
0.9641788	1.198524	1.7106	0.6635489	1.727512	0.8523297
1.478708	1.241782	0.9319911	0.8376325	1.457825	1.355892
0.2384787	0.0320786	0.1512875	0.1772407	0.1712255	0.1518626
0.009828079	0.01916913	0.02008989	0.01222077	0.06046111	0.02268704

TCGA-DU-7010-01	TCGA-P5-A735-01	TCGA-HT-7479-01	TCGA-FG-8182-01	TCGA-27-1835-01	TCGA-S9-A6TZ-01
1.55444	0.04705197	0.8033277	1.37178	0.2885347	1.147615
0.1178714	0	0.0253576	0.0613726	0.2166131	0.04184231
209.9841	49.41028	184.2126	176.1478	173.544	133.7893
1.718899	3.932412	6.627075	7.788141	0.9201428	5.047969
2.060032	3.478776	5.040487	6.961622	2.103321	3.378885
3.410019	2.349181	5.200243	5.321157	4.378901	3.644305
5.953638	10.98382	15.20889	18.33477	3.765512	14.08749
1.745857	1.814961	2.869863	3.213582	1.182535	2.182114
0.7071496	0.05713569	0.4999572	0.5374742	1.691444	0.7934865
3.857699	3.529703	9.835925	5.268318	0.6060357	6.30203
2.263506	0.8641269	1.904093	2.500521	9.926158	2.640113
22.70199	17.29119	40.7338	34.95	16.79401	28.0088
3.71753	0.8305727	3.715239	1.4786	1.733979	1.979488
0.8572181	1.899508	1.766062	1.801735	1.290845	1.193137
1.244011	1.140306	5.733897	5.311839	1.813117	2.927107
0.1538566	0.02359621	0.1985944	0.1577149	0.2869012	0.2918565
0.09960135	0.04935116	0.06357512	0.0628302	0.007454038	0.06425409

TCGA-DB-A4XD-01	TCGA-P5-A737-01	TCGA-DU-8163-01	TCGA-S9-A7QZ-01	TCGA-S9-A6WI-01	TCGA-S9-A7IY-01
1.44477	0.2166772	1.101359	0.07152758	0.05877654	0.7029254
0.03101007	0.06937273	0.1719013	0.02914637	0.06843007	0.1091166
44.9232	83.90698	213.7329	38.03695	60.62837	242.8466
2.815974	1.890771	7.989451	0.9672701	1.095516	5.027312
2.447758	2.286284	6.734935	1.03997	2.211847	4.750535
2.264507	2.59609	6.750093	2.319783	1.853557	4.550547
7.320766	7.419865	25.52936	2.169253	3.469208	17.78524
2.049438	1.338373	3.369212	0.9028283	0.441666	2.773343
0.2014673	0.3362003	0.4690157	0.570271	0.9300111	0.2394976
3.812337	1.983573	15.96953	0.715717	2.466712	4.673885
1.349454	1.19962	2.483029	1.515361	0.9363599	2.374194
17.43018	12.9804	38.30065	4.057484	5.208675	22.70361
0.7848325	1.210118	2.708211	0.539873	1.538721	3.775995
0.9999169	1.124023	2.615556	0.5971579	1.034101	1.855504
2.054656	1.0923	2.489394	0.3787011	1.711551	2.827899
0.04047718	0.1539378	0.3624622	0.1059812	0.08708818	0.3115635
0.03023481	0.02164429	0.09798375	0.009743216	0.0200158	0.122347

TCGA-DU-A7TI-01	TCGA-TQ-A7RV-02	TCGA-S9-A7QX-01	TCGA-HT-A74H-01	TCGA-QH-A86X-01	TCGA-RY-A845-01
1.115068	0.4802024	1.645693	1.806249	0.6298642	0.7723244
1.214279	0.1537446	0.04178026	0.3885104	0.02901601	0.08845511
418.3016	261.1147	193.2313	369.0335	123.2393	77.61434
5.160851	3.429135	3.437479	3.852699	0.6784953	2.937283
7.263497	5.182045	3.979943	5.28738	1.762908	2.832008
7.87826	3.302246	4.168724	4.266423	1.697821	2.912651
16.89254	12.17265	12.04536	18.76576	4.328077	6.870546
2.536263	1.87274	4.296559	3.861827	1.25341	1.838591
3.852252	0.9009931	0.8656719	0.5692725	0.5869356	0.4193599
30.26774	7.304425	7.049976	5.149666	1.249324	4.153446
2.322924	1.01839	1.734589	1.497655	0.7708697	1.821645
34.61685	21.64021	27.53861	41.25041	7.987461	16.70139
9.992593	1.537739	0.8296639	5.36761	0.9964769	1.432459
3.753685	1.861368	1.365074	1.470029	0.5097028	1.282922
30.54228	6.822503	2.138339	5.325892	0.5618625	1.88742
0.3472727	0.197546	0.1726955	0.2448163	0.4658547	0.07872254
0.1489879	0.06745546	0.02715717	0.03657357	0.006789747	0.02822522

TCGA-HT-8111-01	TCGA-TQ-A7RN-01	TCGA-DU-A5TW-01	TCGA-R8-A6MO-01	TCGA-DU-8162-01	TCGA-DU-6394-01
1.374072	0.6572086	0.4462871	1.055627	0.1292894	1.065764
0.08313666	0.09017831	0.07144304	0.06562645	0.1517784	0.2790435
375.5961	77.00652	19.78079	149.6996	113.3758	88.26191
5.990369	1.319522	2.682922	1.391648	2.533193	1.06386
5.269028	1.319141	3.19747	3.317961	2.638378	1.522989
5.275797	1.741576	2.521648	2.923649	3.812302	2.685484
15.65764	3.258873	8.651876	7.814023	6.518462	4.456583
3.729216	1.10743	3.801703	3.050816	1.295099	1.381858
0.1897737	0.5067016	0.1612889	0.466696	0.9885022	0.5336445
10.75834	2.842668	4.045229	3.438952	3.356166	2.348658
2.491709	1.370103	1.428625	2.009713	1.830894	7.208879
33.89605	6.194396	23.14535	19.49482	11.55598	9.588868
2.885048	0.8075898	2.324763	0.6630972	2.828633	3.57839
1.600438	0.6043835	1.387608	2.297726	1.022644	0.8743743
1.66168	0.8770205	1.796642	2.998854	1.801464	0.5695246
0.2509469	0.1569453	0.2098215	0.2184372	0.06078531	0.3503765
0.04863494	0.01953863	0.06567657	0.03455232	0.04843111	0.04613318

TCGA-KT-A74X-01	TCGA-DU-8166-01	TCGA-DB-A75L-01	TCGA-P5-A780-01	TCGA-VM-A8CB-01	TCGA-WH-A86K-01
1.662882	0.3124035	1.454466	0.7255603	0.2688963	0.4508239
0.09485267	0.2822023	0.04923393	0.3839669	0.02295775	0.005551486
80.80553	146.9709	122.8163	156.8225	29.49685	230.9267
0.5471311	3.698796	3.091348	2.711149	0.5263058	2.68959
1.71356	6.464675	3.73023	4.695553	1.314473	3.627167
1.586916	5.210496	3.336855	3.241074	1.799679	1.93891
3.825414	9.767078	7.120657	11.15628	4.525434	7.27654
1.38627	1.0458	2.492306	2.079766	1.050787	1.747043
0.6834003	3.458601	0.5057322	1.201439	0.3204768	0.8129718
0.6638177	16.41184	6.128073	8.695434	1.743244	2.603354
2.13274	2.598078	2.276108	2.305615	1.085394	1.484381
14.99074	20.30849	26.54959	31.39889	7.886808	23.25956
1.837102	23.36287	1.00164	2.834608	0.797808	2.833824
0.8172732	1.841236	1.461172	1.528219	0.8452343	1.218317
1.140092	7.1739	2.371362	3.492238	0.7324988	1.02612
0.964523	0.3427113	0.3614886	0.4285164	0.2584615	0.1195641
0.01431969	0.2549466	0.03360216	0.09883308	0.02014542	0.03247619

TCGA-TQ-A7RV-01	TCGA-DU-6405-01	TCGA-CS-6667-01	TCGA-HT-7472-01	TCGA-FG-8188-01	TCGA-DU-A7T6-01
0.7447958	1.400572	0.3157872	2.829251	2.422095	1.231018
0.01644542	0.2452275	0.06385542	0.4905854	0.06439916	0.04692029
97.13465	438.5917	59.59608	419.2747	82.05623	120.1824
2.563673	4.526916	2.645835	6.051774	3.227463	0.858127
2.231419	3.387956	2.688818	4.142946	3.724355	1.351687
2.103537	4.835328	2.66672	5.812766	3.850088	1.749299
7.588459	15.18788	5.34803	16.12482	12.44828	4.474552
1.696209	4.146813	2.011315	3.488624	2.929468	0.8550668
0.5371035	0.627439	1.872468	0.4725503	0.4636222	1.12706
2.634715	4.168486	4.410615	6.666672	4.469069	1.743546
1.096963	4.887729	2.989562	1.619666	1.950053	4.233746
13.20084	30.23675	21.60208	31.29911	31.73915	9.565928
0.4562372	8.32756	1.810877	3.065848	2.068688	1.227696
1.003181	1.47525	1.090969	1.010183	1.743368	1.284118
1.067521	2.2458	1.883876	3.413289	3.054563	0.8718493
0.08854752	0.1577603	0.1875374	0.6998188	0.09456715	0.4470861
0.04329256	0.01639521	0.1411205	0.06149839	0.04395242	0.0219587

TCGA-HT-7877-01	TCGA-CS-5397-01	TCGA-HT-7856-01	TCGA-DB-5275-01	TCGA-TQ-A8XE-01	TCGA-HT-7884-01
0.04079577	0.6038184	0.2353194	1.586796	1.49325	0.32345
0.2938822	0.1251992	0.03847218	0.2854532	0.08278586	0.04602569
31.87199	316.443	37.29066	102.9126	140.5662	27.31487
1.49467	5.838865	1.35236	1.726891	2.479876	1.764425
1.405389	4.391757	1.435264	6.346685	3.165964	2.701551
2.381477	6.260342	2.436931	3.134865	2.259793	1.680511
3.152828	15.18328	5.090473	6.713789	8.797367	4.177202
1.151576	4.352576	1.060321	1.780462	1.854412	1.14877
1.572166	0.4538998	0.7363317	1.936887	0.3183468	0.2424498
2.937224	12.03242	1.47135	6.79407	4.130903	1.766631
2.410369	2.56879	1.324324	1.78039	1.381113	0.946658
6.495985	38.31416	8.609487	23.15004	23.66275	13.38291
0.8238851	2.955028	1.09353	7.764377	0.9670265	0.9811724
0.7668969	1.628638	0.8038772	2.281478	1.637512	1.146297
0.9005482	2.290377	0.9500256	6.545438	2.307428	1.389743
0.1278673	0.0504684	0.1318207	0.8649635	0.2431343	0.09762505
0.2406895	0.08185819	0.04876349	0.08747101	0.03874378	0.02243752

TCGA-WY-A85D-01	TCGA-S9-A7IQ-01	TCGA-HT-A614-01	TCGA-E1-5322-01	TCGA-FG-5965-01	TCGA-HT-7860-01
1.79728	0.03298193	0.8610409	2.715156	0.645754	1.194948
0.06110744	0.003519901	0.0477132	0.2516399	0.8141731	0.2707504
108.367	22.10512	174.3238	516.7529	196.0536	370.4519
3.747372	1.100123	2.487437	5.706405	2.792387	5.802147
4.673841	0.7179249	4.311439	7.798152	4.343758	5.802386
2.997483	1.820634	2.998163	7.001643	4.255967	7.635065
13.37525	2.506355	10.52792	23.18282	8.827526	20.13056
3.269513	0.5404897	2.39296	5.889461	2.655706	4.717593
0.06437919	0.4932119	0.6981641	0.6300736	2.114019	1.045905
5.74483	1.497919	2.565734	23.64261	12.14301	8.303419
1.070658	0.8205044	1.184304	2.007185	3.38435	6.326556
41.91658	2.766171	17.18873	66.62775	21.74994	33.18035
1.683377	0.4358314	0.9970206	3.439503	3.396037	5.656104
2.085589	0.6115389	1.390526	2.744546	1.849646	2.115069
2.599624	0.3201404	1.290989	4.755747	7.777444	4.938849
0.05982228	0.04824222	0.2179788	0.1215741	0.4518709	0.2612148
0.03574785	0.0247097	0.0372163	0.2791242	0.4537893	0.1010017

TCGA-DB-5279-01	TCGA-FG-5965-02	TCGA-S9-A7J2-01	TCGA-E1-A7YO-01	TCGA-HT-A5R7-01	TCGA-HT-8012-01
0.2507512	0.02333833	0.6889226	0.1303032	0.152626	0.8967932
0.407833	0.02490716	0.02520798	0.05708873	0.08744391	0.02364542
78.50674	36.15324	24.36813	36.37319	144.7447	79.76059
1.99261	0.3692441	1.560309	0.5637726	1.426839	1.117698
1.487579	0.5338415	1.942572	1.02852	2.485015	1.926768
1.807101	1.108739	2.499503	2.747846	2.790167	2.742552
4.080297	1.015253	4.209067	2.654261	5.107367	6.273456
0.9699452	0.5127428	1.074026	0.8447485	1.340987	1.584563
1.18751	0.6035363	0.71263	1.873758	0.4389521	0.3736713
4.351633	0.3657838	3.042945	0.9453973	4.128985	2.249259
1.864072	0.683055	1.204983	2.65952	1.266625	0.9244317
10.25318	3.44497	10.50879	4.693475	10.14041	15.09325
1.256625	0.6691664	1.555708	1.436307	1.970773	1.957099
0.5826481	0.3787892	0.7258847	0.6129026	1.366489	0.9282641
3.745106	0.3288403	0.9244776	0.7343959	2.34671	0.8423961
0.4904244	0.05851999	0.1069372	0.206356	0.1812809	0.2910051
0.3343909	0.005828274	0.03932445	0.01541396	0.0120364	0.01185649

TCGA-CS-4938-01	TCGA-DU-5853-01	TCGA-HT-7686-01	TCGA-DU-A7TA-01	TCGA-HT-8104-01	TCGA-HT-7677-01
1.441422	1.061182	3.078676	0.8180365	1.604728	1.005018
0.2432985	0.1889759	1.614356	0.1925134	0.2400841	0.2462548
79.36889	316.6407	672.5138	598.9902	244.1671	45.75298
3.328861	5.754851	3.831388	3.78044	2.218692	1.435182
3.28087	5.295782	10.98383	4.937188	2.763784	1.895555
3.492282	5.940797	13.11358	5.010589	4.830574	3.180588
9.039129	20.59155	41.12724	16.14765	11.11956	3.801785
2.052698	3.724745	8.944833	2.882057	3.281436	1.351851
0.174952	0.9234567	0.3475172	1.551814	1.811039	1.124238
5.252598	7.542104	22.44668	10.91592	6.96088	3.572635
1.299584	2.308687	3.18827	2.181885	5.025845	1.390055
36.57514	28.32971	86.31472	31.10236	27.67218	10.66037
2.070588	1.737771	4.628219	1.621199	9.991271	2.43545
1.195078	1.776387	2.793984	2.036154	1.925744	1.110875
2.787292	4.022642	4.668079	9.050239	1.760969	1.859213
0.07561322	1.094264	0.2431391	1.349932	0.1065491	0.2892909
0.02259201	0.2822576	0.3904723	0.08381047	0.03745312	0.1037225

TCGA-06-2565-01	TCGA-28-5209-01	TCGA-DU-7015-01	TCGA-FG-A4MT-01	TCGA-E1-A7Z4-01	TCGA-P5-A5F1-01
2.124749	0.827764	4.312713	0.8858321	1.254472	2.846466
0.6297121	0.9427837	0.0542075	0	0.01673498	0.1652434
502.6678	248.596	333.6203	80.37815	336.8875	228.6222
1.141544	2.17461	6.772747	2.532294	4.379994	2.10073
3.396442	3.000099	5.561961	2.760729	5.358584	3.154818
6.659471	9.29599	8.007631	2.533571	3.853621	2.565484
10.88139	9.872962	22.75368	8.793981	17.27474	9.312439
5.091805	3.207347	4.647144	1.41681	3.406263	2.53482
2.188827	4.046281	0.2125755	0.3066617	0.2556493	0.7069139
4.287962	11.90538	21.40485	3.188607	5.781136	2.908085
15.79601	15.41219	1.827266	1.62891	1.164085	1.321729
42.16969	40.24965	46.67049	18.13223	48.58524	25.18482
3.844295	4.471059	3.778099	0.7236086	2.047677	2.380602
3.645567	5.346499	2.000103	1.408043	2.029608	1.751988
3.533224	5.946376	2.067527	1.291945	2.853886	2.18165
3.050844	0.4508448	0.1415132	0.07306549	0.07372361	0.6176603
0.129068	0.1779124	0.2114093	0.03492926	0.03915986	0.04101042

TCGA-HW-7489-01	TCGA-HT-A4DV-01	TCGA-RY-A843-01	TCGA-QH-A6CW-01	TCGA-E1-5311-01	TCGA-CS-6290-01
0.4020561	0.726196	0.3588581	1.639258	0.3678544	3.082191
0.06164288	0.0430562	0.007412533	0.1402785	0.05485392	0.2029335
156.3752	238.0925	207.469	167.3703	19.10125	630.239
2.446709	2.145216	2.894511	3.05324	0.8630073	9.119008
2.381684	2.307087	2.882808	7.692147	1.165659	10.54368
2.347597	2.139646	2.544799	4.100013	2.276497	10.29072
6.004511	7.859271	5.330045	16.35289	3.074402	32.16756
1.563293	1.707337	2.073174	3.522002	0.9283221	7.553558
0.6876349	0.2993857	0.8629395	1.829528	1.244203	0.2431436
3.291929	2.931659	3.426605	7.006975	1.976252	13.01539
1.097994	1.195535	2.562196	3.636967	2.250944	2.72352
14.98071	13.68925	15.06246	24.13336	10.12853	66.40025
1.10973	0.5733539	0.8831795	3.904392	0.9762042	3.54642
0.7085107	1.031933	1.111248	3.335998	0.8447809	2.642707
1.239391	0.8084703	1.859436	7.883619	1.382165	3.72411
0.1135933	0.1348822	0.0943363	0.8618535	0.08844751	0.2726782
0.05515225	0.0201503	0.013009	0.06791416	0.04907812	0.09311065

TCGA-DU-7301-01	TCGA-WY-A85C-01	TCGA-S9-A6WN-01	TCGA-S9-A6U5-01	TCGA-HT-7476-01	TCGA-06-5417-01
8.833275	1.014177	4.264794	3.477262	0.5996691	1.401658
0.2781755	0.3025205	0.5589783	0.02875688	0.05937954	0.4010228
375.3006	162.961	855.7751	379.928	147.2732	211.1098
3.168743	1.809336	4.789974	1.500581	2.47752	1.707353
4.096613	3.353553	4.694492	4.672359	2.346963	2.178514
4.890872	3.015176	8.150328	2.938614	4.713003	2.967226
11.86564	6.378823	28.79353	12.42557	7.102361	6.032562
4.03363	2.239262	3.510055	2.849057	1.923772	2.642282
3.255022	2.889707	4.904558	0.7271173	6.330948	0.5633253
21.3795	4.61107	28.2063	1.693395	3.032326	5.385848
2.449659	2.16857	19.00091	1.59369	8.363783	4.23323
39.20799	15.23351	27.76532	27.46939	14.41707	31.81517
11.20821	2.407668	28.43554	3.647031	2.432279	1.851442
2.297325	1.189982	4.591789	1.302913	1.063081	1.589946
19.71262	2.786973	8.681975	1.566879	1.631812	2.465193
0.6753662	0.402775	0.3926634	1.174344	0.1782675	0.7851777
0.2299955	0.09910571	0.2596049	0	0.01389481	0.0670281

TCGA-DU-6395-01	TCGA-HT-8019-01	TCGA-E1-A7Z3-01	TCGA-DB-A4XE-01	TCGA-P5-A5EZ-01	TCGA-S9-A6WO-01
1.259981	0.03511065	0.2984955	0.0479958	1.888826	0.439547
0.2584576	0	0.04526915	0.1024443	0.08893211	0.1001794
58.21871	49.14735	183.7767	120.5297	393.711	157.3426
2.906519	1.202625	3.659185	3.268374	3.251833	6.441443
7.79382	0.7772148	3.247259	3.209462	4.158076	5.66477
4.370921	2.011576	4.390097	2.721118	3.597778	4.958306
12.91309	2.405604	10.47878	10.47534	10.36526	18.41204
1.835825	0.5722126	1.846012	1.620595	2.438527	3.272164
1.56754	0.9435015	2.071992	0.1942726	0.7401797	0.1206206
10.77152	0.8191853	7.01752	4.68442	3.48774	9.266795
1.287086	0.800673	3.790486	1.0272	2.121316	1.697999
36.87404	3.5673	15.96944	15.98649	29.7726	30.37706
25.36417	0.398306	2.896602	1.035109	1.65692	1.515689
3.082786	0.5392012	1.701773	1.469232	1.114943	2.213209
7.521457	0.5496822	1.933212	0.6086408	2.465692	3.268092
0.6564893	0.08803865	0.1674201	0.1002897	0.6877879	0.158784
0.02860497	0.01315226	0.06473488	0.07191587	0.06763287	0.106047

TCGA-S9-A7J3-01	TCGA-RY-A847-01	TCGA-E1-A7YD-01	TCGA-DU-6407-02	TCGA-TM-A7C5-01	TCGA-VM-A8CA-01
1.07107	0.4227971	0.684515	3.616819	1.139488	0.5203762
0.07686148	0.03575689	0.2560955	0.2082882	0.06030176	0.2768238
109.9599	217.6653	243.8603	533.4777	47.84756	246.6495
2.010542	5.511682	2.047317	3.36903	0.6543438	2.503215
3.464429	3.62003	3.758338	4.123131	1.396694	3.827895
2.915374	4.322509	4.305355	4.286771	1.865521	2.332228
9.937453	13.58278	9.620169	20.20199	2.350454	7.509445
1.676063	2.60737	2.189751	3.686184	0.9920879	1.747337
0.3861967	1.232391	0.5396142	1.165123	0.8703661	0.6156955
2.772613	9.198157	2.3424	9.600439	1.338441	4.825101
2.12981	1.859052	3.182376	1.484694	0.923326	2.767782
15.04097	19.77332	26.42878	44.22881	9.04455	20.97194
4.924216	1.509609	1.369171	1.604639	0.718476	2.131777
1.63756	1.430774	2.002819	1.56569	0.4701445	1.297723
1.756341	2.203066	3.149949	2.044279	1.910742	3.301358
0.2836157	0.2500349	0.2217815	0.3107165	0.2479409	0.6122635
0.0518815	0.07171811	0.04609719	0.04641852	0.03527653	0.0359871

TCGA-DH-A7UU-01	TCGA-HT-8108-01	TCGA-HT-7687-01	TCGA-HT-8564-01	TCGA-HW-8320-01	TCGA-HT-8113-01
1.086871	1.485739	1.822509	0.363311	0.6007223	0.02017881
0.05567671	0.09232677	0.1071746	0.007886099	0.1083029	0.5211532
111.0751	367.0616	160.2009	341.6948	148.6766	29.27956
3.595158	5.822154	1.480252	4.058705	4.357816	0.9840992
4.106094	3.996583	2.452848	3.193069	4.202326	0.9678096
3.091556	7.083053	3.500632	4.697206	3.230788	1.959482
10.70018	16.99145	7.149535	12.58926	11.61922	1.794023
2.278248	2.973582	1.517092	2.05592	2.240254	0.585048
0.3666106	0.3044975	0.8823928	0.6231246	0.7200202	1.320458
5.133617	4.883655	3.126711	3.44153	9.629157	2.256978
1.699185	1.889806	1.592046	4.733131	1.860583	0.6004269
23.23153	34.9533	18.21753	18.23812	24.36791	4.874063
1.452477	0.7221089	1.557936	1.423223	2.9228	1.393612
1.920336	0.9678854	0.706832	1.136699	1.410906	0.5638134
3.328278	1.360286	2.131216	1.613819	5.423701	1.377384
0.09992724	0.18077	0.7616457	0.3705717	0.2193624	0.2867199
0.04342784	0.1596852	0.06501925	0.009226735	0.113606	0.161256

TCGA-FG-8189-01	TCGA-DH-A7US-01	TCGA-S9-A7R2-01	TCGA-HT-A74J-01	TCGA-26-5133-01	TCGA-DU-6404-01
0.03611131	1.05266	1.578476	0.1553201	2.097869	1.343277
0	0.004348725	0.7681696	0.05189036	0.1221213	0.5142498
29.23162	101.7376	813.8164	137.2622	241.2592	250.0024
1.014258	1.531971	7.248789	3.942154	1.290939	2.381224
0.7913718	2.033274	7.73078	3.991289	2.29995	2.685143
1.485728	1.65276	7.081503	2.242702	2.26865	5.144047
1.888023	4.949449	29.16808	10.47099	5.930639	8.716454
0.4018848	1.845506	6.51512	2.360453	2.429865	2.014397
0.8136671	0.1511915	0.2875138	0.6059105	1.903093	3.863431
1.034192	0.8200835	7.885745	1.883491	3.515589	8.255128
0.6367753	1.125513	2.231944	2.191388	6.212622	5.95487
3.032091	17.63438	47.25032	13.26284	21.45637	20.28905
0.5672184	1.513772	4.651809	1.474927	0.9688352	13.60152
0.5163608	0.9836628	1.770644	1.629497	1.290557	5.785117
0.2713671	1.282261	8.592416	1.573169	3.0455	5.06066
0.0362191	0.4086976	0.3562172	0.09313157	0.816945	0.2936701
0.01082168	0	0.0354773	0.03541517	0.02381365	0.1181856

TCGA-E1-5319-01	TCGA-TM-A84M-01	TCGA-TQ-A7RK-01	TCGA-TM-A84T-01	TCGA-CS-6666-01	TCGA-DU-7019-01
2.738115	0.5760245	1.442111	1.091159	2.314222	2.715424
0.05943405	0.0793722	0.02841326	0.009772091	0.04645366	0.1962578
91.8662	80.46671	52.23551	97.46784	127.6441	231.1158
1.223028	0.5708568	2.059064	3.97271	2.211544	3.088928
1.632691	0.9603577	2.545639	3.272621	2.308471	4.020165
1.827386	0.9574916	1.690394	3.525852	3.947176	3.672695
7.230012	1.019962	7.871919	15.00877	9.041565	9.1028
3.463533	0.4963358	1.390384	2.312629	2.825796	2.355908
0.3801699	1.731465	0.3841599	0.5044696	0.646019	3.395309
0.9564421	0.3350469	2.924079	5.968789	4.008004	8.998318
1.967064	0.8616131	0.7575619	1.513028	5.159487	3.300914
21.82847	3.836906	21.32953	27.05398	23.53096	26.81091
1.452972	1.036228	1.548852	1.19285	4.297613	8.401805
0.7866009	0.4831082	1.114462	1.878844	0.7757751	1.123487
1.961717	1.739689	1.333796	2.171793	2.609979	4.712507
1.36317	0.5027835	0.1668942	0.3922295	0.6821503	0.4449331
0.01490097	0.02731337	0.01662176	0.04001671	0.04348063	0.09265432

TCGA-TQ-A7RR-01	TCGA-E1-A7YV-01	TCGA-HT-7477-01	TCGA-HT-8011-01	TCGA-HW-8322-01	TCGA-HT-7879-01
0.7415174	0.1624808	0.9453872	2.350897	2.741067	1.558082
0.08958827	0.7472091	0.08902387	0.06256981	0.0683842	0.1084446
372.7908	60.65903	200.8441	268.4227	152.5159	137.2788
4.908604	0.9938148	1.438813	0.4809497	1.015276	4.394856
6.678006	1.651213	3.346819	3.619885	2.321812	3.948848
4.009956	1.913202	4.423975	3.225285	2.4228	3.289107
13.04075	2.777067	10.77284	7.231717	4.739068	13.2588
2.241101	1.145124	1.825149	2.568071	1.695146	2.705179
0.2406815	4.170249	1.899253	1.074106	1.163021	0.4493867
8.955618	3.10956	4.241605	1.861128	4.896422	3.420483
1.42038	4.633824	3.519677	8.354583	1.285597	2.461803
24.21911	7.220621	16.4308	23.2387	13.29263	21.96318
3.453403	1.46943	2.703722	2.300127	1.570973	1.786115
2.382154	1.026474	1.435283	1.24171	0.669192	1.724038
3.311845	2.122877	2.024215	1.031259	0.8885212	2.34914
0.07893371	0.3425987	0.4270428	0.7062213	1.06476	0.1557071
0.03144548	0.2268584	0.1145737	0.0344502	0.1828789	0.05921077

TCGA-HT-7676-01	TCGA-FG-A87N-01	TCGA-TQ-A7RW-01	TCGA-FG-A6IZ-01	TCGA-S9-A6WH-01	TCGA-DU-6392-01
0.4475652	1.77252	2.634093	1.730803	1.38703	3.376656
0.1770119	0.4931641	0.04977135	0.02289855	0.06876084	0.3149998
198.7624	211.5463	570.0491	137.6084	53.84907	411.7655
4.86315	2.748855	7.70391	1.154887	1.204149	2.390762
6.825889	3.068116	5.639693	2.22439	1.772881	4.501157
7.955764	3.129418	6.750427	1.695671	1.524023	5.972073
27.06264	12.74858	21.94648	6.19239	5.357428	19.45263
3.929171	2.579085	4.003223	1.727154	0.8798668	4.125789
0.3330165	0.9092451	0.7690636	0.2943197	0.2414746	1.562666
23.89276	14.29993	7.309365	4.409927	1.874276	12.27455
1.938381	3.035152	2.436024	0.748332	0.8315416	7.452163
54.11003	19.86657	30.09886	14.62238	10.98749	26.97274
2.239993	3.744441	1.104626	0.5724057	1.539465	5.558591
3.091181	2.523523	1.825776	1.329051	1.12514	3.555956
1.323077	3.989626	3.342354	1.444424	1.109564	3.719169
0.1237779	0.1561975	0.173243	0.5021401	0.280478	0.6287256
0.2465522	0.1230372	0.04529192	0.01071652	0.1072669	0.1502824

TCGA-28-5220-01	TCGA-FG-A6J1-01	TCGA-14-1034-02	TCGA-HT-7880-01	TCGA-HW-7486-01	TCGA-FG-A60J-01
2.765683	0.6759108	2.655409	0.009517002	1.08586	2.198081
0.3912579	0.142572	3.250027	0.02437619	0.04257009	0.07585346
594.4425	101.8919	128.4662	101.6676	110.1143	263.6032
2.454832	2.101152	1.359653	2.92451	2.266299	3.320085
3.968725	3.538089	1.674129	1.882862	2.67041	5.189844
11.13772	2.541052	2.470596	2.060159	3.714521	3.188427
18.21722	7.439433	8.211209	6.10721	11.09819	12.0509
6.263259	1.525071	2.330424	1.272526	2.142999	2.378562
0.5749193	0.8154008	1.095994	0.3563282	0.1457603	0.515006
17.35476	6.73006	3.184843	1.332277	6.151152	6.371603
2.826593	1.143297	4.693367	1.251685	1.402554	1.064181
67.87599	16.34906	20.32959	7.388905	21.8728	23.97673
1.136563	0.7315827	18.11868	0.8328635	1.20172	2.072369
1.655055	2.176083	9.029149	0.9986408	1.219012	1.409529
1.570838	4.474261	4.220379	0.7330574	1.451931	4.685872
0.1411161	0.5084464	0.7023564	0.1431812	0.1111328	0.5280581
0.1626294	0.06553219	0.1846704	0.04278021	0.07886109	0.02465237

TCGA-DB-5280-01	TCGA-FG-A60L-01	TCGA-DU-A6S2-01	TCGA-FG-A4MW-01	TCGA-32-1970-01	TCGA-HT-7873-01
3.076567	1.072954	0.06284757	1.038277	1.403702	0.1116332
0.04236616	0.2071247	0.5142206	0.1228514	0.25612	0.1322424
101.5994	243.7153	27.73457	163.7778	371.2522	24.99194
3.380466	4.30438	1.551298	1.689819	3.205347	4.38907
2.627106	9.055102	2.005652	1.811197	4.143009	4.670435
2.651462	3.984922	1.602395	2.291275	5.912307	4.257713
8.715492	19.29856	3.163997	9.057878	16.11527	14.1498
1.671038	3.968106	0.5583402	2.060788	5.51634	3.528096
0.5058575	0.2501482	2.793558	0.700439	0.758587	0.2522872
2.816329	11.62599	3.52964	3.931572	6.185583	7.479699
1.639905	1.743313	4.662349	5.441727	4.682565	1.76226
17.96298	36.18422	4.999902	16.12865	28.86223	24.29995
2.185727	1.410354	10.58736	2.287613	5.797227	1.498807
1.106128	3.301674	1.407394	1.631137	1.83963	3.364529
1.921455	5.773031	2.472927	1.049505	2.856874	1.431364
0.3525388	0.3956461	0.3677052	0.1131931	0.2365411	0.08397481
0.0289149	0.06501695	0.09940108	0.008455064	0.0961175	0.2049042

TCGA-DU-8168-01	TCGA-HT-7475-01	TCGA-14-1825-01	TCGA-FG-6691-01	TCGA-TM-A84Q-01	TCGA-12-3653-01
0.5107706	0.07059339	3.033899	1.522885	1.479046	2.649745
0.03815736	0.1703815	1.228719	0.03839184	0.4107823	0.5112381
105.1331	47.45567	261.8295	310.2333	473.6889	974.4585
0.3582337	1.214312	1.687564	11.85538	2.964666	3.244266
1.182471	1.30723	3.243142	10.44836	4.595488	4.290279
2.222409	2.43374	4.462724	11.93349	5.244475	7.955132
1.697694	2.664172	11.06173	41.25492	16.50428	19.67326
0.9956891	0.9065054	3.628326	6.633679	2.682747	5.701046
0.8987648	0.3333644	2.536532	0.3438027	1.298328	0.882072
0.3274918	3.23801	9.975647	20.68829	20.15979	13.31703
1.323608	0.711213	7.010608	3.941643	2.49471	7.408026
6.388977	7.29212	41.75606	60.54858	41.36877	42.82164
1.741485	0.4711579	2.831757	3.88364	2.434729	10.29054
0.7975176	0.4853005	3.356268	3.499494	2.5371	3.672804
0.4118185	0.6937191	2.313998	4.798405	2.906993	6.369277
0.587005	0.2178588	0.6827144	0.1127532	0.2457541	0.6092875
0.02551092	0.2034146	0.1165623	0.06737768	0.3426609	0.1993829

TCGA-41-5651-01	TCGA-HT-7691-01	TCGA-E1-A7YI-01	TCGA-RY-A83Z-01	TCGA-DB-5281-01	TCGA-VM-A8CH-01
1.857505	0.9129306	1.472116	0.6657998	1.311832	2.001138
2.87634	0.720528	0.2641027	0.1694402	0.1632526	0.03015044
185.5912	337.7439	106.2702	240.1375	118.8939	390.5458
1.40707	4.394435	1.086714	1.929676	1.800966	2.626553
2.391961	7.108319	2.493185	3.977437	2.478056	3.635001
3.065836	4.729108	2.303522	2.280074	3.608653	3.615626
7.273117	14.72603	5.599988	11.06663	6.756508	15.32382
2.064912	3.122687	2.920685	2.697716	1.665347	4.176036
1.56638	5.926038	1.318444	0.2418552	3.908939	1.604119
3.893588	24.05475	7.580869	7.926694	4.676135	7.841004
6.707358	4.78072	4.359588	1.074245	4.020863	2.193442
23.03431	58.10432	18.39207	28.88961	25.08578	28.90694
6.018374	5.538235	1.642169	0.6767726	1.955504	2.435975
5.050039	3.384246	2.309382	1.583571	1.157095	2.130614
1.64417	11.93098	4.202106	1.747952	1.0189	3.464645
1.030051	0.3859596	0.143196	0.4387705	0.5172331	1.042911
0.06690492	0.111342	0.07130774	0.03837	0.1458588	0.07643138

TCGA-TM-A84F-01	TCGA-VV-A86M-01	TCGA-QH-A65X-01	TCGA-WY-A859-01	TCGA-DU-7304-01	TCGA-DB-A64X-01
1.538931	3.253703	0.847679	0.09517666	4.47366	0.3583245
0.1135048	0.1421941	0.04652542	0.03943482	0.1874388	0.02992785
837.4159	255.0004	287.5287	60.43498	331.0997	154.0261
5.531422	3.996794	0.3318293	1.821782	6.340146	1.200667
10.35357	4.974391	2.778912	1.669985	4.107895	1.603629
7.727751	4.738225	1.128886	1.999831	5.170944	1.476769
29.25158	16.16357	3.140989	3.81078	14.66115	3.443689
4.052534	3.63035	1.116538	1.179607	3.174759	1.245666
0.6087808	0.7294964	3.752481	0.4003543	0.8495126	1.198148
13.16487	9.058699	0.2846947	1.603392	7.129648	1.498354
2.682398	2.659739	2.900344	0.6922997	3.443051	1.835272
65.79916	35.23107	8.983991	7.308926	24.99763	13.01401
7.237968	0.8016021	1.654552	0.3517618	3.631237	1.171123
3.448896	2.520133	0.8608159	0.9265052	1.715657	0.602538
4.790861	3.292143	1.16785	0.8992939	3.667933	0.9073282
1.394021	1.101524	1.599203	0.1474027	0.6647428	0.7031624
0.1328006	0.0144667	0.01814491	0.03355545	0.4965359	0.02917966

TCGA-FG-8181-01	TCGA-DH-A669-01	TCGA-QH-A6CU-01	TCGA-28-5207-01	TCGA-S9-A6WQ-01	TCGA-DU-7290-01
0.1068204	3.1457	0.6300833	3.715888	1.824849	6.955038
0.1748015	0.04896987	0.06128551	0.1684534	0.2591969	4.882261
105.682	322.9172	219.6015	596.769	426.7358	889.3175
1.295119	0.9954015	2.002081	4.030744	6.266787	10.32552
1.829275	2.465958	5.415738	5.30475	6.3604	13.05447
2.782062	3.852505	2.60944	8.93121	4.359953	16.97729
3.221333	8.649706	11.52114	22.33261	25.17097	35.95115
0.5770923	2.437623	2.723198	3.766268	3.122509	6.884981
4.079555	0.7841949	0.0968502	0.942823	0.3312708	1.884582
3.246944	3.007422	3.520013	6.632762	8.190296	36.11292
2.168917	3.39655	1.307209	5.597144	1.536717	4.658155
7.064771	24.9668	39.01139	52.88182	40.12821	92.69405
5.042521	8.542164	3.454119	2.496537	2.099532	13.01198
0.9327039	1.370063	2.244056	1.69996	2.100182	5.9729
2.60329	1.834235	2.427392	4.726064	3.422084	24.4953
0.1116034	0.4346558	0.1399921	0.6081079	0.2412664	1.070973
0.08002867	0.08785195	0.005975337	0.07390893	0.0546863	0.380528

TCGA-DU-5870-01	TCGA-DU-6397-02	TCGA-HW-8321-01	TCGA-06-5859-01	TCGA-TQ-A7RI-01	TCGA-06-0221-02
2.290692	1.657524	1.953512	5.096542	1.664522	2.048154
0.1188297	0.1006035	0.2101779	0.8564042	0.05678696	0.114496
131.2592	118.446	137.7456	929.8448	56.8549	209.4682
2.448915	0.4920177	2.173427	2.30323	2.092958	1.35218
3.847963	1.843255	8.581265	6.032521	1.985764	2.275909
3.896051	3.890391	4.674223	9.875777	2.727207	2.553487
11.42806	3.206915	7.38832	22.28372	9.648387	6.858018
1.438048	1.001569	1.103998	6.832601	2.35869	2.112022
0.4812068	1.966113	1.2893	1.414704	0.05982739	1.244644
4.461956	0.7723045	6.703959	11.13536	3.120087	2.483999
1.843679	7.368709	3.689612	2.702988	1.342697	1.700803
21.51173	9.690606	21.86051	75.01223	14.33947	15.02759
1.006341	1.21041	33.79723	2.335817	2.056329	1.750824
1.109822	0.9368725	2.313439	2.747062	1.345463	0.9567921
3.143181	0.8264555	5.694023	18.58243	1.435396	1.412401
0.2399319	1.142457	1.088524	0.9035109	0.09407989	0.8967044
0.04344712	0.03531183	0.1150216	0.335619	0.01022165	0.2070297

TCGA-DH-A66D-01	TCGA-S9-A6WL-01	TCGA-12-5295-01	TCGA-DU-7012-01	TCGA-12-3650-01	TCGA-DB-A4XA-01
4.335452	0.8274405	3.765646	9.914216	2.636887	3.538915
0.260141	0.02863985	1.217338	0.4058811	0.9612662	0.02266083
379.6735	107.2186	1511.12	1401.202	472.3877	338.5238
5.928122	1.18547	4.839081	10.89803	1.60966	1.575814
7.23468	1.249967	5.948285	9.782876	4.551168	3.079661
5.36178	3.354293	7.279193	14.79907	4.639454	3.084915
33.44827	3.994709	24.13187	44.59986	15.77422	9.40648
4.006324	0.9383415	13.23824	7.217579	7.115781	2.738773
0.4376926	3.796801	2.133416	1.736033	1.030346	1.020618
11.28107	2.1986	47.26299	8.272995	13.41957	1.096709
2.579907	3.534392	7.496764	4.209764	6.43763	1.744596
39.77131	10.65335	92.19727	97.77018	58.82005	26.30552
4.426556	2.932833	3.260983	10.11439	2.421473	3.249222
2.397436	1.024568	4.779871	2.609506	5.125092	1.305984
3.440245	0.6792186	31.98963	10.23395	7.197842	2.144144
0.2888793	0.2429918	2.314718	1.107132	0.7037414	0.6877115
0.07268417	0.02233908	0.260206	0.2272934	0.08801854	0.006628292

TCGA-DB-A75M-01	TCGA-S9-A6U0-01	TCGA-76-4932-01	TCGA-S9-A7R3-01	TCGA-27-1837-01	TCGA-RY-A83X-01
1.036706	2.685038	0.5388651	2.092392	0.6450941	2.099283
0.01680599	0.2726426	1.07602	0.02499677	0.4354032	0.01489462
175.6449	769.1695	491.434	222.2268	736.3305	19.96231
4.70359	5.161833	3.479851	4.515637	4.154738	1.471118
3.769104	5.83304	4.594789	2.976937	6.020716	1.715273
2.029089	6.788017	6.822633	2.890292	7.470209	1.748799
13.44922	24.29268	16.78012	9.405777	25.59052	4.581913
2.286386	6.745901	4.075673	3.315293	6.527484	1.844137
0.2080432	3.112745	1.023617	0.6794461	1.105621	0.1961512
3.632048	3.454285	8.599745	3.145378	8.160537	1.876696
1.40667	17.61155	9.984132	2.279329	10.8179	1.097765
21.75425	34.90636	50.84861	26.58129	62.91892	11.4878
2.120167	2.794784	7.0217	1.640976	6.403105	0.5350036
1.506423	18.04326	7.293649	1.304066	5.797421	0.7365423
2.163364	1.583499	5.827718	3.622924	2.440235	1.207202
0.3290509	1.154788	0.3022386	0.1664032	0.1530114	0.1458137
0.009831506	0.03906022	0.09561598	0.07019092	0.03918629	0.01307003

TCGA-S9-A6TW-01	TCGA-P5-A736-01	TCGA-TQ-A7RG-01	TCGA-19-2629-01	TCGA-DU-5872-01	TCGA-HT-7473-01
0.8740455	4.167354	2.038419	3.885067	3.24717	9.214449
0.0582067	0.1625963	0.01547747	0.6871282	0.2448172	0.3738278
140.8786	384.1181	104.1013	1232.386	337.6163	565.2168
0.5645485	7.235769	2.195945	5.092643	3.723679	7.963321
1.663928	8.09247	2.951706	8.385402	3.150541	7.047775
2.087141	4.826641	3.329334	9.042143	3.910586	8.259484
3.504708	35.89177	9.985725	26.67704	10.92107	35.06311
1.186164	6.15704	3.107867	8.360638	2.297527	8.782335
2.967097	0.6700928	0.3206875	1.151291	1.840807	1.157167
0.9937998	6.687957	4.769864	25.60766	5.602356	20.05366
3.965077	2.11477	1.240941	4.49575	2.352923	3.338023
10.77718	50.03781	19.34531	85.15312	25.79805	72.37597
2.196651	6.904504	1.578927	1.871211	4.352264	8.067148
0.7218731	2.660579	1.07511	3.504144	1.105595	2.782131
1.018075	6.790873	2.096412	5.386066	4.496593	7.999718
0.8854211	0.7958837	0.3030391	0.3403908	1.234456	0.6640203
0.03143162	0.03916659	0.07243454	0.3099528	0.2628934	0.2254528

TCGA-S9-A7J1-01	TCGA-RY-A840-01	TCGA-12-3652-01	TCGA-TM-A84B-01	TCGA-S9-A7R8-01	TCGA-12-0616-01	TCGA-S9-A7IX-01
1.790588	0.2474399	0.3133124	0.8595378	3.259575	2.440637	0.3542838
0.009283983	0.02330057	0.7857779	0.07090916	0.2710665	0.5870103	0.198502
100.3749	130.3493	198.1831	82.54433	270.512	1041.051	658.082
2.174465	0.7478309	0.630275	0.6553969	4.722788	1.468058	7.286465
2.21051	0.8417426	1.430453	1.631291	4.329268	3.775254	5.744004
2.826646	1.904834	2.355947	4.301325	3.737576	4.876959	7.571978
7.438498	2.323959	4.43671	6.283962	17.01559	13.26982	21.33659
1.590069	0.7352307	1.659743	1.216456	4.971884	5.42854	3.355486
0.2640885	0.2749387	0.8219779	1.515459	0.4911971	1.413576	1.553537
2.22333	0.6066088	2.678495	0.2479438	10.0607	18.51124	9.003455
1.148372	0.8972566	9.383203	3.291185	2.050474	6.102158	7.061861
19.42812	7.994115	20.87167	11.236	26.00086	67.64761	44.85682
1.361007	0.4735849	4.673985	2.078623	1.146216	3.223634	2.910814
0.7975519	0.4552804	7.402462	1.282172	2.275144	3.51852	2.123893
0.9192983	0.4580255	3.270085	1.089743	7.046249	9.483513	4.31072
0.236307	0.9671663	1.156606	0.4561744	0.2547511	0.626907	0.1943273
0.02172452	0.02726166	0.1238854	0.003950653	0.05708661	0.2854242	0.1078291

TCGA-DB-A64P-01	TCGA-HT-7611-01	TCGA-TM-A84I-01	TCGA-TQ-A8XE-02	TCGA-HT-A617-01	TCGA-28-2499-01
1.948414	3.572779	1.116173	5.053334	3.370744	3.969528
0.0949286	0.07412499	0.4090275	0.1577347	0.1835878	1.571478
102.6566	135.8713	303.2366	405.2026	860.6281	550.528
1.405918	5.283321	2.628835	4.067212	3.545206	2.788767
2.070571	5.043435	3.121961	4.299964	4.657025	4.086696
2.850693	4.572647	2.673009	4.769347	10.2832	10.29341
6.9788	14.51496	12.06294	15.70923	19.97869	15.10051
2.505698	3.894468	2.481048	5.68822	4.182089	3.777869
1.557317	0.2200823	0.5443292	0.337108	0.9932241	0.9026102
3.123171	8.529924	6.071602	12.79326	4.628834	6.824983
4.214909	1.432205	1.90643	3.37046	4.720052	6.217267
14.51424	46.51108	18.98677	34.82977	54.88039	49.35635
1.731944	5.887837	2.861695	0.8054393	2.637156	7.867872
1.268331	2.090169	1.504361	2.73719	2.01067	3.100379
2.612717	3.420318	2.096937	3.074189	3.173563	8.637916
0.5841449	0.151729	0.5985303	0.4411924	0.7189071	1.473555
0.03702216	0.06701573	0.04533747	0.01581854	0.02902673	0.09968472

TCGA-DU-A5TT-01	TCGA-19-2624-01	TCGA-KT-A7W1-01	TCGA-S9-A6U1-01	TCGA-DU-8167-01	TCGA-FG-8186-01
0.8134189	0.811083	0.3867502	3.374792	2.838521	2.943488
0.6657932	0.6647845	0.2626578	0.03866581	0.3241017	0.2964018
255.8015	144.0903	252.1213	63.5343	243.758	277.9501
2.408901	0.806459	1.235826	3.883801	4.929922	2.52054
2.873126	2.538732	2.24082	4.281052	9.761971	5.934456
3.601208	1.663431	3.555976	4.026364	6.544785	5.947109
9.484712	3.929685	5.29342	16.32444	17.71306	14.60366
2.629033	1.644066	1.3961	3.697178	3.717348	3.751091
2.242701	1.02868	0.8815534	0.5793567	0.6506092	1.076936
11.54194	1.837488	2.504782	4.488245	13.41961	8.675408
3.348472	6.577907	5.62531	1.347557	2.217041	2.462008
28.80022	20.03899	16.47941	31.94887	61.10847	29.19757
1.69828	2.334469	6.223903	1.676151	4.251408	5.064703
1.796121	4.979052	1.655953	1.678909	2.533614	2.318953
5.627608	2.535553	4.287934	2.590269	8.827846	3.088263
0.08867902	0.1382957	0.1763203	0.1177637	0.3858878	0.5133743
0.1271801	0.01458371	0.05268165	0.08545144	0.06149173	0.2134093

TCGA-06-5416-01	TCGA-02-2485-01	TCGA-S9-A6TS-01	TCGA-DU-A7TJ-01	TCGA-CS-4942-01	TCGA-P5-A5F6-01
1.889111	2.786194	3.385101	1.699763	7.072634	1.214684
1.107022	0.6077673	0.03997606	0.2340275	0.08093495	0.3122561
165.3637	428.0839	217.2807	624.9936	678.4637	118.6183
0.565833	1.097415	3.930553	4.672442	6.615018	1.275508
1.774083	3.380299	4.717869	4.035508	7.831087	16.53049
2.996677	7.869677	3.637401	7.615416	12.1793	14.11462
4.547875	8.309865	14.23819	15.74981	34.98049	3.901575
1.521598	4.176597	2.791896	2.999327	6.38311	1.623804
2.255348	1.667898	0.4492418	0.9784031	0.2743415	1.561134
4.110893	6.355566	6.352664	8.214098	17.57064	1.373833
6.194949	5.754634	1.987053	5.465047	1.889846	2.890122
15.14425	38.60608	35.54477	32.37893	67.523	12.36518
1.284555	5.175666	1.924924	7.884306	2.688241	2.745559
8.446753	1.887087	2.520331	1.828894	2.333694	1.661262
2.054145	5.212811	2.280572	1.858229	4.465219	3.348056
1.30623	0.6909505	0.4087466	0.09455153	0.1377962	0.9893207
0.06004311	0.0516112	0.05196887	0.03042358	0.230559	0.06642539

TCGA-TM-A84J-01	TCGA-VW-A8FI-01	TCGA-DB-5274-01	TCGA-DU-A6S7-01	TCGA-28-2514-01	TCGA-TQ-A7RJ-01
1.00955	0.5911176	2.1812	0.4012101	1.512387	2.116821
0.2595586	0.3785119	0.1001214	0.03605725	0.8092991	0.01132704
59.75972	434.0538	182.443	53.25212	424.6971	215.8208
0.9355936	2.051421	1.036702	5.658846	2.522396	5.383871
1.447512	6.859597	2.154581	6.255827	3.053139	5.491816
1.816701	4.683107	2.095299	5.296649	5.320754	3.995719
4.229505	15.73284	5.716727	22.77823	12.40534	18.36008
1.181711	3.79288	2.715773	6.091871	2.631472	2.800076
2.677802	0.3251573	1.518941	0.5033383	2.529149	0.7279438
3.015807	3.819235	5.614147	8.349169	14.24959	4.845507
8.757784	1.606955	1.558776	2.291833	14.17533	2.126976
15.26264	71.52108	50.15417	32.10084	34.95452	33.14764
1.950725	0.516966	0.321619	2.442884	11.03082	1.706823
5.409146	3.0828	0.9588847	2.650698	7.802914	2.676606
1.048892	3.442624	2.269205	3.484429	4.855554	2.713999
0.2684828	0.2736379	1.695672	0.2647419	0.6053364	0.1404584
0.06875855	0.05450572	0.07614232	0.06328048	0.2021429	0.03092283

TCGA-06-0747-01	TCGA-27-2521-01	TCGA-14-1829-01	TCGA-HT-A5RA-01	TCGA-12-0821-01	TCGA-DB-A75O-01	TCGA-S9-A7J0-01
1.09615	1.466341	3.204631	0.5429076	1.081556	0.8844371	2.076971
0.5210428	2.739727	3.910257	0.3654692	0.8158874	0.03670683	0.07410291
290.1049	213.9325	603.1665	292.3504	203.3288	373.8248	118.478
1.984235	1.230514	1.241406	2.523729	1.344818	6.936391	2.497545
2.865598	2.575564	3.670067	3.547587	2.623725	6.816057	3.016764
2.579941	2.244593	8.832969	14.05334	4.837369	3.589513	2.009127
10.89625	7.849289	11.4502	14.25271	8.948643	19.18375	10.1546
2.87739	2.231128	6.362483	4.046083	2.565343	5.105523	2.438291
1.047976	1.495775	2.858509	2.117702	2.373815	0.176787	0.3615895
8.688243	5.949912	26.12157	5.481785	7.615431	6.528378	1.972159
3.555054	5.29455	3.572548	17.79427	15.27436	3.313556	1.916331
22.54744	28.15069	101.6207	31.62311	45.81975	28.46988	15.97302
3.445568	3.322157	2.610054	8.309062	5.058884	3.83447	1.152615
2.71485	2.4035	1.245442	6.643004	5.953821	3.396881	1.27074
19.82254	2.827989	7.324933	1.183406	3.200943	2.753913	1.498999
0.1939165	1.074619	1.049078	0.4450471	0.9287539	0.02053419	1.153075
0.09572547	0.1432914	0.9136664	0.2763749	0.1498482	0.03681171	0.03194226

TCGA-DH-A669-02	TCGA-27-1834-01	TCGA-HT-7478-01	TCGA-28-5204-01	TCGA-DU-A76O-01	TCGA-DB-A64V-01
1.496291	1.98698	16.02408	3.385091	2.500184	5.063824
0.04540873	0.1611246	1.348399	0.4805729	0.09633219	0.05575782
201.3159	986.6007	868.0087	480.0525	528.3252	602.1095
1.228373	4.971739	8.367788	2.318186	8.977375	2.746592
3.104219	6.612021	11.38928	4.248505	6.870482	5.471235
2.533061	14.72675	13.87842	11.31018	5.342608	4.578046
8.222172	28.1285	35.336	11.86045	22.3474	18.07838
1.969192	6.797001	7.484912	4.202393	5.139676	5.395425
0.9567992	0.8311962	1.137906	1.793157	0.6862651	0.4473517
3.432853	7.399191	52.61228	13.94319	7.050653	3.421432
4.786588	4.402816	3.409495	14.94392	2.547468	1.911381
21.26837	74.47639	74.93509	50.26555	33.12445	56.70362
2.418724	8.495434	7.97455	4.040622	1.666459	2.730493
2.563444	2.441299	2.976626	3.315071	2.075231	1.490342
1.172385	5.216295	11.64906	2.768521	2.82631	3.30324
0.3734114	0.8267538	0.6118072	0.49987	0.1886124	0.9573399
0	0.1300109	0.6994014	0.1757095	0.1127087	0.04014563

TCGA-CS-4941-01	TCGA-QH-A6X8-01	TCGA-S9-A6WP-01	TCGA-DH-A7UV-01	TCGA-S9-A7R4-01	TCGA-DU-7013-01
6.765363	0.8232476	5.832396	1.353285	2.624011	4.774148
0.170303	0.06946968	0.03810892	0.01960527	0.1877632	0.1850315
747.5407	56.05987	311.1077	79.06563	374.2737	359.3561
5.593298	2.23275	1.788062	4.03906	5.20215	3.35454
8.987387	3.959028	3.899553	5.296614	5.542256	3.460135
12.54562	2.067914	4.09433	3.597935	4.68391	4.275612
27.33789	11.96961	12.71099	13.91253	10.75355	17.06722
4.606863	2.530626	3.864707	3.996274	2.384347	4.606382
0.4747186	0.2410937	1.067972	0.1982876	3.981685	0.8108303
11.92499	4.194103	1.984261	5.169487	8.364003	5.773032
3.778481	1.21406	3.620838	1.568163	4.489069	4.525822
57.48548	23.9112	34.7745	38.08639	33.40295	37.11418
6.614486	2.56332	2.158989	1.511468	2.991849	2.224012
3.006954	1.860475	1.320522	2.668324	1.957893	1.79648
3.903559	1.726463	1.956649	4.198982	5.169818	2.549118
0.6877255	0.2240285	0.8356866	0.1765751	0.2827912	0.5250437
0.1037784	0.004781149	0.03566994	0.0137629	0.08449343	0.04078738

TCGA-FG-6688-01	TCGA-DU-8161-01	TCGA-HT-7858-01	TCGA-QH-A6CV-01	TCGA-TM-A7C3-01	TCGA-HT-8114-01
2.14409	2.256268	6.982698	1.902404	1.01857	3.457073
0.4354996	0.1530874	0.5039731	0.3505529	0.2219372	0.6151643
803.207	782.4517	464.8993	802.817	277.8916	418.4873
4.236072	6.039307	3.829624	5.059594	1.156028	6.425695
7.125666	7.034211	5.742369	5.968378	1.88285	5.974664
6.726584	10.7439	4.955297	7.011125	2.181074	5.811831
30.75271	34.27368	21.27711	26.74455	3.98669	16.93904
6.918873	5.010296	5.422146	5.161008	1.207555	5.157311
2.107446	1.209629	1.185094	0.9694699	2.199817	1.081774
11.10501	15.19574	5.214548	6.413289	1.103582	10.49135
4.506097	3.589671	1.690767	3.056818	4.161122	4.78462
60.49356	51.77463	51.00288	64.26734	22.10926	40.96504
7.169356	4.399773	3.616935	0.5272092	2.142749	1.800449
2.660749	1.975437	1.701701	2.937657	0.8610287	1.863297
5.863441	2.704235	5.695633	4.152546	1.136184	4.216892
0.1445222	0.5620041	1.66563	0.3260213	0.1684948	1.150723
0.06045325	0.2015013	0.1084953	0.03588785	0.04239454	0.2327791

TCGA-S9-A6TU-01	TCGA-06-0211-01	TCGA-HT-A618-01	TCGA-QH-A6CX-01	TCGA-TM-A84L-01	TCGA-RY-A83Y-01
2.39489	2.655358	2.770697	1.65427	1.545986	2.953646
0.09613151	0.5484879	0.3045575	0.1644549	0.234987	0.06815553
341.9398	476.6935	908.1619	308.4949	197.9619	239.5235
6.170676	2.821106	9.40904	3.744252	6.697543	0.5425222
5.67721	4.065698	9.596795	5.095752	6.529005	3.980863
3.588495	8.876884	10.2421	3.977899	4.129294	2.305663
13.6155	17.17097	22.07897	17.22067	20.74582	6.091612
3.244228	4.723677	6.024136	4.721575	3.404122	2.702617
0.8825697	2.489219	1.415875	0.1834518	0.5846829	0.6402581
7.242232	16.30018	23.42035	5.553593	7.909917	1.450203
2.610161	9.193792	2.839305	3.089585	1.970994	1.664668
28.94407	49.60253	48.8886	31.88634	34.93543	26.37248
3.309934	4.489771	5.172569	2.63856	4.164066	3.655558
1.991596	2.463037	2.943104	2.6165	2.38936	2.170335
5.5334	3.39793	4.489012	2.284769	4.180605	2.099611
0.268885	0.2726067	0.6436302	0.1988777	0.1957829	1.779257
0.04820309	0.1480917	0.3167398	0.01131837	0.05849676	0.01993549

TCGA-HT-8015-01	TCGA-DH-A7UT-01	TCGA-VM-A8C8-01	TCGA-DU-8158-01	TCGA-12-0618-01	TCGA-TM-A84C-01
0.2058155	1.238838	1.502033	2.281963	1.580531	0.2259148
0.06362293	0.09985199	0.07694407	0.4523462	0.8731545	1.012624
347.5914	205.9552	105.1146	828.6075	215.8833	446.9273
5.236905	3.416701	4.153661	6.280675	1.201023	3.429518
3.601567	6.026374	4.042157	6.750191	2.586285	5.10507
3.699246	2.923661	3.127415	9.396476	2.750119	6.545137
11.82066	14.15709	15.8043	27.10572	8.139442	20.6885
2.350345	2.962603	2.021711	5.942056	3.599668	2.757737
0.5936887	0.1636416	0.1331761	0.7538397	1.087159	1.524059
3.754123	6.943255	6.943215	4.588868	6.41815	18.83053
1.962898	2.491134	1.051919	5.261231	5.956879	16.64208
18.81599	23.24216	34.01002	53.87346	27.9107	30.1765
2.988658	1.969881	1.54078	4.819541	4.418181	0.7040787
1.006074	2.835275	1.756312	2.301018	12.32966	1.921192
1.919979	2.042567	2.475163	10.44827	3.819361	2.59201
0.4181984	0.130336	0.3013034	0.8105183	0.9324993	0.1888243
0.01595118	0.006490379	0.03215163	0.141132	0.1625258	0.1088054

TCGA-TQ-A7RK-02	TCGA-WY-A85B-01	TCGA-HT-A615-01	TCGA-FG-7634-01	TCGA-41-2572-01	TCGA-DU-6542-01
3.778084	6.215502	7.766968	5.333508	1.137641	7.270178
0.06026552	0.06152182	0.047097	1.372373	1.118115	0.8308255
132.6453	270.0277	415.5523	277.7143	469.2892	555.4742
4.2566	5.594533	3.948094	3.669189	2.593484	6.800898
6.231495	5.492858	5.254798	3.835852	4.011712	6.005749
4.082518	5.803204	6.029132	8.434757	6.181672	7.727139
22.67066	22.50018	15.8019	20.66169	17.23489	24.0764
3.624574	5.374233	4.44243	3.818302	4.821558	5.009603
0.3401368	0.6481576	0.2332075	5.665678	1.528994	0.7896807
7.53588	12.43896	2.750926	24.66866	15.30471	14.98802
1.165761	1.381647	1.261991	9.197525	11.2761	2.167013
37.23126	53.3421	33.60684	31.80228	52.4931	36.681
4.354527	2.664496	3.454897	7.052725	10.55416	2.336565
2.100706	2.227466	1.382641	3.21164	5.989897	1.403818
3.018472	3.977945	2.521764	4.938274	5.865067	2.993979
0.3539884	0.5374186	0.525614	0.5811624	0.6191676	0.3271091
0.05540123	0.1162762	0.01653105	0.3145983	0.2907099	0.0792445

TCGA-HW-A5KM-01	TCGA-14-1402-02	TCGA-P5-A731-01	TCGA-HT-A4DS-01	TCGA-06-2563-01	TCGA-06-0219-01
12.22502	2.939946	1.198894	1.234095	2.017042	2.484493
0.7436971	2.032149	0.7461266	0.1159973	0.4221499	2.386353
2055.157	265.8517	173.3229	1207.377	375.9912	307.5783
12.28768	1.652933	2.794795	4.387737	2.208223	1.669682
15.77392	2.887615	2.601707	7.825764	3.839513	4.281422
32.14415	8.464233	2.454171	3.638269	10.7671	8.749454
80.88258	9.321943	8.232344	23.37331	10.78988	14.14693
13.00154	4.475619	1.804819	5.205266	3.612539	7.050989
1.336034	2.416781	1.917478	0.3742616	2.080805	1.506351
37.11987	18.23633	4.858339	9.183055	6.952796	22.66814
3.497381	4.864957	4.48711	4.274623	9.524082	4.550437
165.0953	67.01511	20.66529	55.76936	49.71786	122.9663
4.097852	6.101888	6.906946	1.016228	8.118798	2.275881
4.983747	2.69402	2.526059	3.115772	2.290466	3.033522
6.185085	5.651255	2.682568	2.903413	2.705651	4.549414
0.968808	1.257112	0.1942196	0.397452	0.4919899	0.6210059
0.5269287	0.4375978	0.1412896	0.0084823	0.1587585	0.1826013

TCGA-FG-A4MX-01	TCGA-HT-7690-01	TCGA-VM-A8CF-01	TCGA-E1-A7YY-01	TCGA-14-0871-01	TCGA-HT-8105-01
0.7925602	2.615979	5.80319	0.04120915	1.85371	0.8758504
0.01750007	0.5394944	0.195197	0.04523582	1.038617	0.2914869
146.0432	757.2913	930.631	90.38906	96.42724	61.51313
1.564636	3.702735	5.248324	2.751011	0.9297343	1.051692
5.906055	5.560523	9.528307	2.332657	4.282924	2.477705
1.439215	7.374903	4.189745	4.543413	1.931604	3.664564
3.477481	26.05658	31.63271	6.703274	3.912263	4.67879
0.9449426	4.192049	7.520695	2.080006	1.377005	1.459992
0.8020115	2.74015	0.6854934	0.7625247	1.250543	3.580165
2.131966	22.28053	7.902568	3.963322	2.079956	2.703175
3.399454	3.923294	1.672841	1.122094	7.900663	11.78193
29.35223	62.22719	108.9861	10.12309	14.39202	15.65793
1.246958	2.540196	1.528516	1.862618	4.228919	3.146277
1.195955	2.46659	2.588622	1.384243	5.184184	1.088526
1.052549	1.866181	5.366341	1.874646	2.263649	2.522297
0.2055843	0.6194331	0.7502121	0.1476149	1.220128	0.8813546
0	0.2883298	0.02537561	0.03528394	0.1735974	0.2806023

TCGA-DB-A64L-01	TCGA-DU-5852-01	TCGA-28-1747-01	TCGA-HT-A61B-01	TCGA-DU-A7TG-01	TCGA-WY-A858-01
0.5988467	3.74948	3.930884	8.980513	0.1139578	4.384103
0.5061686	0.1910244	0.3902439	0.2472277	0.2582047	0.0985012
97.20888	440.0349	1034.345	835.1771	106.981	811.1509
1.125227	6.631814	5.751746	5.463652	2.419207	8.47638
2.408203	5.881798	10.98223	13.60192	3.090169	8.541287
4.022298	8.090259	13.84133	17.80921	3.893389	6.120595
3.70572	23.71716	36.82517	38.47037	8.726585	24.07641
1.936832	6.538297	10.10788	7.692607	1.264454	4.473819
1.416665	2.01252	0.5242009	1.104369	2.655241	2.179276
4.675824	9.845024	15.89886	45.76406	8.084155	18.96807
1.627067	11.41891	4.264105	2.788268	2.017192	3.742428
11.6866	53.42071	104.8844	101.496	15.50215	67.77567
1.403492	4.156467	2.974396	3.026499	2.655482	1.623622
1.186024	3.31863	4.214024	4.990131	1.358816	3.257805
3.81765	4.428657	3.649511	3.104482	2.602026	5.434706
0.6707083	0.4180154	0.4679948	1.248865	0.1318822	0.8357232
0.4187395	0.03067627	0.09131707	0.4454548	0.2758299	0.1600644

TCGA-08-0386-01	TCGA-S9-A6WG-01	TCGA-DU-5870-02	TCGA-E1-A7YJ-01	TCGA-DH-A66G-01	TCGA-76-4931-01
1.455747	1.666551	1.103058	3.532861	2.766968	1.343441
1.85952	0.9174567	0.01733311	0.5411977	1.875724	1.493874
151.5562	278.3438	117.5721	442.7369	448.9668	200.0463
2.863994	5.014632	0.5861076	3.443617	3.271104	2.802672
2.678466	7.9708	1.647804	7.271597	5.140919	2.80998
3.749839	4.988457	1.140387	5.356156	5.291104	4.920427
12.07278	19.77178	4.349536	22.06923	11.09486	12.08896
3.068819	2.815111	0.9980784	8.827714	1.574567	3.554801
1.893271	43.62829	0.7167497	0.6309926	5.271399	2.178062
7.377263	16.54392	0.3832745	13.27672	23.36295	8.737263
8.820453	43.00162	1.00763	6.202649	4.591695	9.806016
22.20321	34.2176	14.96419	49.57888	24.90905	31.81857
8.419054	30.41897	0.885802	12.23472	9.807268	5.98055
3.954325	4.115636	0.4212639	5.619008	4.322619	5.557971
5.434538	7.352715	1.004366	12.21042	18.60978	3.751931
0.5644688	1.285789	0.8823657	0.6710999	0.6753053	0.4346618
0.2192504	0.3841729	0.03548954	0.1561897	0.5362114	0.2326835

TCGA-FG-A70Z-01	TCGA-TQ-A7RH-01	TCGA-VW-A7QS-01	TCGA-76-4926-01	TCGA-32-2634-01	TCGA-16-0846-01
1.962515	4.114463	0.4438192	1.237782	3.679152	5.039073
0.8981233	0.08511865	1.601809	1.850743	1.008524	0.4139495
623.7204	277.6155	64.20519	344.4596	333.7166	968.5745
3.792366	5.535	0.3619496	3.243779	2.104901	3.025375
5.660416	7.755675	1.272713	3.693314	3.024802	8.287956
6.258806	4.757583	0.7413969	4.715379	7.374133	9.386547
21.87443	17.29619	2.191214	10.53837	14.33432	25.47881
3.776731	3.135887	0.8936932	4.939464	3.06723	6.949124
4.203714	0.6277319	0.9481248	1.467756	3.086773	1.067917
20.42087	5.661722	0.5820636	7.53404	7.623741	10.24716
8.350002	1.528409	11.6684	4.876147	7.488382	3.038161
51.45878	39.60727	10.113	50.4168	38.9883	110.4859
3.835653	3.683555	1.976708	5.254332	17.47072	5.548275
6.867962	1.820009	0.9242491	4.079928	7.850331	2.505579
11.87843	3.603267	1.004346	6.724489	6.47924	6.041334
0.3544741	0.8650292	0.8599373	0.5599444	0.8038999	0.6338426
0.1702884	0.0948465	0.03526563	0.1720824	0.1865571	0.1179756

TCGA-HT-A74K-01	TCGA-76-4925-01	TCGA-DH-5142-01	TCGA-28-5216-01	TCGA-15-0742-01	TCGA-06-2559-01	TCGA-41-2571-01
1.810985	1.760882	3.59013	3.465528	0.8153212	3.72319	2.70094
0.2885472	0.8725567	0.8001393	2.639238	0.8927513	2.775091	2.483385
208.6951	126.456	1639.294	425.1776	325.8718	229.2991	204.5759
1.568448	1.6502	7.771795	2.455451	1.723478	2.797073	3.215107
3.208942	2.510231	8.14725	3.845902	2.937324	3.538867	4.249386
1.42896	3.856733	11.82607	5.56247	3.814897	6.26794	2.661571
9.362139	10.3787	41.74902	18.49397	7.724207	12.85294	17.08056
2.07618	1.889172	4.214927	3.345062	2.814627	5.351613	3.479562
0.9521394	2.228925	1.998332	5.00623	1.375073	3.101038	2.546267
5.151594	7.127905	27.71623	15.45524	9.453263	18.09889	12.08167
5.113675	11.68225	3.172183	12.95228	5.440634	16.52214	5.542356
26.35544	31.0606	78.06452	68.6668	35.94075	45.43387	44.40905
0.8903304	20.54766	4.768125	6.124345	3.939572	6.914567	2.913807
5.198393	7.664258	3.197931	13.78971	5.261353	10.62699	12.04243
2.140396	3.378281	2.12709	10.2434	6.770255	9.424524	8.180548
1.524319	0.6630821	0.5805252	1.332418	0.5622066	1.21216	0.5426689
0.01273963	0.1305249	0.3611593	0.2284776	0.2687651	0.4257131	0.1102556

TCGA-DB-A64S-01	TCGA-32-1982-01	TCGA-19-1390-01	TCGA-DU-8165-01	TCGA-27-1831-01	TCGA-VM-A8CD-01
0.9388856	3.589781	3.339832	7.02815	1.181138	1.670635
0.6374783	1.832914	0.9686617	0.642223	5.698949	0.2272371
459.1585	801.9494	137.3161	844.5474	685.1292	313.0844
4.099098	5.269659	2.973691	7.588545	1.297864	2.615388
8.219264	8.556657	2.257796	5.405404	4.363378	6.075953
6.787065	8.717352	3.108164	8.906221	8.302655	7.702796
24.65432	26.08425	12.57915	24.43197	11.12558	19.67842
4.425134	6.692704	2.250105	4.270026	6.582555	3.300513
6.093628	4.669341	2.023378	2.817518	3.788369	1.300145
35.97219	47.53769	7.159017	8.886132	17.41598	12.64861
2.626481	15.4935	4.822744	5.476478	3.221891	7.21446
49.50378	70.03096	25.1204	60.30589	84.10523	45.57466
3.844291	4.001282	5.192161	6.544519	3.061123	5.904951
3.025572	16.55785	9.668804	2.968561	1.293132	3.489482
12.41552	12.27597	5.173382	9.364911	5.450144	2.179581
0.2739826	1.235465	0.6486136	1.332503	0.5845383	0.1711215
0.3759567	0.4570265	0.1962483	0.1962614	0.3104899	0.1595204

TCGA-DU-7006-01	TCGA-P5-A5EX-01	TCGA-DU-A7T8-01	TCGA-DU-6403-01	TCGA-FG-A710-01	TCGA-S9-A89Z-01
4.490942	7.571802	0.8670388	4.075802	7.455516	4.067981
0.9181243	2.608176	0.2506081	0.3772836	0.2264201	1.463889
941.4704	1115.676	240.6074	465.6978	500.9339	839.5925
9.824896	4.998917	3.488358	4.473221	3.563091	4.754279
8.073697	6.501432	4.604965	7.674298	11.12404	6.916505
9.207724	9.045034	4.034324	6.571754	8.665133	6.588353
26.06115	18.35068	15.48481	19.67912	18.22137	27.29862
6.310363	3.581293	2.036299	7.163669	4.743361	5.081066
0.8221894	3.110553	3.866964	0.2253774	2.102412	1.048968
8.607753	7.918216	13.65352	6.043151	11.36787	13.69704
11.41284	9.369292	2.570649	2.757771	2.924887	4.498563
61.81537	53.28843	23.37362	54.47812	67.55533	54.98334
4.247733	17.18892	5.593523	1.89913	6.737583	4.457256
3.665887	4.128867	2.551355	2.983808	5.53893	2.595334
9.264418	5.334387	3.523612	1.831553	6.080022	5.779212
0.5713896	1.929998	0.6190053	0.2893867	2.254151	1.938902
0.08056541	0.1214005	0.496204	0.0227537	0.08531049	0.151125

TCGA-19-1787-01	TCGA-FG-A6J3-01	TCGA-HT-A74O-01	TCGA-DU-5847-01	TCGA-DU-7007-01	TCGA-DH-A66B-01
8.094373	3.305338	8.740412	4.083828	10.72621	4.633688
0.7654354	1.501188	0.3178413	1.121206	4.397965	0.5997333
847.7018	691.6642	817.5095	659.9507	975.0307	1017.146
4.66933	3.445523	5.808084	7.381957	4.238193	5.020578
6.944104	8.563711	9.932725	9.155242	4.829329	6.15744
8.256564	4.637233	5.22305	15.84341	4.734224	6.558431
34.39277	26.60876	29.96884	49.49678	18.99357	24.34552
6.612932	5.616657	6.377573	4.76439	3.305435	4.124026
1.930794	2.308076	0.3979482	3.201584	2.225076	0.8361239
14.68643	22.47651	10.07817	29.49877	14.19715	18.48215
5.520222	3.412536	2.631792	4.655949	7.500355	2.071347
75.52268	90.47198	55.45857	77.94979	48.29634	61.66012
6.156583	3.606233	3.357593	12.2903	7.69374	7.600725
4.374239	6.188246	3.542619	6.289301	3.261971	2.712707
7.430148	5.890645	4.304464	13.3236	8.66684	4.326161
0.5052675	1.952564	0.9650369	1.278333	1.934737	0.4502726
0.1739944	0.2048087	0.08623174	0.7358942	0.2605377	0.1793789

TCGA-06-2558-01	TCGA-26-5139-01	TCGA-06-0125-01	TCGA-14-2554-01	TCGA-02-0047-01	TCGA-DU-6396-01	TCGA-32-5222-01
4.523137	1.8073	1.892222	0.9003389	4.428474	15.25654	7.972563
2.864774	2.569452	0.982878	5.367412	3.081533	0.8788953	3.141595
502.4663	442.7676	247.1291	700.3439	897.2689	960.4408	368.8436
3.000558	4.075016	1.861518	2.779931	5.972199	7.2406	4.653005
4.411127	5.450247	2.870544	5.874728	6.107073	12.51263	8.231097
8.925499	8.278389	6.867491	9.82199	9.155499	16.3726	10.64999
21.32571	13.46525	10.1643	20.69885	30.42978	52.29733	24.18094
4.164329	5.617444	3.27768	6.470306	4.717398	10.54099	13.79523
5.161325	2.664054	1.861226	3.752595	3.644055	2.553491	2.232296
20.31655	20.77654	7.763036	16.08008	21.89669	37.59946	24.2338
12.63746	11.12954	10.50489	6.41915	10.15521	6.025717	10.03961
66.33011	73.32929	24.82748	100.8884	67.85562	75.93081	105.7507
3.450741	23.96945	6.725325	8.493598	7.179079	3.312897	3.276532
6.172627	7.196287	5.670209	3.555341	10.32901	7.616959	3.937709
6.292198	10.14117	7.780981	10.0176	14.03112	18.67012	14.40186
0.73072	0.5889261	0.3566801	0.7262713	0.9178903	1.058968	1.275835
0.4551048	0.6561279	0.242881	0.4183096	0.7558618	0.961072	0.4291643

TCGA-19-2620-01	TCGA-HW-A5KK-01	TCGA-06-0158-01	TCGA-06-5858-01	TCGA-HT-8110-01	TCGA-DU-A5TU-01
0.9966114	2.226843	4.829619	6.576337	5.551441	3.459404
2.544869	0.7068663	1.6183	2.887573	1.112299	0.7748339
320.7353	594.3235	797.8872	455.3827	1199.674	773.6107
3.586102	7.422587	7.050846	4.934168	9.732425	5.147289
4.889772	11.20695	7.61545	6.315945	11.18211	10.45551
4.553777	8.149146	9.346086	7.909609	13.96519	7.808759
10.32469	31.68233	29.71472	28.70174	43.74442	21.41689
4.12347	6.77268	7.137717	6.515414	12.31905	5.253853
1.197077	0.8559916	3.003739	3.032787	4.707544	1.352185
7.325113	20.02023	24.78707	14.8658	32.72377	17.8493
9.77762	3.251745	8.602078	11.64258	3.893768	3.435594
39.84126	78.70073	67.30759	74.1407	103.5381	60.09108
7.308964	9.789702	13.66789	4.83566	4.728317	5.036287
2.730433	3.935666	7.435631	11.14329	4.911635	3.854849
7.592025	19.07621	10.51512	6.295099	13.86663	4.323456
0.4114151	0.334069	0.4396128	0.2879192	0.4490333	0.5956619
0.2003209	0.1045675	0.1883498	0.318034	0.3756588	0.1334806

TCGA-FG-5964-01	TCGA-06-0174-01	TCGA-14-0736-02	TCGA-HT-8018-01	TCGA-06-0157-01	TCGA-06-0686-01	TCGA-06-0743-01
2.597966	2.451497	4.712909	2.714831	3.183348	4.734913	3.351028
0.1332569	1.398614	4.11287	0.8052606	0.9104861	1.316491	1.668934
653.9282	203.6143	1538.336	282.0037	948.0824	523.8215	694.8648
1.55045	2.705566	2.694869	3.657783	3.392275	2.690336	3.400914
8.890116	3.127521	6.289348	5.985698	4.768273	5.283736	4.249832
5.544998	4.963041	13.28899	9.402492	8.880517	4.495498	5.892744
17.55738	14.7414	22.92184	14.61788	25.35925	16.23586	15.16274
4.780014	3.18106	5.812447	4.397111	4.750441	4.627989	3.696189
1.290698	3.915588	3.215743	9.222893	3.03519	2.637359	1.920514
5.200963	13.68619	44.85192	11.56412	14.76377	13.69814	10.65174
2.863631	12.04202	3.78829	11.1464	8.405888	9.823622	8.786
56.28935	38.56266	142.6838	32.40571	66.02925	72.33145	59.37704
3.836222	2.210676	13.79842	5.777	4.10073	7.444356	4.96076
2.252903	11.98884	5.20368	3.165233	3.568719	10.54534	3.58095
2.560192	5.717532	6.615257	9.485772	4.565122	6.773973	8.321171
1.935775	0.9191831	1.418093	1.563117	0.7627366	2.850991	1.410155
0.03520563	0.2574722	0.5548495	0.3194431	0.3391901	0.1867024	0.2498931

TCGA-32-2616-01	TCGA-28-5208-01	TCGA-HT-7693-01	TCGA-P5-A72U-01	TCGA-02-2483-01	TCGA-06-5408-01	TCGA-28-2510-01
9.296614	6.769522	5.514305	2.242559	9.85827	0.960736	1.533645
1.127225	0.8404565	4.537365	0.08891539	2.548393	2.19649	1.18152
384.4288	543.2141	289.5325	602.1606	488.934	555.7478	218.4575
4.549023	4.125747	5.207579	4.474251	2.278022	3.443042	3.322277
4.85507	4.875295	5.41347	6.816118	5.003152	3.624376	3.682229
6.396814	11.0489	5.206957	6.988832	6.925723	6.258625	4.739855
27.79742	22.69436	14.47131	17.61762	15.66853	14.65164	11.13452
6.100914	6.663125	4.025782	2.280532	4.825786	2.807166	2.61941
1.851224	2.444627	1.599527	2.112394	4.175399	2.496664	4.084603
4.457021	17.90598	12.23422	3.056763	20.42686	7.721652	11.26732
9.205728	11.15639	11.9952	11.40977	8.997933	10.00231	13.06544
52.38991	67.99727	53.31597	32.8615	75.32817	51.45855	36.84208
13.83844	4.583242	3.080759	9.789048	3.411434	10.59187	6.858881
9.594741	4.444931	3.801617	2.813579	5.603394	7.929286	3.524996
8.835379	6.533117	8.374257	2.282619	10.96764	8.17934	5.567388
2.697488	0.9479864	0.7389075	1.375317	3.59183	1.509872	1.001447
0.1379193	0.3313408	0.1725587	0.0312093	0.4150981	0.4561942	0.4607929

TCGA-HT-A61C-01	TCGA-06-0745-01	TCGA-06-0187-01	TCGA-06-0125-02	TCGA-FG-6692-01	TCGA-DU-A5TP-01
2.461028	4.729508	1.99128	4.280923	2.422025	2.44511
0.4392529	2.32579	8.089683	2.708357	0.3363828	2.965532
412.6803	711.5495	488.7829	423.9125	279.0939	841.6667
2.806412	3.369314	5.312142	3.120593	2.091594	8.40864
4.633735	5.550724	5.052791	3.521166	3.522265	18.67408
5.822372	5.297752	8.765881	9.01164	3.957883	22.68516
19.16209	25.29975	24.20051	22.41537	6.819652	48.97374
4.210271	6.836701	4.279214	4.209679	2.861944	7.304372
1.874938	7.06522	4.263894	5.830361	0.6043334	2.595027
17.60826	31.3392	20.61719	20.00639	5.164629	73.19356
10.50928	14.02991	19.80704	11.71931	7.96868	7.877984
56.71553	108.5953	69.34776	42.89485	34.61618	99.83524
4.961671	4.084753	24.33287	19.61791	7.126521	11.13853
6.86499	11.71931	8.261616	16.35066	3.1543	8.847316
4.390993	11.3929	8.770538	12.21524	2.991927	16.47826
0.7625006	2.075739	0.7027269	0.5238463	1.580679	0.5634428
0.1112623	0.4903884	0.9724621	0.6974607	0.07042793	1.420968

TCGA-E1-A7YE-01	TCGA-28-5215-01	TCGA-26-5134-01	TCGA-DB-A75K-01	TCGA-06-0238-01	TCGA-S9-A7R7-01	TCGA-HT-7471-01
7.700238	11.33022	4.115251	2.64878	9.926601	2.812959	4.324816
2.113163	2.900472	1.30719	0.2603317	2.374724	5.897019	0.06958092
695.7997	810.3917	606.7261	138.4319	834.6361	817.2075	222.7521
5.446962	4.826317	2.679617	2.637417	3.590508	8.017534	3.020134
7.656483	5.550101	4.14235	6.006557	6.381156	6.990289	3.960887
7.14503	9.647081	5.40243	4.229507	8.531239	8.993637	7.373335
25.17872	37.28906	19.25432	16.48503	24.6236	36.88214	13.735
4.686305	5.646931	2.660893	3.364495	8.966606	6.417539	5.768754
11.0503	5.378645	3.366437	2.530765	1.837561	0.9669652	1.143579
66.53667	24.11701	5.055554	15.73912	29.54899	33.66894	8.987704
3.481814	10.75569	6.579329	12.55921	3.216134	5.178295	2.750975
119.0516	63.78389	64.85121	40.58063	140.5741	50.82496	44.14082
4.547911	10.3019	3.199296	3.805795	3.284759	13.36794	2.703844
13.2277	6.107546	5.372383	4.163017	4.698766	4.839581	1.919923
49.20812	17.29625	7.396443	6.862561	8.697874	5.797984	1.78967
0.6038057	1.792945	0.9750084	0.532882	2.260065	0.5728072	1.380516
1.627766	0.8460888	0.121382	0.166139	0.3629208	0.3355794	0.331066

TCGA-HT-7601-01	TCGA-DU-6402-01	TCGA-06-5856-01	TCGA-DU-A7TD-01	TCGA-12-5299-01	TCGA-14-0817-01
1.827114	1.585424	4.559575	6.401193	0.7794044	5.370369
0.8906797	1.130255	2.179642	0.8109848	12.03759	2.107825
1141.449	1027.757	477.4776	679.7607	566.1964	1064.686
7.867587	5.505455	4.695234	7.17166	3.181413	3.712993
10.76366	7.291027	4.78688	7.473335	6.233942	10.43401
16.69982	7.264262	10.99292	7.273107	8.076027	14.62493
36.42804	23.98807	21.22021	23.6539	21.707	27.53326
7.059286	3.74013	7.087162	6.611417	6.031734	9.522167
0.9701766	1.757633	2.612917	0.8904349	5.343378	2.908778
43.2571	4.608267	8.846757	6.530625	25.57034	39.88317
2.395598	19.2798	14.34406	8.354138	8.666319	7.892792
90.87552	67.85188	72.98737	59.03762	92.65508	189.044
4.884628	13.81922	3.740498	7.253245	19.39765	11.27624
2.682727	3.885856	6.47741	4.45789	10.39181	6.088946
2.818031	4.715979	8.394949	5.289071	11.25725	7.918959
0.5615934	0.6029344	1.431316	0.8800175	0.5428687	1.850364
0.9317038	0.07522623	0.2203861	0.1257515	1.028101	0.5846988
