# Supplementary material for: HK3 stimulates immune cell infiltration to promote glioma deterioration
Source: Cancer Cell Int. 2023 Oct 1;23:227. doi: 10.1186/s12935-023-03039-w (PMC10543879; doi:10.1186/s12935-023-03039-w)
Supplement: Supplementary file 5 — Supplementary Table S5. Genes positively correlated with HK3 in inflammatory response. [file 12935_2023_3039_MOESM5_ESM.pdf]

Table S5. Genes positively correlated with HK3 in inflammtory_response.

Gene Name	TCGA-E1-5304-01	TCGA-E1-5318-01	TCGA-FG-7638-01	TCGA-DB-A4XH-01	TCGA-E1-A7YM-01	TCGA-TM-A7CF-02	TCGA-S9-A6U2-01	TCGA-HW-7491-01
S100A12	0	0.1636671	0	0.08308159	0.6315145	0	0.07738811	0.7239902
LYZ	0.701459	0.9900562	2.773621	0.5855274	1.026128	0.1001628	1.113529	1.354343
CASP4	0.217489	0.6963702	0.3574258	0.3351087	0.6071219	0.2489161	0.3370052	0.3349959
FPR3	0.5434228	2.770934	0.3782211	0.06986406	0.07867353	0.007082217	0.347074	0.8969299
ADAM8	0.9529226	0.3263481	0.3309289	0.6482449	0.7345474	1.499089	1.08604	0.6276601
RELB	0.4963321	0.9832754	1.545417	0.9075192	0.7976018	1.377791	2.496359	0.7276744
NFAM1	0.4439308	1.133892	0.7430754	0.5585762	0.4573857	0.9044783	1.071607	0.7330574
C3	14.71803	19.56608	19.96824	6.924663	15.26686	23.94112	54.61667	12.40716
TLR2	0.4995608	0.9487238	0.8408452	0.7070243	5.623426	0.8553901	2.414767	1.097962
STAB1	1.043323	5.449658	1.551321	2.221318	0.7971111	2.619936	2.70137	3.172058
CYBA	2.879315	6.934903	3.720339	2.596021	6.91863	5.584888	6.478171	2.544617
TNFRSF1B	2.833273	8.036725	3.990303	2.576919	5.703977	3.096482	5.833165	4.507021
NFKBIZ	0.1792688	0.1637559	0.1527077	0.4265709	0.9163773	0.5388628	1.062965	0.2541694
HAVCR2	2.027121	3.494594	2.31611	1.482058	5.0393	3.364879	5.384984	1.617566
SPP1	76.01996	9.044128	118.2857	80.33374	384.0178	354.5162	126.5393	111.6936
NLRC4	0.4156645	0.542739	0.2791506	0.2196939	0.3731001	0.2491908	0.3816233	0.3142855
LYN	1.101782	2.398775	2.227441	3.284255	2.563401	1.066503	4.235551	1.61244
THEMIS2	1.358263	2.74098	1.492655	1.504081	4.860972	5.331755	2.936478	3.21322
IL10	0.02719861	0.03428901	0.03719613	0.02175746	0.01102543	0.02977539	0.03039968	0.01404437
CCL23	0	0	0	0	0	0.0266015	0	0
HCK	1.826934	2.330616	2.272417	1.190039	2.059516	2.571449	3.67551	1.491411
CCRL2	0.1757558	0.736732	0.4506735	0.2741611	0.4203495	0.160339	0.438718	0.5445235
VNN1	0.04618919	0.09280442	0.03553153	0.04849547	0.1895763	0.05056511	0.2194075	0.08496711
CD14	7.654309	13.55754	9.128476	6.332218	5.561926	2.883371	14.04959	7.59304
IL10RB	13.25418	4.923544	5.14584	2.77326	6.075476	5.646583	3.985077	3.694874
ITGB2	2.757089	3.764378	3.907635	1.766532	5.993513	5.118101	10.82533	3.649748
CCR1	0.8864697	0.9933893	1.31334	0.5515449	1.253718	1.222052	2.88433	0.656095
C5AR2	0.03427351	0.05733402	0.09464453	0.08225101	0.1186279	0.1443098	0.1650158	0.0755551
TLR1	1.372158	0.9347251	0.3041922	0.2657145	0.9088796	0.2207778	0.9900218	0.5053652
S100A8	0.03565199	1.904591	0.07313514	0.8342019	3.706978	0.1170889	0.7172632	4.473482
CCR2	0.004803898	0.004542168	0.02463634	0	0.02921015	0	0.07516992	0.01116251
CLEC7A	0.1540779	0.2711328	0.2633912	0.2978643	2.321361	0.7965212	1.659929	0.1657504
CCL13	0	0	0	0.0252857	0	0	0.04710581	0
FPR1	1.050813	1.627611	1.517427	0.8212394	2.345611	1.021707	2.565323	2.961105
LTBR	0.736736	1.830591	0.8991721	1.278067	2.146104	1.109765	1.509647	0.5883296
XCR1	0.003319073	0	0.01021293	0.003982626	0.004036332	0.01090056	0	0.002570773
CEBPB	1.421067	2.444564	6.131163	6.545648	7.726561	4.506506	8.310478	2.130351
S100A9	0.5594335	5.260154	0.5100447	2.461346	8.693084	0.2381687	2.466361	19.71545
CCL5	0.3202313	0.2242846	1.240831	0.2561682	0.4038574	0.1558088	0.5832771	0.4776945
CCR5	0.05860442	0.1062053	0.2003648	0.03516034	0.2672586	0.1657378	0.447595	0.07565298
IL2RA	0.01129279	0.2295668	0.005791401	0.00677523	0	0.02472532	0.0189328	0.1618154
TLR8	0.1235903	0.1713899	0.0971859	0.01482986	0.2404775	0.04058974	0.2532492	0.2680336
IL15	0.004563092	0.01510069	0.02106125	0.04380281	0.06659023	0.03247007	0.07140186	0.02120593
LY96	0.7681331	3.659349	1.969649	1.347099	1.868256	0.8732478	2.575609	1.647565
C5AR1	0.522614	0.6952442	0.4176089	0.4739676	0.6503323	0.5521681	1.969712	1.962753
SLC11A1	0.4878028	0.2032521	0.5257705	0.3149842	4.615035	0.7806601	1.455442	0.3746235
NAIP	0.05278279	0.05168943	0.01353458	0.02261971	0.08482152	0.1114394	0.1306316	0.04818314
FPR2	0.009693488	0.00458268	0.01491364	0.01163142	0.04715308	0.00530592	0.02166867	0.2552736

TCGA-HT-7855-01	TCGA-CS-4944-01	TCGA-DU-7018-01	TCGA-HT-7608-01	TCGA-P5-A733-01	TCGA-HT-7470-01	TCGA-E1-A7YS-01	TCGA-E1-5307-01
0.09725274	0.06485145	0.04479596	0.227196	0.4866465	0.0275782	0	0.1697682
0.9424254	3.323122	0.5985238	1.834698	0.4695422	0.5425901	0.9704231	1.385901
0.5600521	0.4305614	0.4221291	0.4561689	0.5360087	0.4154137	0.2214747	0.4460018
0.7572288	1.848103	0.2887982	0.3024977	0.1234172	0.03607451	0.252058	0.4568308
0.7377373	0.1159593	0.6140889	0.5324253	0.2825197	0.283917	1.827157	0.2207697
2.495331	1.60494	0.9212913	1.01789	1.198417	1.365007	2.054675	0.7591519
0.8660292	0.8518093	0.5764178	0.6044206	0.5416944	1.083017	0.7627256	1.154996
29.0619	20.26151	4.98887	44.18935	16.15517	59.29823	6.48953	54.14514
1.983091	1.307729	0.4640861	1.7478	0.5144549	1.190459	1.264323	4.744568
10.34532	4.529097	2.255122	1.836783	2.544093	2.498749	1.62006	2.589693
6.877993	9.001176	2.094401	4.820386	3.754586	4.995884	2.27386	6.863348
9.493048	6.841707	3.909488	6.972658	4.202657	5.511641	3.576084	8.275599
0.3129708	0.0711476	0.4108523	0.4586264	0.2806752	0.4623063	0.9115598	0.2354203
3.995015	4.547955	0.8265467	3.116987	1.571495	5.772035	1.597984	6.648665
31.05238	68.14135	8.083017	48.79139	10.2093	134.4906	25.18167	281.2335
0.5606703	0.4912891	0.1600739	0.2110841	0.2832057	0.4099593	0.4241585	0.5872368
2.476085	1.906412	2.783766	1.909925	1.903941	2.926392	0.7314653	4.131825
1.809688	2.060946	0.8159922	2.145677	0.603964	2.479328	1.776624	3.699528
0.1613012	0.3821252	0.04692479	0.1561829	0.06372166	0.1155552	0.02649285	0.1244852
0.04550761	0	0.03144215	0	0	0.03871411	0	0.02383196
3.893947	4.286047	1.080666	2.692882	1.886778	4.458445	0.6383702	4.709848
0.3401251	0.6584721	0.3524993	0.3700563	0.2117652	0.2753495	0.2054342	0.6148037
0.06487691	0.4758835	0.08217881	0.2415512	0.01739142	0.03679461	0.07873362	0.2265033
23.14952	27.85765	7.099091	34.10193	12.66907	10.83153	4.742004	17.40147
5.364301	5.154454	5.333799	3.994208	4.287461	5.006944	7.062545	5.556283
5.232618	6.904442	1.211936	6.806813	2.715326	7.358181	2.08676	11.42947
2.643974	2.091109	0.441825	2.310873	1.925203	3.567453	0.6076249	5.036178
0.15553	0.09630469	0.03581964	0.1340897	0.1297104	0.470443	0.1206966	0.2895993
0.9960596	0.3814867	0.2276939	0.8758443	0.6471809	1.263173	0.3890252	1.807277
1.068298	0.6678539	0.8880376	1.155233	3.633402	1.150225	0.3646322	0.5070095
0.02699005	0.08099048	0.009323992	0.01182235	0.01929378	0.03444135	0.01871697	0
0.5851257	0.3728107	0.1135294	0.881252	0.7090637	0.405722	0.7045411	0.9234741
0.01973244	0.07894959	0.08180132	0	0	0	0.2668379	0
2.757719	2.376378	0.5859222	4.230671	1.096737	3.265731	1.447779	4.732619
1.0669	0.9815804	0.9551454	1.173596	1.390585	1.310263	0.5145715	1.397517
0.00310796	0	0	0.002722736	0	0	0.01616474	0
8.911255	5.504383	2.853086	6.167163	5.771837	5.996115	7.706827	7.229417
2.531942	3.260331	2.312388	3.594875	8.924945	2.698648	1.120107	1.798425
0.1221663	2.910509	0.3146087	0.3697194	0.3810777	0.3212349	0.3812377	0.1163226
0.1783497	0.484867	0.03791553	0.5208122	0.2500822	0.2723272	0.09038255	0.2490673
0.169192	0.04230863	0.07306142	0.04631908	0	0	0.0274994	0
0.2237431	0.2816785	0.01066129	0.1318003	0.07445591	0.2723862	0.03611499	0.2787895
0.03418281	0.06410887	0.01180881	0.05614863	0.03665327	0.02180993	0.02222343	0.01790126
1.853517	7.969356	1.968734	3.126378	2.907052	2.047511	7.424456	1.680565
1.05272	1.644932	0.9121313	2.333013	1.574222	0.9100899	1.675139	3.403165
0.913551	0.7395427	0.08558527	0.7714935	0.2718736	0.4281041	3.100522	1.301462
0.1094421	0.03354714	0.03780792	0.03402089	0.04164094	0.1036161	0.04223223	0.07949998
0.02269231	0.004539601	0.03449289	0.02783151	0.02919879	0.02702663	0.02832586	0.009507018

TCGA-FG-7637-01	TCGA-TQ-A7RP-01	TCGA-DU-7298-01	TCGA-TQ-A7RO-01	TCGA-E1-A7Z6-01	TCGA-DB-A64W-01	TCGA-HT-7603-01	TCGA-IK-7675-01
0.2197982	0.146466	0.05679127	0.04128179	0.1511668	0	0.1163292	0.02979039
0.8943943	1.255888	1.534266	0.8000805	1.220732	0.1677077	0.3757618	2.361952
0.4864278	0.6294565	0.644631	0.5599441	0.8255632	0.1599489	0.1951592	0.3466541
0.2405728	0.2353812	3.470291	0.9179987	0.2330488	0.08209468	0.08151856	0.6513263
0.4831882	0.2666539	0.4461916	1.547877	0.9255646	1.12355	0.3939495	0.5100763
1.2862	3.044774	0.974134	2.473072	1.502877	0.9972434	1.111854	3.111707
0.6598859	0.7669106	1.630955	0.4962757	0.8379662	0.6611437	0.4117717	0.8038028
23.73747	72.99785	56.10697	24.29063	26.43291	5.604452	12.47608	17.63273
1.115319	7.001659	4.318124	2.372602	6.879601	0.3630227	1.076045	1.78931
1.805011	5.258117	3.863388	3.211231	5.57257	1.160615	0.5292824	5.756906
2.776254	10.01195	7.948172	4.204176	7.994887	1.911639	4.079163	4.523241
5.515266	12.60729	9.049551	6.098349	10.28187	1.994077	3.036127	5.765271
0.4905425	1.241779	0.8274089	0.6811566	0.4908951	0.3084601	0.1454903	0.2483879
1.492247	4.67901	11.46477	3.687667	4.317652	1.208423	3.001838	4.778711
77.25382	224.6531	513.0511	86.05182	60.11997	32.12991	140.2873	247.295
0.2019669	0.3517123	0.9700431	0.2124235	0.3997327	0.3202508	0.3034542	0.43007
1.888168	2.288961	4.846696	1.887736	2.307353	1.048609	2.050836	2.083304
1.14746	9.236985	4.462656	2.235113	4.435442	1.196681	1.825621	1.709779
0.01644597	0.09972719	0.2900145	0.06486543	0.07917533	0.01150488	0.01523218	0.1560306
0.04407878	0	0	0	0.02652588	0.03083556	0.02041278	0
2.188314	2.802161	5.090092	1.639069	3.50749	0.9641398	2.14861	2.295748
0.3188187	0.6246025	0.8793648	0.4121706	0.2686038	0.1858596	0.2067019	0.5545431
0.1047332	0.170987	0.3457027	0.08950148	0.1008428	0.007326672	0.08730307	0.1043335
6.57157	17.99786	27.26535	10.79403	21.61052	2.449416	7.31665	16.78147
5.23422	3.881412	7.692572	4.352415	4.830566	5.311374	4.445398	6.43936
2.658598	7.369275	12.78078	4.766325	8.104627	1.215339	3.628189	6.116265
0.7682877	2.316919	8.698255	1.346775	1.763354	0.2166506	0.8715531	2.05677
0.0382595	0.08253945	0.5103366	0.09745708	0.2100934	0.3713595	0.3897934	0.1179703
0.3837624	0.5922328	2.426083	0.6412833	1.096434	0.1605761	0.3521171	0.8506848
1.034756	0.7089186	0.1169697	0.2338207	1.089723	0.271451	1.332756	0.276109
0.02178552	0.1056846	0.02758172	0.09165376	0.01048813	0	0.008071058	0.008267572
0.05046441	2.176491	1.832474	0.2449715	1.588514	0.1031924	0.3775146	0.4750963
0.05733867	0.01783064	0	0	0.02300364	0	0	0
2.489295	4.545143	7.915836	4.410341	3.712969	0.5877776	2.584333	3.396817
1.072251	2.3534	1.44513	1.154326	1.754991	0.9711434	0.9499353	1.147528
0.003010377	0.002808417	0.01633419	0.02374677	0	0.004211853	0	0.002856084
5.563052	21.90748	4.278827	4.296472	9.988215	4.02546	7.986719	5.2227
3.918275	2.761277	0.9686996	1.111818	3.053465	0.7887918	3.655196	1.069769
0.8605856	2.408552	0.5350474	1.088998	0.2201017	0.03010138	0.1992676	0.1735014
0.06201279	0.3925703	0.7811119	0.3552348	0.1599355	0.05577609	0.04512825	0.1302762
0.09218238	0.100331	0.004631278	0.02019896	0	0	0	0.00485876
0.1494608	0.3172117	0.4426535	0.05403714	0.1708918	0.01568342	0.1245869	0.1488904
0.03104021	0.1370668	0.06362645	0.06529456	0.06226495	0.02605723	0.07858141	0.01766957
2.411625	3.09977	3.537899	1.409158	1.967285	1.574595	2.605908	1.906683
0.4795191	3.064598	3.887825	1.811587	2.155958	0.1927876	0.4441284	0.9987806
0.341199	5.862474	1.203705	1.924952	4.244196	0.2360648	0.4115165	0.9480115
0.06326159	0.02552108	0.2473909	0.1146413	0.1049491	0.03588244	0.03167167	0.05353064
0.07912736	0.02870733	0.02782772	0.0231178	0.02116335	0	0.04885826	0.02085327

TCGA-S9-A7QW-01	TCGA-DU-6399-01	TCGA-DU-A76K-01	TCGA-F6-A8O3-01	TCGA-DU-7009-01	TCGA-CS-5390-01	TCGA-HT-7620-01	TCGA-F6-A8O4-01
0.03963283	0.1214112	0.1897824	0.05002892	0.02681982	0.06064698	0.08589453	0.3258963
0.2909553	0.9180518	0.2786488	0.7933157	0.4567884	1.02402	1.828667	3.732288
0.1612733	0.7627354	1.130325	0.2267918	0.2871968	0.3441984	0.4506977	0.2347698
0.1703411	2.558067	0.08106147	0.4487442	0.2355539	0.764979	0.05617849	0.3044991
0.5798167	0.3322171	0.3555046	1.070755	0.6045345	0.3680435	0.9959812	0.9599747
0.8928943	1.859792	2.036018	1.003298	1.49226	1.211061	1.69324	1.451242
0.58586	1.75418	0.4321571	0.320763	0.5970718	1.099017	1.135854	0.6595647
19.58812	100.0513	9.231065	11.31913	12.75997	12.90103	43.20579	26.29845
1.197572	3.459005	0.6353193	0.2498944	0.2447756	0.7816386	0.9852102	0.2850111
2.280228	7.166414	1.155293	1.822223	1.712309	2.386993	2.443605	3.445337
3.858894	11.92437	2.664869	2.597674	2.309237	3.574794	7.799924	4.297274
3.924976	12.81701	3.016865	3.044976	3.22785	5.476378	6.379824	3.190788
0.4243708	0.2370933	0.7257522	0.2261304	0.5461028	0.4098552	0.9272592	0.2288231
2.892738	10.60996	1.299541	1.379543	1.986754	2.374304	6.228369	1.948602
89.94726	74.60347	33.26728	10.87514	92.25665	98.0285	412.4982	28.89501
0.2435932	0.9501331	0.2170139	0.2324053	0.2951808	0.4421005	0.3744613	0.2540853
1.07545	5.88491	1.6008	2.103366	1.810427	1.909797	2.906276	1.821264
2.847783	5.927079	1.370655	0.501924	1.764769	3.429764	3.567365	0.820309
0.03113722	0.09538575	0.0142001	0.006550803	0.02809437	0	0.1687059	0.04655233
0	0	0	0	0	0.02128397	0	0
2.124689	9.137705	0.8130201	1.558922	2.197126	2.697516	4.245001	2.134251
0.2146206	0.7653348	0.2018725	0.3005494	0.3267795	0.2873649	0.3415858	0.1704638
0.04626807	0.2733511	0.05878001	0.03337409	0.05814703	0.0505717	0.1002748	0.02470501
16.33894	45.19249	6.604952	7.962741	8.780519	8.395138	7.97389	9.032288
6.88898	7.444367	4.916745	5.315371	4.372505	4.641293	5.157384	3.744728
3.956752	14.16829	1.799652	2.42107	3.084307	2.756971	8.899327	4.567674
1.526016	9.004168	0.3599681	0.8350487	1.378584	1.351622	3.519065	1.264379
0.1931649	0.1779847	0.1796265	0.1466814	0.1205039	0.08082401	0.2910842	0.1082982
0.3893194	2.253536	0.5233559	0.351438	0.4135634	0.6061355	1.152805	0.4670015
0.5509988	0.7970778	5.486335	0.3606458	0.3176256	0.2966638	0.420166	2.288283
0.005499546	0.01684731	0.00752419	0.01735533	0.007443165	0.06732416	0.02383786	0.01233331
0.6908598	0.664183	0.3519354	0.2628608	0.03315672	0.08997176	2.389262	0.2783659
0	0	0.03300564	0.06090477	0.09795065	0.16612	0.02614181	0.03606757
3.743484	7.043113	1.007003	0.8941367	1.00687	1.955877	3.833565	2.467076
0.6505464	1.714982	1.370846	0.6127985	0.6437526	0.9104387	1.530345	0.7477876
0	0	0.002599278	0.009592811	0.002571288	0.0465151	0.01235241	0.002840413
5.951125	6.342152	8.220995	1.881353	5.07125	2.866872	3.798769	5.35881
1.245309	3.896626	9.443732	1.145289	1.974348	1.06169	1.619343	6.3568
0.2036687	1.237439	1.19819	0.5227558	0.4502253	0.945363	0.3825488	0.8018475
0.1900908	1.520037	0.05736887	0.08116074	0.1248524	0.1240522	0.3695663	0.1546378
0.006464044	0.06930683	0.004421883	0.008159628	0.008748531	0.06429421	0.02101387	0.009664202
0.07545989	0.4081486	0.03548884	0.04167352	0.03510667	0.1479463	0.2402008	0.1269201
0.01828354	0.04200735	0.07504374	0.01648536	0.03358278	0.03796995	0.0594377	0.04295529
1.589622	5.387697	1.388194	1.003298	3.547551	3.622833	2.015762	1.820373
1.954888	3.116845	0.7233727	0.7113227	0.8144464	0.910198	0.9197205	0.6864694
0.9843706	0.453053	0.9501809	0.1583096	0.1585263	0.2878635	0.4871911	0.2847887
0.08416508	0.3999691	0.06643283	0.03813831	0.06863822	0.07760493	0.1496676	0.0564634
0.01664579	0.05949149	0.007591297	0.007004048	0.003754775	0.04245289	0.02405047	0.04562548

TCGA-DU-6410-01	TCGA-HT-A616-01	TCGA-FG-6689-01	TCGA-QH-A6CS-01	TCGA-QH-A65R-01	TCGA-DH-5143-01	TCGA-HT-7606-01	TCGA-CS-5394-01
0.06127139	0.2490569	0.4965117	0.08000381	0.1389415	0.09487116	0.03089978	0.06252801
1.574334	0.3534895	1.976414	3.030621	4.671631	1.838693	0.707752	0.7711788
0.188087	0.2815162	0.8231249	0.4883253	0.4711486	0.5237613	0.4455939	0.3191634
0.05152367	0.1085955	0.1030917	0.433556	1.116444	1.524647	1.414679	0.5783841
0.9528215	0.7467191	0.4244702	0.09103362	0.7076713	0.08224813	0.2879756	0.5929042
0.6326801	2.458373	2.123267	2.82652	1.837827	2.914592	1.070588	1.334
2.670799	0.7244928	2.304043	1.976989	1.407388	1.16867	1.331777	0.2338593
4.897224	26.38683	80.74171	79.95793	6.410301	97.64725	31.17612	6.827544
0.347613	2.98467	3.823876	2.358246	1.19096	1.244183	1.787346	0.4588514
1.362409	1.977981	3.545136	2.57936	3.704624	2.546587	3.049138	3.654068
1.437076	3.599665	9.866175	13.52388	4.443291	10.70602	6.336636	1.707758
1.883583	7.61074	11.13562	6.949179	7.204812	8.490675	5.248942	4.897989
0.3764318	0.9107892	0.355881	0.642484	0.7865423	0.2359187	0.683418	0.3731763
0.7968019	4.889367	10.0891	9.069517	1.40954	10.73986	4.847268	0.7109685
28.83958	63.44036	225.8088	387.0566	45.14122	88.87145	247.7323	3.314308
0.2452208	0.3678583	0.7806632	0.4116752	0.2283869	0.5921419	0.8082542	0.1653438
1.193083	3.462555	5.195609	6.52619	1.486561	4.430971	2.518015	1.047357
0.4876519	4.169165	3.756877	4.502028	1.845627	1.853994	2.105973	0.6728831
0.01604579	0.03261159	0.2383825	0.3352232	0.06367565	0.2567309	0.07282849	0.05731207
0	0	0	0	0.02438065	0.06658978	0	0
0.4465163	2.934608	7.158115	6.708185	1.739936	5.753512	3.395857	0.9427735
0.1503461	0.5338599	0.5741541	0.4129305	1.005158	0.6957012	0.4392392	0.3227311
0.02554619	0.0138454	0.07360465	0.1667819	0.04634364	0.1740425	0.08760574	0.03128414
2.086736	14.75264	25.81931	17.3151	17.03889	39.84964	10.83763	6.740659
6.765493	3.497757	5.08098	5.552736	4.454043	4.24814	5.867849	3.557119
0.7587935	4.542702	13.25003	16.95298	2.853666	16.79561	7.177658	1.181786
0.3195939	1.448682	4.975601	3.702615	0.5007183	7.413776	2.391242	0.4150981
0.02566336	0.1548947	0.2835854	0.237612	0.132262	0.1950703	0.09883204	0.03333234
0.1469703	0.5499938	1.909335	1.04633	0.1547351	1.878316	1.108249	0.4678088
0.3312675	0.8335731	3.437195	0.4737403	3.684435	0.3256677	0.6523354	0.289767
0	0	0.04975905	0.02220304	0.004819962	0.02194091	0.01715091	0.01735307
0.06817357	2.098874	0.6206462	2.344093	0.4122482	0.9461153	0.7640136	0.1391435
0.2797172	0	0.01679025	0	0.08457306	0.03849844	0.03761712	0
0.4098853	8.78644	2.979985	6.278603	1.484381	6.71915	3.733108	0.2622127
0.6165776	1.095639	2.370799	1.946717	1.24919	1.171196	1.022045	0.6085927
0	0.0159185	0.0052891	0	0	0	0	0.002997363
1.046594	9.530755	11.31538	9.54479	9.603582	4.304641	2.577667	1.101177
1.210137	2.459491	7.701429	1.220994	15.06166	1.277554	1.387009	1.68403
0.07346894	0.2559751	1.162358	0.575583	0.4879032	0.6500431	0.3810972	0.3855894
0.05186043	0.1815248	0.2723843	0.3667931	0.07350048	0.8966771	0.2048709	0.02205168
0.01998651	0	0	0.01304848	0.0736486	0	0.005039699	0.1376758
0.04374718	0.06915386	0.3446572	0.1380447	0.04960143	0.2747115	0.2426836	0.02976293
0.006056981	0.04103418	0.05453627	0.07117896	0.03891598	0.02292518	0.02850956	0.02472482
0.8627456	3.542325	4.66083	3.823312	2.519601	3.37337	1.977687	1.28064
0.2312367	5.71247	1.956135	2.373329	5.347269	4.107761	0.5532432	0.4664704
0.1115752	1.261465	1.495385	2.471902	0.7258554	0.4569183	1.079248	0.08213107
0.05504958	0.04294506	0.1111478	0.08277068	0.04161087	0.1325916	0.07907978	0.0459643
0.004288998	0.03486796	0.0270323	0.02240107	0.004862951	0.0221366	0.02162984	0.0218848

TCGA-HT-7875-01	TCGA-S9-A6WM-01	TCGA-HT-A5RB-01	TCGA-P5-A5ET-01	TCGA-DH-5144-01	TCGA-CS-5395-01	TCGA-QH-A870-01	TCGA-S9-A7IZ-01
0.05611364	0.04758609	0.04190409	0	0.209332	0.09887396	0.03921952	0.07337152
0.2966006	2.808712	0.8367515	0.1999023	8.193142	1.529146	0.7485948	0.9372338
0.214316	1.640815	0.2901754	0.2915884	0.4333773	0.6658549	0.2967841	0.874735
0.2883617	0.4268328	0.1722724	0.1205152	2.363821	0.2709879	0.2784982	0.5689999
1.246591	0.1392341	1.444061	1.937472	0.9073975	0.09643314	0.6885241	0.7911384
2.217126	2.745161	1.416096	1.920143	1.393386	2.064419	1.419088	2.335487
0.3447849	0.8432645	0.6866018	0.6093923	0.5512374	1.018406	0.5867354	1.100926
12.9506	46.27719	23.09294	15.77812	12.21687	45.94211	29.21237	72.57415
0.5571149	1.660913	0.8682528	0.3844436	0.398329	1.556825	1.959015	6.338933
2.429559	3.671365	3.743458	2.886971	2.197472	1.417312	4.255785	3.124727
2.437391	11.77042	3.679147	3.8921	3.123955	4.526152	6.420903	10.22336
2.377216	6.074037	4.73781	2.892814	4.047457	4.991113	5.08702	8.926368
0.4654047	0.8394728	0.7171691	0.3836395	0.4776828	0.3557921	0.4165031	0.3928149
1.661946	4.827536	2.607725	2.741943	2.887175	6.076711	4.021486	7.303553
23.72562	159.9409	32.78861	95.72337	87.40393	216.3268	51.09126	69.87226
0.2446301	0.3400882	0.3833346	0.2970315	0.282113	0.3957141	0.280294	0.7813136
2.213499	3.299897	2.726889	1.371321	2.950346	3.450331	2.069345	4.408994
0.8491726	1.573605	1.296461	2.180854	0.9788459	1.311544	1.670628	4.947171
0.02204262	0.1246188	0.08779098	0.1125946	0.1487972	0.146728	0.05135418	0.01921458
0	0.03340054	0	0.02514817	0.04197987	0	0	0.02574963
1.898935	3.188822	2.604477	2.394948	3.015952	3.622053	3.35091	4.591012
0.1804216	0.7489096	0.2694675	0.2182739	0.7894574	0.3569497	0.285389	0.4097396
0.05147062	0.0714251	0.04193107	0.01792596	0.01496192	0.2583371	0.2027647	0.6913601
5.347933	23.66936	12.03861	10.34246	15.82732	11.18838	17.1057	11.82915
3.546272	8.513554	4.784772	5.090147	5.417135	4.870116	3.360674	4.226257
2.473912	8.065426	4.349567	4.002943	4.763174	8.61775	7.278028	14.50195
1.080295	2.825091	1.804224	1.467894	2.393636	2.725557	2.164728	4.237629
0.1645214	0.1014685	0.1595584	0.1882674	0.07287537	0.1581227	0.1941372	0.3268711
0.4550711	0.7555223	0.8136878	0.3273975	0.7719667	1.21595	0.7705187	0.9386378
0.3178285	0.294031	0.2373454	0.1844865	0.4003527	0.984284	0.22214	0.5666965
0.01167969	0.1188569	0.01744414	0.01491509	0.02489778	0.01371999	0.02721097	0.07635906
0.2046474	1.588399	0.07252708	0.141742	0.2772775	0.6559977	1.498223	2.381072
0	0	0	0.0654266	0.01820279	0	0.02387275	0.04466093
1.669608	0.8647314	1.832569	1.209148	1.039075	4.080488	2.404365	2.776493
0.8308124	6.802189	1.043839	0.7299616	0.7621953	1.908393	1.16783	2.024388
0.002689882	0	0.008034914	0	0	0	0.00376008	0.007034323
3.930565	5.532297	4.006125	3.396854	2.407532	2.539888	7.405472	10.24969
1.057893	1.068007	1.54239	0.4824787	1.691345	3.195497	0.9154394	2.140745
0.1057326	1.97262	0.2584086	0.6505591	1.014264	0.7790902	0.3359079	0.5404356
0.09103184	0.7652652	0.1832505	0.1263572	0.3543595	0.4370344	0.08852162	0.6365459
0.004576018	0.01552241	0.01366897	0	0.05365122	0.01075078	0	0.02393353
0.08680662	0.1755427	0.09973039	0.02558146	0.1494611	0.1725657	0.03733648	0.3186849
0.03882978	0.09094634	0.03590104	0.059031	0.1261319	0.04995693	0.1395736	0.1063794
0.7422346	5.197938	1.75224	1.43705	2.577509	2.503178	2.644055	6.4492
0.5614406	1.077532	0.6105132	0.5094229	1.091846	1.209115	1.769278	2.33757
0.2010157	0.6278885	0.3952972	0.2139159	0.273174	0.46439	1.016254	1.931863
0.08555368	0.08809905	0.05476208	0.08193972	0.06513433	0.09152571	0.0726095	0.1078707
0.01963977	0	0.01759972	0.01504812	0.004186641	0.05998354	0.0329444	0.03081604

TCGA-VV-A829-01	TCGA-P5-A730-01	TCGA-QH-A6XA-01	TCGA-14-0790-01	TCGA-DU-A76R-01	TCGA-HT-7480-01	TCGA-R8-A6MK-01	TCGA-S9-A6UB-01
0.1473147	0.08203351	0.1873126	0.1183207	0.07511343	0	0.03104071	0
12.82632	3.697693	0.5610459	3.821946	2.878454	0.3347375	0.3919505	0.9316127
0.7887509	0.6822596	0.5134885	3.860196	0.6327499	0.2615714	0.6005281	0.2235422
1.176844	0.605515	0.1120092	0.9839142	0.9053444	0.2485173	0.800474	0.5697466
0.4709448	0.804529	0.6617387	0.384666	0.9482992	1.570605	0.8275011	1.205533
0.9365769	1.617128	1.617665	2.160805	1.076882	1.394317	2.585369	0.9001078
0.9805912	0.62458	2.02496	0.4952098	0.6989797	0.4882093	0.4781987	0.5576835
30.49199	4.352405	57.16586	22.57166	16.20497	25.0033	25.223	11.81161
2.284728	0.6981051	3.626997	1.707368	2.515173	0.3682113	1.205931	0.53356
4.297247	6.292934	6.302967	2.454383	3.134602	3.080546	1.638585	1.753673
5.427101	2.765802	8.751879	17.46321	4.572387	3.295923	5.287849	3.33773
7.35533	6.662859	9.29572	3.922177	6.457242	3.639804	3.825507	2.312188
0.4977799	0.2231945	0.5786824	0.4880779	1.222903	0.5931354	0.3923057	0.4230872
3.530596	0.8713149	6.861334	2.022058	2.761662	2.819926	4.25225	2.272259
34.45577	121.9785	254.8478	60.26025	41.81727	47.87684	233.4273	121.5533
0.7159234	0.228648	0.4337342	0.3720703	0.3113559	0.3995791	0.4303729	0.3827969
3.482331	1.216059	3.953281	9.820292	4.563994	2.420826	2.006961	2.139416
3.042618	2.993202	4.640375	1.332971	1.852397	1.180455	2.317393	1.70887
0.1639602	0.04296598	0.01962142	0.09295771	0.0885184	0.04975371	0.06503169	0.06473933
0.05169988	0.02878954	0	0	0	0	0.02178738	0
3.566188	1.333071	6.16937	1.724528	1.824634	2.232522	2.990128	1.166515
0.5609124	0.3123491	0.3803774	0.8509735	0.2860003	0.2801691	0.4044723	0.327701
0.3009612	0.0752458	0.09996439	0.06906485	0.1064792	0.01810557	0.2018945	0.1236842
27.80667	11.34433	37.83369	13.25894	14.18333	5.583337	5.908169	4.990455
7.945733	7.14623	6.229613	7.658254	4.823463	4.524284	4.9027	3.983356
4.768612	1.487924	12.79621	4.310236	4.533726	4.19887	7.051899	3.730289
1.117671	1.882716	2.64331	2.390113	1.695402	1.770885	1.089209	1.148785
0.1206001	0.0437303	0.3138216	0.03153711	0.1458649	0.3451704	0.1111022	0.07530397
1.169455	0.3232681	0.7873491	0.5405964	0.8837049	0.6324214	0.3900109	0.2870753
32.18101	4.92095	0.3472173	0.9443308	1.005594	0.06987563	0.5594115	0.04243022
0.3730618	0.1536727	0.01559517	0.09851077	0.04690313	0.003766134	0	0.01143445
1.484292	0.522293	1.171745	0.9727429	1.174692	0.285206	1.013098	0.5195525
0.2017571	0.02496672	0.02280328	0	0.1600243	0	0.3778869	0
2.618158	0.1556233	5.640637	0.8624091	2.894907	1.528385	2.188095	0.6252978
1.403454	1.444639	1.696444	1.054034	1.657339	1.018604	0.8884463	0.6864912
0.003530863	0.007864771	0.003591632	0.01134372	0.01440265	0.005204137	0	0
10.00678	4.898902	10.51632	5.436521	5.430516	2.645043	3.888245	4.015339
71.38265	8.874262	1.042588	3.452216	3.607658	0.4142159	1.254001	0.3328971
0.6056271	0.8993303	0.3850315	2.513219	0.5017994	0.3998254	0.1595147	0.2964222
0.2285945	0.1446533	0.1743966	0.3004418	0.3761603	0.3637262	0.1445016	0.145305
0.1081205	0.1605542	0.03666043	0.05789369	0.02450173	0.00885326	0	0
0.5127587	0.09761855	0.1694032	0.08447983	0.1966443	0.06782415	0.10712	0.0833496
0.04126124	0.06487535	0.03703356	0.1871454	0.07672846	0.03040746	0.09410152	0.05702168
19.98869	5.110404	3.612572	5.149582	4.006256	1.852913	2.119155	1.336098
3.671935	0.3743908	4.32039	1.080002	1.285532	0.5478761	0.7519193	0.4917937
0.6288721	0.08816049	1.664103	0.7700119	1.879065	0.1215333	1.234287	0.4354086
0.124334	0.0491356	0.1591124	0.06442775	0.07771117	0.09901728	0.07944045	0.06730495
0.02578007	0.01148469	0.03146852	0.02484735	0.03680558	0	0.0173828	0

TCGA-HT-7689-01	TCGA-E1-5302-01	TCGA-TM-A7CF-01	TCGA-HT-7692-01	TCGA-E1-5305-01	TCGA-P5-A77X-01	TCGA-QH-A6CZ-01	TCGA-DB-5277-01
0.03060396	0	0	0	0.7976348	0	0	0
0.2785932	5.955602	0.2377869	1.57464	3.94671	0.3922118	0.6403114	5.993604
0.5396436	0.9782228	0.1734245	0.481246	0.5637308	0.3251689	0.2309039	0.4689518
0.3431353	3.663997	0.02389246	0.8016348	0.4620642	0.1023956	0.3191856	2.724366
0.5173734	0.1773575	1.200821	2.281238	0.3025339	1.087413	0.6419457	0.3232447
2.695246	1.626691	1.192938	0.9413596	1.551958	0.6254315	0.7133091	3.403515
1.885882	1.485975	0.8918702	0.4993371	1.072536	0.3934099	0.4854347	0.9520527
68.80229	77.33408	28.794	19.94147	34.80581	12.28894	13.83631	20.97136
2.336408	8.734946	0.5203778	2.743652	3.398855	0.4561725	0.8919538	0.5580252
5.511366	2.791419	3.069211	6.79088	2.829163	2.032364	2.323808	3.013706
9.128258	12.17132	4.570528	6.099302	6.160084	2.383306	2.467402	4.845712
8.086385	13.75385	3.511393	3.498159	6.703394	3.099561	2.879259	7.283601
0.5425747	0.2168696	0.3553508	0.5647573	0.2345198	0.5380325	0.7463718	0.3059169
8.205642	12.95661	2.257574	3.705222	8.189638	1.716177	2.538724	3.386357
183.9846	331.4198	23.39876	70.64948	429.668	40.517	77.33644	104.1198
0.8092653	1.022878	0.4081497	0.4160803	0.5358504	0.4846204	0.2908965	0.4742599
3.811348	5.756877	2.055965	2.281413	4.41566	2.208542	1.756203	3.011823
5.413503	6.003818	1.041486	1.76894	2.771785	0.9904633	1.605088	2.276701
0.2564668	0.1092507	0.1004498	0.1203668	0.1253311	0.1076241	0.03806907	0.09654633
0.02148083	0.02252427	0	0	0	0	0	0.0235241
7.218892	5.220197	3.441442	2.145747	3.494347	1.199117	1.783885	4.3795
0.5230768	1.026374	0.1081834	0.4200137	0.5196743	0.2492067	0.2644501	0.7146223
0.02551972	0.668984	0.03553869	0.06643297	0.1928859	0	0.0424263	0.03912605
29.41939	27.56971	6.063466	16.36891	30.96621	6.584321	12.82626	25.719
6.191644	7.03453	4.476049	7.215847	5.527938	6.949636	3.920367	6.917028
14.21789	20.26934	5.001915	4.791835	11.06642	2.150625	3.090904	5.018971
6.333014	4.838966	2.23919	2.17366	3.948687	1.143262	1.323482	3.477243
0.2796739	0.1637364	0.1817541	0.1120073	0.09415187	0.125187	0.1411472	0.1097492
1.30738	2.316578	0.5257501	0.5459955	1.799383	0.4107397	0.3486909	1.757134
0.3466826	0.2974279	0.2194496	0.1893324	5.934768	0.299782	0.5426735	0.1553152
0	0.1558537	0.01182782	0.01700763	0.02213634	0	0.005042903	0.06510876
0.8626372	2.035216	0.6428042	0.39018	0.981167	0.7197631	0.3639231	0.2983238
0.07451399	0	0.02594199	0.03730287	0.02427584	0.02084611	0.02212122	0.02040045
8.250327	11.02854	1.251066	0.9606639	6.713693	0.6975653	2.155386	1.57947
1.438873	2.514613	0.9350931	0.9907594	1.384925	0.889827	0.5305816	1.456868
0.002934083	0.003076608	0.004085996	0.002937696	0.01529428	0	0.006968409	0.0128527
7.075429	7.818549	4.582369	6.889387	3.876155	2.6211	6.44829	4.002921
1.181408	1.065941	0.3826107	0.4676435	17.3648	0.7686327	1.566044	1.955721
0.7234436	0.7915674	0.1168076	0.6928415	0.7651386	0.4341148	0.2614605	0.562618
0.6821216	1.294704	0.3066199	0.09941844	0.3881953	0.2560518	0.07177347	0.3120403
0.009982904	0.02093566	0.06951081	0.04497838	0.01300928	0.01117131	0	0.08199363
0.2330766	1.042512	0.0557875	0.1130355	0.1898345	0.03260288	0.01729856	0.2193529
0.0907606	0.08459499	0.03089605	0.03231017	0.02365505	0.03159804	0.01916046	0.03092252
4.048092	6.408175	1.163842	2.484144	8.236269	4.179285	2.263981	3.146106
1.283914	3.064339	0.8079555	0.7691457	1.42112	0.3606922	1.569295	0.8236225
1.220575	6.033375	0.2010205	0.7226355	2.538292	0.1881148	0.394903	0.2581306
0.08332204	0.06814818	0.05569633	0.08843011	0.07166388	0.06713356	0.08311327	0.1204469
0.03856099	0.03144876	0.01789997	0.00428983	0.08375166	0.01438382	0.01526364	0.02815262

TCGA-FG-A711-01	TCGA-DU-6401-01	TCGA-CS-6188-01	TCGA-FG-7643-01	TCGA-CS-5393-01	TCGA-P5-A5F0-01	TCGA-FG-A87Q-01	TCGA-HT-7609-01
0.1830193	0.9594975	0.09930773	0.2689197	0.9177932	0	0.1772711	0.09552729
0.4406989	2.883315	6.843301	1.807501	1.814014	0.9199506	5.556956	0.7293433
0.4076468	0.7877242	1.290289	0.4351538	0.7938063	0.3089879	2.910705	0.575121
0.1573227	2.880754	0.8845744	0.1395908	0.1187355	0.06162348	0.87785	1.6899
0.4006362	0.4783035	0.3551394	0.1845682	0.6923918	1.541135	0.5186848	0.8730288
1.286961	4.241453	3.768415	1.637028	1.361605	1.048279	1.792657	2.874967
1.443981	0.6094907	2.27862	0.7769511	2.7222	0.4441563	1.783804	1.086015
55.61426	33.60215	224.0987	31.2177	68.00327	10.89933	255.7368	76.09042
1.972826	3.467304	5.12781	2.15584	3.108495	0.3863792	10.78414	6.334627
2.15073	4.277962	2.472958	1.353652	4.564408	2.355474	6.289867	4.611198
8.093783	5.507636	10.39963	4.725877	12.60414	2.85591	20.43322	8.61324
4.470843	21.85954	9.802916	4.841443	11.91989	2.131852	16.30004	11.11159
0.3662371	0.7831724	0.7553804	0.9257322	0.6444146	0.6150341	1.594749	0.6595516
4.83989	5.831663	14.33436	3.763666	11.60855	1.178768	24.04891	8.914943
198.3083	118.5195	1182.34	278.194	252.9685	18.39943	985.6306	199.4676
0.4028647	0.2834366	0.6576853	0.3630284	0.812342	0.2828151	0.9501906	0.4961058
2.473948	3.383109	6.442998	5.417864	6.474491	1.860795	9.843317	4.976315
1.378669	3.459742	4.337806	3.587264	3.509853	0.7254595	7.889004	5.139992
0.02875754	0.4606688	0.1560407	0.01564997	0.2495966	0.04318005	0.1740895	0.06671134
0	0.06734685	0.0232346	0	0.07433034	0	0.06221302	0
2.863744	3.043415	4.026138	2.347703	8.835118	1.125635	7.327998	6.204063
0.2477727	1.683253	0.9186965	0.4500249	0.6093097	0.1743918	1.769931	0.5550216
0.08546402	0.1600192	0.3422802	0.1893616	0.08241909	0	0.5986751	0.21773
4.815602	40.67164	18.52	4.628575	27.16612	3.860665	44.09957	38.80464
4.634035	4.23134	8.071618	2.724583	6.257943	4.698397	8.47906	4.92563
6.812164	5.537879	12.19498	5.979809	17.08275	1.778069	28.67127	12.73054
1.70793	3.755098	3.553745	1.394194	7.090478	0.7740392	7.640994	3.291628
0.2369404	0.2338229	0.2873821	0.1934159	0.540332	0.2793849	0.4387472	0.1648953
0.7191308	0.8913841	1.928344	0.8430038	2.157137	0.2966284	2.693035	2.084075
0.3015635	5.187582	1.312455	2.338598	5.94362	0.7004311	1.48328	0.6066521
0	0.09763759	0.1791423	0.03316973	0.05877931	0.01143988	0.1906388	0.004418532
1.031757	1.000366	1.65742	0.8828639	1.34412	0.2598996	6.64591	1.799029
0.02228061	0.03893613	0	0	0	0.1756378	0	0
4.846188	12.39037	9.208157	3.257861	4.906146	0.5926687	9.265831	12.5075
1.473387	1.656732	2.563879	1.816426	1.811056	1.288632	3.845597	1.699341
0	0.01226528	0.02538906	0.005729344	0	0.007903956	0	0
9.112042	12.32385	10.01126	6.83683	8.198454	2.205119	14.42417	8.727854
1.084413	17.74455	4.606249	6.706163	15.11624	2.183362	4.058187	2.08681
0.1128619	0.7341341	0.8278709	1.729995	0.2902423	0.7908347	2.307807	0.4690862
0.175563	0.4737388	0.8078579	0.2023245	0.5328222	0.1221142	1.744252	0.2290886
0	0.02086565	0.01619692	0.04386033	0.005757325	0	0.0289126	0.01038689
0.06969282	0.1560442	0.4057328	0.1600051	0.6216906	0.02943148	0.411352	0.2349303
0.04100937	0.01475461	0.1287126	0.04332215	0.02559006	0.05433215	0.505279	0.04406895
1.686793	2.838553	11.49734	2.090909	4.458351	1.160846	9.076747	2.608666
0.5332927	11.34617	5.229556	1.164377	3.023792	0.2387779	4.527531	4.784544
0.8413911	2.134886	3.687938	0.7528402	1.502696	0.2264216	9.731949	2.110277
0.06577384	0.07314492	0.09733473	0.03254036	0.1672257	0.02693477	0.07963502	0.1057665
0.02049817	0.1746286	0.06024669	0.06693113	0.07412945	0	0.05584039	0.04903734

TCGA-QH-A65V-01	TCGA-TM-A84H-01	TCGA-DU-7008-01	TCGA-S9-A6WD-01	TCGA-P5-A72Z-01	TCGA-DB-A64Q-01	TCGA-HT-7468-01	TCGA-E1-A7Z2-01
0.04017208	0	0.09681625	0.07036904	0	0.1189299	0.1154464	0.1013659
0.8729457	0.5129931	0.1563659	0.3616187	1.384158	1.036074	2.72055	0.2778178
0.5993812	0.3694338	0.2522747	0.3189979	0.2607887	0.3565929	0.4841956	0.417301
0.157645	0.7760891	0.4522981	0.3024447	0.2697536	0.5407905	1.893059	0.01894212
0.5006382	2.653965	0.5508197	0.7854539	1.551575	1.155643	0.7944308	0.3112373
2.639709	1.862102	1.400422	1.459248	1.201721	1.339695	1.57631	1.643564
1.477424	0.8218513	0.6530728	0.9869448	0.4171213	1.02023	0.6656577	0.6438953
37.21058	27.59552	21.99217	20.55193	5.132592	17.94119	21.781	24.35179
2.853825	1.784635	2.074692	1.345221	0.07630173	4.703543	1.68013	1.825263
3.812781	5.082841	3.654265	3.623427	2.692324	3.39797	3.055579	3.410247
9.942763	6.291477	4.44814	5.027372	3.295725	4.39822	3.251036	5.688188
4.718801	6.560282	4.790695	3.377975	3.314647	5.409171	4.152423	3.002291
0.3102684	0.9778039	0.3653821	0.355124	0.4628265	0.4488383	0.5572804	0.8540707
6.000953	4.322351	3.69803	2.984894	0.6940168	3.70325	3.655045	3.257567
381.0735	29.2192	45.85634	130.9242	28.98216	112.2138	225.5752	135.8953
0.6143978	0.4334449	0.3217457	0.301748	0.1347492	0.4759819	0.4166616	0.3235841
2.829504	2.062126	2.136579	1.526411	1.088086	1.975498	3.289794	1.516791
3.372872	1.577706	1.901954	2.102193	0.5548016	2.888529	1.572355	1.950616
0.08416233	0.1640067	0.02535431	0.01842829	0.01543008	0.1038181	0.01511659	0.04424297
0	0	0	0.02469591	0.02067797	0	0	0.02371615
4.677345	3.34632	2.684334	2.605339	0.8439583	2.769465	2.910766	1.709496
0.4418797	0.3346742	0.2129894	0.2679357	0.2741976	0.4025198	0.3272365	0.1944089
0.1272935	0.1539185	0.08073216	0.1408287	0.004913183	0.01983441	0.1925346	0.1014312
22.42002	20.17914	21.26623	10.41738	5.121844	11.96618	18.04977	9.131749
6.377653	10.3383	4.611117	4.246668	7.015561	7.729957	5.892009	4.800298
8.361005	9.136089	5.506113	5.22876	1.733263	4.429106	5.56925	4.019631
2.255403	3.313511	2.277079	1.588315	0.4246746	1.789595	2.496281	0.7370156
0.2875711	0.3689903	0.1290267	0.1822022	0.01346105	0.07849393	0.06813579	0.1235109
0.5414505	1.005236	0.6165178	0.3576795	0.1547907	0.4075347	0.840646	0.5518986
2.461518	0.2545819	0.7477756	0.2717532	0.1668626	0.877748	1.248336	0.9221009
0.005574374	0.02286895	0.2216685	0.02441144	0.01635183	0.01100199	0.128157	0
0.730058	1.870393	0.5535741	0.2740362	0.1056205	0.4410907	0.6815059	0.9691132
0	0.2206975	0	0.08566666	0.01793225	0.04826139	0.03513587	0
6.080696	2.613093	1.991339	2.283467	0.2647323	3.309068	3.187171	2.509999
1.28566	0.9107925	0.7706296	0.7690731	0.8043271	0.922123	1.045093	1.270884
0	0.006320178	0.01624356	0.003373234	0	0	0.01936927	0
15.08556	5.394534	2.737014	6.364045	1.224885	11.42245	3.02675	8.617038
7.60958	1.213228	3.063807	1.042364	0.4231638	2.847171	5.311636	2.062689
0.3440664	0.225846	0.9287189	0.5665359	0.4238985	0.2308854	1.146982	0.1389089
0.232346	0.3440831	0.1809636	0.2134259	0.04155867	0.1286248	0.2809291	0.05719777
0.006551995	0.005375932	0.00789528	0.005738533	0.004804895	0.03232875	0.01882911	0.01102174
0.09082784	0.05883515	0.07200611	0.05861662	0	0.1132197	0.106469	0.06031192
0.07148177	0.06516779	0.04306845	0.07651962	0.02135672	0.3265779	0.1635785	0.02894819
3.805295	1.406422	2.127485	2.70231	1.759842	5.176094	5.024487	1.153378
0.8743927	1.695246	1.372307	0.7596565	0.3102747	0.8420067	1.079097	1.364141
1.861214	1.133541	0.539023	0.4180372	0.1882039	2.374	0.6341303	1.589671
0.07656047	0.2351192	0.07512343	0.07088684	0.05133302	0.0626008	0.1100097	0.05887529
0.07311318	0.02307292	0.02033141	0.01970333	0	0.03885042	0.04040625	0.009460821

TCGA-DH-5140-01	TCGA-TM-A84G-01	TCGA-HT-7485-01	TCGA-TQ-A7RF-01	TCGA-19-5960-01	TCGA-QH-A6X5-01	TCGA-TQ-A7RS-01	TCGA-DH-A7UR-01
0	0.07411133	0	0.08424809	0	0.4627568	0.1380229	0
4.153986	0.8052255	2.09018	1.459632	5.062311	0.1811852	0.3850401	1.058388
1.692218	0.2301475	0.4617555	0.5352819	0.6658703	0.3386173	0.2414069	0.9488078
0.8023764	0.4708689	0.8830636	0.8343965	0.779935	0.3963426	0.3352981	0.4911081
0.2932098	1.176587	0.1664335	0.8079885	0.2595792	0.7731169	2.26603	3.612912
1.305068	1.030892	1.673573	1.632026	3.307778	1.513807	1.63722	1.678861
1.021372	0.2045865	1.215089	0.9265262	0.6108246	0.649029	0.5162157	2.771582
26.33966	5.066072	55.71635	44.7986	27.98077	22.31432	10.0423	6.195392
1.606841	0.1462464	1.737538	2.374252	1.454748	1.726469	1.080949	0.1991092
2.696337	3.119976	4.226464	4.758497	3.181837	2.729958	3.474181	5.864855
5.858594	2.750287	7.934374	8.807401	4.048935	6.224173	4.760002	5.764266
7.342008	3.450585	7.328608	8.48823	4.098422	4.344151	3.611813	7.926844
1.646797	0.4488118	0.2618107	0.3401327	0.566989	0.399378	0.3301021	0.3558745
3.868612	0.8679052	7.601277	4.640533	4.749377	3.84915	2.07214	1.155421
127.5705	4.184656	135.8988	29.55747	150.9494	281.7163	114.1054	23.31902
0.496035	0.1906772	0.4728192	0.4937254	0.3683189	0.2976507	0.2860624	0.3756489
2.371948	1.328276	2.93861	2.247292	4.936284	1.601929	1.658797	3.323591
2.162651	0.3655368	2.387067	2.663195	1.891781	3.920782	2.943538	1.07319
0.1001234	0.01940833	0.386731	0.1103147	0.09504462	0.02019785	0.01807277	0.03932837
0.06192748	0	0	0	0.05094804	0	0.04843894	0
3.020227	1.073967	5.304208	3.514388	2.948873	3.042865	2.214013	1.427771
0.5026626	0.2947263	0.6797369	0.6700764	0.5220468	0.3458708	0.3678732	1.029257
0.1765711	0.01235984	0.0935874	0.2107558	0.1755296	0.1800769	0.04603726	0.03130695
26.98692	4.529004	30.73606	24.11103	18.50078	7.29758	8.785349	12.20906
7.679932	4.608269	3.853324	6.035937	7.468221	4.921314	6.952938	8.573879
6.676852	1.443072	11.61217	9.926177	6.508164	4.984366	2.719094	1.937063
2.047212	0.365482	6.268644	1.965501	1.810456	1.338538	0.7853812	0.6907522
0.08958636	0.06208267	0.2833996	0.08019794	0.1630682	0.1409633	0.09985457	0.08863338
0.987617	0.2581885	1.663163	0.8083358	0.9741916	0.4052396	0.268011	0.3216298
0.1968629	0.4006874	0.1216626	0.9326749	0.6914449	1.628235	0.1599063	0.1159913
0.146914	0.0154258	0.012295	0.06429756	0.04532505	0	0	0.01562916
0.8871444	0.06871659	0.7120096	1.791448	0.7358404	0.8915155	0.4137887	0.148528
0.01790148	0.04511124	0.05393329	0	0	0	0.02100349	0
1.703125	0.5697773	3.951574	2.666546	2.44963	2.017589	0.7303934	0.3748628
3.859482	0.7641256	1.042676	1.761603	1.095659	1.075124	0.9733912	2.588922
0.002819574	0	0	0	0.03827468	0	0	0
4.704209	1.462183	2.651527	14.16755	4.997909	8.129158	7.191622	2.813778
0.7656684	2.295396	0.4507527	2.306821	2.508815	3.912046	1.115187	0.5055775
2.216611	0.2285099	0.3440269	0.8803145	1.964532	0.4227655	0.2009639	0.836053
0.692839	0.01568205	0.8999457	0.4040788	0.5375767	0.1414399	0.03407347	0.100629
0.0671531	0.01208743	0	0.06870357	0.3433217	0.006289568	0.005627823	0.006123388
0.2939742	0.004409564	0.2917123	0.2155458	0.1295646	0.05506732	0.004106126	0.008935392
0.1918806	0.07570482	0.03892895	0.122149	0.1650366	0.04828724	0.02274042	0.08412569
2.735624	0.8854289	3.629435	2.767973	2.818425	1.579625	1.648999	2.178675
1.321567	0.2471717	1.887111	2.225651	1.554442	0.8258325	0.7147164	0.4217771
0.7058705	0.2212406	0.4390814	1.584458	0.6955675	1.114364	1.040382	0.1748426
0.08167163	0.04842594	0.1109678	0.1399175	0.09881123	0.06299486	0.03945688	0.1226609
0.008234681	0.01037559	0.03721397	0.005897366	0.0304862	0.01079766	0	0.01576855

TCGA-DU-6407-01	TCGA-QH-A6X3-01	TCGA-TM-A7C4-01	TCGA-FG-A70Y-01	TCGA-27-2528-01	TCGA-FG-8191-01	TCGA-DU-7306-01	TCGA-TQ-A7RM-01
0.1016335	0.03680952	0	0.2803084	0.5523843	0.3964482	0.3019546	0.07778934
0.8157569	82.517	0.1710125	2.263602	4.866242	3.689534	1.241369	1.012702
0.3531027	0.4887711	0.305871	0.9580262	1.354849	0.6901362	0.5428267	0.2906237
1.943527	2.531305	0.03109317	0.4910706	1.032233	1.27652	1.754331	0.751047
0.1101384	0.3590083	0.7032261	0.5999362	0.2522709	0.7105316	0.1725356	1.483661
2.278154	3.103545	1.810422	2.523653	2.747543	2.097583	4.202546	1.155076
1.197669	0.9341909	0.6519464	2.689578	0.7764907	0.9531931	0.9264434	0.4294796
59.16794	7.157117	25.14142	76.08254	33.51151	32.36589	44.70514	14.27836
1.616989	2.360717	0.7439042	6.300632	1.513401	8.045153	1.641993	0.7035614
4.579441	15.07306	4.088503	14.13874	5.105694	5.457611	3.429933	5.024809
8.5156	5.429957	3.799869	18.71226	8.480917	7.806707	6.548425	3.688996
8.99803	7.817301	2.889411	14.32816	5.199101	17.74324	7.887454	2.624981
0.1189341	0.8076639	0.4490598	0.4889605	1.627578	0.8912876	0.1541912	0.3504694
7.270449	2.070138	2.481029	10.83559	3.008781	9.14013	4.276261	1.57126
170.3307	52.23183	27.44275	383.1356	158.0109	422.7399	78.66878	52.43601
0.5762412	0.4366965	0.3091815	0.5158518	0.2763446	0.4925645	0.3335105	0.2668535
3.489883	2.083996	1.930049	4.199289	4.279304	3.629336	2.972607	1.107539
2.031717	5.558038	0.5782079	4.438317	1.618781	7.148105	4.04332	1.1365
0.1064634	0.2988307	0.04357446	0.2752776	0.2583192	0.5510559	0.244417	0.03395255
0	0.02583649	0	0.02459345	0.08308219	0	0	0
4.455287	5.451332	2.104581	7.296381	1.98962	5.492143	4.28603	1.112062
0.6650283	0.5979953	0.2674968	0.6107307	0.6676974	2.064284	0.4459505	0.2193993
0.1242987	0.06752757	0.04856181	0.1519315	0.1908269	0.2339533	0.1419185	0.0648662
36.13027	29.22781	9.962609	36.39617	22.19378	21.04862	20.37901	6.879614
7.378048	4.555181	4.089382	5.612584	11.37955	5.238748	3.112047	6.839838
13.27579	5.369261	4.523817	16.86289	5.872567	8.861809	7.53538	2.790269
3.938985	3.323343	2.303878	4.751823	2.305006	3.863915	3.576957	0.7524859
0.1625359	0.8381551	0.2787689	0.6964337	0.3335258	0.3158458	0.1170658	0.03554385
1.168625	0.6516948	0.5701672	1.480813	0.7841786	0.5399173	0.84971	0.515348
0.5407658	0.1326753	0.02141905	6.007892	3.575672	2.009948	1.272106	0.7877395
0.009401941	0.06129332	0.0404053	0.0291722	0.1478253	0.0126951	0.09141806	0.007196157
0.280612	5.196871	0.8948155	1.425141	0.7511899	1.436428	0.5769174	0.2532455
0.04124259	0.04481158	0	0.04265563	0.04803342	0.01856279	0.01670895	0
6.061548	1.190789	1.819166	11.22303	2.308557	13.53762	4.544261	0.8595413
1.022744	1.193226	1.084241	2.213834	6.115193	1.650126	1.372119	0.8255745
0.009743877	0.003529026	0.01196423	0.003359239	0	0.002923734	0.01315872	0
6.046952	11.96095	3.403987	14.73525	12.3507	16.51571	7.78573	1.229172
2.28103	0.7600495	0.1120324	14.34382	10.41393	7.966912	6.111596	2.374395
0.1392756	0.5548694	0.3277739	0.3241066	1.662633	1.211934	0.7523448	0.5596511
0.372767	0.1453936	0.2112507	0.3163393	0.3784855	1.088405	0.3717468	0.1280248
0.01105083	0.1680999	0	0.01714418	0.2702787	0.01989538	0.08506518	0
0.2620416	0.4029851	0.03465035	0.4252922	0.09390406	0.1923358	0.1731275	0.03702723
0.03125727	0.05822085	0.06853553	0.03001903	0.06500706	0.03617619	0.09407182	0.04784803
3.180164	2.952766	1.703926	5.113088	10.00025	5.387093	16.46817	1.482568
1.635343	2.326072	0.8616107	3.622613	2.957343	18.0827	2.279138	0.5461861
0.7463668	0.5472297	0.208621	4.980991	0.8904618	6.394518	0.9063258	0.5201737
0.03504948	0.1683649	0.05209651	0.08585605	0.1181648	0.1046155	0.07174679	0.1073062
0.05217188	0.005153332	0.017471	0.05886476	0.03314306	0.0811194	0.065332	0.01089051

TCGA-HT-7602-01	TCGA-HT-7695-01	TCGA-DU-8164-01	TCGA-WY-A85E-01	TCGA-DB-5278-01	TCGA-QH-A6CY-01	TCGA-FG-7636-01	TCGA-P5-A5F4-01
0	0.09218257	0.05200016	0	0.141928	0.2709289	0.07308591	0.2537102
1.354518	0.6090634	1.31321	0.2466764	2.483966	0.7558055	0.482889	0.8443583
0.5645069	0.2994282	0.3136851	0.3094415	0.3911001	0.3384744	0.3156614	0.395449
0.4280511	0.499555	0.9328514	0.4897647	1.395052	0.2531406	0.5940999	0.1027227
0.4318588	0.499483	1.000242	1.500222	0.5290887	0.6128915	0.3326479	1.154753
2.474282	1.412064	1.136019	1.846645	1.685965	1.393007	1.646564	1.50114
1.025494	0.5397284	0.3889687	0.3770141	0.9352562	0.6091824	0.8753599	0.613769
68.49293	20.68733	15.30842	18.66157	26.36363	20.17362	34.74909	19.38662
1.644772	0.633832	0.4104546	2.121812	1.515886	0.7225898	1.825321	0.5684518
3.840956	2.204202	2.170049	2.622001	4.186984	1.345356	1.435605	3.25824
7.805121	2.880396	2.459312	5.13817	3.793102	4.196229	6.332305	5.50038
8.299748	2.906749	2.410372	3.623447	5.419224	3.037122	9.077596	5.118218
0.5893598	0.4854345	0.6069975	0.7697105	0.2914839	0.4369313	0.1571558	0.7348225
8.699679	3.157011	1.685275	2.937519	3.857607	2.79926	5.74247	1.577708
224.1345	95.48057	18.46996	48.05444	74.12689	156.7501	66.10965	46.78
0.4619713	0.3491695	0.2489953	0.35541	0.4463057	0.3436886	0.5484468	0.3565991
3.716	3.352576	1.992443	1.770097	2.852788	1.928698	2.925847	2.882711
3.879766	1.196089	0.7927521	2.523626	3.236033	2.148699	3.723586	1.573688
0.1890025	0.04224646	0.006808919	0.03519815	0.05946913	0.03547552	0.08612905	0.1550309
0	0	0	0	0	0	0.02564939	0
6.613131	2.227691	1.842491	2.246051	3.604605	2.626858	3.238543	2.847758
0.5097695	0.2612944	0.299192	0.2786242	0.5668219	0.2922819	0.4205126	0.3935644
0.04186533	0.06149462	0.01734455	0.06164209	0.1893592	0.02259194	0.2864375	0
24.88118	8.836134	8.640684	9.893256	23.52911	6.594769	16.8115	15.00504
5.077446	3.865076	4.297073	7.570873	5.361522	4.356366	5.786624	4.286058
10.96209	3.579361	2.054783	3.518484	6.543891	4.710214	8.285202	3.606029
4.850672	1.787805	1.740834	1.383006	2.702775	1.496681	2.793295	1.475749
0.341716	0.1597068	0.1108807	0.08700184	0.1426708	0.4178048	0.1558421	0.2737151
1.175608	0.6528193	0.6860237	0.4029936	0.7457246	0.5067445	0.4591439	0.3525835
0.9855935	0.3678598	0.281142	0.1211117	0.7892665	1.447355	0.09408176	5.748074
0.01306259	0.009593605	0.01803917	0.0139878	0.04726625	0.04229408	0	0.02347029
1.454733	0.3447386	0.3375046	1.242062	0.6246459	0.632205	0.6189278	0.5645809
0	0	0.04747841	0.02045298	0	0	0.04448708	0
4.311064	2.917681	0.9553275	2.952357	2.516755	1.034705	2.787577	1.579022
1.428714	0.8173864	0.6069197	1.24998	1.266676	1.265417	1.308168	1.335843
0.006016736	0.002209447	0.002492697	0	0	0	0	0
3.880735	4.412534	2.547636	5.55267	5.149317	6.008983	6.280718	5.677479
1.831064	2.296496	0.8636352	0.1809929	3.771497	3.496367	0.06561265	13.55217
0.6557586	0.4105538	0.1068892	0.5410435	0.9724698	0.6497275	0.2253482	2.071575
0.4824916	0.2243184	0.1210363	0.2133025	0.1561672	0.07166112	0.3711618	0.1849164
0.01023565	0.007517407	0	0	0.01851857	0.011047	0	0
0.1344249	0.07130225	0.08353713	0.04398354	0.1182244	0.06448019	0.1174109	0.03019111
0.05997102	0.04556352	0.01713491	0.03100214	0.03180199	0.06918854	0.01926641	0.04180085
4.257694	1.376664	1.309085	1.978548	2.47081	2.54325	3.056123	1.587744
1.057543	1.072001	0.502028	1.038077	1.34531	0.6717447	1.969258	0.6160633
0.7081711	0.1885036	0.1521286	1.49058	0.2237084	0.535823	0.9076266	0.4165631
0.09055748	0.05897921	0.06512456	0.1152681	0.1112866	0.07929503	0.06566438	0.05295741
0.02635818	0.04516946	0.0327601	0.02822511	0.03576586	0.01422376	0.005116014	0

TCGA-HT-A5R5-01	TCGA-FG-5963-02	TCGA-DU-6393-01	TCGA-S9-A6TY-01	TCGA-DU-5855-01	TCGA-QH-A65Z-01	TCGA-S9-A7QY-01	TCGA-HT-7680-01
0.08486393	0.4973725	0.09489046	0.06800469	0.06131222	0.1194008	0.0360213	1.429834
1.096496	14.44605	1.037959	0.4293471	4.204023	0.7947418	0.4442627	4.807105
1.102623	1.139453	0.2895951	0.2645866	0.9366845	0.312544	0.3805866	0.8165976
0.5709028	2.306685	0.5009306	0.4511321	0.8363854	0.245435	0.3702189	1.057859
0.3632638	0.6026928	1.701854	1.798169	0.810604	1.526831	1.651207	0.2561394
10.50104	6.374412	0.7126008	1.537892	3.997436	2.391111	1.684539	1.180345
1.122226	1.091161	0.5217833	1.392828	1.76079	0.510363	0.9398481	1.239477
103.4062	20.07214	6.986692	6.014887	85.63458	16.07882	19.10938	88.13861
10.17872	6.864795	0.7987404	0.566139	5.629791	0.6234048	0.7063776	6.506814
3.911018	4.767077	2.952538	2.13927	7.128542	2.128247	4.830412	1.801626
17.0037	5.895548	2.781984	1.909803	15.99339	4.691997	4.524412	10.05441
29.10012	5.684401	3.971025	1.823992	16.27201	3.613095	3.904227	8.019263
1.31089	2.92077	0.2894063	0.5252331	0.8986573	0.3807527	0.4236378	1.83372
15.49099	2.764551	1.05633	1.569632	10.19417	3.213882	3.310948	8.602738
335.4541	125.9788	38.82917	64.92074	89.22418	101.9457	158.7505	181.059
0.3881635	0.6398318	0.2475295	0.184686	0.727389	0.2218667	0.3861555	0.5297059
6.083968	3.472549	3.45702	1.870959	5.663862	1.423324	1.57478	5.083267
8.742435	1.829743	1.273772	1.112975	5.17874	1.913978	2.70134	3.114012
0.2666907	0.1894578	0.04348746	0.008904557	0.329158	0.02084584	0.01886656	0.3133119
0	0.03173675	0	0	0	0.02793569	0	0
8.224637	1.817986	1.104836	1.213329	8.854104	2.287022	2.781958	4.184596
1.421755	0.3749312	0.2569265	0.1553599	0.8352363	0.3771728	0.225542	0.6419472
0.03538274	0.2714688	0.01582526	0.05670706	0.4652504	0.0265506	0.04205191	0.8857053
49.26216	22.25436	6.365686	3.969044	70.41533	11.68562	11.00151	36.18556
7.038704	11.43937	7.995854	4.422336	6.351946	6.318624	4.501629	4.858305
18.23508	4.507225	1.988693	1.775055	17.61107	3.163609	3.653843	13.72772
10.5593	1.389351	0.4589557	1.070594	4.948285	1.004028	1.619257	3.304243
0.1971134	0.05853709	0.08671554	0.1709015	0.424895	0.1273	0.2139671	0.2311085
1.20198	0.723052	0.3034821	0.2369142	2.486153	0.4091485	0.3085838	3.943
4.0857	4.330457	0.2320855	0.2801306	0.5524804	1.393563	0.148382	4.222091
0.07065553	0.1317589	0.03291807	0.009436493	0.08082439	0.01656834	0	0.3482248
2.082569	1.570765	0.2404873	0.1975707	2.171638	0.605211	0.5655602	3.07719
0.0516563	0	0.1877181	0.1862737	0.03732048	0	0	0
30.07176	2.627495	0.3031812	0.5228286	12.98427	1.692877	1.43863	9.439833
2.530472	5.396693	0.7924789	0.5889769	3.416194	1.214669	1.426168	5.167633
0	0.004334947	0.0272922	0.009779686	0.0176345	0	0.003453458	0.002797592
20.60883	4.226926	3.398762	1.440754	24.88333	6.25031	7.269009	19.61291
12.11363	15.10031	1.213924	1.068393	2.50445	2.715523	1.067154	17.81363
1.16295	2.277112	0.3494693	0.1863832	1.176286	0.5045052	0.7527784	2.019384
1.664045	0.3508155	0.07027631	0.08154266	0.8995131	0.2358101	0.2083389	0.568062
0.006920578	0.567844	0.01934558	0.005545722	0.07499937	0	0	0.03807402
0.09593737	0.3820221	0.1016263	0.04046224	0.5399081	0.02368084	0.05572419	0.5139162
0.1174491	0.1370737	0.02814118	0.02913127	0.08889492	0.1049188	0.08308729	0.1211539
2.896838	4.167291	3.664226	1.27674	3.845701	2.037878	2.059564	6.524269
19.63356	2.762033	0.4747095	0.310365	10.0359	1.397258	0.7018477	7.667819
6.447497	1.00965	0.3087659	0.1461678	2.648475	0.6534748	0.3225974	3.555844
0.06007292	0.05416565	0.06846217	0.06480211	0.1235268	0.08018626	0.1118012	0.1144022
0.2851428	0.1645851	0.009963498	0.01428098	0.06866969	0	0.02017193	0.1266424

TCGA-P5-A5EV-01	TCGA-HT-7482-01	TCGA-DB-A4XF-01	TCGA-FG-A4MT-02	TCGA-DB-A4X9-01	TCGA-HT-A5R9-01	TCGA-S9-A6TX-01	TCGA-S9-A6WE-01
0	0.09600692	0	0	0	1.280985	0.06820222	0
1.753898	2.048656	1.156237	1.685393	0.3137106	4.079011	0.9913679	1.40564
0.5323399	0.9435476	0.5563877	0.4379921	0.4298625	0.3915139	0.5745304	0.4401405
0.5546764	1.363492	0.9936917	0.5769434	0.05444565	0.5909596	0.8284158	1.15927
0.7217869	0.3468032	0.3783068	0.4632631	2.010206	3.014955	1.866215	1.095567
1.791671	2.059177	1.482616	1.276835	3.729572	1.024841	1.978876	0.9150232
0.9767032	2.849784	0.4193074	0.9516922	1.794544	0.87692	1.415093	0.7945471
48.01169	84.12807	45.47479	36.96965	50.39537	5.472817	19.62638	16.98527
1.446488	5.352066	4.706002	3.754126	1.790717	0.5035874	1.488854	2.229233
5.925583	7.685819	4.172398	2.54069	6.238583	1.846808	5.190203	5.030291
8.462143	11.52058	4.885588	6.443665	9.913195	2.563426	6.288033	4.933986
8.623561	13.70282	7.526328	5.553307	11.90902	5.263826	8.039495	6.894807
0.3558262	0.5954533	0.3028261	0.9379861	0.2685014	0.9732054	0.508801	0.17477
5.752404	14.98659	5.477807	5.945205	5.772699	1.270104	2.985855	3.024477
62.52949	291.8258	28.19279	166.9977	41.17175	7.915576	161.156	169.9213
0.4760129	0.7959249	0.5221892	0.4695931	0.5066871	0.3318668	0.4386848	0.3065227
2.655727	7.228568	3.205868	2.850956	2.53369	1.988103	1.747873	2.09108
1.838151	5.126339	4.786889	3.407666	7.82759	0.6686121	6.823395	4.211102
0.1801135	0.3687546	0.08504275	0.3109759	0.4323722	0.06289975	0.07144337	0.1403992
0	0	0.05698325	0	0	0	0.02393547	0.02687859
4.160099	9.907715	2.672555	3.434963	5.897985	1.743644	2.953584	3.10505
0.6493242	1.516372	0.4190252	0.4889803	0.2958314	0.2777439	0.3981842	0.959094
0.09055426	0.1547774	0.4941912	0.2244447	0.01619697	0.02670438	0.06824613	0.2171402
32.82876	78.94823	12.654	15.11141	17.48502	10.84075	11.59517	9.823539
6.100446	8.650104	4.31277	5.599237	4.931942	4.766417	5.552652	7.739702
11.8044	20.24042	8.822788	6.760151	10.55449	2.556529	5.470576	5.477845
4.524627	9.359954	1.547562	1.681737	2.238153	1.138921	1.70111	0.7553955
0.151616	0.414308	0.1576548	0.4280392	0.277351	0.0762127	0.1324439	0.1253992
1.153575	2.99742	0.7650331	0.6781813	0.5602079	0.3795113	0.3388755	0.4680252
0.4286955	0.9886995	0.1881127	0.7133487	0.2000309	10.22369	0.5443296	0.8675959
0.02511488	0.0266443	0.02253075	0.03295529	0.006738251	0.1777526	0.03312366	0.02656897
1.154582	0.767536	2.704882	1.585487	0.4922718	0.4107599	0.3836415	0.3077245
0	0	0	0.02409363	0.02955804	0.02436657	0	0.04661903
2.884177	18.24514	2.107546	3.841476	5.769686	2.006447	1.716046	3.976456
1.352563	1.73955	1.61719	1.501862	1.253689	1.225006	1.268572	1.143436
0.01041131	0.003068146	0.01167508	0	0	0.007675717	0.009808092	0
2.636228	11.78032	6.256446	10.18895	6.056995	1.94002	12.76964	10.06018
2.567263	3.878546	0.2550914	2.842798	0.6975082	34.17658	1.867464	4.194177
2.405853	0.5701152	0.5006386	0.8407594	0.08318085	1.988567	0.5023599	0.6953234
0.561706	0.686202	0.3034917	0.2903571	0.06165169	0.08470568	0.1972329	0.1404538
0.01180779	0.00521952	0.1324107	0.01291164	0	0.0130579	0.04449465	0.04372002
0.2369153	0.3160825	0.4782116	0.1836994	0.2080267	0.190544	0.07710157	0.08658202
0.05009747	0.06748985	0.09898121	0.06260666	0.06720505	0.05803956	0.02696851	0.03028457
3.923143	6.96406	3.775821	3.41165	1.947665	1.913037	3.172021	2.516314
2.173081	6.426402	1.189928	5.065125	2.974825	0.8502372	1.173235	5.962346
0.3846976	1.364237	1.648022	2.467248	1.62358	0.3226548	1.184955	1.088304
0.08869805	0.3014669	0.06630974	0.03448532	0.1084105	0.02397724	0.05756287	0.08132234
0.02533888	0.0985671	0.0227317	0.01662461	0.04758844	0.1737336	0.01909662	0.01608356

TCGA-HT-8558-01	TCGA-HT-8010-01	TCGA-HT-7694-01	TCGA-DU-7299-01	TCGA-E1-A7YU-01	TCGA-P5-A77W-01	TCGA-DU-6400-01	TCGA-WY-A85A-01
0.2372723	0.03210027	0.2917061	0.09934762	0.3593674	0.1284652	0.3705911	0
0.5574016	1.008611	0.4454306	0.9043787	0.1289793	1.25432	2.981788	0.9616582
0.2074988	0.3758245	0.2936278	0.6063956	0.3791231	0.5869456	0.2980732	0.464283
0.01108469	0.1859546	0.09266825	1.596586	0.02984645	0.4681193	0.9602926	0.2068612
0.26677	1.060988	0.587977	0.5060078	1.977489	0.8457335	0.9397508	1.239609
1.457876	1.386118	0.7667209	1.805835	0.8927677	1.20044	1.695122	2.243609
0.3618331	0.731778	0.4519865	1.565891	1.2445	0.4404299	0.5786156	0.9487982
17.94793	39.12398	7.582404	65.97903	28.12451	5.06798	15.86349	44.64345
0.6108774	1.536105	1.269991	2.563914	4.712903	0.2891537	1.069526	1.342529
1.244974	3.949686	1.749653	3.599137	8.540673	3.153469	1.473409	5.932498
2.896938	5.438629	3.330855	9.933696	10.60365	3.062614	4.6967	7.857397
2.468095	4.397618	3.462627	8.198948	6.634566	4.598599	4.709951	6.344255
0.6273425	0.7634991	0.3174664	0.3073598	0.4660991	0.732874	0.5637774	0.4331572
2.427285	3.601846	1.779561	10.41011	5.845125	1.30326	3.014401	4.905367
130.9694	55.4002	165.5045	173.2992	165.638	42.68367	75.408	44.09966
0.3391475	0.3349447	0.2876976	0.6212653	0.6905944	0.2524816	0.3496073	0.5142418
1.818269	2.234085	1.71992	4.67194	2.411134	1.425579	2.369187	3.136731
3.145155	2.180596	2.403403	2.714538	3.424139	0.7380284	1.384738	1.969926
0.00776713	0.04203221	0.03055688	0.3447282	0.1045682	0.05046382	0.01294009	0.1811865
0	0	0	0	0	0.04508466	0	0
1.580063	3.076061	1.392774	6.491011	3.806262	1.275158	2.446141	4.072228
0.135515	0.2716096	0.33074	0.7061113	0.452728	0.673928	0.4515376	0.5151593
0.04946356	0.09100933	0.04864899	0.1532595	0.08657007	0.01606849	0.05768463	0.03846179
2.802229	9.689346	12.154	24.68733	20.22298	7.047908	14.81309	23.30972
2.28187	3.247185	3.390187	5.995482	8.021494	5.889686	6.471788	5.858113
2.640052	5.536123	2.847785	15.612	8.867438	1.98744	4.40964	7.202539
0.9282161	1.473439	0.9018643	6.958026	2.393268	0.8954695	1.813522	4.339313
0.15133	0.2371229	0.1488379	0.1910346	0.4561215	0.1051688	0.0827846	0.298568
0.2710189	0.8396468	0.3765099	1.509251	0.5381838	0.2274414	0.4938491	0.9430543
0.6108701	0.925611	2.012714	1.457921	2.775631	0.8103139	2.925926	0.5699992
0.01646224	0.06236035	0.0242867	0.02757143	0.01108148	0.0267392	0.03428274	0.01280068
0.4766682	1.321505	0.2921099	0.8075491	1.25385	0.2421982	0.4001205	1.283008
0	0.03907859	0	0	0.02430504	0.0390981	0.0751924	0
1.75903	2.48073	1.001922	4.200884	2.623323	0.4040413	1.692223	4.034271
0.9810058	1.46686	1.077938	1.446346	1.505159	0.9563189	1.0658	1.327782
0	0.009232616	0.005593328	0.00476236	0.0114845	0	0.004737274	0
5.647378	8.688014	5.523476	5.242238	10.45811	1.309806	2.826496	6.2052
2.715884	2.881792	5.970825	4.660126	7.169354	2.162423	8.184347	1.490691
0.1727364	0.8138013	0.3697645	0.6211526	0.2325536	0.4291094	1.785929	0.7742928
0.1631732	0.1901889	0.1069907	0.6902251	0.1915147	0.08155019	0.3310964	0.6831996
0	0.010471	0	0	0.006512465	0.047143	0.04432469	0.01504563
0.05647005	0.04583857	0.05206884	0.05024438	0.1140377	0.01146536	0.04409975	0.005488735
0.03322869	0.1163533	0.0288366	0.02946301	0.021052	0.02963198	0.04884636	0.0395168
0.936483	1.588831	1.593186	5.12925	1.976356	2.110363	1.771023	1.456378
0.4060806	1.267803	0.5274039	1.569497	1.541982	0.6595866	1.465821	1.983617
0.4940507	0.7187042	0.7785083	0.7028878	2.865568	0.1399776	0.6269067	0.4268479
0.0549096	0.08390005	0.03653302	0.1054883	0.08914402	0.06820281	0.02286993	0.1054855
0.03737039	0.01797615	0.0285872	0.04868033	0.03913111	0.004496281	0.0276708	0.02582969

TCGA-FG-A60K-01	TCGA-QH-A65S-01	TCGA-DU-7309-01	TCGA-E1-5303-01	TCGA-E1-A7YH-01	TCGA-S9-A89V-01	TCGA-FG-A713-01	TCGA-HT-7684-01
0.714992	0.09538038	0.5081776	0.1739259	0	0.1123969	0.1030684	0.08044736
0.817731	0.6021833	1.162398	3.595574	0.2459773	2.607429	0.2118629	0.5905858
0.3384937	0.4970664	0.4868941	1.296276	0.8635022	0.7435502	0.2428131	0.9045339
0.6258556	1.007034	0.3498617	2.398597	0.3631519	0.5880969	0.147662	0.3081784
2.140961	0.7958874	0.4579529	0.3354951	1.724723	0.438489	0.5919751	0.6058965
1.592833	2.018607	1.693584	1.407064	1.749912	1.464332	1.54803	5.293065
2.164765	2.586289	0.9312565	2.667032	0.9279293	1.070949	0.4558494	0.6913052
36.678	59.99384	42.8013	128.5565	37.61126	71.86116	14.7321	23.14763
3.745429	4.746605	1.72192	12.09829	7.479677	4.158685	0.5296573	3.98858
4.247281	6.299537	3.386429	9.713912	8.273188	2.238023	2.010256	3.278481
4.778877	11.41351	4.217851	23.78343	10.03007	9.428995	3.949473	14.75331
7.774401	8.311035	6.147348	16.99961	8.203388	6.663172	3.090521	11.04865
0.4260571	0.3474063	0.9507087	0.3846763	0.5940263	1.71975	0.2894718	0.2647729
5.242187	10.97501	4.461565	23.20077	8.157941	6.504778	1.866666	7.580662
111.3222	376.0555	34.95566	1282.016	332.6011	255.6047	101.3196	622.0776
0.7046289	0.7498309	0.3784763	1.422008	0.8333254	0.4819668	0.2750009	0.3794611
3.438447	3.390992	4.213478	7.830936	3.207451	5.804901	1.346605	3.203704
7.698467	4.634917	2.095718	6.854993	5.438588	2.288882	2.518697	3.120195
0.1872426	0.1373805	0.1961207	0.3643828	0.06142219	0.2256651	0.04498606	0.1474733
0	0.0334736	0	0.02441562	0	0	0	0.02823288
3.454764	5.736458	4.077496	10.12825	4.04715	5.197011	2.02599	6.698205
0.8278609	0.7989704	0.348513	1.712986	0.4195869	0.678396	0.2034881	0.5921987
0.1004144	0.3499533	0.0847508	0.4234923	0.1341107	0.1874488	0.01145942	0.1073322
8.785259	24.97859	21.52373	64.89035	18.58931	13.16108	7.199744	70.00895
9.359675	8.396285	3.696355	7.936736	7.153873	8.914347	3.454406	5.99287
7.370436	14.64693	7.04039	35.50726	10.36211	12.28047	3.440427	7.718804
0.9493083	4.097654	3.373584	10.71491	3.126777	3.503386	0.8471413	3.067015
0.2550532	0.2251717	0.2484934	0.2543072	0.1811654	0.2539313	0.5599011	0.2297398
0.6662416	0.7789643	1.390035	3.214366	0.9299991	4.827192	0.3257129	0.8867303
4.882923	2.038163	2.83701	1.361256	0.241537	0.4629947	0.5660909	6.296325
0.02610895	0.006617605	0.02597955	0.02896126	0.009298771	0.1247717	0.004767341	0.005581534
1.037452	0.6898119	1.058102	2.322223	1.789465	3.663745	0.5776424	0.4226846
0	0.02902881	0.01628029	0	0.1223701	0	0	0.07345194
5.124975	6.742518	4.310178	18.40077	3.412351	3.366707	1.262357	10.72316
1.198133	1.990443	1.1869	2.834793	2.412727	2.367184	1.258223	2.777009
0.003607799	0	0.005128456	0.006669897	0.003212318	0.00359193	0.006587631	0
7.803155	9.636393	7.316832	13.80883	14.25938	7.272738	8.015697	19.70672
15.60787	6.165176	13.20622	9.493402	0.4211199	1.412656	1.542156	11.73597
0.8379901	1.029313	0.4673153	0.5839403	0.1607051	1.655773	0.4943468	0.9232824
0.1274048	0.4171074	0.4603083	0.966691	0.4112161	0.6553628	0.12601	0.243993
0.0245503	0.02333456	0.04798482	0.01134681	0.0710422	0	0.005603427	0.05248329
0.2328585	0.1759267	0.257803	1.088657	0.2671405	0.3388346	0.04497159	0.1483831
0.02480018	0.01571471	0.0511172	0.03209443	0.03091427	0.2567873	0.06113296	0.06097009
2.344291	4.354657	2.099862	7.658701	2.430434	3.964567	1.524575	8.581493
3.672679	1.741217	5.164343	11.15561	1.25863	2.091321	0.5306983	7.893764
2.878107	2.52559	0.4135917	4.903437	5.201245	1.782796	0.2789675	4.985622
0.09835605	0.08829178	0.1135975	0.1344823	0.1806222	0.2040071	0.04302735	0.04161478
0.05268362	0.08679615	0.1872233	0.04869926	0.01407256	0.03671632	0.009619721	0.0732071

TCGA-EZ-7264-01	TCGA-HW-7487-01	TCGA-S9-A6U6-01	TCGA-S9-A6U9-01	TCGA-DU-5849-01	TCGA-HW-A5KJ-01	TCGA-FG-7641-01	TCGA-S9-A7IS-01
0.1836871	0.4217707	0.1087456	0.1494016	0.2712785	0.1873128	0.0770295	0.1823791
1.733782	0.8256887	0.436421	2.70909	12.02087	0.4510373	0.4523955	1.437079
0.1854592	0.2810255	0.4140408	1.33854	0.457046	0.7622103	0.1594731	0.7811937
1.073894	0.2784823	0.1490218	1.570415	2.200096	0.05600464	0.07916921	1.630205
1.08629	1.011638	0.7070742	0.8176144	1.01717	0.9256231	1.13944	1.103495
0.7121129	1.017401	2.122051	2.333176	2.523382	1.470606	1.018896	2.557653
0.4556653	0.5007787	1.184643	2.564368	1.041174	0.3402737	0.3978467	2.268289
8.986817	14.79951	44.64438	113.7619	13.08369	0.3518548	8.602636	39.69624
0.7864433	1.380221	8.275181	13.33596	0.5855094	0.1755745	0.7030226	1.74324
2.695975	1.550333	5.320197	13.37222	2.373005	2.080219	1.313336	4.044906
1.579151	2.911294	8.692007	21.67589	5.596612	2.456772	2.139479	8.705227
2.674535	4.223352	10.21603	32.38113	6.212681	7.533456	2.140217	9.628688
0.6172286	0.4935665	0.7412711	0.6752942	0.5666604	1.177094	0.5543723	0.4535271
1.733052	2.488787	6.698225	11.11312	3.180591	0.4591232	1.520948	5.807252
44.63209	146.8187	222.1174	589.1445	130.1632	8.268912	100.3377	309.4061
0.2550534	0.3697554	0.2072489	0.6459847	0.3877546	0.2302542	0.2202057	0.5213706
3.072401	2.293687	4.292696	6.981995	2.274257	1.044797	1.345096	2.749398
1.144258	1.897472	3.461976	14.34839	3.04399	0.3989467	1.549942	4.927037
0.04123209	0.05154501	0.01898559	0.1858456	0.09235538	0.03924288	0.04034508	0.1114436
0	0.01973597	0.02544275	0.1310807	0.03808192	0.3155383	0	0.02133522
1.415555	2.22811	3.795429	10.10551	4.190218	0.4769813	1.122707	3.38638
0.2975239	0.3568723	0.6563581	1.64969	0.7620613	0.4120759	0.1955306	0.9361847
0.08752638	0.09847653	0.1874048	0.5855331	0.2035899	0.01249556	0.03211629	0.07097086
13.0451	7.776832	33.00367	72.95837	23.13476	3.49836	4.210156	13.23247
4.384962	4.206438	5.171906	8.377585	4.527633	5.54337	4.037163	6.316717
2.373799	3.135032	9.37318	23.7994	4.562787	0.6966261	1.972463	8.791197
1.090016	0.9759876	5.355945	5.136193	2.485253	0.2842271	0.5698096	2.133209
0.08992622	0.2312608	0.2070356	0.2645278	0.1879966	0.09129365	0.1437197	0.1180557
0.5095444	0.5941681	0.7949582	1.744902	0.6073289	0.1026978	0.2991496	0.7603618
0.7026124	2.577134	2.333095	0.7692846	2.081294	1.099522	1.5667	3.145948
0.0109238	0.01950864	0.005029935	0.01036566	0.1242227	0.03119037	0	0.07170417
0.3698293	0.5457585	1.738754	5.642638	0.4997093	0.08336537	0.4190111	0.75157
0.03194559	0.06846133	0	0.02273503	0.0165126	0.2508363	0.02344376	0.07400889
0.8226974	1.662022	12.00148	14.30552	1.338049	0.1271763	0.6537411	7.059326
0.571419	1.01353	1.820866	4.688998	1.884072	2.079614	0.6385476	1.757567
0.002515796	0	0	0.003580882	0.002600819	0	0.003692512	0.01457097
1.924958	5.4731	12.26824	26.54842	3.980027	7.053878	4.865264	9.217011
3.038955	8.506854	5.271808	4.15787	9.449331	2.522392	3.423074	9.605508
0.3416192	0.2600919	0.4719024	1.791436	1.505596	7.85465	0.1319487	1.541216
0.1443685	0.03966544	0.5778248	0.4267833	0.3558979	0.2695223	0.04346545	0.3044452
0.004279864	0.01375801	0.005912074	0.1218357	0.03539604	0.07332093	0	0.01983047
0.07806597	0.2275282	0.04744871	0.3955728	0.1355832	0.01783194	0.02749916	0.1265998
0.1106797	0.02964916	0.01911119	0.03692273	0.07330042	0.04690923	0.02792074	0.04206784
1.388396	1.295746	2.381894	4.143618	2.592833	3.165	0.591617	2.568029
0.7323832	0.5083735	4.154936	12.01762	2.052358	0.3024934	0.4867659	3.436134
0.3666121	0.730272	4.501572	9.341484	0.3044973	0.1990663	0.4139137	2.689566
0.0928766	0.05358774	0.09474234	0.1749065	0.0635179	0.07751635	0.06291593	0.07944695
0.0257162	0.04330179	0.07104716	0.04706151	0.06456428	0.005244758	0.02156826	0.04681063

TCGA-P5-A72X-01	TCGA-DB-A64U-01	TCGA-DB-A4XG-01	TCGA-DB-A64R-01	TCGA-CS-4943-01	TCGA-W9-A837-01	TCGA-DU-7014-01	TCGA-QH-A6X9-01
0.03702099	0.03558701	0.1026664	0.04548066	0.3040677	0.1641937	0.27391	0.2446005
0.5109485	0.6688099	0.1105429	0.6677716	3.178714	0.4146542	0.4021694	0.2873086
0.9170859	0.4877814	0.1563578	0.2402653	0.8617739	0.1852021	0.8337206	0.3766767
0.3597393	0.4256063	0.5052085	0.3994488	2.233055	0.08591144	0.6700608	0.1893625
0.8625582	1.423049	0.7565525	2.577688	0.4744983	1.035574	0.5909642	0.4922714
3.658455	2.028659	1.547833	1.335138	2.288653	1.07051	2.052737	1.46712
0.7747233	0.770067	0.5180679	0.3199518	1.600253	0.3509121	0.9002682	0.8339209
32.11574	27.89681	5.708932	11.62673	48.59495	17.18	20.84727	30.18261
0.7944705	1.114822	0.4938254	1.497678	2.43386	1.247434	2.195845	3.167577
4.899268	4.502315	2.422654	4.227622	9.642623	2.205834	5.516167	6.338915
7.443392	5.277076	1.758449	3.459809	8.384426	2.721066	6.036286	5.208527
11.58964	4.713758	2.635995	4.109998	11.90713	2.759797	9.542139	5.147523
1.28344	1.111916	0.2883426	0.3991695	0.3896312	0.4438515	0.4108692	0.7636411
2.813012	4.976812	0.8947414	2.415353	8.322535	2.302942	4.747145	4.41567
17.64834	252.6286	12.07518	43.13902	93.37096	37.77335	30.73229	83.08578
0.2857481	0.2899399	0.3473018	0.331542	0.8822831	0.3004056	0.4057529	0.3995682
1.832422	2.819504	1.728281	1.900717	3.972693	2.491751	3.382642	2.300234
1.166133	3.362892	0.5984467	2.268718	4.356071	1.027033	2.520558	1.664723
0.09695083	0.2423083	0.02688635	0.01191051	0.1751848	0.08599831	0.1825899	0.00915088
0	0.02497842	0	0	0	0.02304941	0.1747787	0
3.826692	3.475765	1.32437	2.544858	6.520345	1.512897	5.79439	2.944541
0.5889011	0.7106242	0.1679467	0.3540389	0.8901903	0.2611865	0.6489367	0.2838357
0.04321897	0.02373995	0.005707361	0.05309494	0.09127906	0.1314395	0.1619601	0.1689996
22.28829	17.60327	3.571731	25.50766	67.60445	3.833217	26.16666	15.85468
5.274582	7.28671	5.356429	8.451611	7.643271	3.234996	6.918147	4.146388
5.938991	6.603938	1.048445	2.74514	13.27583	3.148325	6.330729	6.135562
1.509714	3.11847	0.623141	1.664915	7.151549	1.009043	3.036926	2.240186
0.1578808	0.1788663	0.1250954	0.1142967	0.2361912	0.2275731	0.204801	0.1303913
0.5624089	0.8617472	0.2267188	0.3896193	1.88939	0.4951227	0.913004	0.6146566
0.38125	0.4214549	0.1233494	0.6557172	1.424766	0.6087244	2.551526	0.629737
0.07705682	0.004938139	0	0.006311005	0.07172838	0.004556782	0.04146371	0.0145463
1.780383	0.8271135	0.008461606	0.5622669	1.033758	0.4465755	0.5202566	1.235495
0	0	0.02083086	0	0	0.03997759	0.03031415	0.02126961
1.131096	2.309589	0.4783711	2.661057	9.387277	1.324639	3.510586	3.140015
2.610864	1.555742	0.8750621	0.9491119	1.279118	0.8505233	1.645197	1.608287
0.003549301	0.006823643	0.003280967	0.004360352	0.02623661	0	0	0
8.235468	4.316363	3.507349	4.131253	4.718771	9.24444	7.273637	5.274668
0.5982387	1.277925	0.6451791	4.613804	6.578719	1.827814	7.108799	1.756715
2.295644	0.3779473	0.0234485	0.140232	0.770868	0.1012528	1.057827	0.3232221
0.3237925	0.3714928	0.02413816	0.1796437	0.5361761	0.06485472	0.3618091	0.2070312
0.03019029	0	0.01116314	0.02225345	0.01487787	0.01071188	0	0
0.07489246	0.1312787	0.02036189	0.08659401	0.405256	0.04298533	0.1363057	0.09147937
0.1073515	0.02345302	0.03834098	0.05994652	0.01803516	0.04328363	0.03282104	0.05526852
1.769201	2.247317	1.372606	1.97942	8.381349	0.8126911	4.037475	2.087365
1.969025	1.261838	0.1561856	1.421043	4.248582	1.400726	1.263204	1.398475
0.5570047	0.5396789	0.1389396	1.023713	0.7134657	0.3784025	0.4950734	0.9680272
0.07861853	0.09688885	0.04285951	0.04457707	0.09934222	0.1162283	0.06101547	0.1084542
0.01554882	0.03487527	0.004791099	0.0891421	0.07236812	0.01838969	0.05577803	0.02446005

TCGA-S9-A7R1-01	TCGA-DU-5874-01	TCGA-DB-A4XC-01	TCGA-P5-A5EW-01	TCGA-CS-6669-01	TCGA-DU-A7TC-01	TCGA-06-2569-01	TCGA-DB-A64O-01
0.3936857	0.1642325	0.4090458	0.1099402	0	0.1362986	0.1237732	0.1307789
0.9376942	5.67471	2.608247	1.151463	0.2533373	0.3802298	2.919804	0.8192709
0.3278906	0.5080584	0.6007155	0.449983	0.2315732	0.3892098	1.575766	0.5695081
0.4087085	1.253171	1.004612	0.7464456	0.006448587	0.184657	0.7941074	0.3502848
0.8437797	1.779758	2.235375	0.5321595	0.1570649	0.5760468	0.5767625	0.2173082
1.336384	1.181951	3.171554	2.257945	0.641986	2.09947	6.027681	3.987973
0.3778415	1.013986	2.497732	1.892753	0.3964135	0.804096	4.221397	1.040356
5.56131	8.344139	72.58038	57.60776	7.443825	32.09674	3.872432	69.975
0.685159	0.3578443	9.628549	2.006775	0.6682025	3.593171	0.6411453	3.672126
1.936741	5.193156	8.642271	10.31085	0.7310241	2.908992	3.802789	5.280595
2.429898	3.851953	12.11858	9.420817	1.544203	9.947888	2.654727	10.15715
4.30794	4.126366	14.53992	9.63768	2.335299	4.489999	2.57071	6.931443
0.8753316	0.3219164	0.8975166	1.0807	0.3816182	0.3020531	2.176259	0.7307693
1.185358	1.181561	8.617705	8.423583	1.531916	4.482267	0.9438522	7.556732
50.22815	16.99493	138.7488	165.1791	51.19273	60.47311	31.35286	185.5332
0.1563107	0.2660469	0.8436005	0.502861	0.4143324	0.4286034	0.5838244	0.4922492
1.663233	2.386429	5.290414	4.250842	1.219637	2.137486	1.890092	4.611155
1.034626	0.5246838	8.312455	2.30458	1.934157	1.485849	1.003596	4.656966
0.05727705	0.0788504	0.07651514	0.2687179	0.06326005	0.02677047	0.06482764	0.1141615
0	0	0	0	0	0	0.08687606	0
1.59022	1.554525	4.943739	8.509199	1.081085	2.814288	0.8432485	4.359945
0.4515475	0.541951	0.7416544	0.849615	0.07007703	0.3921089	0.2932386	0.5901641
0.02188551	0.04108455	0.4093092	0.0611172	0.02302058	0.1591174	0.1651372	0.1235929
12.79513	11.02874	18.26435	102.8665	2.198893	12.59692	8.147464	26.77172
2.955883	6.178475	7.19567	5.325683	2.303795	4.480287	20.12029	5.953616
1.746831	2.19033	16.94943	14.95292	1.859345	5.676674	1.622436	13.97992
0.5061102	1.121423	3.746266	7.812851	0.4974502	2.68864	0.5634379	3.241235
0.1032671	0.0854643	0.2225036	0.2232638	0.5781544	0.1998091	0.1225359	0.136111
0.2398286	0.4283323	1.374971	1.850163	0.1852586	0.4709439	0.5042451	0.5626616
5.811104	0.6060494	1.895598	1.075578	0.03553774	0.2631809	1.359619	0.897859
0.03034932	0.04178036	0.05676017	0.04576668	0.02394252	0.009456558	0.02862511	0.01209813
0.3677323	0.2673318	2.362313	1.630998	0.3455642	0.4675952	0.3774443	2.020986
0.1863826	0.01666126	0.1067076	0.0223067	0	0.02074109	0	0.07960454
1.257852	0.7160426	6.499634	6.725281	0.4410296	3.762844	0.7662107	4.387394
0.9489275	0.8895576	3.176358	1.269564	1.423207	1.264777	3.933991	3.257727
0.004193745	0	0.00560233	0	0.003308436	0	0.00395549	0
9.302156	1.080078	9.006536	6.230618	3.1803	15.14203	5.736218	8.185091
13.19473	3.513968	8.446051	3.750541	0.09294011	1.682472	4.629877	3.052565
0.4046215	0.7220653	1.121088	1.393593	0.2837378	0.5136437	0.876345	0.7616659
0.06170698	0.1390072	0.5193278	0.6823955	0.04868051	0.2259208	0.09894218	0.3505244
0.01426877	0.04017904	0.2382666	0.005977016	0	0.01111503	0.03364533	0
0.02602665	0.06188743	0.5632485	0.2093233	0.02874532	0.1135352	0.1080113	0.1815622
0.02882799	0.1118424	0.08087241	0.03622711	0.0795982	0.042667	0.02719022	0.08331469
2.725029	2.686251	4.138977	4.565928	1.501894	2.384439	27.42736	2.976099
1.873517	0.5765667	1.405255	4.078354	0.3513311	0.7476561	2.636133	1.438582
0.475323	0.2484057	7.06844	0.665149	0.2905075	1.293894	0.4754151	2.071761
0.06907445	0.03875193	0.09227502	0.1396836	0.02066964	0.04453022	0.0269587	0.09969604
0.024496	0.02682464	0.09817099	0.04104433	0	0.02385225	0.005776084	0.04882411

TCGA-TM-A84R-01	TCGA-HT-7854-01	TCGA-HT-7688-01	TCGA-DU-6408-01	TCGA-HT-7481-01	TCGA-DU-A7TB-01	TCGA-HT-7681-01	TCGA-HT-7474-01
0.1406907	0.5882675	0.1795622	0.1040694	0.03370924	0.05790616	0.04063161	0
0.7539794	8.196761	1.491466	1.130722	0.4949373	0.3995985	6.204383	0.7157108
0.3264234	0.9092122	0.3424451	0.4853893	0.3657843	0.9301217	0.6352441	0.4268968
0.09859009	1.132266	0.4553832	0.298192	0.2708657	0.1244398	1.237622	0.4024928
0.838552	0.3856844	0.6032238	0.4811865	0.7159899	0.3827864	0.8894413	0.2979076
0.900467	2.776138	1.300302	2.007116	0.828481	1.087148	2.309295	1.775191
0.3727204	1.7287	0.4751283	1.424076	0.8044785	0.745115	0.7200254	0.5930708
11.85314	102.1212	14.99961	80.9892	15.63306	28.45744	36.26139	25.29935
0.5379075	10.92648	0.9206411	3.478345	1.014423	1.38193	1.718853	1.219871
1.309501	3.515476	1.622674	4.270002	1.786049	2.810297	4.359318	2.230899
3.001297	13.57953	3.530695	9.800955	3.401772	6.513744	7.523566	3.048029
2.723725	15.90367	3.658854	8.927397	3.936462	2.767764	6.695304	4.02288
0.4661363	1.930977	1.204485	0.8129118	0.4349075	0.4955186	0.4600281	0.4377752
1.59971	12.42441	2.445142	8.762636	2.676127	3.512867	3.386139	2.971584
58.37916	543.3607	81.97552	70.90174	305.2771	80.02217	101.5995	173.6031
0.3519209	0.622226	0.3299901	0.5107171	0.3517332	0.3559056	0.4065404	0.4010373
1.820472	7.235006	2.101095	4.28823	2.792128	2.25996	3.120495	1.962924
1.880692	7.768085	1.705161	4.331922	2.864257	13.68	3.666517	2.506564
0.08289941	0.2850034	0.2754255	0.09993044	0.0353112	0.05307577	0.117047	0.04453102
0	0.1032258	0	0.04869732	0	0.02032208	0	0
1.414187	7.50473	2.34209	6.909932	2.162908	2.177867	3.859556	2.316957
0.2321328	2.17019	0.3516158	0.4578916	0.496289	0.2792776	0.405679	0.3549001
0.01759766	0.9124018	0.1368974	0.2082727	0.09557102	0.04345758	0.06098666	0.07089695
6.994104	32.96853	15.14054	32.71824	8.897345	7.498848	23.39071	8.904757
2.999587	4.603766	3.268385	5.274292	3.883358	7.436971	4.581585	4.135891
2.862788	21.32655	3.49931	13.68914	2.892676	4.699881	8.07887	3.537809
1.227528	4.502218	1.459641	5.276963	1.291542	0.7303894	2.928571	1.585762
0.3482106	0.4211103	0.2461391	0.3909817	0.1540258	0.1455231	0.1918443	0.2676226
0.4097869	3.292482	0.9258837	2.595347	0.5698534	0.8730742	0.7100811	0.6700864
1.62997	6.527606	2.734129	1.482557	1.284436	0.7155958	0.355668	0.2918567
0.01952258	0.07346647	0.04627346	0.009627275	0.0187103	0.004017598	0.2931833	0.0353934
0.5044048	7.308978	0.7896451	2.148598	0.3625634	1.363752	0.6329222	0.5290548
0	0.01790379	0.0156141	0	0.02051867	0	0.07419686	0
1.116766	13.93806	2.873687	5.971297	1.272244	1.266189	4.66543	1.890228
1.215597	2.712419	0.8675027	1.559438	1.120532	1.416184	1.337233	1.180509
0.01348839	0.002819939	0.007377903	0.009977407	0	0.005551618	0.02337276	0.002717082
4.526918	22.95546	7.669583	8.038418	3.454784	3.672748	5.14343	4.30805
4.799577	20.86056	8.382474	4.204258	3.389385	1.507567	1.896799	1.526557
0.5181457	1.380522	0.7294121	0.4991475	0.7506549	0.267816	4.148187	0.9806348
0.14389	0.6514358	0.2460667	0.5040411	0.1521689	0.191964	0.9858698	0.3198343
0	0.01918908	0.01673502	0.005657843	0.005497918	0.004722196	0.07952333	0.009244581
0.07115337	0.4480185	0.1465208	0.2765779	0.1203404	0.0344537	0.2175793	0.0741945
0.06258589	0.1802748	0.04902551	0.0320064	0.02665861	0.08777277	0.166021	0.03922243
1.650856	4.869537	1.729359	4.677349	2.330103	1.704845	4.022195	2.805479
0.3395695	12.1951	2.228859	6.162298	0.4970371	0.5997045	1.426444	0.70641
0.2456979	10.50864	0.5237852	1.480873	0.3501938	0.8816073	0.6355878	0.5042367
0.03064345	0.08969024	0.05726813	0.1397802	0.0697501	0.06621499	0.08186119	0.04166621
0.0393934	0.1029468	0.09696359	0.03399599	0.03775435	0.004053431	0.03413055	0.03967674

TCGA-DU-7302-01	TCGA-DH-A66F-01	TCGA-HT-A5RC-01	TCGA-P5-A72W-01	TCGA-HW-7493-01	TCGA-HT-A61A-01	TCGA-TM-A7CA-01	TCGA-DU-6406-01
0.3563865	0.078979	0	0	0.9879648	0	0.04281093	0.02861844
1.037018	8.975399	3.063857	0.5839482	6.942791	0.479102	0.1760005	10.45436
0.2567344	0.2762741	0.8574455	0.3592537	0.6517946	0.2970089	0.3789735	4.390508
0.6780835	0.7674525	1.057991	0.5839482	1.878028	0.007622077	0.9680026	0.48131
0.5828255	1.420763	0.2848609	0.6640884	0.2067442	0.5392604	1.419639	0.217093
1.094876	1.853469	2.61594	4.25984	1.726915	1.552433	2.367395	3.419125
0.7760891	0.4254982	1.807807	1.120313	1.049664	0.7373328	1.029317	2.640197
16.10344	5.29074	124.8316	34.29223	75.54686	25.73565	34.21738	54.29543
0.5474313	0.681852	5.456515	0.8358801	3.042023	1.292857	0.9979227	4.030811
1.581885	3.433088	4.368501	8.38066	3.226745	3.06709	6.448818	5.179002
1.804738	3.467148	16.24939	11.2594	5.819283	3.69448	7.378808	13.25227
3.101611	3.351323	7.309075	7.831666	7.58647	3.276417	5.529294	8.301793
0.3554423	0.3639163	2.349666	0.2813874	1.010627	0.4832826	0.6425121	1.740643
2.726505	2.269255	9.141447	3.945795	7.668414	2.899323	4.465526	6.075489
77.99808	87.38341	850.0685	63.76382	53.16178	92.03279	64.24465	280.1617
0.3565832	0.3781795	0.6647479	0.3016421	0.6232969	0.5480324	0.6241606	0.392698
2.811114	1.008722	4.660431	1.281382	4.904665	2.23411	2.380744	5.704438
1.183968	1.376689	2.855959	1.815155	2.108028	2.06666	1.727631	3.24012
0.05090768	0.06204923	0.1694498	0.2656994	0.3925542	0.0106817	0.1681703	0.05995692
0	0.02771756	0	0.02848528	0	0.02862927	0	0.04017439
2.127673	1.839148	4.037887	5.228338	6.854515	2.06364	4.095256	4.71771
0.3015489	0.5546591	0.9033539	0.3708577	0.5361581	0.1449514	0.4709062	0.5666282
0.0810491	0.3622202	0.2427998	0.02707294	0.2386278	0.1836661	0.07853724	0.1431843
5.161128	10.83516	21.94788	37.48539	45.19378	4.167425	22.82276	34.04255
4.609436	5.996011	8.617995	8.375813	3.889988	3.810583	4.496207	8.655789
3.17528	3.569265	19.76475	8.149673	12.30649	4.056578	6.681855	12.55828
1.437979	1.250853	3.881291	2.586392	7.172565	0.9670633	3.24805	2.659806
0.355291	0.07217487	0.2340584	0.1668914	0.3087334	1.003304	0.1825727	0.2746057
0.6143122	0.3157419	2.23571	0.5191795	2.190808	0.4099888	0.6845972	1.588673
1.851754	6.466072	0.8954015	0.08358702	5.61339	0.1680191	0.3747446	0.3094546
0.03147012	0.01643897	0.2020188	0	0.1890929	0.02829949	0.01188109	0.1151637
0.3805116	0.6102494	2.154818	0.5970487	2.013201	0.8118549	0.386361	2.044982
0	0.02403709	0	0.2964343	0.02073689	0.02482773	0	0
1.403939	0.820115	7.461899	1.928785	9.714746	1.56386	2.197088	3.663226
0.7696463	0.6123639	2.868707	1.275387	1.472465	1.285199	0.9498981	3.5078
0.01242463	0	0.03877148	0	0	0.007820988	0.008208795	0
2.925795	5.16618	9.060431	4.900866	10.16783	4.558376	4.94313	6.47239
6.45707	19.81743	3.41271	0.5100691	24.8038	0.4760298	1.076135	2.003985
0.2774895	0.4870372	1.385465	0.500527	2.544356	0.4751095	0.2786674	0.9216196
0.1691054	0.1448379	0.6788778	0.4007488	1.143793	0.143848	0.2717654	1.308031
0	0.04508468	0.01978737	0.0661906	0.03333834	0	0.01396477	0.0560114
0.1002405	0.1409756	0.5534221	0.1303923	0.4418868	0.1067826	0.09169977	0.2894716
0.01067593	0.06506222	0.07462474	0.04546772	0.1167492	0.06720229	0.008464145	0.1848329
1.963035	2.257863	7.074773	4.398371	5.087713	2.610593	2.192032	3.321436
0.881497	1.129873	4.00366	0.9403327	8.724877	0.6586963	1.134729	4.701996
0.2224522	0.4526819	4.517536	0.7608288	1.000734	0.4432191	0.4421921	1.862441
0.04939681	0.04300549	0.08367841	0.02872782	0.2355914	0.07107209	0.1049009	0.1215494
0.0635016	0.00552853	0.03963179	0.01136332	0.2527827	0.04568302	0.02397412	0.02403949

TCGA-HT-A74L-01	TCGA-CS-5396-01	TCGA-DB-5273-01	TCGA-CS-6668-01	TCGA-DU-5871-01	TCGA-DU-7292-01	TCGA-R8-A6YH-01	TCGA-HT-8107-01
0.1502308	0.1013435	0.03071544	0	0.2207994	0.08424496	0	0.02748968
0.4146846	10.02155	2.32706	8.616403	1.852512	0.7009269	0.0818184	0.8475985
0.512648	0.508247	0.8354379	0.209934	0.5156652	0.6314889	0.4052751	0.7673243
0.6513035	1.56238	2.525491	1.153397	1.491272	0.6297098	1.041325	0.04623264
1.247066	0.5024456	0.1531145	0.6828816	0.512735	0.1369421	1.600286	0.4885572
1.343573	0.6789015	3.313179	1.020356	4.602958	2.669617	1.438521	1.210488
1.840806	0.879896	1.602821	0.4776918	1.584199	0.5676481	0.9056145	0.4406288
34.3703	23.42714	140.1577	4.711461	70.45966	40.42675	37.45968	13.37447
8.226637	3.109135	4.064779	0.3228304	8.48858	1.014777	2.568689	2.508892
11.1511	5.14027	5.587042	1.664766	7.293053	2.283334	14.80354	0.9669731
5.920498	4.171372	17.47464	1.949684	10.40605	3.219511	7.201335	2.447507
11.38984	4.627896	11.57173	2.240211	11.00702	3.406753	10.14145	4.23106
0.5247743	0.4558484	0.212968	0.7147268	0.8056073	0.3376555	0.3729239	1.211167
5.13478	2.484036	13.30484	1.342141	8.367159	2.875608	3.334067	2.144932
101.0279	12.37195	212.3492	28.2427	136.7603	89.3585	25.88384	167.8451
0.8073992	0.3223054	0.9834364	0.2494275	0.4283167	0.3251242	0.5027971	0.3221997
2.549109	2.353194	5.587359	2.02442	4.770981	3.229113	1.83764	1.79118
9.339692	2.374303	4.252237	1.16643	3.047901	1.281182	3.099874	2.71481
0.09442209	0.1924144	0.329795	0.008462768	0.2230319	0.02941617	0.1276913	0.03599508
0	0.08891591	0	0.02268205	0.02213978	0	0.04889148	0
3.020163	2.292029	9.385811	2.154678	9.427249	2.112318	5.391282	1.805286
0.7525183	0.5187855	0.8368527	0.3281154	0.7312853	0.204342	0.6365316	0.4047207
0.06514192	0.6380303	0.08708311	0.07545105	0.3840175	0.1404987	0.2729958	0.09169127
16.46341	8.712213	59.34838	8.218793	41.04715	6.370753	66.93137	15.9158
6.843227	7.730003	6.564293	4.373938	5.12966	4.246718	10.64735	2.920668
6.617581	6.514688	18.21397	2.317454	20.57719	3.65744	9.090816	2.319155
2.518948	1.398418	9.682684	0.9623137	3.781159	1.214411	2.378156	3.556003
0.1647459	0.09261268	0.1426854	0.04429703	0.2570258	0.1475587	0.08752596	0.3600736
0.9094676	1.565618	1.803318	0.3912617	1.729392	0.6607579	0.7120907	0.872905
6.544268	1.421983	0.6642595	0.2163137	1.42927	0.2602717	0.251067	1.104069
0.004169276	0.10547	0.1193401	0.01345247	0.02188475	0.06234683	0.02899698	0.003814534
1.894414	0.7611287	1.712573	0.2317142	1.824994	0.4999204	0.4865461	0.7102835
0	0.03084368	0	0.1376915	0.0383999	0	0.02119971	0.0334657
9.443944	1.851727	9.666577	0.4259049	8.53491	1.811277	4.527626	5.599247
2.205713	1.19328	1.940687	0.594407	2.081421	1.663096	1.172088	2.095134
0.00576121	0.009716075	0.06184019	0.009294478	0	0	0	0.002635509
6.22238	4.549112	5.938004	1.429178	11.46915	4.261261	9.656892	8.364705
11.67967	6.027481	2.757469	0.6092313	7.787292	2.26892	1.156872	5.083828
0.2676333	1.354062	1.231177	0.4096271	0.6699918	1.144847	0.3698873	0.4143815
0.1992112	0.1572591	1.273888	0.07293837	0.26253	0.174302	0.2014377	0.5080051
0.1176114	1.69835	0.01001927	0.02108231	0.04115656	0.0137402	0.03976281	0.03586815
0.1001124	0.2894332	0.5190224	0.05768213	0.2177053	0.05346674	0.09117874	0.1112217
0.07128508	0.02838522	0.0870427	0.07027974	0.03949669	0.08698167	0.01377172	0.0507265
3.589699	5.945794	6.762659	1.185819	3.499325	3.091387	2.080506	1.548299
5.701316	3.277661	4.000563	0.2836217	3.931142	0.8971284	5.428793	1.022978
4.610332	0.7820541	1.368062	0.1408452	1.802279	0.5365162	0.5489641	1.244082
0.06871483	0.06622006	0.07860801	0.05806777	0.1099239	0.04893109	0.1308552	0.06885568
0.05889046	0.1064107	0.09890371	0.01357245	0.06623982	0.03145145	0.0146278	0.1462451

TCGA-DU-A5TS-01	TCGA-DU-A6S3-01	TCGA-HT-7610-01	TCGA-DU-A6S8-01	TCGA-P5-A781-01	TCGA-HT-7881-01	TCGA-HT-8109-01	TCGA-FG-8185-01
0	0.08030289	0.1159182	0.08085365	0.03366142	0.05842629	0.03104858	0.02634536
1.550558	0.6484777	0.2859317	1.460178	0.6425057	0.4203447	1.632022	0.6111704
0.411858	0.3038362	0.2648099	0.4069314	0.4205358	0.1710112	0.4122752	0.3611086
1.014127	0.2776128	0.06498447	0.460825	0.3899965	0.01637706	0.1798623	0.6892371
2.045136	1.483739	0.7361245	2.576018	1.010448	0.804107	0.6090069	1.104885
3.50472	1.466513	0.9615218	0.8417837	1.826967	1.23652	1.531478	3.134064
1.040547	0.3646969	0.7927637	1.126794	0.9232385	0.3303792	0.8626351	0.450439
42.48636	9.769877	37.54151	12.97503	22.39249	10.37173	36.44784	20.53589
2.007972	0.1683684	1.867134	0.6132749	0.9424076	0.8394883	2.109958	2.138013
7.443692	2.031129	4.131967	2.99441	4.156594	1.594991	3.420787	5.303832
9.347225	3.258857	3.323349	4.797052	6.004388	2.262874	4.565761	5.195332
9.959499	4.66115	3.354648	3.664414	5.782882	1.997028	4.360306	7.743869
0.4033587	0.3805884	0.6083921	0.496739	0.5731453	0.5948354	0.8692867	0.5063825
6.750877	0.9677512	3.58948	2.361653	3.786184	1.837848	4.659214	2.47897
135.6964	33.95318	71.84782	83.03356	116.3468	243.9115	125.0551	10.84813
0.7547416	0.1262599	0.4838116	0.33515	0.3512343	0.2547122	0.4659858	0.3841013
2.587856	1.261022	2.662008	1.459274	2.077734	0.9028066	3.549478	1.308425
4.987331	1.31425	1.88934	0.8836861	2.094205	2.950508	2.230969	2.640191
0.01280373	0.08411911	0.0485708	0.04234802	0.2468277	0.007650358	0.1057033	0.1103894
0	0	0	0	0	0	0	0.01849173
5.856381	1.943969	3.281926	1.943756	3.338447	1.350758	3.235359	2.837954
0.6370744	0.3533222	0.1765472	0.3078567	0.5582452	0.1137032	0.4361002	0.2541238
0.06523059	0.02008865	0.03093144	0.02696857	0.01684155	0.0633359	0.2640829	0.03954348
19.95445	6.881353	12.41028	12.2582	17.0853	2.551488	15.40095	28.96528
7.761927	6.317172	4.638497	6.895339	5.583828	3.062157	3.406219	8.777983
8.659276	2.200101	4.749234	3.872732	4.853624	1.772164	5.80196	4.077805
2.095798	0.7691847	2.088736	1.288211	3.102964	0.5984255	3.398018	1.254255
0.2904161	0.2170964	0.3072021	0.1693267	0.3076145	0.133482	0.3877741	0.06821441
0.5249444	0.2338966	0.9665563	0.4063542	0.5305961	0.3270068	1.131319	0.4694411
0.1258736	1.302487	0.6326878	0.8326479	0.363985	0.4362225	0.527579	0.162786
0	0.03342906	0.01286807	0.02243889	0.03269658	0.01216106	0.05600889	0.0329017
1.239089	0.2531554	0.9085667	0.5497662	0.6824833	0.4478326	0.9788085	1.006418
0	0	0.02822357	0.04921527	0.04097912	0.01778191	0.05669742	0
3.426888	1.026292	5.411979	1.388538	1.707363	0.7992058	1.822842	2.314816
1.419534	1.215451	0.881562	0.9870325	1.16892	0.980715	1.101236	0.9844017
0.004687354	0	0	0	0.01290884	0.005601484	0.005953421	0.002525799
8.170022	2.79652	5.028363	1.5845	4.519614	5.136973	6.059966	7.465325
1.887363	3.676676	2.788949	3.447841	1.450533	0.7867803	2.843125	0.5203322
0.5192454	0.6327571	0.3177019	0.803296	0.6227363	1.020837	1.010516	0.3068746
0.2827769	0.1019532	0.1733344	0.2281165	0.5365844	0.0782995	0.4555145	0.1523755
0.01594822	0.00654863	0	0.006593544	0	0	0.02531985	0.01289064
0.1512681	0.04777958	0.1103527	0.03848582	0.0801131	0.03476317	0.1145367	0.09091659
0.04833163	0.03175336	0.03208546	0.09324915	0.06433357	0.02310291	0.07570956	0.03993366
4.839852	1.130723	1.464046	2.518451	3.70564	0.9473336	2.649616	1.68619
1.965301	0.521547	1.062143	0.7238196	0.8094939	0.517917	1.111815	1.812803
0.9662093	0.2589019	0.6422587	0.3161922	0.2090143	0.5005764	0.6840351	0.9437686
0.06921793	0.05247165	0.08710502	0.1342801	0.1173072	0.1097589	0.106511	0.04447111
0.06160324	0.01124241	0.05193138	0.005659755	0.0188504	0.0204492	0.07824243	0.03319515

TCGA-E1-A7YW-01	TCGA-HT-7607-01	TCGA-VM-A8C9-01	TCGA-P5-A5F2-01	TCGA-HW-8319-01	TCGA-FG-6690-01	TCGA-DU-6397-01	TCGA-DU-A6S6-01
0.1521394	0.02865143	0.09463081	0.04153226	0.1418899	0.3883513	0.5195657	0
1.061052	1.236787	1.542257	0.4024674	1.224983	4.613423	18.99956	0.2459875
0.6027918	0.3865811	1.155211	0.2757408	0.4740563	0.6225318	0.6741904	0.1704352
0.4051289	0.7816921	1.114063	0.2638767	0.04772662	1.253494	1.279311	0.1017494
1.553907	0.2949659	0.4101988	0.9136576	1.479206	0.4055457	0.9240546	1.838268
1.713787	1.652853	5.378335	1.105812	1.588644	1.175	1.371887	2.065955
1.198991	0.7883799	2.763994	0.5695567	1.298899	1.452462	0.8300841	0.9846606
40.53258	36.07711	323.0011	12.91133	56.67577	71.96765	6.356922	10.88452
6.581426	1.848113	5.572966	1.885011	1.361481	7.323835	1.413524	0.1291435
15.60087	2.332467	7.159757	3.218523	4.574597	7.851947	9.461802	2.950735
13.41642	4.155781	58.53193	4.325813	6.034055	9.849381	5.399773	3.160379
10.82933	6.841408	29.85901	4.992826	5.761259	8.833122	7.814846	2.86324
0.7344041	0.6160883	0.4152724	0.5358379	1.297627	0.4307025	0.4962424	0.5072962
8.297239	3.542594	17.31249	2.149109	5.417749	10.26887	1.1112	1.163513
160.3838	175.7633	118.6926	88.87267	50.34412	99.72809	53.149	14.99377
0.6632591	0.3235303	1.602848	0.4749164	0.5475918	0.7216219	0.275216	0.2933524
3.115378	3.232243	5.765747	1.495699	2.740318	4.458856	1.855751	1.090944
3.453509	2.823118	9.212896	2.118316	1.880022	2.920668	3.124498	0.9671926
0.1892513	0.1575684	0.2849928	0.1196415	0.2229494	0.1201929	0.3761776	0.03290612
0.08008967	0	0	0.02915137	0	0	0.150163	0
6.486641	2.908769	13.05529	1.955159	4.749385	4.991888	3.363932	1.543707
0.431244	0.4751589	1.040908	0.3443881	0.4274035	0.6512159	0.6154673	0.1701099
0.1141781	0.1099012	0.06312783	0.08311801	0.07572337	0.8419696	0.1172324	0.01397045
51.84587	22.5848	81.68927	19.93954	16.93672	20.96408	28.44797	7.166846
13.16051	3.598102	11.71057	5.773621	5.671985	5.511495	5.107128	3.350321
16.35413	6.880298	35.28745	3.950141	8.246334	16.50666	4.183217	1.988623
4.739748	2.434627	7.745	1.323436	4.04231	5.23657	1.194172	1.040714
0.2606859	0.1963731	0.428788	0.7242928	0.2053044	0.2796138	0.1815429	0.08931062
1.086111	0.9065225	0.9295729	0.5408077	2.350023	1.609005	0.4852425	0.2009341
0.7246284	1.372022	5.091909	1.881915	1.052073	1.345227	2.18745	0.0215667
0	0.06361186	0.2626239	0.01152623	0.03150238	0.07838335	0.1102647	0.0232479
1.434159	0.8536486	1.918634	0.8112569	0.908651	1.540716	0.6423271	0.150164
0.02315165	0	0	0.02528051	0	0	0.09301688	0.05098966
8.795291	2.420173	12.46256	1.476269	4.040461	8.354303	0.653033	0.5185653
1.866676	1.498324	12.66058	1.339468	1.282721	1.589992	1.464663	0.7980573
0.003646503	0	0.01814502	0.003981808	0.005441347	0	0.02051089	0.004015566
11.80512	5.288592	36.68889	12.14271	5.749154	10.69594	6.418384	4.259357
1.843867	5.375843	13.93254	7.457092	2.955245	6.370626	9.356209	0.4512188
0.1433351	1.305497	13.68116	0.3414875	0.2333301	1.632841	1.193643	0.2295884
0.5365484	0.4850144	2.162592	0.1054594	0.6925559	0.5777197	0.1250307	0.1713472
0.03722051	0.02803799	0.007717059	0	0.0138852	0.01151626	0.6629679	0
0.1312565	0.3136716	0.2984142	0.05930727	0.2431393	0.4159186	0.243673	0.01495252
0.04010597	0.09629943	0.1496756	0.04379381	0.01870203	0.1163347	0.04229783	0.01656191
2.823854	1.931588	24.67089	1.488593	1.695199	4.549358	4.016552	0.5361475
5.020653	0.6739252	3.189292	1.720514	1.120791	3.079988	3.723159	0.3014369
3.201925	0.7954438	3.805225	1.755614	0.6336704	2.449336	0.1459795	0.1875527
0.186396	0.06240489	0.04122254	0.04296862	0.2626896	0.1364906	0.09319484	0.04105223
0.0266244	0.0240672	0.01987247	0.0348871	0.05562086	0.05931184	0.0727392	0.03518287

TCGA-DU-7294-01	TCGA-TM-A84S-01	TCGA-HW-A5KL-01	TCGA-HT-7874-01	TCGA-HW-7495-01	TCGA-FG-A4MY-01	TCGA-12-1597-01	TCGA-DU-7304-02
0.4836146	0	0	0.4940452	0.1052556	0.173356	0.4583551	0.1615607
2.227842	1.220744	0.6258381	1.083809	0.247267	2.205932	6.251252	0.869778
0.3560376	0.4548688	0.537586	0.3049773	0.2053857	0.3671471	1.519737	0.2306738
1.169193	1.502854	0.411536	0.005430681	0.3737104	0.9070545	2.826523	0.2817793
0.4421962	0.7283796	1.054707	0.601524	0.6767771	2.097801	0.8002568	2.071785
0.6964409	2.201501	1.673246	0.7192118	1.323388	3.777352	1.616752	3.731742
0.4817959	0.9618012	1.192481	0.5745151	0.5280088	1.214396	4.417222	0.4651757
19.43779	29.70203	48.98396	13.71982	15.09055	27.80501	71.60344	7.034333
1.752095	1.063759	2.702995	1.161297	1.39335	1.788841	4.604092	0.1793324
2.660274	4.204465	9.100767	1.21136	2.736075	8.450051	4.504782	8.035821
2.672468	9.466084	9.76162	2.843056	2.896383	8.675786	18.31809	9.090972
2.565157	6.268408	9.439221	4.005299	3.957192	6.660565	14.89966	3.592574
0.1803927	0.3955472	0.3366928	0.5942979	0.5604356	0.5528084	0.3754649	0.1087109
3.543821	5.433373	7.124782	2.022218	2.603812	4.587493	12.12938	0.7993377
156.6242	151.8709	52.99509	151.8811	200.9803	361.8245	1578.034	7.199395
0.432037	0.3776246	0.6549541	0.2824673	0.2958812	0.4625377	0.8589801	0.1462547
2.465633	2.821103	3.590682	2.261119	1.768929	1.805163	4.892299	0.3472925
1.565363	2.39969	4.45159	2.029549	4.125657	3.465494	14.98018	0.6217926
0	0.08139604	0.2511579	0.05327448	0.03675253	0.1210629	0.8402404	0.01410321
0	0	0	0	0	0.04055935	0.03574645	0
2.243228	4.582186	7.069461	1.523886	2.110127	4.898447	4.965612	1.037535
0.3631652	0.7232175	0.595088	0.3491745	0.3028032	0.7431861	1.508218	0.2642889
0.2772493	0.0647945	0.06516293	0.1550944	0.05266167	0.3469352	0.1528834	0
11.50617	26.28224	31.58963	6.832499	8.653428	49.21613	41.61353	26.10642
4.361819	5.377256	5.595804	2.744591	3.299193	8.350604	10.83223	15.84984
3.701756	7.908538	10.22714	3.160488	3.072248	7.085755	15.89373	0.948067
2.023775	3.780378	4.150194	0.9040025	1.018177	2.893541	3.274668	0.194078
0.0874694	0.1272247	0.2164027	0.3341857	0.1068752	0.2860382	0.6709633	0.1230351
0.5558502	1.091685	0.835794	0.6904521	0.4247977	0.7260437	1.506648	0.1414799
2.427928	0.1600411	0.4572475	2.977848	0.9213523	1.725748	3.697519	0.9428123
0.0209711	0.03773806	0.01971566	0.01613056	0	0.04811058	0.03533469	0
0.280257	0.8213375	1.018787	0.3736516	0.303625	0.7929692	1.448115	0.1065247
0	0.02364885	0	0.0176896	0	0	0	0
2.752352	2.769732	3.582043	2.100807	1.527355	2.13476	34.37447	0.3441314
0.700577	1.253016	1.69347	1.129039	0.9289641	1.582435	2.024775	0.6218888
0.002897839	0	0	0.005572403	0.006727421	0.005540033	0	0
3.273887	7.29486	5.352452	5.271925	6.708445	8.738051	12.61049	9.526907
8.818947	1.778828	1.466869	9.496708	2.771791	17.17116	23.27187	4.544607
0.6627311	1.504064	0.4745939	0.3683812	0.1442391	0.8512635	1.099203	0.3689967
0.1108613	0.4603797	0.5862631	0.04509595	0.1534308	0.1793359	0.3951385	0.03798496
0	0.01267328	0.03476003	0.004739876	0	0.01884937	0.09967587	0.03513369
0.08992087	0.1340753	0.1183528	0.1348725	0.0876767	0.1306509	0.7454281	0.05126791
0.01991988	0.1203414	0.04681842	0.03638971	0.02081007	0.1789875	0.1308975	0.04968781
2.373058	5.139047	4.577176	1.636827	1.167695	2.514947	12.25603	7.353187
0.4509817	1.029787	2.163563	0.9947476	0.8252568	2.028655	15.00072	0.397031
0.332053	0.8188319	0.8080063	0.4372487	0.9971051	1.362777	6.631708	1.006397
0.08558448	0.04865751	0.1392593	0.03639635	0.04967174	0.07236985	0.1053793	0.03225667
0.05924279	0.01087847	0.04475588	0.1464699	0.05403119	0.09707935	0.1782492	0

TCGA-HT-7605-01	TCGA-HT-7902-01	TCGA-VM-A8CE-01	TCGA-TM-A84O-01	TCGA-HT-7467-01	TCGA-QH-A6X4-01	TCGA-S9-A6U8-01	TCGA-DU-5854-01
0.02455425	0.5714576	0.03521786	0	0.1219304	0	0.0308351	0.2295694
0.7642997	1.524873	1.127251	3.2049	1.024022	0.4353716	1.385377	3.94849
0.3470758	0.4806821	0.4450089	0.7220607	0.3568851	0.259796	0.6978099	1.940899
0.7249704	0.0510723	3.501153	1.927423	0.3964586	0.3463183	0.4091102	2.610731
0.6066851	0.8750672	1.251811	1.200058	0.6421702	2.72041	0.8186784	0.2594423
2.066068	1.878586	3.690646	2.067447	1.32355	0.7418857	2.547193	3.179328
0.5991118	1.024396	0.7871678	1.028843	0.70793	0.7681515	3.468003	2.569978
20.51581	40.49297	20.88903	38.70178	25.29887	20.89168	136.9196	175.3646
0.8691386	1.078647	0.8947683	1.972246	1.341397	0.8145406	2.821823	5.12071
2.362908	5.704565	7.744768	6.452364	1.953019	2.8239	12.81216	7.577916
3.959327	6.456196	7.537334	8.599276	3.469376	5.693361	17.01934	14.44776
4.694517	5.437206	11.02515	6.736064	4.762992	4.704264	14.17853	11.78704
0.43532	0.8351904	0.862378	0.8070103	0.473004	0.5234558	0.5493789	0.8462406
3.768251	4.50999	4.81968	4.518435	2.908944	3.51016	14.91446	16.78717
173.2614	385.2748	186.7121	70.09335	308.3817	191.8409	384.5807	1214.37
0.2948136	0.3799982	0.3372712	0.6685719	0.2753662	0.5538799	1.154751	0.7687788
2.539707	1.972671	3.306424	4.741044	2.983886	1.919001	6.117874	7.594535
2.455191	6.659315	5.487462	1.617788	4.220687	2.908444	5.402542	6.727611
0.1286057	0.05856015	0.09222876	0.3140837	0.07024868	0.05294573	0.2584038	0.1631823
0	0.01743932	0.02471931	0	0	0	0	0.1841532
2.451677	3.721208	4.283412	3.690308	2.259214	2.574135	10.35222	10.06542
0.6772994	0.2522748	0.9356835	0.7171219	0.2434784	0.3079189	1.085363	1.315318
0.05323513	0.08287327	0.01762027	0.1600148	0.06100446	0.07305465	0.1337048	0.393801
20.04978	4.108098	47.27084	16.76671	7.253683	5.238183	42.59073	26.03166
4.692823	6.240413	6.336612	8.283435	3.682883	6.981777	6.583591	8.383929
5.058074	6.519371	5.842972	7.91539	4.443591	6.401931	28.94429	30.51966
2.221535	2.346895	4.201667	2.282418	1.327492	1.604198	8.790477	6.948279
0.1552022	0.2497602	0.1126433	0.2679147	0.24328	0.2001538	0.5612243	0.2647372
0.7684749	0.9024762	0.9292388	0.6895252	0.551518	0.4041271	1.944182	1.629513
0.1770054	3.697309	0.5258873	0.2470209	1.104985	0.104102	0.1905278	2.093965
0.03747927	0.08963989	0.04398222	0.06656926	0.03383871	0.004675714	0.03850877	0.2366416
0.4310531	0.9798568	0.8098258	1.200999	0.5245743	0.9206281	1.017823	2.063714
0.02989213	0.01512364	0.04287391	0	0.0148437	0.02051051	0.01876919	0.03992512
2.044667	2.381536	5.281565	1.700435	2.201102	1.002587	8.811398	7.347991
0.8911472	1.629271	1.504745	1.579524	1.389921	1.481404	2.044898	3.370576
0.002354081	0.002382051	0	0.003832794	0.007013876	0	0.002956243	0.01886523
4.948485	5.132296	8.300683	7.156955	3.003004	4.36361	15.60512	7.582129
0.7053921	13.82935	2.655805	0.9331425	3.130632	0.06050066	0.9411921	7.30166
1.219755	1.259783	0.3981574	1.835285	0.7686132	0.196247	0.5281939	5.134642
0.3740912	0.3750309	0.5266182	0.5639593	0.2889671	0.1093279	0.4741317	1.535964
0.004004755	0.008104672	0.00574397	0.01304067	0.03181862	0	0.005029151	0.04279128
0.04090683	0.224704	0.06705389	0.1379621	0.1450952	0.03608775	0.6678176	0.3902627
0.0534008	0.08678392	0.03249362	0.1211953	0.03374959	0.019986	0.06096406	0.1210351
2.786887	2.035488	1.953518	3.172815	2.642937	2.24291	5.736447	10.85897
1.168037	0.9943782	2.200764	0.9824473	0.5950006	0.6801968	3.566904	2.34875
0.3327842	0.8233024	1.112318	1.248339	0.3494327	1.368029	2.034314	3.324788
0.02139237	0.162349	0.1093075	0.0631293	0.04249172	0.1412795	0.2619282	0.1696492
0.02062557	0.1078316	0.0049305	0.02798458	0.05803887	0	0.09928903	0.05968805

TCGA-DB-A75P-01	TCGA-CS-6186-01	TCGA-QH-A6XC-01	TCGA-TQ-A7RQ-01	TCGA-HT-7469-01	TCGA-DB-A4XB-01	TCGA-DU-7010-01	TCGA-P5-A735-01
0	0.04564079	0	0.08729118	0.3654243	0	0.2189041	0
1.618234	1.956759	0.6320798	0.2648756	1.74618	2.569654	1.960581	0.251392
0.6038932	1.133874	1.508472	0.2492659	0.7470467	0.3470591	0.6407228	0.5464462
0.3804303	0.5629031	0.160893	0.2446798	1.123621	0.8075232	1.55444	0.04705197
0.418413	0.08408218	0.1749459	3.033375	0.5364051	1.428302	0.6078824	1.04779
3.702288	2.079867	4.181973	0.5363446	2.630836	1.475093	1.657919	2.690213
0.8157965	0.8331779	1.5462	0.3368396	0.8194363	0.894689	1.081876	2.260125
45.1907	48.86794	81.28788	5.37882	40.16064	50.90145	62.83858	54.4296
1.142865	2.195322	2.56182	0.979698	2.650876	2.065241	2.382588	0.8819422
2.433304	2.954125	2.190382	2.818579	5.478723	3.646896	4.855425	7.834554
14.86728	8.944073	11.70788	2.155107	5.76681	6.092903	5.738015	12.8602
10.25933	5.511847	6.242082	3.377855	7.384065	7.97962	7.320368	7.965602
0.5206284	0.6329084	0.6989933	0.6461007	0.7551532	1.090403	0.5067303	0.3580032
3.828533	6.177729	5.793799	1.137157	6.50056	4.898825	5.373019	6.157961
154.3468	217.0046	228.0018	38.47619	189.8236	62.00113	146.7236	84.74172
0.2458376	0.476231	0.4461186	0.237064	0.7360003	0.6640006	0.9112999	0.6046335
5.403139	3.191756	3.890893	2.266325	4.97743	2.456509	3.410019	2.349181
1.499096	2.404611	3.973198	1.076358	4.319729	2.549203	2.748309	2.545902
0.1378813	0.107572	0.03757967	0.02285988	0.05219867	0.1273135	0.08599012	0.211006
0	0.03203514	0	0	0.02331729	0	0	0
3.49879	3.731327	4.417404	1.282021	3.500625	4.651381	4.10277	6.335801
0.46331	0.5638226	0.3521145	0.5022451	0.7589356	0.2468077	0.3658115	0.2386139
0.0819534	0.02283509	0.2632513	0.0291158	0.1662089	0.1081031	0.1825375	0.01679692
19.38701	23.69207	11.57752	9.677905	16.45086	21.67538	27.85206	26.43139
5.522454	6.465534	6.016705	4.506878	22.40104	8.32274	6.081748	4.632887
7.468588	9.833124	10.82673	1.689727	7.607846	10.9708	5.953638	10.98382
7.023784	3.696487	2.354364	0.8223249	2.659042	4.251916	3.139983	4.240942
0.2886872	0.1320778	0.3223774	0.2282335	0.0708363	0.1604302	0.1416987	0.2492745
0.6775632	1.068709	0.4179671	0.2658836	3.043208	1.789283	1.228312	0.3911404
0.3614701	0.8225324	0.2586124	0.194771	1.95003	0.2225104	1.676651	0.1815102
0.1558592	0	0.009956146	0.004037578	0.01843896	0.1723964	0.1139087	0.02096353
1.284453	1.309052	1.166436	0.4532475	0.5872953	1.449119	0.9471911	0.4856033
0	0.02778135	0	0.4427814	0	0	0	0
1.54904	1.357998	3.460162	0.8134614	7.522766	5.123679	3.857699	3.529703
13.05163	1.291271	3.050832	1.012819	2.468878	0.9802148	1.494142	1.212898
0.003365158	0.004375705	0.006878825	0	0.006369855	0.02589365	0.002623364	0
6.516127	4.532579	14.5929	5.702191	6.387079	6.437273	3.304377	8.028395
1.228936	2.376485	0.7085429	1.436699	10.46805	0.5819205	4.249755	0.7233446
7.599861	0.6410841	0.7620073	0.5382956	0.4097181	1.091839	0.8905646	0.1552715
0.7476781	0.7082278	0.2732816	0.04104651	0.3842781	0.9296413	0.7295461	0.2060138
0.04007356	0.007443933	0.01170223	0.004745679	0.02709094	0.008810037	0.06248002	0.008213355
0.09606818	0.1140548	0.1451475	0.01731252	0.1620801	0.04499535	0.25398	0.1558066
0.1272274	0.02105516	0.04019256	0.04218704	0.006568001	0.09967672	0.06852588	0.009956342
4.942374	2.843264	3.55131	1.042892	2.749904	2.397027	5.884444	2.621454
1.478708	1.241782	0.9319911	0.8376325	1.457825	1.355892	1.244011	1.140306
0.6706112	1.697663	2.73521	0.6792601	2.038957	0.6385632	1.091316	0.5171422
0.07836222	0.05716014	0.05078964	0.04277846	0.1628019	0.1205937	0.1370768	0.08500516
0.009828079	0.01916913	0.02008989	0.01222077	0.06046111	0.02268704	0.09960135	0.04935116

TCGA-HT-7479-01	TCGA-FG-8182-01	TCGA-27-1835-01	TCGA-S9-A6TZ-01	TCGA-DB-A4XD-01	TCGA-P5-A737-01	TCGA-DU-8163-01	TCGA-S9-A7QZ-01
0.06054773	0.2564498	0.1064863	0.3059719	0.04319259	0	0.110508	0.0695944
2.791443	0.6965869	1.766742	6.347182	0.3044049	1.021478	2.725864	0.5006931
0.866652	0.787233	3.873203	0.7847908	0.3546007	0.3035153	0.8415015	0.327907
0.8033277	1.37178	0.2885347	1.147615	1.44477	0.2166772	1.101359	0.07152758
0.3411953	1.302703	0.4788986	1.33341	0.9033758	1.390578	0.7943783	2.386986
1.756782	0.984818	6.461067	2.674507	1.068928	1.530433	2.885733	1.03933
1.366805	1.829789	0.4599027	1.249451	0.9077201	1.029099	2.650419	0.4214188
88.14269	78.42971	16.61486	67.28141	31.27437	25.46615	147.901	11.61839
2.057311	4.337101	1.556297	3.606534	3.430644	0.5672608	5.92421	0.6737898
6.626431	11.6101	6.824655	10.10953	10.06481	3.912296	14.17202	2.907689
11.65512	12.45013	7.837731	8.521913	5.705076	7.538429	17.54069	3.473978
11.60524	12.43841	3.573821	9.781838	8.65602	4.327422	13.41145	3.393439
1.998096	1.288571	1.051421	0.9168787	0.310852	0.4274216	1.09598	0.745186
13.3032	12.31416	1.776311	6.166411	4.952342	3.157882	13.07684	1.407311
278.098	223.3286	89.26347	169.9558	128.3527	51.16949	303.0771	29.11484
0.6966832	0.9713815	0.3881283	0.6653875	0.60503	0.2486048	0.8529607	0.2387411
5.200243	5.321157	4.378901	3.644305	2.264507	2.59609	6.750093	2.319783
5.076838	5.142163	1.844233	2.133857	4.54641	1.087484	4.649482	0.9790657
0.7373171	0.09234397	0.04183004	0.1545328	0.02262261	0.2125583	0.3762188	0.03645086
0.02124914	0	0	0.03068011	0	0	0.07756523	0
9.585615	8.383118	1.266608	5.400522	4.131765	3.36057	10.10826	1.375772
1.157825	0.9656035	0.6577379	0.5880537	0.4970323	0.2877892	0.6358271	0.2590976
0.1009779	0.7912294	0.02663871	0.2587856	0.2233054	0.07090492	0.215015	0.09865556
75.998	36.7056	11.5087	23.49429	17.91325	28.02511	44.35382	3.966254
6.02011	7.672938	10.23004	6.04081	5.600034	4.940342	7.842347	5.573924
15.20889	18.33477	3.765512	14.08749	7.320766	7.419865	25.52936	2.169253
10.28997	7.618536	1.343146	5.853238	2.588835	2.734464	10.70383	0.409208
0.3504327	0.2660921	0.04054681	0.3495151	0.05591796	0.1795471	0.39834	0.1059979
2.593472	2.460561	1.070357	1.759929	1.011383	0.5209425	3.311308	0.2384778
1.449715	1.370005	1.973912	2.52077	1.023055	0.1194094	0.6828207	0.322514
0.07561573	0.02224099	0.08126958	0.1122088	0.02397403	0.02681619	0.3475787	0.01931417
1.343625	1.926036	0.6648148	1.67788	0.2883492	0.5733929	1.94909	0.2796233
0.05528271	0.01951249	0	0.02660625	0.02629114	0.07057921	0.08968764	0.06354272
9.835925	5.268318	0.6060357	6.30203	3.812337	1.983573	15.96953	0.715717
1.742984	2.136456	1.857693	1.483622	1.378765	0.9532277	2.591741	1.383444
0.002902436	0.003073316	0	0.03142965	0.004140989	0.01111659	0.00706313	0.0133444
7.520239	11.87657	2.749534	8.554385	6.554277	4.984771	8.369907	5.612063
4.593128	4.2592	4.684288	7.828531	3.295962	0.624571	2.943177	1.280802
1.908375	0.7467913	3.155641	1.108136	0.192367	0.6753105	1.728904	0.453008
0.9779811	0.8094546	0.3905656	0.5672812	0.170606	0.2835214	1.517338	0.09817511
0.009875228	0.01045663	0.06078702	0.04633894	0.02817854	0.02521533	0.06608675	0
0.259383	0.2899126	0.05068677	0.3771052	0.2518526	0.05059285	0.4690264	0.06625298
0.03192237	0.08239184	0.1052669	0.05905335	0.01138614	0.02292477	0.04855228	0.03669201
6.820448	3.88456	4.952576	4.289655	2.58017	2.077959	4.998165	1.692623
5.733897	5.311839	1.813117	2.927107	2.054656	1.0923	2.489394	0.3787011
0.5675547	2.139757	1.071282	1.410553	1.71233	0.5399847	1.570294	0.419668
0.09066566	0.2094623	0.0376894	0.163037	0.09172466	0.07786985	0.3068853	0.07579081
0.06357512	0.0628302	0.007454038	0.06425409	0.03023481	0.02164429	0.09798375	0.009743216

TCGA-S9-A6WI-01	TCGA-S9-A7IY-01	TCGA-DU-A7TI-01	TCGA-TQ-A7RV-02	TCGA-S9-A7QX-01	TCGA-HT-A74H-01	TCGA-QH-A86X-01	TCGA-RY-A845-01
0.08578198	1.101883	0.2280428	0.1606082	0.03879595	0.2612398	0.09699639	0.2016087
0.4450224	2.834017	4.006736	1.792184	0.318989	1.720569	19.51802	0.3552154
0.3102779	0.7215254	0.7949964	0.4958912	0.7284079	1.156281	0.2856354	0.3766086
0.05877654	0.7029254	1.115068	0.4802024	1.645693	1.806249	0.6298642	0.7723244
0.4276178	1.227031	0.7413775	0.7266515	0.6516584	0.2952337	1.74488	1.431042
1.342081	2.581022	4.437029	2.974189	2.377125	1.745271	1.734126	3.372147
0.415043	1.705292	3.289743	1.022597	1.775744	1.458937	0.6003038	1.113094
15.93395	67.06219	92.96808	73.97948	73.06628	105.7118	10.67869	44.82223
0.6171537	3.092872	7.284446	5.293782	5.071924	4.271395	1.040771	4.148324
1.264617	7.723681	6.90594	7.788734	10.6764	4.131822	8.762352	7.566846
3.640049	13.53168	12.7146	9.677592	12.5355	17.48898	4.591119	7.906902
3.468909	9.877539	26.61968	9.206465	13.20187	9.588873	2.875096	7.598176
0.7729584	0.6369442	1.76462	1.02549	0.3609299	0.5732048	0.6363519	0.5249388
2.550383	7.212561	14.30515	6.626737	8.748549	10.108	2.024162	5.852183
153.2349	294.1856	350.2645	109.6931	207.301	439.0246	50.17919	50.49935
0.2288782	0.7060288	0.4672024	0.4476556	1.175612	0.8214924	0.3084801	0.6435828
1.853557	4.550547	7.87826	3.302246	4.168724	4.266423	1.697821	2.912651
3.990598	2.334059	14.63902	6.422818	6.588596	3.777659	1.255831	3.103695
0.01497643	0.2388098	0.6171066	0.2418461	0.07111938	0.2736546	0.1651096	0.03519829
0.02007004	0.02666926	0	0	0.05446153	0.1047791	0.01702037	0
1.853856	6.257218	8.876017	4.708647	6.980411	6.314702	2.648704	4.103703
0.3677518	0.5851205	1.485742	0.5843496	0.8731815	1.149421	0.3529071	0.4833297
0.02384367	0.266143	0.0507088	0.4352607	0.1358733	0.441904	0.08088237	0.2689847
10.54724	22.60393	42.58012	36.1828	26.45765	28.54661	14.35927	16.03046
2.758641	5.676433	3.867916	3.669409	5.35375	7.603566	4.673987	5.443937
3.469208	17.78524	16.89254	12.17265	12.04536	18.76576	4.328077	6.870546
1.583674	4.734901	10.06372	3.122481	4.084016	5.294816	1.301638	2.83613
0.265661	0.3761612	1.950824	0.4127953	0.1743137	0.4348363	0.1403467	0.2021521
0.2906814	1.762041	1.558519	1.064016	1.2851	1.172267	0.3323757	0.7829621
0.3239137	7.39539	1.585192	1.591953	0.2397173	4.631163	1.198667	1.505252
0.007935542	0.1054482	0.04219168	0.1615763	0.01076684	0.2123229	0.0269189	0.09791498
0.9403125	2.89357	5.774743	4.566794	1.031195	2.620628	0.77045	0.6854216
0	0.1156399	0.02313477	0.07332115	0	0.09086601	0.04428096	0.02045306
2.466712	4.673885	30.26774	7.304425	7.049976	5.149666	1.249324	4.153446
1.344373	2.529188	1.369988	1.662577	1.836828	3.115243	0.9760802	1.729469
0.002741382	0.007285549	0	0	0	0.003577965	0.006974477	0.006442922
9.404968	11.5113	18.85227	8.538553	6.750139	12.92736	6.447483	7.981995
1.334849	25.58307	6.209983	8.651134	1.01004	14.00462	3.940289	3.800866
0.4800084	1.405851	1.158866	1.719475	0.2392417	4.065803	1.619974	0.2993019
0.2218526	0.4287998	1.688895	0.6910266	0.4159366	0.9634284	0.1505135	0.1943431
0.00466363	0.06816783	0.03099448	0.07203603	0.02531021	0	0.2412541	0.005480338
0.04083167	0.3481523	0.3527774	0.1720091	0.2769994	0.2442559	0.124081	0.1239541
0.04145765	0.1377233	0.0551055	0.07673846	0.03579494	0.1082184	0.02237334	0.06200452
1.390883	4.182811	4.281343	5.208258	4.535739	7.707219	1.924507	3.125544
1.711551	2.827899	30.54228	6.822503	2.138339	5.325892	0.5618625	1.88742
0.5206968	1.960024	11.61837	2.88632	2.971825	2.24824	0.4169637	1.713271
0.0295829	0.1820675	0.237999	0.1705355	0.1225256	0.03251423	0.06469995	0.08599401
0.0200158	0.122347	0.1489879	0.06745546	0.02715717	0.03657357	0.006789747	0.02822522

TCGA-HT-8111-01	TCGA-TQ-A7RN-01	TCGA-DU-A5TW-01	TCGA-R8-A6MO-01	TCGA-DU-8162-01	TCGA-DU-6394-01	TCGA-KT-A74X-01	TCGA-DU-8166-01
0.1736962	0.1116493	0.08529424	0.05484496	0.2594523	0.1013916	0.3835632	1.343395
3.442907	0.8442374	1.077009	1.111263	1.693075	4.726578	6.780545	5.128358
0.713002	0.2849474	0.3897016	0.6656074	0.5186186	0.4885828	0.4490692	0.7949368
1.374072	0.6572086	0.4462871	1.055627	0.1292894	1.065764	1.662882	0.3124035
0.6682218	1.693893	2.823793	2.03266	0.1999387	0.9998738	1.393848	1.184061
1.976379	2.272404	2.998873	1.193488	1.44163	2.029018	2.011952	4.886309
1.709163	0.8326676	0.8127074	2.263691	0.9729376	1.841886	0.6762898	1.302621
89.22018	17.57496	21.25563	23.69862	43.19216	20.9314	6.804176	68.40505
7.330077	1.631755	4.933703	3.094627	1.301298	0.7034042	0.8862015	3.486757
7.204713	4.507726	15.09329	4.808289	2.147983	4.263474	4.223417	4.023729
13.35191	4.018428	6.935995	10.51929	4.466088	5.100326	6.830007	7.098594
14.43831	6.200377	9.039446	13.75222	4.044499	5.696964	4.807991	8.027633
0.421772	0.4483094	0.5988806	0.4067466	1.285314	0.634041	0.3366414	1.878629
7.478177	2.546597	5.331159	4.56691	4.447687	2.309178	2.023071	8.443394
303.4173	46.16034	161.7579	383.6165	225.5508	66.55054	102.8326	450.4073
0.7944781	0.3351329	0.6949221	0.6310651	0.416177	0.3840514	0.2704699	0.4181758
5.275797	1.741576	2.521648	2.923649	3.812302	2.685484	1.586916	5.210496
12.70146	4.458665	2.868367	8.963715	2.481819	1.045643	1.665389	2.412795
0.1137192	0.05847761	0.03350537	0.02872567	0.1283416	0.0796575	0.1071443	0.1329057
0.04063896	0.01959158	0	0	0	0.01779161	0.03589625	0
7.265094	2.094906	4.822628	3.146934	3.438629	2.760232	3.097236	5.571431
0.6025744	0.3448144	0.5990102	1.127663	0.3853973	0.3260033	0.757269	0.6466476
0.2607121	0.06517072	0.2062605	0.1829342	0.0721165	0.0422737	0.183376	0.2140859
21.84962	11.50707	33.87475	18.22679	13.50772	9.537852	16.98907	61.37213
6.381306	6.493583	9.452781	7.903363	3.068731	8.265068	8.473095	5.426062
15.65764	3.258873	8.651876	7.814023	6.518462	4.456583	3.825414	9.767078
4.425703	0.9159048	2.620854	1.862078	2.980023	1.639473	1.333787	7.83674
0.2270751	0.2210663	0.1883696	0.1253	0.7530128	0.05404968	0.06815643	0.1295864
1.755845	0.5260567	1.003482	0.4667082	1.247978	0.7498842	0.3329684	2.339203
0.6409725	0.445543	2.064189	0.4659641	1.588293	1.78811	14.32539	7.040258
0.1044442	0.01161955	0.01183563	0.03805209	0.04000248	0.2356614	0.04967587	0.1035626
1.929051	0.3795811	0.7381311	0.4441133	1.350735	0.4637879	0.8092848	2.251318
0	0.06796047	0	0	0.035095	0.1080041	0	0.01817153
10.75834	2.842668	4.045229	3.438952	3.356166	2.348658	0.6638177	16.41184
2.60277	0.8863944	1.150291	2.060607	1.666876	1.33602	1.1474	1.282919
0.04440725	0	0	0.005258132	0.002763821	0	0.004903097	0.008586325
6.025909	4.257744	5.104922	10.74804	7.435924	6.02137	4.476185	9.068086
1.767266	1.703959	7.542406	2.240279	5.693663	7.350188	22.31341	27.17585
2.281103	0.1912512	0.1168847	1.888342	0.6222052	0.3386761	1.77836	0.3681898
0.7881766	0.1023756	0.1203225	0.2669211	0.6140718	0.2789097	0.2525055	0.7622492
0.09915342	0.01365736	0.00695567	0.008945116	0.004701802	0.02067099	0.09175244	0.01460703
0.434061	0.09632416	0.1573231	0.2284262	0.2126904	0.1417689	0.08824392	0.2735414
0.09730083	0.02575321	0.04777997	0.03975911	0.05889576	0.09020756	0.06909343	0.02360912
4.940947	1.786481	4.36729	4.095301	2.902941	12.67595	2.902274	2.522131
1.66168	0.8770205	1.796642	2.998854	1.801464	0.5695246	1.140092	7.1739
2.663496	1.0299	1.163633	5.072223	0.6953552	0.3284066	0.5969427	0.9036692
0.2317228	0.06839446	0.1253995	0.07316697	0.1145909	0.1283622	0.03898666	0.09590816
0.04863494	0.01953863	0.06567657	0.03455232	0.04843111	0.04613318	0.01431969	0.2549466

TCGA-DB-A75L-01	TCGA-P5-A780-01	TCGA-VM-A8CB-01	TCGA-WH-A86K-01	TCGA-TQ-A7RV-01	TCGA-DU-6405-01	TCGA-CS-6667-01	TCGA-HT-7472-01
0.1714396	0.03208866	0.7674447	0.09278912	0.06871835	0.05855433	1.037651	0.05856989
0.5437084	1.064783	1.211314	0.3814664	0.4136735	6.035278	1.38424	21.14629
0.6755891	0.9071504	0.335572	0.755152	0.3777425	1.590546	0.9227883	0.8717922
1.454466	0.7255603	0.2688963	0.4508239	0.7447958	1.400572	0.3157872	2.829251
1.021823	0.7267746	2.942018	0.9301235	1.474483	0.1459448	1.076291	0.5109427
1.66785	2.207129	0.9578203	2.826873	2.609599	3.128055	1.305491	1.654411
1.471698	1.551608	0.5339097	0.6568933	0.8597649	2.346401	0.8553794	1.786347
44.3666	49.55242	8.947312	34.1007	38.09869	173.5955	21.57167	90.97572
5.171875	2.991947	1.200891	1.945478	2.292559	3.05839	2.150012	5.222677
11.71551	12.08967	2.914503	8.48669	6.090134	3.337342	7.16516	14.61801
9.72974	9.155181	4.100297	11.51865	7.300541	8.262684	7.566535	10.05432
11.45561	12.34948	4.915105	8.180965	5.444332	6.334425	14.11348	9.39742
0.4483921	1.2251	0.5935766	0.4397657	0.5880413	1.243669	0.3174487	0.5320413
5.887697	6.379604	1.972528	4.390774	4.978214	7.3077	4.113832	9.538825
49.76333	254.6428	120.5797	45.1552	65.74007	388.3588	38.2045	98.44229
0.8919778	0.8393523	0.6581727	0.4310444	0.6777404	0.7114092	0.4958046	0.7660147
3.336855	3.241074	1.799679	1.93891	2.103537	4.835328	2.66672	5.812766
5.430966	4.661765	1.059222	1.88785	2.803935	2.02492	2.266529	3.614311
0.1077521	0.1176476	0.02512237	0.08504887	0.01799601	0.2606822	0.1941005	0.2684207
0	0.04504587	0	0	0	0.04109911	0.1872833	0.02055502
5.692526	6.252038	1.824077	4.935499	3.171645	5.512969	4.067778	7.417995
0.5918446	0.7819528	0.3490294	0.8322223	0.3372385	0.6787596	0.5167575	0.6938074
0.08577499	0.05886709	0.03199745	0.07737405	0.1203345	0.1708934	0.0988875	0.595844
25.81298	59.6045	16.09854	39.2342	10.98459	30.90206	19.7706	48.85481
9.875055	6.988394	7.644179	4.948121	9.322123	8.414061	5.738595	6.407592
7.120657	11.15628	4.525434	7.27654	7.588459	15.18788	5.34803	16.12482
3.563934	4.948234	1.401057	3.009548	2.196244	5.314336	1.664496	9.29284
0.2480606	0.2639177	0.1205408	0.2119881	0.2590425	0.1739069	0.309312	0.2430883
0.8420386	1.334152	0.2739363	0.7366044	0.4474042	1.765694	0.6942077	2.88005
5.579046	0.2974102	4.297423	0.5733371	0.3715299	1.23616	5.633019	0.3920573
0.004757873	0.0267162	0.01996734	0.04506474	0.0238388	0.1421899	0.02468347	0.8086662
1.23353	1.455906	0.2609129	1.330671	0.9514949	1.856787	0.7220463	2.541542
0.02087091	0.01953223	0.08758879	0	0	0	0.0180461	0.1604306
6.128073	8.695434	1.743244	2.603354	2.634715	4.168486	4.410615	6.666672
2.203173	2.067154	0.700278	1.614915	1.181813	1.149492	2.029697	1.384105
0.006574548	0.006152851	0.00459856	0.00444797	0.006588211	0.008420639	0	0.01965338
7.600243	11.09828	4.58559	5.946663	5.259342	7.668072	11.58858	6.165559
13.20542	0.748995	13.34882	1.915926	0.9562211	6.33432	14.4523	1.814043
0.1527082	1.000394	0.6573021	1.017244	0.3413647	0.9628934	0.7312966	1.725643
0.2998885	0.4390868	0.1150279	0.3141488	0.3102057	0.6071184	0.2467529	1.652462
0.02796149	0.01046721	0	0.01513374	0	0.004775054	0.0338478	0.458527
0.1346468	0.2100175	0.09132468	0.07729229	0.09403978	0.2821988	0.1552304	0.7213664
0.01355812	0.03595073	0.02844964	0.03669066	0.02037945	0.09068471	0.0468923	0.1138685
5.179114	4.929072	5.239359	4.909416	2.726884	3.347918	3.036026	8.422003
2.371362	3.492238	0.7324988	1.02612	1.067521	2.2458	1.883876	3.413289
3.214038	2.875693	0.8133091	0.8476151	0.6933773	2.020678	1.118692	0.7728175
0.07468155	0.2026851	0.04701234	0.113682	0.0991587	0.1307239	0.05973063	0.2710851
0.03360216	0.09883308	0.02014542	0.03247619	0.04329256	0.01639521	0.1411205	0.06149839

TCGA-FG-8188-01	TCGA-DU-A7T6-01	TCGA-HT-7877-01	TCGA-CS-5397-01	TCGA-HT-7856-01	TCGA-DB-5275-01	TCGA-TQ-A8XE-01	TCGA-HT-7884-01
0.08969883	0.1568478	2.701617	0	0.1875519	0.3407961	0.3805193	0.160268
0.5268026	5.611459	1.169964	3.307446	1.833203	1.734634	0.49775	0.7247676
0.7075897	0.7054262	0.4636583	1.203909	0.3768097	0.5028027	0.4395786	0.407314
2.422095	1.231018	0.04079577	0.6038184	0.2353194	1.586796	1.49325	0.32345
0.291615	0.8526988	1.721129	0.07670262	0.9029956	2.4067	1.109628	2.400252
3.123115	2.507464	1.11316	2.725957	1.028909	3.591722	1.186728	1.36222
1.449753	1.063835	1.076522	1.86072	0.5225143	1.183559	0.9795843	0.4823855
48.33862	19.04353	13.51959	140.3836	10.53579	23.67049	28.75475	14.40269
2.638491	1.209035	2.366052	6.194142	1.642329	1.908932	4.684539	1.897573
11.15961	6.48412	3.014591	4.281796	3.751534	4.987969	11.56578	7.83457
10.60907	8.099709	3.503405	8.44763	4.853666	6.533855	8.994196	4.486034
9.407539	4.46579	5.525172	7.877281	4.79826	10.92585	7.075293	5.206864
0.5786349	0.6883021	0.926894	1.342355	0.4656066	0.6131674	0.5920375	0.7961481
8.708945	1.890214	2.140426	11.25578	2.477322	6.353898	4.606935	4.168401
194.2039	103.9539	79.77158	602.3372	167.2995	131.9683	135.5467	124.0525
0.8974833	0.2316651	0.5148777	1.033689	0.2680791	0.4871203	0.5883998	0.5268856
3.850088	1.749299	2.381477	6.260342	2.436931	3.134865	2.259793	1.680511
4.167783	2.209492	3.771772	2.573435	2.519876	5.284639	2.644613	3.111634
0.117452	0.1026885	0.08575773	0.1450629	0.04911622	0.1041226	0.0362366	0.1259133
0.06295934	0.0183485	0	0	0	0	0.02428049	0
7.175471	2.132782	2.048127	6.934147	1.746076	4.053622	3.940789	3.076959
1.115682	0.4644971	0.3186428	0.650964	0.3083179	1.047699	0.5327112	0.3146091
0.1944726	0.1395101	0.1774931	0.5748133	0.1027731	0.09472655	0.2711504	0.1176056
42.5961	17.246	3.395274	35.99782	11.86251	28.25985	34.68837	17.17601
5.340164	7.209556	4.199063	7.378495	3.081157	9.265928	6.611372	7.046
12.44828	4.474552	3.152828	15.18328	5.090473	6.713789	8.797367	4.177202
5.920714	1.110666	0.921333	6.502401	1.026556	2.090033	2.959161	1.678008
0.2390826	0.1612521	0.1371594	0.2952869	0.3244245	0.1643691	0.1001061	0.2880395
1.173126	0.3792132	0.3833947	2.801504	0.4559971	0.551461	0.8297663	0.6956374
0.6466162	1.049914	12.36525	1.711339	2.290146	2.705309	1.567467	0.6766943
0.006223412	0.09068575	0.03029356	0.1110262	0.0334609	0.01970404	0.0240008	0.0133435
0.792882	0.475073	0.2361578	2.218017	0.5995393	0.9514774	1.466878	1.200702
0	0.0477363	0.01661073	0	0.01630886	0	0	0.01951089
4.469069	1.743546	2.937224	12.03242	1.47135	6.79407	4.130903	1.766631
1.616621	1.770274	1.262762	2.594188	2.015526	1.344607	1.147044	1.232063
0.004299833	0.005012473	0.002616275	0.002950362	0.005137457	0.005445506	0.003316491	0.02765758
11.03401	4.236816	6.879968	7.77481	7.138094	10.46168	7.493733	3.259635
3.663972	3.989607	34.88611	8.205233	7.913584	10.68271	6.024759	2.53229
0.3841269	1.352328	1.028393	1.054286	0.798584	1.255108	0.6162619	0.2635519
0.2404183	0.2433875	0.1116384	1.541118	0.07559281	0.3365269	0.4343113	0.1175648
0.02194458	0.3624061	0	0.07026803	0.004369912	0.01852775	0.08463004	0.03659521
0.1547732	0.1057663	0.1753571	0.8898727	0.1084035	0.1182831	0.1440765	0.1068012
0.03842442	0.03790157	0.04316254	0.1784721	0.06533296	0.02245959	0.01823817	0.06337316
5.932388	4.439371	1.74659	4.123075	1.349014	3.22334	3.247265	1.559168
3.054563	0.8718493	0.9005482	2.290377	0.9500256	6.545438	2.307428	1.389743
1.405809	0.7007834	1.575525	2.416109	0.8942221	1.348003	1.999261	0.8611913
0.1636228	0.0412798	0.09955788	0.3217318	0.04230909	0.05103165	0.04520719	0.148357
0.04395242	0.0219587	0.2406895	0.08185819	0.04876349	0.08747101	0.03874378	0.02243752

TCGA-WY-A85D-01	TCGA-S9-A7IQ-01	TCGA-HT-A614-01	TCGA-E1-5322-01	TCGA-FG-5965-01	TCGA-HT-7860-01	TCGA-DB-5279-01	TCGA-FG-5965-02
0.1702279	0.02941631	0.04430512	0.1092463	1.741222	0.06558551	4.320781	0.04163053
0.7373163	0.2332294	1.144901	3.584968	5.120979	12.43184	8.456046	0.37897
0.5559737	0.1953007	0.4649473	1.002172	0.7133163	1.470178	0.4464007	0.1307667
1.79728	0.03298193	0.8610409	2.715156	0.645754	1.194948	0.2507512	0.02333833
0.7655624	0.5514882	2.131762	0.6748127	1.355687	0.241651	0.5932919	0.8842395
2.396932	1.445945	3.856516	1.981101	1.741955	3.548446	0.8748646	1.350009
1.436285	0.358872	1.084969	1.712182	1.323928	1.620696	0.6333051	0.2224303
77.42843	16.22751	36.79228	99.13025	48.2368	115.0821	13.84823	6.092884
3.637344	0.4535015	1.710325	9.967173	4.334661	9.941233	1.287557	0.6931484
9.317074	1.431948	3.010731	10.15357	3.690535	4.907646	1.754118	1.000021
15.55658	2.446558	11.06826	17.69583	7.560194	15.01828	3.259039	1.65091
9.722764	1.89358	7.812524	13.07676	9.904203	12.47612	5.208502	1.769375
0.4743566	0.3356311	0.641606	0.4626307	0.8534499	1.749895	0.5841423	0.6028739
11.85675	1.249776	7.577619	11.15353	7.820867	7.229596	1.776078	1.099773
254.9223	43.15912	261.152	703.2612	287.3109	412.8246	15.17142	105.6025
0.9854333	0.3700092	0.6459451	1.042316	0.6049779	0.7359005	0.3414035	0.3332281
2.997483	1.820634	2.998163	7.001643	4.255967	7.635065	1.807101	1.108739
5.304803	1.470676	4.733044	4.922961	3.348185	6.094755	1.022843	1.964662
0.2006073	0.01540713	0.1160265	0.1001332	0.364794	0.2404581	0.105422	0.02180446
0	0	0	0.0191699	0	0	0	0
7.806836	1.3158	5.032385	8.331952	5.268648	6.993532	2.180562	0.6974326
0.9002168	0.1841859	0.5698151	0.9613384	0.8749124	1.009987	0.3360737	0.1479441
0.2626036	0.0196235	0.08127835	0.2915109	0.4601574	0.8094088	0.1253207	0.05554312
45.47995	4.362848	19.62801	98.44663	24.00009	15.65365	15.80335	4.825168
7.05944	2.604471	7.601923	11.69036	7.104397	6.200627	3.539594	3.677424
13.37525	2.506355	10.52792	23.18282	8.827526	20.13056	4.080297	1.015253
3.55166	0.4686791	2.689131	10.21554	2.261043	4.584093	1.30312	0.1895096
0.2398256	0.2822617	0.4048818	0.18303	0.2896825	0.2572221	0.1021879	0.1521761
1.059692	0.2721609	0.7894615	3.038491	1.315715	3.31867	1.033044	0.1046133
2.235129	0.4846967	1.43723	2.179767	12.04167	0.8273814	24.18278	0.278668
0.005905306	0	0.01229577	0.02273891	0.0780606	0.2548225	0.1079956	0.005776752
1.557321	0.2800237	1.70893	1.701738	1.304827	3.86355	0.50099	0.2058675
0.02590424	0.01790558	0	0.01662443	0.03261152	0.03992161	0.3430488	0.02534032
5.74483	1.497919	2.565734	23.64261	12.14301	8.303419	4.351633	0.3657838
2.021917	0.9851093	1.604311	3.183163	2.156556	2.36677	0.9939233	1.064495
0	0	0	0	0.03081888	0.01571964	0	0
12.58881	7.836951	6.5471	7.789744	14.56434	8.840699	7.66154	5.865223
5.272344	1.980631	5.528695	9.439766	50.35835	3.091158	84.73498	1.158584
0.3499128	0.1814006	0.4553582	0.6362583	4.267483	2.550244	4.404026	0.3137709
0.3301874	0.07884406	0.2062505	1.479466	0.3816711	1.272148	0.2309369	0.04110902
0	0.009595498	0.02890435	0.03118127	0.01310724	0.2139376	0.02626255	0.006789866
0.1569905	0.04900692	0.3163343	0.7572575	0.4048421	0.7297273	0.2522927	0.0297238
0.05328825	0.04846581	0.04379781	0.1529934	0.04943176	0.343623	0.03891046	0.06584615
5.157035	0.9288189	4.537078	6.805663	3.154721	6.744287	6.229402	0.8526372
2.599624	0.3201404	1.290989	4.755747	7.777444	4.938849	3.745106	0.3288403
1.758276	0.2458819	1.18771	4.953869	2.947653	4.871228	0.3893614	0.3380345
0.1459901	0.03203543	0.09649974	0.1561522	0.08022612	0.185705	0.02922663	0.03853658
0.03574785	0.0247097	0.0372163	0.2791242	0.4537893	0.1010017	0.3343909	0.005828274

TCGA-S9-A7J2-01	TCGA-E1-A7YO-01	TCGA-HT-A5R7-01	TCGA-HT-8012-01	TCGA-CS-4938-01	TCGA-DU-5853-01	TCGA-HT-7686-01	TCGA-DU-A7TA-01
0.2457778	0.3302991	0	0.08468922	0.03227429	0.3696231	0.875644	0.6360616
0.608314	0.9483719	0.6311603	2.006104	0.5496876	19.05372	8.923486	9.448871
0.2907603	0.4113362	0.3559828	0.3022948	0.5322323	1.146639	1.764214	0.9615796
0.6889226	0.1303032	0.152626	0.8967932	1.441422	1.061182	3.078676	0.8180365
1.723632	2.362398	0.7965929	1.954833	0.2063529	1.285414	0.3655098	2.91934
1.941604	1.42814	1.716823	1.243076	2.902942	2.305492	2.789751	4.24023
0.5815516	0.5523092	0.7387993	0.9426898	1.422632	1.807316	3.57821	1.965771
17.21135	8.61501	30.55074	17.00398	38.93258	106.586	239.5224	103.0451
1.125896	0.9776853	4.299716	1.08628	1.078714	6.241254	16.09142	9.076849
5.21853	2.716562	2.55944	4.547937	5.027425	6.692423	6.519522	29.82592
4.50761	2.219879	5.803967	6.660284	6.987034	11.23061	28.09407	15.44951
4.095339	5.446729	7.958532	8.165741	7.977269	12.08614	23.63781	18.19751
0.7272557	0.8696794	0.6262924	0.4806613	0.2464374	1.256338	0.708751	0.6337794
2.400146	1.714148	4.55415	3.55196	8.635919	10.66591	28.36112	7.234822
41.95441	31.14912	118.273	93.4461	68.99721	133.0752	716.7716	66.24336
0.336249	0.3514643	0.4178197	0.4519251	0.4290237	1.263176	1.960856	1.053557
2.499503	2.747846	2.790167	2.742552	3.492282	5.940797	13.11358	5.010589
1.680607	1.2095	5.690916	1.547425	2.06941	7.017349	8.339711	9.864643
0.03677969	0.09610994	0.0788025	0.06653541	0.4395047	0.08799741	0.1273968	0.9700384
0	0.02575955	0	0	0.06795969	0	0.0227634	0.07878525
2.799896	1.272698	2.455744	4.081388	7.450686	7.942985	14.70762	7.007811
0.2792601	0.4036869	0.4000991	0.6067042	1.196099	0.7164789	1.262287	0.6774862
0.04098934	0.1162912	0.1362136	0.1224076	0.1076503	0.2689899	0.6652689	0.361915
20.28983	8.819845	8.736918	21.53133	60.53436	44.41916	73.9583	66.74481
5.352644	4.825467	3.954749	8.515243	5.833265	6.709303	8.673635	8.567316
4.209067	2.654261	5.107367	6.273456	9.039129	20.59155	41.12724	16.14765
1.917985	0.5916867	1.736705	1.959729	6.586839	6.271474	12.3335	4.42836
0.1390404	0.1732805	0.405932	0.2579771	0.1818786	0.3915173	0.5384104	0.4103041
0.5694891	0.4527319	0.7266958	0.6158752	1.161234	2.890107	5.030556	1.534254
1.084745	2.39994	0.3984215	0.4942169	0.3157491	2.802936	7.748427	6.83928
0	0.02546283	0.04771998	0.003917226	0.008956916	1.268257	0.1260067	0.08826143
0.1953318	0.5989003	1.286085	0.2268485	0.4029896	2.637885	2.309415	4.218527
0.04274396	0.04467814	0	0.06873328	0.01964522	0.02045345	0	0.06832376
3.042945	0.9453973	4.128985	2.249259	5.252598	7.542104	22.44668	10.91592
0.7703647	1.147293	1.755148	1.117682	0.8465527	1.693471	3.247526	1.987496
0	0	0.01236387	0.01082584	0	0.2416142	0.003109271	0.03228402
6.220591	5.753703	10.1774	5.25561	5.213717	11.24798	9.885531	25.95056
4.129237	5.633975	2.817184	2.432944	1.854346	14.8116	26.0871	30.43213
0.1443457	0.4903514	0.4270851	0.4738937	0.9730102	3.844952	2.66657	1.948372
0.1584972	0.06212597	0.2850122	0.1314159	0.4826026	1.810743	3.051523	0.6967088
0.04008593	0.02394275	0.0280445	0.02762532	0.00526388	0.1205698	0.132237	0.6712623
0.02924716	0.05240674	0.0613848	0.1041381	0.1689859	1.123605	1.636329	0.6366891
0.04165089	0.03627967	0.06515887	0.055813	0.01701586	0.09079407	0.228694	0.1898664
1.528114	2.223639	2.824687	1.879068	4.241491	5.849755	12.59262	5.268358
0.9244776	0.7343959	2.34671	0.8423961	2.787292	4.022642	4.668079	9.050239
0.7148406	0.4952033	2.225194	0.5275473	0.6262537	1.15959	4.449631	2.953194
0.139566	0.02198213	0.025748	0.06456073	0.07908271	0.2744542	0.2436998	0.1731735
0.03932445	0.01541396	0.0120364	0.01185649	0.02259201	0.2822576	0.3904723	0.08381047

TCGA-HT-8104-01	TCGA-HT-7677-01	TCGA-06-2565-01	TCGA-28-5209-01	TCGA-DU-7015-01	TCGA-FG-A4MT-01	TCGA-E1-A7Z4-01	TCGA-P5-A5F1-01
0.02675223	0.2469584	0.1920655	0.2178519	0.3523488	0	0.1748208	0
1.531883	6.591219	88.58213	9.020107	1.566793	0.2014769	2.27933	4.423864
2.135179	0.5269459	3.8008	3.138967	0.7258695	0.320602	0.6265118	0.7946616
1.604728	1.005018	2.124749	0.827764	4.312713	0.8858321	1.254472	2.846466
0.1449545	2.280754	1.073992	1.160738	0.3245589	1.162603	0.6517085	0.6620988
4.986016	1.723456	3.619007	5.794191	3.410643	1.181668	2.213951	2.842642
2.001105	0.7501538	1.823212	1.151041	2.680443	0.8498131	1.559881	1.047141
156.5692	17.46007	48.66023	91.78328	152.4573	36.43749	73.93327	47.56536
3.629385	0.7952974	3.600588	2.857007	4.516356	3.615594	3.26437	2.394785
3.752351	3.373569	20.67038	5.750011	6.813962	14.7938	5.980126	8.045934
9.609954	4.293363	14.14257	9.563615	14.28351	10.01914	17.5935	11.84257
8.518446	5.10656	9.246275	7.386314	11.64875	6.623203	7.430209	10.41828
1.305465	1.141537	2.481348	1.47544	0.7201001	0.3448835	0.2853886	0.312189
8.564496	2.992974	6.632662	5.067844	17.09466	4.977235	9.855591	4.695529
603.5333	133.9015	249.3892	479.4106	359.7983	84.06611	130.6349	79.72181
0.5621068	0.2549389	0.4941552	0.332148	1.266278	0.4903493	0.6996691	0.6220754
4.830574	3.180588	6.659471	9.29599	8.007631	2.533571	3.853621	2.565484
3.754551	2.202359	3.496465	4.892247	3.385476	2.538059	3.582309	2.88025
0.1681414	0.07904554	0.4426245	0.1901707	0.3756843	0.06533784	0.2655367	0.3287705
0.05633192	0.01925993	0.05392408	0.07645481	0	0	0	0.05874508
4.759647	2.509945	6.531842	4.330923	10.95825	3.437854	7.380468	4.669094
1.122737	0.7151026	1.027077	0.5468479	0.9625418	0.3799883	0.6271846	0.6585936
0.2766177	0.06864372	0.2178144	0.1756048	0.3609703	0.1560346	0.2040889	0.3280154
17.45065	18.02729	39.43726	45.17482	49.00936	41.52618	41.15886	36.95193
6.528615	6.15467	14.06541	9.917555	6.100964	8.721677	4.931719	5.996291
11.11956	3.801785	10.88139	9.872962	22.75368	8.793981	17.27474	9.312439
4.597229	2.076645	6.040069	3.388311	10.63575	2.697393	3.727069	3.57975
0.2342885	0.1295585	0.4124692	0.160373	0.3756591	0.1330003	0.1996997	0.1211002
1.570636	1.018631	2.79932	1.111469	3.067338	0.6697021	1.026381	1.046802
0.9918002	3.066004	3.461379	4.3561	1.52918	0.1284674	6.895307	0.3016671
0.09651727	0.04188376	0.309157	0.09572724	0.4819429	0.00865512	0.02911026	0.09290944
1.29316	1.370498	2.806613	1.22543	1.250796	0.8173779	1.741983	1.557221
0	0.033405	0.04676377	0.02210092	0.03063902	0.07593317	0.02128253	0.1783062
6.96088	3.572635	4.287962	11.90538	21.40485	3.188607	5.781136	2.908085
5.148074	0.9936098	2.189412	9.853174	1.73341	1.368526	2.261045	1.242627
0.005129614	0	0.003682766	0.01044302	0.01689031	0	0	0
5.823615	2.623308	9.084305	10.77896	5.685317	3.9974	14.43544	7.513784
2.906024	11.89823	19.65659	22.32825	6.959041	0.8959324	12.96364	1.502734
2.40126	1.720324	2.868889	0.9329307	2.336641	0.1923188	0.3473754	2.809973
1.056684	0.2554773	1.414318	0.6402466	1.217772	0.2287714	0.6954554	0.5253941
0.008726482	0.02685232	1.271817	0.4026877	0.01641928	0.02034607	0.005702593	0.1365046
0.222843	0.1469382	0.8365107	0.1252995	0.6049747	0.0445342	0.1123383	0.1344538
0.1710168	0.06148474	0.1316406	0.08614297	0.0464419	0.03699568	0.03917231	0.1158309
6.369498	1.803072	6.851201	5.329417	8.397742	2.661414	4.714344	7.606568
1.760969	1.859213	3.533224	5.946376	2.067527	1.291945	2.853886	2.18165
2.047662	0.5013175	1.821	1.838299	1.487614	2.27165	1.795278	1.481607
0.09468595	0.07321323	0.4350658	0.1265326	0.2932721	0.1086835	0.0837699	0.05924527
0.03745312	0.1037225	0.129068	0.1779124	0.2114093	0.03492926	0.03915986	0.04101042

TCGA-HW-7489-01	TCGA-HT-A4DV-01	TCGA-RY-A843-01	TCGA-QH-A6CW-01	TCGA-E1-5311-01	TCGA-CS-6290-01	TCGA-DU-7301-01	TCGA-WY-A85C-01
0.1818206	0.1439307	0.1238952	0.04042509	0.5932521	0.09976141	0.2479736	2.696754
1.014443	0.6551133	0.8276887	0.6647682	0.3880108	3.115037	38.50232	1.583808
0.4261775	0.364766	0.4223382	0.6060421	0.3118632	1.614294	0.7877671	0.6593779
0.4020561	0.726196	0.3588581	1.639258	0.3678544	3.082191	8.833275	1.014177
0.6173785	1.645538	0.4296416	1.274811	0.5698402	0.5225299	0.4266001	1.658475
1.494723	1.817843	1.887269	2.118168	0.8514504	3.870764	3.608036	1.58793
0.8041532	0.9100025	0.87159	2.325489	0.4754583	3.849598	1.813474	1.494897
24.97638	35.06233	41.24967	55.00322	8.476974	147.8416	63.88968	33.57058
1.255774	1.70414	6.566755	5.85827	1.024349	6.254486	3.712061	1.313769
2.02812	3.534867	5.023355	12.34499	2.755947	6.579127	10.4742	4.578114
5.377347	8.117445	7.857325	14.1535	2.767048	24.12181	9.747606	7.615016
5.26018	6.453876	7.566358	19.02745	5.003547	19.01388	22.29377	9.486509
0.5319274	0.6000366	0.3996155	0.6067052	0.5041116	1.56436	0.2394025	0.7751454
3.829385	5.487832	4.534111	7.139705	2.272984	22.79069	9.653189	4.392912
79.46654	354.2942	72.28633	211.2605	85.32092	683.4155	188.1581	111.4796
0.4548023	0.5966136	0.6773724	0.4969252	0.3314794	1.468728	0.824081	0.5589216
2.347597	2.139646	2.544799	4.100013	2.276497	10.29072	4.890872	3.015176
2.099298	6.316538	3.086669	7.735321	1.163781	7.15336	12.33148	2.675336
0.07142295	0.01884634	0.04866864	0.07410586	0.03530938	0.6618482	0.2597579	0.05296708
0	0	0	0.02837425	0	0.02334074	0.04351297	0.07098163
3.685689	3.387819	3.139353	8.472247	1.865765	11.79934	10.26626	4.947043
0.2820466	0.5114925	0.4664992	1.935993	0.4015741	1.356203	1.563141	0.4620647
0.2173144	0.1320215	0.1962937	0.1415789	0.1169279	0.6544126	0.1395749	0.05621855
20.27483	16.02996	11.89295	52.19505	9.939331	58.98308	108.1884	19.36873
3.780892	6.781824	4.501266	7.199479	5.060454	8.088187	6.283718	7.903979
6.004511	7.859271	5.330045	16.35289	3.074402	32.16756	11.86564	6.378823
1.977235	1.965595	1.938726	4.769243	1.273573	14.84759	5.6441	1.771084
0.2353884	0.180855	0.4717562	0.2370472	0.2998214	0.5495335	0.1841209	0.2156372
0.7718789	0.5260851	0.6049828	0.9004054	0.47434	3.433699	2.01456	1.035753
1.310699	0.4817245	0.5263083	0.270599	3.124143	2.277324	2.13871	14.56281
0.01681991	0	0.01719199	0.03365689	0.01496747	0.1891895	0.1247341	0.08419681
0.6968198	1.245568	1.07984	1.914104	0.2000247	3.831536	2.283902	0.5542666
0	0.04380501	0.01885362	0	0.03282818	0.06072433	0.05660266	0.02051878
3.291929	2.931659	3.426605	7.006975	1.976252	13.01539	21.3795	4.61107
1.399748	2.119151	1.451152	1.937302	0.95421	3.033658	1.805054	1.557075
0	0	0	0.003875661	0	0.02231691	0.08915207	0.006463625
8.265896	8.519066	7.382405	18.62778	5.255999	8.920961	4.917033	11.70348
5.114505	2.196628	1.418138	2.032324	12.68533	9.254585	13.27357	36.46635
0.2803067	1.577907	0.350176	0.8448096	0.3787725	2.130399	1.051305	0.9007907
0.1538939	0.2436475	0.1966229	0.2908361	0.1065128	2.298606	0.4241448	0.0855955
0.004942436	0	0.01515532	0	0.004398107	0.02711818	0.7128264	0.06047742
0.1550606	0.1884028	0.03685832	0.2116629	0.109103	0.8982728	0.4352497	0.2446934
0.03195354	0.0426848	0.04490807	0.04262636	0.01599434	0.1271091	0.04902683	0.04443125
1.758498	3.74623	1.982425	2.518343	1.426755	10.58501	9.813645	3.48079
1.239391	0.8084703	1.859436	7.883619	1.382165	3.72411	19.71262	2.786973
0.5807739	1.392127	2.908931	4.344439	0.7341615	5.511515	0.9475356	0.9016533
0.05115237	0.06661695	0.09782159	0.08144511	0.04845547	0.2897167	0.1670948	0.1468431
0.05515225	0.0201503	0.013009	0.06791416	0.04907812	0.09311065	0.2299955	0.09910571

TCGA-S9-A6WN-01	TCGA-S9-A6U5-01	TCGA-HT-7476-01	TCGA-06-5417-01	TCGA-DU-6395-01	TCGA-HT-8019-01	TCGA-E1-A7Z3-01	TCGA-DB-A4XE-01
0	0.06866439	0.2315802	0.3191814	0.1751325	0	0.5464633	0.04280707
14.27278	61.98214	2.661859	11.62226	3.282807	0.2666737	1.086254	0.4148209
2.415907	0.8235184	0.956513	1.276022	0.3959146	0.2347323	0.6421905	0.3942191
4.264794	3.477262	0.5996691	1.401658	1.259981	0.03511065	0.2984955	0.0479958
0.7342382	1.711438	0.4947483	0.7436661	0.4902854	0.3902571	1.138832	1.048397
2.404084	3.492359	3.032136	1.661531	5.430164	0.7910134	3.493958	2.929752
8.989842	1.470542	0.7512323	1.165339	1.489363	0.2593371	1.68446	1.254129
274.8181	25.68313	36.89544	25.124	39.42251	10.63488	53.50273	52.08827
12.31899	1.482006	1.954427	3.700377	1.32478	0.7145017	3.509835	2.909026
9.666861	2.895707	5.182515	8.104399	7.775514	1.191918	7.136133	4.117322
15.99576	12.30105	7.968649	5.639252	10.88605	2.488495	10.15104	6.446793
24.0782	5.562837	7.144181	5.378029	13.77481	2.110555	9.534276	5.540571
0.4632056	0.3495345	1.129493	0.3221559	0.6045858	0.9152208	0.4537883	0.3418908
7.473248	4.768601	4.338293	5.085255	9.404146	1.5735	5.466511	7.070751
369.382	111.0566	53.64798	489.4187	468.5304	36.36997	199.3475	121.5513
0.8716039	0.5152671	0.3310114	0.707149	0.1794012	0.4252225	0.6248741	0.7648338
8.150328	2.938614	4.713003	2.967226	4.370921	2.011576	4.390097	2.721118
12.3479	2.640094	1.850537	2.9339	3.641701	1.516452	2.996874	3.099858
0.1214027	0.08091845	0.1646116	0.1253812	0.03057587	0.008200769	0.1431082	0.1569448
0.07508897	0	0	0.2240325	0	0	0	0
5.137718	5.918439	4.018201	3.582636	7.672354	1.180386	4.091514	4.173772
0.621563	1.103879	0.4758705	0.5941509	2.909358	0.1854751	0.4054702	0.4708638
0.184362	0.1603201	0.09931256	0.4524649	0.04867923	0.03655758	0.217324	0.1998949
16.96358	23.97927	15.16519	25.64198	138.0661	4.971266	30.71053	21.1747
8.012952	6.624481	5.077112	6.626433	5.340932	2.147944	4.988019	6.334201
28.79353	12.42557	7.102361	6.032562	12.91309	2.405604	10.47878	10.47534
4.423513	2.656867	1.86994	2.572969	5.159877	0.7008759	2.375975	2.817429
0.4209266	0.1516431	0.2947694	0.1944557	0.1533761	0.2146282	0.7074618	0.3585926
3.209669	0.7176375	0.7141964	1.604142	0.7934961	0.168113	0.8978663	0.8165531
1.39549	0.7778324	0.6813891	2.876123	2.735386	0.6933481	2.142815	0.6171707
0.6977059	0.4239975	0.0688599	0.02214519	0.0445533	0.004345331	0.03499779	0
3.090404	1.409144	0.6503037	1.341629	1.266596	0.2748686	1.507063	0.8943714
0.04341219	0.06269357	0.02013741	0	0.01776706	0	0.02558691	0.02605648
28.2063	1.693395	3.032326	5.385848	10.77152	0.8191853	7.01752	4.68442
2.861966	1.41593	2.125744	1.066259	0.984711	1.211418	2.64206	1.115765
0.00341882	0.01645759	0.01585872	0	0.002798402	0.006004487	0.0120902	0
15.49712	8.773842	6.746135	5.240711	6.640021	6.866609	8.616274	5.520842
5.922529	2.835589	2.702705	8.453053	11.97528	2.389593	6.037978	2.190505
1.930263	6.304408	1.416744	0.546746	1.529977	0.1609238	0.5472417	0.19065
1.338105	1.017063	0.3406852	0.3376961	0.6711662	0.03534013	0.391371	0.2355088
0.1454021	0.07839329	0.04316608	0.2342607	0.009521265	0	0.04799157	0
2.075064	0.257385	0.1220411	0.5507401	0.1563036	0.05216993	0.2801217	0.1630071
0.7144343	0.1900588	0.07848978	0.08414045	0.06540358	0.04746638	0.0664869	0.08745495
43.27351	5.888961	3.529023	6.400981	4.558349	0.9085965	4.268798	2.849385
8.681975	1.566879	1.631812	2.465193	7.521457	0.5496822	1.933212	0.6086408
15.25485	0.9265102	0.4839268	1.553122	0.9288655	0.2954054	3.342967	1.198667
0.281554	0.08225579	0.07205691	0.09559003	0.07311146	0.01705153	0.0709564	0.1468481
0.2596049	0	0.01389481	0.0670281	0.02860497	0.01315226	0.06473488	0.07191587

TCGA-P5-A5EZ-01	TCGA-S9-A6WO-01	TCGA-S9-A7J3-01	TCGA-RY-A847-01	TCGA-E1-A7YD-01	TCGA-DU-6407-02	TCGA-TM-A7C5-01	TCGA-VM-A8CA-01
0.1486437	0.7176113	0.4199931	0.04268935	0.1646328	0.3315609	0.1007901	0.4712596
1.254918	2.212635	0.7399878	3.798336	2.683123	2.141989	4.36557	2.151269
0.8568829	0.7172157	0.5220564	0.6399873	2.283021	1.076979	0.3885474	0.896123
1.888826	0.439547	1.07107	0.4227971	0.684515	3.616819	1.139488	0.5203762
0.6040593	1.162173	1.499281	0.9946262	0.2809952	1.890851	0.7154198	0.7428299
1.864682	2.755769	1.931211	1.719502	1.49626	5.941877	0.7138993	3.729141
1.224387	1.838981	0.9284026	1.27729	0.8649662	1.542696	0.5026162	0.8736424
65.36183	86.05683	28.56527	78.74468	17.98679	111.7304	7.43518	32.05695
2.452001	3.545133	2.845906	1.711132	4.081248	4.825316	0.5718157	1.500605
6.997617	11.13701	4.451545	5.286005	5.378314	5.626679	2.608491	4.059878
10.80303	13.12158	6.469243	12.46949	8.243461	19.0512	3.196009	10.55725
9.435558	10.74787	7.306024	10.90975	5.780051	10.40935	4.138201	12.87563
0.7305754	0.8607607	0.8543188	0.4046447	1.177618	0.4365009	0.4732625	0.5000918
7.625862	10.82508	5.487018	7.568674	4.691062	11.00518	1.674429	5.104637
143.2879	121.3984	461.1708	90.14822	197.5563	161.9993	54.08753	88.84466
0.6586424	0.9972325	0.3707866	0.7932398	0.505937	0.9063716	0.3097404	0.600116
3.597778	4.958306	2.915374	4.322509	4.305355	4.286771	1.865521	2.332228
3.461635	4.25449	2.871876	6.019966	3.460282	3.81426	1.112879	2.319575
0.4963181	0.07308333	0.06469887	0.1341542	0.1832352	0.1302441	0.01319749	0.05609718
0.05216628	0.02798274	0.0173407	0	0	0.0872706	0	0
6.468331	9.45069	3.580137	8.417562	2.192673	7.408461	1.874265	3.940308
0.7357643	0.8568148	0.7859908	0.7224138	0.7661531	0.8907246	0.7248917	0.5727447
0.2231091	0.2194113	0.2760554	0.09967263	0.1441465	0.3525103	0.04202291	0.135753
35.17141	32.88215	8.013665	52.586	17.13735	41.09307	9.799617	29.62861
6.30804	5.615195	5.249714	5.621676	7.975086	15.0997	6.790281	6.025423
10.36526	18.41204	9.937453	13.58278	9.620169	20.20199	2.350454	7.509445
4.031722	5.762087	2.549178	5.48981	1.452036	5.148997	1.481588	2.137132
0.1811171	0.4493364	0.1298182	0.1690503	0.1598526	0.2777468	0.09594468	0.6101017
1.426193	1.93079	0.7760297	1.126378	1.118886	1.121952	0.3511317	0.9150853
1.339418	4.885686	2.264357	0.6594359	2.076894	2.176733	0.830366	9.044471
0.7992634	0.01106416	0.01371276	0.04738941	0.02855606	0.06901227	0.03496468	0.03566897
1.966283	1.764475	1.313341	1.55161	1.322956	1.183589	0.3862738	1.811379
0.1130984	0.02426705	0.01503812	0.05196965	0	0	0.03067524	0.07823282
3.48774	9.266795	2.772613	9.198157	2.3424	9.600439	1.338441	4.825101
1.673591	1.624478	2.339404	2.260605	3.354158	1.805001	0.9477477	2.109658
0.01425087	0.007644368	0.00236858	0.004092742	0	0.007946906	0.01449452	0
4.271074	10.10391	5.849135	15.25079	10.25668	12.0404	3.042871	19.072
4.437027	17.25114	7.651846	4.943827	4.951254	6.994958	2.624036	25.03816
11.1906	0.5463296	0.482443	0.7020027	2.058673	1.221096	1.346667	1.658532
1.761379	0.2811991	0.2788112	0.7587823	0.3657835	1.08746	0.135073	0.2538306
0.1636438	0	0.004029419	0.006962558	0.03356416	0.02027887	0.008219342	0.006987419
0.1636173	0.4981351	0.2234333	0.3606775	0.122444	0.3846883	0.05996929	0.1172564
0.1224514	0.01839171	0.05698604	0.07877435	0.1735976	0.09559795	0.0232484	0.09034928
5.391088	3.606316	2.066143	4.298755	2.845002	11.24712	1.763245	5.37435
2.465692	3.268092	1.756341	2.203066	3.149949	2.044279	1.910742	3.301358
0.974005	0.6783782	2.786348	0.9557869	5.393879	2.655114	0.4242454	1.62936
0.1133148	0.2453056	0.05784608	0.1603913	0.1165392	0.0812434	0.109764	0.02799375
0.06763287	0.106047	0.0518815	0.07171811	0.04609719	0.04641852	0.03527653	0.0359871

TCGA-DH-A7UU-01	TCGA-HT-8108-01	TCGA-HT-7687-01	TCGA-HT-8564-01	TCGA-HW-8320-01	TCGA-HT-8113-01	TCGA-FG-8189-01	TCGA-DH-A7US-01
0.03877485	1.74446	0.2322116	0	0.280894	0.6838982	0.1545954	0.03634292
0.5009957	2.620417	9.809493	1.645016	2.456213	0.9935664	0.2610332	3.553818
0.5148676	1.26691	0.7838742	1.129182	0.5882154	0.2826591	0.2703929	0.3917683
1.086871	1.485739	1.822509	0.363311	0.6007223	0.02017881	0.03611131	1.05266
0.8824119	0.694373	1.258218	0.9963137	1.437443	0.9283606	0.4649021	2.225206
2.011846	1.929561	1.460753	3.352026	3.121538	0.9399405	1.226932	2.28885
1.367339	1.675912	0.8448559	1.426123	1.397973	0.3846359	0.3957903	0.7022799
55.1166	119.8313	22.42872	64.7422	54.8898	8.794242	13.6256	17.59139
1.975062	10.69958	2.876217	4.019452	2.659858	1.167546	0.4242356	0.5737336
11.66462	7.612095	5.084662	5.594166	10.17996	1.155201	1.818978	7.938841
10.09953	10.00617	5.809787	7.545749	8.365236	2.382723	2.284093	6.869645
8.389351	9.685443	6.059453	7.301885	10.33727	3.208207	1.60012	5.763663
0.6874357	1.389729	0.5357156	1.018036	0.5314131	0.8340121	0.4986371	0.4337997
5.596871	13.85084	3.722223	7.93344	6.914755	1.298892	1.210469	3.855197
101.4826	62.14273	151.7655	97.64379	70.21261	92.64773	33.07933	153.2087
0.6429093	1.256321	0.5168374	0.6829668	0.6870088	0.3447103	0.4861395	0.394798
3.091556	7.083053	3.500632	4.697206	3.230788	1.959482	1.485728	1.65276
2.403638	3.786874	4.183427	2.884927	4.635639	2.437073	1.447028	3.287662
0.1116982	0.1142101	0.1042487	0.1035559	0.2125087	0.0188526	0	0.1808327
0.02721595	0	0.02328409	0	0.02190652	0	0	0
6.054194	6.300188	3.334475	4.863582	6.875686	1.58593	1.191365	4.018408
0.7021023	0.7266646	0.7410137	0.373621	0.6179485	0.1400981	0.09156534	0.5535142
0.1616659	0.889576	0.3208797	0.3242428	0.09889672	0.07803861	0.01289125	0.07273264
28.6797	21.77256	15.78054	21.75768	60.27912	5.911901	4.288507	21.45084
5.327162	6.527659	4.800622	7.572268	6.35246	2.337064	1.832882	5.680977
10.70018	16.99145	7.149535	12.58926	11.61922	1.794023	1.888023	4.949449
2.853579	7.222067	2.246271	4.050169	5.173917	0.4505986	0.9603201	2.254113
0.3336735	0.324455	0.2046275	0.5972555	0.2020282	0.3974651	0.3943979	0.1992716
1.284401	2.268463	0.7957152	1.313614	2.003497	0.3248011	0.2648746	0.5230495
0.7986232	6.21859	1.656874	0.2884501	1.687412	4.559369	3.184113	0.318127
0.01076098	0.09310208	0.2439684	0.1920486	0.05197003	0.02996822	0.005363008	0.02017212
0.7382214	4.309123	1.25494	2.240621	2.365245	0.5028426	0.4634727	0.8806262
0	0.0204201	0.1211539	0	0.03799533	0	0	0.04424355
5.133617	4.883655	3.126711	3.44153	9.629157	2.256978	1.034192	0.8200835
1.53199	1.718381	1.680058	2.693604	1.202228	0.9471062	1.048824	1.058192
0	0	0.06678824	0.009477768	0.005984457	0.006901805	0	0
9.673018	5.961301	5.095359	4.921869	8.339731	5.910041	6.4789	5.726611
2.15822	23.28043	8.60672	0.976243	8.994126	17.96663	11.1377	1.663964
0.3719514	0.9194446	2.76166	2.890067	0.5667008	1.899051	0.1986122	0.7470489
0.2133251	0.7903167	0.4586053	1.08776	0.5855698	0.04062141	0.06542519	0.2255795
0.01897233	0.03830054	0.102799	0.07524321	0.03563258	0.01761199	0.006303561	0
0.03691316	0.6107875	0.3434363	0.2980195	0.2228396	0.154199	0.03219406	0.06487124
0.03066469	0.09727864	0.1136832	0.6102441	0.03496682	0.05218765	0.06367719	0.08861939
2.878793	8.788951	4.472847	3.205797	3.009674	1.443699	1.088408	3.070408
3.328278	1.360286	2.131216	1.613819	5.423701	1.377384	0.2713671	1.282261
1.3844	1.876754	0.9625701	0.688602	0.9559332	0.4491531	0.2515205	0.3927431
0.1393498	0.2100718	0.07767238	0.152518	0.244723	0.03527952	0.016836	0.1088416
0.04342784	0.1596852	0.06501925	0.009226735	0.113606	0.161256	0.01082168	0

TCGA-S9-A7R2-01	TCGA-HT-A74J-01	TCGA-26-5133-01	TCGA-DU-6404-01	TCGA-E1-5319-01	TCGA-TM-A84M-01	TCGA-TQ-A7RK-01	TCGA-TM-A84T-01
0.08446977	0.2891034	0	0.1279065	0.2128709	0	0.07915122	0
9.314133	0.7428352	2.759697	3.43297	7.626186	10.4726	0.2905352	0.2398151
1.510572	0.3637599	2.380052	1.07017	0.5166873	0.1671326	0.36446	0.6005028
1.578476	0.1553201	2.097869	1.343277	2.738115	0.5760245	1.442111	1.091159
0.5492302	2.290978	0.4055295	1.527482	0.3152687	1.780169	2.487468	1.181486
3.424018	2.448639	3.389424	2.920921	0.9385727	1.385198	2.641043	2.467117
2.557472	1.071613	1.071655	2.683482	0.6476639	0.8200116	0.8880939	1.778095
160.0159	38.15818	30.22186	38.89413	19.35766	1.152816	26.14707	50.57306
10.18354	2.085881	1.688785	3.126632	1.890294	0.6352311	1.029889	2.457625
5.952925	5.38352	8.342537	4.779563	3.90191	7.168084	7.762105	17.51787
25.11012	9.133885	9.540712	7.760409	6.04931	1.481122	8.262355	12.77166
14.51734	8.156827	5.89587	12.49461	6.368323	2.672736	6.134808	9.699674
0.6746418	0.8119584	0.5747635	0.6690668	0.2304238	0.6986152	0.5210141	0.530405
19.06532	6.038055	3.468598	4.343886	2.623338	0.5791527	4.677746	7.478713
490.2257	108.0663	135.5349	183.0207	369.4813	8.185775	48.64042	126.254
1.334152	0.6198492	0.8144873	0.4424352	0.3549807	0.189626	0.7466948	0.9980517
7.081503	2.242702	2.26865	5.144047	1.827386	0.9574916	1.690394	3.525852
7.248369	2.657424	1.997311	4.456229	2.764036	1.246568	2.604111	3.181222
0.154847	0.05678295	0.1113631	0.0937895	0.09291132	0.2145854	0.04145635	0.203176
0.02964454	0	0	0.01795544	0.02490225	0	0	0.02866083
8.985978	3.553789	3.234328	3.235638	2.989671	1.287757	3.706207	8.229458
1.636716	0.3180043	0.6404635	0.6363652	0.6904455	0.2311063	0.4219244	0.7601065
1.01429	0.1566985	0.1489311	0.05546185	0.3905147	0.006507372	0.3036084	0.1974884
29.3017	10.88825	34.29115	14.11532	20.13184	10.1381	20.44582	45.05108
8.18371	7.634886	8.343667	5.784672	6.123546	4.622131	13.017	8.701698
29.16808	10.47099	5.930639	8.716454	7.230012	1.019962	7.871919	15.00877
9.701157	2.289379	2.13743	1.203324	1.608315	0.8437042	2.582225	5.3828
0.5789445	0.4017994	0.1036289	0.1909158	0.06214213	0.1366873	0.1054845	0.158591
1.56786	0.9081099	1.267742	1.002234	0.6119199	0.1426198	0.510809	1.142704
0.8916347	1.656094	0.4160287	1.567478	1.004754	0.1004567	0.1630232	0.3153831
0.4805703	0.005014585	0.1003133	0.1064917	0.09353852	0	0.005491607	0.08499206
3.216383	2.113198	0.5677765	1.119545	0.3772066	0.7476955	0.4745862	1.357949
0.05141638	0.065991	0	0.03114246	0.107978	0.1187538	0.192716	0
7.885745	1.883491	3.515589	8.255128	0.9564421	0.3350469	2.924079	5.968789
4.236186	2.095505	1.543337	2.160691	1.998738	0.4151807	1.260042	1.714365
0.01619668	0.01385856	0.02038463	0.007357643	0.03401417	0	0.003794219	0.003914805
16.39707	7.771059	11.18274	9.714775	5.693754	3.079318	6.026378	6.077326
4.512033	3.568699	2.061511	8.451318	9.141153	0.4553807	0.5684622	0.6598448
11.53208	0.5571264	1.252894	2.278631	1.470715	0.1069413	0.2847245	0.6994607
1.459703	0.2548944	0.3539297	0.5340864	0.2051994	0.03302598	0.2065649	0.5702662
0.01377687	0.04715226	0.1040348	0.04172266	0.06365127	0.362745	0.01290943	0.0133197
0.2462683	0.1591131	0.1315686	0.2252659	0.2575349	0.09750743	0.04709434	0.1943643
0.205973	0.0523954	0.07006242	0.1129548	0.04910115	0.02314339	0.0286898	0.03229266
9.551207	2.066232	14.84234	1.72461	5.722266	3.063418	3.512384	4.739094
8.592416	1.573169	3.0455	5.06066	1.961717	1.739689	1.333796	2.171793
4.334684	1.818867	1.061266	2.845416	0.8282322	0.1584151	0.318986	0.8971672
0.1908806	0.09248544	0.09493668	0.05571793	0.03477362	0.08498643	0.1292978	0.2290155
0.0354773	0.03541517	0.02381365	0.1181856	0.01490097	0.02731337	0.01662176	0.04001671

TCGA-CS-6666-01	TCGA-DU-7019-01	TCGA-TQ-A7RR-01	TCGA-E1-A7YV-01	TCGA-HT-7477-01	TCGA-HT-8011-01	TCGA-HW-8322-01	TCGA-HT-7879-01
0.6599738	0.8056897	0	1.383283	1.376373	0.03075911	0.5170683	0.1208383
6.247259	3.608019	0.9673704	0.7543753	2.315801	4.93171	36.78463	1.117754
1.089188	0.7970288	0.5986314	0.7420465	0.7090519	1.537108	0.4759845	0.785016
2.314222	2.715424	0.7415174	0.1624808	0.9453872	2.350897	2.741067	1.558082
0.9087269	0.280643	1.294111	1.16925	1.55605	0.1866653	1.548301	1.430632
6.606083	2.760051	2.498198	1.113007	3.263386	4.425602	2.113376	1.763363
1.064778	1.317054	1.520108	0.8376268	1.026897	1.824226	0.831226	1.490339
38.52928	52.86642	86.32284	10.82136	53.20784	29.00619	13.48584	53.35129
1.206587	2.746826	6.69926	2.208114	1.968213	1.604703	1.741976	3.97548
6.848876	4.604751	9.710342	3.973476	4.915579	4.80946	7.320063	11.69208
6.289925	6.202755	15.50964	4.305524	8.518176	8.728385	5.04751	12.56267
11.8117	10.99886	10.97781	5.153481	8.601049	7.704299	4.933545	11.10275
0.456576	2.068351	1.455503	1.276501	0.6268535	0.2888604	0.7619308	0.9173843
5.24948	6.763007	8.441673	1.73834	5.511279	5.910034	2.697832	8.531839
187.391	129.4015	313.0275	122.5532	104.1252	190.9583	96.59514	119.5183
0.754672	0.5963749	0.7598168	0.6327068	0.6274193	0.316554	0.4706751	0.9413317
3.947176	3.672695	4.009956	1.913202	4.423975	3.225285	2.4228	3.289107
1.584065	4.077093	5.925261	1.767704	3.28173	5.316819	2.480154	5.983771
0.1321675	0.1657815	0.1078388	0.1138516	0.1461265	0.08860738	0.05701482	0.1265809
0.02724904	0.02019683	0.05255117	0.08322189	0.0522201	0.02158973	0.01910151	0
4.116914	5.65455	6.058238	1.73474	5.06035	4.586211	4.873739	5.460801
1.136555	1.31961	0.5067986	0.3344101	0.8813101	0.9213262	0.3131624	0.9508763
0.1424389	0.2543396	0.1685664	0.3031999	0.1302812	0.2770102	0.1316196	0.2821376
32.17993	39.46021	35.52345	8.434358	37.94585	17.24268	18.86565	27.08213
7.45129	4.855191	5.455492	5.293108	7.639544	7.788182	5.998805	8.362296
9.041565	9.1028	13.04075	2.777067	10.77284	7.231717	4.739068	13.2588
3.622855	4.393531	4.736013	0.9670316	4.565061	1.633578	2.838998	4.824639
0.07095486	0.2257045	0.5730179	0.2106852	0.1331451	0.1288337	0.1388552	0.2139541
1.121891	1.53161	1.706099	0.3972629	1.980034	1.071584	0.8765887	1.366445
3.498224	3.555927	0.3855145	14.9169	8.351271	0.3009256	3.307029	0.544433
0.1292888	0.1317636	0.01558375	0.02193687	0.01032372	0.02134104	0.07552592	0
0.5087441	1.33756	3.683899	0.752453	0.8737831	0.8061676	1.147267	1.774001
0	0	0	0.1683996	0	0.01872294	0.1159558	0.01838844
4.008004	8.998318	8.955618	3.10956	4.241605	1.861128	4.896422	3.420483
1.99817	2.031672	2.21704	1.248567	2.00363	2.42804	0.9382978	2.337317
0.007443935	0.005517401	0.01076701	0.007578229	0.01069918	0.008846873	0.02869999	0.005792545
2.38521	9.502484	11.2125	10.34096	7.516571	4.015888	3.344053	10.91239
14.1849	11.46956	1.680362	31.82653	24.01138	0.7731891	11.50719	2.006921
2.513724	2.050459	0.2564997	1.96331	1.197954	1.433147	1.062863	0.3829343
0.5366992	0.462745	0.5492112	0.1393831	0.3515897	0.3427898	0.3839029	0.3963279
0.03799079	0.02815855	0.01831679	0.05156822	0.0182014	0.03511729	0.3195774	0.02956277
0.1894099	0.3389894	0.2806471	0.09876505	0.2434659	0.1866745	0.3367974	0.2839964
0.05628694	0.06826837	0.02467097	0.01823258	0.05393419	0.09324784	0.06815302	0.0358364
5.0026	11.00092	3.322411	2.158559	4.69826	7.034765	2.577854	4.150607
2.609979	4.712507	3.311845	2.122877	2.024215	1.031259	0.8885212	2.34914
0.4566192	0.8452499	3.424112	3.013312	1.103827	0.4811553	0.6304291	2.066995
0.06130389	0.1049777	0.2283018	0.08177843	0.05671587	0.05192156	0.1348491	0.1398221
0.04348063	0.09265432	0.03144548	0.2268584	0.1145737	0.0344502	0.1828789	0.05921077

TCGA-HT-7676-01	TCGA-FG-A87N-01	TCGA-TQ-A7RW-01	TCGA-FG-A6IZ-01	TCGA-S9-A6WH-01	TCGA-DU-6392-01	TCGA-28-5220-01	TCGA-FG-A6J1-01
1.021431	0.09091418	0	0	0.6225312	0.9200964	0.2151183	0.9787275
2.482295	2.020075	2.225716	0.8092068	1.870254	2.859479	15.83032	0.6997665
1.174249	0.9627264	1.237253	0.3114822	0.3794663	1.165908	3.968281	0.4982075
0.4475652	1.77252	2.634093	1.730803	1.38703	3.376656	2.765683	0.6759108
0.6717775	0.5911324	1.712863	1.841538	2.00312	0.3379034	0.3543422	2.951317
1.929688	2.529241	1.885227	2.978734	0.948084	2.695817	5.07407	3.544254
3.001605	4.865604	2.177113	0.9099925	0.6993464	3.38888	3.371481	1.114069
134.5931	80.40435	133.7025	26.27221	12.46529	119.9076	125.0043	49.23334
8.93564	5.774566	11.14351	1.51524	0.8209431	5.692841	7.391609	2.0993
8.4728	10.59471	14.73754	4.286052	4.840207	8.915304	2.10907	9.278813
22.17971	8.468515	16.82291	12.50245	5.460603	15.0454	30.33905	18.43782
14.57264	9.512079	14.90053	8.412441	4.749003	13.60477	4.964995	10.55893
0.7048169	0.4122614	1.829371	0.1209286	0.4623182	0.9959656	0.366277	0.2091474
18.05302	5.905263	13.54965	3.674297	2.451855	10.32613	17.10136	4.994942
236.3893	660.8111	210.2101	97.44509	52.51792	530.85	641.2508	68.63516
1.3442	1.017934	1.327799	0.4431211	0.3901523	0.5187531	1.100784	0.3831917
7.955764	3.129418	6.750427	1.695671	1.524023	5.972073	11.13772	2.541052
5.456482	5.713867	4.678173	5.169835	1.433253	4.357665	3.424203	3.453554
0.2490452	0.09523467	0.1210314	0.05011518	0.06270342	0.1405574	0.3718132	0.06686343
0.04944408	0	0.03243905	0	0.03361175	0.1076355	0	0
14.91689	3.685847	6.248252	3.231593	2.511033	8.345127	7.993357	5.090055
0.8881035	0.5333474	0.758635	0.4404242	0.4538081	0.7958204	1.084826	0.7849227
0.2290888	0.06570252	2.790176	0.07659588	0.207644	0.370833	0.7390484	0.04258077
92.92692	39.58588	26.36448	29.40197	14.77017	71.22193	28.2655	48.22267
9.534645	10.89289	6.697561	9.464858	5.565393	15.61129	11.52405	8.886347
27.06264	12.74858	21.94648	6.19239	5.357428	19.45263	18.21722	7.439433
11.31035	3.558026	7.039119	0.5880157	1.208029	6.006344	5.997949	0.9362676
0.3272381	0.1523167	0.4293858	0.1428187	0.2953901	0.2102073	0.2752199	0.2365647
2.039724	1.208059	3.441872	0.2754158	0.3172475	2.209933	2.702854	0.3839843
7.145603	1.778881	0.5711336	0.5715123	5.917807	6.158975	3.898987	12.68657
0.02443727	0.02523094	0.2950016	0.06904162	0.04651442	0.1028491	0.8835698	0.07085769
1.180038	1.517334	6.056424	0.2649718	0.3433693	1.203848	3.244539	0.6891615
0.04287865	0	0.08439489	0.06989036	0	0.09334311	0.05237663	0.07770615
23.89276	14.29993	7.309365	4.409927	1.874276	12.27455	17.35476	6.73006
2.472354	1.917237	2.325384	1.145968	0.8176327	2.725834	2.168297	2.039296
0	0.002905395	0.070894	0.0293549	0	0.02205302	0.008249586	0.00815941
8.562774	6.725356	13.67127	6.44635	3.931338	6.903859	7.975654	9.387282
21.31203	4.108102	1.410677	2.130303	21.19428	23.38078	10.38995	28.346
1.448005	0.6021661	1.504168	1.088308	0.2132746	2.477965	2.741563	2.361712
0.9738545	0.5087266	1.147452	0.1079824	0.1148402	0.998706	1.068187	0.2881392
0	0.01482794	0.03768894	0.04993845	0.007810288	0.1042126	0.1964783	0.02776152
0.5071511	0.3678338	0.8304486	0.1229704	0.09687412	0.7147263	0.6706868	0.0860843
0.04178213	0.065907	0.2741223	0.2043093	0.06311826	0.09937795	0.7910771	0.08693664
9.918955	5.482609	14.59276	4.997218	2.574539	7.044893	17.54997	7.008617
1.323077	3.989626	3.342354	1.444424	1.109564	3.719169	1.570838	4.474261
1.970429	8.793401	5.695274	1.903492	0.689041	2.797071	2.522488	1.417681
0.1821993	0.1353121	0.4932454	0.02709265	0.1069089	0.1294273	0.1686756	0.02317108
0.2465522	0.1230372	0.04529192	0.01071652	0.1072669	0.1502824	0.1626294	0.06553219

TCGA-14-1034-02	TCGA-HT-7880-01	TCGA-HW-7486-01	TCGA-FG-A60J-01	TCGA-DB-5280-01	TCGA-FG-A60L-01	TCGA-DU-A6S2-01	TCGA-FG-A4MW-01
0.1798738	0.4838238	0.05929405	0	0	0.211094	0.7473765	0.06039332
4.366459	0.8449738	0.5049406	7.073722	2.694556	0.5578912	0.482829	1.489702
0.9587979	0.3163118	0.5714489	0.512889	0.5813494	0.8197987	0.5308784	1.793555
2.655409	0.009517002	1.08586	2.198081	3.076567	1.072954	0.06284757	1.038277
2.306624	0.8664931	0.4015993	1.110595	0.6906393	1.29935	1.514548	0.2552439
2.99837	1.360345	2.013888	2.470436	1.928704	6.297793	2.276925	4.555984
0.9236838	0.9025003	1.441466	1.59628	1.111391	2.067756	0.4958227	1.25307
31.43114	29.35091	47.75324	60.70842	45.26902	92.52715	10.16431	77.22516
1.878282	0.9987141	1.791669	3.865727	1.157186	9.200742	0.723585	2.636772
4.696277	2.725817	3.214154	6.399093	12.95525	20.21902	1.981776	4.621058
15.50597	5.088406	6.990092	9.710994	6.574175	33.64764	3.182929	9.505336
4.189476	4.563809	7.246328	8.762917	8.347942	20.89956	5.525446	6.587362
2.494339	0.4201671	0.4319364	0.6552803	0.2667248	0.7559039	1.649711	0.7897792
2.996414	3.249095	7.727025	6.344681	2.603514	13.11355	1.964201	4.745194
426.8529	138.1685	502.9486	172.9014	37.77108	333.6464	227.0805	349.6878
0.4970684	0.513209	0.6441192	0.7248777	0.5609044	0.8267382	0.2830915	0.4834132
2.470596	2.060159	3.714521	3.188427	2.651462	3.984922	1.602395	2.291275
2.352385	3.239838	5.380489	6.896796	2.391265	7.652611	2.127977	4.793094
0.2512291	0.08002364	0.2173915	0.1291196	0.2704377	0.04422515	0.02935852	0.01581584
0	0	0	0.02471918	0.02070947	0	0	0
2.069208	3.348833	6.238225	4.36009	4.656256	9.873737	1.54005	3.359056
0.3043927	0.2930279	0.9181183	0.5780945	0.5492307	0.8930634	0.6576718	0.6029861
0.1799896	0.04671481	0.09888705	0.3700237	0.07381001	0.2886809	0.08725007	0.1208644
23.84532	7.826215	26.88724	51.08165	36.5072	104.291	5.922067	13.75091
18.68374	4.058236	5.756672	3.490854	4.810278	8.756328	5.11878	6.773022
8.211209	6.10721	11.09819	12.0509	8.715492	19.29856	3.163997	9.057878
2.843116	1.60837	5.353356	4.148206	4.454683	3.211133	1.977524	2.531566
0.4520379	0.416932	0.2167432	0.3298822	0.152791	0.6140905	0.4581719	0.3081462
1.225892	0.4770127	1.273273	1.230934	1.19977	0.8149764	0.2561021	0.5518631
2.469837	3.12063	0.5342948	0.1813395	0.1975017	2.543452	5.618548	1.306081
0.1331185	0.04240203	0.02468335	0.043982	0.06960114	0.03515029	0	0.04190161
0.8820826	1.31276	0.4654795	3.935907	0.5763264	2.416588	0.5035605	0.7018309
0	0.01550008	0.09023008	0.02143685	0.0538787	0.07709519	0	0
3.184843	1.332277	6.151152	6.371603	2.816329	11.62599	3.52964	3.931572
1.196829	1.724435	1.291175	1.490212	1.068391	3.736592	0.9524409	1.798317
0.01149665	0.004882682	0.01136936	0.05064619	0.02262979	0	0	0
9.749354	6.912233	4.027721	9.783564	2.539452	17.31882	10.49002	8.876521
13.9412	6.172361	3.220479	1.264662	1.086009	10.4609	14.02296	5.042266
0.739481	1.596477	0.954744	1.628818	0.5862753	2.184041	1.369844	0.9724443
0.5328613	0.2263087	0.6189705	0.630945	0.357949	0.3632978	0.2108611	0.3578205
0.1564645	0.008306401	0.03384764	0.05743941	0.1588031	0.03442906	0.02437916	0
0.2996656	0.1606019	0.2152048	0.356222	0.1263977	0.165791	0.04002147	0.1149873
0.06717423	0.06209299	0.06252278	0.1903188	0.009722387	0.1112943	0.01477638	0.06368191
8.084343	1.347306	3.617914	3.215774	2.996289	11.38503	1.785824	4.329216
4.220379	0.7330574	1.451931	4.685872	1.921455	5.773031	2.472927	1.049505
1.886546	0.4804309	0.6814784	2.674595	0.4580145	8.429122	1.066467	3.869001
0.08488524	0.03605116	0.106546	0.1073893	0.1590535	0.02988556	0.0447656	0.0394623
0.1846704	0.04278021	0.07886109	0.02465237	0.0289149	0.06501695	0.09940108	0.008455064

TCGA-32-1970-01	TCGA-HT-7873-01	TCGA-DU-8168-01	TCGA-HT-7475-01	TCGA-14-1825-01	TCGA-FG-6691-01	TCGA-TM-A84Q-01	TCGA-12-3653-01
0.403855	1.075299	0.04555521	3.80676	0.4162939	0.09625382	0.06357345	0.1238403
4.245606	1.605126	27.68437	3.268241	3.259867	0.8479503	3.397647	13.2917
4.327516	0.6247797	0.3642408	0.3277734	1.733602	0.9345217	1.11873	5.755399
1.403702	0.1116332	0.5107706	0.07059339	3.033899	1.522885	1.479046	2.649745
0.7308762	1.174993	2.522672	1.417094	1.263165	0.2016632	0.8473888	1.570181
2.47452	1.458021	1.115735	0.9869799	3.410455	2.929689	2.023605	3.170475
1.442117	1.912434	0.7951057	0.8151273	1.050336	2.971398	2.052175	1.135873
87.90641	40.56478	3.123962	12.87106	50.51802	165.4359	131.2497	56.16913
4.537581	3.768625	0.471952	1.27231	2.875203	15.82445	7.919132	7.193883
7.110877	14.72125	5.558407	1.447264	5.451426	16.94946	17.43902	6.231349
16.94389	9.697302	2.681705	2.425827	17.34372	19.2784	18.53536	21.11556
8.537856	8.114155	3.850432	3.449407	5.783235	18.78226	12.79292	10.77388
0.9818289	0.8179221	0.6177278	0.3570613	1.924272	0.402683	1.277738	0.4619356
7.589162	8.712354	0.908793	1.954791	7.615055	23.45737	10.33207	7.495886
134.27	270.303	23.77044	49.5422	701.1791	344.4336	268.0808	860.833
0.554164	0.9179242	0.387433	0.3239816	0.6347027	0.8988648	1.249453	0.7169
5.912307	4.257713	2.222409	2.43374	4.462724	11.93349	5.244475	7.955132
3.060314	3.937566	0.9370612	1.449566	3.842107	8.020388	5.879271	6.240013
0.1374904	0.1720889	0.06561517	0.0152201	0.1090193	0.05041403	0.4328651	0.1783726
0.08503939	0	0.03197507	0.02039659	0.09739848	0.02252009	0.04462201	0.08692314
3.84971	6.690344	2.339154	1.961912	4.788905	12.91638	11.19357	4.595428
0.7381008	0.606561	0.2659645	0.1426095	1.056715	1.531131	0.5648088	0.7439723
0.4512618	0.05977731	0.05318197	0.3343964	0.277708	3.157018	0.2756623	0.4337201
26.73195	17.98272	8.80218	6.64042	28.01265	51.21656	103.3941	41.13921
9.198088	6.894167	6.114977	5.530161	8.670799	7.052814	7.692537	11.19146
16.11527	14.1498	1.697694	2.664172	11.06173	41.25492	16.50428	19.67326
2.589109	5.450168	0.8813458	0.9755859	3.790088	14.29512	6.945544	6.048494
0.2337396	0.3298283	0.1977453	0.1128619	0.2007823	0.3396289	0.3413171	0.1744724
2.071211	2.023177	0.437089	0.325284	1.394806	3.67945	3.122459	2.404714
3.389572	6.598054	1.208028	22.99799	4.001277	1.073846	4.451911	1.179683
0.1176838	0.02901326	0.01896405	0.0564526	0.03851062	0.01780855	0.1235024	0.3007266
2.701087	1.562007	0.6589306	0.3987704	1.183705	2.947143	1.984508	2.56444
0	0	0.06932315	0	0	0.07811904	0.03869688	0.03769053
6.185583	7.479699	0.3274918	3.23801	9.975647	20.68829	20.15979	13.31703
4.197263	1.650713	0.7083038	0.949182	2.781347	3.62037	2.343977	1.670128
0.007743732	0.008590982	0	0.002785986	0.01330373	0.003076037	0.03047478	0.00593645
7.604244	3.599143	1.550251	4.340344	5.40203	10.61256	8.392702	23.40005
12.32703	20.51361	3.496698	65.2196	17.56515	7.489003	14.18261	10.45066
2.089202	0.2660591	0.319941	1.174746	1.426192	0.9562996	1.491916	2.227405
0.9229275	0.2486027	0.02891863	0.2172637	0.5089548	0.420927	1.69498	0.7599383
0.07245485	0.0194866	0.2154693	0.00947902	0.02263227	0.01569883	0.1192402	0.1009907
0.2691254	0.4976178	0.1843139	0.2074801	0.3467681	0.446708	0.5484725	0.4273671
0.1756615	0.01377945	0.04053023	0.0172359	0.08230543	0.03806067	0.2199593	0.2407638
4.618186	2.19213	1.691097	1.562305	7.223533	7.529576	9.955266	4.385823
2.856874	1.431364	0.4118185	0.6937191	2.313998	4.798405	2.906993	6.369277
4.27026	2.337974	0.1332737	0.6315326	2.684318	4.836914	4.645874	4.76905
0.1165504	0.2797483	0.1041837	0.06171078	0.1511197	0.3371835	0.2042396	0.1180082
0.0961175	0.2049042	0.02551092	0.2034146	0.1165623	0.06737768	0.3426609	0.1993829

TCGA-41-5651-01	TCGA-HT-7691-01	TCGA-E1-A7YI-01	TCGA-RY-A83Z-01	TCGA-DB-5281-01	TCGA-VM-A8CH-01	TCGA-TM-A84F-01	TCGA-VV-A86M-01
1.003574	0.2840356	0.1697803	2.969107	0.3968946	0.08399052	0.1724683	0.1033336
2.694402	6.038687	2.044101	0.6036097	1.689952	14.1694	4.583414	2.882679
0.7062084	2.633992	0.7078361	1.076116	0.5578254	0.9683566	2.360898	0.7229364
1.857505	0.9129306	1.472116	0.6657998	1.311832	2.001138	1.538931	3.253703
1.113449	0.3201165	0.8426645	1.628585	0.6209678	1.060372	1.401756	1.438953
1.427379	1.880943	2.868756	2.377844	3.75958	3.304245	6.601047	1.746014
1.608617	1.315246	3.165882	1.517232	1.113311	1.4996	2.898858	1.660923
32.26331	82.14253	33.70215	68.52403	31.14853	47.94505	116.1225	68.34501
1.668003	11.17491	3.124184	3.740771	1.87235	8.535677	7.99793	5.926181
4.297258	7.332899	7.492207	8.281972	7.84827	21.76998	11.4542	11.86255
7.956644	13.9592	7.188692	16.162	6.629512	11.90386	29.98577	13.45744
5.922489	15.81477	11.0178	12.22029	8.011296	13.95554	15.58562	10.3614
0.9814687	1.715109	0.5573006	0.248562	0.2416622	0.460724	2.985773	0.3627706
4.574826	10.30377	4.831955	8.154634	4.41827	7.38271	17.13262	8.105004
413.4514	397.4994	401.3848	147.1376	83.41356	315.8435	316.3802	273.4802
0.5464641	0.6292831	0.8251024	0.992425	0.6949489	0.8763846	1.497605	1.307153
3.065836	4.729108	2.303522	2.280074	3.608653	3.615626	7.727751	4.738225
3.503496	8.128631	8.366404	5.25868	2.816816	6.877717	6.853609	7.668198
0.08760554	0.7661495	0.1600639	0.05981168	0.13642	0.1869617	1.095278	0.08118321
0	0.0797455	0.02383366	0	0	0.02947635	0.09079124	0.03626477
2.730076	6.295394	2.747608	4.430792	4.188959	5.90976	10.81338	7.089012
0.768281	1.307399	0.8734286	0.6183993	0.4449667	0.6253878	1.503083	0.6382647
0.15143	0.2131639	0.2378455	0.11427	0.2564928	0.1891004	0.8269427	0.1378668
21.80157	54.58493	22.39224	57.20914	33.83374	36.82878	99.04929	32.69505
8.676346	7.503942	6.925116	10.25487	5.377084	5.897818	7.749113	7.203508
7.273117	14.72603	5.599988	11.06663	6.756508	15.32382	29.25158	16.16357
1.586267	2.952306	1.558622	2.105362	3.754622	4.173877	14.22188	3.439749
0.1492131	0.2682178	0.2249727	0.2191524	0.2418011	0.2174715	0.7059613	0.302967
0.7205373	1.245823	1.074359	0.5269706	1.02569	1.261562	2.191602	1.333744
5.512008	3.597822	1.398747	13.7359	2.61843	1.383923	2.442158	0.2660378
0.05305078	0.06306154	0.0942365	0.01267694	0.07572682	0.01165473	0.9393355	0.05735526
0.6676121	2.861845	0.7682175	0.9995431	0.4109375	2.502437	4.920018	1.628799
0	0.03457825	0	0.0556087	0	0	0.1312259	0
3.893588	24.05475	7.580869	7.926694	4.676135	7.841004	13.16487	9.058699
1.371764	2.436555	1.926974	2.019517	1.677796	2.258489	4.321114	1.585348
0.0229084	0.002723125	0.009766376	0	0.02378209	0	0.2728274	0.03962747
6.859465	22.58106	13.74047	7.645229	7.344428	8.730334	24.54984	13.54589
17.50428	12.26512	3.962914	33.42136	11.46876	7.917235	7.19973	1.855348
1.817318	1.595859	0.7794175	0.9858959	1.291745	0.5323288	13.58986	1.221346
0.4247151	0.6851663	0.3113564	0.5541637	0.3114385	0.5331751	4.202952	0.4373105
0.1558868	0.4493594	0.04984357	0.007450095	0.3317557	0.5137015	0.3375513	0.1601085
0.10805	0.3244779	0.400031	0.2826552	0.1269302	0.7895843	0.7029282	0.2028928
0.04724203	0.0823631	0.05818324	0.03010369	0.03106105	0.07472592	0.3040467	0.1770607
4.73074	8.241244	6.693764	7.36742	3.641034	6.164102	14.86523	5.687774
1.64417	11.93098	4.202106	1.747952	1.0189	3.464645	4.790861	3.292143
1.572142	7.037706	6.055679	3.880857	0.9774834	4.322627	3.925811	2.736184
0.111895	0.07269138	0.1516155	0.06964386	0.08644651	0.1669304	0.1878242	0.1800544
0.06690492	0.111342	0.07130774	0.03837	0.1458588	0.07643138	0.1328006	0.0144667

TCGA-QH-A65X-01	TCGA-WY-A859-01	TCGA-DU-7304-01	TCGA-DB-A64X-01	TCGA-FG-8181-01	TCGA-DH-A669-01	TCGA-QH-A6CU-01	TCGA-28-5207-01
0	0.02996022	2.571347	0	0.09527222	0	0.04268098	0.3519473
6.520785	0.1319676	6.101377	9.315324	1.650629	6.826	0.3759991	18.78379
0.9437556	0.2309943	0.8228837	0.3779361	0.5871882	1.174479	0.4966546	2.996184
0.847679	0.09517666	4.47366	0.3583245	0.1068204	3.1457	0.6300833	3.715888
1.23598	1.516225	0.8679856	1.721111	0.3269418	0.4848878	0.6475365	0.352794
5.957829	1.227234	1.906796	4.233214	1.837451	3.701977	2.600601	2.387733
0.9117686	1.056503	2.242393	0.8240733	0.477928	1.29981	1.193424	1.617962
0.6314842	21.95226	74.7863	15.91209	16.76639	43.56566	41.92114	75.36816
0.7139879	1.034626	2.795872	0.8328708	2.052375	2.453034	3.30052	4.563143
12.27152	2.541616	8.174029	9.1976	1.740119	5.385858	9.972741	4.631757
13.5059	4.64611	9.235169	5.795426	3.48902	9.476532	15.38458	16.67905
9.584941	3.556356	12.78125	5.813927	4.55158	8.124617	7.009518	14.03259
0.1971694	0.3181712	0.6459089	0.3292723	4.055446	0.3160822	0.4195493	1.065681
0.9118566	2.52129	9.55055	2.849542	2.136295	5.008119	7.573002	11.81963
9.690056	60.50625	137.7623	9.852189	53.34842	142.3561	410.3216	520.3869
0.2902318	0.5652759	0.713953	0.3396242	0.2723566	0.4874691	0.3050324	0.911795
1.128886	1.999831	5.170944	1.476769	2.782062	3.852505	2.60944	8.93121
1.762791	2.246803	3.412498	2.102754	1.993549	3.076715	3.980627	3.5507
0.04525526	0	0.6037254	0.07641589	0.1081165	0.4501317	0.06706395	0.1612944
0.1819409	0	0	0	0.02229043	0	0	0.1235154
2.388415	2.273702	7.268716	4.155176	1.4684	3.999391	5.334129	6.529977
0.8188231	0.2129417	1.170371	0.3950359	0.2257152	0.8449151	0.4622541	1.727198
0.007204999	0.01498976	0.3943299	0.02085603	0.1218151	0.07735222	0.37014	0.8730978
26.70971	12.15384	34.56488	17.92924	8.425782	39.75292	64.87264	27.99637
8.371139	6.073602	8.000829	6.906349	1.926208	13.02208	5.594512	11.42563
3.140989	3.81078	14.66115	3.443689	3.221333	8.649706	11.52114	22.33261
0.5572147	0.6478253	8.481807	1.043672	1.216759	5.749336	2.080538	4.848103
0.148051	0.3148598	0.360126	0.2380875	0.2587748	0.1101196	0.1787682	0.2646719
0.2023215	0.2635046	2.616348	0.333298	0.4352901	1.081378	0.8092728	3.145679
0.4893956	0.6016465	13.04229	2.232273	1.17736	0.8288565	0.5494222	2.786279
0.02997419	0	0.1189354	0.05784341	0.01762693	0.09086122	0.02369006	0.1770343
0.4593247	0.4444695	1.187519	0.6235672	1.044341	0.8331213	1.350797	1.849193
0.05259395	0	0	0.05074723	0	0.1494646	0.05195945	0.0803358
0.2846947	1.603392	7.129648	1.498354	3.246944	3.007422	3.520013	6.632762
2.170166	1.089817	2.133141	0.907729	1.286165	1.856605	2.235518	4.949623
0.02485145	0	0.002833585	0	0.003044667	0.03662	0	0.02952436
10.09102	9.298942	7.083548	10.48737	12.63211	10.5056	20.4472	12.5477
4.537801	1.828975	47.81345	5.613418	3.934396	5.43754	2.912071	10.42667
4.114607	0.1231698	0.8505476	0.4427125	0.7833494	3.383624	1.242886	3.873457
0.1279829	0.06762263	0.7421445	0.1999347	0.1209584	1.170028	0.09031351	1.582541
0.4509567	0.004886459	0.009640968	0.1359757	0.02071831	0.1601944	0	0.2511337
0.1902166	0.09269567	0.7210018	0.1091304	0.09825609	0.1883062	0.04063174	0.7800366
0.04270749	0.0177703	0.06038239	0.07417424	0.0460442	0.2355451	0.09844862	0.176858
10.39671	0.894858	4.363435	3.30831	1.924175	17.48544	4.880679	8.672428
1.16785	0.8992939	3.667933	0.9073282	2.60329	1.834235	2.427392	4.726064
0.2605177	0.8997528	1.445225	0.5599831	1.058009	1.415551	3.136914	2.033007
0.03293411	0.06362413	0.191514	0.04539671	0.04150195	0.07130977	0.04415707	0.1101939
0.01814491	0.03355545	0.4965359	0.02917966	0.08002867	0.08785195	0.005975337	0.07390893

TCGA-S9-A6WQ-01	TCGA-DU-7290-01	TCGA-DU-5870-01	TCGA-DU-6397-02	TCGA-HW-8321-01	TCGA-06-5859-01	TCGA-TQ-A7RI-01	TCGA-06-0221-02
0.1420423	0.06177403	0.03103366	0.08407578	0.5382782	0.7990928	0	0.4349363
2.440084	23.90851	1.959308	10.96188	4.043157	32.57613	1.500798	29.24774
0.8492491	3.197249	0.5671598	1.053369	0.6512407	4.256172	0.3544333	0.6272049
1.824849	6.955038	2.290692	1.657524	1.953512	5.096542	1.664522	2.048154
0.5425988	0.3782303	0.6423448	1.366671	1.139014	0.2673189	2.041337	0.9897985
2.357645	5.282199	1.811466	1.456492	4.588708	5.247878	1.850507	2.360608
2.308402	6.152798	1.025268	1.014474	0.9738032	5.185303	1.813961	1.533739
103.2039	241.7615	41.86601	6.021766	28.22079	72.94949	37.62711	29.41197
4.300805	12.14436	3.142365	0.762147	4.682076	10.65677	3.457839	1.206948
9.08258	9.873495	4.656532	8.841464	10.06527	9.465911	8.442373	9.800887
19.28763	22.70048	7.063231	8.225051	8.200955	19.01019	6.680765	5.96631
14.14021	36.24735	8.174312	7.181447	15.41165	18.74723	5.819638	4.785916
0.6981301	2.40453	0.4167302	0.4722603	1.641072	0.7135359	0.4485612	0.5725948
11.82779	25.28203	5.671197	0.9216288	6.245818	13.86201	5.363815	3.133073
355.5976	1067.264	411.3469	54.09663	333.773	125.5445	230.2448	114.2695
1.339995	1.505469	0.3637378	0.2884189	0.3644494	0.9137529	0.5948528	0.5222116
4.359953	16.97729	3.896051	3.890391	4.674223	9.875777	2.727207	2.553487
6.997944	9.109417	3.450948	1.507942	6.024407	13.12481	6.895358	2.47188
0.2324884	0.8654923	0.02438135	0.1321069	0	2.183655	0.04780096	0.1366817
0	0.1083975	0.0653473	0.05901254	0	0	0	0.03052805
8.661826	15.78215	3.935674	2.598708	4.997719	6.961308	3.718407	3.818099
0.9013957	1.855542	0.5199177	0.5548837	1.457448	1.78147	0.6548403	0.5078566
0.1302893	0.2369529	0.6831801	0.09114075	0.2456885	0.5330716	0.1522059	0.1668329
49.47784	174.1618	17.23464	18.85451	45.86183	82.6467	17.08523	25.68338
6.439876	10.68099	5.476386	10.0108	5.385699	16.44123	5.193765	4.665285
25.17097	35.95115	11.42806	3.206915	7.38832	22.28372	9.648387	6.858018
7.36857	16.30411	2.690031	1.124263	2.729763	6.840295	1.807223	2.763621
0.4407969	0.5268865	0.118167	0.08323527	0.23948	0.4524375	0.2251859	0.2384796
1.123544	7.751013	0.9110004	0.3841346	1.258796	6.222543	0.6963572	1.033336
6.783651	4.023725	0.3355707	1.060642	3.690672	2.701333	0.1127836	2.508277
0.05420287	0.3814501	0.03014413	0.04083295	0.04324318	0.3760415	0.05065647	0.2112348
2.440891	8.793983	1.243065	0.436552	0.9771768	3.388926	1.110233	1.016256
0	0	0.05667016	0.07676485	0.03448922	0	0	0.07942314
8.190296	36.11292	4.461956	0.7723045	6.703959	11.13536	3.120087	2.483999
2.249516	2.940031	1.277332	2.38561	2.299421	2.561988	1.117139	1.732778
0.006808989	0.05034075	0.005950559	0.01209085	0.02987725	0.04663288	0.006999837	0.0166794
10.97462	22.715	5.358478	4.809341	8.357941	16.94806	9.426026	3.697359
14.66458	13.58708	2.173109	5.170296	14.59886	11.22861	2.195795	11.28437
0.7664368	7.386001	1.201405	1.872243	0.8929332	5.439557	0.8004258	1.609264
1.157169	3.594653	0.2801819	0.1541847	0.2238042	2.50938	0.2883887	0.4417588
0.01158343	0.02015047	0.01012307	0.3633842	0.0323445	1.065311	0.01786215	0.1702496
0.4901815	0.8086112	0.1994196	0.07003418	0.2056478	2.369	0.07819459	0.3260673
0.06552742	0.197449	0.04704008	0.08311308	0.0485438	0.1717267	0.0962344	0.09459034
6.394128	12.62561	5.005366	10.22414	2.683597	15.03206	2.834951	2.52392
3.422084	24.4953	3.143181	0.8264555	5.694023	18.58243	1.435396	1.412401
2.397898	10.12046	1.452644	0.2083192	1.982399	6.044622	3.607209	0.4855986
0.1546892	0.3481437	0.06590371	0.05722597	0.1172409	0.2062093	0.1411347	0.1563082
0.0546863	0.380528	0.04344712	0.03531183	0.1150216	0.335619	0.01022165	0.2070297

TCGA-DH-A66D-01	TCGA-S9-A6WL-01	TCGA-12-5295-01	TCGA-DU-7012-01	TCGA-12-3650-01	TCGA-DB-A4XA-01	TCGA-DB-A75M-01	TCGA-S9-A6U0-01
0	0.07978243	0.7336637	0.115966	0.5588479	0.04734494	0.03511252	0.1860011
3.115809	0.9254129	102.751	115.5351	18.29783	8.605883	1.979677	16.27657
0.9219501	0.5837993	4.441456	3.911695	2.009746	0.6489441	0.4988243	4.076489
4.335452	0.8274405	3.765646	9.914216	2.636887	3.538915	1.036706	2.685038
0.9845816	1.327142	1.515911	0.6566272	1.112815	1.185189	1.40788	0.4232886
3.638549	2.376149	3.865074	5.027308	3.493352	2.626201	2.283274	3.484103
1.90129	1.11897	6.450472	4.275253	2.513134	1.285891	1.410163	2.944122
107.8083	21.87201	100.0079	272.2228	49.28566	20.78178	45.85073	158.4753
4.494197	1.19062	13.94678	13.81262	3.721927	1.267108	1.580649	9.675001
13.73327	9.3326	12.47889	6.052144	3.896048	9.258136	9.648999	14.46184
17.72949	4.912002	36.05283	19.84452	28.02563	10.51997	11.44285	32.99156
15.98253	4.120659	23.21668	24.8552	10.23299	9.169079	10.83763	22.29627
1.085044	0.5076634	0.2275161	1.910914	0.7663801	0.3781338	0.4992379	0.5917714
12.95081	2.705178	17.60322	19.16678	12.92743	4.641895	4.7192	7.997386
453.7132	44.13171	500.5168	501.2552	986.9837	102.4204	77.14153	263.817
1.085299	0.4789588	1.125572	1.085709	0.3794275	0.3789686	0.4818089	1.934149
5.36178	3.354293	7.279193	14.79907	4.639454	3.084915	2.029089	6.788017
5.834317	1.184996	17.7806	7.260185	9.796134	3.386009	3.632934	4.837235
0.3144105	0.01044674	0.794147	0.842747	0.1280575	0.2231771	0.1563199	0.1704853
0.04555072	0.02799952	0	0.1831415	0.09806342	0.06646255	0.02464537	0.0326384
8.561753	1.583857	8.423433	14.84761	7.080261	5.964605	6.194135	6.598256
1.103708	0.3510323	1.928538	1.81526	1.655	0.8733062	0.6357878	2.13251
0.1461113	0.2261958	0.122356	0.7204193	0.2796039	0.2210853	0.1112612	1.543252
68.35578	13.33386	73.5492	127.7611	49.2321	42.14448	43.18512	16.10492
10.69914	5.19636	19.03424	10.13762	9.739489	6.533491	6.2319	13.03261
33.44827	3.994709	24.13187	44.59986	15.77422	9.40648	13.44922	24.29268
8.364103	1.286276	2.569766	15.507	1.894732	2.828735	5.134811	3.845475
0.2817019	0.2126515	0.2085868	0.3488357	0.3830269	0.2776243	0.2968097	0.1841416
2.064427	0.3371797	1.776582	7.642915	1.380391	0.7462858	0.8582785	1.821806
1.603965	0.9859395	3.92882	1.462947	1.546692	0.4631898	0.2531173	3.280256
0.373716	0.0110708	0.6786995	2.321231	0.09693384	0.1708121	0.01948919	1.34857
2.418939	0.5276875	2.763361	7.601988	2.659929	1.381342	1.614807	2.466201
0	0.02428161	0	0.105882	0	0.02881866	0.02137284	0.02830451
11.28107	2.1986	47.26299	8.272995	13.41957	1.096709	3.632048	3.454285
3.088643	1.836038	3.844756	3.637224	3.566682	1.999464	1.445216	7.057378
0.01244362	0	0.009378439	0.1111797	0	0.004539086	0.006732662	0.04012294
5.568366	8.155592	17.20144	6.080746	9.08335	5.015272	6.00697	14.38332
2.650862	3.939344	28.14607	7.027299	16.61895	3.867844	1.670674	6.554039
1.578549	0.3963266	7.40638	15.6334	2.847925	3.292665	0.9262553	15.15005
1.698216	0.05627355	1.697336	9.430987	0.5617015	0.3740147	0.396259	2.099097
0.03175355	0.1106052	0.7259328	0.704541	0.2962281	0.1698813	0.05154111	0.1516822
0.3745456	0.06171087	2.857777	2.818603	0.8229623	0.1633852	0.2172733	0.6861487
0.1026456	0.0920137	0.2610947	0.3993229	0.09667855	0.09672584	0.02776838	0.4259685
6.507299	5.140381	22.33061	22.1924	9.955456	6.060465	2.277281	10.83326
3.440245	0.6792186	31.98963	10.23395	7.197842	2.144144	2.163364	1.583499
4.637951	0.7383277	12.67085	2.814502	4.104015	0.4975039	1.436028	4.342126
0.1696193	0.08905809	0.1065315	0.4309686	0.08368326	0.1031207	0.1013328	0.08608879
0.07268417	0.02233908	0.260206	0.2272934	0.08801854	0.006628292	0.009831506	0.03906022

TCGA-76-4932-01	TCGA-S9-A7R3-01	TCGA-27-1837-01	TCGA-RY-A83X-01	TCGA-S9-A6TW-01	TCGA-P5-A736-01	TCGA-TQ-A7RG-01	TCGA-19-2629-01
0.2276571	0.4178031	0.09330068	0.03111912	0.3741859	0	0.3018106	0.1383718
7.743653	3.484345	2.38361	0.4843221	1.824008	9.623537	4.140143	3.88045
2.091124	0.6024979	3.443561	0.3621157	1.012416	1.035688	0.5417284	1.585459
0.5388651	2.092392	0.6450941	2.099283	0.8740455	4.167354	2.038419	3.885067
2.820694	0.5206806	0.4347656	1.136473	1.338145	0.4807447	1.354991	0.416114
4.170488	1.725671	4.649848	1.604003	1.583836	4.058614	1.464403	3.572021
1.58465	1.53285	3.414738	1.213758	0.5731214	4.829478	0.9944132	4.50366
66.05982	46.18198	212.0462	14.01764	4.589691	116.9628	28.85611	191.6862
3.902801	5.333257	3.613224	1.681055	1.222964	2.77017	2.531185	8.788914
5.995816	15.07677	5.541398	7.753839	8.935361	11.20367	8.475438	7.279018
33.46575	11.07575	19.98263	5.185963	5.993262	29.23735	7.630944	31.57335
8.835606	11.48772	8.63673	7.687463	10.47373	17.81083	5.888913	17.3672
1.838229	0.6453791	1.433024	0.6036012	0.6239814	0.5647368	0.8022373	0.2246724
6.954473	8.221078	15.46196	2.923921	1.569378	13.20291	4.550167	20.58535
903.9921	100.3784	736.732	144.8481	20.10938	205.4981	150.9011	1101.676
0.5640334	0.8420393	0.8801776	0.4403561	0.3048624	1.062537	0.4252331	1.57732
6.822633	2.890292	7.470209	1.748799	2.087141	4.826641	3.329334	9.042143
4.482954	4.009029	4.141654	6.338346	1.392577	3.606445	2.406908	10.94305
0.1291745	0.2188291	0.3665047	0.07334546	0.1959841	1.13036	0.06774717	0.4348428
0	0.02932548	0	0	0.183848	0.1122078	0	0.04856137
5.523826	5.557538	8.753125	2.81	2.03106	14.75678	4.738387	10.95896
0.821876	1.095897	1.215744	0.4792202	0.6458833	1.765211	0.6493709	1.194219
0.423968	0.8988551	0.1867215	0.01037972	0.2308966	0.4665691	0.2948143	0.2711526
48.28145	29.80106	46.12596	15.89748	12.38405	78.74792	38.43908	71.63858
7.359779	5.901646	10.23565	7.752658	8.362416	6.588957	6.204372	7.723891
16.78012	9.405777	25.59052	4.581913	3.504708	35.89177	9.985725	26.67704
4.022998	4.532896	7.220263	0.938496	1.44076	9.339173	3.03402	9.336852
0.1791491	0.3181741	0.2771038	0.07109542	0.09688566	0.5661034	0.1379046	0.3292992
1.391193	0.8399179	2.312581	0.3092424	0.3675686	1.364939	0.876615	1.995406
2.989187	2.968811	4.900233	0.3525183	2.215731	0.4733139	1.753861	2.600592
0.08424064	0.04638029	0.07767975	0.008636326	0.1038459	0.188556	0.06581136	0.1104046
1.580795	0.8935802	2.306909	0.3000804	0.4070857	1.729312	0.9008217	1.680722
0	0	0	0	0.04555307	0.02432707	0	0.04211315
8.599745	3.145378	8.160537	1.876696	0.9937998	6.687957	4.769864	25.60766
2.528782	1.654181	3.363074	1.329619	2.333347	3.036123	1.244728	3.737911
0.003637682	0.01201677	0	0.005966945	0.00358742	0.02682147	0.004133626	0.02321562
23.3823	18.31178	9.16684	4.887006	8.670745	13.06068	8.323862	20.57587
21.4938	13.05284	17.42216	1.815911	7.894221	4.44903	13.04428	17.82598
1.260897	0.8874448	1.885883	0.2771906	1.999816	3.190242	1.240777	6.399695
0.8885157	0.3831	1.658374	0.05267876	0.1530779	1.020458	0.3953456	2.264293
0.03713047	0.115843	0.06086871	0.02537736	0.1220581	0.01955511	0.04219266	0.06770458
0.1760904	0.2237305	0.3663868	0.09998437	0.08905505	0.2901086	0.1539213	0.6380582
0.125028	0.04956229	0.09223235	0.02871194	0.1011064	0.1422298	0.05967095	0.2256992
5.342628	5.989934	10.90803	2.071395	6.130978	8.969877	4.599255	15.52796
5.827718	3.622924	2.440235	1.207202	1.018075	6.790873	2.096412	5.386066
3.481882	2.01056	3.386871	1.044176	0.388729	2.293101	0.7027636	5.675635
0.144624	0.1501509	0.2286176	0.01863941	0.04686273	0.09140106	0.09625705	0.1224371
0.09561598	0.07019092	0.03918629	0.01307003	0.03143162	0.03916659	0.07243454	0.3099528

TCGA-DU-5872-01	TCGA-HT-7473-01	TCGA-S9-A7J1-01	TCGA-RY-A840-01	TCGA-12-3652-01	TCGA-TM-A84B-01	TCGA-S9-A7R8-01	TCGA-12-0616-01
0.9248914	0.1932453	0.03879378	0.4283975	0.04657346	0	0.04530684	0.3185538
11.25885	65.43833	3.053009	1.555337	11.91209	4.855899	1.756179	11.05685
1.224505	1.722154	0.4597299	0.3058296	2.097975	2.717598	0.7374474	2.442416
3.24717	9.214449	1.790588	0.2474399	0.3133124	0.8595378	3.259575	2.440637
0.6469315	0.6317396	1.736257	0.9369343	0.9892281	0.9418753	2.857515	0.4625828
3.228878	4.232719	1.807573	2.326448	4.173197	2.282918	0.8892679	2.207394
1.56985	3.952188	0.708185	0.4335059	0.5847744	2.093228	2.606315	1.95165
59.15629	114.8093	25.60639	8.291827	27.4014	5.304254	61.51579	71.03711
2.754962	13.05286	2.420991	0.9750588	1.780659	0.9988567	10.46603	6.105403
11.36429	12.52997	6.678782	10.21447	6.780899	2.375951	13.75376	8.197556
8.683983	18.24899	6.70438	4.188567	12.66978	4.911661	12.00363	31.26212
12.7524	24.0861	4.546228	3.144429	4.734045	6.773072	18.11583	9.578167
0.5362458	1.045899	0.5413646	0.338392	1.091391	0.4458039	0.5089841	0.7213285
5.209532	18.67904	4.268377	1.081867	2.283128	1.694606	8.982854	11.39113
63.13857	538.8105	157.861	1.494987	275.9465	267.8873	364.6432	372.6502
0.6249467	1.160112	0.4824174	0.3618339	0.1131694	0.2299097	1.411763	0.9835086
3.910586	8.259484	2.826646	1.904834	2.355947	4.301325	3.737576	4.876959
3.471626	8.260522	3.200617	1.09141	3.142887	1.021732	14.80458	6.963238
0.3890059	0.7169355	0.1015934	0.05099502	0.1341636	0.05172999	0.3915445	0.5005386
0.01967208	0.04521276	0	0.02733554	0.03268977	0	0.0954022	0.2683104
5.930215	11.89752	3.444517	1.676786	2.106651	1.720808	5.146165	4.087915
1.086122	2.152887	0.3676348	0.1054486	0.4334786	0.4202328	1.096393	1.282999
0.2570797	0.859421	0.2264428	0.0129901	0.1165086	0.06118043	0.181344	0.2550072
61.10489	97.09343	31.66642	15.42438	25.8903	11.16845	23.04585	49.34578
6.098127	9.852776	5.424514	4.803623	11.05297	6.392607	8.179548	11.89116
10.92107	35.06311	7.438498	2.323959	4.43671	6.283962	17.01559	13.26982
5.140571	12.2362	3.384761	0.376732	1.492909	1.107948	2.853053	3.782381
0.3073495	0.3752686	0.2127099	0.05338508	0.3156615	0.01934089	0.3760827	0.3105174
2.090463	4.410921	0.8330527	0.2090762	0.8032818	0.9315178	1.278242	1.85569
5.498365	4.494266	2.936373	1.744638	1.942474	0.2906047	0.3732635	4.72397
0.4550242	1.376512	0.01076624	0.005404133	0.1357155	0.231028	0.02514754	0.4862362
1.62158	5.013017	1.237364	0.5392474	0.8406343	0.596558	1.478711	2.110879
0.05117976	0.03920919	1.227908	0.2607637	0	0	0	0.1551219
5.602356	20.05366	2.22333	0.6066088	2.678495	0.2479438	10.0607	18.51124
1.627495	2.916958	1.158353	0.6521094	8.028879	1.133548	2.036715	2.306999
0.04836644	0.08645906	0	0	0.01339537	0.01623256	0.004343687	0.07940557
7.75629	7.028227	6.760035	2.887841	29.03393	2.667903	14.87806	12.77231
24.80891	28.53834	8.428135	5.768889	26.75916	2.077345	2.318422	15.90053
3.072591	3.409527	0.5847804	0.4536398	2.521004	3.006625	0.1707396	8.534293
1.024009	2.094526	0.3611879	0.005493908	0.2102401	0.7603285	0.4921319	1.455978
0.379406	0.08404791	0.03796319	0.3938177	0.4253788	0.1840984	0.1403998	0.3013423
0.6403525	1.513895	0.1292591	0.05097862	0.2549397	0.1007403	0.5445355	0.697496
0.1422246	0.316265	0.05880269	0.06929873	0.1043577	0.08926666	0.03881631	0.2309313
6.768685	13.44026	4.568591	3.689082	7.551499	2.287734	4.253021	16.96323
4.496593	7.999718	0.9192983	0.4580255	3.270085	1.089743	7.046249	9.483513
1.037478	5.501559	0.7944498	0.2883276	2.624948	0.6874027	10.78771	6.923001
0.1327726	0.3647819	0.1436426	0.04241276	0.06086421	0.02612174	0.3009787	0.1595816
0.2628934	0.2254528	0.02172452	0.02726166	0.1238854	0.003950653	0.05708661	0.2854242

TCGA-S9-A7IX-01	TCGA-DB-A64P-01	TCGA-HT-7611-01	TCGA-TM-A84I-01	TCGA-TQ-A8XE-02	TCGA-HT-A617-01	TCGA-28-2499-01	TCGA-DU-A5TT-01
0.2073637	0.1511108	0.08447361	0	0.1129896	0	0.1582297	0.7948756
3.079422	7.2884	2.406157	1.29964	2.112426	64.48804	11.56961	4.890618
2.193037	0.7497438	0.6814469	0.5497092	0.8146896	1.426854	1.829935	0.9295458
0.3542838	1.948414	3.572779	1.116173	5.053334	3.370744	3.969528	0.8134189
0.3274438	0.8064988	0.6621577	0.4991127	0.8816022	0.4448741	1.148854	0.4881223
5.632368	2.024586	3.604393	1.854662	1.639186	2.335528	2.065954	2.8296
3.044187	1.00586	1.873058	1.579663	2.022391	1.801982	1.391412	0.9538871
147.2766	28.76114	71.24578	66.70601	90.35339	49.58816	64.25503	40.92076
7.03674	2.129278	2.781716	2.600548	11.62211	11.85479	4.185967	7.875451
5.162315	7.272709	13.42586	8.398699	8.662099	4.01275	6.545118	6.472065
16.99963	5.928366	11.13159	8.444898	14.81376	22.35637	14.87517	9.426746
11.2744	8.287924	11.24336	8.572038	12.03416	8.846292	6.034052	9.990892
1.362375	0.4509258	0.8723787	0.6347656	0.4660867	1.437566	0.7395001	0.9368273
11.80316	3.77076	9.670171	4.98797	9.370113	10.8133	8.629385	7.468571
278.5092	175.0573	223.0223	121.6537	411.81	1064.716	345.5971	139.6307
0.8087415	0.4265837	0.7486078	0.9669103	1.103602	0.9305533	0.4749511	0.6005434
7.571978	2.850693	4.572647	2.673009	4.769347	10.2832	10.29341	3.601208
4.582555	3.133727	2.954401	2.710503	10.94324	6.614526	4.735797	8.825453
0.1706715	0.3066908	0.3981961	0.2544216	0.009863261	0.07601539	0.2486238	0.4956252
0	0.07954819	0.0790557	0	0	0.2619485	0.05553048	0
8.173699	4.151736	8.623157	5.009312	5.300131	5.210166	6.289043	4.451509
1.062765	0.5881524	0.9339472	0.4566826	0.911422	1.361345	1.111226	1.063296
0.4347562	0.1827098	0.314632	0.1200176	0.288937	2.247562	0.1187487	0.08206378
34.08264	30.8474	99.88087	31.21939	24.2783	22.82369	48.29341	22.21634
7.548855	6.320471	7.204056	7.719148	6.853249	11.01419	10.5473	6.217208
21.33659	6.9788	14.51496	12.06294	15.70923	19.97869	15.10051	9.484712
6.0402	2.608225	9.335752	2.750425	3.814752	4.192135	6.197495	3.202011
1.38966	0.2128926	0.2616091	0.1452298	0.1089918	0.183156	0.2289469	0.1643039
2.473458	0.7378774	2.794937	1.907029	1.639059	1.50619	2.670349	0.968457
2.043955	1.575623	0.3044745	0.8152132	1.454488	0.5551434	2.159065	3.293809
0.1109865	0.07338975	0.08595958	0.09486634	0.02613117	0.4718301	0.065869	0.03151395
3.973564	0.8079741	1.486426	1.396793	2.342075	2.701633	2.543005	2.073
0.01803163	0.04599026	0.03427915	0	0.02292542	0.0757219	0	0.09215949
9.003455	3.123171	8.529924	6.071602	12.79326	4.628834	6.824983	11.54194
2.682917	1.620351	1.681545	1.199053	1.941633	3.522957	1.523755	2.644438
0.00852022	0.0434622	0	0.003449702	0.00361087	0.1033637	0.003792476	0
15.66656	6.973155	4.228086	4.726388	6.004467	16.74676	14.48809	18.91528
7.792133	9.597895	2.350914	3.52101	6.931462	5.621224	8.807115	12.47096
1.644098	1.061274	1.340888	1.257375	1.48386	10.68306	3.130531	1.348625
1.512761	0.1385594	0.7705993	0.7207791	0.8979059	1.222569	0.6305704	0.5926932
0.04831523	0.06777626	0.101035	0.04694896	0.2088552	0.7709996	0.1161314	0.02469387
0.2643852	0.2697292	0.2781115	0.2440634	0.4930044	0.5526614	0.2306563	0.1306228
0.1425164	0.04979352	0.1206203	0.05691218	0.1290707	0.2705468	0.2111642	0.1546603
7.12894	4.868034	4.70974	3.654053	8.260213	11.85457	13.50296	3.714657
4.31072	2.612717	3.420318	2.096937	3.074189	3.173563	8.637916	5.627608
2.555717	1.055581	0.9464952	2.090306	6.856219	3.520545	2.635739	6.180839
0.1806613	0.07405432	0.2821174	0.1645805	0.05537237	0.04064291	0.09908277	0.06595415
0.1078291	0.03702216	0.06701573	0.04533747	0.01581854	0.02902673	0.09968472	0.1271801

TCGA-19-2624-01	TCGA-KT-A7W1-01	TCGA-S9-A6U1-01	TCGA-DU-8167-01	TCGA-FG-8186-01	TCGA-06-5416-01	TCGA-02-2485-01	TCGA-S9-A6TS-01
0.3819543	0	0.3590397	0	0.381088	0.1838054	0.4915353	0.07424125
0.9380754	1.970585	0.9910635	1.279044	7.833475	6.081139	5.929957	4.087688
1.990521	3.210198	0.5767099	1.15756	0.9431263	2.974257	1.579064	0.8188535
0.811083	0.3867502	3.374792	2.838521	2.943488	1.889111	2.786194	3.385101
0.4252057	0.1835041	0.9104585	1.031294	1.71386	0.5776412	0.4083787	0.9976284
2.417965	5.228965	3.174266	4.210546	4.449982	3.273049	3.747221	3.383199
0.8657799	1.069497	1.950307	1.698149	1.1425	1.183934	1.276651	1.748645
18.90664	50.93066	51.41989	70.91086	74.69004	14.39753	37.92375	66.16833
2.141259	2.189006	2.129943	3.638505	5.945608	0.9218863	2.995771	3.030775
4.963806	7.288715	15.70348	15.36422	5.995627	3.47705	2.613165	14.11626
7.543968	8.559657	10.53735	16.77422	11.21188	6.334179	14.05049	14.25669
4.384407	7.500654	9.017635	17.90774	10.48783	4.264195	7.205986	10.56392
1.587768	4.04574	0.4096532	1.09545	0.9922579	1.892824	2.045579	0.4658884
3.688099	3.091562	9.206234	12.75959	8.778682	2.394464	6.464666	9.508091
167.5673	175.1724	113.096	325.0476	400.0662	154.2003	352.8339	127.4498
0.4814288	0.372023	1.000734	0.8004615	0.5492511	0.4729034	0.6264678	1.034645
1.663431	3.555976	4.026364	6.544785	5.947109	2.996677	7.869677	3.637401
2.790819	3.075757	5.240192	7.463352	6.158627	2.115347	4.876315	3.308081
0.1363997	0.1067571	0.09402555	0.2204646	0.08316632	0.3690357	0.3647168	0.16526
0	0	0.05040177	0	0.02229038	0	0.143753	0.07816457
1.74514	1.712608	8.360794	15.12136	5.341559	3.027955	3.636969	7.717512
0.6052327	0.5465801	1.172644	1.604256	0.7631305	0.3732553	1.420999	0.8857289
0.1158183	0.06275664	0.155684	0.1587118	0.4501852	0.04087195	0.2322633	0.4271621
15.21134	18.94789	45.02632	178.6518	29.47272	20.96733	31.14064	27.31342
10.88382	7.033536	6.255444	7.814927	5.326148	20.68833	8.195377	7.623395
3.929685	5.29342	16.32444	17.71306	14.60366	4.547875	8.309865	14.23819
1.396248	2.617043	7.988192	9.40708	7.204158	1.045894	3.014486	2.767043
0.1824572	0.1265666	0.2788918	0.4264735	0.2660295	0.1166461	0.2245945	0.4947052
0.5830268	0.6447259	1.1729	2.521026	2.227229	1.112722	1.48315	1.081205
1.430344	0.4843989	1.294113	0.433478	3.270437	4.13276	1.455306	0.7836658
0.01445479	0.08702658	0.03487484	0.03047407	0.1189815	0.1105229	0.1818843	0.08756609
0.5237142	1.147513	0.8566738	2.715029	3.930012	0.6059584	2.243329	1.376741
0	0	0.02185459	0	0.1546444	0.07458771	0.02493295	0
1.837488	2.504782	4.488245	13.41961	8.675408	4.110893	6.355566	6.352664
2.339973	6.533616	1.462984	2.569255	2.229173	2.769413	1.611617	2.345385
0.009986994	0.003006387	0.003442209	0.003509153	0.2161709	0.01174794	0.0117812	0.02135312
7.715101	11.30236	9.983726	9.363657	10.03334	5.760051	20.03555	11.33458
3.335466	2.252133	5.608486	3.384532	10.94786	14.46594	5.92043	3.632417
1.094421	3.631155	0.5289186	2.16936	1.969249	2.686736	5.599178	0.763035
0.176339	0.8670283	0.2988282	1.043004	0.8108671	0.3284337	1.265449	0.2042241
0.06229608	0.01022891	0.01756762	0.05372782	0.08287304	0.1698773	0.2137828	0
0.04545195	0.1268733	0.1153579	0.2221359	0.4799421	0.1895621	0.2632136	0.1281015
0.08466963	0.2479925	0.05205618	0.09166389	0.144411	0.137285	0.1592695	0.07339116
3.733622	3.773204	5.852169	11.27602	5.203389	10.61388	3.775185	5.670343
2.535553	4.287934	2.590269	8.827846	3.088263	2.054145	5.212811	2.280572
3.949335	3.113527	0.7395572	1.791973	3.737153	0.7681864	2.247497	2.369208
0.1985273	0.09220536	0.3597266	0.1793751	0.1296933	0.09007683	0.1427466	0.08287268
0.01458371	0.05268165	0.08545144	0.06149173	0.2134093	0.06004311	0.0516112	0.05196887

TCGA-DU-A7TJ-01	TCGA-CS-4942-01	TCGA-P5-A5F6-01	TCGA-TM-A84J-01	TCGA-VW-A8FI-01	TCGA-DB-5274-01	TCGA-DU-A6S7-01	TCGA-28-2514-01
0.03104447	0	0.2372335	0	0.4866582	1.715294	0.3013356	0.721939
3.62826	3.10885	4.876467	0.6850519	2.357975	2.027075	0.6194121	5.311107
1.748613	1.11689	1.636009	0.6866221	2.421527	0.4689071	0.8900634	1.722476
1.699763	7.072634	1.214684	1.00955	0.5911176	2.1812	0.4012101	1.512387
0.2960525	0.2294566	0.3393531	1.632176	0.5326565	1.029159	1.334781	0.774117
4.795168	2.268692	1.425246	2.368035	2.657939	2.077872	3.304415	4.358
1.642107	3.616517	0.477436	1.253737	1.781134	1.229417	4.172646	1.28238
193.5768	223.6093	6.380442	12.18624	44.41221	17.62222	78.30598	61.45058
4.632869	9.085615	1.281536	0.7167806	4.969753	2.925622	4.041664	2.867998
6.21644	5.248519	11.46001	7.662874	3.540388	6.183313	23.16942	7.292577
11.8512	21.90392	4.129178	5.589064	16.96363	23.49174	16.94831	10.85841
11.67434	18.50575	3.726418	5.567537	8.139675	9.310707	15.74493	9.396931
0.8255766	0.252943	0.7787144	1.167431	1.392426	0.3598416	0.4991924	1.443994
8.125065	24.63608	1.427866	2.909224	10.07958	5.600122	10.83287	7.124757
370.6883	691.6775	61.15228	423.5817	774.8286	178.7017	175.452	903.7338
0.3860513	1.895764	0.2102371	0.4797034	0.8486438	0.7415116	1.37291	0.4399198
7.615416	12.1793	14.11462	1.816701	4.683107	2.095299	5.296649	5.320754
3.191151	11.32366	1.287109	1.426828	6.317225	6.532804	11.04989	2.994305
0.1788588	0.2695489	0.2609329	0.05359088	0.1529357	0.5587639	0.2170134	0.15921
0	0.1444899	0	0	0.170792	0.1468244	0	0
5.403685	11.63687	1.875894	2.118684	6.253306	5.557995	13.523	4.226737
0.6356763	1.094851	0.2007301	0.4086353	0.8647285	0.7646187	1.05812	0.9387851
0.0569515	0.8435734	0.1661704	0.1023852	0.1379753	0.1883852	0.2324291	0.1774325
37.38883	89.37285	32.1096	22.25721	24.43365	63.8188	76.25616	31.9551
7.228567	8.595329	3.06131	12.02892	7.706294	13.36892	10.3269	8.476165
15.74981	34.98049	3.901575	4.229505	15.73284	5.716727	22.77823	12.40534
4.009954	9.789292	1.133926	1.855339	2.676887	1.817181	8.901979	3.812507
0.2647862	0.2754637	0.06503868	0.07168667	0.2038355	0.1210686	0.6683586	0.3530204
2.233969	3.70505	0.3251695	0.6124085	0.7115189	0.4539738	1.802715	1.905302
1.454646	0.7268405	1.441418	2.107411	3.357845	8.918398	1.74556	6.652082
0.1163107	0.2285209	0.06583818	0.005679226	0.0472709	0.1219118	0.01045351	0.05272525
2.306611	2.734007	1.049965	0.3845446	1.738752	0.5534192	0.5727714	1.413934
0	0.01790055	0.08664181	0	0	0.4074505	0.02292771	0
8.214098	17.57064	1.373833	3.015807	3.819235	5.614147	8.349169	14.24959
2.294364	2.54698	9.709636	1.134297	2.175672	1.652952	2.41408	9.173598
0.005952631	0.02255542	0	0	0.004665722	0.07620845	0.01083369	0.01457141
5.968727	5.101153	14.91481	8.757692	22.38643	16.06511	13.9154	8.231161
4.737913	4.329761	9.796881	15.57892	7.951505	52.3566	9.130156	25.03784
1.4039	3.032568	1.381666	0.5748827	4.601623	4.342855	0.1935663	1.21062
1.983853	2.128191	0.1204774	0.2482635	0.82382	0.548864	0.318815	0.4931304
0.04556968	0.0383712	1.501264	0	0.01587462	0.334349	0	0.117747
0.3915896	1.392805	0.02823041	0.01948133	0.1216142	0.3385359	0.3092792	0.20347
0.153445	0.08721393	0.08129927	0.03236726	0.1186678	0.01930012	0.02234139	0.1051728
7.9213	14.75654	1.133718	3.213263	5.52353	14.03099	5.400212	6.420268
1.858229	4.465219	3.348056	1.048892	3.442624	2.269205	3.484429	4.855554
3.130575	2.990136	1.090629	2.260317	7.508043	1.788455	4.841888	1.882934
0.1943991	0.1889559	0.03875338	0.08022915	0.1006978	0.01366842	0.2953488	0.1634505
0.03042358	0.230559	0.06642539	0.06875855	0.05450572	0.07614232	0.06328048	0.2021429

TCGA-TQ-A7RJ-01	TCGA-06-0747-01	TCGA-27-2521-01	TCGA-14-1829-01	TCGA-HT-A5RA-01	TCGA-12-0821-01	TCGA-DB-A75O-01	TCGA-S9-A7J0-01
0.1893234	0.3238832	0.6065243	1.667274	0.0372473	0.2378542	0.3067642	0.03259414
1.06557	2.990636	13.06855	79.4685	13.70494	9.382657	1.351223	2.517251
0.5991937	1.878001	2.714324	6.131507	2.962187	4.813893	0.431736	0.4165096
2.116821	1.09615	1.466341	3.204631	0.5429076	1.081556	0.8844371	2.076971
1.484037	0.822868	2.723602	0.8775863	1.780062	1.383303	0.6886151	1.953289
1.744895	5.325201	3.836666	2.813107	7.755783	3.788949	2.954438	2.102821
1.857314	1.185709	0.9316826	0.6659926	2.918825	0.889592	4.483915	1.181312
75.88967	82.38826	26.29481	194.7682	57.1224	56.60371	97.4123	26.07374
4.238005	2.973727	1.631677	10.26977	6.403803	2.987317	3.55642	1.503459
13.95061	5.926193	8.232452	10.31726	7.662607	6.138315	5.647266	9.235999
15.34193	9.723227	11.87415	39.92062	13.57541	13.48185	20.74629	7.629631
12.49089	5.474575	4.640531	4.658628	7.756576	5.152771	21.85064	4.075237
0.6923468	3.218478	0.2761448	2.061183	1.529929	1.116851	0.2307748	0.517784
10.33374	4.528007	4.212187	20.9782	4.914822	6.197132	9.136316	4.536069
98.45686	561.1819	236.9037	2094.911	388.7416	544.7503	282.0893	123.1984
0.8704664	0.4063634	0.5201649	1.252848	0.6548494	0.6232953	0.989704	0.670878
3.995719	2.579941	2.244593	8.832969	14.05334	4.837369	3.589513	2.009127
3.781156	3.186888	2.685586	5.558832	4.46758	2.408534	10.04601	2.162992
0.2479008	0.2638805	0.1092004	1.659182	0.06828043	0.2595393	0.2295303	0.1877871
0.02214759	0.05051836	0.0532147	0.06687171	0.02614377	0.05564972	0	0.06863319
6.385727	4.413135	3.937406	5.666145	5.747046	4.270324	9.654342	3.374569
0.7529024	0.4871944	0.654327	2.490944	0.6618361	0.8184382	0.7564379	0.4081668
0.7840932	0.08402378	0.1390846	1.604792	0.1739326	0.4561808	0.07308613	0.22287
32.42985	21.8437	33.8272	88.47127	35.68283	40.74286	51.41928	15.46932
7.905933	11.4631	5.354632	22.04811	13.03205	13.66832	5.449916	7.08521
18.36008	10.89625	7.849289	11.4502	14.25271	8.948643	19.18375	10.1546
4.698193	3.453858	1.970093	5.529661	4.295427	3.233226	5.294856	1.891773
0.4301296	0.2713152	0.1760967	0.7110305	0.05389413	0.208306	0.1935654	0.2085028
1.153335	1.340034	2.338161	3.98837	1.629469	2.073851	0.9360536	0.9121328
0.7473822	2.668334	2.459408	41.52673	5.389306	13.00262	2.617559	0.5370581
0.01751398	0.02996187	0.02630086	1.500502	0.6202228	0.1870296	0	0.01356852
2.254741	1.570494	1.021646	6.701886	2.187279	0.882161	1.018545	1.261245
0	0.04381028	0	0.1159843	0.09068907	0	0	0
4.845507	8.688243	5.949912	26.12157	5.481785	7.615431	6.528378	1.972159
2.717213	4.698908	2.135596	3.053024	2.430196	3.286214	3.564131	1.381888
0.01210063	0.003450171	0	0.01826812	0	0	0	0.003124887
7.863279	16.48743	10.24979	10.24368	12.19198	9.427507	10.1665	4.782007
2.86107	12.82597	16.77757	132.1452	24.81148	34.52115	6.806231	2.574993
0.5729371	0.7643909	0.9090844	2.953899	6.09959	1.996434	0.2852583	0.3573283
0.249269	0.812256	0.2192498	0.9273533	1.050877	0.5760019	0.6862095	0.2712805
0.03087832	0.05869419	0.3709614	2.113281	0.09112452	0.387936	0.007147529	0.02126419
0.2553308	0.1798607	0.1443508	2.6586	0.4299397	0.2217168	0.4328385	0.1706606
0.02703359	0.08537989	0.03247721	0.853916	0.5768592	0.1828794	0.03176922	0.04081317
3.715979	8.107573	8.993196	107.4021	5.657907	8.017686	3.291019	2.308653
2.713999	19.82254	2.827989	7.324933	1.183406	3.200943	2.753913	1.498999
2.27578	3.863187	1.371548	4.670059	2.795376	2.539625	4.066005	1.436171
0.1683804	0.2488639	0.07843756	0.2256683	0.06490184	0.09281974	0.05249786	0.177481
0.03092283	0.09572547	0.1432914	0.9136664	0.2763749	0.1498482	0.03681171	0.03194226

TCGA-DH-A669-02	TCGA-27-1834-01	TCGA-HT-7478-01	TCGA-28-5204-01	TCGA-DU-A76O-01	TCGA-DB-A64V-01	TCGA-CS-4941-01	TCGA-QH-A6X8-01
0.1138462	0.1857298	1.703088	0.4601915	0.4983716	0.1075329	0.7412742	0
1.693838	13.25315	13.7281	6.461938	3.579878	6.441735	8.454527	0.9627347
0.7937748	4.836257	1.71836	3.339029	1.360188	1.021	1.69129	0.4412811
1.496291	1.98698	16.02408	3.385091	2.500184	5.063824	6.765363	0.8232476
1.542163	0.5635617	0.7320902	0.4896339	1.179859	1.375072	0.3598809	1.450749
4.520887	5.959509	4.587825	7.668691	4.455825	3.517703	6.341048	2.65208
1.22331	2.828189	3.975989	1.452918	2.137051	1.595956	2.175689	1.736485
47.39926	193.063	185.2728	69.70421	143.526	37.86376	182.7741	26.78603
1.310496	6.614302	15.58202	4.623111	7.513016	6.719614	5.660963	1.255165
7.812477	6.378734	9.100008	4.601061	10.43737	5.246838	3.925387	5.323115
18.25794	22.14619	18.32112	16.88092	23.64408	23.02529	34.27339	8.932075
7.537266	10.96583	32.8697	10.35781	15.41831	9.766843	13.16911	8.309626
0.1998384	1.05956	1.185832	2.8493	0.5147922	0.5127887	0.775507	0.4196264
4.1909	19.80307	23.23002	11.74122	12.58625	13.09574	18.61758	6.868691
97.00833	747.6123	543.7759	621.825	60.73817	501.7186	568.6544	607.5507
0.4339391	1.048625	1.910945	0.5262234	1.287714	0.9068493	0.9663074	0.6052955
2.533061	14.72675	13.87842	11.31018	5.342608	4.578046	12.54562	2.067914
2.335411	5.569281	16.39925	8.084942	6.299959	8.176708	6.494672	3.361186
0.1689467	0.5228697	0.5872409	0.2410305	0.2509883	0.1032563	0.4503709	0.2951356
0.07990829	0.1303632	0.09961602	0.02936427	0.02690812	0.07547705	0.2081193	0
4.367614	10.84363	17.14278	5.235483	9.980475	10.05832	11.4896	5.532494
1.040348	1.359359	2.118316	1.146905	0.6811859	1.746945	1.329692	0.9940291
0.05063088	0.2168243	0.3171676	0.2511752	0.1790178	1.093956	0.4203259	0.1309967
52.18706	52.1977	100.1401	34.18385	82.80213	49.44075	43.05626	26.91995
14.43605	9.431789	9.110861	15.11424	8.481672	7.850088	8.44907	7.277679
8.222172	28.1285	35.336	11.86045	22.3474	18.07838	27.33789	11.96961
1.864254	14.73417	14.38029	6.49887	4.966366	4.867897	10.89935	5.259789
0.1647272	0.2510574	0.4215155	0.1561116	0.7940941	0.2620503	0.1919336	0.1482423
0.5418258	4.842223	3.757696	1.87798	1.637699	0.8679778	2.729772	0.7138513
1.152872	2.677758	11.80943	2.972739	1.57918	0.8120911	8.015488	0.8088981
0.02106342	0.5927631	0.2875282	0.2147926	0.01063927	0.2337706	0.4237873	0
0.703727	4.563567	5.512869	2.001578	6.203908	2.853789	2.126095	0.5741943
0.04619845	0.05652646	0.0518331	0	0.04667025	0	0.03609683	0.0207876
3.432853	7.399191	52.61228	13.94319	7.050653	3.421432	11.92499	4.194103
2.810889	2.313282	3.586862	2.991817	4.287852	3.199025	2.783244	1.346487
0.01091474	0.04896763	0.005442652	0.05615245	0	0.00687298	0.05969707	0.006548307
8.683041	13.08351	11.89947	12.02089	9.786962	16.82073	10.37296	4.820503
5.110253	10.12936	48.95162	9.577232	4.370867	8.302214	22.7593	2.023495
2.067151	5.933465	8.440797	3.640471	0.9456282	6.041758	8.167195	0.4445962
0.4818	4.847082	2.995122	1.699673	1.032929	0.8697127	2.421835	0.5010311
0.07427244	0.1893263	0.06481314	0.1501128	0.01875774	0.04092301	0.2031129	0.01670993
0.06773756	0.8840619	1.354476	0.2290049	0.6204259	0.2516595	0.4939785	0.1503651
0.04501704	0.6120104	0.1010152	0.1764546	0.06568868	0.210241	0.2188589	0.07652271
14.18445	26.74647	13.92819	10.46055	8.080681	12.4802	11.6649	2.564649
1.172385	5.216295	11.64906	2.768521	2.82631	3.30324	3.903559	1.726463
1.821644	3.335012	7.668573	2.687623	3.126589	2.405453	3.556551	2.33464
0.01859739	0.1441148	0.5162315	0.1321253	0.1669984	0.128818	0.09202928	0.06694513
0	0.1300109	0.6994014	0.1757095	0.1127087	0.04014563	0.1037784	0.004781149

TCGA-S9-A6WP-01	TCGA-DH-A7UV-01	TCGA-S9-A7R4-01	TCGA-DU-7013-01	TCGA-FG-6688-01	TCGA-DU-8161-01	TCGA-HT-7858-01	TCGA-QH-A6CV-01
0.1592408	0.163844	0.1207049	0.1120533	0.4318089	0.2132289	1.314074	0.03662026
4.778994	0.4907519	2.103075	5.027812	11.74721	23.12052	27.69423	6.226316
0.7889444	0.88427	1.232235	1.395094	2.212902	1.826668	1.214728	1.680989
5.832396	1.353285	2.624011	4.774148	2.14409	2.256268	6.982698	1.902404
1.608319	2.617158	0.6409481	0.3035752	0.2038897	0.4477025	1.540882	0.2460452
2.505858	2.181204	2.376024	2.849586	2.421544	3.598351	3.243436	2.650076
1.857618	1.464856	1.408079	2.243115	2.480173	2.760364	2.535377	2.820773
30.80575	35.16937	80.35621	94.3501	138.2212	194.2867	40.19692	100.7573
3.020586	2.663339	7.438506	5.378706	10.07688	11.79475	5.713975	9.186558
6.346213	24.66299	17.01509	4.265008	2.160839	9.358684	11.59837	10.96808
13.84362	13.72299	12.63416	9.734447	15.78656	20.51861	12.87833	26.78982
10.89456	13.05745	13.42038	8.359756	13.72845	14.49946	12.2444	11.78655
0.3717634	0.5636989	1.617334	0.8851106	0.5089229	1.017597	0.7895815	0.7520865
7.563454	8.268472	8.853893	9.567121	15.11592	14.55787	10.14456	17.10927
294.5925	113.1738	121.1082	742.2076	1084.173	325.4269	227.5961	672.94
0.9787335	0.6978923	0.8569026	0.8969207	0.8332339	1.062923	0.6742559	1.198667
4.09433	3.597935	4.68391	4.275612	6.726584	10.7439	4.955297	7.011125
5.615739	4.676089	6.178997	5.041916	8.522642	7.505323	6.60978	7.47524
0.05838294	0.2917716	0.1685882	0.04108242	0.09692778	0.2582624	0.5647271	0.07672111
0.04470826	0	0.141204	0.07864984	0.1082448	0.05612426	0.09459944	0.0514073
8.179313	8.108329	6.443945	5.714252	6.84135	9.689603	12.84747	7.932502
1.573743	1.036976	1.116642	1.1567	1.586732	0.8073753	1.328549	1.388148
0.6267506	0.1912744	0.5301015	0.2915261	1.172807	0.5467508	1.213772	1.355822
35.37396	62.13688	66.66917	19.19193	36.11328	63.13247	62.05929	26.382
7.389775	7.65675	5.388545	8.789783	7.520771	8.332766	6.933432	20.69852
12.71099	13.91253	10.75355	17.06722	30.75271	34.27368	21.27711	26.74455
5.279635	5.612501	6.074699	3.710879	4.428611	11.75404	5.886035	5.313625
0.2449621	0.6288604	0.4167117	0.1553063	0.1691177	0.2557524	0.3284417	0.2537791
1.069508	1.302544	1.934809	1.820022	1.941188	4.161759	1.454787	0.9411447
4.821293	1.586062	1.160175	0.6808293	3.239854	2.388015	8.64005	0.4148354
0.08396722	0.01818831	0.0446648	0.1990243	0.05991883	0.5732703	0.2244234	0.05081514
1.07095	0.7413571	3.969376	1.388059	2.249735	5.130508	3.253284	2.453785
0.0969292	0	0	0	0	0.06489574	0.04101903	0.02229059
1.984261	5.169487	8.364003	5.773032	11.10501	15.19574	5.214548	6.413289
2.248683	2.246114	2.920495	1.733913	3.475237	4.331041	1.867646	6.104609
0.009160105	0.006283265	0.007714867	0.01074284	0.008871143	0.4369652	0.01615176	0
9.749967	10.1533	23.49488	6.718337	14.62918	6.910343	9.735109	19.82272
9.663959	5.08933	5.020794	4.0037	13.90021	8.709863	27.43572	2.268425
1.789395	0.482733	0.2481156	3.247677	2.451487	8.59258	5.067545	2.458982
0.6065202	0.3189604	0.9365149	1.343601	0.9659255	3.365163	0.8745811	1.017688
0.01558314	0.005344534	0.03937351	0.1096541	0.08048831	1.42152	0.08792743	0.0238908
0.284241	0.2144689	0.2824859	0.5867029	0.7267246	1.579525	0.577376	0.4488484
0.04617579	0.02591487	0.1007615	0.06203131	0.1199286	0.4865674	0.1176896	0.2027253
5.734663	5.033548	6.832786	5.221054	9.659857	7.642516	8.511144	10.15654
1.956649	4.198982	5.169818	2.549118	5.863441	2.704235	5.695633	4.152546
1.192236	1.88407	3.963673	2.395067	6.992208	5.574505	1.740129	7.263254
0.1144565	0.2444519	0.2234684	0.158639	0.07557677	0.3773476	0.1981487	0.1196423
0.03566994	0.0137629	0.08449343	0.04078738	0.06045325	0.2015013	0.1084953	0.03588785

TCGA-TM-A7C3-01	TCGA-HT-8114-01	TCGA-S9-A6TU-01	TCGA-06-0211-01	TCGA-HT-A618-01	TCGA-QH-A6CX-01	TCGA-TM-A84L-01	TCGA-RY-A83Y-01
0.07570454	0.3440085	0.1147693	1.128318	0.4040048	0.04042275	0.208917	0.09493092
3.912603	23.61585	1.190807	8.41789	8.174043	1.982319	1.668683	5.686814
1.183582	1.338438	0.8848747	3.652426	1.897776	1.849764	0.7844983	0.5286093
1.01857	3.457073	2.39489	2.655358	2.770697	1.65427	1.545986	2.953646
0.2584249	1.060397	1.243734	0.4623477	0.9150286	0.2321686	2.010428	1.635711
1.531127	1.745105	2.722773	4.61581	3.737291	3.435787	2.824035	4.625779
0.6505489	2.149467	2.357442	2.135114	3.410559	2.037384	2.254795	1.555451
46.15338	81.62223	86.91752	137.1888	198.2152	116.8012	60.90308	13.59554
3.30526	9.164386	3.968083	5.448953	12.96007	2.567518	2.772468	1.691827
7.187062	13.54631	14.80085	8.468185	5.154778	2.962467	17.26721	5.064647
10.78791	13.65388	16.93019	27.03516	22.6979	13.49152	18.5813	13.38851
6.455993	15.56734	17.04899	10.05865	24.6117	7.978609	13.83341	7.697345
0.8571211	1.362019	1.034155	4.638886	2.655822	0.70246	2.530366	0.5623957
2.33496	9.42615	9.205134	9.521239	16.60882	9.010808	11.92571	4.679565
60.68096	68.48392	123.4773	836.1446	263.5722	622.2875	193.0504	61.69553
0.5843287	1.608991	1.137389	0.987823	1.524516	0.6297873	0.9376592	0.4884856
2.181074	5.811831	3.588495	8.876884	10.2421	3.977899	4.129294	2.305663
1.565713	8.537265	10.61815	4.213398	6.85203	4.79408	4.355926	5.464393
0.06938945	0.3703666	0.3306144	0.175444	0.708867	0.2117187	0.2297876	0.07458172
0.05313679	0.05365745	0	0.02474884	0	0	0	0.1332635
1.984845	7.793051	9.773822	6.692705	17.94265	6.230228	8.72192	5.844607
0.531663	0.5530434	1.035835	1.044206	1.080215	1.094491	1.131332	1.164695
0.01893832	0.3155442	0.07018193	0.09996748	0.107804	0.4449365	0.2787354	0.1583201
20.959	44.70132	52.26346	58.39651	102.7176	21.16103	91.28038	41.73029
8.875368	6.138265	8.806612	12.29365	9.456411	6.853506	6.437862	6.43419
3.98669	16.93904	13.6155	17.17097	22.07897	17.22067	20.74582	6.091612
2.354907	6.883823	6.269402	4.079617	9.915825	10.17431	9.145715	3.160042
0.1037738	0.3332923	0.4632277	0.2738891	0.7814719	0.3755595	0.5791196	0.1554316
0.5058599	3.012508	1.59059	2.04999	5.366841	0.660259	1.78973	0.3307196
3.527786	3.326175	1.221315	6.517901	6.011961	1.561057	1.850265	1.05094
0.0735346	0.8221103	0.02123421	0.2006029	0.2578791	0.01682747	0.0985654	0.01317284
1.562983	3.097527	1.144552	1.739283	8.016361	1.474224	1.869942	0.7569778
0	0.01163314	0	0.1717006	0	0.0492103	0.07630012	0.02889202
1.103582	10.49135	7.242232	16.30018	23.42035	5.553593	7.909917	1.450203
2.076269	3.201671	2.612791	2.413958	3.482354	1.513742	2.185059	1.62087
0.01088699	0.2052152	0.003667745	0.04056557	0.0116199	0.01162631	0.004005886	0
7.518204	12.52659	30.9106	9.599211	10.04646	14.07708	8.000239	10.47089
3.364194	10.29442	4.49914	35.8962	21.72537	6.568381	6.601922	3.068062
1.685828	2.376738	0.7601692	1.207979	2.394477	1.440248	1.302636	1.707438
0.6140685	1.685015	0.6853858	1.153975	2.735614	0.5645314	0.9607679	0.3749668
0.02469455	0.6483496	0.04991643	0.2472858	0.04612473	0	0	0.3096613
0.06306103	0.9483612	0.1957554	0.5454638	1.399009	0.158738	0.4226334	0.0847247
0.2020618	0.2896888	0.02773347	0.1394248	0.4499668	0.1625036	0.1018856	0.2002004
6.105128	9.557302	3.460599	5.927735	9.239796	9.796822	4.742381	9.032826
1.136184	4.216892	5.5334	3.39793	4.489012	2.284769	4.180605	2.099611
1.446379	2.74538	2.859689	3.197787	5.24151	3.533272	2.504654	0.9436961
0.2679462	0.2393518	0.2124793	0.2015965	0.2199879	0.1078535	0.1865649	0.05427618
0.04239454	0.2327791	0.04820309	0.1480917	0.3167398	0.01131837	0.05849676	0.01993549

TCGA-HT-8015-01	TCGA-DH-A7UT-01	TCGA-VM-A8C8-01	TCGA-DU-8158-01	TCGA-12-0618-01	TCGA-TM-A84C-01	TCGA-TQ-A7RK-02	TCGA-WY-A85B-01
0.189895	0.2781591	0.0459309	0.2520215	0.497528	0	0	0
1.316002	0.8304301	0.3102158	20.95051	14.98007	3.042934	0.5070738	6.654715
0.7130693	0.8704229	1.042712	2.047772	1.245502	0.7808631	0.7113953	0.9769048
0.2058155	1.238838	1.502033	2.281963	1.580531	0.2259148	3.778084	6.215502
0.2592901	1.969382	0.6371134	0.8118849	2.282453	0.3181712	2.03893	1.221491
2.171498	3.315333	1.787355	3.175206	3.17019	4.971763	2.652489	2.470562
1.1465	1.795817	1.541974	3.080722	1.562473	2.42739	2.165589	2.732978
83.67927	50.62402	45.31604	174.8509	24.61663	89.06121	54.41015	111.2061
3.147699	2.435747	4.877402	8.403618	3.223539	7.185381	2.91504	7.399625
2.846545	8.239925	15.71841	9.477075	5.775241	3.952911	12.42389	24.37399
8.925279	15.609	13.6077	13.56839	7.349522	10.5976	15.79836	18.54
10.00372	11.2165	8.851003	13.75242	6.587838	7.688781	11.95982	17.54377
1.52665	0.9561818	0.2781535	0.9229711	0.8645961	5.972235	0.8809139	0.531087
6.345667	8.535088	8.524915	10.82277	4.697306	7.310161	10.8263	12.64535
335.6467	316.5639	121.5545	371.6587	630.6892	1391.92	260.2793	323.6132
0.711142	0.9343352	0.7156044	0.5927411	0.7727792	0.7446959	1.095267	1.571557
3.699246	2.923661	3.127415	9.396476	2.750119	6.545137	4.082518	5.803204
2.818352	6.288126	5.900426	6.41294	4.138236	3.797774	6.714393	10.4052
0.6365418	0.3035188	0.1082557	0.1259992	0.1389792	0.3241386	0.1601589	0.2900048
0	0	0	0.04824358	0.02328089	0	0	0.02775985
4.511074	5.459937	6.225063	5.688311	3.545171	11.28887	9.347615	12.91355
0.3920485	0.7453017	0.7694963	1.039073	0.4939413	0.2825226	1.461088	1.412188
0.1013425	0.100511	0.4366245	0.3744547	0.2378609	0.1056112	0.2039886	0.4946901
37.56252	32.7747	38.62764	75.4173	31.54764	38.39066	75.20185	86.05311
4.900887	9.530274	6.515979	11.28035	7.527367	5.571618	9.01361	8.806492
11.82066	14.15709	15.8043	27.10572	8.139442	20.6885	22.67066	22.50018
4.862452	4.097661	4.556325	5.379892	2.28371	5.465136	9.546067	9.827046
1.084596	0.3389272	0.3882586	0.3541882	0.2273327	0.1841328	0.3314952	0.2891399
1.379504	1.027298	0.6295622	4.276115	0.9888246	1.975995	1.77515	1.581118
1.310238	1.456142	0.543957	2.147079	2.886322	5.795186	0.3334287	1.547708
0.0843209	0.01286601	0.03186741	0.8615608	0.06443563	0.04793049	0.004991953	0.1426884
3.657604	2.137798	1.890884	5.322153	0.9513274	3.138652	1.965788	1.911772
0	0.02821904	0	0.04183756	0.1009477	0	0	0.1203688
3.754123	6.943255	6.943215	4.588868	6.41815	18.83053	7.53588	12.43896
3.452194	2.263778	1.818512	7.088626	1.622374	11.3015	2.056374	2.492339
0.007282295	0	0	0.02855506	0.006359911	0.002759648	0.01034701	0.02654217
10.62107	11.02502	5.972379	9.131503	11.13798	21.29074	7.678823	10.85086
4.671087	4.078703	4.164667	10.387	14.17383	14.21266	1.970074	9.728551
1.639427	0.07941271	0.4248608	9.623062	1.238599	0.7691861	0.5176378	1.449791
0.9536534	0.3858523	0.2008601	2.7569	0.3322092	1.372469	0.8576548	0.8591944
0.01238861	0	0.0149825	0.2653093	0.3354032	0.01877881	0	0.3289751
0.2530886	0.1875694	0.08198554	1.281397	0.5091633	0.4144626	0.3810043	0.4047466
0.1351588	0.02138687	0.03632398	0.4001271	0.07213523	0.2257423	0.1043178	0.06516147
7.195111	4.154057	7.447313	9.306639	2.915443	6.484908	5.679519	10.26026
1.919979	2.042567	2.475163	10.44827	3.819361	2.59201	3.018472	3.977945
1.03853	2.510496	1.606988	6.449659	4.208191	5.281188	1.550766	2.122822
0.1178774	0.1792308	0.2125867	0.0785955	0.1282321	0.161439	0.2311495	0.2476588
0.01595118	0.006490379	0.03215163	0.141132	0.1625258	0.1088054	0.05540123	0.1162762

TCGA-HT-A615-01	TCGA-FG-7634-01	TCGA-41-2572-01	TCGA-DU-6542-01	TCGA-HW-A5KM-01	TCGA-14-1402-02	TCGA-P5-A731-01	TCGA-HT-A4DS-01
0.1180789	0.1751011	0.04719317	0	0.04952337	3.542458	0.648779	0.06058786
4.727217	10.50653	4.393085	4.487826	9.852596	22.15111	2.878885	1.014125
1.095843	1.510456	5.596046	0.8943765	3.275579	5.545056	0.9649127	2.954193
7.766968	5.333508	1.137641	7.270178	12.22502	2.939946	1.198894	1.234095
1.2796	1.353578	1.222303	0.5888629	0.4025069	1.281515	0.996016	0.6894087
3.15733	3.222648	5.630247	2.634172	5.270086	2.854118	2.153096	8.758709
1.784019	3.30304	1.454072	2.416069	7.212594	0.7144096	0.9115349	2.400912
50.37093	134.0114	128.5003	151.1792	232.1224	82.9743	30.15111	166.1103
2.257275	5.931646	5.232622	4.598193	13.26327	4.234106	2.129317	6.590747
6.306352	12.92974	17.82198	10.78148	15.59708	5.54843	4.8122	4.342492
13.41607	11.19932	28.87359	13.14195	47.38603	19.7586	6.768513	51.65309
12.338	11.83755	9.217581	13.3808	45.82274	5.381276	7.074532	12.37546
0.791074	1.032222	2.191116	0.3874951	0.6541499	2.7479	0.9047339	0.9890747
12.01644	11.80673	8.200099	12.055	32.06976	11.10195	4.451648	15.03222
213.1415	1155.338	718.6785	281.0267	355.6011	1366.711	490.5308	398.9119
1.305211	1.622665	0.6475778	1.402377	5.38333	0.4616673	0.4842768	0.7707565
6.029132	8.434757	6.181672	7.727139	32.14415	8.464233	2.454171	3.638269
4.690057	6.680164	5.004539	5.651322	25.6087	4.023545	2.210067	6.071792
0.4226087	0.4891263	0.3460515	0.4348165	0.5511916	2.100971	0.235976	0.06346716
0.08287923	0	0.03312475	0.02648642	0.03476031	0.2925225	0	0
10.07546	8.375008	5.399955	10.1528	27.93467	5.541915	3.212375	7.866428
1.465346	1.017352	1.349685	0.8365416	1.986209	1.384082	0.5733483	1.240616
0.242874	0.282289	0.2282472	0.1950922	0.5285895	0.4691567	0.1202216	0.5052239
60.32167	50.13638	59.39123	48.43214	185.3253	47.43156	21.64026	39.7049
7.650616	7.769397	12.38152	7.557652	9.716933	17.09239	5.634138	14.62555
15.8019	20.66169	17.23489	24.0764	80.88258	9.321943	8.232344	23.37331
7.63721	7.450591	5.988435	7.479528	43.40645	5.157918	3.069578	2.608672
0.3147266	0.2666932	0.2587646	0.5028993	0.2432557	0.5633487	0.244291	0.359893
2.122001	3.670096	1.423101	2.913728	7.636537	2.731247	0.8110413	0.8304612
0.9322678	7.768907	4.884353	0.8355069	5.954275	27.73628	5.679073	1.466274
0.2348504	0.2956189	0.2292023	0.1204341	0.8040218	0.3108394	0.04001161	0.1261097
2.929296	1.500882	1.686137	1.576821	5.054091	3.902871	1.122899	2.23212
0.02395804	0.05329163	0.02872628	0	0.01507233	0.09512996	0	0
2.750926	24.66866	15.30471	14.98802	37.11987	18.23633	4.858339	9.183055
2.408935	2.544384	3.121934	1.814632	4.946801	1.250429	1.861096	3.6482
0.02641459	0.04476642	0.02262268	0.003617801	0.009495875	0	0.00691113	0
4.919874	6.499718	25.72086	7.345106	16.25615	15.07915	8.69568	20.90696
3.215484	34.63562	31.01303	3.828091	39.79121	153.5397	17.73204	7.071034
3.263205	2.249563	3.637807	1.357429	1.73905	8.049156	0.8643712	6.725252
1.188206	1.181535	0.4593623	0.7878411	9.763131	0.7642866	0.4118474	1.324782
0.1348092	0.6568496	0.5080101	0.3323478	0.04846305	1.384946	0.08230033	0.02964532
0.9414304	0.8543066	0.1403978	0.7094928	2.425052	0.5331328	0.231611	0.2883943
0.2101083	0.1115508	0.1368482	0.08206746	0.3639086	0.861741	0.1211439	0.267527
9.169693	8.168691	21.50609	5.635454	27.42812	40.27774	5.16743	14.21868
2.521764	4.938274	5.865067	2.993979	6.185085	5.651255	2.682568	2.903413
0.8906365	3.988353	7.875374	2.744147	2.844432	2.836618	2.070187	7.213084
0.1628834	0.3273536	0.2261383	0.3945148	0.842699	0.1843832	0.07261699	0.03958942
0.01653105	0.3145983	0.2907099	0.0792445	0.5269287	0.4375978	0.1412896	0.0084823

TCGA-06-2563-01	TCGA-06-0219-01	TCGA-FG-A4MX-01	TCGA-HT-7690-01	TCGA-VM-A8CF-01	TCGA-E1-A7YY-01	TCGA-14-0871-01	TCGA-HT-8105-01
0.3359968	1.893332	0.07312531	0.05566212	0	0	0.2479963	3.546073
16.53884	29.21975	0.5368326	5.418443	4.985453	0.7585847	3.568391	15.51095
5.519903	5.854078	0.4802729	1.43251	1.250828	0.3306046	2.390074	1.109463
2.017042	2.484493	0.7925602	2.615979	5.80319	0.04120915	1.85371	0.8758504
0.6463018	1.951461	5.245991	0.7147925	0.9690155	0.4813711	1.048121	2.716703
4.272308	3.798752	2.246525	4.19907	4.568167	1.919545	7.44953	1.457809
1.424954	0.861733	0.6055937	2.277592	2.824601	0.8135566	0.9496079	1.013227
71.59454	59.16768	21.55477	166.8058	112.7511	31.54816	16.85988	9.300173
3.019911	4.327735	2.23666	7.3936	7.242923	1.697935	1.712828	1.650517
8.062877	5.450646	15.1902	10.93219	7.661574	3.917874	3.579176	4.773117
16.31847	36.95366	9.320221	21.61829	53.53142	4.919466	6.84978	4.753283
6.637655	2.939865	5.904121	16.12763	19.51258	4.2682	3.172324	6.48071
7.313368	1.709723	0.4492585	1.248191	0.2704381	0.7023011	2.633668	0.9769843
9.522449	24.55016	3.674374	11.97963	20.13216	3.603525	2.161297	2.653726
736.7912	5176.191	50.41273	276.9196	343.6017	173.4815	280.2809	128.7041
0.4742571	0.8359312	0.5957776	1.623048	1.571738	0.3917598	0.5671615	0.5288989
10.7671	8.749454	1.439215	7.374903	4.189745	4.543413	1.931604	3.664564
3.14967	3.386354	4.508418	7.743287	10.87693	2.482608	2.140571	1.790496
0.2749721	1.928216	0.03830021	0.6413807	0.2373351	0.1650033	0.2597817	0.0888272
0.0294794	0.6792277	0	0.03906908	0.1696292	0.04422445	0.3481357	0.04328657
5.277117	7.971996	4.005952	12.77529	14.9094	3.150821	1.371038	3.419779
1.705779	3.218245	0.4207393	0.8242031	2.617421	0.30921	0.3777062	1.106247
0.3432176	0.4069776	0.1097586	0.3527538	0.8363221	0.02101587	0.1654373	0.3548356
32.49527	57.21306	25.10532	92.28541	98.02169	25.55116	15.1212	16.24376
11.50621	20.65539	10.86507	10.84777	8.263774	2.327596	8.480011	6.464412
10.78988	14.14693	3.477481	26.05658	31.63271	6.703274	3.912263	4.67879
6.24553	7.557397	1.733747	7.073635	4.652659	2.115292	2.257847	1.427078
0.3070503	0.5190657	0.3007147	0.4026952	0.4647096	0.7605214	0.1133156	0.3264057
2.3171	2.772953	0.5763274	2.613088	0.683211	0.7448724	0.6231897	0.6938547
4.044069	22.9209	1.581426	3.367669	1.306617	0.2595439	3.703181	22.9112
0.2272906	0.1634722	0	0.2857809	0.03353505	0.01311451	0.1032377	0.2182186
2.28981	4.442094	0.5062575	1.868294	2.210928	1.273569	0.7971393	0.8348523
0.02556498	0.07683085	0.08902212	0.06776258	0.03677624	0	0.3019085	0.01876938
6.952796	22.66814	2.131966	22.28053	7.902568	3.963322	2.079956	2.703175
5.844272	1.655399	1.483792	3.663808	3.363884	1.514948	6.995864	1.114023
0.01207984	0.0080675	0.04206431	0.08805185	0.005792445	0	0	0.01773764
7.496629	9.961222	24.45862	11.50518	22.67242	5.489665	10.37679	4.107511
19.83284	151.6539	1.969441	11.49322	6.942743	1.357545	18.92422	74.35493
3.654743	4.324276	0.3507307	3.890164	3.642988	0.2266503	1.954119	0.9507587
1.730036	1.151444	0.08252486	1.966956	0.5966124	0.2088738	0.2448896	0.1130968
0.1918017	0.102933	0.1669725	1.348141	0	0.01027634	0.161791	0.06537963
0.3598477	0.3554787	0.01740359	0.9637488	0.3594829	0.2174343	0.05902232	0.3229041
0.2158972	0.3909674	0.0867456	0.3521585	0.1154709	0.04775228	0.0653751	0.04064312
14.26492	31.16556	3.307384	9.523908	18.04581	2.903514	4.867591	4.131336
2.705651	4.549414	1.052549	1.866181	5.366341	1.874646	2.263649	2.522297
2.472477	4.428705	6.14288	2.650334	5.017698	0.9912424	1.095688	1.215077
0.1738085	0.2176457	0.07167249	0.2273177	0.03618863	0.1080717	0.05401538	0.1175331
0.1587585	0.1826013	0	0.2883298	0.02537561	0.03528394	0.1735974	0.2806023

TCGA-DB-A64L-01	TCGA-DU-5852-01	TCGA-28-1747-01	TCGA-HT-A61B-01	TCGA-DU-A7TG-01	TCGA-WY-A858-01	TCGA-08-0386-01	TCGA-S9-A6WG-01
2.00824	0.06260463	0	0.123967	4.081157	0.0457327	1.084205	3.793304
6.888448	13.99015	11.80353	7.086453	1.570363	2.16214	5.082251	11.80267
0.713783	2.927387	4.144254	2.424874	0.5726545	1.423462	1.91208	3.01338
0.5988467	3.74948	3.930884	8.980513	0.1139578	4.384103	1.455747	1.666551
1.90773	0.1797853	0.2915746	1.29415	0.9201201	0.8772068	2.014793	2.309009
1.101199	2.500307	4.132962	7.623978	1.633297	3.192429	3.207485	10.66872
1.385	2.687102	3.822637	4.014582	1.106982	3.200959	1.534036	2.098618
14.91239	129.6642	234.1312	177.339	37.07336	117.3639	46.33945	56.92496
2.656005	4.258262	9.779215	11.06943	2.79442	10.23726	2.580274	6.395484
3.605975	7.886776	5.57245	16.83408	3.791316	23.35617	10.51821	14.47532
5.703118	19.05469	21.09191	35.03733	7.875377	28.65044	21.91599	19.53362
7.305306	14.3417	9.218393	30.28866	6.226455	21.41871	5.89334	19.17729
0.7725305	0.8599067	1.044762	2.658394	1.045751	0.7987493	0.9304267	9.778827
3.479431	12.51474	27.81192	30.1549	3.366386	14.93985	4.904891	10.57815
63.56788	623.7835	3498.228	1349.168	122.7947	415.6335	499.6016	186.9339
0.6107443	1.078289	1.287768	2.415738	0.5229983	0.8759377	0.459176	0.778691
4.022298	8.090259	13.84133	17.80921	3.893389	6.120595	3.749839	4.988457
2.687042	4.659811	8.915691	7.124182	2.418888	10.81691	3.164439	6.60897
0.1566568	0.4098736	0.1708155	0.6168268	0.03685435	0.08383558	0.2103203	0.5072645
0.02999102	0	0.2575253	0.02900403	0	0.09629894	0.0281852	0.02832456
3.55766	10.43041	12.50497	18.71897	5.831204	15.07029	3.84799	9.714867
0.7953842	1.663307	1.897156	1.67128	0.2540273	1.509134	0.4960631	1.372626
0.2565362	0.1618328	0.305946	0.2618767	0.2425236	0.3279621	0.2209988	0.2422817
23.75289	58.69409	51.46027	266.9833	42.32408	109.0397	38.06104	57.65649
5.844254	8.919622	8.952975	12.27947	3.628187	7.707245	10.96837	9.297757
3.70572	23.71716	36.82517	38.47037	8.726585	24.07641	12.07278	19.77178
1.629003	6.180665	14.5523	15.66772	2.304474	10.40917	3.823485	4.416454
0.2961095	0.2812882	0.3973809	0.6199323	0.3429481	0.4771342	0.256874	0.5654585
0.5563847	2.846046	3.417846	4.337634	0.6536968	1.64547	2.192442	1.428907
20.63728	2.192033	2.896775	8.000268	38.18807	1.106768	6.182308	32.02021
0.1482278	0.1129332	0.2262745	0.2293595	0.05858391	0.02538391	0.07243739	0.2351857
1.014226	2.852067	5.110432	5.491729	1.287457	1.469995	1.146768	2.948445
0.1300433	0.03810716	0.04962884	0.1006109	0.08566147	0	0.02444263	0.09825394
4.675824	9.845024	15.89886	45.76406	8.084155	18.96807	7.377263	16.54392
1.695247	2.922759	3.739201	3.9197	2.15134	3.681686	1.589861	4.397063
0	0.009003108	0	0.1980841	0	0	0.02309903	0.003868874
9.686179	7.518732	11.56257	10.23193	13.88887	19.29317	11.11284	39.28495
72.23075	13.82597	11.67478	39.47121	96.8182	5.583669	32.15633	91.22198
3.820642	2.080444	6.550203	2.052725	0.6910516	1.770448	0.2751412	6.940192
0.2411043	1.713307	3.243472	2.68728	0.5095442	1.458016	0.5891259	0.8368242
0.06968946	0.04084282	0.03324476	0.0471772	0	0.193932	0.3536639	0.1777063
0.3762622	0.7524348	0.4560084	1.003128	0.2177057	0.3755061	0.2484804	0.3169384
0.07321472	0.08045411	0.1880655	0.1225475	0.09274543	0.1145296	0.1190878	0.2127585
4.156844	5.983641	10.40198	37.13251	3.442735	13.19121	4.420572	8.60936
3.81765	4.428657	3.649511	3.104482	2.602026	5.434706	5.434538	7.352715
1.34188	3.504192	8.192723	11.61286	2.215393	9.39827	3.011255	4.570538
0.07677928	0.2812371	0.2264208	0.2160073	0.04086922	0.2116694	0.1180738	0.1977628
0.4187395	0.03067627	0.09131707	0.4454548	0.2758299	0.1600644	0.2192504	0.3841729

TCGA-DU-5870-02	TCGA-E1-A7YJ-01	TCGA-DH-A66G-01	TCGA-76-4931-01	TCGA-FG-A70Z-01	TCGA-TQ-A7RH-01	TCGA-VW-A7QS-01	TCGA-76-4926-01
0.1448553	0.8442684	0.1974269	0.1159553	0.2076688	0.2032425	0.1439414	0.20486
5.593597	5.100071	3.188601	6.083663	7.927675	2.198299	1.585069	11.49004
0.6928537	5.740874	0.7498086	2.872449	1.522771	0.9068324	0.3981895	3.968193
1.103058	3.532861	2.766968	1.343441	1.962515	4.114463	0.4438192	1.237782
1.145932	1.022748	1.168156	1.084853	0.3086358	2.085036	2.491887	1.039714
1.860423	19.32429	3.073068	5.356687	3.336797	3.451506	2.616413	2.65148
1.080313	2.89986	2.289011	1.273508	5.233454	2.615249	0.4454214	1.158408
5.74836	95.7862	97.84144	68.38071	180.7415	52.63981	1.083328	63.81956
0.929005	11.75141	7.538548	3.146253	5.674996	4.390828	0.9675258	4.37524
8.252577	8.148769	8.745056	7.285913	10.26725	18.65694	11.68634	3.654845
6.018224	19.29922	9.230545	10.83684	22.94366	16.69462	4.975217	13.4662
5.603717	15.77242	29.94151	6.124506	24.66307	15.85997	3.494457	6.109879
0.4036533	1.675162	1.292634	3.324496	0.619699	1.215954	1.294911	1.375464
1.099685	12.62574	10.09767	6.237015	11.1176	10.86745	1.205452	5.444425
51.34613	539.497	125.5052	1113.696	521.0447	103.4577	10.74574	936.6089
0.3468092	0.6637199	0.5756759	0.5745715	0.966829	0.9247795	0.4269188	0.5173129
1.140387	5.356156	5.291104	4.920427	6.258806	4.757583	0.7413969	4.715379
1.12447	16.56537	11.99055	3.136906	10.3758	7.556316	1.899942	3.015962
0.04741849	0.2289939	0.7858748	0.1923208	0.2719223	0.2749973	0.4806171	0.2861273
0.02541837	0.02116391	0	0.0542591	0.04164632	0	0.025258	0.0479302
2.408546	6.132414	6.290817	5.173759	7.365661	10.40198	1.700063	4.490201
0.3799552	1.46954	0.8218789	0.6867915	1.079389	1.186588	0.1400619	0.7395751
0.3019761	0.3922342	0.0790216	0.3545359	0.3958144	0.434993	0.04200993	0.2220745
27.05398	27.78453	52.21805	50.46804	70.03372	69.87939	14.7957	40.29575
8.926072	8.855862	6.308817	9.550582	8.049064	7.149637	7.243757	8.269796
4.349536	22.06923	11.09486	12.08896	21.87443	17.29619	2.191214	10.53837
0.8448652	4.140656	5.235058	5.21984	3.781382	6.624147	7.023427	3.782046
0.1158288	0.3811744	0.7186664	0.25314	0.5761115	0.3379307	0.1096172	0.2314137
0.3185059	1.59806	1.41618	1.262664	1.728208	1.632782	0.275393	3.958443
0.8764031	7.825009	7.766609	5.891049	11.09026	2.372105	5.78112	6.276331
0.07035161	0.1506249	0.1205399	0.1072682	0.1728997	0.07520644	0.01498024	0.07580496
0.4790422	3.179708	5.799205	1.61033	1.712797	3.433941	0.2580295	1.772845
0.04408638	0	0.02403458	0.02352715	0.03611632	0.04123761	0.06571236	0.0207829
0.3832745	13.27672	23.36295	8.737263	20.42087	5.661722	0.5820636	7.53404
1.015601	9.928489	1.924837	1.527126	4.348413	2.260382	1.092304	2.306534
0.006943829	0.002890795	0.01892784	0.003705646	0.06541779	0	0.01380004	0.02291388
5.715858	29.84852	9.076469	13.91072	12.94766	7.831382	16.68678	7.613404
5.494332	20.65385	25.9124	28.48828	20.58765	6.690212	14.21452	25.44122
1.091779	4.142332	1.515069	5.256996	1.900609	0.998021	0.1849247	1.076147
0.1123891	1.016594	0.8745065	1.134122	1.397806	0.6642096	0.1015273	1.17523
0.23035	0.1278631	0.2704798	0.05043226	0.7596651	0.03314849	0.7747272	0.1893364
0.112044	0.4592758	0.5450488	0.2437733	0.624867	0.2700717	0.06423296	0.3981741
0.06921169	0.2921104	0.1118953	0.1604785	0.1603224	0.2232392	0.0403164	0.3195225
7.447875	8.362708	6.537009	5.640354	7.240697	7.833821	11.23952	7.954439
1.004366	12.21042	18.60978	3.751931	11.87843	3.603267	1.004346	6.724489
0.3113486	18.79281	7.244511	3.433858	5.521032	1.533002	0.7842033	3.41453
0.07493248	0.08866017	0.3203574	0.1641633	0.09046353	0.3578304	0.02155413	0.100395
0.03548954	0.1561897	0.5362114	0.2326835	0.1702884	0.0948465	0.03526563	0.1720824

TCGA-32-2634-01	TCGA-16-0846-01	TCGA-HT-A74K-01	TCGA-76-4925-01	TCGA-DH-5142-01	TCGA-28-5216-01	TCGA-15-0742-01	TCGA-06-2559-01	TCGA-41-2571-01
0.1665688	0.1330552	0	0.9323208	0.03394354	0.6923563	1.047137	0.8169334	0.3242813
11.12283	8.218094	0.5210682	4.018652	6.449004	15.11762	7.379822	12.14126	8.978401
2.05718	2.919268	0.7470663	4.043376	1.79317	2.531356	1.220986	2.68273	0.6515122
3.679152	5.039073	1.810985	1.760882	3.59013	3.465528	0.8153212	3.72319	2.70094
3.101121	0.5959833	1.636963	0.5953785	0.6547553	3.805063	1.139492	1.603369	2.600492
4.110864	8.53868	4.007002	3.847398	2.601205	3.907992	4.639288	2.471018	3.257558
1.572281	2.547423	1.470741	1.257199	2.895699	1.801174	1.196668	1.236705	2.046144
78.65729	113.4006	46.06123	50.05173	313.6964	44.04876	38.53898	62.91226	57.91739
3.176023	10.23993	1.992084	1.946553	9.980307	4.787973	2.534508	8.250725	3.548101
7.638965	6.91092	13.44249	7.006363	11.86354	7.734996	10.07298	11.81471	7.039489
12.2857	23.30954	23.3577	7.259855	23.36511	15.0025	12.04428	15.624	13.19575
6.744985	12.13984	8.993009	4.579908	27.82614	13.37998	5.845217	9.931932	8.882518
1.479465	1.04322	0.1757042	1.914164	1.072483	2.803914	1.688734	2.386006	1.118117
6.074262	19.50149	3.364247	4.992107	16.92178	10.10815	4.402503	9.770915	11.58206
665.2946	1297.255	469.437	1066.585	50.83166	1511.337	313.6347	1349.058	2513.059
0.504744	0.9635977	0.5202719	0.5044926	2.217251	0.7280822	0.2681651	0.7849483	0.5164878
7.374133	9.386547	1.42896	3.856733	11.82607	5.56247	3.814897	6.26794	2.661571
6.938462	8.372332	6.089715	2.265634	6.890797	4.312956	3.15461	5.60647	8.002401
0.1570362	0.6155877	0.09532181	0.2354369	0.8800267	0.725259	0.1942427	0.4397637	0.3518239
0.04676567	0.1556517	0.03193539	0.02337119	0	0.06942326	0.03062426	0.03185573	0.2601281
4.944789	8.626469	4.687747	4.551848	10.79304	7.365354	3.662016	3.535553	5.192067
0.9865707	1.786298	0.5312688	0.5409349	0.9592683	1.046112	0.6718929	1.35174	1.034819
0.1944554	0.9541762	0.106232	0.1166151	0.2434187	0.2886675	0.2328469	0.4919895	0.2704084
55.34717	88.69307	86.4005	60.8982	154.87	99.8065	38.91012	44.13957	55.76779
12.74068	11.07636	12.59446	8.363046	8.325038	11.37423	13.83572	9.228298	10.62649
14.33432	25.47881	9.362139	10.3787	41.74902	18.49397	7.724207	12.85294	17.08056
2.792891	10.82673	1.777764	4.073569	15.82512	7.935605	2.275794	5.500764	4.999703
0.2536979	0.3445116	0.1385964	0.2155358	0.2920981	0.2673949	0.1860687	0.3248891	0.6067998
2.895749	2.244227	0.3533948	1.163808	6.447657	2.067451	1.086429	3.644366	0.9947964
2.075587	7.627616	2.553625	15.01908	17.53032	15.02399	6.986898	5.44505	5.772594
0.06009507	0.1969393	0.01894052	0.05082435	0.5652105	0.1578342	0.0181629	0.3904609	0.03214146
1.363225	4.18909	0.3881179	0.7409614	2.929061	1.669091	1.278369	2.766157	1.122524
0.06083383	0.02699671	0.1107794	0.06080353	0	0.09030734	0.1062313	0.0828773	0
7.623741	10.24716	5.151594	7.127905	27.71623	15.45524	9.453263	18.09889	12.08167
2.415365	3.808302	2.323055	1.647892	4.81006	6.885638	2.50839	2.317261	2.418787
0.009581638	0.004252119	0	0.01915373	0.1952555	0.02370644	0.004182992	0.0174048	0.01332416
17.90938	23.6202	10.45874	14.24983	5.95081	15.75535	15.11335	12.75236	17.04068
13.39848	36.31277	16.25683	48.57521	113.2671	49.41408	41.08863	42.21121	27.28234
1.164131	11.33515	1.558751	1.562809	3.872393	1.897569	1.569493	1.539314	1.618833
0.8036129	2.577715	0.1668781	0.6716925	3.725322	1.765018	0.5231644	1.075599	0.888771
0.2879711	0.1519075	0.5565566	0.3475654	0.1107226	0.3468321	0.5692873	0.36271	0.02266702
0.1942499	0.3852783	0.08662832	0.166417	1.547024	0.5178755	0.1505675	0.7020994	0.2315337
0.08123303	0.4910515	0.03898068	0.05705424	0.279624	0.1368857	0.06038356	0.1525427	0.04579545
8.471905	16.88055	9.007989	7.444743	16.27926	17.38761	4.356314	8.404559	8.104361
6.47924	6.041334	2.140396	3.378281	2.12709	10.2434	6.770255	9.424524	8.180548
3.88252	8.566335	2.330753	3.785162	3.554651	5.208473	3.323939	8.79575	9.401242
0.1251656	0.1328267	0.03220731	0.08884146	0.2587602	0.1400287	0.2209467	0.2471305	0.1185589
0.1865571	0.1179756	0.01273963	0.1305249	0.3611593	0.2284776	0.2687651	0.4257131	0.1102556

TCGA-DB-A64S-01	TCGA-32-1982-01	TCGA-19-1390-01	TCGA-DU-8165-01	TCGA-27-1831-01	TCGA-VM-A8CD-01	TCGA-DU-7006-01	TCGA-P5-A5EX-01
0.2165649	1.757794	0.4906209	0.1602134	0.1663339	0.2045134	0.2192256	0.5124045
5.278341	7.373966	6.771347	46.36462	17.22568	4.67575	83.92991	15.34774
1.419264	5.118098	0.6854898	2.081626	9.087905	2.600893	3.004865	2.158229
0.9388856	3.589781	3.339832	7.02815	1.181138	1.670635	4.490942	7.571802
1.215681	1.292604	5.396522	1.085127	0.4205904	0.2469563	0.317751	1.183181
4.479833	3.707295	2.739394	3.247159	2.64965	8.751285	3.367479	6.942571
3.59086	2.224968	2.084612	2.835546	0.7060349	1.821178	3.733562	3.18352
144.4824	199.3636	35.32288	180.1791	144.8086	105.7035	166.735	183.8346
11.1967	10.54551	3.284823	10.4825	7.740808	7.282331	11.31533	4.564736
15.30564	20.38131	8.508991	19.65617	16.03922	8.220208	14.51393	12.22187
19.54222	43.01019	6.662038	14.4807	61.78674	17.71267	14.46125	22.80758
26.47535	15.31839	9.510306	14.14762	3.195383	7.145043	19.80722	16.93675
0.6614514	2.534535	1.254899	1.652218	7.630202	2.797557	2.145343	2.016827
16.39064	19.28583	6.213241	10.33019	16.70067	13.57842	14.45234	8.721922
836.42	596.358	1062.211	299.9155	1576.103	2822.994	1340.971	129.4712
1.176287	0.9511684	0.6511828	0.9274606	0.9430787	0.6180547	1.167237	1.064812
6.787065	8.717352	3.108164	8.906221	8.302655	7.702796	9.207724	9.045034
16.09031	12.77967	5.660773	4.714352	2.548221	4.896194	6.78564	4.241648
1.474569	1.939972	0.0734195	0.734244	1.873064	0.2677905	0.4162294	0.3922443
0.121605	0	0.02459752	0.05622666	0.03891641	0.1025338	0	0.02766578
13.22803	10.85375	3.65173	9.199551	5.238763	6.294408	11.60104	11.6746
1.34133	2.337217	0.628623	1.050599	2.111098	0.7910611	1.228892	1.327361
0.16614	0.6281879	0.1519566	0.6145469	0.7767245	0.5700826	0.6078288	0.2958083
161.9303	89.52261	38.5871	94.71747	101.1911	51.04311	83.35528	115.8571
9.626818	14.02989	9.893191	12.50565	15.91184	7.284533	13.59806	10.99087
24.65432	26.08425	12.57915	24.43197	11.12558	19.67842	26.06115	18.35068
6.868035	6.76342	3.762205	7.931356	5.531631	7.4811	9.64689	6.810767
0.8608982	0.9466039	0.3389338	0.3080728	0.7937987	0.1802195	0.2545976	0.3331852
2.681451	3.700089	1.296927	7.84613	2.19123	1.741994	6.498021	3.19204
3.612975	26.82995	3.735254	1.711783	68.60329	5.084779	2.553946	5.215963
0.1021736	0.3658736	0.02917702	1.645138	0.453924	0.06081163	1.308072	0.6891471
4.771024	5.960558	1.481698	5.134916	4.715889	2.07686	5.03765	2.631353
0.02636443	0	0.04266268	0.0243803	0.0337489	0	0	0.04798437
35.97219	47.53769	7.159017	8.886132	17.41598	12.64861	8.607753	7.918216
2.733063	5.68568	1.335457	3.016336	1.861332	10.83719	5.004366	3.641311
0	0	0.00671959	0.1420808	0.01063125	0.01120414	0.02627218	0.05290448
16.91886	20.51569	9.243538	8.644849	21.36404	12.42596	6.574919	20.43823
17.26454	86.00396	15.85632	10.10414	194.5216	17.46835	7.183533	23.31895
1.469032	11.82907	0.5642789	9.042786	5.698475	1.651526	5.614104	11.53202
1.020379	1.275262	0.6723323	4.22072	1.384394	2.052485	3.742	3.202721
0.5439489	0.4300397	0.3429403	0.4311537	2.007528	0.03335577	0.0983271	0.6107205
0.9586767	0.8366987	0.3753199	2.130529	0.771945	0.2572743	1.884821	1.26172
0.1712682	0.7447143	0.06235754	0.3880283	0.668679	0.4370757	0.4551023	0.7143475
13.71302	23.14381	1.854175	8.750239	79.11098	5.983784	22.42647	48.90746
12.41552	12.27597	5.173382	9.364911	5.450144	2.179581	9.264418	5.334387
10.55346	12.60137	8.953039	2.48943	4.333205	4.556244	4.198249	2.706311
0.2594317	0.2871452	0.2976835	0.7371703	0.1086861	0.1002251	0.4386932	0.1051666
0.3759567	0.4570265	0.1962483	0.1962614	0.3104899	0.1595204	0.08056541	0.1214005

TCGA-DU-A7T8-01	TCGA-DU-6403-01	TCGA-FG-A710-01	TCGA-S9-A89Z-01	TCGA-19-1787-01	TCGA-FG-A6J3-01	TCGA-HT-A74O-01	TCGA-DU-5847-01
0.7088628	0.03250529	0.09621484	0.04497767	0.1827672	0.398978	0	0.1428366
6.973297	3.560361	10.95297	6.934058	15.0812	4.569248	7.81139	6.174169
1.094911	1.881947	1.176837	1.637569	1.933655	1.417806	1.291664	3.876896
0.8670388	4.075802	7.455516	4.067981	8.094373	3.305338	8.740412	4.083828
2.604835	0.1268114	0.8932146	0.5751497	1.010114	0.6725681	1.059631	1.167113
4.624945	1.536756	5.008561	7.024075	7.810948	8.224453	9.159103	8.430155
1.523578	3.097194	2.484682	3.476542	7.388987	4.993759	2.87958	3.085752
81.36617	106.4126	101.827	147.9792	147.0667	205.3305	146.0072	120.0035
4.307738	3.936824	3.164399	7.577539	7.848878	4.680159	5.331867	9.016132
15.21975	2.962666	8.967118	43.05194	9.141869	24.33843	9.33585	27.68382
17.03853	15.89479	20.06735	28.59385	40.02399	53.33501	34.55386	22.00065
13.34442	8.409927	19.28195	16.76547	14.97781	20.31096	21.33368	32.60169
3.356764	0.7160746	0.6530393	1.204003	1.084365	0.4357676	0.3175978	3.893781
6.177766	18.12137	14.79631	12.5128	17.5559	15.80997	13.50374	15.25301
104.8172	2177.146	1060.354	512.6158	1704.937	604.6072	493.8448	1531.851
0.5710877	1.050036	1.12771	1.755097	1.097209	2.369843	1.51319	1.294406
4.034324	6.571754	8.665133	6.588353	8.256564	4.637233	5.22305	15.84341
3.174842	6.649832	6.172838	6.201488	8.268815	10.72387	11.18962	6.862549
0.3375227	0.1872752	0.2183723	1.342781	0.2967519	0.2554069	0.2318731	0.6059796
0.0452317	0.06844609	0.2025987	0.0315697	0.102627	0.06223143	0.02702045	0.1002566
9.980831	10.20502	10.65989	11.56633	9.608189	15.70007	15.14929	17.74596
0.4525682	1.325676	1.845296	1.088427	1.515517	1.380355	1.980428	1.131232
0.1988243	0.449946	0.1497639	0.9526405	0.1036348	0.155258	0.2824881	0.30015
72.38154	41.88901	126.2366	81.33452	86.62167	312.4654	101.8941	144.597
6.413873	8.878682	9.184529	8.165896	21.85115	22.34984	10.60913	14.37923
15.48481	19.67912	18.22137	27.29862	34.39277	26.60876	29.96884	49.49678
6.802096	5.46872	10.23187	5.587871	9.803605	3.178383	6.70301	12.11031
0.4490387	0.151	0.4762649	0.5822896	0.2505324	0.442253	0.2843701	0.3306787
1.604643	1.681917	2.190657	2.481399	1.86633	1.012719	1.011345	6.699015
15.72822	2.477123	2.51013	2.755978	11.80124	33.41791	4.360874	8.340352
0.1564874	0.0811892	0.1290597	0.09361814	0.2485399	0.05536313	0.2884594	1.066335
2.071374	2.166	3.029214	2.874771	2.277585	1.139949	1.389689	4.99384
0.05883842	0	0	0.05475543	0.02224992	0	0.04686507	0.06955522
13.65352	6.043151	11.36787	13.69704	14.68643	22.47651	10.07817	29.49877
2.187306	3.115518	2.655979	3.509605	3.171861	4.647528	3.537321	3.501535
0.01235647	0	0.07379498	0.03018491	0.06308057	0.00850024	0.01845372	0.06025415
13.14078	7.867941	11.38965	24.40618	19.10893	35.36082	11.86678	11.60528
52.47238	11.87688	8.637658	14.17288	31.66719	88.62996	19.97565	44.47056
1.501262	1.893132	2.010712	4.283707	5.798121	14.1395	7.029498	6.586619
1.263605	1.274753	2.36619	0.8248357	2.495748	1.069374	1.341353	4.029922
0.1261247	0.005301555	0.04184659	0.7555853	0.1788541	0.5711928	0.08162285	0.5218393
0.4294362	0.5686073	0.3015012	0.6369202	0.7264168	0.174085	0.196983	0.9008583
0.1380257	0.04498632	0.08031777	0.3883073	0.4552997	0.1928224	0.04566662	0.9300444
6.489221	5.742029	9.989753	13.16534	16.90708	28.63804	10.11838	12.28687
3.523612	1.831553	6.080022	5.779212	7.430148	5.890645	4.304464	13.3236
3.131873	4.933325	3.368192	3.875016	7.845804	11.37053	4.663494	13.35322
0.2561561	0.09203852	0.1851139	0.127354	0.1293761	0.03862235	0.07965535	0.3235528
0.496204	0.0227537	0.08531049	0.151125	0.1739944	0.2048087	0.08623174	0.7358942

TCGA-DU-7007-01	TCGA-DH-A66B-01	TCGA-06-2558-01	TCGA-26-5139-01	TCGA-06-0125-01	TCGA-14-2554-01	TCGA-02-0047-01	TCGA-DU-6396-01
0.1163115	0	2.108594	0.1704228	0.2832432	1.19517	0.4300099	0.4802535
28.23761	4.050201	29.75983	8.13228	3.742856	25.8835	11.23827	10.04609
1.403272	1.17027	2.819316	4.842207	1.71116	4.831372	3.049341	2.295072
10.72621	4.633688	4.523137	1.8073	1.892222	0.9003389	4.428474	15.25654
1.373887	0.6534106	1.442435	1.154276	3.518363	1.529937	3.282669	0.6796361
8.610593	3.962015	3.553866	5.61379	6.393316	5.666992	6.067083	2.169728
2.832765	3.097396	2.135871	3.83195	1.443995	1.413512	3.607966	11.59972
106.6843	160.4593	115.1943	175.5113	86.75602	188.8382	178.7203	281.2735
6.05721	5.71676	4.838197	6.9257	3.628699	5.983694	8.179106	17.38612
26.55398	14.62758	10.99507	7.866572	11.05631	7.778561	16.95857	26.62189
11.59849	22.22457	23.39919	16.36988	9.609786	29.22482	17.86355	30.94984
18.054	21.05493	13.71598	20.4406	6.228586	11.8516	26.767	49.49237
0.8906735	0.922782	1.337863	6.244747	1.255398	3.192782	1.308341	1.229798
7.360904	11.02783	12.77141	12.96105	5.153905	13.29967	9.271287	24.72403
430.7659	196.1316	1239.312	1362.724	429.4056	1323.928	1283.08	1374.84
0.972568	1.637493	0.9920883	1.035281	0.5819811	0.8167945	1.529768	2.002834
4.734224	6.558431	8.925499	8.278389	6.867491	9.82199	9.155499	16.3726
6.444111	5.800009	5.628487	7.168731	3.052036	5.999565	17.24053	24.81158
0.9823262	0.3947552	0.6787458	0.3124131	0.08344791	1.388902	0.513007	0.525271
0.04081933	0.02645074	0.1233346	0.02990482	0.02485094	0.0524304	0	0.05948621
10.5904	13.28313	8.007018	9.229041	3.594449	9.172845	8.908844	19.78282
0.6150904	0.8035286	2.371422	1.016608	0.5632014	1.181919	1.301752	1.826213
0.1260852	0.07541781	0.4835305	0.2486933	0.3247584	0.3550446	0.438255	0.1460534
107.0336	94.80863	63.51197	69.19325	34.77403	106.346	78.44764	143.667
9.2623	7.309694	13.70679	10.12	8.640336	13.31091	8.499421	10.19465
18.99357	24.34552	21.32571	13.46525	10.1643	20.69885	30.42978	52.29733
7.931028	7.01914	6.899062	8.0812	3.948706	6.729944	5.781821	16.2036
0.378662	0.3472505	0.4683525	0.3017476	0.2426639	0.4977497	0.6003617	1.017597
3.802949	3.396204	4.174721	2.370005	1.455876	3.297705	2.903354	7.931512
2.799857	7.664666	17.91466	19.87594	4.794651	53.34799	4.821962	4.494816
0.9966238	0.6065884	0.4327945	0.1773621	0.1326493	0.5908217	0.2651964	0.4351273
2.020294	2.208302	3.551772	1.991019	1.930295	3.227548	4.500975	2.965139
0.123897	0	0.1069576	0	0.04310222	0	0.05816559	0
14.19715	18.48215	20.31655	20.77654	7.763036	16.08008	21.89669	37.59946
2.302522	2.850387	3.119787	3.72515	2.488165	3.222416	6.534172	4.695337
0.122662	0.1083878	0.01263478	0.01225417	0.006788819	0.01432302	0.01832275	0.1760475
8.347429	11.2573	8.10859	17.17289	12.24542	24.44069	12.84289	15.71093
22.37161	25.81326	89.08854	52.93682	24.69701	195.1782	23.41976	30.48457
6.515057	2.633737	2.302614	4.554077	0.5458332	3.672313	1.685975	3.910038
1.825365	2.200857	1.54924	2.289918	0.7391937	1.870403	1.893954	3.550802
0.4268294	0.2458519	0.1934482	0.7921764	0.3637971	1.139123	0.467559	0.9445496
1.076128	0.8744602	1.014132	0.5475612	0.2317252	0.8844514	1.142807	4.282843
0.2702022	0.2086176	0.2692414	0.2470914	0.1026666	0.3938284	0.2424565	0.3518768
10.59574	17.30166	15.33261	10.43496	3.897627	33.46649	9.010923	12.5121
8.66684	4.326161	6.292198	10.14117	7.780981	10.0176	14.03112	18.67012
3.267325	4.308671	3.858109	6.7995	4.217184	5.416485	9.291031	16.27812
0.1979178	0.1969917	0.2415935	0.1832767	0.1966445	0.1138886	0.3200024	0.7568312
0.2605377	0.1793789	0.4551048	0.6561279	0.242881	0.4183096	0.7558618	0.961072

TCGA-32-5222-01	TCGA-19-2620-01	TCGA-HW-A5KK-01	TCGA-06-0158-01	TCGA-06-5858-01	TCGA-HT-8110-01	TCGA-DU-A5TU-01	TCGA-FG-5964-01
3.570359	0.5853532	0.1358019	0.7788901	0.4096457	1.756908	1.271244	0.2155446
28.92208	4.71741	2.512335	7.277508	9.612488	21.3965	2.823094	20.35985
3.452534	3.050516	1.749909	2.620986	1.850365	3.15613	1.49459	0.9822392
7.972563	0.9966114	2.226843	4.829619	6.576337	5.551441	3.459404	2.597966
2.235499	2.177889	0.3826318	2.40176	2.913767	0.6438742	1.446505	1.055011
2.339006	4.473542	3.256516	5.958013	4.067867	3.549264	5.519177	2.624224
1.692458	1.4653	3.168384	4.902466	2.921611	6.809905	2.939751	2.261706
62.19464	162.7557	219.0015	229.9946	127.821	263.4466	164.9769	37.01627
9.967793	4.050861	12.13039	6.789915	8.919601	18.57584	5.306251	3.362856
3.812828	7.89438	10.54051	7.752815	11.57107	8.751653	13.07423	14.00221
29.49938	13.719	27.2832	22.51306	15.54395	29.93915	26.29751	18.48674
11.80105	9.250079	17.39022	17.61373	12.01692	23.16387	19.98812	10.84601
0.4557952	2.009318	3.331334	1.656194	1.323736	1.289658	0.857718	0.3657417
26.03297	7.722914	21.3958	16.89782	16.20734	26.74295	14.2598	10.79147
2878.574	1290.567	1174.679	748.2696	2309.593	1586.547	433.9255	1093.246
1.139228	0.5298975	1.543173	1.07283	1.053956	1.442829	1.436617	0.5494287
10.64999	4.553777	8.149146	9.346086	7.909609	13.96519	7.808759	5.544998
7.212159	6.240839	12.28875	9.231175	7.814755	11.49437	7.681778	6.322363
0.8027847	0.2214228	0.7112779	0.5470273	0.204804	0.6859675	0.603407	0.1034861
0.2531338	0.0913017	0.04765948	0.09940011	0.104556	0.08968496	0.1673029	0.1765052
12.86905	5.197376	13.49121	10.3559	7.892172	15.3856	18.02261	9.147867
3.850991	0.8805051	2.838176	1.953159	1.62593	2.237964	1.236981	1.282729
0.8420406	0.5098021	0.4756127	0.1535165	0.670761	1.47036	0.1656331	0.4433493
57.06405	36.74496	64.85315	60.05496	54.06684	60.76546	103.0659	59.60503
11.81801	6.380643	9.798601	11.21977	10.33885	9.668057	10.69746	6.529492
24.18094	10.32469	31.68233	29.71472	28.70174	43.74442	21.41689	17.55738
6.279534	2.787995	12.62791	8.38062	11.7679	8.470352	6.917188	3.679492
0.4256979	0.4507233	0.6592941	0.4394751	0.2240454	0.5108562	0.5899383	0.2270687
3.291364	1.552652	2.675762	3.328172	3.083942	3.356808	2.708979	0.5744689
45.94181	8.77422	3.094218	4.995004	7.459284	12.71445	12.39605	3.070618
0.1801569	0.0361	0.4287055	0.2554634	0.09818409	0.2038994	0.1488382	0.02492458
4.409493	3.626333	4.696688	4.236865	2.071782	7.53495	3.369137	2.029635
0.02195214	0.0395891	0.02066551	0.1508522	0.02266814	0.05833211	0.1692689	0.02186685
24.2338	7.325113	20.02023	24.78707	14.8658	32.72377	17.8493	5.200963
4.506708	3.277833	5.17229	3.358315	7.621011	8.036301	4.551465	3.828412
0	0.006235485	0.006509845	0.01018285	0.007140696	0.01837521	0.003808673	0
16.5419	14.33045	37.0608	13.5576	14.93147	12.97695	10.40486	8.724012
141.7769	22.53807	10.05805	24.79146	31.05885	45.76917	48.53901	12.31979
2.261027	1.893967	4.3268	2.66842	1.620307	3.764633	0.9799171	3.236829
1.516073	0.5688454	1.479897	2.831807	1.875474	1.973729	0.8238028	0.572653
0.1176402	0.2439791	0.03876084	0.08084084	0.1154034	0.2969685	0.0194379	0.4921693
0.5536145	0.3250617	0.3151241	0.5729721	0.819839	1.136576	0.8083809	0.5728381
0.2543125	0.2143151	0.239407	0.3266546	0.1693449	0.3473581	0.1151964	0.08996567
25.9445	9.60202	12.02361	6.797936	8.358196	11.8854	14.13696	6.867127
14.40186	7.592025	19.07621	10.51512	6.295099	13.86663	4.323456	2.560192
6.935497	8.818664	11.94112	10.4781	8.522456	10.83099	4.613268	3.56259
0.1413909	0.1345771	0.2236884	0.1869982	0.1703363	0.3130913	0.1600748	0.0723769
0.4291643	0.2003209	0.1045675	0.1883498	0.318034	0.3756588	0.1334806	0.03520563

TCGA-06-0174-01	TCGA-14-0736-02	TCGA-HT-8018-01	TCGA-06-0157-01	TCGA-06-0686-01	TCGA-06-0743-01	TCGA-32-2616-01	TCGA-28-5208-01	TCGA-HT-7693-01
0.6538976	0.8647005	0.548772	1.022113	0.6667943	0.3320839	0.9235664	0.2290374	0.8337919
6.576601	57.91492	6.123599	28.62487	10.35317	14.34714	17.66455	32.27216	5.045908
1.9256	6.785186	1.771185	2.162009	1.933828	3.289374	2.243908	5.423015	1.182711
2.451497	4.712909	2.714831	3.183348	4.734913	3.351028	9.296614	6.769522	5.514305
1.594388	1.553958	0.9014315	1.846076	3.161344	1.390011	2.428735	0.5212279	3.134979
3.264425	6.370673	3.59366	4.018801	4.296155	7.219432	5.94791	5.205627	2.648163
2.136282	2.130361	1.908416	2.71707	1.966883	1.726257	2.960747	2.831594	1.194434
57.95041	237.0897	97.81408	210.2927	51.30393	102.0668	50.81068	164.6254	56.20455
4.198704	6.638738	7.031791	6.050908	4.163297	4.51551	3.364039	6.172177	8.083673
7.011991	11.48201	13.21474	12.58463	15.49943	11.51278	9.363616	13.99912	10.55445
17.31054	55.67247	14.1635	35.29346	21.24665	25.4929	22.78741	38.26895	15.2935
7.189683	13.72725	16.20472	11.93436	10.69629	8.884169	7.173147	9.540247	12.06687
0.6779257	2.403248	2.238358	0.960204	1.192395	2.517482	1.196964	1.537795	0.5727075
8.370027	21.63627	9.215097	10.047	8.659956	7.551853	10.12822	14.23239	9.354666
1701.813	1088.283	244.0922	604.5127	1114.103	295.2624	799.6543	1551.752	1211.982
0.7301992	1.349265	1.011454	1.017266	0.7982121	0.5399341	0.6600544	0.8948303	1.295422
4.963041	13.28899	9.402492	8.880517	4.495498	5.892744	6.396814	11.0489	5.206957
4.993557	10.58156	6.145092	5.694838	7.306383	4.211145	4.807196	5.605577	5.238133
0.2568647	2.283354	1.104319	0.505602	0.2182757	0.4674441	0.4837284	0.3898731	0.3132905
0.02868555	0.2528878	0.02027271	0.02657104	0.05850262	0.05827217	0.06482485	0.2143476	0
5.422571	13.94302	6.914501	9.132462	8.028974	8.004301	8.339638	10.16177	8.441797
1.134227	1.999838	1.339231	1.409367	0.9873392	0.9764253	1.364943	1.634339	1.116523
0.2317379	0.9013097	0.2504782	0.2272825	0.3614128	0.3530663	0.5288255	0.8021472	0.5924943
62.81387	185.602	83.28645	96.80984	77.12426	67.7566	36.63732	123.9675	65.5752
10.2564	16.58781	5.937474	9.045762	9.78858	12.38608	12.92045	14.8632	7.998362
14.7414	22.92184	14.61788	25.35925	16.23586	15.16274	27.79742	22.69436	14.47131
4.046395	9.731337	6.40962	7.862439	4.96409	3.747769	7.170564	10.9113	5.507702
0.2832202	0.773743	0.9458014	0.4122534	0.539528	0.4362446	0.1781778	0.2790741	0.5438626
1.419109	3.209974	1.794691	3.057081	1.589904	1.849162	2.665438	3.025988	2.368527
6.58667	48.42022	6.275995	7.231706	5.965522	9.896248	5.944426	16.17659	11.19987
0.09073639	1.109888	0.2685251	0.6933952	0.08096023	0.4953681	0.1238844	0.4078662	0.2012156
1.192388	4.846175	3.831368	3.074795	1.246818	3.833488	1.712676	2.770187	1.819582
0	0.1754465	0.03516159	0.0230428	0.1014687	0.1516035	0	0.02323568	0
13.68619	44.85192	11.56412	14.76377	13.69814	10.65174	4.457021	17.90598	12.23422
2.288915	8.303305	3.299577	2.604182	4.186868	2.369376	3.707404	3.520199	2.146962
0.003918182	0.04145057	0.06922663	0.1742092	0	0.03979721	0.008854477	0.2049453	0.02432891
11.64563	17.04107	17.39361	9.684409	13.7736	12.56507	17.46334	12.15124	15.45669
39.44139	210.2431	25.82567	40.06848	32.92369	32.9058	26.83614	58.05272	42.76408
1.260115	13.15799	4.116325	4.83751	1.570517	4.650327	3.005867	2.367074	2.546019
0.8128978	2.703916	1.637916	2.638087	1.140514	1.428811	1.71108	1.852426	1.012563
0.1399775	2.291756	0.09421447	0.8458726	0.5845473	0.7176504	0.08535817	1.799295	0.08868912
0.4231069	2.863993	0.6186599	1.112687	0.6942902	0.4840887	0.3187185	0.7813125	0.4443326
0.05656089	0.8168083	0.178926	0.2195457	0.1043669	0.2626251	0.08724139	0.1937105	0.2245768
4.045655	41.63075	11.75703	6.945678	6.330427	11.33571	12.50532	22.28191	8.662214
5.717532	6.615257	9.485772	4.565122	6.773973	8.321171	8.835379	6.533117	8.374257
4.997237	7.004047	3.90931	3.815204	6.07614	4.11659	5.372617	2.79419	6.971573
0.1045923	0.2001091	0.1934442	0.2288075	0.12254	0.3390481	0.1341063	0.174601	0.1855538
0.2574722	0.5548495	0.3194431	0.3391901	0.1867024	0.2498931	0.1379193	0.3313408	0.1725587

TCGA-P5-A72U-01	TCGA-02-2483-01	TCGA-06-5408-01	TCGA-28-2510-01	TCGA-HT-A61C-01	TCGA-06-0745-01	TCGA-06-0187-01	TCGA-06-0125-02	TCGA-FG-6692-01
0.7059247	3.615837	0.6155	1.068629	0.113533	2.884637	1.381338	0.156909	0.3255072
6.99349	8.027159	9.643121	10.19235	2.91161	25.72489	8.56461	6.704126	3.806039
1.397809	1.535884	4.399175	1.623421	2.239689	5.082106	4.569991	2.268326	4.132085
2.242559	9.85827	0.960736	1.533645	2.461028	4.729508	1.99128	4.280923	2.422025
0.4912092	4.666836	3.028993	1.440619	0.8981456	4.70393	3.357403	5.704826	1.164063
3.4116	2.375969	9.442209	2.414827	5.283541	4.214168	8.99258	6.280033	5.757631
2.044673	2.637081	1.724899	1.659603	3.046491	2.143683	1.690471	1.676718	1.509918
214.4924	41.72201	129.3954	61.86625	99.90699	64.90873	176.9808	351.3545	59.76211
3.551297	5.578875	4.572562	3.82213	5.946352	5.90412	6.425196	6.753888	4.664223
18.39869	10.83151	11.04413	10.32234	7.815053	20.07013	14.31144	12.25939	18.81743
27.81086	20.20858	19.21835	10.49387	17.60623	45.75119	21.15023	10.38685	8.744101
13.76229	17.86466	11.28466	9.148419	9.026082	13.23989	13.86444	10.87747	8.791665
0.8478298	0.6473954	5.246344	2.622375	1.298698	0.8439185	3.578186	1.972755	0.9765317
4.755781	12.30944	6.315643	6.525021	14.43455	12.29396	9.582362	7.574491	5.250511
149.5048	894.6438	818.1665	552.3288	1655.973	2118.284	1032.742	1360.642	157.7422
0.6744505	1.617688	0.6158397	0.6598608	0.9358116	0.9326251	0.7615656	0.8186201	0.4991045
6.988832	6.925723	6.258625	4.739855	5.822372	5.297752	8.765881	9.01164	3.957883
5.535075	7.145868	4.801287	5.256388	7.46633	8.915471	5.379716	5.566927	4.620754
0.2140577	0.6249659	0.3034119	0.3134357	0.4063386	1.142139	0.3927526	0.226003	0.2014859
0	0.02537945	0.05082561	0.06000541	0.1328141	0.07231137	0.2216131	0.0275335	0.04154046
6.485788	8.632052	6.162588	5.972324	7.563651	9.630696	9.408647	6.900294	4.109725
0.7293386	1.76837	0.6433316	0.8824971	1.165571	1.731788	0.8882803	0.657188	0.6710257
0.3965638	0.6512698	0.2837954	0.3493103	0.3155724	0.6929878	0.2961916	0.7261716	0.1036372
53.67581	122.063	72.56239	36.57069	58.87244	139.2528	105.9154	64.22368	22.12301
7.993219	12.71729	14.06046	6.307642	12.5631	17.20147	12.16186	7.137851	11.86523
17.61762	15.66853	14.65164	11.13452	19.16209	25.29975	24.20051	22.41537	6.819652
3.389674	6.92689	5.953966	4.183548	6.331074	4.32502	9.117743	12.20225	3.047725
0.1499594	0.4268091	0.2784798	0.7128929	0.3170199	0.4785818	0.3005558	0.4122496	0.1374646
0.7935949	2.601968	2.890739	1.332914	1.12822	2.584936	2.677755	2.477796	0.5272845
8.321963	20.29398	8.743469	21.17356	7.17101	22.10313	11.42089	6.524125	7.054735
0.3866676	0.1003484	0.1456964	0.2728454	0.04201095	0.20967	0.2628724	0.1905144	0.2381594
1.258552	1.604793	1.57557	1.654044	1.646869	2.610975	1.605253	1.474271	0.7243507
0.2035389	0	0.06611509	0.07805639	0	0.08361268	0.9369077	0.1671422	0.05403677
3.056763	20.42686	7.721652	11.26732	17.60826	31.3392	20.61719	20.00639	5.164629
4.06367	3.453723	2.135282	3.307196	2.381746	5.707647	2.366663	2.17434	4.696163
0.003562046	0.0207996	0.006942309	0.008196186	0.003628236	0.01975413	0.03783788	0.02632575	0.002837022
16.89414	22.66564	18.61082	11.00308	14.01099	33.57643	24.10195	19.55098	17.06816
21.31374	72.1285	60.65195	78.62903	25.00533	138.3626	64.76824	28.70117	19.44612
11.634	3.580014	1.835772	1.713369	5.756542	3.611838	4.056311	3.440376	12.37832
1.200238	0.8008213	1.567995	0.6572652	1.558873	0.9737222	1.575598	1.173143	1.014381
0.8362442	0.4776867	1.251885	0.8784293	0.0432064	0.7785312	0.9397971	1.158019	0.1206583
0.1901141	0.9939441	0.4782371	0.4120151	0.2386809	0.71514	0.3522365	0.6535173	0.07042697
0.2301653	0.1167649	0.3674575	0.1324013	0.152138	0.2919503	0.1066407	0.1835496	0.1111604
6.880271	23.01912	4.974462	7.14964	10.78669	22.44821	7.241216	9.373183	10.42939
2.282619	10.96764	8.17934	5.567388	4.390993	11.3929	8.770538	12.21524	2.991927
6.155707	4.185978	3.791569	3.708197	7.777174	8.881997	6.604886	7.688986	5.174847
0.03236958	0.08269332	0.1833472	0.1792215	0.07006352	0.1458542	0.3202068	0.1901036	0.06767519
0.0312093	0.4150981	0.4561942	0.4607929	0.1112623	0.4903884	0.9724621	0.6974607	0.07042793

TCGA-DU-A5TP-01	TCGA-E1-A7YE-01	TCGA-28-5215-01	TCGA-26-5134-01	TCGA-DB-A75K-01	TCGA-06-0238-01	TCGA-S9-A7R7-01	TCGA-HT-7471-01
0	0.1757215	0.2627605	0	0.4285331	1.019918	0.3835193	0.07753303
2.381996	1.470621	23.63984	3.411631	1.664948	76.70937	9.291212	14.81035
2.834316	2.193217	3.0787	2.005412	1.009557	3.428191	1.878893	1.524898
2.44511	7.700238	11.33022	4.115251	2.64878	9.926601	2.812959	4.324816
0.8657943	2.421586	1.231539	1.747596	3.079287	0.8243448	0.7169324	1.86527
9.872116	4.098816	3.666223	2.367648	3.240668	2.197697	4.033808	2.686296
4.573599	9.795572	3.416203	1.590465	1.67613	2.03595	5.511302	1.674284
155.7925	198.2455	177.3552	75.28364	34.73104	84.10915	402.3445	70.37532
11.6081	12.35322	7.700744	4.191724	2.455604	6.551557	14.28482	3.485497
21.93839	62.57665	17.42832	15.7283	19.12676	9.012784	22.44059	12.97265
28.00209	25.72272	17.89053	19.23816	20.57491	40.72808	25.23233	13.91975
40.41299	62.82957	17.08151	7.465345	15.09853	8.002154	19.52473	8.195587
4.865486	1.997216	2.822171	1.582778	0.8158694	1.450889	1.691429	0.8472014
26.73207	19.72898	13.71744	8.35889	7.2539	22.65557	13.65678	7.047373
3364.064	647.9508	988.4391	826.7864	822.8192	1816.438	385.6853	732.4264
2.050884	2.528436	1.309833	0.6692085	1.149668	1.135892	2.048849	0.7702171
22.68516	7.14503	9.647081	5.40243	4.229507	8.531239	8.993637	7.373335
9.558996	33.00701	9.443897	4.490161	8.990356	5.521172	5.916684	3.758951
1.323677	3.482033	1.109592	0.2724651	0.4402655	1.702742	0.326418	0.4771535
0	0.04111279	0.02305385	0	0.04627478	1.759865	0.1009467	0.0816303
35.78996	13.05105	10.68586	5.141853	7.7756	10.39435	14.44033	10.10547
1.462376	1.516566	1.306184	0.9977034	0.9650582	2.063965	0.8112668	0.8725189
0.07477868	0.2197933	0.3943946	0.4627052	0.1429363	0.9922255	0.7195618	0.1616312
286.6057	162.5895	132.8624	102.7769	88.48241	79.31066	79.30457	105.7503
11.73965	7.773079	11.15085	12.95281	8.487377	14.60917	9.240367	8.411161
48.97374	25.17872	37.28906	19.25432	16.48503	24.6236	36.88214	13.735
19.80815	4.194001	7.525669	5.525524	5.177014	8.229773	9.483958	7.235356
0.5618605	1.521076	0.5127635	0.2905173	0.3012419	0.7443462	0.6717508	0.2538916
6.467129	4.917488	4.884651	1.941041	0.7041011	2.708574	4.73659	2.152016
35.15632	15.66826	7.762732	7.232241	5.414563	21.42242	7.553539	4.471328
0.0905	0.01625569	0.2916902	0.07218536	0.02744505	0.3774034	0.5122238	0.24207
6.425142	5.655491	2.582516	1.757866	1.071797	3.535777	4.000523	1.988935
0.02481173	0	0.09996324	0	0.1003255	12.15772	0.02918082	0.02359701
73.19356	66.53667	24.11701	5.055554	15.73912	29.54899	33.66894	8.987704
7.820538	4.002821	3.928332	4.820101	2.645598	2.639485	3.993822	2.13601
0.003907973	0.01684688	0.08187254	0.01662459	0.01264142	0.03666835	0.05515353	0.09663288
25.90771	18.61426	14.49995	15.5215	17.26745	13.20119	11.99748	5.636247
138.289	60.2618	26.65585	18.52495	21.27766	100.2995	24.66072	44.51242
2.010931	0.8628791	4.287193	2.910919	0.7001815	3.974611	2.38146	1.952325
3.898644	0.5701376	2.437154	1.663382	0.5580194	1.522702	1.880049	1.585922
0.03988938	0.5254308	1.028539	0.3959437	0.01612915	5.413203	0.1329216	0.0632275
1.23691	0.6795935	1.450057	0.2888852	0.05884009	1.031633	1.74566	0.7011992
0.3760902	0.1158063	0.2078014	0.1599895	0.07820791	0.2912692	0.2337955	0.1813938
15.02732	20.36912	18.55535	8.582725	4.022704	31.62362	14.23697	13.10066
16.47826	49.20812	17.29625	7.396443	6.862561	8.697874	5.797984	1.78967
25.97975	26.03975	7.802868	5.021206	7.100971	5.008543	8.53809	2.571467
0.4372555	0.4018707	0.230713	0.292705	0.06102842	0.152725	0.5273038	0.2680851
1.420968	1.627766	0.8460888	0.121382	0.166139	0.3629208	0.3355794	0.331066

TCGA-HT-7601-01	TCGA-DU-6402-01	TCGA-06-5856-01	TCGA-DU-A7TD-01	TCGA-12-5299-01	TCGA-14-0817-01
0	0.2828054	0.8245737	1.143195	0.5347265	0.7443125
8.243062	31.69036	21.14294	13.32011	3.904635	24.17604
2.618663	5.180561	2.351951	4.87339	2.522009	7.049358
1.827114	1.585424	4.559575	6.401193	0.7794044	5.370369
0.3657236	1.284114	2.526382	0.7300427	2.047473	2.392907
3.192222	7.578066	4.286005	5.003316	4.593656	5.84365
5.98243	2.689612	1.935822	4.03931	2.30149	2.540754
387.9396	93.32608	78.95605	162.9254	117.0127	166.1155
19.07259	6.860764	6.332976	6.420267	5.825548	9.302252
19.85457	12.54905	14.88945	11.98277	9.255617	12.16842
26.11052	21.97616	17.22304	18.36451	23.71907	63.72939
29.21826	18.45363	14.04311	15.03351	15.91126	15.07225
2.200721	2.469684	1.210557	5.48974	1.539345	1.738397
21.91705	8.318225	12.99945	17.81885	12.27661	31.19477
661.1056	2170.649	1105.097	1529.889	3842.75	3534.373
2.245113	0.5295422	1.896495	1.464798	0.8305573	2.086407
16.69982	7.264262	10.99292	7.273107	8.076027	14.62493
8.690465	5.931373	6.045906	7.798765	6.006109	12.18153
0.2973537	0.2221838	0.3828026	0.8553737	0.8402072	2.696408
0.04427624	0.05955008	0.07892262	0.1146294	0.2752368	0.3482871
27.07489	8.08271	7.020029	10.17784	9.191139	19.72243
0.950066	0.8231568	1.433447	1.775669	0.9410907	4.079602
0.6627751	0.3065698	0.331292	0.4357835	0.4756185	0.8137539
175.9403	145.9883	95.22185	53.55683	138.8368	210.5282
9.385485	17.8785	10.14613	9.132762	18.74886	17.28722
36.42804	23.98807	21.22021	23.6539	21.707	27.53326
18.16855	5.085154	8.317652	10.95789	8.134186	11.80395
0.624502	0.2067531	0.302556	0.3762192	0.1981785	0.6990832
6.488339	2.144906	2.889769	3.035959	1.571738	4.789313
18.35175	10.67391	8.896917	14.96836	39.31802	36.79242
0.3282467	1.181207	0.1612279	0.1359707	0.03957329	0.2639444
3.314378	2.541779	2.905294	4.769908	1.471982	6.962667
0	0.1032854	0.1140715	0.02485207	0.7811656	0.05033997
43.2571	4.608267	8.846757	6.530625	25.57034	39.88317
4.651628	7.261677	8.891311	3.748243	2.193559	6.442951
0.04233409	0.02169064	0.003593367	0.003914328	0	0
10.60796	11.49627	9.866295	10.47725	29.56808	29.95029
56.17757	40.13964	43.67526	43.87434	144.2387	191.8489
6.180753	9.330221	1.926088	2.81149	1.795295	7.692377
3.701841	2.54129	2.194229	2.298066	1.106344	1.685806
0.2469209	0.8625386	1.222605	0.05327233	0.7151462	0.2832574
2.867489	0.6730677	0.731462	0.5975973	0.6532843	0.8217512
0.1891542	0.3783476	0.6768066	0.3175058	0.2067428	0.4114973
23.76671	21.98605	15.96057	9.477034	11.2864	39.69871
2.818031	4.715979	8.394949	5.289071	11.25725	7.918959
7.056044	7.165942	4.786608	9.56293	5.850937	7.394177
0.5616004	0.1016351	0.1612301	0.2245412	0.1785832	0.254433
0.9317038	0.07522623	0.2203861	0.1257515	1.028101	0.5846988
