# Supplementary material for: HK3 stimulates immune cell infiltration to promote glioma deterioration
Source: Cancer Cell Int. 2023 Oct 1;23:227. doi: 10.1186/s12935-023-03039-w (PMC10543879; doi:10.1186/s12935-023-03039-w)
Supplement: Supplementary file 6 — Supplementary Table S6. Genes positively correlated with HK3 in chemotaxis. [file 12935_2023_3039_MOESM6_ESM.pdf]

Table S6. Genes positively correlated with HK3 in chemotaxis.

Gene Name	TCGA-E1-5304-01	TCGA-E1-5318-01	TCGA-FG-7638-01	TCGA-DB-A4XH-01	TCGA-E1-A7YM-01	TCGA-TM-A7CF-02
RAC2	0.4886942	0.381709	0.690119	0.4079267	0.7665635	0.6435419
TYMP	0.3480567	0.3949123	0.4613476	0.6489493	1.543317	0.6155121
RNASE2	0.3325316	0.1572072	0.5359692	0.2280067	0.7510143	0.2860277
PLAUR	1.215111	0.445107	0.3549114	0.3089882	0.7143846	0.3964262
FES	0.3809218	0.9804576	1.16343	1.21887	3.371356	1.056425
CCL23	0	0	0	0	0	0.0266015
CCRL2	0.1757558	0.736732	0.4506735	0.2741611	0.4203495	0.160339
CCR1	0.8864697	0.9933893	1.31334	0.5515449	1.253718	1.222052
C5AR2	0.03427351	0.05733402	0.09464453	0.08225101	0.1186279	0.1443098
CCR2	0.004803898	0.004542168	0.02463634	0	0.02921015	0
CMTM7	0.3011933	0.9673595	0.7894832	0.4474578	1.482569	0.7536641
CCL13	0	0	0	0.0252857	0	0
FPR1	1.050813	1.627611	1.517427	0.8212394	2.345611	1.021707
NCKAP1L	0.8106724	1.648225	1.081459	0.6234491	1.409842	1.469243
XCR1	0.003319073	0	0.01021293	0.003982626	0.004036332	0.01090056
LSP1	0.6344127	0.4114141	0.5450932	0.1155817	1.696515	0.1709019
CCL5	0.3202313	0.2242846	1.240831	0.2561682	0.4038574	0.1558088
CCR5	0.05860442	0.1062053	0.2003648	0.03516034	0.2672586	0.1657378
C5AR1	0.522614	0.6952442	0.4176089	0.4739676	0.6503323	0.5521681
FPR2	0.009693488	0.00458268	0.01491364	0.01163142	0.04715308	0.00530592

TCGA-S9-A6U2-01	TCGA-HW-7491-01	TCGA-HT-7855-01	TCGA-CS-4944-01	TCGA-DU-7018-01	TCGA-HT-7608-01
0.9815943	0.7789747	0.6698353	1.247135	0.2520215	1.225912
1.310699	0.5516139	0.7471047	0.8124924	0.342972	0.9092758
0.5575019	0.091986	1.401212	0.400447	0.04610127	3.273428
0.409235	0.2929435	0.4182064	0.9835973	0.4633573	0.5082981
1.036	0.4884572	1.609081	1.018812	0.7557693	1.312423
0	0	0.04550761	0	0.03144215	0
0.438718	0.5445235	0.3401251	0.6584721	0.3524993	0.3700563
2.88433	0.656095	2.643974	2.091109	0.441825	2.310873
0.1650158	0.0755551	0.15553	0.09630469	0.03581964	0.1340897
0.07516992	0.01116251	0.02699005	0.08099048	0.009323992	0.01182235
0.7641225	0.3221595	1.052037	1.101558	0.3278668	0.7922181
0.04710581	0	0.01973244	0.07894959	0.08180132	0
2.565323	2.961105	2.757719	2.376378	0.5859222	4.230671
2.614208	1.056554	2.312191	1.594687	0.5179142	1.448047
0	0.002570773	0.00310796	0	0	0.002722736
0.3749575	0.702341	0.2146081	1.37197	0.1504261	0.948214
0.5832771	0.4776945	0.1221663	2.910509	0.3146087	0.3697194
0.447595	0.07565298	0.1783497	0.484867	0.03791553	0.5208122
1.969712	1.962753	1.05272	1.644932	0.9121313	2.333013
0.02166867	0.2552736	0.02269231	0.004539601	0.03449289	0.02783151

TCGA-P5-A733-01	TCGA-HT-7470-01	TCGA-E1-A7YS-01	TCGA-E1-5307-01	TCGA-FG-7637-01	TCGA-TQ-A7RP-01
0.5049054	0.9817054	0.2759497	0.9933182	0.8736371	1.438282
0.3172139	0.6611685	1.449984	0.693226	0.6362269	2.161224
0.476978	0.8136116	0.2313592	1.421016	0.5170353	0.7637182
0.5049899	0.522445	0.5604373	0.5761377	0.4488677	1.348184
1.457642	1.308184	0.5400656	1.124934	0.9289937	2.822047
0	0.03871411	0	0.02383196	0.04407878	0
0.2117652	0.2753495	0.2054342	0.6148037	0.3188187	0.6246025
1.925203	3.567453	0.6076249	5.036178	0.7682877	2.316919
0.1297104	0.470443	0.1206966	0.2895993	0.0382595	0.08253945
0.01929378	0.03444135	0.01871697	0	0.02178552	0.1056846
0.8496529	0.9597301	0.442395	1.852107	0.4336195	1.62216
0	0	0.2668379	0	0.05733867	0.01783064
1.096737	3.265731	1.447779	4.732619	2.489295	4.545143
1.242198	2.651905	0.6017063	3.44755	1.023858	1.739926
0	0	0.01616474	0	0.003010377	0.002808417
0.1800926	0.8890406	1.100017	0.8013788	0.4729794	1.571068
0.3810777	0.3212349	0.3812377	0.1163226	0.8605856	2.408552
0.2500822	0.2723272	0.09038255	0.2490673	0.06201279	0.3925703
1.574222	0.9100899	1.675139	3.403165	0.4795191	3.064598
0.02919879	0.02702663	0.02832586	0.009507018	0.07912736	0.02870733

TCGA-DU-7298-01	TCGA-TQ-A7RO-01	TCGA-E1-A7Z6-01	TCGA-DB-A64W-01	TCGA-HT-7603-01	TCGA-IK-7675-01
1.748574	0.8276576	1.144259	0.350517	0.4462272	0.8715219
1.058481	1.941095	1.917277	0.7338661	0.1889264	0.5667557
1.14944	0.6514322	1.374216	0.120565	0.4988288	0.6131691
0.6831389	1.012344	0.8520897	0.3676191	0.3143394	0.6301366
1.468464	1.075002	1.607855	0.8593493	1.329755	0.7539071
0	0	0.02652588	0.03083556	0.02041278	0
0.8793648	0.4121706	0.2686038	0.1858596	0.2067019	0.5545431
8.698255	1.346775	1.763354	0.2166506	0.8715531	2.05677
0.5103366	0.09745708	0.2100934	0.3713595	0.3897934	0.1179703
0.02758172	0.09165376	0.01048813	0	0.008071058	0.008267572
1.654805	0.5529845	1.456074	0.2912074	0.7229095	0.5882947
0	0	0.02300364	0	0	0
7.915836	4.410341	3.712969	0.5877776	2.584333	3.396817
4.859946	1.251233	2.362951	0.4080561	0.956823	1.989366
0.01633419	0.02374677	0	0.004211853	0	0.002856084
1.304979	0.4396408	0.4387309	0.7713402	2.137342	1.097549
0.5350474	1.088998	0.2201017	0.03010138	0.1992676	0.1735014
0.7811119	0.3552348	0.1599355	0.05577609	0.04512825	0.1302762
3.887825	1.811587	2.155958	0.1927876	0.4441284	0.9987806
0.02782772	0.0231178	0.02116335	0	0.04885826	0.02085327

TCGA-S9-A7QW-01	TCGA-DU-6399-01	TCGA-DU-A76K-01	TCGA-F6-A8O3-01	TCGA-DU-7009-01	TCGA-CS-5390-01
0.470272	2.409331	0.6045755	0.4810442	0.4608942	1.098041
0.803047	1.816872	1.031591	0.433335	0.410682	0.8817626
0.5166441	1.457738	0.4092262	0.1716224	0.6072292	0.2704615
0.5158937	0.5220927	0.9326889	0.3052583	0.2555976	0.2149791
0.5911344	1.7144	3.80183	0.6819731	1.052524	0.9156879
0	0	0	0	0	0.02128397
0.2146206	0.7653348	0.2018725	0.3005494	0.3267795	0.2873649
1.526016	9.004168	0.3599681	0.8350487	1.378584	1.351622
0.1931649	0.1779847	0.1796265	0.1466814	0.1205039	0.08082401
0.005499546	0.01684731	0.00752419	0.01735533	0.007443165	0.06732416
0.6075202	2.020359	0.4268207	0.303988	0.5037064	0.5569466
0	0	0.03300564	0.06090477	0.09795065	0.16612
3.743484	7.043113	1.007003	0.8941367	1.00687	1.955877
1.181878	5.939805	0.4466586	0.666462	1.060699	1.334165
0	0	0.002599278	0.009592811	0.002571288	0.0465151
0.08745796	0.4280875	1.20956	0.4607961	0.1724039	0.2414759
0.2036687	1.237439	1.19819	0.5227558	0.4502253	0.945363
0.1900908	1.520037	0.05736887	0.08116074	0.1248524	0.1240522
1.954888	3.116845	0.7233727	0.7113227	0.8144464	0.910198
0.01664579	0.05949149	0.007591297	0.007004048	0.003754775	0.04245289

TCGA-HT-7620-01	TCGA-F6-A8O4-01	TCGA-DU-6410-01	TCGA-HT-A616-01	TCGA-FG-6689-01	TCGA-QH-A6CS-01
0.922552	0.7576398	0.3259096	0.8152383	2.099259	1.522158
0.8569191	0.7286156	0.4454205	0.7897089	1.058092	1.627238
0.9134399	0.1219609	0.58853	0.3702316	4.920544	1.893706
0.4292606	0.4820609	0.2527978	1.775343	0.8110817	1.320341
1.249637	1.477809	0.7415907	1.451392	1.821051	2.963634
0	0	0	0	0	0
0.3415858	0.1704638	0.1503461	0.5338599	0.5741541	0.4129305
3.519065	1.264379	0.3195939	1.448682	4.975601	3.702615
0.2910842	0.1082982	0.02566336	0.1548947	0.2835854	0.237612
0.02383786	0.01233331	0	0	0.04975905	0.02220304
1.061625	0.5482444	0.1565353	0.8139886	2.197938	2.204125
0.02614181	0.03606757	0.2797172	0	0.01679025	0
3.833565	2.467076	0.4098853	8.78644	2.979985	6.278603
2.571931	1.131164	0.2755709	1.333216	4.263896	3.302921
0.01235241	0.002840413	0	0.0159185	0.0052891	0
0.2307491	1.401361	2.492528	1.035469	1.156524	1.266517
0.3825488	0.8018475	0.07346894	0.2559751	1.162358	0.575583
0.3695663	0.1546378	0.05186043	0.1815248	0.2723843	0.3667931
0.9197205	0.6864694	0.2312367	5.71247	1.956135	2.373329
0.02405047	0.04562548	0.004288998	0.03486796	0.0270323	0.02240107

TCGA-QH-A65R-01	TCGA-DH-5143-01	TCGA-HT-7606-01	TCGA-CS-5394-01	TCGA-HT-7875-01	TCGA-S9-A6WM-01
0.9024884	1.481544	0.9735138	0.2558413	0.3329143	2.015192
1.284057	0.9831587	0.9080788	0.3384989	0.438303	1.700226
0.04766336	1.258415	1.632409	0.06435002	0.3849916	1.207993
1.768207	0.742423	0.8295727	0.2434482	0.2739081	1.565141
1.520289	1.670198	0.8348701	0.6497777	0.9638646	4.613436
0.02438065	0.06658978	0	0	0	0.03340054
1.005158	0.6957012	0.4392392	0.3227311	0.1804216	0.7489096
0.5007183	7.413776	2.391242	0.4150981	1.080295	2.825091
0.132262	0.1950703	0.09883204	0.03333234	0.1645214	0.1014685
0.004819962	0.02194091	0.01715091	0.01735307	0.01167969	0.1188569
0.4940737	1.790521	0.8107587	0.2460946	0.3797058	0.9660065
0.08457306	0.03849844	0.03761712	0	0	0
1.484381	6.71915	3.733108	0.2622127	1.669608	0.8647314
0.5586703	4.303977	2.253782	0.5517465	0.9244557	1.837786
0	0	0	0.002997363	0.002689882	0
0.9831285	1.180263	1.168068	0.1139842	0.6056718	3.104598
0.4879032	0.6500431	0.3810972	0.3855894	0.1057326	1.97262
0.07350048	0.8966771	0.2048709	0.02205168	0.09103184	0.7652652
5.347269	4.107761	0.5532432	0.4664704	0.5614406	1.077532
0.004862951	0.0221366	0.02162984	0.0218848	0.01963977	0

TCGA-HT-A5RB-01	TCGA-P5-A5ET-01	TCGA-DH-5144-01	TCGA-CS-5395-01	TCGA-QH-A870-01	TCGA-S9-A7IZ-01
0.6772509	0.6010531	1.040046	1.274349	1.115278	2.018905
0.823142	0.5430923	0.6198079	1.039933	0.9402614	3.966334
0.4025013	0.6391298	0.4103462	1.130612	1.775943	0.5537363
0.5016268	0.4747062	0.4483472	0.850356	0.5652116	0.4817959
1.511297	1.208964	0.778733	1.970338	0.9877358	2.148347
0	0.02514817	0.04197987	0	0	0.02574963
0.2694675	0.2182739	0.7894574	0.3569497	0.285389	0.4097396
1.804224	1.467894	2.393636	2.725557	2.164728	4.237629
0.1595584	0.1882674	0.07287537	0.1581227	0.1941372	0.3268711
0.01744414	0.01491509	0.02489778	0.01371999	0.02721097	0.07635906
0.7927928	0.6135321	0.5740308	0.8966224	0.7853314	1.104426
0	0.0654266	0.01820279	0	0.02387275	0.04466093
1.832569	1.209148	1.039075	4.080488	2.404365	2.776493
1.438074	1.208564	1.213399	2.703588	1.612449	3.387796
0.008034914	0	0	0	0.00376008	0.007034323
0.4301864	0.1753152	0.3557753	0.9897407	0.2935035	3.78727
0.2584086	0.6505591	1.014264	0.7790902	0.3359079	0.5404356
0.1832505	0.1263572	0.3543595	0.4370344	0.08852162	0.6365459
0.6105132	0.5094229	1.091846	1.209115	1.769278	2.33757
0.01759972	0.01504812	0.004186641	0.05998354	0.0329444	0.03081604

TCGA-VV-A829-01	TCGA-P5-A730-01	TCGA-QH-A6XA-01	TCGA-14-0790-01	TCGA-DU-A76R-01	TCGA-HT-7480-01
1.499358	0.7384312	1.233926	1.537094	0.514788	0.4886216
1.760189	1.084856	1.211038	4.053676	1.11533	0.6003086
1.945627	0.1407065	0.7967857	1.217684	3.040552	0.3351809
0.6891268	0.7817939	0.8533756	1.856443	0.8031424	0.4006035
1.476833	1.519411	1.653391	3.406614	1.175436	0.7499006
0.05169988	0.02878954	0	0	0	0
0.5609124	0.3123491	0.3803774	0.8509735	0.2860003	0.2801691
1.117671	1.882716	2.64331	2.390113	1.695402	1.770885
0.1206001	0.0437303	0.3138216	0.03153711	0.1458649	0.3451704
0.3730618	0.1536727	0.01559517	0.09851077	0.04690313	0.003766134
0.9052923	0.3908347	1.733104	1.01306	0.7312896	0.6671548
0.2017571	0.02496672	0.02280328	0	0.1600243	0
2.618158	0.1556233	5.640637	0.8624091	2.894907	1.528385
2.423366	0.6175894	3.812989	0.708573	1.292552	1.50594
0.003530863	0.007864771	0.003591632	0.01134372	0.01440265	0.005204137
0.6218923	0.4958476	0.3630236	1.68579	0.6954426	0.6770391
0.6056271	0.8993303	0.3850315	2.513219	0.5017994	0.3998254
0.2285945	0.1446533	0.1743966	0.3004418	0.3761603	0.3637262
3.671935	0.3743908	4.32039	1.080002	1.285532	0.5478761
0.02578007	0.01148469	0.03146852	0.02484735	0.03680558	0

TCGA-R8-A6MK-01	TCGA-S9-A6UB-01	TCGA-HT-7689-01	TCGA-E1-5302-01	TCGA-TM-A7CF-01	TCGA-HT-7692-01
0.9208007	0.497315	1.302288	2.22558	0.7498383	0.7146328
1.181085	1.026016	1.448484	2.342794	0.6262408	1.018979
1.192621	0.5370935	1.595784	3.786942	0.08772173	0.6306902
0.7106978	0.3064648	0.6260044	1.331474	0.2922417	0.3881733
0.6944708	0.609495	1.5677	1.34176	1.615236	0.9665026
0.02178738	0	0.02148083	0.02252427	0	0
0.4044723	0.327701	0.5230768	1.026374	0.1081834	0.4200137
1.089209	1.148785	6.333014	4.838966	2.23919	2.17366
0.1111022	0.07530397	0.2796739	0.1637364	0.1817541	0.1120073
0	0.01143445	0	0.1558537	0.01182782	0.01700763
0.6558518	0.3186282	2.214581	2.494988	0.7121495	1.180589
0.3778869	0	0.07451399	0	0.02594199	0.03730287
2.188095	0.6252978	8.250327	11.02854	1.251066	0.9606639
1.585754	0.9849409	3.794152	4.353396	1.75638	1.282253
0	0	0.002934083	0.003076608	0.004085996	0.002937696
0.6283918	0.2371817	0.1057052	2.915707	0.4170803	0.1322942
0.1595147	0.2964222	0.7234436	0.7915674	0.1168076	0.6928415
0.1445016	0.145305	0.6821216	1.294704	0.3066199	0.09941844
0.7519193	0.4917937	1.283914	3.064339	0.8079555	0.7691457
0.0173828	0	0.03856099	0.03144876	0.01789997	0.00428983

TCGA-E1-5305-01	TCGA-P5-A77X-01	TCGA-QH-A6CZ-01	TCGA-DB-5277-01	TCGA-FG-A711-01	TCGA-DU-6401-01
1.56654	0.4413992	0.3420052	1.481015	0.7937777	1.511474
1.634687	0.3707982	0.8319269	1.612184	1.313498	1.335676
3.064608	0.6344121	0.9474913	0.3679107	1.406364	0.921626
0.4959581	0.2626975	0.4308203	0.8140911	0.9188991	6.564482
1.677273	1.143033	0.6708989	1.5898	1.145614	2.064562
0	0	0	0.0235241	0	0.06734685
0.5196743	0.2492067	0.2644501	0.7146223	0.2477727	1.683253
3.948687	1.143262	1.323482	3.477243	1.70793	3.755098
0.09415187	0.125187	0.1411472	0.1097492	0.2369404	0.2338229
0.02213634	0	0.005042903	0.06510876	0	0.09763759
1.007878	0.3688945	0.5570761	0.7451577	1.162619	1.183696
0.02427584	0.02084611	0.02212122	0.02040045	0.02228061	0.03893613
6.713693	0.6975653	2.155386	1.57947	4.846188	12.39037
2.63011	0.9057534	1.234757	1.859616	2.265419	1.579181
0.01529428	0	0.006968409	0.0128527	0	0.01226528
0.6657939	1.649472	0.1185507	0.5273511	0.1791074	0.5922381
0.7651386	0.4341148	0.2614605	0.562618	0.1128619	0.7341341
0.3881953	0.2560518	0.07177347	0.3120403	0.175563	0.4737388
1.42112	0.3606922	1.569295	0.8236225	0.5332927	11.34617
0.08375166	0.01438382	0.01526364	0.02815262	0.02049817	0.1746286

TCGA-CS-6188-01	TCGA-FG-7643-01	TCGA-CS-5393-01	TCGA-P5-A5F0-01	TCGA-FG-A87Q-01	TCGA-HT-7609-01
2.316082	0.9047069	2.12317	0.708378	3.925833	1.706764
4.265023	0.7856763	1.506937	0.6567069	8.870065	1.374121
3.815521	0.5535116	2.228138	0.1414075	5.837971	2.599776
2.000563	1.010556	0.4923114	0.4407524	2.508384	1.317478
2.687207	2.027423	2.321801	1.269964	4.139449	1.249633
0.0232346	0	0.07433034	0	0.06221302	0
0.9186965	0.4500249	0.6093097	0.1743918	1.769931	0.5550216
3.553745	1.394194	7.090478	0.7740392	7.640994	3.291628
0.2873821	0.1934159	0.540332	0.2793849	0.4387472	0.1648953
0.1791423	0.03316973	0.05877931	0.01143988	0.1906388	0.004418532
1.796541	0.6684637	2.676249	0.2333922	3.053945	1.561052
0	0	0	0.1756378	0	0
9.208157	3.257861	4.906146	0.5926687	9.265831	12.5075
3.929147	1.362855	5.560128	0.6750755	5.862644	3.67839
0.02538906	0.005729344	0	0.007903956	0	0
3.947747	0.6364284	0.9449136	0.9175382	3.886338	0.2780123
0.8278709	1.729995	0.2902423	0.7908347	2.307807	0.4690862
0.8078579	0.2023245	0.5328222	0.1221142	1.744252	0.2290886
5.229556	1.164377	3.023792	0.2387779	4.527531	4.784544
0.06024669	0.06693113	0.07412945	0	0.05584039	0.04903734

TCGA-QH-A65V-01	TCGA-TM-A84H-01	TCGA-DU-7008-01	TCGA-S9-A6WD-01	TCGA-P5-A72Z-01	TCGA-DB-A64Q-01
1.660129	0.9373146	1.02005	0.6910173	0.4218895	0.6650427
3.106769	0.9329755	0.8423361	0.7999882	0.7153999	0.7480731
2.618368	0.3844468	0.6310368	0.2896781	0.06063713	0.7071732
0.522913	0.4711907	0.3572557	0.5070613	0.3458144	0.608178
1.114863	1.261305	0.6894702	0.795783	0.5726681	1.061415
0	0	0	0.02469591	0.02067797	0
0.4418797	0.3346742	0.2129894	0.2679357	0.2741976	0.4025198
2.255403	3.313511	2.277079	1.588315	0.4246746	1.789595
0.2875711	0.3689903	0.1290267	0.1822022	0.01346105	0.07849393
0.005574374	0.02286895	0.2216685	0.02441144	0.01635183	0.01100199
1.531144	0.7146392	0.9024741	0.7968529	0.2725788	1.565735
0	0.2206975	0	0.08566666	0.01793225	0.04826139
6.080696	2.613093	1.991339	2.283467	0.2647323	3.309068
2.054205	2.2414	1.697962	1.333034	0.3816527	1.325603
0	0.006320178	0.01624356	0.003373234	0	0
0.3623002	0.3352176	0.7129237	0.2768101	0.3844058	0.3575312
0.3440664	0.225846	0.9287189	0.5665359	0.4238985	0.2308854
0.232346	0.3440831	0.1809636	0.2134259	0.04155867	0.1286248
0.8743927	1.695246	1.372307	0.7596565	0.3102747	0.8420067
0.07311318	0.02307292	0.02033141	0.01970333	0	0.03885042

TCGA-HT-7468-01	TCGA-E1-A7Z2-01	TCGA-DH-5140-01	TCGA-TM-A84G-01	TCGA-HT-7485-01	TCGA-TQ-A7RF-01
1.529278	0.5737398	1.913295	0.2729123	1.274923	1.4047
1.330305	1.317001	1.919622	0.7221705	0.4933713	1.250967
1.049492	0.8577393	0.8676409	0.2288126	3.242189	0.953733
0.3756865	0.7539788	0.909196	0.275627	0.6556257	1.091747
0.8574288	3.213821	1.492135	0.6614222	1.086859	1.581029
0	0.02371615	0.06192748	0	0	0
0.3272365	0.1944089	0.5026626	0.2947263	0.6797369	0.6700764
2.496281	0.7370156	2.047212	0.365482	6.268644	1.965501
0.06813579	0.1235109	0.08958636	0.06208267	0.2833996	0.08019794
0.128157	0	0.146914	0.0154258	0.012295	0.06429756
0.7493092	0.8398969	0.9503582	0.179104	1.537657	1.122716
0.03513587	0	0.01790148	0.04511124	0.05393329	0
3.187171	2.509999	1.703125	0.5697773	3.951574	2.666546
1.873727	1.057155	1.591564	0.483675	3.616854	1.937793
0.01936927	0	0.002819574	0	0	0
0.3710579	0.3176971	1.518055	0.09599195	0.8869441	1.834858
1.146982	0.1389089	2.216611	0.2285099	0.3440269	0.8803145
0.2809291	0.05719777	0.692839	0.01568205	0.8999457	0.4040788
1.079097	1.364141	1.321567	0.2471717	1.887111	2.225651
0.04040625	0.009460821	0.008234681	0.01037559	0.03721397	0.005897366

TCGA-19-5960-01	TCGA-QH-A6X5-01	TCGA-TQ-A7RS-01	TCGA-DH-A7UR-01	TCGA-DU-6407-01	TCGA-QH-A6X3-01
1.885922	0.8441535	0.7976929	0.8295304	1.469324	1.385619
2.329629	0.858912	0.8806225	0.7084626	1.236637	1.372119
1.245022	0.661446	0.09469652	0.3348642	1.069194	0.1515284
1.986699	0.4078496	0.3208251	0.5628846	0.614225	0.7058805
2.236278	0.6788993	0.8437086	2.418936	1.085146	1.548067
0.05094804	0	0.04843894	0	0	0.02583649
0.5220468	0.3458708	0.3678732	1.029257	0.6650283	0.5979953
1.810456	1.338538	0.7853812	0.6907522	3.938985	3.323343
0.1630682	0.1409633	0.09985457	0.08863338	0.1625359	0.8381551
0.04532505	0	0	0.01562916	0.009401941	0.06129332
0.7718403	1.05976	0.6385262	0.6532746	1.314634	0.8438221
0	0	0.02100349	0	0.04124259	0.04481158
2.44963	2.017589	0.7303934	0.3748628	6.061548	1.190789
1.886015	1.26686	0.8012584	0.5671381	3.121871	1.959638
0.03827468	0	0	0	0.009743877	0.003529026
1.204805	0.5179836	0.1754622	0.3530084	0.2795314	1.16191
1.964532	0.4227655	0.2009639	0.836053	0.1392756	0.5548694
0.5375767	0.1414399	0.03407347	0.100629	0.372767	0.1453936
1.554442	0.8258325	0.7147164	0.4217771	1.635343	2.326072
0.0304862	0.01079766	0	0.01576855	0.05217188	0.005153332

TCGA-TM-A7C4-01	TCGA-FG-A70Y-01	TCGA-27-2528-01	TCGA-FG-8191-01	TCGA-DU-7306-01	TCGA-TQ-A7RM-01
0.8169695	1.232936	1.533674	1.434951	1.544357	0.5623035
0.5340246	1.951027	1.452462	2.009404	3.957123	1.347574
0.6278762	4.375224	1.543018	0.9624623	2.18469	0.5514973
0.3094109	0.7248585	2.352436	5.872901	0.6763507	0.5846389
1.561271	3.49977	2.267161	1.774685	1.372599	0.5801245
0	0.02459345	0.08308219	0	0	0
0.2674968	0.6107307	0.6676974	2.064284	0.4459505	0.2193993
2.303878	4.751823	2.305006	3.863915	3.576957	0.7524859
0.2787689	0.6964337	0.3335258	0.3158458	0.1170658	0.03554385
0.0404053	0.0291722	0.1478253	0.0126951	0.09141806	0.007196157
0.5687042	2.109674	1.318595	2.362592	0.8718856	0.2972074
0	0.04265563	0.04803342	0.01856279	0.01670895	0
1.819166	11.22303	2.308557	13.53762	4.544261	0.8595413
1.287242	4.566612	1.163104	2.559771	1.937778	0.7656382
0.01196423	0.003359239	0	0.002923734	0.01315872	0
0.9738112	0.2285975	2.725601	0.5910318	0.3239444	0.8781931
0.3277739	0.3241066	1.662633	1.211934	0.7523448	0.5596511
0.2112507	0.3163393	0.3784855	1.088405	0.3717468	0.1280248
0.8616107	3.622613	2.957343	18.0827	2.279138	0.5461861
0.017471	0.05886476	0.03314306	0.0811194	0.065332	0.01089051

TCGA-HT-7602-01	TCGA-HT-7695-01	TCGA-DU-8164-01	TCGA-WY-A85E-01	TCGA-DB-5278-01	TCGA-QH-A6CY-01
1.046381	0.5516209	0.3351053	0.8317787	0.8884963	0.6096969
1.47545	0.402793	0.4825814	1.434432	0.5005156	0.6704862
1.507013	0.458532	0.6600232	0.7838232	0.7205808	0.6273528
0.9299584	0.2999811	0.3112426	0.6365479	0.5014514	0.4014695
1.634245	0.9354018	0.6961296	0.8585665	1.148676	1.018525
0	0	0	0	0	0
0.5097695	0.2612944	0.299192	0.2786242	0.5668219	0.2922819
4.850672	1.787805	1.740834	1.383006	2.702775	1.496681
0.341716	0.1597068	0.1108807	0.08700184	0.1426708	0.4178048
0.01306259	0.009593605	0.01803917	0.0139878	0.04726625	0.04229408
1.642321	0.5442114	0.3662331	0.7795579	0.9055091	0.5518613
0	0	0.04747841	0.02045298	0	0
4.311064	2.917681	0.9553275	2.952357	2.516755	1.034705
3.379392	1.495022	0.9532872	1.273051	1.867803	1.319506
0.006016736	0.002209447	0.002492697	0	0	0
0.2739643	0.1857311	0.2619271	0.8639878	0.473875	0.5848631
0.6557586	0.4105538	0.1068892	0.5410435	0.9724698	0.6497275
0.4824916	0.2243184	0.1210363	0.2133025	0.1561672	0.07166112
1.057543	1.072001	0.502028	1.038077	1.34531	0.6717447
0.02635818	0.04516946	0.0327601	0.02822511	0.03576586	0.01422376

TCGA-FG-7636-01	TCGA-P5-A5F4-01	TCGA-HT-A5R5-01	TCGA-FG-5963-02	TCGA-DU-6393-01	TCGA-S9-A6TY-01
1.562486	1.115944	3.055638	1.507796	0.4998801	0.4591122
1.006093	0.7390601	16.54553	2.748503	0.6567955	0.6048133
1.880389	0.5512176	4.221278	1.147819	0.2441387	0.3032739
0.6200742	0.8600265	9.670698	2.937568	0.3556636	0.2133977
0.8667157	2.765743	2.988043	5.500263	0.7308603	1.022622
0.02564939	0	0	0.03173675	0	0
0.4205126	0.3935644	1.421755	0.3749312	0.2569265	0.1553599
2.793295	1.475749	10.5593	1.389351	0.4589557	1.070594
0.1558421	0.2737151	0.1971134	0.05853709	0.08671554	0.1709015
0	0.02347029	0.07065553	0.1317589	0.03291807	0.009436493
1.50889	0.9167882	3.375191	3.677791	0.2620808	0.2441715
0.04448708	0	0.0516563	0	0.1877181	0.1862737
2.787577	1.579022	30.07176	2.627495	0.3031812	0.5228286
1.938301	1.095596	3.928809	1.251104	0.5497038	0.6200197
0	0	0	0.004334947	0.0272922	0.009779686
0.7993831	3.334839	0.56588	2.264519	0.4324467	0.6916098
0.2253482	2.071575	1.16295	2.277112	0.3494693	0.1863832
0.3711618	0.1849164	1.664045	0.3508155	0.07027631	0.08154266
1.969258	0.6160633	19.63356	2.762033	0.4747095	0.310365
0.005116014	0	0.2851428	0.1645851	0.009963498	0.01428098

TCGA-DU-5855-01	TCGA-QH-A65Z-01	TCGA-S9-A7QY-01	TCGA-HT-7680-01	TCGA-P5-A5EV-01	TCGA-HT-7482-01
2.226442	1.066653	0.876945	1.558103	1.56997	1.833181
2.731204	0.9603403	1.264736	1.105784	0.9070243	1.282023
4.564147	0.4642134	0.963844	4.804891	1.09276	3.930222
2.176935	0.5319501	0.4312049	1.376901	0.7025753	0.8591725
2.548574	0.8417892	1.62061	1.990651	1.982616	2.155779
0	0.02793569	0	0	0	0
0.8352363	0.3771728	0.225542	0.6419472	0.6493242	1.516372
4.948285	1.004028	1.619257	3.304243	4.524627	9.359954
0.424895	0.1273	0.2139671	0.2311085	0.151616	0.414308
0.08082439	0.01656834	0	0.3482248	0.02511488	0.0266443
3.208994	0.6375682	0.5223136	1.555462	1.259716	2.359963
0.03732048	0	0	0	0	0
12.98427	1.692877	1.43863	9.439833	2.884177	18.24514
4.725294	0.9961936	1.190403	3.791686	2.905697	6.027929
0.0176345	0	0.003453458	0.002797592	0.01041131	0.003068146
1.047089	0.2825752	0.3248655	1.343839	0.3716118	0.3561687
1.176286	0.5045052	0.7527784	2.019384	2.405853	0.5701152
0.8995131	0.2358101	0.2083389	0.568062	0.561706	0.686202
10.0359	1.397258	0.7018477	7.667819	2.173081	6.426402
0.06866969	0	0.02017193	0.1266424	0.02533888	0.0985671

TCGA-DB-A4XF-01	TCGA-FG-A4MT-02	TCGA-DB-A4X9-01	TCGA-HT-A5R9-01	TCGA-S9-A6TX-01	TCGA-S9-A6WE-01
1.038054	1.473803	0.9636364	1.564205	0.9487974	1.034125
1.236872	1.873433	1.179206	1.331212	1.139297	0.8884621
0.5848519	1.439331	0.9661759	0.2197187	0.2105687	0.7093811
0.4623353	1.10408	0.8578396	0.302409	0.4597414	0.9657837
1.612867	1.08398	1.84632	1.081578	1.634278	1.423239
0.05698325	0	0	0	0.02393547	0.02687859
0.4190252	0.4889803	0.2958314	0.2777439	0.3981842	0.959094
1.547562	1.681737	2.238153	1.138921	1.70111	0.7553955
0.1576548	0.4280392	0.277351	0.0762127	0.1324439	0.1253992
0.02253075	0.03295529	0.006738251	0.1777526	0.03312366	0.02656897
1.188399	1.328284	1.830715	0.3206305	0.8194085	1.322074
0	0.02409363	0.02955804	0.02436657	0	0.04661903
2.107546	3.841476	5.769686	2.006447	1.716046	3.976456
2.043943	1.811197	2.746607	0.6223133	1.473698	1.73166
0.01167508	0	0	0.007675717	0.009808092	0
0.5958702	4.196438	0.2795394	0.7527776	0.5398447	0.2902529
0.5006386	0.8407594	0.08318085	1.988567	0.5023599	0.6953234
0.3034917	0.2903571	0.06165169	0.08470568	0.1972329	0.1404538
1.189928	5.065125	2.974825	0.8502372	1.173235	5.962346
0.0227317	0.01662461	0.04758844	0.1737336	0.01909662	0.01608356

TCGA-HT-8558-01	TCGA-HT-8010-01	TCGA-HT-7694-01	TCGA-DU-7299-01	TCGA-E1-A7YU-01	TCGA-P5-A77W-01
0.6067687	0.8865608	0.5072599	1.321104	1.176318	0.49935
0.4679203	0.7646172	0.4782621	1.283094	1.185801	0.4421097
0.4476747	0.462499	0.9206322	2.113012	1.232797	0.2203475
0.4929243	0.4364974	0.4203919	0.5109258	0.5243988	0.2762131
1.657084	1.605104	1.722519	1.393739	1.850149	1.087618
0	0	0	0	0	0.04508466
0.135515	0.2716096	0.33074	0.7061113	0.452728	0.673928
0.9282161	1.473439	0.9018643	6.958026	2.393268	0.8954695
0.15133	0.2371229	0.1488379	0.1910346	0.4561215	0.1051688
0.01646224	0.06236035	0.0242867	0.02757143	0.01108148	0.0267392
0.4833056	1.001843	0.5357718	1.728664	1.444708	0.5632638
0	0.03907859	0	0	0.02430504	0.0390981
1.75903	2.48073	1.001922	4.200884	2.623323	0.4040413
0.7061124	1.788963	0.7515259	4.606622	2.158932	0.5840582
0	0.009232616	0.005593328	0.00476236	0.0114845	0
0.3955381	0.3695782	0.1623267	0.2239968	0.6244536	0.2033695
0.1727364	0.8138013	0.3697645	0.6211526	0.2325536	0.4291094
0.1631732	0.1901889	0.1069907	0.6902251	0.1915147	0.08155019
0.4060806	1.267803	0.5274039	1.569497	1.541982	0.6595866
0.03737039	0.01797615	0.0285872	0.04868033	0.03913111	0.004496281

TCGA-DU-6400-01	TCGA-WY-A85A-01	TCGA-FG-A60K-01	TCGA-QH-A65S-01	TCGA-DU-7309-01	TCGA-E1-5303-01
1.662898	1.830623	1.062411	1.814714	1.11077	3.47992
1.757827	2.603986	1.705414	0.9072956	0.5543465	3.351946
0.3390131	1.424054	0.8520093	2.683031	1.137723	7.374552
0.6288325	0.600394	0.8047343	0.8092246	0.932546	2.001182
1.144759	1.736814	1.4216	1.836585	1.876917	2.262441
0	0	0	0.0334736	0	0.02441562
0.4515376	0.5151593	0.8278609	0.7989704	0.348513	1.712986
1.813522	4.339313	0.9493083	4.097654	3.373584	10.71491
0.0827846	0.298568	0.2550532	0.2251717	0.2484934	0.2543072
0.03428274	0.01280068	0.02610895	0.006617605	0.02597955	0.02896126
0.5220088	1.050988	1.439495	2.193088	0.8236628	3.458673
0.0751924	0	0	0.02902881	0.01628029	0
1.692223	4.034271	5.124975	6.742518	4.310178	18.40077
1.132907	2.466814	2.273808	3.508758	2.677809	8.351262
0.004737274	0	0.003607799	0	0.005128456	0.006669897
1.941351	0.6726532	0.4332567	0.6039757	0.5440191	0.9111155
1.785929	0.7742928	0.8379901	1.029313	0.4673153	0.5839403
0.3310964	0.6831996	0.1274048	0.4171074	0.4603083	0.966691
1.465821	1.983617	3.672679	1.741217	5.164343	11.15561
0.0276708	0.02582969	0.05268362	0.08679615	0.1872233	0.04869926

TCGA-E1-A7YH-01	TCGA-S9-A89V-01	TCGA-FG-A713-01	TCGA-HT-7684-01	TCGA-EZ-7264-01	TCGA-HW-7487-01
1.000789	2.138471	0.7942349	3.917011	0.595895	0.5464807
2.684529	2.010838	0.6323624	6.501487	0.5438762	0.5088451
1.241367	1.979277	0.282858	5.933392	0.4140867	0.5015813
0.5607553	0.9448871	0.5789747	3.464074	0.338525	0.3398644
1.880216	2.171114	1.499491	1.583467	0.5325496	1.598488
0	0	0	0.02823288	0	0.01973597
0.4195869	0.678396	0.2034881	0.5921987	0.2975239	0.3568723
3.126777	3.503386	0.8471413	3.067015	1.090016	0.9759876
0.1811654	0.2539313	0.5599011	0.2297398	0.08992622	0.2312608
0.009298771	0.1247717	0.004767341	0.005581534	0.0109238	0.01950864
1.804559	1.267599	0.4175128	2.77182	0.3877458	0.5669186
0.1223701	0	0	0.07345194	0.03194559	0.06846133
3.412351	3.366707	1.262357	10.72316	0.8226974	1.662022
3.105632	4.027598	0.797785	1.695028	0.7735454	1.142284
0.003212318	0.00359193	0.006587631	0	0.002515796	0
0.2185995	2.559349	0.5274009	0.5441484	0.2265894	0.7283923
0.1607051	1.655773	0.4943468	0.9232824	0.3416192	0.2600919
0.4112161	0.6553628	0.12601	0.243993	0.1443685	0.03966544
1.25863	2.091321	0.5306983	7.893764	0.7323832	0.5083735
0.01407256	0.03671632	0.009619721	0.0732071	0.0257162	0.04330179

TCGA-S9-A6U6-01	TCGA-S9-A6U9-01	TCGA-DU-5849-01	TCGA-HW-A5KJ-01	TCGA-FG-7641-01	TCGA-S9-A7IS-01
1.238435	2.1701	1.298666	1.724432	0.2206232	1.430269
2.450116	2.807671	2.110561	0.9329061	0.4170035	5.011821
0.5968768	1.896312	0.5583666	0.02570279	0.2642469	1.501547
2.776279	4.067448	0.8260452	0.4092724	0.2551462	4.211018
1.418116	3.840277	1.691441	1.809114	0.645088	1.215186
0.02544275	0.1310807	0.03808192	0.3155383	0	0.02133522
0.6563581	1.64969	0.7620613	0.4120759	0.1955306	0.9361847
5.355945	5.136193	2.485253	0.2842271	0.5698096	2.133209
0.2070356	0.2645278	0.1879966	0.09129365	0.1437197	0.1180557
0.005029935	0.01036566	0.1242227	0.03119037	0	0.07170417
1.281484	3.316568	0.7267747	0.4449166	0.2818939	1.498562
0	0.02273503	0.0165126	0.2508363	0.02344376	0.07400889
12.00148	14.30552	1.338049	0.1271763	0.6537411	7.059326
2.234568	5.976255	1.258547	0.1666582	0.6420506	2.072935
0	0.003580882	0.002600819	0	0.003692512	0.01457097
1.505896	0.508862	1.751648	1.358644	0.1071622	1.181123
0.4719024	1.791436	1.505596	7.85465	0.1319487	1.541216
0.5778248	0.4267833	0.3558979	0.2695223	0.04346545	0.3044452
4.154936	12.01762	2.052358	0.3024934	0.4867659	3.436134
0.07104716	0.04706151	0.06456428	0.005244758	0.02156826	0.04681063

TCGA-P5-A72X-01	TCGA-DB-A64U-01	TCGA-DB-A4XG-01	TCGA-DB-A64R-01	TCGA-CS-4943-01	TCGA-W9-A837-01
1.378433	0.7498853	0.399069	1.051408	2.108803	0.4030916
1.047885	0.8256527	0.2805418	1.969697	1.735447	0.4012621
0.2793982	0.8057277	0.09391822	0.5304672	3.629965	0.2478346
0.8390151	0.5500856	0.2664827	0.3700087	1.198001	0.312959
2.860464	1.518407	0.7405453	0.9674918	1.750905	1.059892
0	0.02497842	0	0	0	0.02304941
0.5889011	0.7106242	0.1679467	0.3540389	0.8901903	0.2611865
1.509714	3.11847	0.623141	1.664915	7.151549	1.009043
0.1578808	0.1788663	0.1250954	0.1142967	0.2361912	0.2275731
0.07705682	0.004938139	0	0.006311005	0.07172838	0.004556782
1.053169	0.8207118	0.3402689	0.4836155	1.709021	0.371863
0	0	0.02083086	0	0	0.03997759
1.131096	2.309589	0.4783711	2.661057	9.387277	1.324639
1.482244	1.751898	0.6541414	0.913811	3.959974	1.11573
0.003549301	0.006823643	0.003280967	0.004360352	0.02623661	0
0.4937184	0.1160879	0.6074286	0.1090896	0.9860603	0.4221897
2.295644	0.3779473	0.0234485	0.140232	0.770868	0.1012528
0.3237925	0.3714928	0.02413816	0.1796437	0.5361761	0.06485472
1.969025	1.261838	0.1561856	1.421043	4.248582	1.400726
0.01554882	0.03487527	0.004791099	0.0891421	0.07236812	0.01838969

TCGA-DU-7014-01	TCGA-QH-A6X9-01	TCGA-S9-A7R1-01	TCGA-DU-5874-01	TCGA-DB-A4XC-01	TCGA-P5-A5EW-01
1.360168	0.7720567	0.7338159	0.6327793	1.936667	2.024254
0.7510423	0.6918054	0.8457301	1.12617	4.817433	1.247017
1.400915	1.030886	0.06002332	0.6572924	1.282941	1.760014
0.5643349	0.5117019	0.8083351	0.4803655	0.8896769	1.098859
1.902683	1.439662	0.9198286	1.301753	2.457557	1.402332
0.1747787	0	0	0	0	0
0.6489367	0.2838357	0.4515475	0.541951	0.7416544	0.849615
3.036926	2.240186	0.5061102	1.121423	3.746266	7.812851
0.204801	0.1303913	0.1032671	0.0854643	0.2225036	0.2232638
0.04146371	0.0145463	0.03034932	0.04178036	0.05676017	0.04576668
1.97383	0.8203345	0.3020368	0.4044587	1.912515	1.958524
0.03031415	0.02126961	0.1863826	0.01666126	0.1067076	0.0223067
3.510586	3.140015	1.257852	0.7160426	6.499634	6.725281
2.370112	2.138708	0.5559917	0.8309735	4.082197	4.106176
0	0	0.004193745	0	0.00560233	0
0.5351551	0.2514416	0.4868348	0.3597864	1.216606	0.2461215
1.057827	0.3232221	0.4046215	0.7220653	1.121088	1.393593
0.3618091	0.2070312	0.06170698	0.1390072	0.5193278	0.6823955
1.263204	1.398475	1.873517	0.5765667	1.405255	4.078354
0.05577803	0.02446005	0.024496	0.02682464	0.09817099	0.04104433

TCGA-CS-6669-01	TCGA-DU-A7TC-01	TCGA-06-2569-01	TCGA-DB-A64O-01	TCGA-TM-A84R-01	TCGA-HT-7854-01
0.6212648	0.9759452	4.895528	1.10587	0.6907848	2.846244
0.1708019	1.270173	2.105882	2.373415	0.4950641	2.388465
0.4735227	0.7481077	0.311373	1.315988	0.2654489	3.107767
0.3288736	0.6138329	9.393491	1.519934	0.5028028	3.879966
1.240357	1.387225	6.874999	3.874557	1.818935	1.388045
0	0	0.08687606	0	0	0.1032258
0.07007703	0.3921089	0.2932386	0.5901641	0.2321328	2.17019
0.4974502	2.68864	0.5634379	3.241235	1.227528	4.502218
0.5781544	0.1998091	0.1225359	0.136111	0.3482106	0.4211103
0.02394252	0.009456558	0.02862511	0.01209813	0.01952258	0.07346647
0.390773	1.03523	7.144728	2.113032	0.3740063	2.408698
0	0.02074109	0	0.07960454	0	0.01790379
0.4410296	3.762844	0.7662107	4.387394	1.116766	13.93806
0.7186632	1.627473	0.4659135	2.525355	0.7514061	4.700539
0.003308436	0	0.00395549	0	0.01348839	0.002819939
0.9038723	0.568849	3.744666	0.8113991	0.7424113	4.961125
0.2837378	0.5136437	0.876345	0.7616659	0.5181457	1.380522
0.04868051	0.2259208	0.09894218	0.3505244	0.14389	0.6514358
0.3513311	0.7476561	2.636133	1.438582	0.3395695	12.1951
0	0.02385225	0.005776084	0.04882411	0.0393934	0.1029468

TCGA-HT-7688-01	TCGA-DU-6408-01	TCGA-HT-7481-01	TCGA-DU-A7TB-01	TCGA-HT-7681-01	TCGA-HT-7474-01
0.6402399	1.95874	0.5517026	0.8529495	1.487932	0.8696916
0.6824329	2.09794	0.5995997	1.047913	1.9608	1.135331
0.8271753	1.475625	0.346915	0.2979675	1.338099	0.3305519
0.5694261	0.7700557	0.4270341	0.2658331	0.7744529	0.4743049
1.574316	1.747311	1.079746	1.313227	1.520046	1.503727
0	0.04869732	0	0.02032208	0	0
0.3516158	0.4578916	0.496289	0.2792776	0.405679	0.3549001
1.459641	5.276963	1.291542	0.7303894	2.928571	1.585762
0.2461391	0.3909817	0.1540258	0.1455231	0.1918443	0.2676226
0.04627346	0.009627275	0.0187103	0.004017598	0.2931833	0.0353934
0.5136503	1.815614	0.5586154	12.25885	0.9146066	0.6418514
0.0156141	0	0.02051867	0	0.07419686	0
2.873687	5.971297	1.272244	1.266189	4.66543	1.890228
1.126111	3.713929	1.043135	1.316332	1.515571	1.33836
0.007377903	0.009977407	0	0.005551618	0.02337276	0.002717082
0.5685194	1.154908	0.2134565	1.102813	0.9901801	0.57101
0.7294121	0.4991475	0.7506549	0.267816	4.148187	0.9806348
0.2460667	0.5040411	0.1521689	0.191964	0.9858698	0.3198343
2.228859	6.162298	0.4970371	0.5997045	1.426444	0.70641
0.09696359	0.03399599	0.03775435	0.004053431	0.03413055	0.03967674

TCGA-DU-7302-01	TCGA-DH-A66F-01	TCGA-HT-A5RC-01	TCGA-P5-A72W-01	TCGA-HW-7493-01	TCGA-HT-A61A-01
0.7092159	0.8482756	2.010434	1.594096	2.669367	1.20996
0.4459983	1.374291	2.489518	1.274259	2.434445	0.9778745
0.2889713	0.3251215	2.025458	0.5847216	2.243869	0.307831
0.282408	0.6517141	1.504024	0.8442814	0.9106645	0.8438086
1.081341	1.13937	3.362001	1.567853	1.616019	1.979697
0	0.02771756	0	0.02848528	0	0.02862927
0.3015489	0.5546591	0.9033539	0.3708577	0.5361581	0.1449514
1.437979	1.250853	3.881291	2.586392	7.172565	0.9670633
0.355291	0.07217487	0.2340584	0.1668914	0.3087334	1.003304
0.03147012	0.01643897	0.2020188	0	0.1890929	0.02829949
0.483209	0.5453362	1.541379	1.210552	1.289071	0.6533976
0	0.02403709	0	0.2964343	0.02073689	0.02482773
1.403939	0.820115	7.461899	1.928785	9.714746	1.56386
1.198981	0.6254815	3.635692	1.668518	3.973761	1.298092
0.01242463	0	0.03877148	0	0	0.007820988
0.9418628	1.004024	3.457098	0.3504338	1.899048	1.068356
0.2774895	0.4870372	1.385465	0.500527	2.544356	0.4751095
0.1691054	0.1448379	0.6788778	0.4007488	1.143793	0.143848
0.881497	1.129873	4.00366	0.9403327	8.724877	0.6586963
0.0635016	0.00552853	0.03963179	0.01136332	0.2527827	0.04568302

TCGA-TM-A7CA-01	TCGA-DU-6406-01	TCGA-HT-A74L-01	TCGA-CS-5396-01	TCGA-DB-5273-01	TCGA-CS-6668-01
1.050998	2.792739	0.8052417	0.6790057	4.071906	0.8131677
1.112445	3.620877	1.654449	1.477379	5.791286	0.714759
0.6755621	2.807791	1.443011	0.3476553	7.270405	0.08868532
0.3880935	1.789432	0.6425307	0.9540435	0.9924043	0.2516349
1.538768	3.323845	1.899113	0.873487	1.787454	0.6242195
0	0.04017439	0	0.08891591	0	0.02268205
0.4709062	0.5666282	0.7525183	0.5187855	0.8368527	0.3281154
3.24805	2.659806	2.518948	1.398418	9.682684	0.9623137
0.1825727	0.2746057	0.1647459	0.09261268	0.1426854	0.04429703
0.01188109	0.1151637	0.004169276	0.10547	0.1193401	0.01345247
1.016871	1.387189	2.336038	0.8117209	2.464425	0.2231319
0	0	0	0.03084368	0	0.1376915
2.197088	3.663226	9.443944	1.851727	9.666577	0.4259049
2.1724	2.793906	3.127176	1.452854	5.308037	0.8720389
0.008208795	0	0.00576121	0.009716075	0.06184019	0.009294478
0.1642975	4.571688	0.121075	0.4594247	1.470529	0.3689541
0.2786674	0.9216196	0.2676333	1.354062	1.231177	0.4096271
0.2717654	1.308031	0.1992112	0.1572591	1.273888	0.07293837
1.134729	4.701996	5.701316	3.277661	4.000563	0.2836217
0.02397412	0.02403949	0.05889046	0.1064107	0.09890371	0.01357245

TCGA-DU-5871-01	TCGA-DU-7292-01	TCGA-R8-A6YH-01	TCGA-HT-8107-01	TCGA-DU-A5TS-01	TCGA-DU-A6S3-01
2.529601	1.126017	1.368035	0.4724056	1.370312	0.7803526
2.449166	0.8990992	1.416773	1.960132	1.686368	0.9315519
2.164127	1.155997	0.7885451	0.3017675	0.3354407	0.2203809
1.253756	0.4177532	1.388389	1.300323	0.50572	0.3873169
1.519383	2.272751	1.869242	2.376076	2.235506	1.556079
0.02213978	0	0.04889148	0	0	0
0.7312853	0.204342	0.6365316	0.4047207	0.6370744	0.3533222
3.781159	1.214411	2.378156	3.556003	2.095798	0.7691847
0.2570258	0.1475587	0.08752596	0.3600736	0.2904161	0.2170964
0.02188475	0.06234683	0.02899698	0.003814534	0	0.03342906
1.637836	0.5661848	1.06293	0.3302719	1.492134	0.393679
0.0383999	0	0.02119971	0.0334657	0	0
8.53491	1.811277	4.527626	5.599247	3.426888	1.026292
3.736294	1.295933	2.2747	0.8452947	2.342323	0.6654091
0	0	0	0.002635509	0.004687354	0
0.3328963	0.6520103	0.1202953	0.2030845	0.3987203	0.4892393
0.6699918	1.144847	0.3698873	0.4143815	0.5192454	0.6327571
0.26253	0.174302	0.2014377	0.5080051	0.2827769	0.1019532
3.931142	0.8971284	5.428793	1.022978	1.965301	0.521547
0.06623982	0.03145145	0.0146278	0.1462451	0.06160324	0.01124241

TCGA-HT-7610-01	TCGA-DU-A6S8-01	TCGA-P5-A781-01	TCGA-HT-7881-01	TCGA-HT-8109-01	TCGA-FG-8185-01
0.6402973	0.992469	0.812607	0.502023	0.8575148	0.7815164
0.5235393	1.3819	0.6716404	0.4066637	0.4418189	1.548468
1.033899	0.4437848	0.6697509	0.2204722	1.25683	1.681008
0.4634441	0.6812786	0.4929356	0.2580352	0.3716774	0.6552469
1.43418	1.018142	1.27575	1.139306	1.241255	1.214274
0	0	0	0	0	0.01849173
0.1765472	0.3078567	0.5582452	0.1137032	0.4361002	0.2541238
2.088736	1.288211	3.102964	0.5984255	3.398018	1.254255
0.3072021	0.1693267	0.3076145	0.133482	0.3877741	0.06821441
0.01286807	0.02243889	0.03269658	0.01216106	0.05600889	0.0329017
0.7299605	0.59736	0.7948978	0.3187044	0.8489648	1.15331
0.02822357	0.04921527	0.04097912	0.01778191	0.05669742	0
5.411979	1.388538	1.707363	0.7992058	1.822842	2.314816
2.19306	0.9466577	1.852929	0.7183467	2.461957	1.251867
0	0	0.01290884	0.005601484	0.005953421	0.002525799
0.2557974	0.5585326	0.5038178	0.1933944	0.9294218	0.414538
0.3177019	0.803296	0.6227363	1.020837	1.010516	0.3068746
0.1733344	0.2281165	0.5365844	0.0782995	0.4555145	0.1523755
1.062143	0.7238196	0.8094939	0.517917	1.111815	1.812803
0.05193138	0.005659755	0.0188504	0.0204492	0.07824243	0.03319515

TCGA-E1-A7YW-01	TCGA-HT-7607-01	TCGA-VM-A8C9-01	TCGA-P5-A5F2-01	TCGA-HW-8319-01	TCGA-FG-6690-01
1.828588	1.066802	6.020866	0.620261	1.439791	1.625102
2.906186	0.6868986	9.499306	1.214121	1.101717	1.053911
2.374685	1.474315	16.16645	0.6838796	0.6814476	3.51223
0.985766	0.8324794	2.177634	0.5309644	1.362131	0.4308326
2.022751	1.698867	6.062268	1.462343	1.682653	1.329395
0.08008967	0	0	0.02915137	0	0
0.431244	0.4751589	1.040908	0.3443881	0.4274035	0.6512159
4.739748	2.434627	7.745	1.323436	4.04231	5.23657
0.2606859	0.1963731	0.428788	0.7242928	0.2053044	0.2796138
0	0.06361186	0.2626239	0.01152623	0.03150238	0.07838335
2.295334	0.6251527	6.109376	0.7628162	1.179589	2.145198
0.02315165	0	0	0.02528051	0	0
8.795291	2.420173	12.46256	1.476269	4.040461	8.354303
3.289255	1.574347	5.963116	1.072031	3.088123	4.589213
0.003646503	0	0.01814502	0.003981808	0.005441347	0
1.178693	0.907145	9.937216	0.693348	0.4873614	0.7384227
0.1433351	1.305497	13.68116	0.3414875	0.2333301	1.632841
0.5365484	0.4850144	2.162592	0.1054594	0.6925559	0.5777197
5.020653	0.6739252	3.189292	1.720514	1.120791	3.079988
0.0266244	0.0240672	0.01987247	0.0348871	0.05562086	0.05931184

TCGA-DU-6397-01	TCGA-DU-A6S6-01	TCGA-DU-7294-01	TCGA-TM-A84S-01	TCGA-HW-A5KL-01	TCGA-HT-7874-01
1.588149	0.6169508	0.5750802	2.305016	1.300764	0.5945437
0.9690809	0.6478382	0.3927112	1.526364	0.8735571	0.332632
0.3145326	0.1436828	0.6428711	0.2399027	0.8773339	0.5582884
0.8560433	0.3164116	0.1545679	0.7766501	0.4541082	0.5066361
1.617897	1.79222	0.646679	1.111468	1.928119	1.549086
0.150163	0	0	0	0	0
0.6154673	0.1701099	0.3631652	0.7232175	0.595088	0.3491745
1.194172	1.040714	2.023775	3.780378	4.150194	0.9040025
0.1815429	0.08931062	0.0874694	0.1272247	0.2164027	0.3341857
0.1102647	0.0232479	0.0209711	0.03773806	0.01971566	0.01613056
0.5951047	0.2949883	0.6177658	1.196458	2.020968	0.3692226
0.09301688	0.05098966	0	0.02364885	0	0.0176896
0.653033	0.5185653	2.752352	2.769732	3.582043	2.100807
1.35963	0.7105084	1.603234	2.222202	3.533717	0.9774487
0.02051089	0.004015566	0.002897839	0	0	0.005572403
0.8503662	0.2049456	0.1333993	1.479847	0.2112943	0.6050537
1.193643	0.2295884	0.6627311	1.504064	0.4745939	0.3683812
0.1250307	0.1713472	0.1108613	0.4603797	0.5862631	0.04509595
3.723159	0.3014369	0.4509817	1.029787	2.163563	0.9947476
0.0727392	0.03518287	0.05924279	0.01087847	0.04475588	0.1464699

TCGA-HW-7495-01	TCGA-FG-A4MY-01	TCGA-12-1597-01	TCGA-DU-7304-02	TCGA-HT-7605-01	TCGA-HT-7902-01
0.5024443	1.832379	3.261144	0.6830812	0.9594582	1.468994
0.5806604	2.395339	5.411665	1.416048	0.4671393	4.419454
0.6017923	0.6739836	3.773689	0.03694855	0.2695439	0.630726
0.4770861	0.9066475	3.663847	0.5758237	0.7933386	0.6814834
1.599941	1.617798	2.68354	1.165357	1.203767	2.010877
0	0.04055935	0.03574645	0	0	0.01743932
0.3028032	0.7431861	1.508218	0.2642889	0.6772994	0.2522748
1.018177	2.893541	3.274668	0.194078	2.221535	2.346895
0.1068752	0.2860382	0.6709633	0.1230351	0.1552022	0.2497602
0	0.04811058	0.03533469	0	0.03747927	0.08963989
0.6880101	1.069314	2.320899	0.4908414	0.7866786	0.9607202
0	0	0	0	0.02989213	0.01512364
1.527355	2.13476	34.37447	0.3441314	2.044667	2.381536
1.169758	1.375734	3.012539	0.3685789	1.43324	1.988349
0.006727421	0.005540033	0	0	0.002354081	0.002382051
0.1413805	0.443531	5.570329	0.465022	0.3298155	0.529207
0.1442391	0.8512635	1.099203	0.3689967	1.219755	1.259783
0.1534308	0.1793359	0.3951385	0.03798496	0.3740912	0.3750309
0.8252568	2.028655	15.00072	0.397031	1.168037	0.9943782
0.05403119	0.09707935	0.1782492	0	0.02062557	0.1078316

TCGA-VM-A8CE-01	TCGA-TM-A84O-01	TCGA-HT-7467-01	TCGA-QH-A6X4-01	TCGA-S9-A6U8-01	TCGA-DU-5854-01
1.808434	1.872933	0.7433517	0.8754795	2.54224	3.918275
1.846619	3.017555	1.565326	1.084062	2.837769	4.484178
0.4349289	1.618287	0.4852023	0.3005401	2.242508	2.272585
1.60858	1.012895	0.3486055	0.4072834	0.7454111	0.9833833
1.618908	1.632445	1.493658	1.042241	2.318418	3.436118
0.02471931	0	0	0	0	0.1841532
0.9356835	0.7171219	0.2434784	0.3079189	1.085363	1.315318
4.201667	2.282418	1.327492	1.604198	8.790477	6.948279
0.1126433	0.2679147	0.24328	0.2001538	0.5612243	0.2647372
0.04398222	0.06656926	0.03383871	0.004675714	0.03850877	0.2366416
0.9726926	1.429892	0.6869971	0.4560211	2.55919	2.404879
0.04287391	0	0.0148437	0.02051051	0.01876919	0.03992512
5.281565	1.700435	2.201102	1.002587	8.811398	7.347991
1.640755	2.263169	1.317323	1.406766	6.772821	6.052304
0	0.003832794	0.007013876	0	0.002956243	0.01886523
0.4798082	1.135348	0.5849227	0.8922811	0.3993885	4.615965
0.3981574	1.835285	0.7686132	0.196247	0.5281939	5.134642
0.5266182	0.5639593	0.2889671	0.1093279	0.4741317	1.535964
2.200764	0.9824473	0.5950006	0.6801968	3.566904	2.34875
0.0049305	0.02798458	0.05803887	0	0.09928903	0.05968805

TCGA-DB-A75P-01	TCGA-CS-6186-01	TCGA-QH-A6XC-01	TCGA-TQ-A7RQ-01	TCGA-HT-7469-01	TCGA-DB-A4XB-01
2.391228	1.774079	1.82015	0.440501	1.515569	1.723928
1.57443	3.720304	4.883001	0.8325982	2.671915	1.411984
0.4575589	3.44452	1.107605	0.139743	1.595459	1.519481
1.648083	1.925515	0.8172082	0.3922783	1.714253	0.8475189
4.123873	3.280967	3.521894	0.7968351	1.364631	1.333986
0	0.03203514	0	0	0.02331729	0
0.46331	0.5638226	0.3521145	0.5022451	0.7589356	0.2468077
7.023784	3.696487	2.354364	0.8223249	2.659042	4.251916
0.2886872	0.1320778	0.3223774	0.2282335	0.0708363	0.1604302
0.1558592	0	0.009956146	0.004037578	0.01843896	0.1723964
1.090626	1.329898	1.347538	0.2852924	1.862574	1.081631
0	0.02778135	0	0.4427814	0	0
1.54904	1.357998	3.460162	0.8134614	7.522766	5.123679
1.453389	2.034866	2.14313	0.5874718	2.587071	2.532415
0.003365158	0.004375705	0.006878825	0	0.006369855	0.02589365
4.398152	0.5254736	1.54544	0.3182515	0.9370624	0.9276795
7.599861	0.6410841	0.7620073	0.5382956	0.4097181	1.091839
0.7476781	0.7082278	0.2732816	0.04104651	0.3842781	0.9296413
1.478708	1.241782	0.9319911	0.8376325	1.457825	1.355892
0.009828079	0.01916913	0.02008989	0.01222077	0.06046111	0.02268704

TCGA-DU-7010-01	TCGA-P5-A735-01	TCGA-HT-7479-01	TCGA-FG-8182-01	TCGA-27-1835-01	TCGA-S9-A6TZ-01
1.573027	1.277495	2.675578	1.829712	1.437817	1.614086
2.387028	1.191729	0.8241289	0.9371062	1.992937	3.407443
3.454335	0.8983126	4.195677	1.517554	1.936075	2.669047
0.5056478	0.5267456	1.052025	0.7525769	1.683138	0.695971
1.515421	2.277951	2.309531	2.813897	4.940565	2.150902
0	0	0.02124914	0	0	0.03068011
0.3658115	0.2386139	1.157825	0.9656035	0.6577379	0.5880537
3.139983	4.240942	10.28997	7.618536	1.343146	5.853238
0.1416987	0.2492745	0.3504327	0.2660921	0.04054681	0.3495151
0.1139087	0.02096353	0.07561573	0.02224099	0.08126958	0.1122088
0.9106753	1.474314	2.449899	2.44805	0.830854	1.496986
0	0	0.05528271	0.01951249	0	0.02660625
3.857699	3.529703	9.835925	5.268318	0.6060357	6.30203
2.732877	3.064986	5.225808	5.649441	0.6923617	4.132641
0.002623364	0	0.002902436	0.003073316	0	0.03142965
1.212892	0.222252	0.6361045	0.2152915	1.236219	0.775839
0.8905646	0.1552715	1.908375	0.7467913	3.155641	1.108136
0.7295461	0.2060138	0.9779811	0.8094546	0.3905656	0.5672812
1.244011	1.140306	5.733897	5.311839	1.813117	2.927107
0.09960135	0.04935116	0.06357512	0.0628302	0.007454038	0.06425409

TCGA-DB-A4XD-01	TCGA-P5-A737-01	TCGA-DU-8163-01	TCGA-S9-A7QZ-01	TCGA-S9-A6WI-01	TCGA-S9-A7IY-01
1.086877	0.9804898	3.30075	0.4271315	0.5557308	2.277567
0.8350912	1.889109	2.666447	0.882679	0.7297488	2.773921
0.3556095	0.8750888	4.650215	0.1671187	0.3727445	2.215844
0.4467725	0.7366926	0.9332888	0.3275793	0.9903261	0.8920229
1.668659	1.299449	2.954252	1.246474	2.579898	1.913842
0	0	0.07756523	0	0.02007004	0.02666926
0.4970323	0.2877892	0.6358271	0.2590976	0.3677518	0.5851205
2.588835	2.734464	10.70383	0.409208	1.583674	4.734901
0.05591796	0.1795471	0.39834	0.1059979	0.265661	0.3761612
0.02397403	0.02681619	0.3475787	0.01931417	0.007935542	0.1054482
1.365927	0.7952878	2.609591	0.3123492	0.4659502	1.490179
0.02629114	0.07057921	0.08968764	0.06354272	0	0.1156399
3.812337	1.983573	15.96953	0.715717	2.466712	4.673885
2.799888	1.630624	8.072889	0.8029234	0.7534447	3.954585
0.004140989	0.01111659	0.00706313	0.0133444	0.002741382	0.007285549
0.1036014	0.7639044	0.7669174	0.1769443	0.444433	0.9879233
0.192367	0.6753105	1.728904	0.453008	0.4800084	1.405851
0.170606	0.2835214	1.517338	0.09817511	0.2218526	0.4287998
2.054656	1.0923	2.489394	0.3787011	1.711551	2.827899
0.03023481	0.02164429	0.09798375	0.009743216	0.0200158	0.122347

TCGA-DU-A7TI-01	TCGA-TQ-A7RV-02	TCGA-S9-A7QX-01	TCGA-HT-A74H-01	TCGA-QH-A86X-01	TCGA-RY-A845-01
2.379318	2.357522	2.563628	2.44319	1.03187	0.9486439
14.53209	3.328796	2.274261	5.229788	0.9151663	1.179773
1.173439	4.572974	1.064705	2.304446	0.1996455	0.7377188
8.167499	1.059305	0.4824556	1.648208	0.4030127	0.5311096
2.123501	1.408838	2.096428	3.066068	0.8152664	1.980051
0	0	0.05446153	0.1047791	0.01702037	0
1.485742	0.5843496	0.8731815	1.149421	0.3529071	0.4833297
10.06372	3.122481	4.084016	5.294816	1.301638	2.83613
1.950824	0.4127953	0.1743137	0.4348363	0.1403467	0.2021521
0.04219168	0.1615763	0.01076684	0.2123229	0.0269189	0.09791498
1.879017	1.269773	1.923373	2.143963	0.5223996	1.466317
0.02313477	0.07332115	0	0.09086601	0.04428096	0.02045306
30.26774	7.304425	7.049976	5.149666	1.249324	4.153446
4.106248	3.079772	4.876142	3.637929	1.157	2.909139
0	0	0	0.003577965	0.006974477	0.006442922
3.059451	0.8051384	0.279167	8.192448	0.2768592	0.570621
1.158866	1.719475	0.2392417	4.065803	1.619974	0.2993019
1.688895	0.6910266	0.4159366	0.9634284	0.1505135	0.1943431
30.54228	6.822503	2.138339	5.325892	0.5618625	1.88742
0.1489879	0.06745546	0.02715717	0.03657357	0.006789747	0.02822522

TCGA-HT-8111-01	TCGA-TQ-A7RN-01	TCGA-DU-A5TW-01	TCGA-R8-A6MO-01	TCGA-DU-8162-01	TCGA-DU-6394-01
2.345311	0.4682482	1.727512	1.716699	1.238513	1.892783
2.503027	0.8505052	1.490777	2.099552	0.6376256	1.72508
3.634737	0.3447081	0.4681581	0.6773171	1.364731	1.565191
0.8209488	0.4476745	1.075565	0.7999587	0.7907045	0.5450054
1.720072	0.9657259	1.887425	2.048421	2.319062	1.202389
0.04063896	0.01959158	0	0	0	0.01779161
0.6025744	0.3448144	0.5990102	1.127663	0.3853973	0.3260033
4.425703	0.9159048	2.620854	1.862078	2.980023	1.639473
0.2270751	0.2210663	0.1883696	0.1253	0.7530128	0.05404968
0.1044442	0.01161955	0.01183563	0.03805209	0.04000248	0.2356614
2.546603	0.7863376	1.160215	2.116904	0.7126082	0.598578
0	0.06796047	0	0	0.035095	0.1080041
10.75834	2.842668	4.045229	3.438952	3.356166	2.348658
5.285883	1.323597	2.293349	2.157002	2.205552	1.418846
0.04440725	0	0	0.005258132	0.002763821	0
2.324776	0.3427856	0.1595772	1.257625	2.533536	0.2359012
2.281103	0.1912512	0.1168847	1.888342	0.6222052	0.3386761
0.7881766	0.1023756	0.1203225	0.2669211	0.6140718	0.2789097
1.66168	0.8770205	1.796642	2.998854	1.801464	0.5695246
0.04863494	0.01953863	0.06567657	0.03455232	0.04843111	0.04613318

TCGA-KT-A74X-01	TCGA-DU-8166-01	TCGA-DB-A75L-01	TCGA-P5-A780-01	TCGA-VM-A8CB-01	TCGA-WH-A86K-01
1.527547	1.734506	1.269655	1.857822	1.11866	1.499651
2.297928	2.502679	1.262213	3.523908	1.39476	1.980572
0.4210559	1.433746	2.023123	2.223596	0.6581727	2.387323
0.6597597	2.883231	0.7770748	0.9771023	0.3289018	0.8627285
1.000385	1.598589	2.724744	2.420517	0.879609	2.115666
0.03589625	0	0	0.04504587	0	0
0.757269	0.6466476	0.5918446	0.7819528	0.3490294	0.8322223
1.333787	7.83674	3.563934	4.948234	1.401057	3.009548
0.06815643	0.1295864	0.2480606	0.2639177	0.1205408	0.2119881
0.04967587	0.1035626	0.004757873	0.0267162	0.01996734	0.04506474
0.9075311	1.195562	1.879811	1.36485	0.3908065	1.255758
0	0.01817153	0.02087091	0.01953223	0.08758879	0
0.6638177	16.41184	6.128073	8.695434	1.743244	2.603354
0.9000487	3.631027	3.147926	3.115435	0.916836	2.107029
0.004903097	0.008586325	0.006574548	0.006152851	0.00459856	0.00444797
0.699209	0.7246499	0.4770079	0.2278241	0.7225089	0.3872601
1.77836	0.3681898	0.1527082	1.000394	0.6573021	1.017244
0.2525055	0.7622492	0.2998885	0.4390868	0.1150279	0.3141488
1.140092	7.1739	2.371362	3.492238	0.7324988	1.02612
0.01431969	0.2549466	0.03360216	0.09883308	0.02014542	0.03247619

TCGA-TQ-A7RV-01	TCGA-DU-6405-01	TCGA-CS-6667-01	TCGA-HT-7472-01	TCGA-FG-8188-01	TCGA-DU-A7T6-01
0.7872756	2.204159	1.316161	2.552232	1.578161	1.090997
0.85031	3.129116	0.6511575	1.295463	2.171279	1.79525
0.5657659	1.78773	1.627256	2.210141	0.8615839	0.6098022
0.4712071	1.731944	0.4375966	0.6058311	0.5837971	1.108938
1.419812	3.794077	2.544436	1.747172	2.719629	1.498896
0	0.04109911	0.1872833	0.02055502	0.06295934	0.0183485
0.3372385	0.6787596	0.5167575	0.6938074	1.115682	0.4644971
2.196244	5.314336	1.664496	9.29284	5.920714	1.110666
0.2590425	0.1739069	0.309312	0.2430883	0.2390826	0.1612521
0.0238388	0.1421899	0.02468347	0.8086662	0.006223412	0.09068575
1.015405	0.9662947	1.330605	1.880529	1.511226	0.8158652
0	0	0.0180461	0.1604306	0	0.0477363
2.634715	4.168486	4.410615	6.666672	4.469069	1.743546
2.450675	3.439304	2.340688	5.278438	3.845689	0.9661329
0.006588211	0.008420639	0	0.01965338	0.004299833	0.005012473
0.5340404	2.036495	0.2872903	1.550958	0.8390886	1.259063
0.3413647	0.9628934	0.7312966	1.725643	0.3841269	1.352328
0.3102057	0.6071184	0.2467529	1.652462	0.2404183	0.2433875
1.067521	2.2458	1.883876	3.413289	3.054563	0.8718493
0.04329256	0.01639521	0.1411205	0.06149839	0.04395242	0.0219587

TCGA-HT-7877-01	TCGA-CS-5397-01	TCGA-HT-7856-01	TCGA-DB-5275-01	TCGA-TQ-A8XE-01	TCGA-HT-7884-01
0.8262593	2.952701	0.6906531	1.812734	1.811716	0.6491994
0.8863852	2.246659	0.7791047	2.134835	1.471402	0.9072826
0.3557337	4.201616	0.5882422	0.8378469	1.162955	0.5497936
0.5328255	1.437784	0.4733192	3.003599	0.7558385	0.4619401
1.704829	3.12646	2.456722	1.180494	1.865065	0.8934763
0	0	0	0	0.02428049	0
0.3186428	0.650964	0.3083179	1.047699	0.5327112	0.3146091
0.921333	6.502401	1.026556	2.090033	2.959161	1.678008
0.1371594	0.2952869	0.3244245	0.1643691	0.1001061	0.2880395
0.03029356	0.1110262	0.0334609	0.01970404	0.0240008	0.0133435
0.7310936	1.546908	0.503205	1.270694	0.9124311	1.004813
0.01661073	0	0.01630886	0	0	0.01951089
2.937224	12.03242	1.47135	6.79407	4.130903	1.766631
1.159301	5.725812	0.9614069	2.403246	2.288072	1.653169
0.002616275	0.002950362	0.005137457	0.005445506	0.003316491	0.02765758
0.7330997	1.139683	0.6426579	0.4441375	0.1692663	0.2798561
1.028393	1.054286	0.798584	1.255108	0.6162619	0.2635519
0.1116384	1.541118	0.07559281	0.3365269	0.4343113	0.1175648
0.9005482	2.290377	0.9500256	6.545438	2.307428	1.389743
0.2406895	0.08185819	0.04876349	0.08747101	0.03874378	0.02243752

TCGA-WY-A85D-01	TCGA-S9-A7IQ-01	TCGA-HT-A614-01	TCGA-E1-5322-01	TCGA-FG-5965-01	TCGA-HT-7860-01
1.758685	0.5356046	1.54088	3.184839	3.085427	2.965284
1.954977	0.3139438	1.78173	3.675195	2.32444	7.161883
2.423436	0.1614586	0.5167561	10.71829	1.59898	4.319783
0.939748	0.4136764	0.6693979	1.95213	1.363647	2.229569
1.196657	2.236917	1.912053	2.200408	2.004304	4.730763
0	0	0	0.0191699	0	0
0.9002168	0.1841859	0.5698151	0.9613384	0.8749124	1.009987
3.55166	0.4686791	2.689131	10.21554	2.261043	4.584093
0.2398256	0.2822617	0.4048818	0.18303	0.2896825	0.2572221
0.005905306	0	0.01229577	0.02273891	0.0780606	0.2548225
2.062816	0.3290455	1.443938	2.625055	1.472335	1.684626
0.02590424	0.01790558	0	0.01662443	0.03261152	0.03992161
5.74483	1.497919	2.565734	23.64261	12.14301	8.303419
3.863406	0.7607321	2.456149	6.876091	2.852243	4.971271
0	0	0	0	0.03081888	0.01571964
0.3429781	0.3753672	0.4505844	2.557483	1.819661	2.894557
0.3499128	0.1814006	0.4553582	0.6362583	4.267483	2.550244
0.3301874	0.07884406	0.2062505	1.479466	0.3816711	1.272148
2.599624	0.3201404	1.290989	4.755747	7.777444	4.938849
0.03574785	0.0247097	0.0372163	0.2791242	0.4537893	0.1010017

TCGA-DB-5279-01	TCGA-FG-5965-02	TCGA-S9-A7J2-01	TCGA-E1-A7YO-01	TCGA-HT-A5R7-01	TCGA-HT-8012-01
2.075364	0.2469881	0.9553496	0.5706167	0.8970253	0.8836176
0.6018926	0.5022514	1.422855	0.9082375	3.151602	1.205121
0.6444471	0.2284992	0.6745054	0.2769749	0.5308769	0.2905233
1.016813	0.8709066	0.2856471	0.5843493	0.8343381	0.4068325
1.164759	3.247162	0.9744101	1.588325	1.618678	1.846426
0	0	0	0.02575955	0	0
0.3360737	0.1479441	0.2792601	0.4036869	0.4000991	0.6067042
1.30312	0.1895096	1.917985	0.5916867	1.736705	1.959729
0.1021879	0.1521761	0.1390404	0.1732805	0.405932	0.2579771
0.1079956	0.005776752	0	0.02546283	0.04771998	0.003917226
0.4299096	0.2414593	0.6885185	0.4206546	1.27632	0.7523967
0.3430488	0.02534032	0.04274396	0.04467814	0	0.06873328
4.351633	0.3657838	3.042945	0.9453973	4.128985	2.249259
1.18471	0.3622595	1.440106	0.7660913	1.559302	1.718162
0	0	0	0	0.01236387	0.01082584
2.044435	0.363471	0.2189648	0.9225344	1.039335	0.5741947
4.404026	0.3137709	0.1443457	0.4903514	0.4270851	0.4738937
0.2309369	0.04110902	0.1584972	0.06212597	0.2850122	0.1314159
3.745106	0.3288403	0.9244776	0.7343959	2.34671	0.8423961
0.3343909	0.005828274	0.03932445	0.01541396	0.0120364	0.01185649

TCGA-CS-4938-01	TCGA-DU-5853-01	TCGA-HT-7686-01	TCGA-DU-A7TA-01	TCGA-HT-8104-01	TCGA-HT-7677-01
1.89498	4.042126	4.777078	2.104985	1.981228	0.9487111
0.6888909	3.679716	7.148144	4.7744	3.107524	1.387853
0.7307242	2.259307	11.8597	2.079305	1.193043	0.6589191
0.7464435	0.8474485	1.111918	0.9218776	1.747364	1.447852
1.096915	3.294671	3.104537	2.310005	2.351582	0.8453821
0.06795969	0	0.0227634	0.07878525	0.05633192	0.01925993
1.196099	0.7164789	1.262287	0.6774862	1.122737	0.7151026
6.586839	6.271474	12.3335	4.42836	4.597229	2.076645
0.1818786	0.3915173	0.5384104	0.4103041	0.2342885	0.1295585
0.008956916	1.268257	0.1260067	0.08826143	0.09651727	0.04188376
1.738217	1.573073	3.1037	1.684421	1.259787	0.507772
0.01964522	0.02045345	0	0.06832376	0	0.033405
5.252598	7.542104	22.44668	10.91592	6.96088	3.572635
3.537367	6.800715	10.67481	3.722224	2.772577	1.287776
0	0.2416142	0.003109271	0.03228402	0.005129614	0
0.20437	2.98534	1.6398	1.155906	1.419389	0.4844127
0.9730102	3.844952	2.66657	1.948372	2.40126	1.720324
0.4826026	1.810743	3.051523	0.6967088	1.056684	0.2554773
2.787292	4.022642	4.668079	9.050239	1.760969	1.859213
0.02259201	0.2822576	0.3904723	0.08381047	0.03745312	0.1037225

TCGA-06-2565-01	TCGA-28-5209-01	TCGA-DU-7015-01	TCGA-FG-A4MT-01	TCGA-E1-A7Z4-01	TCGA-P5-A5F1-01
3.324189	4.137437	3.19744	1.008079	2.603697	2.474184
8.745845	5.58789	2.273381	1.176998	4.028969	10.77698
3.136239	5.256243	5.732784	1.882941	3.454366	1.923648
3.625121	4.27893	0.8131612	0.630678	1.028089	1.629296
3.390688	3.098396	1.938459	1.319224	1.55595	2.046442
0.05392408	0.07645481	0	0	0	0.05874508
1.027077	0.5468479	0.9625418	0.3799883	0.6271846	0.6585936
6.040069	3.388311	10.63575	2.697393	3.727069	3.57975
0.4124692	0.160373	0.3756591	0.1330003	0.1996997	0.1211002
0.309157	0.09572724	0.4819429	0.00865512	0.02911026	0.09290944
1.48532	1.168285	2.707478	1.00779	1.805832	1.392733
0.04676377	0.02210092	0.03063902	0.07593317	0.02128253	0.1783062
4.287962	11.90538	21.40485	3.188607	5.781136	2.908085
3.321964	2.025273	8.324622	2.241179	4.317618	2.068269
0.003682766	0.01044302	0.01689031	0	0	0
3.644954	5.218412	0.5602078	0.4129209	0.8218747	1.132227
2.868889	0.9329307	2.336641	0.1923188	0.3473754	2.809973
1.414318	0.6402466	1.217772	0.2287714	0.6954554	0.5253941
3.533224	5.946376	2.067527	1.291945	2.853886	2.18165
0.129068	0.1779124	0.2114093	0.03492926	0.03915986	0.04101042

TCGA-HW-7489-01	TCGA-HT-A4DV-01	TCGA-RY-A843-01	TCGA-QH-A6CW-01	TCGA-E1-5311-01	TCGA-CS-6290-01
1.357694	1.310327	0.7287161	2.290851	0.937846	4.8166
0.6890072	1.202159	2.026513	3.082567	0.6464916	14.70517
0.9563843	0.6418739	0.5312724	0.610178	0.3330212	10.17558
0.5388531	0.7276624	0.6731676	1.855817	0.3353416	1.692788
1.926489	2.049985	1.018634	2.495823	1.031887	3.130425
0	0	0	0.02837425	0	0.02334074
0.2820466	0.5114925	0.4664992	1.935993	0.4015741	1.356203
1.977235	1.965595	1.938726	4.769243	1.273573	14.84759
0.2353884	0.180855	0.4717562	0.2370472	0.2998214	0.5495335
0.01681991	0	0.01719199	0.03365689	0.01496747	0.1891895
0.891362	1.282023	1.43292	1.953897	0.5734823	3.995244
0	0.04380501	0.01885362	0	0.03282818	0.06072433
3.291929	2.931659	3.426605	7.006975	1.976252	13.01539
1.620679	1.650001	2.098677	3.341815	0.9662919	8.692392
0	0	0	0.003875661	0	0.02231691
0.5553178	0.4971329	0.5854329	0.6089296	1.974047	1.349578
0.2803067	1.577907	0.350176	0.8448096	0.3787725	2.130399
0.1538939	0.2436475	0.1966229	0.2908361	0.1065128	2.298606
1.239391	0.8084703	1.859436	7.883619	1.382165	3.72411
0.05515225	0.0201503	0.013009	0.06791416	0.04907812	0.09311065

TCGA-DU-7301-01	TCGA-WY-A85C-01	TCGA-S9-A6WN-01	TCGA-S9-A6U5-01	TCGA-HT-7476-01	TCGA-06-5417-01
2.54288	1.917177	2.852491	4.158046	1.475455	1.795711
3.984107	1.86659	9.652374	6.653762	1.693737	1.653854
2.743392	1.040751	5.773994	0.9186477	0.3858648	3.613303
2.820748	0.752211	1.521019	1.396551	1.1035	1.632215
1.602976	1.693809	3.522607	1.401907	2.762456	1.190169
0.04351297	0.07098163	0.07508897	0	0	0.2240325
1.563141	0.4620647	0.621563	1.103879	0.4758705	0.5941509
5.6441	1.771084	4.423513	2.656867	1.86994	2.572969
0.1841209	0.2156372	0.4209266	0.1516431	0.2947694	0.1944557
0.1247341	0.08419681	0.6977059	0.4239975	0.0688599	0.02214519
1.832069	1.010168	2.378545	1.370194	1.210687	1.035829
0.05660266	0.02051878	0.04341219	0.06269357	0.02013741	0
21.3795	4.61107	28.2063	1.693395	3.032326	5.385848
4.411085	2.249426	4.841113	2.317838	2.010307	1.786615
0.08915207	0.006463625	0.00341882	0.01645759	0.01585872	0
1.046826	0.6080309	7.256691	3.340073	3.910484	1.148379
1.051305	0.9007907	1.930263	6.304408	1.416744	0.546746
0.4241448	0.0855955	1.338105	1.017063	0.3406852	0.3376961
19.71262	2.786973	8.681975	1.566879	1.631812	2.465193
0.2299955	0.09910571	0.2596049	0	0.01389481	0.0670281

TCGA-DU-6395-01	TCGA-HT-8019-01	TCGA-E1-A7Z3-01	TCGA-DB-A4XE-01	TCGA-P5-A5EZ-01	TCGA-S9-A6WO-01
3.385825	0.6214252	1.47915	1.296114	3.383082	2.357115
1.923267	0.3535807	1.222335	1.171935	2.023223	1.547765
1.542016	0.1289096	2.653312	1.351002	1.912188	2.215566
2.646044	0.4840496	1.646397	0.5273616	1.084045	0.4818782
1.106238	3.158467	3.10403	0.9681853	1.89904	3.299717
0	0	0	0	0.05216628	0.02798274
2.909358	0.1854751	0.4054702	0.4708638	0.7357643	0.8568148
5.159877	0.7008759	2.375975	2.817429	4.031722	5.762087
0.1533761	0.2146282	0.7074618	0.3585926	0.1811171	0.4493364
0.0445533	0.004345331	0.03499779	0	0.7992634	0.01106416
1.624438	0.4367724	1.248069	1.318266	1.719151	2.075586
0.01776706	0	0.02558691	0.02605648	0.1130984	0.02426705
10.77152	0.8191853	7.01752	4.68442	3.48774	9.266795
3.281943	0.705732	2.710505	2.900458	3.257276	6.326349
0.002798402	0.006004487	0.0120902	0	0.01425087	0.007644368
0.688917	0.6549739	1.141354	0.4558846	1.536668	0.5125521
1.529977	0.1609238	0.5472417	0.19065	11.1906	0.5463296
0.6711662	0.03534013	0.391371	0.2355088	1.761379	0.2811991
7.521457	0.5496822	1.933212	0.6086408	2.465692	3.268092
0.02860497	0.01315226	0.06473488	0.07191587	0.06763287	0.106047

TCGA-S9-A7J3-01	TCGA-RY-A847-01	TCGA-E1-A7YD-01	TCGA-DU-6407-02	TCGA-TM-A7C5-01	TCGA-VM-A8CA-01
1.718455	1.982492	2.07979	2.484317	0.9278897	1.980806
2.350082	2.951493	4.437147	9.487453	0.6625541	1.159632
0.2712039	2.196664	1.779016	4.179973	0.2766053	2.616016
1.180109	1.165936	2.874869	0.775508	0.2987068	1.429014
1.703506	2.604302	1.580001	1.798759	1.022745	2.650265
0.0173407	0	0	0.0872706	0	0
0.7859908	0.7224138	0.7661531	0.8907246	0.7248917	0.5727447
2.549178	5.48981	1.452036	5.148997	1.481588	2.137132
0.1298182	0.1690503	0.1598526	0.2777468	0.09594468	0.6101017
0.01371276	0.04738941	0.02855606	0.06901227	0.03496468	0.03566897
0.7847002	1.969014	1.074239	2.123391	0.4175627	1.413996
0.01503812	0.05196965	0	0	0.03067524	0.07823282
2.772613	9.198157	2.3424	9.600439	1.338441	4.825101
2.202956	3.593689	1.422537	3.586199	1.010089	1.815827
0.00236858	0.004092742	0	0.007946906	0.01449452	0
0.8746534	0.4628225	11.43988	3.125448	0.7832839	1.200236
0.482443	0.7020027	2.058673	1.221096	1.346667	1.658532
0.2788112	0.7587823	0.3657835	1.08746	0.135073	0.2538306
1.756341	2.203066	3.149949	2.044279	1.910742	3.301358
0.0518815	0.07171811	0.04609719	0.04641852	0.03527653	0.0359871

TCGA-DH-A7UU-01	TCGA-HT-8108-01	TCGA-HT-7687-01	TCGA-HT-8564-01	TCGA-HW-8320-01	TCGA-HT-8113-01
1.94349	2.889387	1.418398	2.885359	1.941063	0.8615684
1.343423	3.113318	1.713748	2.854254	0.9799651	0.5177683
1.702601	6.352571	0.4779561	2.871287	1.884367	0.2716523
0.7931402	0.5809396	0.5898811	0.8693667	0.9358512	0.748821
2.569335	2.345983	1.415411	5.188923	1.480482	1.945053
0.02721595	0	0.02328409	0	0.02190652	0
0.7021023	0.7266646	0.7410137	0.373621	0.6179485	0.1400981
2.853579	7.222067	2.246271	4.050169	5.173917	0.4505986
0.3336735	0.324455	0.2046275	0.5972555	0.2020282	0.3974651
0.01076098	0.09310208	0.2439684	0.1920486	0.05197003	0.02996822
1.1459	2.066213	0.9528662	1.301484	1.551619	0.3877173
0	0.0204201	0.1211539	0	0.03799533	0
5.133617	4.883655	3.126711	3.44153	9.629157	2.256978
3.332398	5.951582	1.952541	2.841688	3.793053	0.6352327
0	0	0.06678824	0.009477768	0.005984457	0.006901805
0.569191	1.728416	1.085317	3.737007	0.5000724	0.8633647
0.3719514	0.9194446	2.76166	2.890067	0.5667008	1.899051
0.2133251	0.7903167	0.4586053	1.08776	0.5855698	0.04062141
3.328278	1.360286	2.131216	1.613819	5.423701	1.377384
0.04342784	0.1596852	0.06501925	0.009226735	0.113606	0.161256

TCGA-FG-8189-01	TCGA-DH-A7US-01	TCGA-S9-A7R2-01	TCGA-HT-A74J-01	TCGA-26-5133-01	TCGA-DU-6404-01
0.4665033	1.152438	5.037394	1.323373	1.670341	1.983481
0.7352861	1.607683	18.53298	1.995474	2.696723	4.59773
0.0795501	0.6233653	3.187475	0.9421708	2.596634	1.737564
0.3593468	0.6166799	1.23697	0.5880015	1.541983	3.097972
2.060129	0.9997091	4.368305	2.107429	1.79881	1.929653
0	0	0.02964454	0	0	0.01795544
0.09156534	0.5535142	1.636716	0.3180043	0.6404635	0.6363652
0.9603201	2.254113	9.701157	2.289379	2.13743	1.203324
0.3943979	0.1992716	0.5789445	0.4017994	0.1036289	0.1909158
0.005363008	0.02017212	0.4805703	0.005014585	0.1003133	0.1064917
0.3469227	0.767881	3.353682	1.102905	0.9630847	2.335107
0	0.04424355	0.05141638	0.065991	0	0.03114246
1.034192	0.8200835	7.885745	1.883491	3.515589	8.255128
0.8634569	1.538599	6.567844	2.623478	1.673484	1.697033
0	0	0.01619668	0.01385856	0.02038463	0.007357643
0.4931792	0.7252698	8.643281	1.02976	0.6609512	3.129315
0.1986122	0.7470489	11.53208	0.5571264	1.252894	2.278631
0.06542519	0.2255795	1.459703	0.2548944	0.3539297	0.5340864
0.2713671	1.282261	8.592416	1.573169	3.0455	5.06066
0.01082168	0	0.0354773	0.03541517	0.02381365	0.1181856

TCGA-E1-5319-01	TCGA-TM-A84M-01	TCGA-TQ-A7RK-01	TCGA-TM-A84T-01	TCGA-CS-6666-01	TCGA-DU-7019-01
1.807301	0.3033381	0.8177397	1.395076	2.11264	1.81312
1.306041	1.086337	1.824141	1.673693	3.632852	1.01475
0.4381476	0.1873951	0.2986779	1.344743	0.719158	1.441171
1.13805	1.224414	0.473753	0.6976214	1.421266	0.9965823
1.392328	0.5581289	1.441834	2.141627	1.765599	1.751904
0.02490225	0	0	0.02866083	0.02724904	0.02019683
0.6904455	0.2311063	0.4219244	0.7601065	1.136555	1.31961
1.608315	0.8437042	2.582225	5.3828	3.622855	4.393531
0.06214213	0.1366873	0.1054845	0.158591	0.07095486	0.2257045
0.09353852	0	0.005491607	0.08499206	0.1292888	0.1317636
0.9798911	0.1293217	1.169564	1.697323	1.297406	1.557681
0.107978	0.1187538	0.192716	0	0	0
0.9564421	0.3350469	2.924079	5.968789	4.008004	8.998318
1.368453	0.4558931	2.22298	4.020338	2.586792	3.160827
0.03401417	0	0.003794219	0.003914805	0.007443935	0.005517401
0.7352505	0.3331837	0.1746634	0.2507331	1.627705	1.695097
1.470715	0.1069413	0.2847245	0.6994607	2.513724	2.050459
0.2051994	0.03302598	0.2065649	0.5702662	0.5366992	0.462745
1.961717	1.739689	1.333796	2.171793	2.609979	4.712507
0.01490097	0.02731337	0.01662176	0.04001671	0.04348063	0.09265432

TCGA-TQ-A7RR-01	TCGA-E1-A7YV-01	TCGA-HT-7477-01	TCGA-HT-8011-01	TCGA-HW-8322-01	TCGA-HT-7879-01
2.167362	1.584766	1.719922	2.038848	1.659115	1.297866
3.091967	1.571052	6.346356	1.760314	1.95732	2.238179
3.261861	0.4067401	1.097452	0.5909008	0.6908421	0.7461565
0.9006093	0.9002976	1.534793	1.969756	0.464942	0.6460254
1.560647	1.444726	1.559911	1.334976	1.626951	2.411732
0.05255117	0.08322189	0.0522201	0.02158973	0.01910151	0
0.5067986	0.3344101	0.8813101	0.9213262	0.3131624	0.9508763
4.736013	0.9670316	4.565061	1.633578	2.838998	4.824639
0.5730179	0.2106852	0.1331451	0.1288337	0.1388552	0.2139541
0.01558375	0.02193687	0.01032372	0.02134104	0.07552592	0
1.158003	0.5075847	1.42811	1.609888	0.6426479	1.631188
0	0.1683996	0	0.01872294	0.1159558	0.01838844
8.955618	3.10956	4.241605	1.861128	4.896422	3.420483
3.859609	0.8713794	2.356824	2.369833	1.760119	3.845682
0.01076701	0.007578229	0.01069918	0.008846873	0.02869999	0.005792545
0.2909246	1.289253	0.8851205	1.103727	1.067908	0.3652007
0.2564997	1.96331	1.197954	1.433147	1.062863	0.3829343
0.5492112	0.1393831	0.3515897	0.3427898	0.3839029	0.3963279
3.311845	2.122877	2.024215	1.031259	0.8885212	2.34914
0.03144548	0.2268584	0.1145737	0.0344502	0.1828789	0.05921077

TCGA-HT-7676-01	TCGA-FG-A87N-01	TCGA-TQ-A7RW-01	TCGA-FG-A6IZ-01	TCGA-S9-A6WH-01	TCGA-DU-6392-01
4.006376	1.456949	2.968865	0.8064902	0.9013048	2.478422
4.325597	4.3264	1.779954	3.06648	1.222127	5.067963
11.3094	3.36828	2.187896	0.1575542	0.952793	5.27813
0.7900514	1.799776	0.6821589	0.5382305	0.4563717	1.506003
3.246785	2.323	2.638657	1.810832	1.182607	2.124766
0.04944408	0	0.03243905	0	0.03361175	0.1076355
0.8881035	0.5333474	0.758635	0.4404242	0.4538081	0.7958204
11.31035	3.558026	7.039119	0.5880157	1.208029	6.006344
0.3272381	0.1523167	0.4293858	0.1428187	0.2953901	0.2102073
0.02443727	0.02523094	0.2950016	0.06904162	0.04651442	0.1028491
3.083776	1.238753	1.991278	1.511627	0.6414639	2.431827
0.04287865	0	0.08439489	0.06989036	0	0.09334311
23.89276	14.29993	7.309365	4.409927	1.874276	12.27455
6.830632	3.77335	7.225385	1.075849	1.299267	4.308101
0	0.002905395	0.070894	0.0293549	0	0.02205302
0.3818614	1.631134	3.715824	1.057559	0.8315967	2.344257
1.448005	0.6021661	1.504168	1.088308	0.2132746	2.477965
0.9738545	0.5087266	1.147452	0.1079824	0.1148402	0.998706
1.323077	3.989626	3.342354	1.444424	1.109564	3.719169
0.2465522	0.1230372	0.04529192	0.01071652	0.1072669	0.1502824

TCGA-28-5220-01	TCGA-FG-A6J1-01	TCGA-14-1034-02	TCGA-HT-7880-01	TCGA-HW-7486-01	TCGA-FG-A60J-01
4.647371	1.123026	2.539118	0.8543651	1.419264	2.010162
11.39268	2.942091	3.66318	0.6774491	1.375679	3.420857
9.150649	0.8758667	2.427065	0.4891865	1.871336	4.494242
1.790101	1.963413	3.261216	0.4883202	0.4927246	1.019171
4.334169	3.553252	1.612652	1.917722	1.504182	1.330425
0	0	0	0	0	0.02471918
1.084826	0.7849227	0.3043927	0.2930279	0.9181183	0.5780945
5.997949	0.9362676	2.843116	1.60837	5.353356	4.148206
0.2752199	0.2365647	0.4520379	0.416932	0.2167432	0.3298822
0.8835698	0.07085769	0.1331185	0.04240203	0.02468335	0.043982
1.806191	2.121406	0.6623983	0.843971	1.273281	1.517393
0.05237663	0.07770615	0	0.01550008	0.09023008	0.02143685
17.35476	6.73006	3.184843	1.332277	6.151152	6.371603
4.183406	1.722472	1.099172	1.673095	3.189987	2.973314
0.008249586	0.00815941	0.01149665	0.004882682	0.01136936	0.05064619
2.138226	0.7879664	3.681654	0.6278894	0.1450667	1.256956
2.741563	2.361712	0.739481	1.596477	0.954744	1.628818
1.068187	0.2881392	0.5328613	0.2263087	0.6189705	0.630945
1.570838	4.474261	4.220379	0.7330574	1.451931	4.685872
0.1626294	0.06553219	0.1846704	0.04278021	0.07886109	0.02465237

TCGA-DB-5280-01	TCGA-FG-A60L-01	TCGA-DU-A6S2-01	TCGA-FG-A4MW-01	TCGA-32-1970-01	TCGA-HT-7873-01
1.128764	2.539331	1.483123	1.519708	2.685189	1.955432
0.7073624	4.956353	0.8438727	3.792534	7.714487	1.894197
0.5060792	1.708995	0.1281924	0.5386604	3.85144	1.455017
0.7818396	0.9126535	3.761096	1.417841	1.478506	0.4998922
1.666514	4.345995	1.110164	2.488227	3.90547	2.384576
0.02070947	0	0	0	0.08503939	0
0.5492307	0.8930634	0.6576718	0.6029861	0.7381008	0.606561
4.454683	3.211133	1.977524	2.531566	2.589109	5.450168
0.152791	0.6140905	0.4581719	0.3081462	0.2337396	0.3298283
0.06960114	0.03515029	0	0.04190161	0.1176838	0.02901326
1.201989	3.47484	0.3405933	1.459519	1.701015	1.559198
0.0538787	0.07709519	0	0	0	0
2.816329	11.62599	3.52964	3.931572	6.185583	7.479699
2.645348	3.991629	0.694194	1.64154	2.906171	3.961543
0.02262979	0	0	0	0.007743732	0.008590982
0.3340366	1.288099	0.6561105	2.468396	2.041985	0.2865782
0.5862753	2.184041	1.369844	0.9724443	2.089202	0.2660591
0.357949	0.3632978	0.2108611	0.3578205	0.9229275	0.2486027
1.921455	5.773031	2.472927	1.049505	2.856874	1.431364
0.0289149	0.06501695	0.09940108	0.008455064	0.0961175	0.2049042

TCGA-DU-8168-01	TCGA-HT-7475-01	TCGA-14-1825-01	TCGA-FG-6691-01	TCGA-TM-A84Q-01	TCGA-12-3653-01
0.9459544	1.38518	3.718914	3.104731	3.310012	5.510453
1.067494	0.4989095	4.721892	1.707143	5.708113	17.00942
0.4375714	0.5383079	4.665065	3.478056	15.72403	5.225404
0.4076767	0.5808978	3.241619	0.7159539	1.034406	5.454919
1.495387	1.534756	1.917028	2.941909	2.296701	3.694223
0.03197507	0.02039659	0.09739848	0.02252009	0.04462201	0.08692314
0.2659645	0.1426095	1.056715	1.531131	0.5648088	0.7439723
0.8813458	0.9755859	3.790088	14.29512	6.945544	6.048494
0.1977453	0.1128619	0.2007823	0.3396289	0.3413171	0.1744724
0.01896405	0.0564526	0.03851062	0.01780855	0.1235024	0.3007266
0.3208418	0.4574796	10.92286	4.873849	2.058739	1.530621
0.06932315	0	0	0.07811904	0.03869688	0.03769053
0.3274918	3.23801	9.975647	20.68829	20.15979	13.31703
0.7605305	0.8878754	2.320025	10.51679	5.866114	2.388344
0	0.002785986	0.01330373	0.003076037	0.03047478	0.00593645
2.196295	1.070611	5.312123	0.283205	0.7654836	3.932841
0.319941	1.174746	1.426192	0.9562996	1.491916	2.227405
0.02891863	0.2172637	0.5089548	0.420927	1.69498	0.7599383
0.4118185	0.6937191	2.313998	4.798405	2.906993	6.369277
0.02551092	0.2034146	0.1165623	0.06737768	0.3426609	0.1993829

TCGA-41-5651-01	TCGA-HT-7691-01	TCGA-E1-A7YI-01	TCGA-RY-A83Z-01	TCGA-DB-5281-01	TCGA-VM-A8CH-01
2.287768	2.940285	1.22958	1.784892	2.106054	2.809403
3.799337	3.483859	3.051463	4.980997	1.20092	5.449754
1.409877	4.540582	0.8386924	5.359095	1.378552	2.881264
2.343849	3.971235	3.567574	1.422769	1.006162	1.09329
1.092554	2.343947	2.150396	2.797832	1.598222	2.079345
0	0.0797455	0.02383366	0	0	0.02947635
0.768281	1.307399	0.8734286	0.6183993	0.4449667	0.6253878
1.586267	2.952306	1.558622	2.105362	3.754622	4.173877
0.1492131	0.2682178	0.2249727	0.2191524	0.2418011	0.2174715
0.05305078	0.06306154	0.0942365	0.01267694	0.07572682	0.01165473
1.564088	2.32208	1.261399	2.20151	1.06879	1.693425
0	0.03457825	0	0.0556087	0	0
3.893588	24.05475	7.580869	7.926694	4.676135	7.841004
1.434453	3.672707	2.197825	3.099487	2.524781	3.956123
0.0229084	0.002723125	0.009766376	0	0.02378209	0
2.485111	1.098232	2.287025	2.015983	1.099547	0.535881
1.817318	1.595859	0.7794175	0.9858959	1.291745	0.5323288
0.4247151	0.6851663	0.3113564	0.5541637	0.3114385	0.5331751
1.64417	11.93098	4.202106	1.747952	1.0189	3.464645
0.06690492	0.111342	0.07130774	0.03837	0.1458588	0.07643138

TCGA-TM-A84F-01	TCGA-VV-A86M-01	TCGA-QH-A65X-01	TCGA-WY-A859-01	TCGA-DU-7304-01	TCGA-DB-A64X-01
7.444882	2.600236	1.847217	0.7232577	2.841877	1.142755
17.15946	3.811926	2.59938	0.5421825	1.632015	1.876246
9.643833	1.666066	0.296407	0.2877768	2.311687	0.4289989
2.86136	1.3751	1.430993	0.7869381	1.14386	0.5329199
3.974586	2.065526	2.894393	2.076822	2.435413	2.053802
0.09079124	0.03626477	0.1819409	0	0	0
1.503083	0.6382647	0.8188231	0.2129417	1.170371	0.3950359
14.22188	3.439749	0.5572147	0.6478253	8.481807	1.043672
0.7059613	0.302967	0.148051	0.3148598	0.360126	0.2380875
0.9393355	0.05735526	0.02997419	0	0.1189354	0.05784341
3.036702	1.897911	0.6085391	0.7240453	2.057096	1.036185
0.1312259	0	0.05259395	0	0	0.05074723
13.16487	9.058699	0.2846947	1.603392	7.129648	1.498354
7.29101	4.200409	0.7265272	1.174646	4.281154	1.220666
0.2728274	0.03962747	0.02485145	0	0.002833585	0
3.024009	2.701621	2.147098	1.672956	0.941447	1.347809
13.58986	1.221346	4.114607	0.1231698	0.8505476	0.4427125
4.202952	0.4373105	0.1279829	0.06762263	0.7421445	0.1999347
4.790861	3.292143	1.16785	0.8992939	3.667933	0.9073282
0.1328006	0.0144667	0.01814491	0.03355545	0.4965359	0.02917966

TCGA-FG-8181-01	TCGA-DH-A669-01	TCGA-QH-A6CU-01	TCGA-28-5207-01	TCGA-S9-A6WQ-01	TCGA-DU-7290-01
0.5652367	2.550808	1.798737	4.104106	3.552489	7.709067
1.2673	9.36413	1.657001	3.470343	3.268055	13.19515
0.3268279	2.508321	3.133293	3.018356	2.996717	12.52409
1.306578	1.588621	0.8631191	1.902032	0.8749451	7.987173
2.01504	1.197461	1.748036	4.71692	3.229996	4.538912
0.02229043	0	0	0.1235154	0	0.1083975
0.2257152	0.8449151	0.4622541	1.727198	0.9013957	1.855542
1.216759	5.749336	2.080538	4.848103	7.36857	16.30411
0.2587748	0.1101196	0.1787682	0.2646719	0.4407969	0.5268865
0.01762693	0.09086122	0.02369006	0.1770343	0.05420287	0.3814501
0.5876687	0.9080197	1.933263	2.035238	2.525502	3.536008
0	0.1494646	0.05195945	0.0803358	0	0
3.246944	3.007422	3.520013	6.632762	8.190296	36.11292
0.889585	1.9033	2.341073	3.681961	5.428421	9.204681
0.003044667	0.03662	0	0.02952436	0.006808989	0.05034075
0.9414992	1.355944	0.6019607	8.163202	1.819347	5.274878
0.7833494	3.383624	1.242886	3.873457	0.7664368	7.386001
0.1209584	1.170028	0.09031351	1.582541	1.157169	3.594653
2.60329	1.834235	2.427392	4.726064	3.422084	24.4953
0.08002867	0.08785195	0.005975337	0.07390893	0.0546863	0.380528

TCGA-DU-5870-01	TCGA-DU-6397-02	TCGA-HW-8321-01	TCGA-06-5859-01	TCGA-TQ-A7RI-01	TCGA-06-0221-02
1.593576	1.711434	1.976394	4.662719	1.374191	1.815189
1.502422	2.022162	2.12963	9.565419	1.948035	2.838917
0.7239269	0.05768378	0.8163667	9.439465	0.4508358	0.7161758
0.6816821	1.065086	1.471797	3.444336	0.5558038	1.30922
0.872634	2.153408	1.246887	2.820387	1.825399	1.579259
0.0653473	0.05901254	0	0	0	0.03052805
0.5199177	0.5548837	1.457448	1.78147	0.6548403	0.5078566
2.690031	1.124263	2.729763	6.840295	1.807223	2.763621
0.118167	0.08323527	0.23948	0.4524375	0.2251859	0.2384796
0.03014413	0.04083295	0.04324318	0.3760415	0.05065647	0.2112348
1.204265	0.7953242	1.099366	2.130272	1.315789	0.8348782
0.05667016	0.07676485	0.03448922	0	0	0.07942314
4.461956	0.7723045	6.703959	11.13536	3.120087	2.483999
2.739924	0.8816293	2.606517	4.682674	2.56096	2.443056
0.005950559	0.01209085	0.02987725	0.04663288	0.006999837	0.0166794
0.836673	1.351146	0.5490615	6.293436	0.3642612	1.614927
1.201405	1.872243	0.8929332	5.439557	0.8004258	1.609264
0.2801819	0.1541847	0.2238042	2.50938	0.2883887	0.4417588
3.143181	0.8264555	5.694023	18.58243	1.435396	1.412401
0.04344712	0.03531183	0.1150216	0.335619	0.01022165	0.2070297

TCGA-DH-A66D-01	TCGA-S9-A6WL-01	TCGA-12-5295-01	TCGA-DU-7012-01	TCGA-12-3650-01	TCGA-DB-A4XA-01
3.697542	0.6610409	3.632273	10.56926	3.701429	2.440845
3.468043	1.28338	7.481982	16.19246	5.283566	2.387293
2.560192	0.8484412	4.597367	5.231296	2.49224	0.2923472
1.278435	0.8669738	5.54808	5.168723	4.400379	1.089497
2.796741	1.789842	4.700019	5.111017	2.784149	2.141641
0.04555072	0.02799952	0	0.1831415	0.09806342	0.06646255
1.103708	0.3510323	1.928538	1.81526	1.655	0.8733062
8.364103	1.286276	2.569766	15.507	1.894732	2.828735
0.2817019	0.2126515	0.2085868	0.3488357	0.3830269	0.2776243
0.373716	0.0110708	0.6786995	2.321231	0.09693384	0.1708121
2.236017	0.5563926	4.444412	2.478244	2.112664	1.484165
0	0.02428161	0	0.105882	0	0.02881866
11.28107	2.1986	47.26299	8.272995	13.41957	1.096709
5.551977	1.316342	4.535873	9.840241	2.591989	2.08307
0.01244362	0	0.009378439	0.1111797	0	0.004539086
1.618869	1.048683	12.74536	8.759107	5.241153	1.826064
1.578549	0.3963266	7.40638	15.6334	2.847925	3.292665
1.698216	0.05627355	1.697336	9.430987	0.5617015	0.3740147
3.440245	0.6792186	31.98963	10.23395	7.197842	2.144144
0.07268417	0.02233908	0.260206	0.2272934	0.08801854	0.006628292

TCGA-DB-A75M-01	TCGA-S9-A6U0-01	TCGA-76-4932-01	TCGA-S9-A7R3-01	TCGA-27-1837-01	TCGA-RY-A83X-01
1.587524	6.640135	5.728651	1.359048	6.317989	0.7066694
1.64015	6.580989	4.876919	1.085664	12.13655	0.9578443
1.252703	1.403754	6.586175	0.5446382	11.9064	0.320259
0.8324905	2.415739	4.290717	0.5487029	2.656673	0.3616716
2.215047	6.242076	2.458539	2.007407	4.037935	1.517999
0.02464537	0.0326384	0	0.02932548	0	0
0.6357878	2.13251	0.821876	1.095897	1.215744	0.4792202
5.134811	3.845475	4.022998	4.532896	7.220263	0.938496
0.2968097	0.1841416	0.1791491	0.3181741	0.2771038	0.07109542
0.01948919	1.34857	0.08424064	0.04638029	0.07767975	0.008636326
1.474071	2.844735	1.881079	2.302116	2.087289	1.065765
0.02137284	0.02830451	0	0	0	0
3.632048	3.454285	8.599745	3.145378	8.160537	1.876696
2.912941	4.760142	2.394661	3.562099	5.541775	1.478704
0.006732662	0.04012294	0.003637682	0.01201677	0	0.005966945
0.808518	8.14653	11.66741	0.525121	3.115161	0.122413
0.9262553	15.15005	1.260897	0.8874448	1.885883	0.2771906
0.396259	2.099097	0.8885157	0.3831	1.658374	0.05267876
2.163364	1.583499	5.827718	3.622924	2.440235	1.207202
0.009831506	0.03906022	0.09561598	0.07019092	0.03918629	0.01307003

TCGA-S9-A6TW-01	TCGA-P5-A736-01	TCGA-TQ-A7RG-01	TCGA-19-2629-01	TCGA-DU-5872-01	TCGA-HT-7473-01
1.515719	5.134704	2.575641	7.041686	3.096254	5.646828
1.284858	3.962436	1.907291	16.03574	4.061917	4.882007
0.2310536	4.414671	0.9761873	7.642337	1.249893	8.927335
0.761049	1.741839	0.5311649	2.05445	1.21499	2.811155
2.401696	2.814385	1.623523	2.080769	2.042182	3.303626
0.183848	0.1122078	0	0.04856137	0.01967208	0.04521276
0.6458833	1.765211	0.6493709	1.194219	1.086122	2.152887
1.44076	9.339173	3.03402	9.336852	5.140571	12.2362
0.09688566	0.5661034	0.1379046	0.3292992	0.3073495	0.3752686
0.1038459	0.188556	0.06581136	0.1104046	0.4550242	1.376512
1.074814	3.145917	0.9050294	3.730966	1.130166	3.624916
0.04555307	0.02432707	0	0.04211315	0.05117976	0.03920919
0.9937998	6.687957	4.769864	25.60766	5.602356	20.05366
0.931093	6.805044	2.47242	6.798317	3.35273	8.810959
0.00358742	0.02682147	0.004133626	0.02321562	0.04836644	0.08645906
0.91906	3.535388	0.438489	1.868583	1.613409	4.032595
1.999816	3.190242	1.240777	6.399695	3.072591	3.409527
0.1530779	1.020458	0.3953456	2.264293	1.024009	2.094526
1.018075	6.790873	2.096412	5.386066	4.496593	7.999718
0.03143162	0.03916659	0.07243454	0.3099528	0.2628934	0.2254528

TCGA-S9-A7J1-01	TCGA-RY-A840-01	TCGA-12-3652-01	TCGA-TM-A84B-01	TCGA-S9-A7R8-01	TCGA-12-0616-01	TCGA-S9-A7IX-01
1.222219	0.4143083	1.991365	2.239952	2.066972	4.405504	4.078645
1.986117	1.030066	8.082505	2.426779	3.223563	16.88048	2.767492
1.197726	0.1336002	5.496038	0.4646596	1.740743	6.818993	3.129956
0.4959546	0.3983126	2.717248	1.816928	1.174237	4.442743	1.56651
0.8584441	0.7856146	4.646222	2.166565	2.271009	2.827423	5.026845
0	0.02733554	0.03268977	0	0.0954022	0.2683104	0
0.3676348	0.1054486	0.4334786	0.4202328	1.096393	1.282999	1.062765
3.384761	0.376732	1.492909	1.107948	2.853053	3.782381	6.0402
0.2127099	0.05338508	0.3156615	0.01934089	0.3760827	0.3105174	1.38966
0.01076624	0.005404133	0.1357155	0.231028	0.02514754	0.4862362	0.1109865
0.7125191	0.3710958	1.125536	0.4910142	2.809265	2.9914	2.008625
1.227908	0.2607637	0	0	0	0.1551219	0.01803163
2.22333	0.6066088	2.678495	0.2479438	10.0607	18.51124	9.003455
2.196132	0.6511307	0.6671046	0.7311467	4.930338	2.852963	5.695692
0	0	0.01339537	0.01623256	0.004343687	0.07940557	0.00852022
0.2940395	0.2540854	2.341457	1.935813	2.043045	3.740939	5.66446
0.5847804	0.4536398	2.521004	3.006625	0.1707396	8.534293	1.644098
0.3611879	0.005493908	0.2102401	0.7603285	0.4921319	1.455978	1.512761
0.9192983	0.4580255	3.270085	1.089743	7.046249	9.483513	4.31072
0.02172452	0.02726166	0.1238854	0.003950653	0.05708661	0.2854242	0.1078291

TCGA-DB-A64P-01	TCGA-HT-7611-01	TCGA-TM-A84I-01	TCGA-TQ-A8XE-02	TCGA-HT-A617-01	TCGA-28-2499-01
1.692567	2.379118	1.79615	2.419428	5.217245	3.657906
1.93994	2.39104	3.172315	12.96169	4.329307	10.42587
1.399627	2.511458	3.209319	1.808831	1.223352	6.106514
0.8210401	0.9752214	1.413483	1.230015	2.906059	4.868679
1.985982	1.593867	1.596852	1.920102	4.02524	6.896343
0.07954819	0.0790557	0	0	0.2619485	0.05553048
0.5881524	0.9339472	0.4566826	0.911422	1.361345	1.111226
2.608225	9.335752	2.750425	3.814752	4.192135	6.197495
0.2128926	0.2616091	0.1452298	0.1089918	0.183156	0.2289469
0.07338975	0.08595958	0.09486634	0.02613117	0.4718301	0.065869
0.8608	1.594287	1.063367	1.705979	3.825244	1.835482
0.04599026	0.03427915	0	0.02292542	0.0757219	0
3.123171	8.529924	6.071602	12.79326	4.628834	6.824983
1.900373	5.197801	2.923517	4.126166	3.237373	2.380531
0.0434622	0	0.003449702	0.00361087	0.1033637	0.003792476
0.8735124	0.4943873	2.84466	1.87543	4.579223	3.522025
1.061274	1.340888	1.257375	1.48386	10.68306	3.130531
0.1385594	0.7705993	0.7207791	0.8979059	1.222569	0.6305704
2.612717	3.420318	2.096937	3.074189	3.173563	8.637916
0.03702216	0.06701573	0.04533747	0.01581854	0.02902673	0.09968472

TCGA-DU-A5TT-01	TCGA-19-2624-01	TCGA-KT-A7W1-01	TCGA-S9-A6U1-01	TCGA-DU-8167-01	TCGA-FG-8186-01
1.719091	1.165007	1.943832	2.034643	4.185866	2.481838
5.099324	3.410414	7.498497	1.005161	4.183758	11.87722
1.40235	2.382328	2.904469	0.6158362	2.611702	3.704041
1.728859	3.099325	2.955685	0.7886628	1.659049	1.151558
3.331836	2.835741	3.189663	2.229859	3.34719	1.514188
0	0	0	0.05040177	0	0.02229038
1.063296	0.6052327	0.5465801	1.172644	1.604256	0.7631305
3.202011	1.396248	2.617043	7.988192	9.40708	7.204158
0.1643039	0.1824572	0.1265666	0.2788918	0.4264735	0.2660295
0.03151395	0.01445479	0.08702658	0.03487484	0.03047407	0.1189815
2.226754	0.8535352	0.9526985	1.809748	3.068166	1.477939
0.09215949	0	0	0.02185459	0	0.1546444
11.54194	1.837488	2.504782	4.488245	13.41961	8.675408
2.731203	1.0134	1.419544	4.596907	5.699079	3.305274
0	0.009986994	0.003006387	0.003442209	0.003509153	0.2161709
13.01923	2.045519	1.973651	0.3272521	0.2493344	0.6489931
1.348625	1.094421	3.631155	0.5289186	2.16936	1.969249
0.5926932	0.176339	0.8670283	0.2988282	1.043004	0.8108671
5.627608	2.535553	4.287934	2.590269	8.827846	3.088263
0.1271801	0.01458371	0.05268165	0.08545144	0.06149173	0.2134093

TCGA-06-5416-01	TCGA-02-2485-01	TCGA-S9-A6TS-01	TCGA-DU-A7TJ-01	TCGA-CS-4942-01	TCGA-P5-A5F6-01
1.930296	3.779341	2.202314	2.902461	4.578433	1.912222
4.273938	6.519337	2.026771	6.199049	12.4496	1.519116
1.471255	1.208439	1.40075	2.875417	11.37963	0.6185039
1.780181	5.384225	0.8757869	2.074623	1.33638	1.009123
1.183492	2.033156	3.267526	2.823764	1.923496	1.519773
0	0.143753	0.07816457	0	0.1444899	0
0.3732553	1.420999	0.8857289	0.6356763	1.094851	0.2007301
1.045894	3.014486	2.767043	4.009954	9.789292	1.133926
0.1166461	0.2245945	0.4947052	0.2647862	0.2754637	0.06503868
0.1105229	0.1818843	0.08756609	0.1163107	0.2285209	0.06583818
1.31991	1.176575	2.060744	1.071783	2.428566	4.416199
0.07458771	0.02493295	0	0	0.01790055	0.08664181
4.110893	6.355566	6.352664	8.214098	17.57064	1.373833
0.8536302	1.623985	3.883442	3.918576	10.56818	0.8280605
0.01174794	0.0117812	0.02135312	0.005952631	0.02255542	0
1.587147	2.782423	1.374735	4.053767	2.291071	1.279172
2.686736	5.599178	0.763035	1.4039	3.032568	1.381666
0.3284337	1.265449	0.2042241	1.983853	2.128191	0.1204774
2.054145	5.212811	2.280572	1.858229	4.465219	3.348056
0.06004311	0.0516112	0.05196887	0.03042358	0.230559	0.06642539

TCGA-TM-A84J-01	TCGA-VW-A8FI-01	TCGA-DB-5274-01	TCGA-DU-A6S7-01	TCGA-28-2514-01	TCGA-TQ-A7RJ-01
2.051396	5.82433	2.601922	2.735614	4.298709	2.207727
2.576478	6.247651	3.882581	2.598424	6.323737	1.605697
3.425785	2.871477	0.9185176	1.137093	0.9906341	1.731912
1.374682	1.832551	1.030808	0.7661003	4.786999	0.887474
1.330976	3.153343	2.664795	3.789934	3.335359	3.140139
0	0.170792	0.1468244	0	0	0.02214759
0.4086353	0.8647285	0.7646187	1.05812	0.9387851	0.7529024
1.855339	2.676887	1.817181	8.901979	3.812507	4.698193
0.07168667	0.2038355	0.1210686	0.6683586	0.3530204	0.4301296
0.005679226	0.0472709	0.1219118	0.01045351	0.05272525	0.01751398
0.8025802	2.372365	2.703854	2.860921	1.143895	2.09595
0	0	0.4074505	0.02292771	0	0
3.015807	3.819235	5.614147	8.349169	14.24959	4.845507
1.2405	2.483775	1.499155	6.947604	2.247605	4.726392
0	0.004665722	0.07620845	0.01083369	0.01457141	0.01210063
2.607366	2.913567	1.497201	0.2963403	2.486267	0.4389728
0.5748827	4.601623	4.342855	0.1935663	1.21062	0.5729371
0.2482635	0.82382	0.548864	0.318815	0.4931304	0.249269
1.048892	3.442624	2.269205	3.484429	4.855554	2.713999
0.06875855	0.05450572	0.07614232	0.06328048	0.2021429	0.03092283

TCGA-06-0747-01	TCGA-27-2521-01	TCGA-14-1829-01	TCGA-HT-A5RA-01	TCGA-12-0821-01	TCGA-DB-A75O-01	TCGA-S9-A7J0-01
1.803757	2.45065	3.781262	6.187523	3.065614	3.67584	1.333631
6.668333	4.233309	2.984068	11.10174	4.702942	5.178627	2.233355
2.88878	1.508478	17.02784	4.421032	6.06523	4.269508	1.252306
3.651298	1.678573	4.379279	3.116839	5.035782	1.161258	2.466083
4.474434	1.603525	1.939343	5.350618	2.990309	2.319882	1.067937
0.05051836	0.0532147	0.06687171	0.02614377	0.05564972	0	0.06863319
0.4871944	0.654327	2.490944	0.6618361	0.8184382	0.7564379	0.4081668
3.453858	1.970093	5.529661	4.295427	3.233226	5.294856	1.891773
0.2713152	0.1760967	0.7110305	0.05389413	0.208306	0.1935654	0.2085028
0.02996187	0.02630086	1.500502	0.6202228	0.1870296	0	0.01356852
1.436238	2.737869	1.5854	1.265355	1.324822	2.929103	0.8417126
0.04381028	0	0.1159843	0.09068907	0	0	0
8.688243	5.949912	26.12157	5.481785	7.615431	6.528378	1.972159
1.578069	1.43065	2.584936	2.647567	1.637586	4.687505	2.16863
0.003450171	0	0.01826812	0	0	0	0.003124887
1.412166	2.098555	2.609706	2.673085	4.290275	2.83389	0.866235
0.7643909	0.9090844	2.953899	6.09959	1.996434	0.2852583	0.3573283
0.812256	0.2192498	0.9273533	1.050877	0.5760019	0.6862095	0.2712805
19.82254	2.827989	7.324933	1.183406	3.200943	2.753913	1.498999
0.09572547	0.1432914	0.9136664	0.2763749	0.1498482	0.03681171	0.03194226

TCGA-DH-A669-02	TCGA-27-1834-01	TCGA-HT-7478-01	TCGA-28-5204-01	TCGA-DU-A76O-01	TCGA-DB-A64V-01
1.451794	8.178823	6.248332	4.373537	4.525346	5.463143
11.83907	20.26717	8.4734	10.58629	3.949106	9.779879
1.978762	15.5462	15.07334	6.802633	6.075818	0.7377757
1.371654	2.552524	2.457702	3.568856	0.7708026	0.9706515
2.01817	3.048366	3.321001	4.004782	4.490004	2.532908
0.07990829	0.1303632	0.09961602	0.02936427	0.02690812	0.07547705
1.040348	1.359359	2.118316	1.146905	0.6811859	1.746945
1.864254	14.73417	14.38029	6.49887	4.966366	4.867897
0.1647272	0.2510574	0.4215155	0.1561116	0.7940941	0.2620503
0.02106342	0.5927631	0.2875282	0.2147926	0.01063927	0.2337706
1.346831	2.750816	4.233428	1.663876	2.647053	2.880885
0.04619845	0.05652646	0.0518331	0	0.04667025	0
3.432853	7.399191	52.61228	13.94319	7.050653	3.421432
1.473009	7.758566	10.29621	3.210948	5.618616	3.483569
0.01091474	0.04896763	0.005442652	0.05615245	0	0.00687298
2.133591	5.301332	2.312117	6.129165	0.6142467	5.124163
2.067151	5.933465	8.440797	3.640471	0.9456282	6.041758
0.4818	4.847082	2.995122	1.699673	1.032929	0.8697127
1.172385	5.216295	11.64906	2.768521	2.82631	3.30324
0	0.1300109	0.6994014	0.1757095	0.1127087	0.04014563

TCGA-CS-4941-01	TCGA-QH-A6X8-01	TCGA-S9-A6WP-01	TCGA-DH-A7UV-01	TCGA-S9-A7R4-01	TCGA-DU-7013-01
7.060865	2.801655	3.765983	1.991054	2.263614	2.902179
17.86786	2.139311	1.714266	1.692862	2.74446	5.511454
6.591234	0.5857699	0.2840603	1.506323	1.51827	0.9686744
2.42949	1.198666	0.9512719	0.658861	0.9632921	1.57318
4.310147	1.240029	2.367333	3.116818	2.892359	2.189135
0.2081193	0	0.04470826	0	0.141204	0.07864984
1.329692	0.9940291	1.573743	1.036976	1.116642	1.1567
10.89935	5.259789	5.279635	5.612501	6.074699	3.710879
0.1919336	0.1482423	0.2449621	0.6288604	0.4167117	0.1553063
0.4237873	0	0.08396722	0.01818831	0.0446648	0.1990243
2.68612	1.098861	2.097903	2.009214	1.933595	1.48552
0.03609683	0.0207876	0.0969292	0	0	0
11.92499	4.194103	1.984261	5.169487	8.364003	5.773032
5.721288	2.192082	3.602762	4.017686	4.645916	4.387779
0.05969707	0.006548307	0.009160105	0.006283265	0.007714867	0.01074284
3.240254	1.572758	2.102274	0.3364037	0.9882346	3.87889
8.167195	0.4445962	1.789395	0.482733	0.2481156	3.247677
2.421835	0.5010311	0.6065202	0.3189604	0.9365149	1.343601
3.903559	1.726463	1.956649	4.198982	5.169818	2.549118
0.1037784	0.004781149	0.03566994	0.0137629	0.08449343	0.04078738

TCGA-FG-6688-01	TCGA-DU-8161-01	TCGA-HT-7858-01	TCGA-QH-A6CV-01	TCGA-TM-A7C3-01	TCGA-HT-8114-01
4.170912	5.89451	4.928642	5.648834	1.16158	3.38595
7.456536	16.11934	2.605792	9.119308	1.732999	4.105949
1.502466	6.510117	1.872505	1.23112	2.337315	6.307025
3.964659	2.177702	1.879678	1.919487	1.394564	1.150571
3.133545	4.099283	2.0885	4.257682	3.854857	2.899611
0.1082448	0.05612426	0.09459944	0.0514073	0.05313679	0.05365745
1.586732	0.8073753	1.328549	1.388148	0.531663	0.5530434
4.428611	11.75404	5.886035	5.313625	2.354907	6.883823
0.1691177	0.2557524	0.3284417	0.2537791	0.1037738	0.3332923
0.05991883	0.5732703	0.2244234	0.05081514	0.0735346	0.8221103
2.806931	2.385139	2.573137	3.398391	0.9774988	2.657717
0	0.06489574	0.04101903	0.02229059	0	0.01163314
11.10501	15.19574	5.214548	6.413289	1.103582	10.49135
4.470705	7.08831	4.373282	4.591944	0.9881457	5.596433
0.008871143	0.4369652	0.01615176	0	0.01088699	0.2052152
5.356222	3.613388	2.49568	6.893453	0.6464402	1.84097
2.451487	8.59258	5.067545	2.458982	1.685828	2.376738
0.9659255	3.365163	0.8745811	1.017688	0.6140685	1.685015
5.863441	2.704235	5.695633	4.152546	1.136184	4.216892
0.06045325	0.2015013	0.1084953	0.03588785	0.04239454	0.2327791

TCGA-S9-A6TU-01	TCGA-06-0211-01	TCGA-HT-A618-01	TCGA-QH-A6CX-01	TCGA-TM-A84L-01	TCGA-RY-A83Y-01
2.551455	4.587802	5.430226	2.596699	2.419114	2.592716
1.579902	5.661004	7.698601	4.564186	2.068085	4.258144
1.207383	6.507536	5.155636	0.7210776	1.462032	0.03256571
0.7425195	2.180119	1.920423	1.8792	0.9712273	0.9378076
3.634098	2.724394	3.45251	3.543368	3.085404	0.9516841
0	0.02474884	0	0	0	0.1332635
1.035835	1.044206	1.080215	1.094491	1.131332	1.164695
6.269402	4.079617	9.915825	10.17431	9.145715	3.160042
0.4632277	0.2738891	0.7814719	0.3755595	0.5791196	0.1554316
0.02123421	0.2006029	0.2578791	0.01682747	0.0985654	0.01317284
2.456631	1.494867	3.085278	1.691419	2.290746	1.245415
0	0.1717006	0	0.0492103	0.07630012	0.02889202
7.242232	16.30018	23.42035	5.553593	7.909917	1.450203
4.892489	3.799102	6.936315	2.966158	4.888035	1.492026
0.003667745	0.04056557	0.0116199	0.01162631	0.004005886	0
0.8955927	4.198264	3.903304	3.09489	1.186621	0.5874675
0.7601692	1.207979	2.394477	1.440248	1.302636	1.707438
0.6853858	1.153975	2.735614	0.5645314	0.9607679	0.3749668
5.5334	3.39793	4.489012	2.284769	4.180605	2.099611
0.04820309	0.1480917	0.3167398	0.01131837	0.05849676	0.01993549

TCGA-HT-8015-01	TCGA-DH-A7UT-01	TCGA-VM-A8C8-01	TCGA-DU-8158-01	TCGA-12-0618-01	TCGA-TM-A84C-01
2.191082	2.029653	1.653803	6.060514	2.558195	3.863036
2.108883	1.986262	1.61267	5.191551	4.62238	10.56949
2.449369	2.13108	0.9138728	8.629786	1.479185	3.456047
3.08096	1.395497	0.6939615	2.61483	2.613624	3.117898
2.377304	1.99506	2.100138	3.07552	1.232739	1.541361
0	0	0	0.04824358	0.02328089	0
0.3920485	0.7453017	0.7694963	1.039073	0.4939413	0.2825226
4.862452	4.097661	4.556325	5.379892	2.28371	5.465136
1.084596	0.3389272	0.3882586	0.3541882	0.2273327	0.1841328
0.0843209	0.01286601	0.03186741	0.8615608	0.06443563	0.04793049
1.295457	1.61974	2.175614	5.897575	1.452005	1.093136
0	0.02821904	0	0.04183756	0.1009477	0
3.754123	6.943255	6.943215	4.588868	6.41815	18.83053
2.655017	3.188776	3.201933	4.872164	1.499878	3.890826
0.007282295	0	0	0.02855506	0.006359911	0.002759648
5.039438	0.8317639	0.1277966	8.007934	3.825139	2.734075
1.639427	0.07941271	0.4248608	9.623062	1.238599	0.7691861
0.9536534	0.3858523	0.2008601	2.7569	0.3322092	1.372469
1.919979	2.042567	2.475163	10.44827	3.819361	2.59201
0.01595118	0.006490379	0.03215163	0.141132	0.1625258	0.1088054

TCGA-TQ-A7RK-02	TCGA-WY-A85B-01	TCGA-HT-A615-01	TCGA-FG-7634-01	TCGA-41-2572-01	TCGA-DU-6542-01
2.539124	2.937084	3.945601	2.901616	4.441228	3.18063
2.982484	3.945645	3.841449	4.518417	9.27038	5.521481
0.8391903	4.287297	0.2160349	5.065717	7.350009	5.411017
0.9867289	1.043414	0.8508463	3.001705	4.887019	0.9385355
1.957177	3.302445	2.62252	1.723281	4.373405	2.029896
0	0.02775985	0.08287923	0	0.03312475	0.02648642
1.461088	1.412188	1.465346	1.017352	1.349685	0.8365416
9.546067	9.827046	7.63721	7.450591	5.988435	7.479528
0.3314952	0.2891399	0.3147266	0.2666932	0.2587646	0.5028993
0.004991953	0.1426884	0.2348504	0.2956189	0.2292023	0.1204341
2.007071	2.747226	1.723031	1.817594	1.935612	2.527402
0	0.1203688	0.02395804	0.05329163	0.02872628	0
7.53588	12.43896	2.750926	24.66866	15.30471	14.98802
4.904955	7.082203	4.581401	6.254548	2.332481	6.855093
0.01034701	0.02654217	0.02641459	0.04476642	0.02262268	0.003617801
0.5522493	0.7285533	2.10718	2.707575	3.015573	1.303373
0.5176378	1.449791	3.263205	2.249563	3.637807	1.357429
0.8576548	0.8591944	1.188206	1.181535	0.4593623	0.7878411
3.018472	3.977945	2.521764	4.938274	5.865067	2.993979
0.05540123	0.1162762	0.01653105	0.3145983	0.2907099	0.0792445

TCGA-HW-A5KM-01	TCGA-14-1402-02	TCGA-P5-A731-01	TCGA-HT-A4DS-01	TCGA-06-2563-01	TCGA-06-0219-01
13.98155	4.348326	2.197387	6.110807	4.210245	7.591871
86.84446	7.380842	2.335893	24.6278	6.320809	5.570607
27.55585	10.4724	2.744922	2.868253	2.996825	12.18174
2.29652	5.467273	2.345846	2.182903	3.01174	6.029265
5.839609	2.426566	1.546688	9.659052	3.732939	2.515326
0.03476031	0.2925225	0	0	0.0294794	0.6792277
1.986209	1.384082	0.5733483	1.240616	1.705779	3.218245
43.40645	5.157918	3.069578	2.608672	6.24553	7.557397
0.2432557	0.5633487	0.244291	0.359893	0.3070503	0.5190657
0.8040218	0.3108394	0.04001161	0.1261097	0.2272906	0.1634722
5.248936	0.964014	1.30907	4.359192	1.4848	2.138182
0.01507233	0.09512996	0	0	0.02556498	0.07683085
37.11987	18.23633	4.858339	9.183055	6.952796	22.66814
24.05887	1.882037	1.927655	3.859383	2.966886	2.581343
0.009495875	0	0.00691113	0	0.01207984	0.0080675
1.7129	2.469103	2.393023	7.179083	2.312989	7.839346
1.73905	8.049156	0.8643712	6.725252	3.654743	4.324276
9.763131	0.7642866	0.4118474	1.324782	1.730036	1.151444
6.185085	5.651255	2.682568	2.903413	2.705651	4.549414
0.5269287	0.4375978	0.1412896	0.0084823	0.1587585	0.1826013

TCGA-FG-A4MX-01	TCGA-HT-7690-01	TCGA-VM-A8CF-01	TCGA-E1-A7YY-01	TCGA-14-0871-01	TCGA-HT-8105-01
1.286566	3.837565	6.575743	1.398572	10.75591	1.949281
4.795658	8.015379	15.3913	1.768806	3.950909	2.356087
1.555293	16.59328	3.564909	1.79399	1.871633	0.7193024
1.079341	1.77578	2.050393	0.8164903	4.928655	3.798768
1.796946	2.371558	3.959162	3.262224	5.184568	1.873603
0	0.03906908	0.1696292	0.04422445	0.3481357	0.04328657
0.4207393	0.8242031	2.617421	0.30921	0.3777062	1.106247
1.733747	7.073635	4.652659	2.115292	2.257847	1.427078
0.3007147	0.4026952	0.4647096	0.7605214	0.1133156	0.3264057
0	0.2857809	0.03353505	0.01311451	0.1032377	0.2182186
1.646031	2.709577	4.355323	0.5351147	8.664597	0.5195081
0.08902212	0.06776258	0.03677624	0	0.3019085	0.01876938
2.131966	22.28053	7.902568	3.963322	2.079956	2.703175
1.562201	6.266738	4.864616	1.504671	0.9092729	1.358196
0.04206431	0.08805185	0.005792445	0	0	0.01773764
0.2245092	1.898523	4.283789	4.603365	2.093844	1.834247
0.3507307	3.890164	3.642988	0.2266503	1.954119	0.9507587
0.08252486	1.966956	0.5966124	0.2088738	0.2448896	0.1130968
1.052549	1.866181	5.366341	1.874646	2.263649	2.522297
0	0.2883298	0.02537561	0.03528394	0.1735974	0.2806023

TCGA-DB-A64L-01	TCGA-DU-5852-01	TCGA-28-1747-01	TCGA-HT-A61B-01	TCGA-DU-A7TG-01	TCGA-WY-A858-01
2.412642	3.669417	11.2591	11.3027	2.83108	5.033559
3.238385	5.408094	20.6946	33.05659	5.354699	6.500653
0.7035774	2.920775	7.048347	14.34558	1.995439	4.800661
1.971494	3.099582	3.070196	2.218779	0.9050223	1.23311
2.241024	4.113923	2.442144	4.885204	2.655317	4.120649
0.02999102	0	0.2575253	0.02900403	0	0.09629894
0.7953842	1.663307	1.897156	1.67128	0.2540273	1.509134
1.629003	6.180665	14.5523	15.66772	2.304474	10.40917
0.2961095	0.2812882	0.3973809	0.6199323	0.3429481	0.4771342
0.1482278	0.1129332	0.2262745	0.2293595	0.05858391	0.02538391
0.8969002	1.979815	3.073826	4.205669	0.8874979	4.054568
0.1300433	0.03810716	0.04962884	0.1006109	0.08566147	0
4.675824	9.845024	15.89886	45.76406	8.084155	18.96807
1.399524	5.862413	8.119191	8.54906	1.79791	6.559561
0	0.009003108	0	0.1980841	0	0
1.980074	3.7781	5.53448	3.742599	0.8101268	1.877963
3.820642	2.080444	6.550203	2.052725	0.6910516	1.770448
0.2411043	1.713307	3.243472	2.68728	0.5095442	1.458016
3.81765	4.428657	3.649511	3.104482	2.602026	5.434706
0.4187395	0.03067627	0.09131707	0.4454548	0.2758299	0.1600644

TCGA-08-0386-01	TCGA-S9-A6WG-01	TCGA-DU-5870-02	TCGA-E1-A7YJ-01	TCGA-DH-A66G-01	TCGA-76-4931-01
2.563116	4.780072	1.363195	4.342714	2.778822	3.416009
5.478111	9.112914	1.725201	20.30139	6.089001	5.129453
3.498921	1.633521	0.4223826	4.385723	5.634851	4.296027
3.733574	7.780783	0.5597744	3.921387	8.017082	5.017755
4.325087	6.462974	0.9297471	5.772788	2.862611	4.753773
0.0281852	0.02832456	0.02541837	0.02116391	0	0.0542591
0.4960631	1.372626	0.3799552	1.46954	0.8218789	0.6867915
3.823485	4.416454	0.8448652	4.140656	5.235058	5.21984
0.256874	0.5654585	0.1158288	0.3811744	0.7186664	0.25314
0.07243739	0.2351857	0.07035161	0.1506249	0.1205399	0.1072682
1.036984	2.429735	0.4100821	2.839812	1.864855	0.8807158
0.02444263	0.09825394	0.04408638	0	0.02403458	0.02352715
7.377263	16.54392	0.3832745	13.27672	23.36295	8.737263
1.874731	4.042216	0.8745588	3.705746	3.198502	2.146522
0.02309903	0.003868874	0.006943829	0.002890795	0.01892784	0.003705646
4.615523	4.332478	0.6983719	1.8023	5.133245	4.468612
0.2751412	6.940192	1.091779	4.142332	1.515069	5.256996
0.5891259	0.8368242	0.1123891	1.016594	0.8745065	1.134122
5.434538	7.352715	1.004366	12.21042	18.60978	3.751931
0.2192504	0.3841729	0.03548954	0.1561897	0.5362114	0.2326835

TCGA-FG-A70Z-01	TCGA-TQ-A7RH-01	TCGA-VW-A7QS-01	TCGA-76-4926-01	TCGA-32-2634-01	TCGA-16-0846-01
3.647655	3.166981	0.9938605	3.094394	2.821565	8.030081
9.443506	2.59872	2.337704	4.06641	8.06404	16.10732
7.714278	0.5577728	1.061639	6.442009	3.451306	8.215938
5.251221	1.090511	0.7946325	3.861054	8.43274	5.319581
5.172066	2.803646	1.117456	3.539753	4.072818	2.667761
0.04164632	0	0.025258	0.0479302	0.04676567	0.1556517
1.079389	1.186588	0.1400619	0.7395751	0.9865707	1.786298
3.781382	6.624147	7.023427	3.782046	2.792891	10.82673
0.5761115	0.3379307	0.1096172	0.2314137	0.2536979	0.3445116
0.1728997	0.07520644	0.01498024	0.07580496	0.06009507	0.1969393
2.62202	2.273433	0.54167	1.56069	1.651585	3.681016
0.03611632	0.04123761	0.06571236	0.0207829	0.06083383	0.02699671
20.42087	5.661722	0.5820636	7.53404	7.623741	10.24716
4.638094	4.919871	0.6790484	2.371853	2.152995	4.327718
0.06541779	0	0.01380004	0.02291388	0.009581638	0.004252119
3.842585	0.4289964	0.9252878	4.166865	4.954181	2.919114
1.900609	0.998021	0.1849247	1.076147	1.164131	11.33515
1.397806	0.6642096	0.1015273	1.17523	0.8036129	2.577715
11.87843	3.603267	1.004346	6.724489	6.47924	6.041334
0.1702884	0.0948465	0.03526563	0.1720824	0.1865571	0.1179756

TCGA-HT-A74K-01	TCGA-76-4925-01	TCGA-DH-5142-01	TCGA-28-5216-01	TCGA-15-0742-01	TCGA-06-2559-01	TCGA-41-2571-01
2.103649	2.643048	6.465065	5.908544	3.338335	3.630421	4.624979
6.305528	6.942435	13.37213	6.868985	7.693282	6.73907	6.470327
1.623248	3.906486	26.12961	16.72751	7.842893	11.11643	3.972983
1.877216	3.823415	1.495146	6.23043	4.812221	6.171449	5.292544
2.375188	2.37327	3.021064	2.279367	4.261969	3.02955	1.665108
0.03193539	0.02337119	0	0.06942326	0.03062426	0.03185573	0.2601281
0.5312688	0.5409349	0.9592683	1.046112	0.6718929	1.35174	1.034819
1.777764	4.073569	15.82512	7.935605	2.275794	5.500764	4.999703
0.1385964	0.2155358	0.2920981	0.2673949	0.1860687	0.3248891	0.6067998
0.01894052	0.05082435	0.5652105	0.1578342	0.0181629	0.3904609	0.03214146
1.514253	1.278306	3.18749	3.045925	0.9941659	1.34752	2.405439
0.1107794	0.06080353	0	0.09030734	0.1062313	0.0828773	0
5.151594	7.127905	27.71623	15.45524	9.453263	18.09889	12.08167
1.394614	1.844272	9.737223	2.918077	1.409878	2.908738	2.355293
0	0.01915373	0.1952555	0.02370644	0.004182992	0.0174048	0.01332416
3.50098	2.606837	2.527175	9.745822	3.826087	11.68292	12.04508
1.558751	1.562809	3.872393	1.897569	1.569493	1.539314	1.618833
0.1668781	0.6716925	3.725322	1.765018	0.5231644	1.075599	0.888771
2.140396	3.378281	2.12709	10.2434	6.770255	9.424524	8.180548
0.01273963	0.1305249	0.3611593	0.2284776	0.2687651	0.4257131	0.1102556

TCGA-DB-A64S-01	TCGA-32-1982-01	TCGA-19-1390-01	TCGA-DU-8165-01	TCGA-27-1831-01	TCGA-VM-A8CD-01
4.403932	7.988522	3.168884	5.531064	3.913313	6.072722
8.414385	9.379976	5.382473	12.80542	7.34945	27.71928
7.458898	13.35225	1.779232	5.990709	31.76353	10.72408
2.068936	3.983729	3.8041	3.696124	4.478487	3.701155
3.791425	6.324242	1.563803	5.871234	2.548699	3.264684
0.121605	0	0.02459752	0.05622666	0.03891641	0.1025338
1.34133	2.337217	0.628623	1.050599	2.111098	0.7910611
6.868035	6.76342	3.762205	7.931356	5.531631	7.4811
0.8608982	0.9466039	0.3389338	0.3080728	0.7937987	0.1802195
0.1021736	0.3658736	0.02917702	1.645138	0.453924	0.06081163
2.721524	3.606501	3.068244	2.212491	1.722759	1.226535
0.02636443	0	0.04266268	0.0243803	0.0337489	0
35.97219	47.53769	7.159017	8.886132	17.41598	12.64861
6.703735	5.180483	2.230575	6.806228	2.775538	4.23626
0	0	0.00671959	0.1420808	0.01063125	0.01120414
1.242527	4.710133	7.329779	6.721174	2.06399	3.052586
1.469032	11.82907	0.5642789	9.042786	5.698475	1.651526
1.020379	1.275262	0.6723323	4.22072	1.384394	2.052485
12.41552	12.27597	5.173382	9.364911	5.450144	2.179581
0.3759567	0.4570265	0.1962483	0.1962614	0.3104899	0.1595204

TCGA-DU-7006-01	TCGA-P5-A5EX-01	TCGA-DU-A7T8-01	TCGA-DU-6403-01	TCGA-FG-A710-01	TCGA-S9-A89Z-01
5.785582	9.361983	3.948502	5.891879	9.330104	3.938282
4.641197	11.79073	13.87469	5.324325	10.36272	6.629862
8.592119	8.004683	3.758125	3.635167	4.378816	12.59041
3.675312	3.270807	1.580298	2.312025	3.861604	1.291163
4.492637	3.739406	3.017447	2.261193	2.99169	3.524734
0	0.02766578	0.0452317	0.06844609	0.2025987	0.0315697
1.228892	1.327361	0.4525682	1.325676	1.845296	1.088427
9.64689	6.810767	6.802096	5.46872	10.23187	5.587871
0.2545976	0.3331852	0.4490387	0.151	0.4762649	0.5822896
1.308072	0.6891471	0.1564874	0.0811892	0.1290597	0.09361814
3.954579	2.825008	1.690853	2.863897	1.749446	3.192588
0	0.04798437	0.05883842	0	0	0.05475543
8.607753	7.918216	13.65352	6.043151	11.36787	13.69704
7.909253	4.214113	4.024558	5.831871	5.707028	7.132892
0.02627218	0.05290448	0.01235647	0	0.07379498	0.03018491
7.821764	6.79948	1.8703	6.203049	2.430885	2.852429
5.614104	11.53202	1.501262	1.893132	2.010712	4.283707
3.742	3.202721	1.263605	1.274753	2.36619	0.8248357
9.264418	5.334387	3.523612	1.831553	6.080022	5.779212
0.08056541	0.1214005	0.496204	0.0227537	0.08531049	0.151125

TCGA-19-1787-01	TCGA-FG-A6J3-01	TCGA-HT-A74O-01	TCGA-DU-5847-01	TCGA-DU-7007-01	TCGA-DH-A66B-01
7.126681	6.203382	4.977397	11.81726	5.068368	3.754563
18.52761	48.10726	12.71252	26.19343	6.962226	9.79238
5.442155	8.577057	3.063796	15.32707	14.00498	8.920029
10.22991	2.024818	1.476455	8.293714	3.30175	1.54168
3.972843	6.373603	2.908566	9.433338	2.161411	3.487639
0.102627	0.06223143	0.02702045	0.1002566	0.04081933	0.02645074
1.515517	1.380355	1.980428	1.131232	0.6150904	0.8035286
9.803605	3.178383	6.70301	12.11031	7.931028	7.01914
0.2505324	0.442253	0.2843701	0.3306787	0.378662	0.3472505
0.2485399	0.05536313	0.2884594	1.066335	0.9966238	0.6065884
2.306892	4.866943	3.168459	2.536665	2.14832	2.550017
0.02224992	0	0.04686507	0.06955522	0.123897	0
14.68643	22.47651	10.07817	29.49877	14.19715	18.48215
5.843377	5.13404	5.275092	6.343142	4.860147	6.683738
0.06308057	0.00850024	0.01845372	0.06025415	0.122662	0.1083878
5.653401	7.205037	4.376772	4.963687	3.453819	1.489629
5.798121	14.1395	7.029498	6.586619	6.515057	2.633737
2.495748	1.069374	1.341353	4.029922	1.825365	2.200857
7.430148	5.890645	4.304464	13.3236	8.66684	4.326161
0.1739944	0.2048087	0.08623174	0.7358942	0.2605377	0.1793789

TCGA-06-2558-01	TCGA-26-5139-01	TCGA-06-0125-01	TCGA-14-2554-01	TCGA-02-0047-01	TCGA-DU-6396-01	TCGA-32-5222-01
5.527043	5.42156	3.353641	8.764139	7.604691	5.935511	5.850809
5.415326	11.35449	4.736965	5.26275	11.41771	20.80777	8.23337
12.99008	14.35265	5.319813	34.87552	16.25925	8.295608	7.620969
5.881543	6.605577	4.703301	5.998946	4.697788	3.450734	7.276349
3.114949	2.661705	3.584026	3.438318	4.597079	4.444997	2.411767
0.1233346	0.02990482	0.02485094	0.0524304	0	0.05948621	0.2531338
2.371422	1.016608	0.5632014	1.181919	1.301752	1.826213	3.850991
6.899062	8.0812	3.948706	6.729944	5.781821	16.2036	6.279534
0.4683525	0.3017476	0.2426639	0.4977497	0.6003617	1.017597	0.4256979
0.4327945	0.1773621	0.1326493	0.5908217	0.2651964	0.4351273	0.1801569
2.699569	2.094597	1.100106	2.718148	3.635551	6.222502	2.699347
0.1069576	0	0.04310222	0	0.05816559	0	0.02195214
20.31655	20.77654	7.763036	16.08008	21.89669	37.59946	24.2338
4.252112	4.076115	2.158359	3.79779	6.68254	13.497	4.686185
0.01263478	0.01225417	0.006788819	0.01432302	0.01832275	0.1760475	0
3.123103	7.877092	3.655091	4.991687	10.43337	2.366205	9.619176
2.302614	4.554077	0.5458332	3.672313	1.685975	3.910038	2.261027
1.54924	2.289918	0.7391937	1.870403	1.893954	3.550802	1.516073
6.292198	10.14117	7.780981	10.0176	14.03112	18.67012	14.40186
0.4551048	0.6561279	0.242881	0.4183096	0.7558618	0.961072	0.4291643

TCGA-19-2620-01	TCGA-HW-A5KK-01	TCGA-06-0158-01	TCGA-06-5858-01	TCGA-HT-8110-01	TCGA-DU-A5TU-01
2.328518	6.834505	5.830629	5.683562	7.639533	6.030437
5.009789	7.503998	23.54158	11.56629	22.1992	8.909667
6.425705	3.424097	9.861939	8.636052	5.873593	11.09318
4.626089	2.884366	6.069217	6.600437	4.748363	2.599408
3.188535	4.694397	4.536136	4.234186	4.791838	6.138994
0.0913017	0.04765948	0.09940011	0.104556	0.08968496	0.1673029
0.8805051	2.838176	1.953159	1.62593	2.237964	1.236981
2.787995	12.62791	8.38062	11.7679	8.470352	6.917188
0.4507233	0.6592941	0.4394751	0.2240454	0.5108562	0.5899383
0.0361	0.4287055	0.2554634	0.09818409	0.2038994	0.1488382
1.966991	4.107075	2.434813	2.463394	4.459848	3.889625
0.0395891	0.02066551	0.1508522	0.02266814	0.05833211	0.1692689
7.325113	20.02023	24.78707	14.8658	32.72377	17.8493
2.470497	6.846327	6.132146	6.595867	9.934996	6.193287
0.006235485	0.006509845	0.01018285	0.007140696	0.01837521	0.003808673
8.411661	8.54073	9.483864	8.975358	5.758766	1.387384
1.893967	4.3268	2.66842	1.620307	3.764633	0.9799171
0.5688454	1.479897	2.831807	1.875474	1.973729	0.8238028
7.592025	19.07621	10.51512	6.295099	13.86663	4.323456
0.2003209	0.1045675	0.1883498	0.318034	0.3756588	0.1334806

TCGA-FG-5964-01	TCGA-06-0174-01	TCGA-14-0736-02	TCGA-HT-8018-01	TCGA-06-0157-01	TCGA-06-0686-01	TCGA-06-0743-01
6.460125	4.088506	9.25785	3.781677	5.351545	5.712323	6.437142
3.561711	6.928112	11.81422	10.39113	8.665855	11.77029	13.78493
0.8873017	6.028524	34.65655	9.987383	6.700969	3.831418	9.996494
1.210796	5.020557	5.862321	5.139261	4.84406	6.470912	4.906433
2.626386	2.193439	4.475272	5.134215	3.540526	2.669774	4.882072
0.1765052	0.02868555	0.2528878	0.02027271	0.02657104	0.05850262	0.05827217
1.282729	1.134227	1.999838	1.339231	1.409367	0.9873392	0.9764253
3.679492	4.046395	9.731337	6.40962	7.862439	4.96409	3.747769
0.2270687	0.2832202	0.773743	0.9458014	0.4122534	0.539528	0.4362446
0.02492458	0.09073639	1.109888	0.2685251	0.6933952	0.08096023	0.4953681
2.406082	2.127717	2.149418	1.731055	2.227037	6.583861	2.098078
0.02186685	0	0.1754465	0.03516159	0.0230428	0.1014687	0.1516035
5.200963	13.68619	44.85192	11.56412	14.76377	13.69814	10.65174
3.591428	2.731153	4.269479	4.038241	3.945573	2.929669	2.550826
0	0.003918182	0.04145057	0.06922663	0.1742092	0	0.03979721
3.384651	6.842292	8.953043	2.48292	7.191454	10.15599	7.1927
3.236829	1.260115	13.15799	4.116325	4.83751	1.570517	4.650327
0.572653	0.8128978	2.703916	1.637916	2.638087	1.140514	1.428811
2.560192	5.717532	6.615257	9.485772	4.565122	6.773973	8.321171
0.03520563	0.2574722	0.5548495	0.3194431	0.3391901	0.1867024	0.2498931

TCGA-32-2616-01	TCGA-28-5208-01	TCGA-HT-7693-01	TCGA-P5-A72U-01	TCGA-02-2483-01	TCGA-06-5408-01	TCGA-28-2510-01
5.290445	3.881304	3.248404	6.514058	5.888274	3.585012	4.162555
12.15661	9.582711	5.276356	7.010988	11.20223	9.436125	4.198318
2.534609	11.57605	8.332157	2.778523	5.135255	12.84259	3.695221
9.653303	3.60689	2.63328	2.42245	4.177172	5.899488	4.168081
2.916889	3.420822	2.459768	5.81869	2.289865	5.98449	3.334103
0.06482485	0.2143476	0	0	0.02537945	0.05082561	0.06000541
1.364943	1.634339	1.116523	0.7293386	1.76837	0.6433316	0.8824971
7.170564	10.9113	5.507702	3.389674	6.92689	5.953966	4.183548
0.1781778	0.2790741	0.5438626	0.1499594	0.4268091	0.2784798	0.7128929
0.1238844	0.4078662	0.2012156	0.3866676	0.1003484	0.1456964	0.2728454
2.214967	2.224592	1.917394	2.442274	3.090881	1.549971	1.47574
0	0.02323568	0	0.2035389	0	0.06611509	0.07805639
4.457021	17.90598	12.23422	3.056763	20.42686	7.721652	11.26732
3.55028	4.986313	4.349028	2.795318	3.761563	2.276187	2.883726
0.008854477	0.2049453	0.02432891	0.003562046	0.0207996	0.006942309	0.008196186
6.57489	4.208155	5.815439	10.61207	6.782223	4.953534	4.995175
3.005867	2.367074	2.546019	11.634	3.580014	1.835772	1.713369
1.71108	1.852426	1.012563	1.200238	0.8008213	1.567995	0.6572652
8.835379	6.533117	8.374257	2.282619	10.96764	8.17934	5.567388
0.1379193	0.3313408	0.1725587	0.0312093	0.4150981	0.4561942	0.4607929

TCGA-HT-A61C-01	TCGA-06-0745-01	TCGA-06-0187-01	TCGA-06-0125-02	TCGA-FG-6692-01	TCGA-DU-A5TP-01
5.164076	8.866196	8.162989	5.152157	3.372013	17.77077
20.89113	12.65227	9.553465	8.91301	9.6987	49.23419
3.63506	24.95116	14.54083	7.024434	2.456609	14.45873
6.729446	7.455486	7.687641	6.560484	4.766705	11.00508
3.507044	5.898744	4.35221	3.65918	4.786291	3.89704
0.1328141	0.07231137	0.2216131	0.0275335	0.04154046	0
1.165571	1.731788	0.8882803	0.657188	0.6710257	1.462376
6.331074	4.32502	9.117743	12.20225	3.047725	19.80815
0.3170199	0.4785818	0.3005558	0.4122496	0.1374646	0.5618605
0.04201095	0.20967	0.2628724	0.1905144	0.2381594	0.0905
2.576501	3.670588	1.929381	1.809327	2.464155	3.264882
0	0.08361268	0.9369077	0.1671422	0.05403677	0.02481173
17.60826	31.3392	20.61719	20.00639	5.164629	73.19356
3.232075	3.151113	4.570722	3.467163	1.942816	8.947278
0.003628236	0.01975413	0.03783788	0.02632575	0.002837022	0.003907973
7.218277	10.5104	4.051653	7.218601	4.508529	2.080582
5.756542	3.611838	4.056311	3.440376	12.37832	2.010931
1.558873	0.9737222	1.575598	1.173143	1.014381	3.898644
4.390993	11.3929	8.770538	12.21524	2.991927	16.47826
0.1112623	0.4903884	0.9724621	0.6974607	0.07042793	1.420968

TCGA-E1-A7YE-01	TCGA-28-5215-01	TCGA-26-5134-01	TCGA-DB-A75K-01	TCGA-06-0238-01	TCGA-S9-A7R7-01	TCGA-HT-7471-01
3.636874	6.020661	4.753648	5.921088	7.63334	4.629193	4.893369
32.43405	12.9089	11.58656	25.48817	13.19216	8.245494	10.3913
9.062184	12.43919	7.376165	4.952995	15.51131	11.64349	7.234499
15.81734	8.627117	3.788795	3.084068	4.499451	1.894363	1.184949
8.750765	2.208534	2.946743	4.384713	3.080917	3.756874	2.630395
0.04111279	0.02305385	0	0.04627478	1.759865	0.1009467	0.0816303
1.516566	1.306184	0.9977034	0.9650582	2.063965	0.8112668	0.8725189
4.194001	7.525669	5.525524	5.177014	8.229773	9.483958	7.235356
1.521076	0.5127635	0.2905173	0.3012419	0.7443462	0.6717508	0.2538916
0.01625569	0.2916902	0.07218536	0.02744505	0.3774034	0.5122238	0.24207
4.545928	5.474697	1.652296	1.61604	5.422614	2.840124	1.525753
0	0.09996324	0	0.1003255	12.15772	0.02918082	0.02359701
66.53667	24.11701	5.055554	15.73912	29.54899	33.66894	8.987704
8.563182	5.854295	3.136503	3.21332	4.22772	9.882997	4.133331
0.01684688	0.08187254	0.01662459	0.01264142	0.03666835	0.05515353	0.09663288
0.8120594	8.593531	9.715953	0.4048251	3.139505	1.996197	2.015916
0.8628791	4.287193	2.910919	0.7001815	3.974611	2.38146	1.952325
0.5701376	2.437154	1.663382	0.5580194	1.522702	1.880049	1.585922
49.20812	17.29625	7.396443	6.862561	8.697874	5.797984	1.78967
1.627766	0.8460888	0.121382	0.166139	0.3629208	0.3355794	0.331066

TCGA-HT-7601-01	TCGA-DU-6402-01	TCGA-06-5856-01	TCGA-DU-A7TD-01	TCGA-12-5299-01	TCGA-14-0817-01
7.710833	7.666014	4.991762	6.94112	6.643927	14.61816
11.84976	12.84713	7.768304	21.74905	14.4518	23.0506
31.35585	11.89408	8.768878	3.501509	22.79498	24.93759
0.8174465	8.792221	5.588826	7.013326	8.476825	11.71185
3.508973	4.245779	2.863902	3.988233	3.713226	5.171657
0.04427624	0.05955008	0.07892262	0.1146294	0.2752368	0.3482871
0.950066	0.8231568	1.433447	1.775669	0.9410907	4.079602
18.16855	5.085154	8.317652	10.95789	8.134186	11.80395
0.624502	0.2067531	0.302556	0.3762192	0.1981785	0.6990832
0.3282467	1.181207	0.1612279	0.1359707	0.03957329	0.2639444
3.702278	2.925177	2.267063	1.759137	1.895326	3.785986
0	0.1032854	0.1140715	0.02485207	0.7811656	0.05033997
43.2571	4.608267	8.846757	6.530625	25.57034	39.88317
15.32645	3.872475	4.067688	6.391041	3.947267	6.309056
0.04233409	0.02169064	0.003593367	0.003914328	0	0
1.546339	15.22183	6.889993	9.969346	5.342418	11.67191
6.180753	9.330221	1.926088	2.81149	1.795295	7.692377
3.701841	2.54129	2.194229	2.298066	1.106344	1.685806
2.818031	4.715979	8.394949	5.289071	11.25725	7.918959
0.9317038	0.07522623	0.2203861	0.1257515	1.028101	0.5846988
