# Supplementary material for: HK3 stimulates immune cell infiltration to promote glioma deterioration
Source: Cancer Cell Int. 2023 Oct 1;23:227. doi: 10.1186/s12935-023-03039-w (PMC10543879; doi:10.1186/s12935-023-03039-w)
Supplement: Supplementary file 7 — Supplementary Figures: Supplementary Figure S1. The correlation between HK3 expression and immune cells infiltration levels in LGG and GBM from CGGA datasets. A. Pearson’s correlation coefficients between HK3 mRNA expression and immune scores in LGG and GBM datasets. B. Immune cells infiltration in LGG and GBM samples with low and high HK3 expression levels. Supplementary Figure S2. RNA-seq analysis between GL261 Hk3-OE and NC groups. A. Volcano map of differential expression genes. B. Heatmap of differential immune related cytokines. C. KEGG enrichment analysis of intracellular transduction signaling pathway. Supplementary Figure S3. HK3 expression in stratified glioma. A. HK3 expression in different tumor grades in the CGGA datasets. B. HK3 expression in LGG stratified according to IDH mutation status in CGGA datasets. C and D. HK3 expression in GBM according to IDH mutation status and MGMT methylation status in CGGA datasets. Supplementary Figure S4. Survival analysis of HK3 in glioma. A. Kaplan-Meier survival analysis of LGG and GBM in CGGA datasets. In GBM group, the high HK3 expression patients is 119 and low HK3 expression patients is 118. In LGG group, the high HK3 expression patients is 210 and low HK3 expression patients is 210. B. Timedependent ROC curve analysis of the efficiency of HK3 expression, patient age at diagnosis, and tumor grade in predicting 1-year, 3-years, 5-years OS in the CGGA datasets. C. Univariable and multivariable Cox regression analyses of HK3 expression and several other clinical factors in the CGGA datasets. Supplementary Figure S5. Validation of Hk3-overexpression (Hk3-OE) in in GL261 cells. A and B. PCR and qPCR were used to examine whether the Hk3 expression level was high in the GL261 Hk3-OE groups. All the experiments were repeated three times. C. Western blotting assays revealed that the Hk3 expression level was high in the GL261 Hk3-OE groups. All the experiments were repeated three times. D. The results of the immunofluore [file 12935_2023_3039_MOESM7_ESM.pdf]

Supplementary Figures

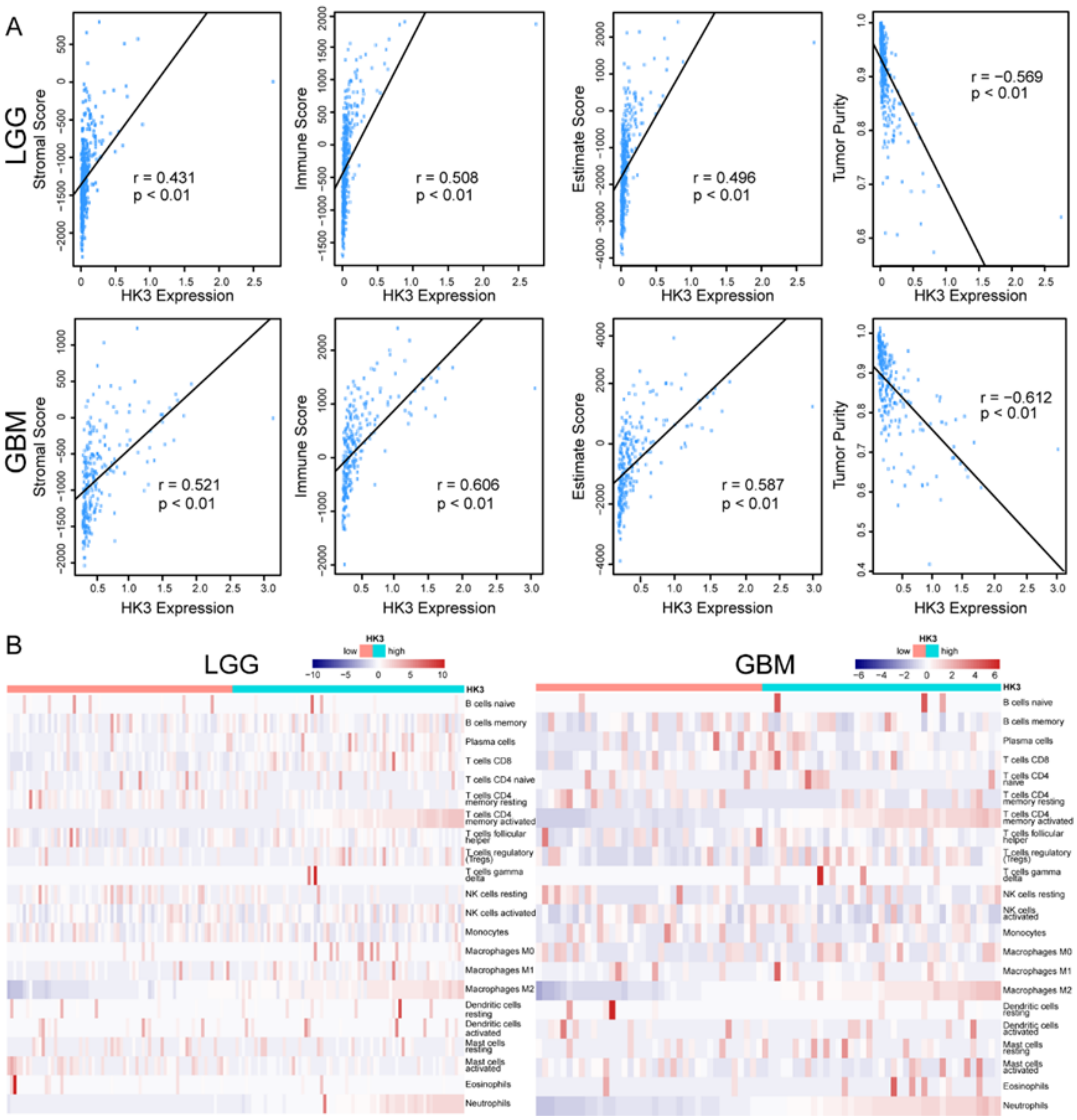

Supplementary Figure S1. The correlation between HK3 expression and immune cells infiltration levels in LGG and GBM from CGGA datasets. A. Pearson's correlation coefficients between HK3 mRNA expression and immune scores in LGG and GBM datasets. B. Immune cells infiltration in LGG and GBM samples with low and high HK3 expression levels.

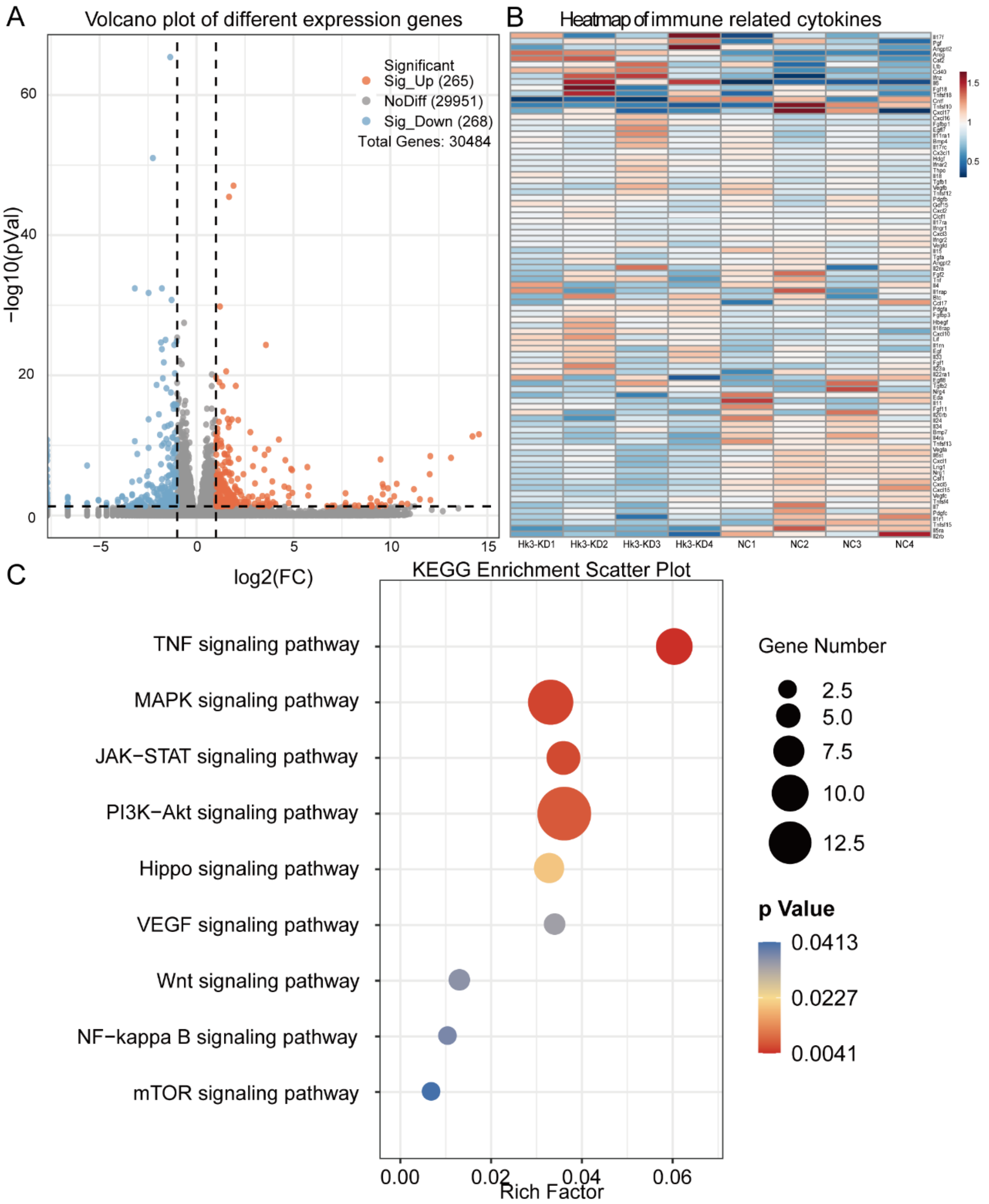

Supplementary Figure S2. RNA-seq analysis between GL261 Hk3-OE and NC groups. A. Volcano map of differential expression genes. B. Heatmap of differential immune related cytokines. C. KEGG enrichment analysis of intracellular transduction signaling pathway

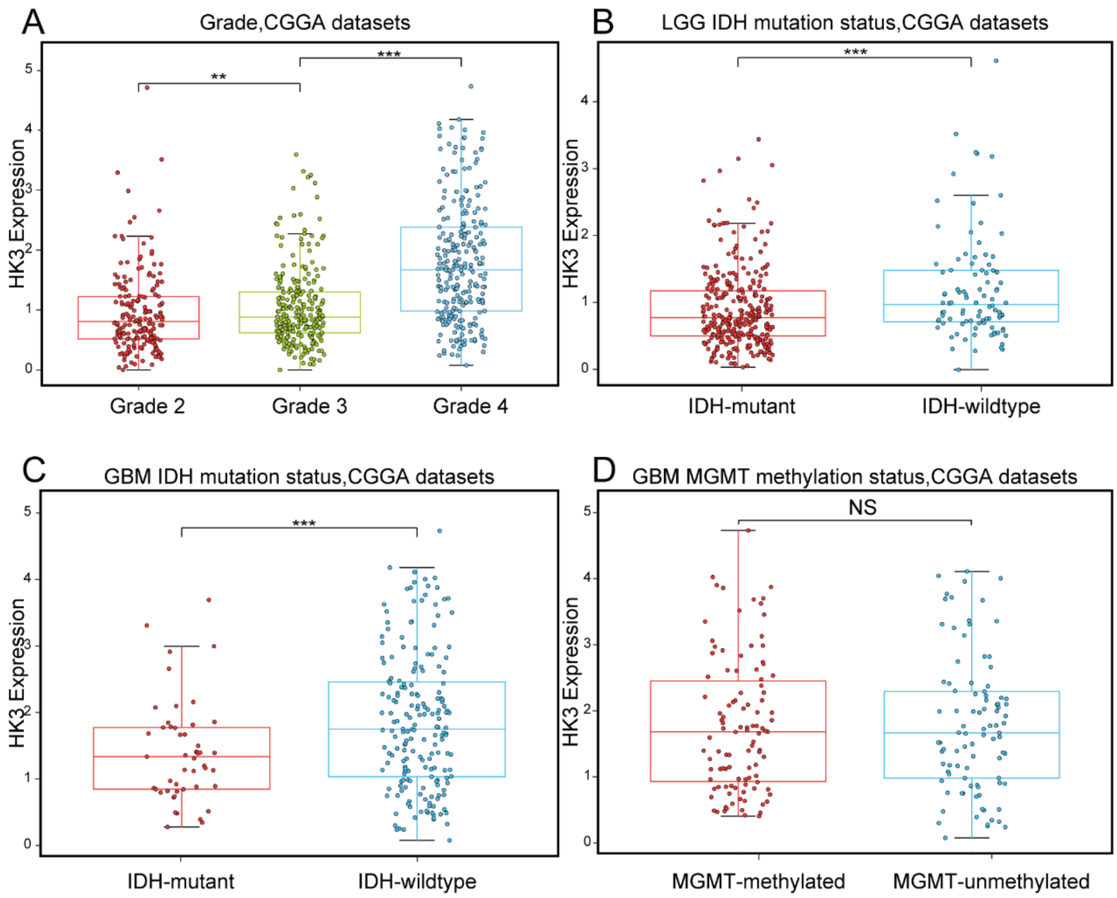

Supplementary Figure S3. HK3 expression in stratified glioma. A. HK3 expression in different tumor grades in the CGGA datasets. B. HK3 expression in LGG stratified according to IDH mutation status in CGGA datasets. C and D. HK3 expression in GBM according to IDH mutation status and MGMT methylation status in CGGA datasets.

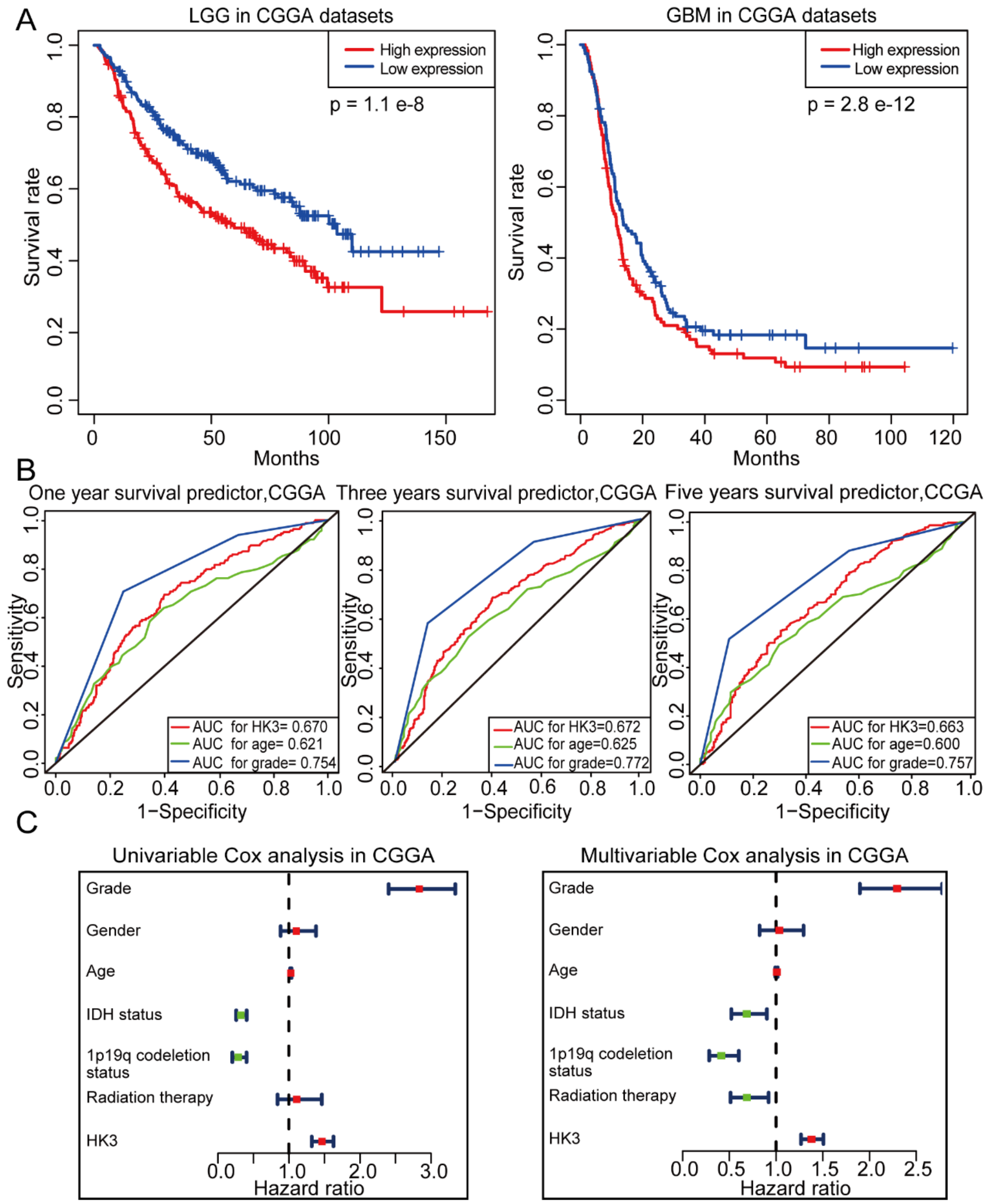

Supplementary Figure S4. Survival analysis of HK3 in glioma. A. Kaplan-Meier survival analysis of LGG and GBM in CGGA datasets. In GBM group, the high HK3 expression patients is 119 and low HK3 expression patients is 118. In LGG group, the high HK3 expression patients is 210 and low HK3 expression patients is 210. B. Time-dependent ROC curve analysis of the efficiency of HK3 expression, patient age at diagnosis, and tumor grade in predicting 1-year, 3-years, 5-years OS in the CGGA

datasets. C. Univariable and multivariable Cox regression analyses of HK3 expression and several other clinical factors in the CGGA datasets.

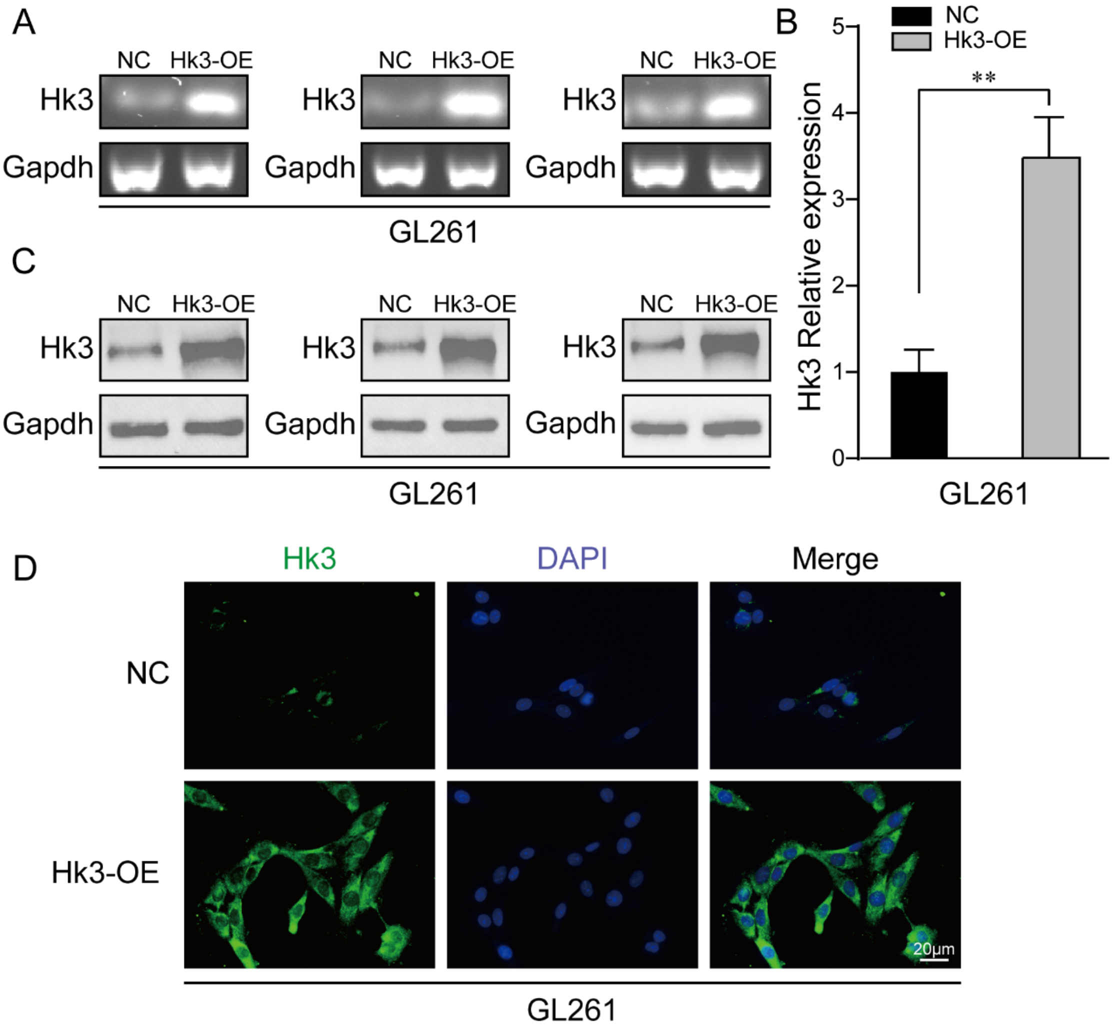

Supplementary Figure S5. Validation of Hk3-overexpression (Hk3-OE) in in GL261 cells. A and B. PCR and qPCR were used to examine whether the Hk3 expression level was high in the GL261 Hk3-OE groups. All the experiments were repeated three times. C. Western blotting assays revealed that the Hk3 expression level was high in the GL261 Hk3-OE groups. All the experiments were repeated three times. D. The results of the immunofluorescence assay illustrate that the Hk3 expression level is high in the GL261 Hk3-OE groups. Scale bar = 20 μm. The data are presented as the mean ± S.D. Significant results are presented as $^{**}p < 0.01$.

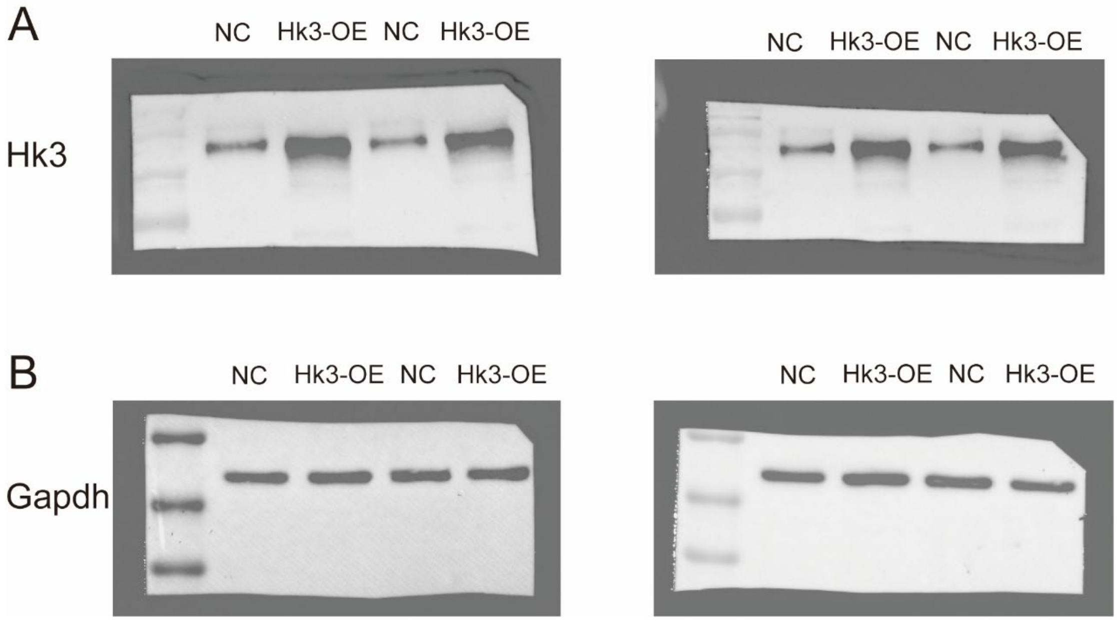

Supplementary Figure S6. The original blots for figure S7. A. The original blots of Hk3. B. The original blots of Gapdh.

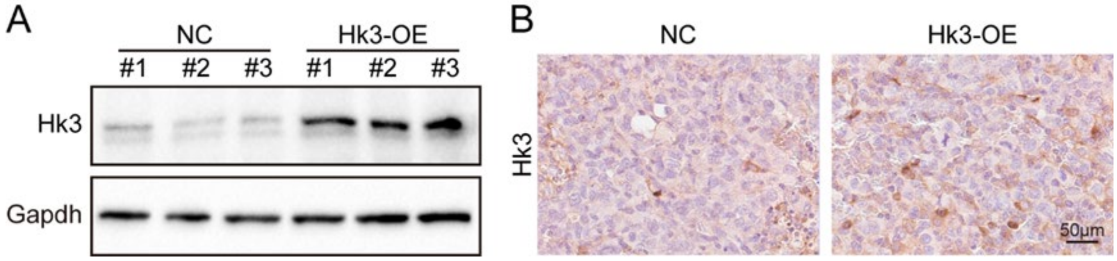

Supplementary Figure S7. Validation of Hk3-overexpression (Hk3-OE) in brain sections from mice tumors derived from GL261-Luc cells. A. Western blotting assays revealed that the Hk3 expression level was high in the GL261 Hk3-OE groups. B. IHC staining for Hk3 in brain sections from mice tumors derived from GL261-Luc cells.

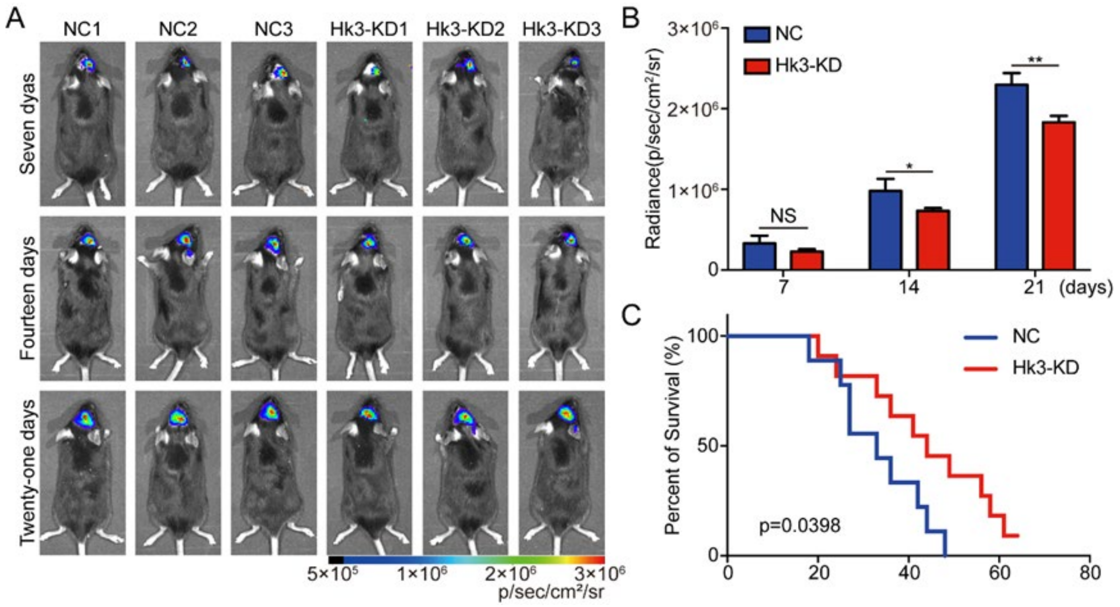

Supplementary Figure S8. Knock-down of Hk3 in GL261 cells suppress orthotopic tumor growth in vivo. A. Bioluminescence images of C57BL/6N mice. B. Quantification of the signal intensities revealed by bioluminescence imaging of C57BL/6N mice. C. Kaplan–Meier survival curve of C57BL/6N mice is shown N=10.

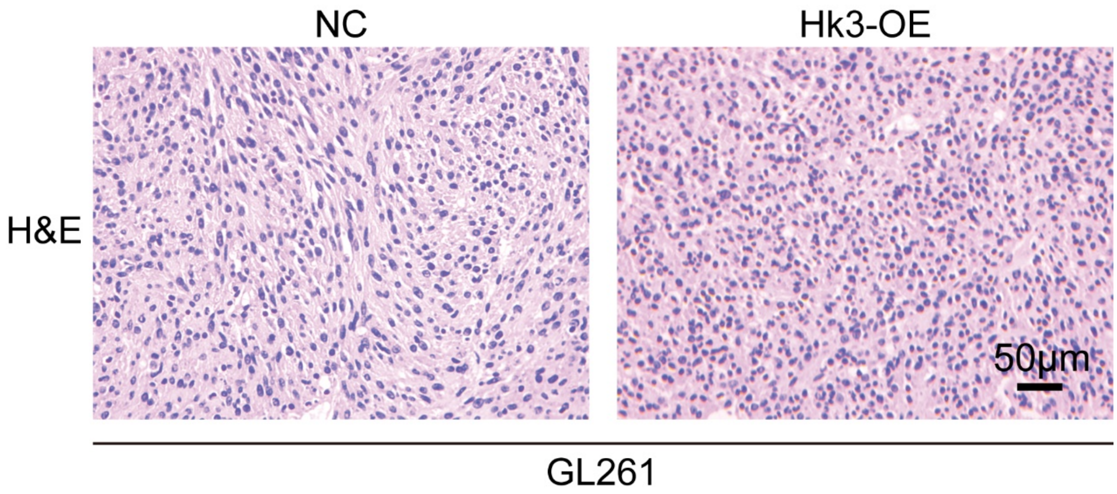

Supplementary Figure S9. H&E staining for Hk3 NC group and Hk3-OE group. Scale bar = 50 μm.

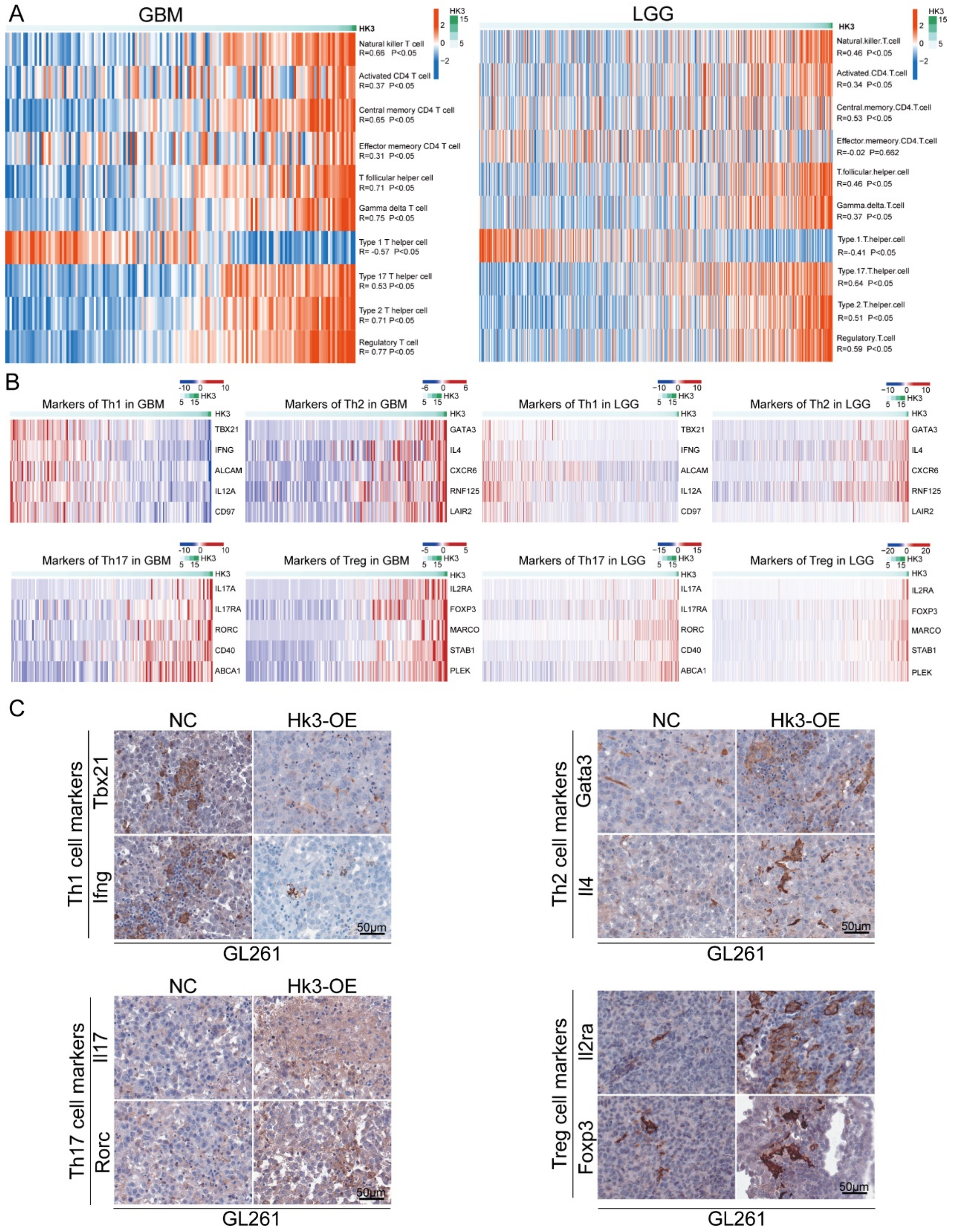

Supplementary Figure S10. Correlation between HK3 expression and markers of CD4^{+} T cell in glioma. A. Correlation between HK3 expression and subtypes of CD4^{+} T cell in LGG and GBM samples in TCGA datasets. B. Correlation between HK3 expression and markers of CD4^{+} T cell in LGG and GBM samples in TCGA datasets.

C. IHC staining for markers of $CD4^+$ T cell in brain sections from mice tumors derived from GL261-Luc cells. Scale bar = 50 μm.
